# SIRT1 promotes lipid metabolism and mitochondrial biogenesis in adipocytes and coordinates adipogenesis by targeting key enzymatic pathways

**DOI:** 10.1038/s41598-021-87759-x

**Published:** 2021-04-14

**Authors:** Yasser Majeed, Najeeb Halabi, Aisha Y. Madani, Rudolf Engelke, Aditya M. Bhagwat, Houari Abdesselem, Maha V. Agha, Muneera Vakayil, Raphael Courjaret, Neha Goswami, Hisham Ben Hamidane, Mohamed A. Elrayess, Arash Rafii, Johannes Graumann, Frank Schmidt, Nayef A. Mazloum

**Affiliations:** 1 Department of Microbiology and Immunology, Weill Cornell Medicine-Qatar, Qatar Foundation, Doha, Qatar; 2 Department of Genetic Medicine, Weill Cornell Medicine-Qatar, Qatar Foundation, Doha, Qatar; 3 College of Health and Life Sciences, Hamad Bin Khalifa University, Qatar Foundation, Doha, Qatar; 4 Department of Biochemistry, Weill Cornell Medicine-Qatar, Qatar Foundation, Doha, Qatar; 5 Biomolecular Mass Spectrometry, Max-Plank Institute for Heart and Lung Research, Ludwigstr 43, 61231 Bad Nauheim, Germany; 6 Neurological Disorders Research Center, Qatar Biomedical Research Institute, Qatar Foundation, Doha, Qatar; 7 Interim Translational Research Institute, Hamad Medical Corporation, Doha, Qatar; 8 Department of Physiology and Biophysics, Weill Cornell Medicine-Qatar, Qatar Foundation, Doha, Qatar; 9 CSL Behring, Bern, Switzerland; 10 Biomedical Research Center, Qatar University, Doha, Qatar

**Keywords:** Metabolism, Endocrinology, Endocrine system and metabolic diseases

## Abstract

The NAD^+^-dependent deacetylase SIRT1 controls key metabolic functions by deacetylating target proteins and strategies that promote SIRT1 function such as SIRT1 overexpression or NAD^+^ boosters alleviate metabolic complications. We previously reported that SIRT1-depletion in 3T3-L1 preadipocytes led to C-Myc activation, adipocyte hyperplasia, and dysregulated adipocyte metabolism. Here, we characterized SIRT1-depleted adipocytes by quantitative mass spectrometry-based proteomics, gene-expression and biochemical analyses, and mitochondrial studies. We found that SIRT1 promoted mitochondrial biogenesis and respiration in adipocytes and expression of molecules like leptin, adiponectin, matrix metalloproteinases, lipocalin 2, and thyroid responsive protein was SIRT1-dependent. Independent validation of the proteomics dataset uncovered SIRT1-dependence of SREBF1c and PPARα signaling in adipocytes. SIRT1 promoted nicotinamide mononucleotide acetyltransferase 2 (NMNAT2) expression during 3T3-L1 differentiation and constitutively repressed NMNAT1 and 3 levels. Supplementing preadipocytes with the NAD^+^ booster nicotinamide mononucleotide (NMN) during differentiation increased expression levels of leptin, SIRT1, and PGC-1α and its transcriptional targets, and reduced levels of pro-fibrotic collagens (Col6A1 and Col6A3) in a SIRT1-dependent manner. Investigating the metabolic impact of the functional interaction of SIRT1 with SREBF1c and PPARα and insights into how NAD^+^ metabolism modulates adipocyte function could potentially lead to new avenues in developing therapeutics for obesity complications.

## Introduction

The prevalence of obesity has reached epidemic proportions in the developing world and obese subjects are at an increased risk of developing type 2 diabetes (T2D), hypertension, and cancer^1,2^. Obesity is associated with increased visceral adiposity, which can occur by hypertrophy of existing adipocytes and by de novo adipogenesis through proliferation and differentiation of preadipocytes^3–5^. In addition to lipid storage, a critical function of adipocytes is to secrete key adipokines like leptin and adiponectin, which fulfill important physiological roles in metabolism^6–8^. However, obesity is associated with white adipose tissue (WAT) inflammation and secretion of pro-inflammatory cytokines like IL-6 and TNF-α by inflamed WAT promotes systemic inflammation and insulin-resistance^9^. A widely used in vitro model system to study adipogenesis is the 3T3-L1 preadipocyte cell-line, which was developed from murine Swiss 3T3 cells^10,11^. 3T3-L1 preadipocytes may be converted to mature adipocytes by incubating with fetal bovine serum and a chemical cocktail consisting of insulin, 3-isobutyl-1-methylxanthine (IBMX) and dexamethasone^10^. Mechanistic studies of 3T3-L1 adipocytes suggest a key requirement for transcription factors such as CEBPα/β/δ, PPARα, PPARγ and PGC-1α^12,13^, which regulate key aspects including fatty acid-oxidation, triglyceride synthesis and mitochondrial biogenesis^14–16^. 3T3-L1 adipocytes also secrete a wide array of molecules, including adipokines like leptin, adiponectin, and lipocalin-2 (LCN2)^17^, and extracellular matrix components like matrix metalloproteinases (MMP) and collagens^18^. Proteomics approaches have therefore provided useful insights into molecular changes associated with 3T3-L1 differentiation. For example, Adachi et al. discovered differential expression of proteins linked to insulin-signaling, proteasome-degradation and cytosolic ribosomal proteins by analyzing the proteome of specific cellular compartments in adipocytes^19^. Newton et al. profiled the mitochondrial proteome and reported changes in key enzymes involved in the TCA cycle^20^ and Jiang et al. profiled the proteome during the Mitotic Clonal Expansion (MCE) stage of 3T3-L1 differentiation and reported induction of PKM2, a putative target of CEBPβ^21^. Ojima et al. analyzed the secretory profile of 3T3-L1 adipocytes and discovered key components of the adipocyte ‘secretome’, including adiponectin, collagens, and growth factors^18^.

Sirtuin 1 (SIRT1) is a NAD^+^-dependent deacetylase with established roles in metabolism. In vitro and in vivo studies have highlighted a key role for SIRT1 in adipogenesis^22–24^ and its targets include transcription factors like PPARγ and PGC-1α^16,22^. Evidence in the literature also supports a role for SIRT1 in 3T3-L1 adipogenesis. For example, SIRT1 was shown to limit preadipocyte hyperplasia through C-Myc deacetylation^25^, improve insulin-sensitivity and reduce inflammation^26^, and suppress lipid accumulation by inhibiting PPARγ^27^. There is therefore significant interest in the identification of SIRT1-dependent molecular pathways relevant to adipogenesis and obesity. SIRT1 activity depends on NAD^+^, which is generated from its precursor—Nicotinamide Mononucleotide (NMN)—by enzymes called Nicotinamide Mononucleotide Adenylyltransferases (NMNATs). Three NMNAT isoforms have been discovered and they show distinct subcellular localizations: NMNAT1 (nucleus), NMNAT2 (cytosol) and NMNAT3 (mitochondria)^28^, which suggests a localization-component to NAD^+^ synthesis in response to metabolic signals. Importantly, several studies have demonstrated a decline in cellular NAD^+^ levels in obesity^29,30^ and strategies that replenished NAD^+^ by supplementation with precursors like NMN or Nicotinamide Riboside (NR) improved metabolic function^31–34^.

A previously unrecognized function of NAD^+^ in 3T3-L1 differentiation was suggested by the discovery that CEBPβ activity was suppressed by the NAD^+^-dependent enzyme Poly(ADP-ribose) polymerase-1 (PARP-1) via a post-translational modification termed PARylation. PARylation of CEBPβ inhibited its transcriptional activity and suppressed adipogenesis. Importantly, in the early stages of differentiation, CEBPβ activity was de-repressed by reduced PARP-1 activity, a consequence of depleted nuclear NAD^+^ levels^35^. Using NAD^+^-detection probes localized to specific cellular compartments, Ryu et al. reported that induction of the cytosolic NMNAT–NMNAT2—led to increased cytosolic NAD^+^ synthesis from NMN, which triggered a sequence of events culminating in depletion of nuclear NAD^+^ levels, inhibition of PARP-1 activity, and de-repression of CEBPβ to promote 3T3-L1 differentiation^36^. These data further support the hypothesis that compartmentalized NAD^+^ synthesis modulates molecular pathways underlying 3T3-L1 adipogenesis. However, the precise mechanisms underlying the induction of NMNAT2 during early stages of 3T3-L1 adipogenesis are yet to be discovered.

In this study, quantitative proteomics analysis was performed to identify SIRT1-dependent pathways in 3T3-L1 adipocytes. Pathway analysis of the dataset revealed SIRT1-dependence of key transcription factors, including PPARα, SREBF1/2, PGC-1α, PGC-1β, NFE2L2, and KLF15. SIRT1 promoted mitochondrial biogenesis and respiration in adipocytes and the expression of key molecules like leptin, adiponectin, matrix metalloproteinases 3/13 (MMP3/13), lipocalin 2 (LCN2), glutathione s-transferase A3 (GSTA3), and thyroid hormone responsive (THRSP) was SIRT1-dependent. SIRT1 promoted NMNAT2 expression during 3T3-L1 differentiation and constitutively repressed NMNAT1/3 expression in preadipocytes. Supplementation with NMN during differentiation increased the expression levels of leptin, SIRT1, and PGC-1α and its transcriptional targets, and reduced the expression of pro-fibrotic collagens, Col6A1 and Col6A3. Therefore, the data confirmed previous findings linking SIRT1 to key transcription factors like PGC-1α and identified a positive interaction of SIRT1 with SREBF1c and PPARα in adipocytes. SIRT1-dependent regulatory effects of NAD^+^ boosting on gene-expression during adipogenesis that are predicted to improve adipocyte metabolism were also discovered.

## Results

### SIRT1-depletion suppressed the expression of leptin, adiponectin, and metalloproteinases, and promoted the expression of pro-fibrotic collagens in 3T3-L1 adipocytes

**Figure 1:**
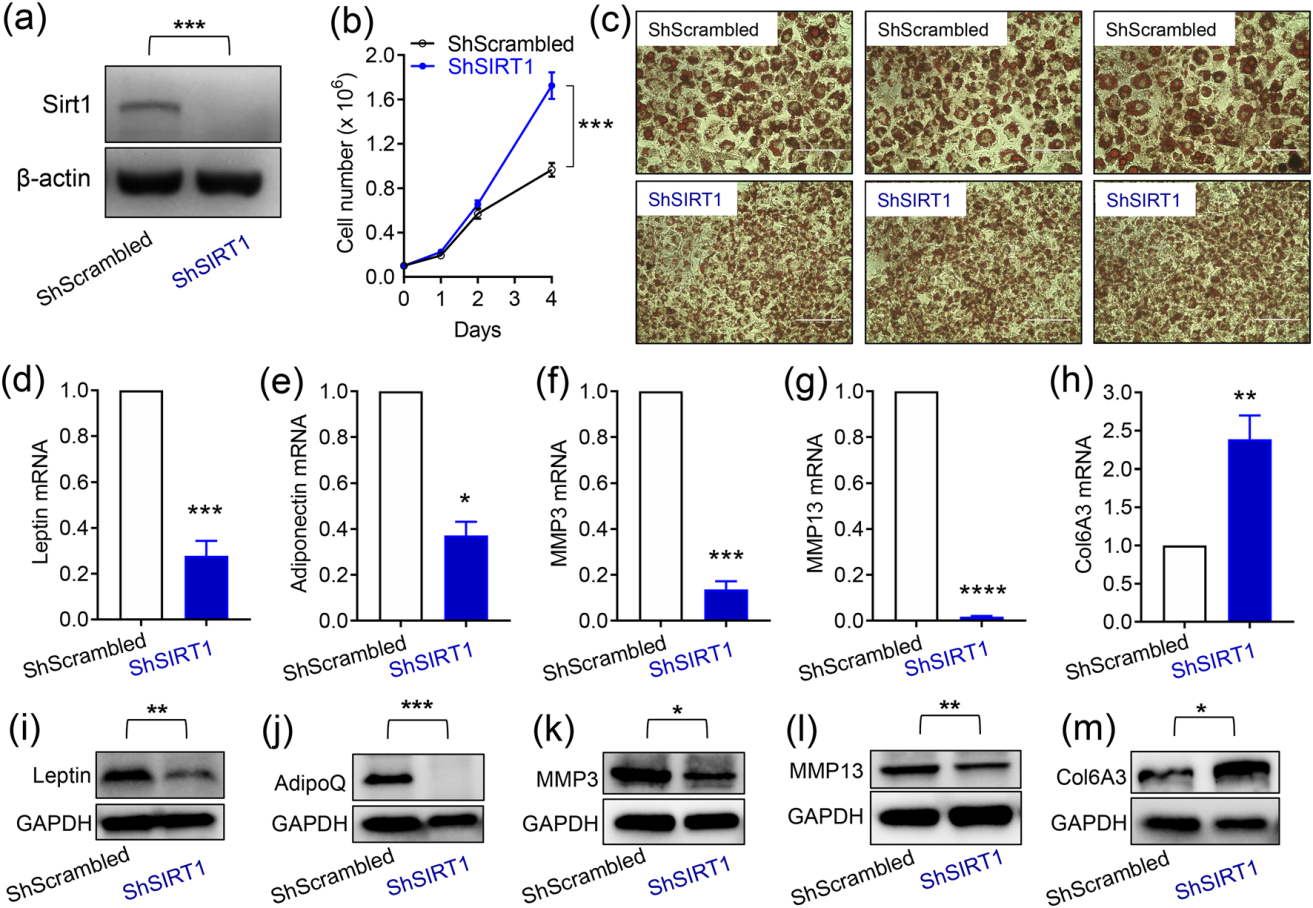
SIRT1-depletion suppressed the expression of key adipokines and metalloproteinases and promoted the expression of pro-fibrotic collagens in 3T3-L1 adipocytes. (**a**) Representative western blot data showing SIRT1 expression in 3T3-L1 preadipocytes infected with either Scrambled shRNA (ShScrambled) or SIRT1-specific shRNA (ShSIRT1). Expression of β-actin was monitored to control for equal loading. (**b**) Cell proliferation analysis of ShScrambled or ShSIRT1 preadipocytes (n = 6). (**c**) Representative images of Oil Red O (ORO)-stained Control (ShScrambled) or SIRT1-depleted (ShSIRT1) adipocytes at Day 6 post-differentiation. Scale bar 100 μm (Images representative of 3 independent experiments). (**d–h**) Quantitative gene-expression analysis of leptin (**d**), adiponectin (**e**), MMP3 (**f**), MMP13 (**g**), and collagen 6A3 (Col6A3) (**h**) in ShScrambled or SIRT1-depleted (ShSIRT1) adipocytes (n = 4). (**i–m**) Western blot data comparing the expression levels of the indicated proteins in ShScrambled or SIRT1-depleted (ShSIRT1) adipocytes (n = 3). Statistical analysis was performed using a Student’s t test (*P < 0.05; **P < 0.01; ***P < 0.001; ****P < 0.0001).

To investigate the function of SIRT1 in regulating adipocyte metabolism, we employed lentiviral technology to robustly down-regulate SIRT1 expression, as described previously^25^. As Control for the SIRT1-specific ShRNA, we used a lentiviral plasmid encoding a ‘scrambled’ sequence with no known complementarity to the mouse genome (ShScrambled). To confirm SIRT1-depletion, western blotting was performed on lysates prepared from ShScrambled and ShSIRT1 preadipocytes. These experiments demonstrated that, when compared to ShScrambled, ShSIRT1 robustly downregulated SIRT1 protein expression to undetectable levels (Fig. 1a; Supplementary Figure S1). As reported previously, depletion of SIRT1 triggered hyperplasia, assessed by cell counts (Fig. 1b) and microscopic comparison of Oil Red O-stained ShSIRT1 adipocytes vs ShScrambled adipocytes (Fig. 1c; Day 6 post-differentiation). Importantly, SIRT1-depleted adipocytes showed significantly lower expression levels of leptin, adiponectin, MMP3, and MMP13. In contrast, expression of the pro-fibrotic collagen, Collagen 6A3, was significantly higher (Fig. 1d–m).

### Mass spectrometry-based proteomics and downstream analyses uncovered inhibition of key pathways linked to lipid metabolism and mitochondrial function in SIRT1-depleted adipocytes

**Figure 2:**
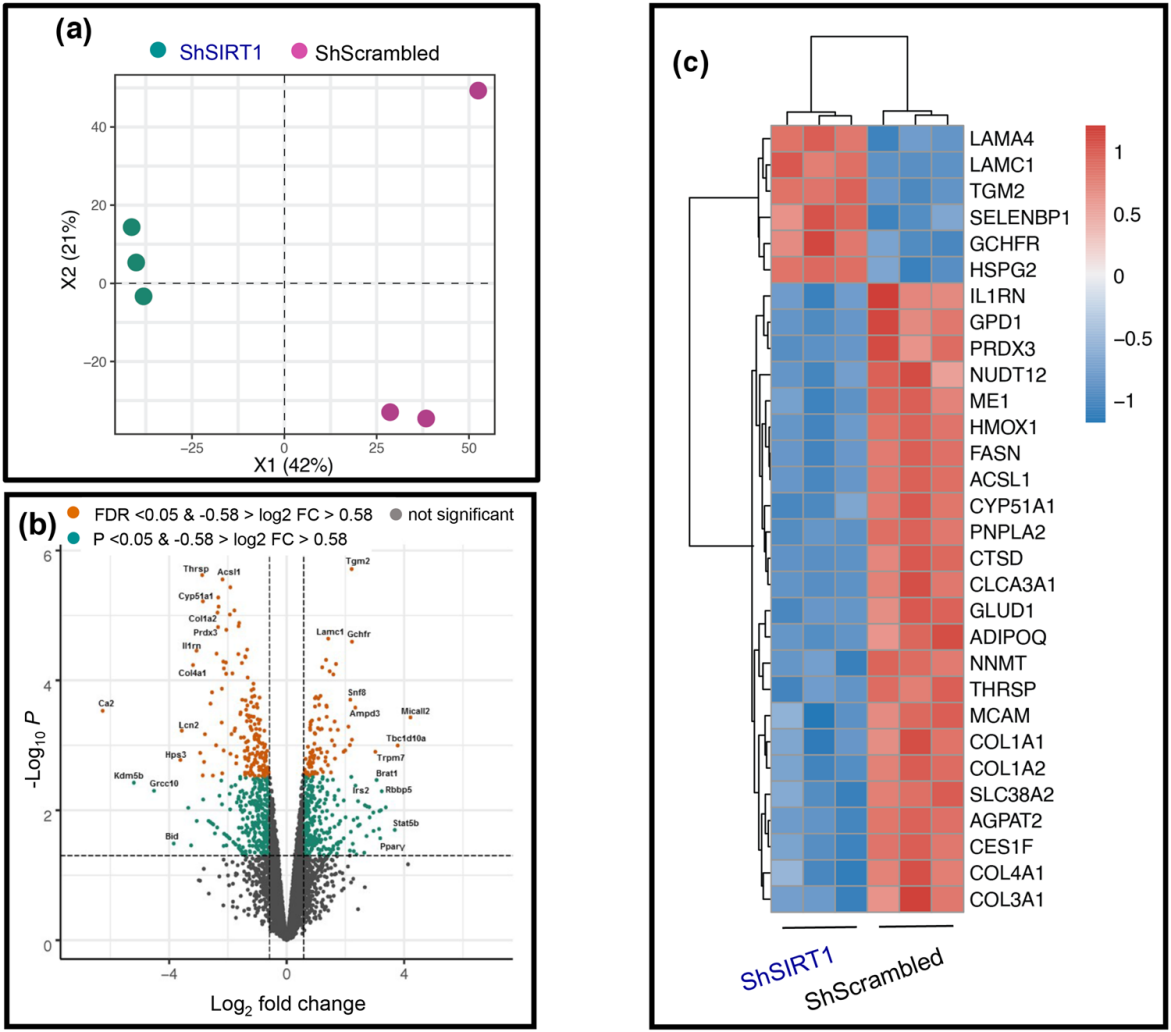
Quantitative proteomics identified altered expression levels of key molecules linked to adipocyte function in SIRT1-depleted adipocytes. (**a**) Principal component analysis (PCA) plot shows the relationship in overall proteomics profiles upon SIRT1-depletion in three replicates (ShSIRT1; green circles) compared to ShScrambled (purple circles). (**b**) Volcano plot analysis highlights the most significant protein alterations in SIRT1-depleted adipocytes *vs* ShScrambled adipocytes based on false discovery rate threshold (orange) [FDR q value < 0.05 and − 0.58 > log_2_ fold change (FC) > 0.58] and P value threshold (green) [P value < 0.05 and − 0.58 > log_2_ fold change (FC) > 0.58]. Highlighted in grey are the filtered non-significant changes, as indicated. (**c**) Heatmap clustering of the thirty most-significant protein alterations in SIRT1-depleted adipocytes *vs* ShScrambled adipocytes based on fold-change cut-off of > 1.5 and FDR values < 0.01. The color scale of the heatmap represents the log2 ratio of each protein in each replicate.

As described in “Materials and methods” section, a quantitative proteomics approach was employed to identify SIRT1-dependent proteome changes in 3T3-L1 adipocytes. Lysates from ShScrambled and ShSIRT1 adipocytes were subjected to dimethyl-labeling prior to separation by isoelectric-focusing, mass-spectrometry, and downstream bioinformatics analyses. Principal component analysis (PCA) was used to gain insights into the overall proteomics profile in ShScrambled *vs* ShSIRT1 adipocyte samples. Shown in Fig. 2a is the PCA plot representing the reproducible separation of ShScrambled and ShSIRT1 samples, compared to the internal standard. A Volcano plot was generated to visualize the distribution of log-ratio differences between ShScrambled and ShSIRT1 samples in relation to their significance. Plotted are log_2_ fold-changes of ShSIRT1 on the x-axis *vs* ShScrambled − log_10_ P value on the y-axis for 5273 identified proteins, using a log2 fold-change cutoff of − 0.58 and 0.58, corresponding to fold-change values of 0.67 and 1.50, respectively (Fig. 2b). Of 5273 proteins that were identified, 4514 were quantified and—using a cut-off filter of P < 0.05 and FC 1.5–351 significantly upregulated proteins and 465 downregulated proteins were discovered. The full dataset is included in Supplementary Table S2. Molecules whose expression was most-significantly altered in SIRT1-depleted adipocytes are highlighted, based on a false discovery rate threshold (orange) [FDR q-value < 0.05 and − 0.58 > log_2_ fold change (FC) > 0.58] and P value threshold (green) [P value < 0.05 and − 0.58 > log_2_ fold change (FC) > 0.58]. The most-significantly upregulated or downregulated proteins were identified and chosen for further validation and downstream characterization (Fig. 2b,c). Differentially expressed molecules broadly related to the following functional categories: (**a**) extracellular matrix components such as laminin α4 (Lama4), laminin C1 (Lamc1), and collagens (Col1a1, 1a2, 3a1, 4a1); (**b**) enzymes linked to lipid metabolism such as fatty acid synthase (Fasn), adipose triglyceride lipase (ATGL/PnPLA2), Acyl-CoA Synthetase Long Chain Family Member 1 (ACSL1), and 1-Acylglycerol-3-Phosphate O-acyltransferase 2 (AGPAT2); and (**c**) secreted factors such as adiponectin and Interleukin 1 Receptor Antagonist (IL1RN) (Fig. 2c).

**Figure 3:**
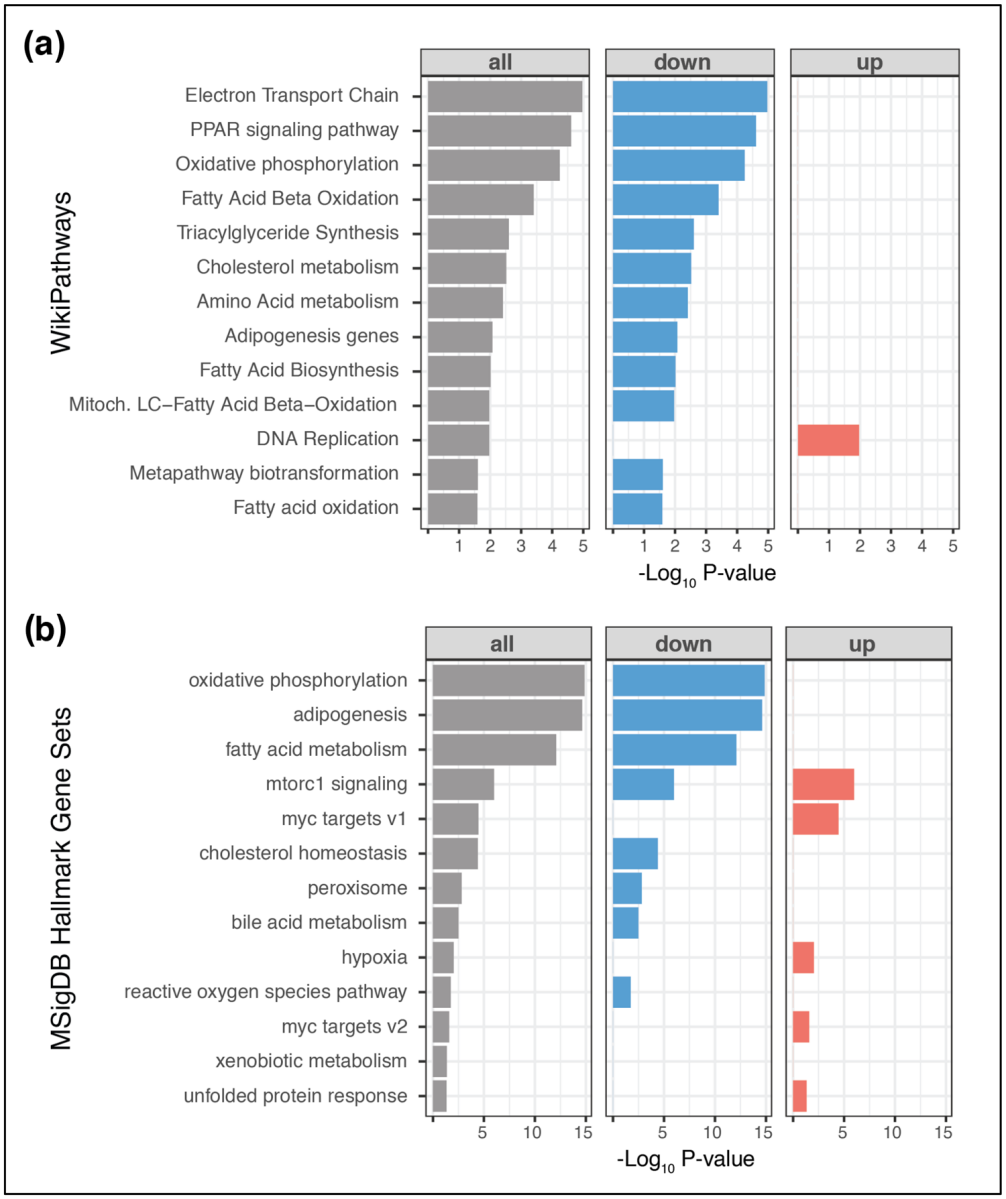
Identification of significantly-altered molecular pathways in SIRT1-depleted adipocytes. Prediction of significantly-affected molecular pathways by “WikiPathways” analysis (**a**) or MSigDB Hallmark gene-sets (**b**) in SIRT1-depleted adipocytes. Annotations from WikiPathways analysis or MSigDB Hallmark gene-sets were used for functional enrichment analysis among differentially expressed proteins (P < 0.05). Significantly enriched pathways (P < 0.05) were used for plotting. Axes show p-value of overlap and highlighted in red and blue are the up-regulated and down-regulated protein sets, respectively.

To identify molecular pathways that were regulated by SIRT1 during adipogenesis, the proteomics dataset was subjected to WikiPathways analysis. Annotations from WikiPathways were used for functional enrichment analysis of differentially expressed proteins (P < 0.05). Differential proteins (all, down- or upregulated) were analyzed separately against the complete dataset using Fisher’s exact test for overrepresentation of gene groups as annotated by WikiPathways. The most-significantly enriched pathways (P < 0.05) are shown in Fig. 3a. Importantly, molecular pathways that were predicted to be significantly inhibited in SIRT1-depleted adipocytes included PPAR signaling, oxidative phosphorylation, fatty acid β-oxidation, triglyceride synthesis, adipogenesis, fatty acid biosynthesis, mitochondrial long chain fatty acid β-oxidation, electron transport chain, cholesterol metabolism, amino acid metabolism, and metapathway biotransformation (Fig. 3a). The most-significantly upregulated molecular pathway was DNA replication, which was consistent with the hyperplastic phenotype observed in SIRT1-depleted adipocytes. To support the WikiPathways analysis, MSigDB hallmark gene-sets were used for functional enrichment analysis of differentially expressed proteins (P < 0.05). Significantly enriched hallmark gene sets (P < 0.05) are plotted in Fig. 3b. Consistent with the WikiPathways analysis, the most-significantly downregulated hallmark gene-sets in SIRT1-depleted adipocytes included oxidative phosphorylation, adipogenesis, fatty acid metabolism, cholesterol homeostasis, peroxisome metabolism, bile acid metabolism, and reactive oxygen species pathway. In contrast, the most-significantly upregulated hallmark gene-sets included C-Myc transcriptional targets, hypoxia, and unfolded protein response (UPR) (Fig. 3b).

### SIRT1 promoted mitochondrial biogenesis and respiration in 3T3-L1 adipocytes

**Figure 4:**
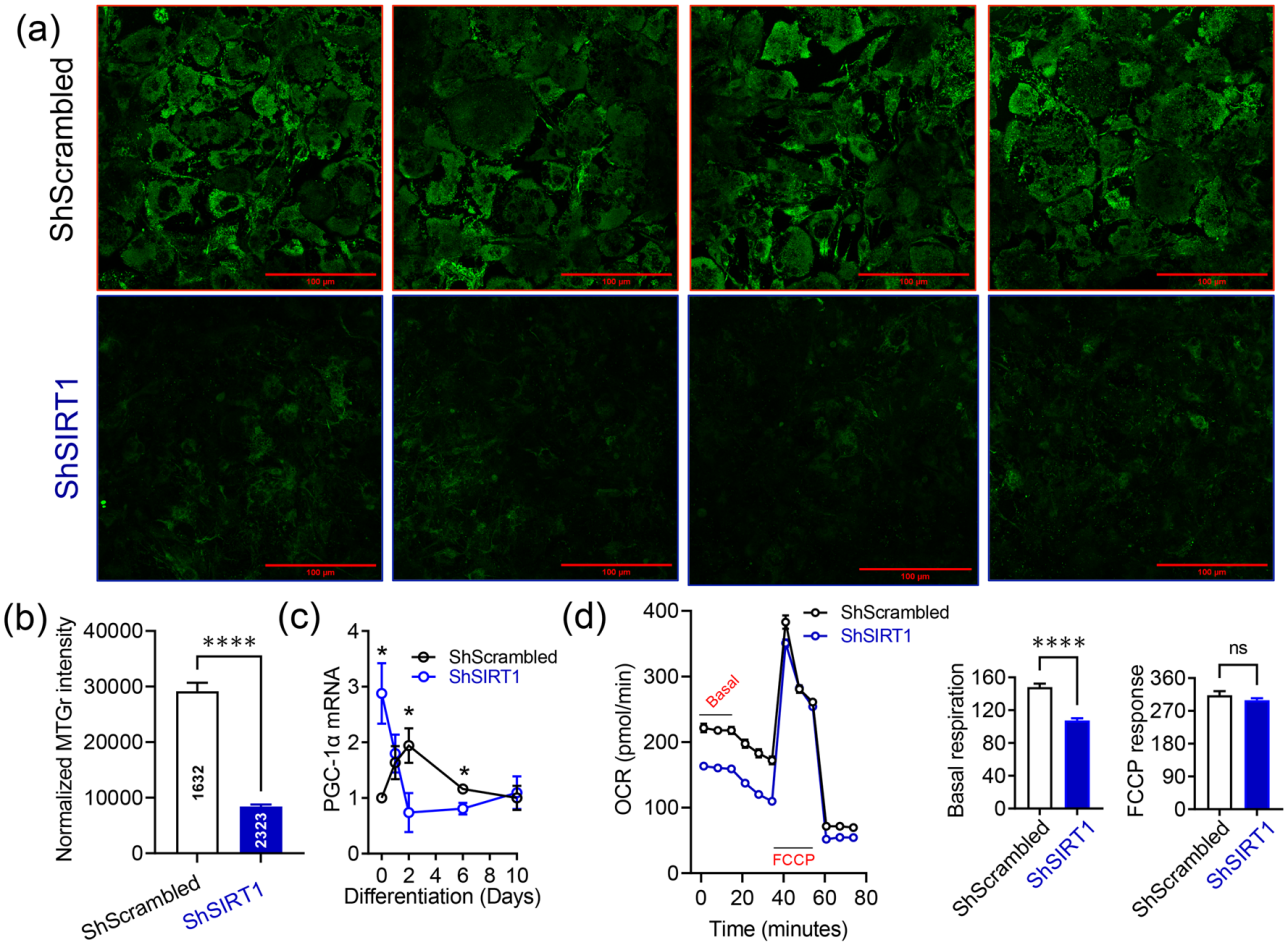
SIRT1 promoted mitochondrial biogenesis and respiration in 3T3-L1 adipocytes. (**a**) Representative confocal microscopy images of ShScrambled or SIRT1-depleted (ShSIRT1) adipocytes labelled with 250 nM MitoTracker Green at Day 6 post-differentiation. Scale bar 100 µm. (**b**) Quantification of MitoTracker Green staining intensity in ShScrambled or SIRT1-depleted (ShSIRT1) adipocytes (normalized to cell number) (n = 3). (**c**) Quantification of changes in the expression of PGC-1α in ShScrambled or SIRT1-depleted (ShSIRT1) (pre)adipocytes at specific time-points during adipogenesis (n = 4). (**d**) Evaluation of mitochondrial respiration parameters in ShScrambled or SIRT1-depleted (ShSIRT1) adipocytes using Seahorse technology at Day 6 post-differentiation (n = 12 biological replicates). Statistical analysis of the dataset was performed using a Student’s t test. For statistical analysis of data shown in (**c**), pair-wise comparisons were made between ShScrambled and ShSIRT1 groups for each individual time-point using the Student’s t test (*P < 0.05; ****P < 0.0001; n.s., not significant).

To functionally validate the proteomics discovery and to gain insights into how SIRT1 impacted on mitochondrial function in adipocytes, experiments were performed to quantify mitochondrial mass in ShScrambled and SIRT1-depleted (ShSIRT1) adipocytes. At Day 6 post-differentiation, adipocyte mitochondria were labelled with 250 nM MitoTracker Green and analyzed by confocal microscopy. These experiments revealed that the intensity of MitoTracker Green fluorescence was significantly lower in SIRT1-depleted adipocytes (Fig. 4a,b). Consistent with this observation, expression levels of a key transcription factor that promotes mitochondrial biogenesis—PGC-1α—were significantly reduced in SIRT1-depleted adipocytes (Fig. 4c). Furthermore, assays to evaluate mitochondrial respiration revealed that basal respiration was significantly lower in SIRT1-depleted (ShSIRT1) adipocytes when compared to ShScrambled adipocytes. In contrast, the response elicited by mitochondrial depolarization using the potent uncoupling agent FCCP (trifluoromethoxy carbonylcyanide phenylhydrazone) was unaffected by SIRT1-depletion (Fig. 4d). Collectively, these data suggested a positive impact of SIRT1 signaling on adipocyte mitochondrial function.

### SIRT1-dependence of SREBF1c and PPARα signaling in 3T3-L1 adipocytes

**Figure 5:**
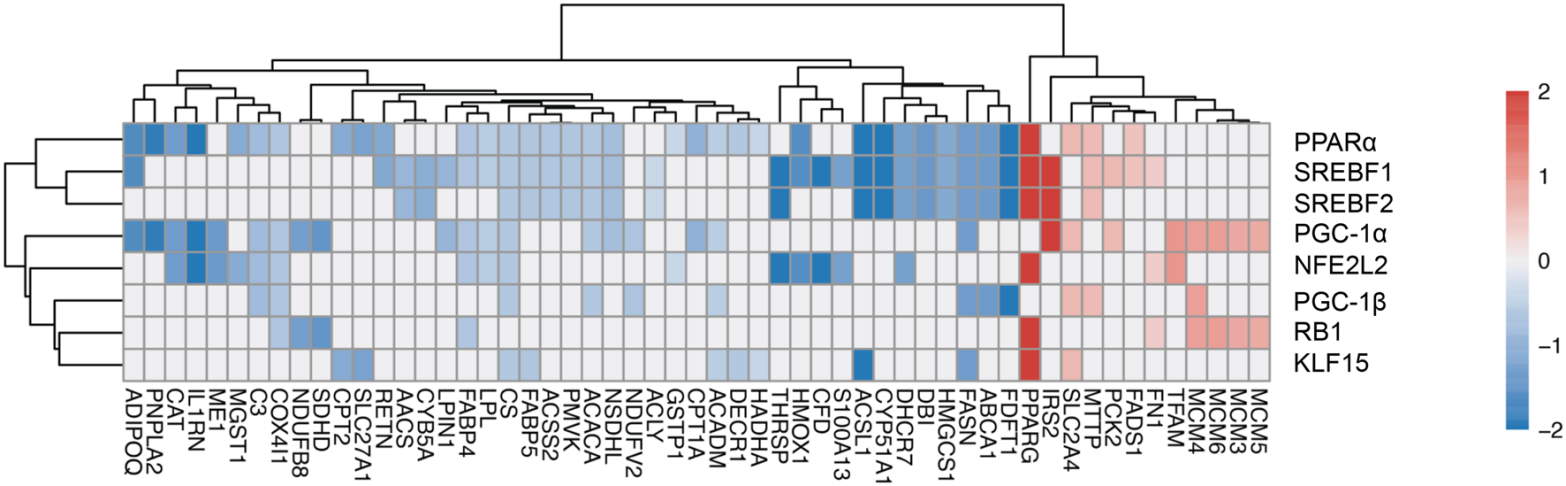
Identification of significantly-affected transcription factors and target mapping in SIRT1-depleted adipocytes. Heatmap clustering of the most significantly-affected molecules mapped to their upstream regulators in SIRT1-depleted adipocytes. Red and blue indicate up- and down-regulated molecules, respectively. Shown are upstream regulators with a downstream target overlap of P < 10^–5^ and an IPA activation z score > 2.7 or < − 2.7. The activation z score was calculated by IPA and it predicted if a pathway was activated or inhibited based on the directional change in proteins included in the quantified pathways.

To broaden our analysis and discover upstream transcription factors as well as link them to their targets in SIRT1-depleted adipocytes, the dataset was subjected to Ingenuity Pathway Analysis (IPA). IPA Upstream Regulator analysis was performed with differential proteins at P < 0.05 significance level. Transcriptional regulators and nuclear receptors (IPA activation z score > 2.7, < − 2.7; gene overlap P value < 1 × 10^–5^) were used to visualize the relationship between the predicted regulator (rows) and its measured downstream target log-ratio (columns). Shown in Fig. 5 is the heat map clustering of the most-significantly affected molecules mapped to their upstream regulators (Red and Blue indicate upregulated and downregulated, respectively). The IPA analysis revealed that the upstream regulators most-significantly affected by SIRT1-depletion were: (a) Peroxisome proliferator-activated receptor-α (PPARα); (b) Sterol regulatory element-binding transcription factor 1/2 (SREBF1/2); (c) peroxisome proliferator-activated receptor gamma coactivator 1-α/β (PGC-1α/β); (d) nuclear factor erythroid 2 Like 2 (NFE2L2); (e) retinoblastoma protein 1 (RB1); and (f) Kruppel-like factor 15 (KLF15). In summary, the data confirmed known regulatory interactions between SIRT1 and transcriptions factors like PGC-1α and uncovered new targets whose function was predicted to be SIRT1-dependent in adipocytes, including SREBF1/2 and PPARα.

**Figure 6:**
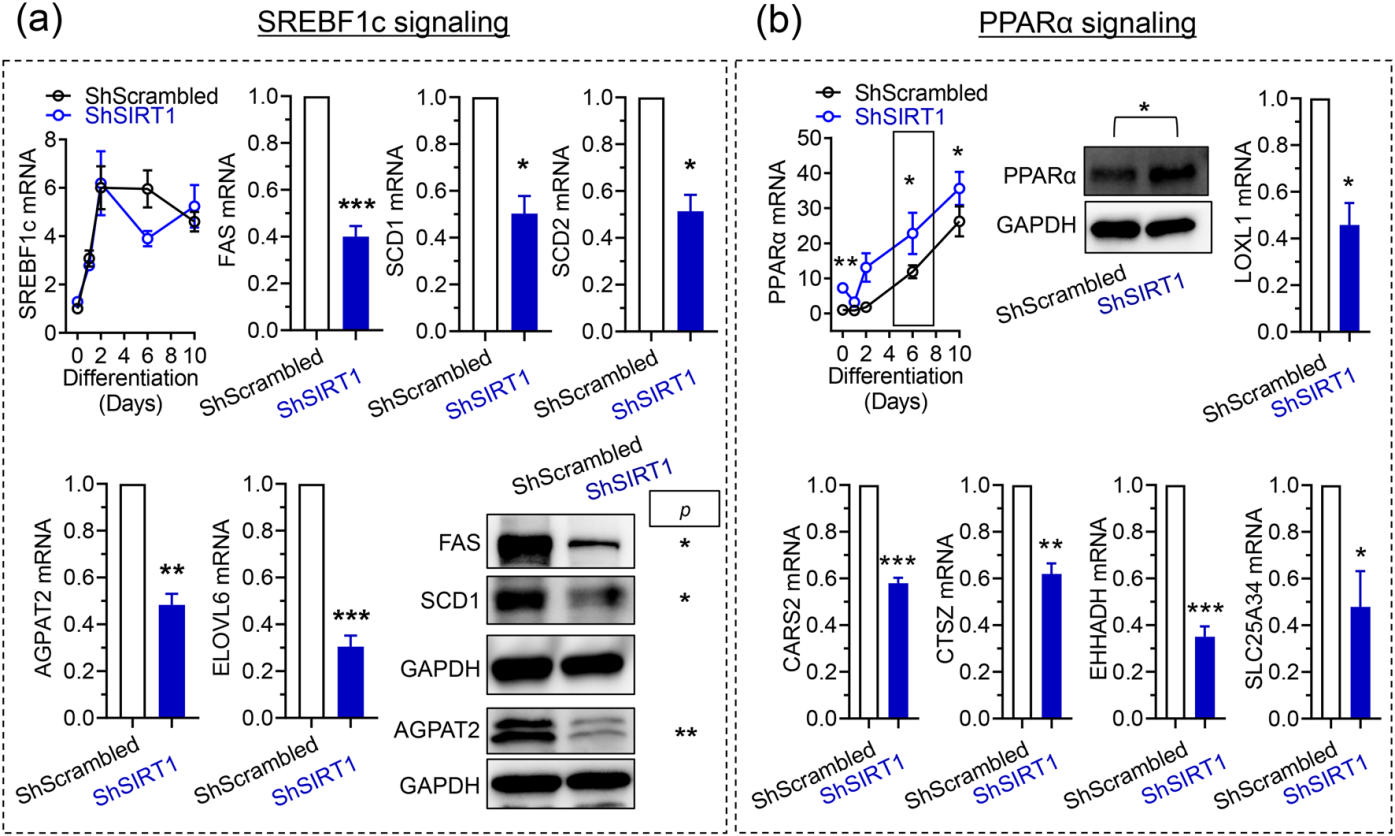
SIRT1-dependence of SREBF1c and PPARα signaling in 3T3-L1 adipocytes. (**a**) Quantitative analysis of the expression levels of SREBF1c and its downstream transcriptional targets in ShScrambled or SIRT1-depleted (ShSIRT1) adipocytes at Day 6 post-differentiation. (**b**) Quantitative analysis of the expression levels of PPARα and its downstream transcriptional targets in ShScrambled or SIRT1-depleted (ShSIRT1) adipocytes at Day 6 post-differentiation. Boxed region indicates the time-point at which PPARα protein levels were quantified. In (**a**,**b**), qPCR and western blot data were from 4 and 3 independent experiments, respectively. Statistical analysis of the dataset was performed using a Student’s t test (*P < 0.05; ****P < 0.0001; *n.s.* not significant).

To independently investigate the positive functional interaction of SIRT1 with SREBF1 and PPARα uncovered by the proteomics analysis, experiments were performed to compare changes in signaling by these transcription factors in ShScrambled and SIRT1-depleted (ShSIRT1) adipocytes. Because bioinformatics analysis predicted that fatty acid biosynthesis was suppressed in SIRT1-depleted adipocytes, experiments were designed to quantify changes in signaling via SREBF1c, which transcriptionally induces enzymes essential for fatty acid synthesis^37^. Analysis of SREBF1c transcript levels indicated that the increase in SREBF1c mRNA during differentiation was largely unaffected by SIRT1-depletion. However, expression levels of its downstream targets were significantly reduced, including fatty acid synthase (FAS), stearoyl-CoA desaturase-1 (SCD1), stearoyl-CoA desaturase-2 (SCD2), 1-Acylglycerol-3-Phosphate O-Acyltransferase-2 (AGPAT2), and elongation of very-long chain fatty acids 6 (ELOVL6) (Fig. 6a, Supplementary Figure S2a). These data also independently validated the proteomics study, which uncovered downregulation of FAS, SCD1, SCD2, and AGPAT2 in SIRT1-depleted adipocytes (Supplementary Table S2). Investigations of PPARα revealed that its mRNA and protein levels were significantly higher in SIRT1-depleted adipocytes, prompting us to investigate if its transcriptional function was altered (Fig. 6b, Supplementary Figure S2a). Genes induced by the specific PPARα agonist fenofibrate in brown adipocytes were recently reported^38^. Therefore, expression levels of these targets were compared in ShScrambled and ShSIRT1 adipocytes, revealing that SIRT1-depletion was associated with significantly reduced expression of molecules like lysyl oxidase like-1 (LOXL1), cysteinyl-tRNA synthetase-2 (CARS2), cathepsin Z (CTSZ), solute carrier family 25 member 34 (SLC25A34), and enoyl-CoA hydratase and 3-hydroxyacyl CoA dehydrogenase (EHHADH) (Fig. 6b). Importantly, reduced expression of LOXL1, CARS2, and CTASZ in SIRT1-depleted adipocytes was also uncovered by the proteomics analysis (Supplementary Table S2). Collectively, these data suggested SIRT1-dependence of SREBF1c and PPARα signaling in 3T3-L1 adipocytes.

CEBPα/β/δ and PPARγ transcription factors also play key roles in 3T3-L1 adipogenesis. Therefore, expression levels of these transcription factors were quantified in ShScrambled and SIRT1-depleted (ShSIRT1) adipocytes to gain insights into their SIRT1-dependence. In ShScrambled adipocytes, CEBPα expression was maximal at Day 6 post-differentiation and SIRT1-depleted adipocytes showed a small but significant reduction (Supplementary Figure S2b). The increase in CEBPβ expression in ShScrambled adipocytes was relatively transient—mRNA levels were maximal by Day1–2 post-differentiation and returned to basal levels by Day 10. However, SIRT1-depletion led to a sustained increase in CEBPβ levels and expression was significantly higher than ShScrambled throughout differentiation (Supplementary Figure S2b). In contrast to changes in CEBPα and CEBPβ expression triggered by SIRT1-depletion, changes in CEBPδ levels during differentiation were relatively unaffected in SIRT1-depleted adipocytes (Supplementary Figure S2b). PPARγ expression increased gradually over the course of differentiation and SIRT1-depletion caused a significant increase in its expression levels (Supplementary Figure S2b). Overall, these data suggested that SIRT1-depletion had little inhibitory effect on the expression levels of key adipogenic transcription factors during differentiation.

### SIRT1 promoted NMNAT2 expression during 3T3-L1 differentiation and supplementation with NMN elicited SIRT1-dependent and -independent effects on gene-expression

**Figure 7:**
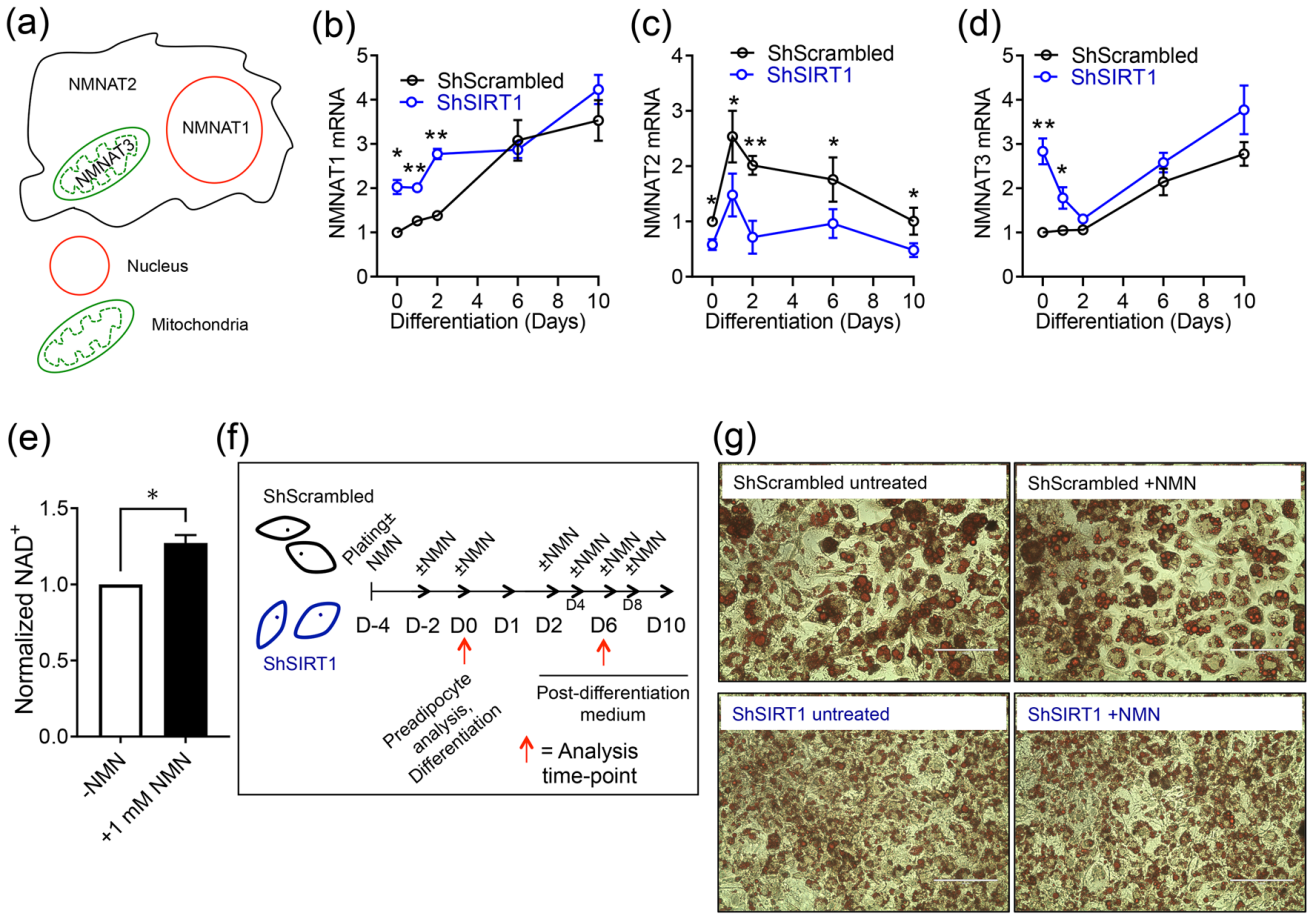
SIRT1 promoted NMNAT2 expression during 3T3-L1 differentiation and constitutively inhibited NMNAT1/3 expression. (**a**) Illustration of the subcellular localization of NMNAT enzymes. (**b–d**) qPCR data comparing the expression of NMNAT1 (**b**), NMNAT2 (**c**) and NMNAT3 (**d**) at specific time-points during adipogenesis in ShScrambled or SIRT1-depleted (ShSIRT1) (pre)adipocytes (n = 4). (**e**) Effect of supplementation with 1 mM NMN on total cellular NAD^+^ levels in 3T3-L1 preadipocytes (n = 4). (**f**) Experimental design to test the effect of NMN supplementation on gene-expression during 3T3-L1 adipogenesis. (**g**) Representative images of Oil Red O (ORO)-stained ShScrambled or SIRT1-depleted (ShSIRT1) adipocytes with (+NMN) or without (−NMN) supplementation with 1 mM NMN. Scale bar, 100 μm. Images were collected at Day 6 post-differentiation and are representative of 3 independent experiments. For statistical analysis of data shown in (**b–d**), pair-wise comparisons were made between ShScrambled and ShSIRT1 groups for each individual time-point using the Student’s t test (n = 4). A Student’s t test was used to perform statistical analysis on the dataset shown in (**e**) (n = 4) (*P < 0.05; **P < 0.01).

Emerging evidence has highlighted a role for NAD^+^ biosynthesis in 3T3-L1 adipogenesis. Specifically, evidence suggests that increased expression and activity of the cytosolic NMNAT–NMNAT2—depletes nuclear NAD^+^, which inhibits the NAD^+^-dependent enzyme PARP-1 to promote CEBPβ function and 3T3-L1 differentiation^36^. We hypothesized a role for SIRT1 in this signaling network and investigated if SIRT1 regulated the expression of NMNAT1 (nuclear), NMNAT2 (cytosolic) and NMNAT3 (mitochondrial) isoforms in 3T3-L1 (pre)adipocytes (Fig. 7a). In ShScrambled cells, NMNAT1 expression was robustly induced during late stages of adipogenesis. In contrast, SIRT1-depleted preadipocytes showed significantly higher NMNAT1 expression, which gradually increased during differentiation to levels that were comparable to ShScrambled adipocytes (Fig. 7b). NMNAT2 expression was biphasic in ShScrambled cells—expression was maximal by D1 and gradually returned to basal levels by Day 10. Importantly, however, SIRT1-depletion significantly suppressed the induction of NMNAT2 at all stages of differentiation (Fig. 7c). The expression pattern of NMNAT3 mRNA was similar to NMNAT1 in ShScrambled adipocytes and highest expression was observed by D6/D10. In contrast, NMNAT3 mRNA levels were significantly higher in SIRT1-depleted preadipocytes, expression gradually declined by D2, but increased again to levels that were comparable to ShScrambled adipocytes (Fig. 7d). The data suggested that SIRT1 promoted NMNAT2 expression during 3T3-L1 differentiation and constitutively repressed NMNAT1/3 expression in preadipocytes.

The observation that SIRT1-depletion downregulated NMNAT2 and upregulated NMNAT1/3 prompted us to investigate the functional impact of NAD^+^-signaling during 3T3-L1 differentiation. We supplemented ShScrambled and ShSIRT1 (pre)adipocytes with the NAD^+^ precursor NMN (1 mM) throughout the process of differentiation. Quantification of cellular NAD^+^ levels indicated that supplementation with 1 mM NMN significantly increased total intracellular NAD^+^ levels (Fig. 7e,f). Oil Red O staining of adipocytes at Day 6 post-differentiation suggested that supplementation with NMN did not elicit any overt effects of triglyceride synthesis (Fig. 7g). Next, we comprehensively profiled NMN-induced changes in gene-expression in ShScrambled and ShSIRT1 (pre)adipocytes during differentiation. This analysis was focused on a specific set of molecules (Supplementary Figure S3), including adipokines (leptin, adiponectin, LCN2), chemokines (CXCL1, CXCL10), extracellular matrix components (MMP3, MMP13, Col6A1, Col6A3), and transcription factors (including SREBF1c and PGC-1α) and their transcriptional targets (FAS, SCD1, AGPAT2, NDUFB4/6/8). Some molecules whose expression levels were altered in the proteomics dataset were also included (THRSP, TRPM7, GCHFR, TBC1D10A). Because NMNAT1-3 were dysregulated upon SIRT1-depletion, effects of NMN on their expression levels in ShScrambled and SIRT1-depleted (ShSIRT1) (pre)adipocytes were also investigated.

**Figure 8:**
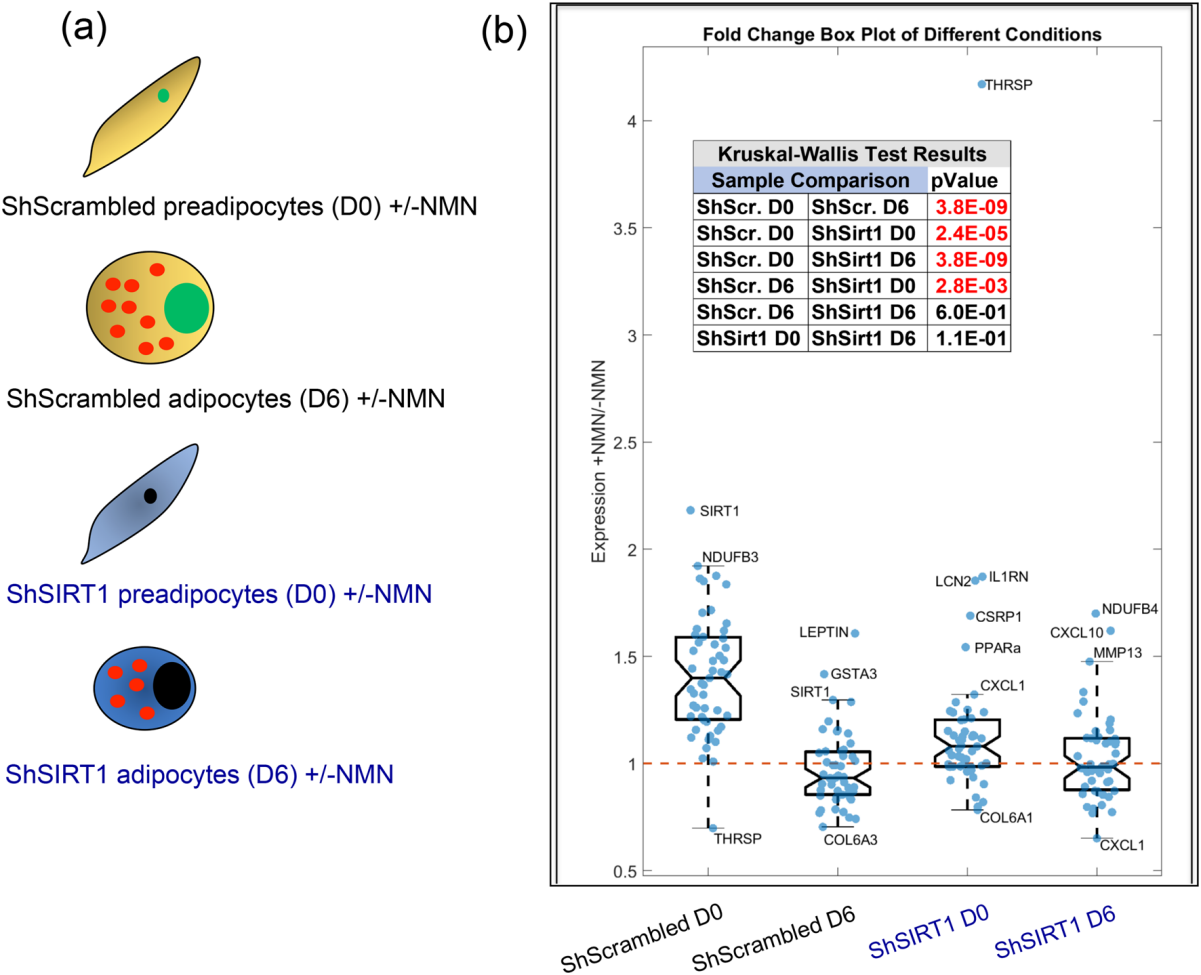
Supplementation with NMN elicited SIRT1-dependent and -independent changes in gene-expression during 3T3-L1 adipogenesis. (**a**) NMN-induced changes in expression of the indicated genes were analyzed in four experimental conditions: ShScrambled preadipocytes (ShScrambled D0), ShScrambled adipocytes (ShScrambled D6), ShSIRT1 preadipocytes (ShSirt D0) and ShSIRT1 adipocytes (ShSirt1 D6). (**b**) Shown are box plots indicating the effects of NMN on gene-expression in each of the 4 conditions, as indicated. Individual blue dots represent the ratio of +NMN/−NMN for each gene. The notched box shows the interquartile range (25th–75th percentile) and the central line within the box represents the median. The whiskers extend to points not considered outliers and data points outside the whiskers were considered quartiles. The dashed red line is set at 1 and the inset table shows the results of Kruskal–Wallis testing on each of these groups. Some of the molecules whose expression was significantly affected by NMN supplementation are also indicated.

NMN-induced changes in mRNA levels of the selected gene-set were quantified in ShScrambled and ShSIRT1 (pre)adipocytes. Transcriptional changes in response to supplementation with 1 mM NMN were tested in four different conditions: ShScrambled preadipocytes (ShScrambled D0), ShScrambled adipocytes (ShScrambled D6), ShSIRT1 preadipocytes (shSIRT1 D0), and ShSIRT1 adipocytes (ShSIRT1 D6) (Fig. 8a). Throughout this section, Day 0 (D0) represents preadipocytes and Day 6 (D6) represents mature adipocytes analysed 6 days post-differentiation (to ensure consistency with the proteomics analysis). To determine an NMN-induced change in transcript levels, data for each gene was analysed as a ratio of +NMN/−NMN. Shown in Fig. 8b are boxplots for all four experimental conditions, where individual dots represent the +NMN/−NMN ratios for all the genes that were analyzed. This analysis indicated that supplementation with 1 mM NMN increased the expression levels of several genes in ShScrambled preadipocytes (ShScrambled D0), including SIRT1, PGC-1α, mitochondrial NADH Dehydrogenase complex subunits (NDUFA3, NDUFB3, NDUFB5, NDUFB6, and NDUFB8), and NMNAT1-3. Statistical analysis comparing the ‘NMN-responsiveness’ of ShScrambled preadipocytes *vs* ShScrambled adipocytes indicated that the cellular response to NMN was significantly weaker in adipocytes (Fig. 8b inset). A key molecule whose expression levels were increased by NMN supplementation in ShScrambled adipocytes was the ‘appetite-suppressant’ hormone leptin and, importantly, this increase was significantly smaller in SIRT1-depleted adipocytes (ShScrambled D6 *vs* ShSIRT1 D6). Other genes whose expression levels were altered by supplementation with NMN in adipocytes in a SIRT1-dependent manner included SIRT1 and GSTA3 (increased expression), and Col6A3 (decreased expression) (Fig. 8b).

Statistical analysis of the dataset indicated that SIRT1-depletion significantly attenuated the effects of NMN in (pre)adipocytes. However, expression of a small number of genes was increased by supplementation with NMN in SIRT1-depleted preadipocytes, including IL1RN, LCN2, CRSP1, PPARα and CXCL1 (Fig. 8b). In addition, genes that were induced in SIRT1-depleted adipocytes were NDUFB4, CXCL10 and MMP13. The levels of Col6A1 and CXCL1 were reduced by NMN supplementation in both SIRT1-depleted preadipocytes and adipocytes (Fig. 8b). These apparently SIRT1-independent effects of NMN may reflect residual SIRT1 function or engagement of other NAD^+^-dependent pathways.

To highlight the effects of NMN supplementation on individual genes, to profile these effects in preadipocytes *vs* adipocytes, and to integrate their SIRT1-dependence, scatterplots were generated to compare (**a**) ShScrambled preadipocytes (D0) *vs* ShScrambled adipocytes (D6) and (**b**) ShSIRT1 preadipocytes (D0) *vs* ShSIRT1 adipocytes (D6) (Supplementary Figure S3a). For clarity, the data are divided into 4 quadrants: Quadrant 1 represents genes whose expression levels decreased with NMN supplementation in preadipocytes and adipocytes; Quadrant 2 represents genes whose expression decreased with NMN supplementation in preadipocytes but increased in adipocytes; Quadrant 3 represents genes whose expression increased with NMN supplementation in preadipocytes but decreased in adipocytes; and Quadrant 4 represents genes whose expression increased with NMN supplementation in both preadipocytes and adipocytes. The scatterplots suggested that a large number of genes occupied quadrant 3 in ShScrambled (pre)adipocytes, indicating their sensitivity to NMN. However, these NMN-induced changes were smaller in SIRT1-depleted (pre)adipocytes (compare quadrant 3 in panel a *vs* panel b). This gene-set included PGC-1α, NDUFB3, NDUFB5, NDUFB6, and NDUFB8, and NMNAT1-3. Furthermore, the increase in expression levels of leptin, SIRT1, and GSTA3 was significantly smaller in SIRT1-depleted adipocytes (compare quadrant 4 in panel a *vs* panel b). Shown in Supplementary Figure S3b is an integration of NMN-induced changes in gene-expression across both ShScrambled and ShSIRT1 groups. Gene-expression profiles are divided into groups based on the quadrant identities highlighted in Supplementary Figure S3a. In the Group column, the first and second number indicate the gene’s quadrant identity in ShScrambled and ShSIRT1, respectively. The +NMN/−NMN ratio shown in green and red bars represents ratios of > 1 and < 1, respectively. The height of each bar is proportional to the effect observed with NMN supplementation. For example, leptin, SIRT1, and GSTA3 were all assigned to group 44, indicating that they were sensitive to NMN in both ShScrambled and ShSIRT1 conditions. However, the amplitude of the NMN-effect was significantly reduced for each of these molecules after SIRT1-depletion (Supplementary figure S3b). Collectively, these experiments revealed that preadipocytes exhibited significantly higher sensitivity to NMN supplementation when compared to adipocytes and that SIRT1-depletion significantly reduced NMN responsiveness. These experiments also highlighted leptin, SIRT1, and PGC-1α and its transcriptional targets as metabolic regulators whose expression levels were sensitive to NAD^+^-boosting in a SIRT1-dependent manner.

## Discussion

We previously demonstrated that SIRT1 restricted adipocyte hyperplasia by inhibiting C-Myc. Using stable isotope-labeling with amino acids (SILAC) coupled with proteomics analysis in 3T3-L1 preadipocytes, we established that C-Myc signaling was activated in SIRT1-silenced preadipocytes^25^. Importantly, we also discovered that SIRT1-depletion was associated with adipocyte dysfunction, evidenced by increased expression of white adipose tissue (WAT) markers (PANK3 and AGT), decreased expression of brown adipose tissue (BAT) markers (PRDM16), and increased inflammation (TNF-α)^25^. These observations prompted us to investigate how SIRT1 function regulated molecular pathways that were integral to 3T3-L1 differentiation. We employed quantitative proteomics using dimethyl-labelling of protein lysates extracted from ShSIRT1 and ShScrambled adipocytes, followed by isoelectric-focusing and mass-spectrometry analyses. Pathway analysis of the dataset revealed SIRT1-dependence of key transcription factors, including PPARα, SREBF1/2, and PGC-1α. SIRT1-dependence of SREBF1c and PPARα signaling was functionally validated by quantifying expression levels of downstream transcriptional targets. Studies of adipocyte mitochondrial function revealed that SIRT1 promoted mitochondrial biogenesis and respiration in adipocytes and the expression of key molecules like leptin, adiponectin, matrix metalloproteinases 3/13 (MMP3/13), lipocalin 2 (LCN2), and thyroid hormone responsive (THRSP) was SIRT1-dependent. In addition, expression-profiling of NMN acetyl transferases (NMNAT) revealed that SIRT1 promoted NMNAT2 expression during adipogenesis and constitutively repressed NMNAT1/3 levels. Supplementation with NMN during differentiation induced the expression of Sirt1, PGC-1α and its transcriptional targets, leptin, and reduced the expression of pro-fibrotic collagens, Col6A1 and Col6A3.

To identify significantly-affected transcription factors and to link them to downstream targets in SIRT1-depleted adipocytes, the proteomics dataset was subjected to Ingenuity Pathway Analysis (IPA). This analysis uncovered key transcription factors whose activity was predicted to be inhibited in SIRT1-depleted adipocytes, including PPARα, PGC-1α, SREBF1, and NFE2L2. PPARα is a master regulator of lipid metabolism and fatty acid-oxidation^39^, and its metabolic significance is exemplified by the clinical use of PPARα agonists as ‘lipid-lowering’ agents^40^. Importantly, activation of PPARα in 3T3-L1 adipocytes using a specific agonist (GW7647) promotes insulin-stimulated glucose uptake and expression of aP2 and PPARγ, without affecting triglyceride accumulation. Genes whose expression is induced by PPARα-activation include those involved in fatty acid-oxidation, such as acyl-coA oxidase (ACO), carnitine palmitoyl transferase 1b (CPT1b) and uncoupling protein-3 (UCP-3)^41^. There is little known about the metabolic consequences of SIRT1-PPARα signaling in adipocytes. However, their cooperation has been discovered in other systems. For example, PPARα-activation downstream of SIRT1 is protective in cardiac hypertrophy and benefits associated with SIRT1-activation are lost when PPARα signaling is inhibited^42^. Post-translational ‘PARylation’ of PPARα by PARP-1 disrupts SIRT1-PPARα interaction and reduces fatty acid-oxidation in the liver^43^, and SIRT1 activation by adipose triglyceride lipase (ATGL) supports PPARα transcriptional activity in hepatocytes^44^. Upregulation of PPARα mRNA and protein in SIRT1-depleted adipocytes (Fig. 6b) probably reflects a feedback mechanism to compensate for its functional inhibition. It will be interesting to investigate if PPARα is a deacetylation target of SIRT1, identify putative target lysine residues, and determine how SIRT1-dependent deacetylation affects its function as a transcription factor in adipocytes.

Another upstream regulator predicted by the IPA to be inhibited in SIRT1-depleted adipocytes was PGC-1α, which is a central regulator of oxidative phosphorylation, fatty acid-oxidation, and mitochondrial biogenesis^16^. We discovered that SIRT1 promoted PGC-1α expression levels during 3T3-L1 differentiation and this was consistent with reduced mitochondrial mass and respiratory capacity in SIRT1-depleted adipocytes. SIRT1 physically interacts with and deacetylates PGC-1α at 13 lysine residues, including Lys^183^, Lys^253^, Lys^320^, Lys^346^, and Lys^441^. This triggers an increase in PGC-1α transcriptional activity and this effect is recapitulated by putative SIRT1 activators like resveratrol^45,46^. PGC-1α overexpression in 3T3-L1 preadipocytes also promotes mitochondrial biogenesis independently of cell proliferation^47^. However, increased mitochondrial gene expression in 3T3-L1 adipocytes in response to rosiglitazone was reported to occur independently of PGC-1α, instead requiring PGC-1β function^48^. Our proteomics analysis also suggested that SIRT1-depletion was associated with inhibition of PGC-1β signaling. This raises the possibility that functional interaction between SIRT1 and PGC-1β may regulate mitochondrial function in adipocytes, particularly because PGC-1β is a known SIRT1 target^49,50^. Finally, our analysis suggested that expression levels of the transcription factor CEBPα were significantly lower in SIRT1-depleted adipocytes (Supplementary figure S2b). CEBPα plays a central role in adipogenesis and energy metabolism, and its overexpression is sufficient to induce terminal differentiation of 3T3-L1 preadipocytes^12,51,52^. Importantly, the adipokine leptin is a direct transcriptional target of CEBPα^53,54^ and reduced levels of leptin in SIRT1-depleted adipocytes suggest that both CEBPα expression and function may be compromised when SIRT1 levels are downregulated. Improved leptin sensitivity was also proposed as a downstream mechanism underlying the beneficial metabolic effects observed in mice overexpressing SIRT1 in the hypothalamus^55^. Suppression of CEBPα signaling may also underlie reduced expression levels of the adipokines adiponectin and lipocalin 2 (LCN2) in SIRT1-depleted adipocytes because experimental evidence not only suggests transcriptional regulation of these adipokines by the CEBP family of transcription factors (including CEBPα) but also the presence of specific CEBP binding-sites in their promoter regions^56,57^.

An unexpected observation in the proteomics study was the predicted positive effect of SIRT1 on SREBF1/2 transcriptional activity in adipocytes. In the liver, SIRT1 physically interacts with and negatively regulates SREBF function via deacetylation at lysine residues 289 (Lys^289^) and 309 (Lys^309^), and this constitutes an essential and conserved biological response that links hepatic de novo lipogenesis (DNL) to fasting and re-feeding^58,59^. However, in contrast, the data reported in this study suggested suppression of SREBF1/2 signaling in SIRT1-depleted adipocytes (Fig. 5). This inhibitory effect of SIRT1-depletion was supported by validation experiments that indicated reduced levels of transcriptional targets of SREBF1c, including FAS, AGPAT2, ELOVL6, SCD1, and SCD2 (Fig. 6 and Supplementary figure S2a). These observations are intriguing because SREBF1 overexpression promotes fatty acid metabolism in 3T3-L1 adipocytes and its overexpression generates lipids that are PPARγ agonists^60,61^. Therefore, the data suggest functional relevance of SIRT1-SREBF signaling to adipocyte DNL and encourage further investigations, especially because SREBF-dependent DNL in adipose tissue promotes metabolic benefits in mice^62^ and lipids generated by adipocyte DNL elicit insulin-sensitizing effects in vivo^63–65^.

Proteomics and bioinformatics analyses of the dataset not only uncovered key SIRT1-regulated metabolic pathways but also identified proteins whose expression levels were altered by SIRT1-depletion. For example, our data indicated that the enzyme transglutaminase (TGM2) was induced in SIRT1-depleted adipocytes. TGM2 is a negative regulator of adipogenesis and TGM2-null fibroblasts show accelerated adipogenesis and increased expression levels of CEBPα, PPARγ, and GLUT4^66^. Importantly, TGM2 also supports the trans-differentiation of glioma stem cells by triggering degradation of CEBP Homologous Protein (CHOP/GADD153), leading to a reciprocal increase in CEBPβ levels^67^. These data therefore suggest that TGM2 hyperactivity may explain the increased levels of CEBPβ in SIRT1-depleted adipocytes (Supplementary figure S2b and ^25^) and compromised SIRT1-TGM2-CEBPβ signaling could underlie dysfunctional adipogenesis in these cells. SIRT1-depleted adipocytes also showed increased levels of ECM components such as laminin α4 (LAMA4), laminin C1 (LAMC1) and collagen 6A3 (Col6A3), and decreased levels of matrix metalloproteinases (MMP) such as MMP3 and MMP13. Interestingly, elevated levels of LAMA4 and LAMC1 are observed in cancer cells^68,69^, which suggests that their upregulation in SIRT1-depleted adipocytes reflects their hyperplastic phenotype. Importantly—in a metabolic context—LAMA4 knock-out mice resist diet-induced obesity (DIO), show reduced visceral fat expansion, and increased energy expenditure^70,71^, suggesting that LAMA4 promotes metabolic dysfunction in obesity. MMP-dependent proteolytic processing of ECM components like collagens allows adipose tissue remodeling and expansion in obesity^72^ and inhibition of MMP function in vivo promotes metabolic dysfunction^73–76^. Our in vitro data therefore encourage investigations of how SIRT1 regulates adipose tissue MMPs and pro-fibrotic collagen levels in vivo in obesity, especially because metabolic dysfunction in SIRT1 knock-out mice is associated with compromised expansion of visceral adipose tissue^77^.

To address the emerging role of NAD^+^ as a regulator of 3T3-L1 adipogenesis, we performed a comprehensive analysis of the effects of NMN supplementation on gene-expression in (pre)adipocytes and investigated the contribution of SIRT1 in transducing these effects. The data indicated that preadipocytes were more responsive to NMN supplementation and many of these effects of NMN were SIRT1-dependent. For example, supplementation with NMN significantly increased the expression of SIRT1, as reported previously^78^. Expression levels of PGC-1α and subunits of the mitochondrial NADH dehydrogenase complex were also increased by supplementation with NMN. These subunits are encoded by the nuclear genome and available evidence suggests their transcriptional regulation by PGC-1α^79–81^. Therefore, it is conceivable that a SIRT1-mediated increase in PGC-1α transcriptional activity underlies the induction of these genes in response to NMN in 3T3-L1 preadipocytes.

Our analysis also indicated that supplementation with NMN significantly increased the expression of the adipokine leptin in 3T3-L1 adipocytes. Importantly, this increase was significantly blunted when SIRT1 levels were depleted, thereby suggesting that enhanced NAD^+^-dependent SIRT1 function is responsible for this induction (Fig. 8 and Supplementary Figures S3a,b). We also discovered significantly reduced expression of leptin in SIRT1-depleted adipocytes (Fig. 1d,i). Leptin was discovered nearly 25 years ago and its function as an ‘appetite-suppressant’ hormone and regulator of metabolism and energy expenditure is well-established^82,83^. Leptin-null mice are obese, hyperphagic, glucose-intolerant, and diabetic^84^. Obesity is also associated with leptin-resistance and MMP-dependent cleavage of leptin is a mechanism underlying leptin-resistance in obesity^85^. To our knowledge, stimulation of leptin transcription by ‘NAD^+^-boosting’ and the dependence of this effect on SIRT1 function has not been previously reported. However, a SIRT1-dependent improvement in leptin-sensitivity has been reported, which is mediated by the downregulation of proteins that promote leptin-resistance^55,86,87^. Our data therefore encourage further investigations to evaluate the metabolic relevance of increased leptin transcription by NAD^+^-boosters like NMN and the relevance of SIRT1 in transducing this effect. As discussed above, it is conceivable that inhibition of CEBPα-signaling in SIRT1-depleted adipocytes may account for some of these effects. Our data also suggested that NMN supplementation reduced the expression of Col6A1 and Col6A3. These subunits of Collagen VI have established pro-fibrotic and pro-inflammatory roles in metabolism^88,89^, so their reduction by NMN supplementation is predicted to be metabolically beneficial. The observation that SIRT1 was required to induce NMNAT2 expression during differentiation raises the possibility that SIRT1 directly coordinates the adipogenic program by suppressing nuclear NAD^+^ synthesis, leading to inhibition of PARP-1 and de-repression of CEBPβ activity^35,36^. Finally, expression levels of a small number of genes were altered by supplementation with NMN in SIRT1-depleted (pre)adipocytes (Fig. 8). These effects may reflect residual SIRT1 function and/or involvement of other NAD^+^-dependent proteins, including other Sirtuins.

**Figure 9:**
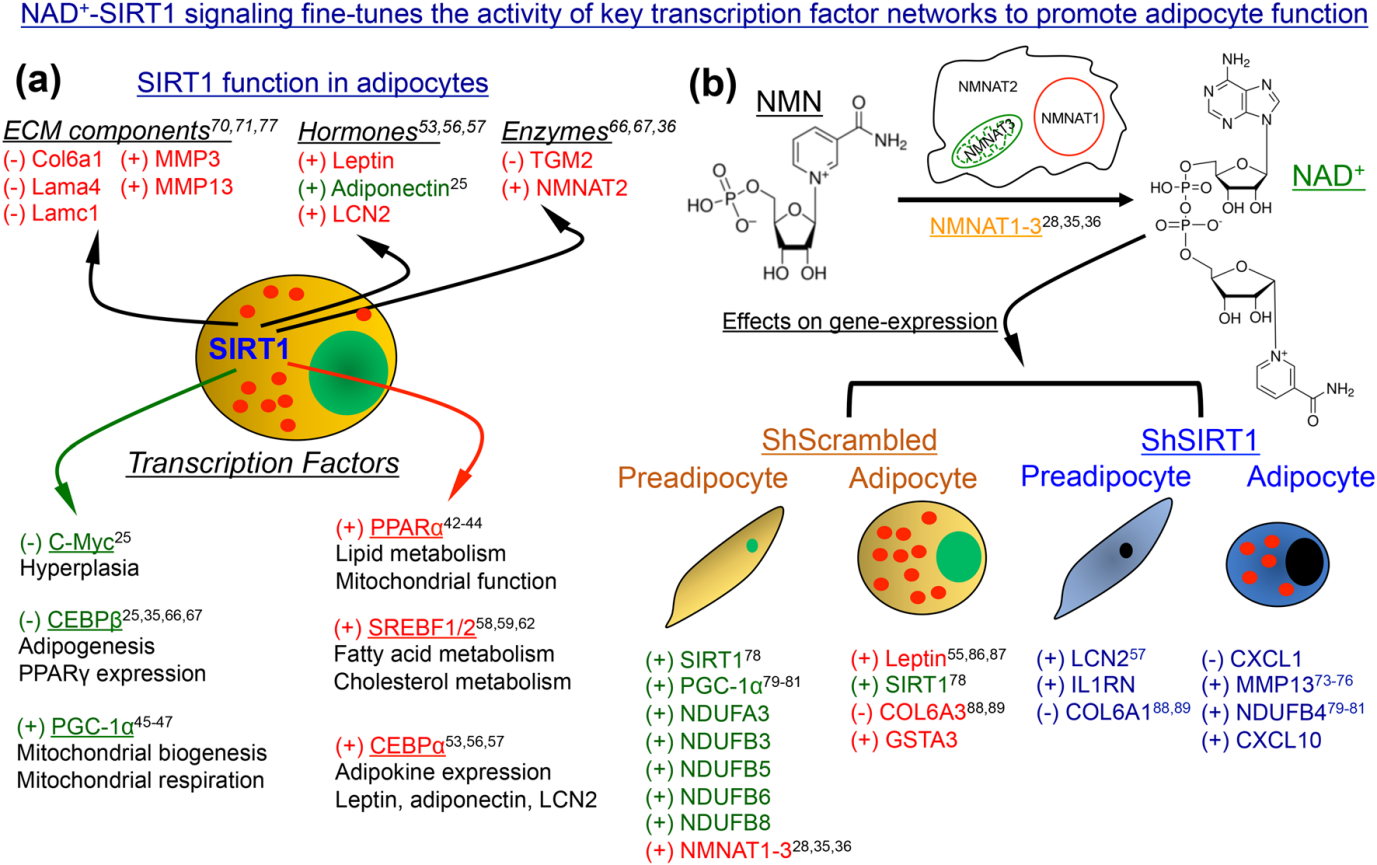
NAD^+^-SIRT1 signaling fine-tunes the activity of key transcription factor networks to promote adipocyte function. (**a**) Schematic summary of the data obtained by combining quantitative proteomics and gene-expression analysis in adipocytes. Positive or negative effect of SIRT1 on the expression levels of individual molecules (top) or transcription factor pathways (bottom) is indicated by ‘+’ or ‘−’ sign, respectively. Molecules highlighted in red represent those whose interaction with SIRT1 to our knowledge has not been previously reported in the context of adipogenesis, adipose tissue function, or obesity. Evidence of an interaction between SIRT1 and molecules highlighted in green can be found in the literature. (**b**) Schematic summary of the effects of supplementation with NMN on gene-expression in ShScrambled and ShSIRT1 (pre)adipocytes. Positive or negative effect of NMN supplementation on the expression of indicated genes is indicated by ‘+’ or ‘−’ sign, respectively. Genes highlighted in red represent those whose SIRT1-dependence in response to NMN has not been previously reported, while those in green represent molecules whose association with NAD^+^-SIRT1 signaling is known. Molecules highlighted in blue represent those whose expression was altered by NMN in SIRT1-depleted (pre)adipocytes, which may reflect residual SIRT1 function or involvement of other NAD^+^-dependent proteins. Chemical structures of NMN and NAD^+^ are shown, and conversion of NMN to NAD^+^ occurs intracellularly by the action of NMNAT enzymes. The numbers indicate citations that link each molecule or pathway to the relevant section in “Discussion”.

In summary, we used a combination of quantitative proteomics, gene-expression and biochemical studies, and mitochondrial analysis to identify key Sirt1-regulated metabolic pathways in 3T3-L1 adipocytes. These data confirm previously reported interactions of SIRT1 with transcription factors like PGC-1α and provide new evidence suggesting that SIRT1 supports PPARα and SREBF1c function in adipocytes, which may be relevant to lipid metabolism (Fig. 9). In addition, we identified SIRT1 as a positive regulator of NMNAT2 and discovered key Sirt1-dependent changes in gene-expression elicited by supplementation with the NAD^+^ precursor NMN, including a significant increase in the expression of a key adipokine and metabolic regulator, leptin (Fig. 9). These data provide new insights into how SIRT1-NAD^+^ signaling fine-tunes adipogenic and metabolic pathways in adipocytes.

## Materials and methods

### 3T3-L1 preadipocyte culture and SIRT1 gene-silencing

3T3-L1 preadipocytes (#SP-L1-F, Zen-Bio) were cultured in DMEM (#12430-054, ThermoFisher Scientific) supplemented with 10% bovine serum (#16170-078, ThermoFisher Scientific) and 1% antibiotic–antimycotic (#15240-062, ThermoFisher Scientific). To deplete endogenous SIRT1 and establish a stable cell-line, a lentiviral RNA gene-silencing system was used as described previously^25^. To generate lentiviral particles, HEK293T cells (ATCC) grown in DMEM supplemented with 10% fetal bovine serum (FBS) (#Alpha FBS, Alphabioregen) and 1% antibiotic–antimycotic (complete medium) were co-transfected (using Lipofectamine 2000) with plasmids encoding ShSIRT1 (or Scrambled ShRNA) and the lentiviral packaging plasmids (pVSVg and psPAX2). 6 h later, the transfection medium was replaced with complete medium and lentiviral supernatants were collected 72 h post-transfection, centrifuged to clear cell debris and filter-sterilized prior to storage at -80 °C. 3T3-L1 preadipocytes (passage 11–12) were transduced with lentiviral particles in the presence of polybrene (4 µg/ml) for 48 h, sub-cultured 2–3 times in the presence of puromycin (2 µg/ml) prior to validation of SIRT1-depletion by western blotting. Hairpin sequences of SIRT1-specific (ShSIRT1) or Scrambled ShRNA (shScrambled) were reported previously^25^.

### 3T3-L1 differentiation

For differentiation experiments, cells were plated in 6-well plates or 10 cm petri dishes at a density of 200 × 10^3^/well or 1.2 × 10^6^, respectively. Medium was replaced 2 days post-plating, followed by incubation with the differentiation cocktail 48 h later. Cells were incubated with the differentiation cocktail for 2 days then switched to post-differentiation medium, which was replenished every 2 days for the duration of the experiment. The differentiation cocktail was prepared in DMEM (#12430-054, ThermoFisher Scientific) supplemented with 10% fetal bovine serum (#Alpha FBS, AlphaBioregen), 10 μg/ml insulin (#I9278, Sigma-Aldrich), 0.5 μM 3-isobutyl-1-methylxanthine (IBMX) (#PHZ1124) and 1 μM dexamethasone (#D1756, Sigma-Aldrich). Post-differentiation medium contained 10% FBS and 10 μg/ml insulin. For experiments with NMN (#N3501, Sigma-Aldrich), cells were left untreated or treated with 1 mM NMN every 2 days throughout the course of the differentiation protocol described above. Cells were collected for analysis at specific time-points during the experiments.

### Oil Red O staining

Standard protocols were used for Oil Red O (ORO) staining of adipocytes at Day 6 post-differentiation. Briefly, cells were fixed using 4% paraformaldehyde (PFA) for 15 min, washed once with 60% isopropanol (1 min), and stained with ORO for 1 h. Cells were then washed using 60% isopropanol (1×), distilled water (3×), followed by counter-staining with hematoxylin for 10 min. Following washes using distilled water (5×), cells were mounted using 70% glycerol prior to microscopy.

### RNA extraction, cDNA synthesis and qPCR

Cells were lysed in Qiazol (#79306, Qiagen) and total RNA was isolated using the miRNeasy kit (#217004, Qiagen) according to the manufacturer’s instructions. A DNA digestion step was incorporated into the protocol and sample incubation with DNase I (#79254, Qiagen) was performed for 15 min at room temperature prior to RNA elution. RNA was quantified using NanoDrop (ThermoFisher Scientific) and 500–1000 ng RNA was reverse-transcribed to cDNA using the High-Capacity RNA-to-cDNA kit (#4387406, ThermoFisher Scientific). cDNA was diluted 1:10 prior to use in quantitative PCR (qPCR) reactions. QuantStudio6 Flex Real-Time PCR System (ThermoFisher Scientific) was used for qPCR and the thermal cycling parameters were as follows: Stage I: denaturation (50 °C, 20 s; 95 °C, 10 min); Stage II (40 cycles): 95 °C, 15 s; 60 °C, 1 min; Stage III (Melt curve analysis): 95 °C, 15 s; 60 °C, 1 min; 95 °C, 30; seconds; 60 °C, 15 s. Sequences of all the primers used in the study are listed in Supplementary Table S1.

### Western blotting

Western blotting was performed as described previously^25,90^. Briefly, cells were lysed in RIPA buffer (#89900, ThermoFisher Scientific) containing 1× protease inhibitor cocktail and protease, phosphatase and deacetylase inhibitors. The lysates were sonicated and centrifuged at 15,000*g* for 10 min at 4 °C to pellet any cell debris. Protein concentration of the supernatants was quantified using the BioRad DC Protein assay kit on a CLARIOstar microplate reader (BMG Labtech). 50 μg protein was loaded on an SDS-PAGE gel for electrophoresis and then transferred to a PVDF membrane. To reduce non-specific binding, a solution containing 4% bovine serum albumin (BSA) and 0.1% Tween-20 dissolved in TRIS-buffered saline (TBS) was used. Membranes were incubated with the primary antibody at 4 °C overnight on a rocker and washed 3 times prior to incubation with the appropriate HRP-conjugated secondary antibodies at room temperature for 1 h. Membranes were then washed 3 times prior to detection of proteins using the SuperSignal West Chemiluminescent Substrate (#34095, ThermoFisher Scientific) on a ChemiDoc MP imaging system (Bio-rad). The suppliers and catalogue numbers of the antibodies used in this study are as follows: Cell Signaling Technology—SIRT1 (1F3) (#8469), β-actin (#3700), Adiponectin (#2789), SCD-1 (#2438), CEBPα (#8178), PPARγ (#2443), GAPDH (#2118), anti-mouse IgG HRP-conjugated antibody (#7076), and anti-rabbit IgG HRP-conjugated antibody (#7074); EMD Millipore—Leptin (#AB1673); Abcam—MMP3 (#ab53015), MMP13 (#ab39012), CEBPβ (#ab32358), CEBPδ (#ab65081), Col6A3 (#ab231025), and LCN2 (#ab216462); Invitrogen—AGPAT2 (#PA5-76010), THRSP (#PA5-77177), and PPARα (#MA1-822); Santa Cruz Biotechnology—FAS (#sc48357). For data quantification, Fiji—ImageJ software (www.imagej.net/Fiji) was used. Signal intensity of the protein of interest was divided by the corresponding signal for GAPDH (or β-actin), followed by normalization to ShScrambled within each experiment. All the original, uncropped western blot data can be found in Supplementary Figures S4–S7.

### Adipocyte mitochondrial mass analysis

For mitochondrial mass analysis in adipocytes, ShScrambled and ShSIRT1 preadipocytes were plated in 1% w/v gelatin-coated glass-bottom 6-well plates (MatTek, #P06G-1.5-10-F) at a density of 100 × 10^3^ cells/well and differentiated using the protocol described above. At Day 6 post-differentiation, adipocytes were incubated for 40 min in a humidified CO_2_ incubator with 250 nM MitoTracker Green FM (#7514, ThermoFisher Scientific) and 1 µg/ml Hoechst 33342 (#H3570, ThermoFisher Scientific) prepared in Hank’s Buffered Saline Solution supplemented with 1.5 mM each of CaCl_2_ and MgCl_2_ (HBSS/Ca^2+^/Mg^2+^). Adipocytes were washed 3 × with HBSS/Ca^2+^/Mg^2+^ prior to imaging. Images were captured on a Carl Zeiss LSM-880 microscope using a 63 × objective and a series of z-sections through the sample were collected for each field using the Tile Scan function. Images were analyzed using Fiji-ImageJ software (www.imagej.net/Fiji). For analysis and presentation, 2-D images were generated by compacting the z-sections using the Z-project tool followed by quantification of MitoTracker Green intensity, which was normalized to cell number. Data were averaged from 3 independent experiments and measurements were made from 1632 (ShScrambled) and 2323 (ShSIRT1) adipocytes for quantification.

### Adipocyte mitochondrial respiration analysis

For mitochondrial respiration assays in adipocytes, ShScrambled and ShSIRT1 preadipocytes (5 × 10^3^ cells/well) were seeded for differentiation in 1% w/v gelatin-coated XFe96 microplates in 100 µl culture medium. Differentiation was performed as described above. At Day 6 post-differentiation, adipocytes were gently washed twice with 200 µl XF assay medium (pH 7.4) supplemented with 10 mM glucose, 2 mM glutamine, and 1 mM sodium pyruvate. A final volume of 180 µl assay medium was added to each well and adipocytes were incubated at 37 °C in a non-CO_2_ incubator for 1 h prior to analysis. Oxygen Consumption Rate (OCR) was measured using a Seahorse Biosciences XFe96 Analyzer under basal conditions, followed by sequential injection of oligomycin (1 μM), FCCP (1 μM), and antimycin A/rotenone (0.5 μM) at specified time-points. For quantification, data were normalized to cell number determined by Hoechst 33342-labelling and imaging of adipocytes post-measurement using the ImageXpress cellular imaging system (Molecular Devices). XF Cell Mito Stress test report generator was used to calculate the Mito Stress test parameters from Wave data. Data are presented as the mean ± SEM from 12 biological replicates.

### Cellular NAD^+^ quantification assay

Changes in total cellular NAD^+^ levels after treatment with NMN were quantified using the NAD/NADH-Glo assay (#G9071, Promega) according to the manufacturer’s instructions. Standard curve for NAD^+^ was generated using defined concentrations of NAD^+^ prepared in PBS (#N8285, Sigma-Aldrich). Lysates were mixed with 0.4 N HCl, incubated at 60 °C for 15 min, cooled for 10 min at room temperature, and incubated with Trizma base prior to quantification. The Detection Reagent contained: Luciferin Detection Reagent, Reductase substrate, Reductase, Cycling Enzyme and NAD^+^ Cycling substrate. Samples and standards were mixed 1:1 with the Detection reagent prior to measuring luminescence on a luminometer. Sample luminescence values were normalized to protein concentration measured using the BioRad DC Protein assay kit on a CLARIOstar microplate reader (BMG Labtech).

### Quantitative proteomics and data analysis

To quantify proteome changes in SIRT1-depleted adipocytes, dimethyl-labeled protein extracts of ShSIRT1 and ShScrambled adipocytes were prepared, separated by isoelectric-focusing and subjected to mass-spectrometry and downstream bioinformatics analyses. Three biological replicates of differentiated ShSIRT1, ShScrambled and non-infected control adipocytes were used. Lysates from the three conditions were subjected to methanol/chloroform precipitation to remove detergents and enzymes. The pellets were then resuspended in 6 M/2 M Urea/Thiourea in 30 mM HEPES buffer (pH 8) prior to quantification of protein concentration using the Bradford assay. A 50 μg aliquot of each sample was reduced with 1 mM dithiothreitol (DTT) at room temperature (30 min) prior to alkylation with iodoacetamide (5.5 mM, 30 min). Trypsin was used to digest the samples overnight at 37 °C using a protein:enzyme ratio of 50:1. An internal standard was prepared by pooling all non-infected control samples, containing 50 latexµg from each of the Non-infected sample tubes.

### Dimethyl-labeling

To chemically-label the peptide mixtures prepared from ShSIRT1, ShScrambled and non-infected control samples, the dimethyl-labeling protocol was followed^91^. For ‘heavy’ labeling, Formaldehyde (13CD_2_O) in D_2_O and Sodium cyanoborodeuteride (NaBD_3_CN) were used. For ‘light’ labeling of the internal standard, Formaldehyde (CH_2_O) and Sodium cyanoborohydride (NaBH_3_CN) were used. A solution containing 1% Ammonia and 5% Formic acid was used to quench the labeling reaction after 1 h and the peptide samples labelled ‘heavy’ and ‘light’ were then mixed at a ratio of 1:1 prior to cleaning using R2/R3 Oligo beads.

### Peptide isoelectric-focusing

As described previously, the labelled peptide mixtures were fractionated using the 3100 OFFGEL fractionator kit (Agilent Technologies) on 12-cm immobilized pH gradient strips (IPG; pH 3–10)^92^. Rehydration and running buffer contained 0.1% IPG ampholyte solution and 0.3% glycerol. Peptides were focused using 50 μA current and 200 mW power to achieve a final voltage of 50 kV h. Peptide fractions were then incubated with 50 μl of a solution containing methanol, water, and trifluoroacetic acid at a ratio of 50:49:1 for 15 min prior to centrifugation in a SpeedVac vacuum concentrator (Eppendorf) and storage at − 80 °C. To solubilize the peptides, a solution containing 1% acetonitrile and 0.05% trifluoroacetic acid was used prior to desalting using C_18_ StageTips^93^. To pack the StageTips, 3 C_18_ disks were used and incubated with methanol for activation. Prior to loading, peptides were washed 2 times using a solution containing 2% acetonitrile and 0.1% trifluoroacetic acid. A solution containing 0.1% acetic acid and 2% acetonitrile and 0.1% trifluoroacetic acid was used to wash the StageTips prior to an elution step with 60% acetonitrile and 0.1% trifluoroacetic acid. Samples were dried by centrifugation in a SpeedVac vacuum concentrator and resuspended in mobile phase A (0.5% acetic acid) before mass-spectrometric analysis.

### Mass-spectrometry analysis

Peptide fractions were analyzed by nano-liquid chromatography (nLC) coupled to mass-spectrometry (MS) and the analytical platform comprised an EASY-nLC II (ThermoFisher Scientific) coupled to a Q Exactive mass-spectrometer (Thermo Scientific, Bremen, Germany). Conditions for chromatography were as follows: for mobile phase A, 0.5% acetic acid with H_2_O; for mobile phase B, H_2_O and acetonitrile mixed at a ratio of 20:80 with 0.5% acetic acid. The flow-rate, injection volume, and loading pressure were adjusted to 250 nl/min, 6 µl, and 280 bars, respectively. For LC separation, in-house packed emitter columns (ReproSil-Pur 120 C18-AQ) were used on a 5–30% mobile phase B gradient for 90 min prior to washing and column re-equilibration. After excluding singly-charged ions and charge states that were unassigned, higher energy collisional dissociation (HCD) was used to isolate and fragment the 10 most intense ions. To acquire the precursor scans, a resolution of 7 × 10^4^ at *m/z* 300 and AGC target value of 3 × 10^6^ charges was used. The fragmentation spectra were acquired at a resolution and AGC target value of 17.5 × 10^2^ at *m/z* 300 and 1 × 10^5^, respectively. We used an exclusion list of 25 s to record the scan events in profile mode, with functionalities like ‘exclude isotopes’ and ‘peptide match’ enabled.

### Proteomics data analysis

MaxQuant (1.6.10.43) and the Andromeda peptide search engine were used to analyze the mass-spec data^94,95^. To identify peptides, we set the enzyme specificity to trypsin with 2 maximum mis-cleavages: N-terminal to proline and between aspartic acid and proline. We used carbamidomethyl cysteine as a fixed modification and oxidized methionine/*N*-acetylation as variable modifications, as described previously^96^. The false discovery rate (FDR) and the minimum required peptide length were set to 0.01 and 7, respectively. We enabled both re-quantify and match-between runs during the analysis. Protein sequences were searched using the reviewed Swiss-Prot *Mus musculus* database (April 2018; 17,020 protein sequence entries).

MaxQuant results containing protein identification and corresponding dimethyl ratios were further analyzed using R version 3.6.1 (R Foundation for Statistical Computing, Vienna, Austria) and standard packages. Dimethyl peptide ratios were Log2-transformed and quantile-normalized. Data import, principal component analysis, and differential protein profiling were performed using the in-house autonomics package and limma^97^. PCA was performed using ‘mpm’ (http://mpm.r-forge.r-project.org) as backend, which incorporates PCA preceded by double-centering, the latter of which substantially improves interpretability and biological relevance of (transcript)omics data^98^. Functional over-representation analysis was performed with clusterProfiler^99^ using WikiPathways (11/2019 release) and Molecular Signatures Database v7.0 annotations^100,101^. For heatmaps, Log2-transformed ratios of protein subsets were scaled and clustered using complete Euclidean distance. Upstream regulator analysis was performed with Ingenuity Pathway Analysis (IPA) software (QIAGEN Bioinformatics, Aarhus, Denmark) using differential proteins (P value < 0.05).

### NMN treatment box plot and scatter plot analysis

The effects of NMN supplementation (+/−NMN) on gene-expression during adipogenesis were analysed across four conditions: ShScrambled D0 (preadipocyte) *vs* ShScrambled D6 (adipocyte) *vs* ShSIRT1 D0 (preadipocyte) *vs* ShSIRT1 D6 (adipocyte). NMN-induced changes in the expression of 51 genes were quantified by qPCR using 18S RNA as the reference gene. This set of genes not only included molecules predicted to be differentially expressed in ShScrambled *vs* ShSIRT1 adipocytes in proteomics studies but also genes predicted to be impacted by SIRT1-reduction from IPA and genes with established roles in adipogenesis. Fold-change was calculated as +NMN/−NMN for every gene in each of the four conditions and plotted as boxplots (Fig. 8) and scatterplots (Supplementary Figure S3a) in Matlab 2019a. To determine if the effects of NMN were statistically significant between the four conditions, the Kruskal–Wallis test was used, and P values were calculated comparing each condition using the Matlab 2019a 'kruskalwallis' and 'multcompare' built-in functions in the Statistics and Machine Learning Toolbox. To generate the fold-change scatterplots (Supplementary Figure S3b), the propagated standard error was calculated for each gene and error bars were plotted with a log2-scale adjustment.

### Data analysis and statistics

qPCR data were analyzed using GraphPad Prism 9.0 and presented as mean ± SEM. For statistical analysis, a Student’s *t* test (comparing 2 groups) or one-way ANOVA (comparing > 2 groups) was used, considering a ‘P’ value of < 0.05 as statistically significant. Only data generated from independent experiments was subjected to statistical analysis. The experimental ‘n’ and details of the statistical test are indicated in the Figure legends. Statistical analysis of the dataset presented in Fig. 8 is described above in the “NMN treatment box plot and scatter plot analysis” section.

## Supplementary Information

Supplementary Information.

## Data Availability

All the datasets generated in the current study are available from the corresponding author upon request.
