# Supplementary material for: SIRT1 promotes lipid metabolism and mitochondrial biogenesis in adipocytes and coordinates adipogenesis by targeting key enzymatic pathways
Source: Sci Rep. 2021 Apr 14;11:8177. doi: 10.1038/s41598-021-87759-x (PMC8046990; doi:10.1038/s41598-021-87759-x)
Supplement: Supplementary file 1 — Supplementary Information. [file 41598_2021_87759_MOESM1_ESM.pdf]

Supplementary Data

SIRT1 promotes lipid metabolism and mitochondrial biogenesis in adipocytes and coordinates adipogenesis by targeting key enzymatic pathways

Yasser Majeed[1], Najeeb Halabi[2,#], Aisha Y. Madani[1,3,#], Rudolf Engelke[4], Aditya M. Bhagwat[4,5], Houari Abdesselem[1,6], Maha V. Agha[1,7], Muneera Vakayil[1,3], Raphael Courjaret[8], Neha Goswami[4], Hisham Ben Hamidane[4,9], Mohamed A. Elrayess[10], Arash Rafii[2], Johannes Graumann[4,5], Frank Schmidt[4] and Nayef A. Mazloum[1]*

[1]Department of Microbiology and Immunology, Weill Cornell Medicine-Qatar, Qatar Foundation, Doha, Qatar
[2]Department of Genetic Medicine, Weill Cornell Medicine-Qatar, Qatar Foundation, Doha, Qatar
[3]College of Health & Life Sciences, Hamad Bin Khalifa University, Qatar Foundation, Doha, Qatar
[4]Department of Biochemistry, Weill Cornell Medicine-Qatar, Qatar Foundation, Doha, Qatar
[5]Biomolecular Mass Spectrometry, Max-Plank Institute for Heart and Lung Research, Ludwigstr 43,61231, Bad Nauheim, Germany
[6]Neurological Disorders Research Center, Qatar Biomedical Research Institute, Qatar Foundation, Doha, Qatar
[7]Interim Translational Research Institute, Hamad Medical Corporation, Doha, Qatar
[8]Department of Physiology and Biophysics, Weill Cornell Medicine-Qatar, Qatar Foundation, Doha, Qatar
[9]CSL Behring, Bern, Switzerland
[10]Biomedical Research Center, Qatar University, Doha, Qatar

*Corresponding author: Nayef A. Mazloum (nam2016@qatar-med.cornell.edu)

[#]Equal contributors

Supplementary Figures

Supplementary Figure 1: Validation of Sirt1-depletion by RNA-interference and effect of Sirt1-depletion on protein expression of target molecules. (a) Western blot data from 4 independent experiments used to derive the image presented in Figure 1a. Data show the effect of SIRT1-specific ShRNA (or Scrambled ShRNA) on the expression of SIRT1 protein in 3T3-L1 preadipocytes. Expression of β**-**actin served as a loading control. **(b)** Data showing quantification of proteins levels of SIRT1 in preadipocytes (n=4), and leptin, adiponectin, MMP3, MMP13, and Col6A3 (n=3) in ShScrambled and ShSIRT1 adipocytes at Day 6 post-differentiation (*, $P<0.05$; **, $P<0.01$; ***$P<0.0001$).

Supplementary Figure 2: Quantification of changes in SREBF1c targets and transcription factors. (a) Quantification of changes in levels of the indicated proteins in Sirt1-depleted adipocytes at Day 6 post-differentiation (n=3). **(b)** qPCR and western blotting data showing changes in mRNA and protein levels of the indicated transcription factors in Sirt1-depleted adipocytes at Day 6 post-differentiation. The boxed region in each panel indicates the time-point at which changes in protein expression were evaluated by western blotting (n=3) (*, P<0.05; **, P<0.01; ***P<0.0001).

Supplementary Figure 3: Profiling Sirt1-dependence of NMN-induced changes in gene-expression in 3T3-L1 (pre)adipocytes. (3a) Scatterplots showing the expression ratio of selected genes after NMN supplementation for **(a)** ShScrambled preadipocytes (D0) *vs* ShScrambled adipocytes (D6) and **(b)** ShSIRT1 preadipocytes (D0) *vs* ShSIRT1 adipocytes (D6). Dashed lines pass through 0 on the x- and y-axis and divide the data into quadrants, which are numbered 1 to 4 as indicated. <u>Quadrant 1:</u> Genes whose expression decreased with NMN supplementation in preadipocytes and adipocytes, <u>Quadrant 2:</u> Genes whose expression decreased with NMN supplementation in preadipocytes (D0) but increased in adipocytes (D6), <u>Quadrant 3:</u> Genes whose expression increased with NMN supplementation in preadipocytes (D0) and decreased in adipocytes (D6), and <u>Quadrant 4:</u> Genes whose expression increased with NMN supplementation in preadipocytes (D0) and adipocytes (D6). The points are colored based on the log2 expression value ratio of -NMN D6/-NMN D0. Error bars represent the propagated standard error. **(3b)** To compare NMN-induced changes in gene-expression across both ShScrambled and ShSIRT1 groups, gene-expression profiles are divided into groups based on the quadrant identities highlighted in Supplementary Figure 3a. In the Group column for each gene, the first and second number indicate its quadrant identity in ShScrambled and ShSIRT1 conditions, respectively. For example, group 44 represents genes whose expression was increased in response to NMN in both ShScrambled and ShSIRT1 in both preadipocytes and adipocytes. The +NMN/-NMN ratio indicated as green or red bars represents ratios of >1 and <1, respectively. The height of the bar is proportional to the ratio. Upward and downward arrows indicate an increase and decrease in the +NMN/-NMN ratio, respectively. The function or localization of each gene is indicated in the last column. For brevity, ShScrambled and ShSIRT1 groups are labelled as Mock and SIRT, respectively.

Supplementary Figure 4: Original western blotting data underlying representative images and quantification. Data from 3 independent experiments for the indicated proteins are shown. The dashed boxes indicate separate gels. After probing for the target protein, the membrane was stripped and re-probed for GAPDH.

Supplementary Figure 5: Original data from western blotting experiments underlying representative images and quantification. Data were generated from 3 independent experiments for the indicated proteins and dashed boxes indicate

separate gels. The membrane was stripped and re-probed for GAPDH after probing for the target protein.

Supplementary Figure 6: Original data from western blotting experiments underlying representative images and quantification. 3 independent experiments were performed to generate the data shown for the indicated proteins. After probing for the target protein, the membrane was stripped and re-probed for GAPDH. Dashed boxes indicate separate gels.

Supplementary Figure 7: Original western blotting data underlying representative images and quantification. Data are shown from 3 independent experiments for the indicated proteins and dashed boxes indicate separate gels. After probing for the target protein, the membrane was stripped and re-probed for GAPDH.

Supplementary Table 1: Sequences of the primer sets used in the study.

Gene (Mouse)	Forward Primer sequence	Reverse Primer sequence
SIRT1	CCAGACCCTCAAGCCATGTT	CTGTCCGGGATATATTTCCTTTGC
LEPTIN	CAGCTGCAAGGTGCAAGAAG	GATACCGACTGCGTGTGTGA
ADIPONECTIN	GATGCAGGTCTTCTTGGTCCT	ACATAAGCGGCTTCTCCAGG
MMP3	ACATGGAGACTTTGTCCCTTTTG	TTGGCTGAGTGGTAGAGTCCC
MMP13	TGTTTGCAGAGCACTACTTGAA	CAGTCACCTCTAAGCCAAAGAAA
NMNAT1	CAAGGCCTGACAACATCGCT	GAACAGCCTGAGGTGCATGT
NMNAT2	ACTCTGGAATGAGGCAGATATGG	TGCGGAGTATGGAGGAGTGA
NMNAT3	ACCAAACAGGAAGGTACCAGG	TCCACCCGAATCCAGTCAGA
CEBPα	TTCATGGAGAATGGGGGCAC	GGCTGGCGACATACAGTACA
CEBPδ	TCCACGACTCCTGCCATGTA	GTTGAAGAGGTCGGCGAAGA
CEBPβ	GCTGAGCGACGAGTACAAGA	CTTGAACAAGTTCCGCAGGG
PPARγ	ATTGAGTGCCGAGTCTGTGG	GCCCAAACCTGATGGCATTG
PPARα	TAATTTGCTGTGGAGATCGGC	GGTGTCATCTGGATGGTTGC
PGC-1α	ACACCGCAATTCTCCCTTGT	CGGCGCTCTTCAATTGCTTT
SREBF1C	ATGCCATGGGCAAGTACACA	AGATCTCTGCCAGTGTTGCC
COL6A1	TGATGGCATTCAAGGACCCC	TGGGCCAGCATTTCCTTCAT
COL1A1	ACGCCATCAAGGTCTACTGC	ACTCGAACGGGAATCCATCG
COL6A3	ACTGGAACCACGGAAGTTCA	GTCACTTCCAACATCGAGGC
COL6A2	CTTTGTCCAGACCCCCAGAT	GCACCTTGTGGAAGTTCTGC
AK4	ACCTCAAGTGCAGGGGATTG	CGTCCCAGAAAACTGGTGGA
NDUFA3	GTCTGGGGCCTCGCTATAAT	TCTCTCACAGGCACTGGGTA
NDUFB4	TCCAAGTATAAGCCTGCGCC	AAGGCAGGATCCTCGATGTG

NDUFB6	TGAAGGACCAGGAGCTGAGT	CGGTACGCCTTAAAGACCATGT
NDUFB8	TGGGGTGAACCGATACACTG	TAAGGGTACTGCTTCGGACC
CSRP1	ACCCAGAAGGGACAAGATGGA	CCCCAGTTTGGCATTCTGCT
AACS	GTGCACTAAGCCCATTCCCT	TTGAGGGTGCCATCACTACG
CYB5A	GCCAAGCCTTCGGATACTCT	CGCTCTGGGGCAGTCTTTTA
SCD1	CAGGTTTCCAAGCGCAGTTC	GAACTGGAGATCTCTTGGAGCA
SCD2	GAGCAGATGTTCGCCCTGAA	ACAAATACGCGAAGAGACAGGT
ELOVL6	AAGCACCCGAACTAGGTGAC	CCGCAAGGCGTAGTAAGAGT
HMGCR	CCTGCAGATGCTAGGTGTTCA	CTGCCATCAAGGACAGCTCA
18S RNA	GTAACCCGTTGAACCCCATT	CCATCCAATCGGTAGTAGCG
NDUFB3	GATCCATGGGCTCGCAATGA	GCTACCACAAACGCAGCAAA
NDUFB5	GACGCTCGCTTCTTGAGGTT	GCAAGCTCGGCTTCACCAAT
TBC1D10A	AGTTCGGATTCATCGTGGGT	CTGGCATCGAAGACGAATCTTT
TRPM7	AGCATTTGTGGGACACAGAGA	TTTCTGCTTGCACCGAGTGA
GCHFR	AGCACTCAGATCCGTATGGAGG	GTCATGCTCAGCACTCGGAA
NOP9	GAAGAACGAGAGTTGATGGCG	ACACTCGACACAGGGGTTTC
LCN2	ACCCTGTATGGAAGAACCAAGG	TCGGTGGGGACAGAGAAGAT
GSTA3	GGCTGAGCAGGGCTGATATT	GCTTCTCAGCGCTTTCAGGA
IL1RN	GAGTGAGACGTTGGAAGGCA	TCCCAGATTCTGAAGGCTTGC
DOLPP1	ACCCTGCAGGTGATCTGTCT	GTGTGTAGCTCCCGCTTGAA
THRSP	GGCCGAAGAAGACAGGATCT	TCTCGTGTAAAGCGATCTTCAG
AGPAT2	TACGCCAAGGTCGGTCTCTA	ACCAGCTGATGATGCTCATGT
GPAM	ATTCTCTTCACCGCCAGCAA	TCGAAATCGCGAGCTAGGAC
FAS	GGAGGTGGTGATAGCCGGTAT	TGGGTAATCCATAGAGCCCAG
MMP11	ATTGGGGACAACCAGGGAAC	AGGGTAGCGGAAGGTGTAGA
CDK1	ACAGAGAGGGTCCGTCGTAA	ATTGCAGTACTGGGCACTCC
CDK2	ACTCATGAGGTGGTGACCCT	CATGGTGCTGGGTACACACT
IL-6	CTGCAAGAGACTTCCATCCAG	AGTGGTATAGACAGGTCTGTTGG
CXCL10	CCAAGTGCTGCCGTCATTTTC	GGCTCGCAGGGATGATTTCAA
CXCL1	CCGAAGTCATAGCCACACTCA	GTGCCATCAGAGCAGTCTGT
CTSZ	TGCAACAACTACCAGGCCAA	GCCATTATCCCGCAGCTGAT
LOXL1	TATGCCTGCACCTCTCACAC	GTTCACGTGCACCTTGAGGA
EHHADH	CGGTTGGTGTTCTTGGCTTG	GCTGCTTTGGGTCTGACTCT
CARS2	TGCTATGGGAGAGGCTGACT	TTGGGCCACTAGCTTCCTTG

Supplementary Figure 1

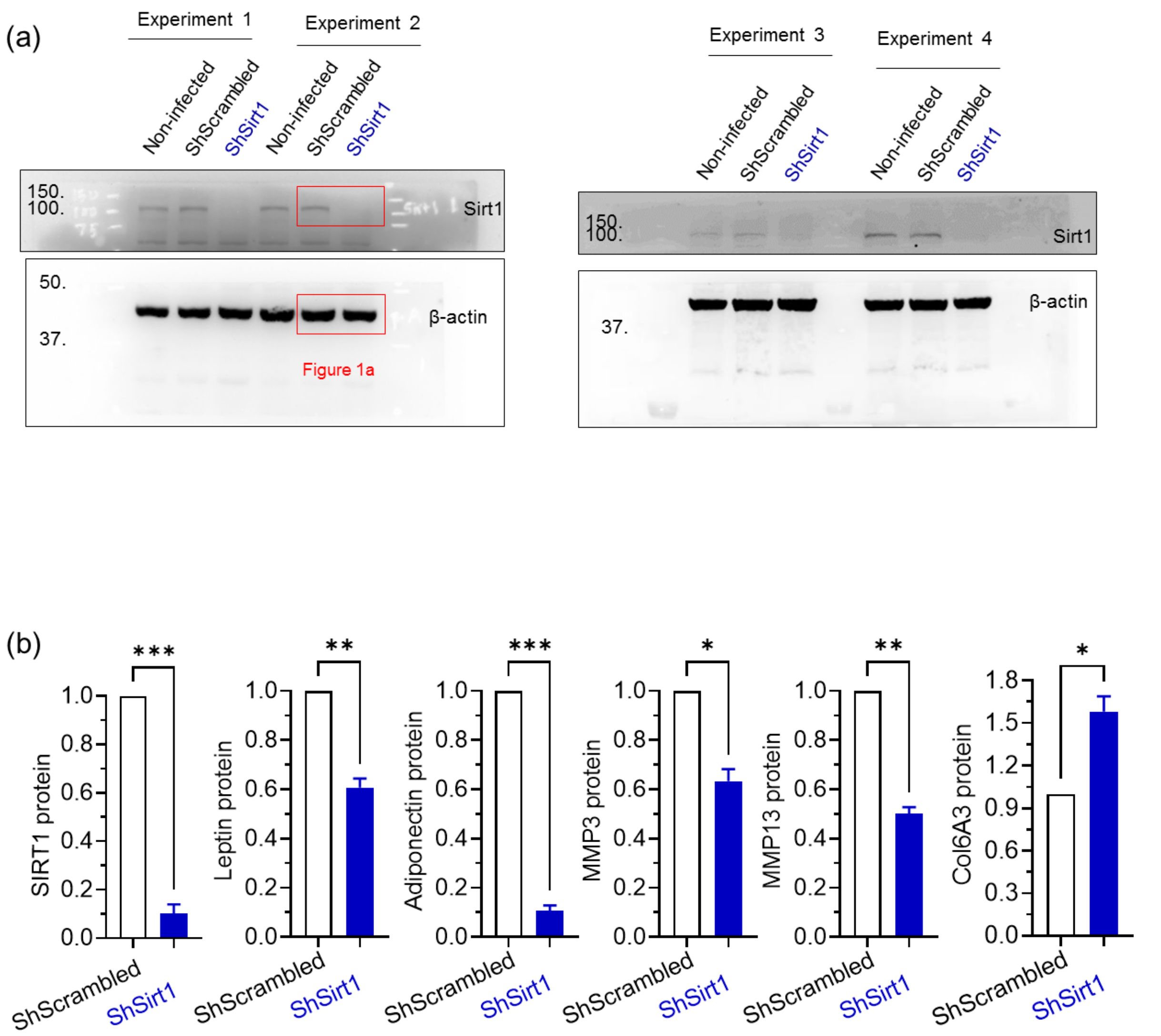

Supplementary Figure 2

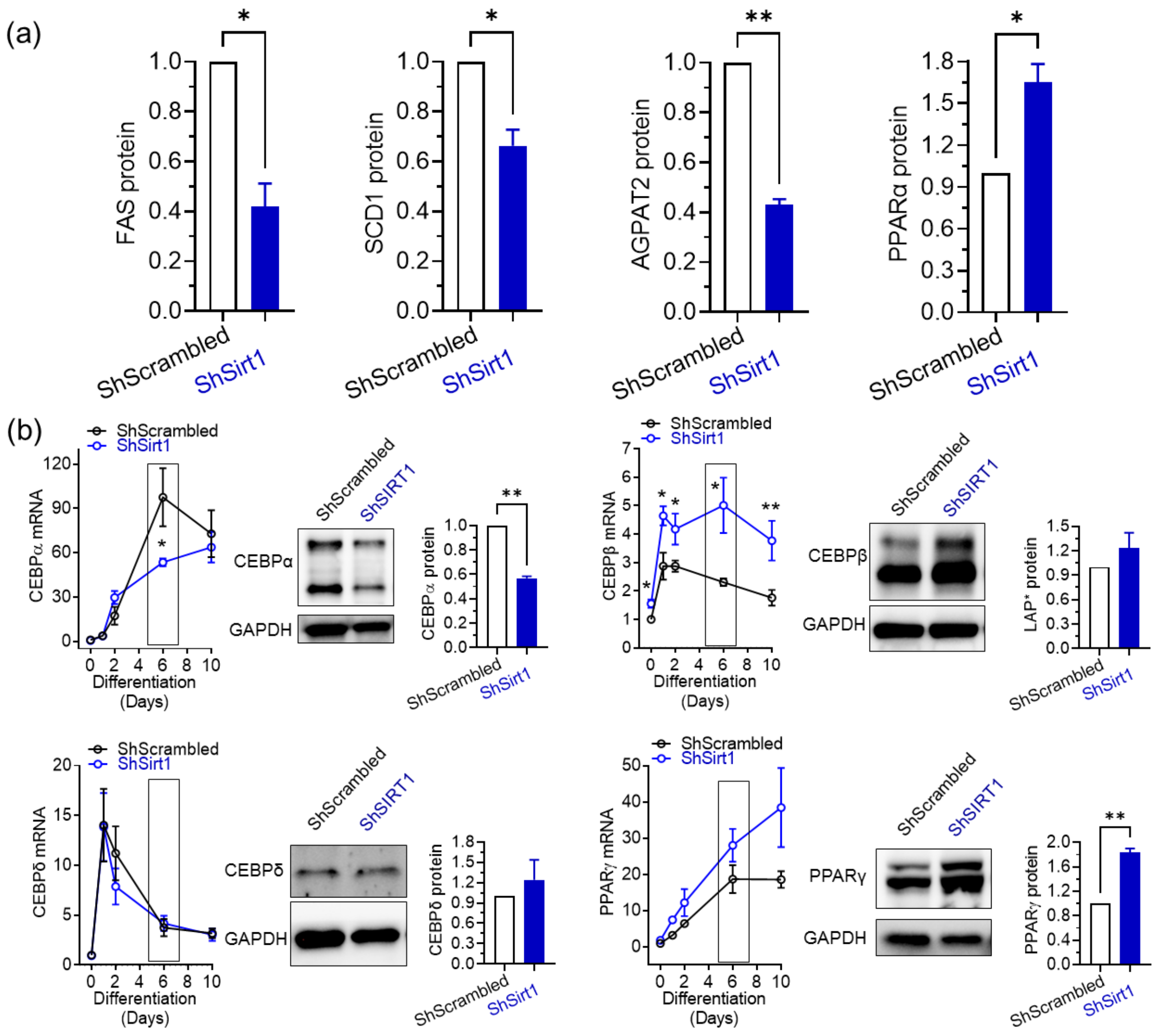

Supplementary Figure 3a

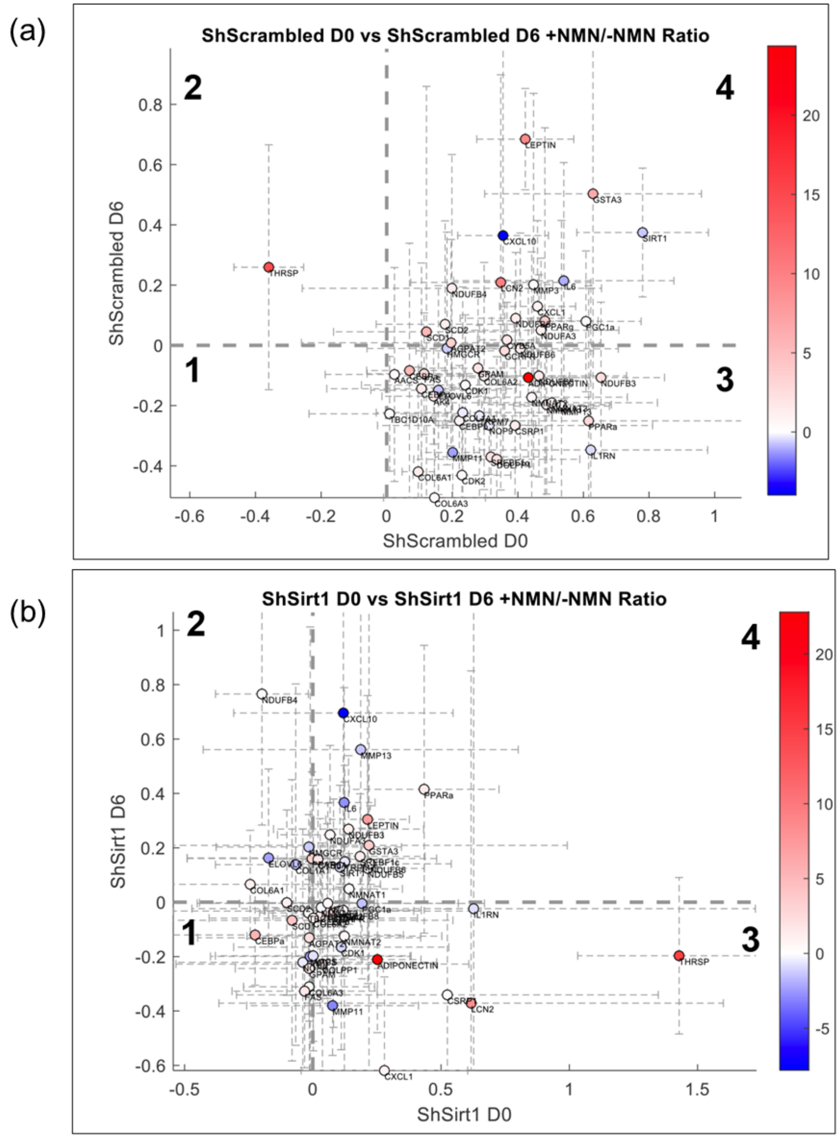

Supplementary Figure 3b

Gene Symbol	Group	NMN Induced Directional Change MOCK d0	MOCK d6	SIRT d0	SIRT d6	NMN Induced Transcriptional Change (+NMN/-NMN -1) MOCK d0	MOCK d6	SIRT d0	SIRT d6	Gene Name	GENE MOLECULAR TYPE OR LOCALIZATION
THRSP	23	▼	▲	▲	▼					thyroid hormone responsive	Nucleus
CEBPA	31	▲	▼	▼	▼					CCAAT enhancer binding protein alpha	transcription regulator
COL6A3		▲	▼	▼	▼					collagen type VI alpha 3 chain	Extracellular Space
FAS		▲	▼	▼	▼					Fas cell surface death receptor	transmembrane receptor
GPAM		▲	▼	▼	▼					glycerol-3-phosphate acyltransferase, mitochondrial	enzyme
NOP9		▲	▼	▼	▼					NOP9 nucleolar protein	Nucleus
TBC1D10A		▲	▼	▼	▼					TBC1 domain family member 10A	Plasma Membrane
COL1A1	32	▲	▼	▼	▲					collagen type I alpha 1 chain	Extracellular Space
COL6A1		▲	▼	▼	▲					collagen type VI alpha 1 chain	Extracellular Space
ELOVL6		▲	▼	▼	▲					ELOVL fatty acid elongase 6	enzyme
HMGCR		▲	▼	▼	▲					3-hydroxy-3-methylglutaryl-CoA reductase	enzyme
AACS	33	▲	▼	▲	▼					acetoacetyl-CoA synthetase	enzyme
ADIPOQ		▲	▼	▲	▼					adiponectin, C1Q and collagen domain containing	Extracellular Space
AK4		▲	▼	▲	▼					adenylate kinase 4	kinase
CDK1		▲	▼	▲	▼					cyclin dependent kinase 1	kinase
CDK2		▲	▼	▲	▼					cyclin dependent kinase 2	kinase
CEBPb		▲	▼	▲	▼					CCAAT enhancer binding protein beta	transcription regulator
CEBPd		▲	▼	▲	▼					CCAAT enhancer binding protein delta	transcription regulator
COL6A2		▲	▼	▲	▼					collagen type VI alpha 2 chain	Extracellular Space
CSRP1		▲	▼	▲	▼					cysteine and glycine rich protein 1	Nucleus
DOLPP1		▲	▼	▲	▼					dolichyldiphosphatase 1	enzyme
GCHFR		▲	▼	▲	▼					GTP cyclohydrolase I feedback regulator	Cytoplasm
IL1RN		▲	▼	▲	▼					interleukin 1 receptor antagonist	cytokine
MMP11		▲	▼	▲	▼					matrix metallopeptidase 11	peptidase
NDUFB8		▲	▼	▲	▼					NADH:ubiquinone oxidoreductase subunit B8	enzyme
NMNAT2		▲	▼	▲	▼					nicotinamide nucleotide adenylyltransferase 2	enzyme
NMNAT3		▲	▼	▲	▼					nicotinamide nucleotide adenylyltransferase 3	enzyme
MMP13	34	▲	▼	▲	▲					matrix metallopeptidase 13	peptidase
NDUFB3		▲	▼	▲	▲					NADH:ubiquinone oxidoreductase subunit B3	enzyme
NDUFB6		▲	▼	▲	▲					NADH:ubiquinone oxidoreductase subunit B6	enzyme
NMNAT1		▲	▼	▲	▲					nicotinamide nucleotide adenylyltransferase 1	enzyme
PPARA		▲	▼	▲	▲					peroxisome proliferator activated receptor alpha	ligand-dependent nuclear receptor
SREBF1c		▲	▼	▲	▲					sterol regulatory element binding transcription factor 1 (isoform c)	transcription regulator
TRPM7		▲	▼	▲	▲					transient receptor potential cation channel subfamily M member 7	kinase
AGPAT2	41	▲	▲	▼	▼					1-acylglycerol-3-phosphate O-acyltransferase 2	enzyme
MMP3		▲	▲	▼	▼					matrix metallopeptidase 3	peptidase
SCD		▲	▲	▼	▼					stearoyl-CoA desaturase	enzyme
SCD2		▲	▲	▼	▼					stearoyl-Coenzyme A desaturase 2	enzyme
NDUFB4	42	▲	▲	▼	▲					NADH:ubiquinone oxidoreductase subunit B4	transporter
PPARG		▲	▲	▼	▲					peroxisome proliferator activated receptor gamma	ligand-dependent nuclear receptor
CXCL1	43	▲	▲	▲	▼					C-X-C motif chemokine ligand 1	cytokine
LCN2		▲	▲	▲	▼					lipocalin 2	transporter
PPARGC1A		▲	▲	▲	▼					PPARG coactivator 1 alpha	transcription regulator
CXCL10	44	▲	▲	▲	▲					C-X-C motif chemokine ligand 10	cytokine
CYB5A		▲	▲	▲	▲					cytochrome b5 type A	enzyme
GSTA3		▲	▲	▲	▲					glutathione S-transferase alpha 3	enzyme
IL6		▲	▲	▲	▲					interleukin 6	cytokine
LEP		▲	▲	▲	▲					leptin	growth factor
NDUFA3		▲	▲	▲	▲					NADH:ubiquinone oxidoreductase subunit A3	enzyme
NDUFB5		▲	▲	▲	▲					NADH:ubiquinone oxidoreductase subunit B5	enzyme
SIRT1		▲	▲	▲	▲					sirtuin 1	transcription regulator

Supplementary Figure 4

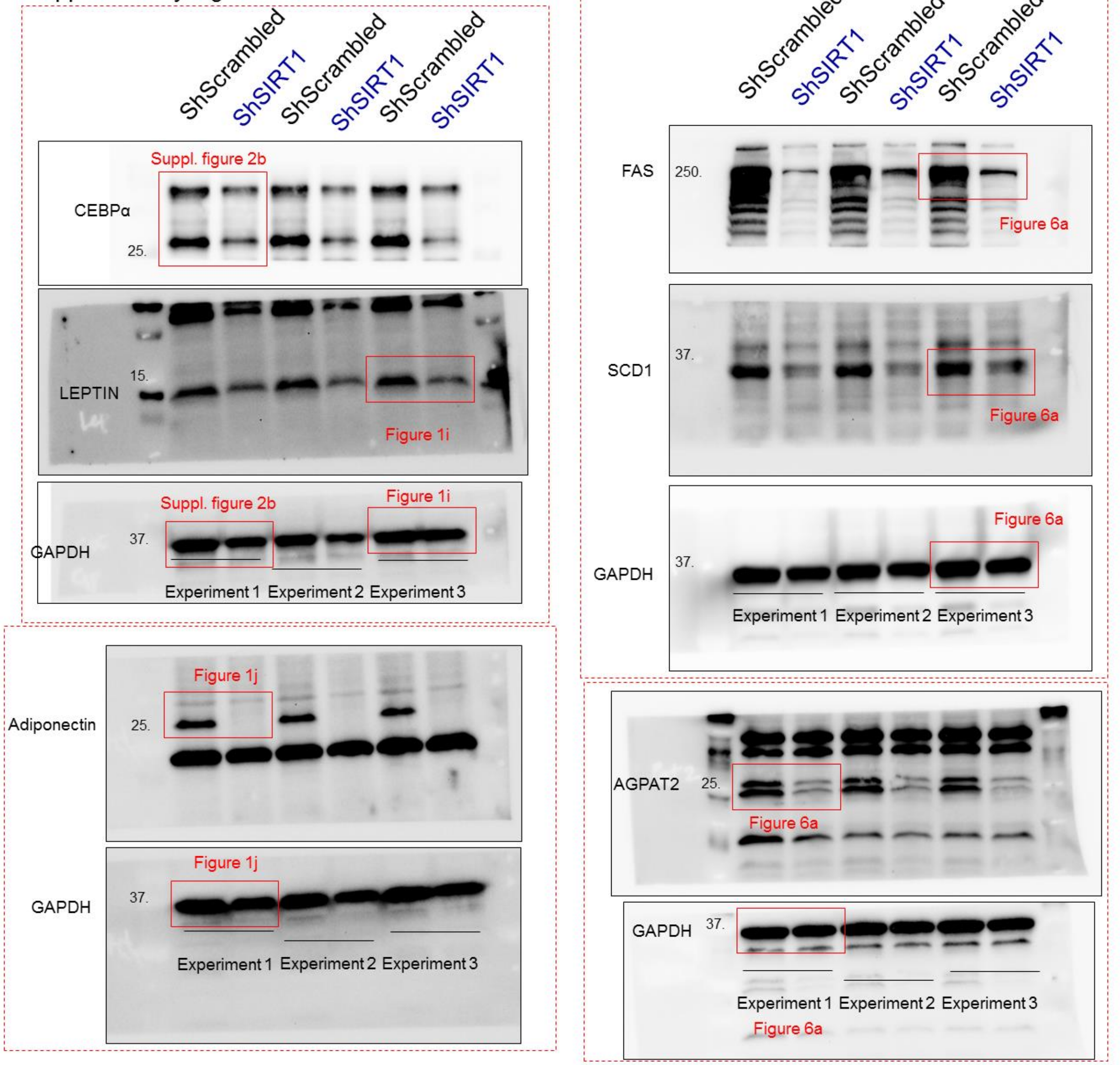

Supplementary Figure 5

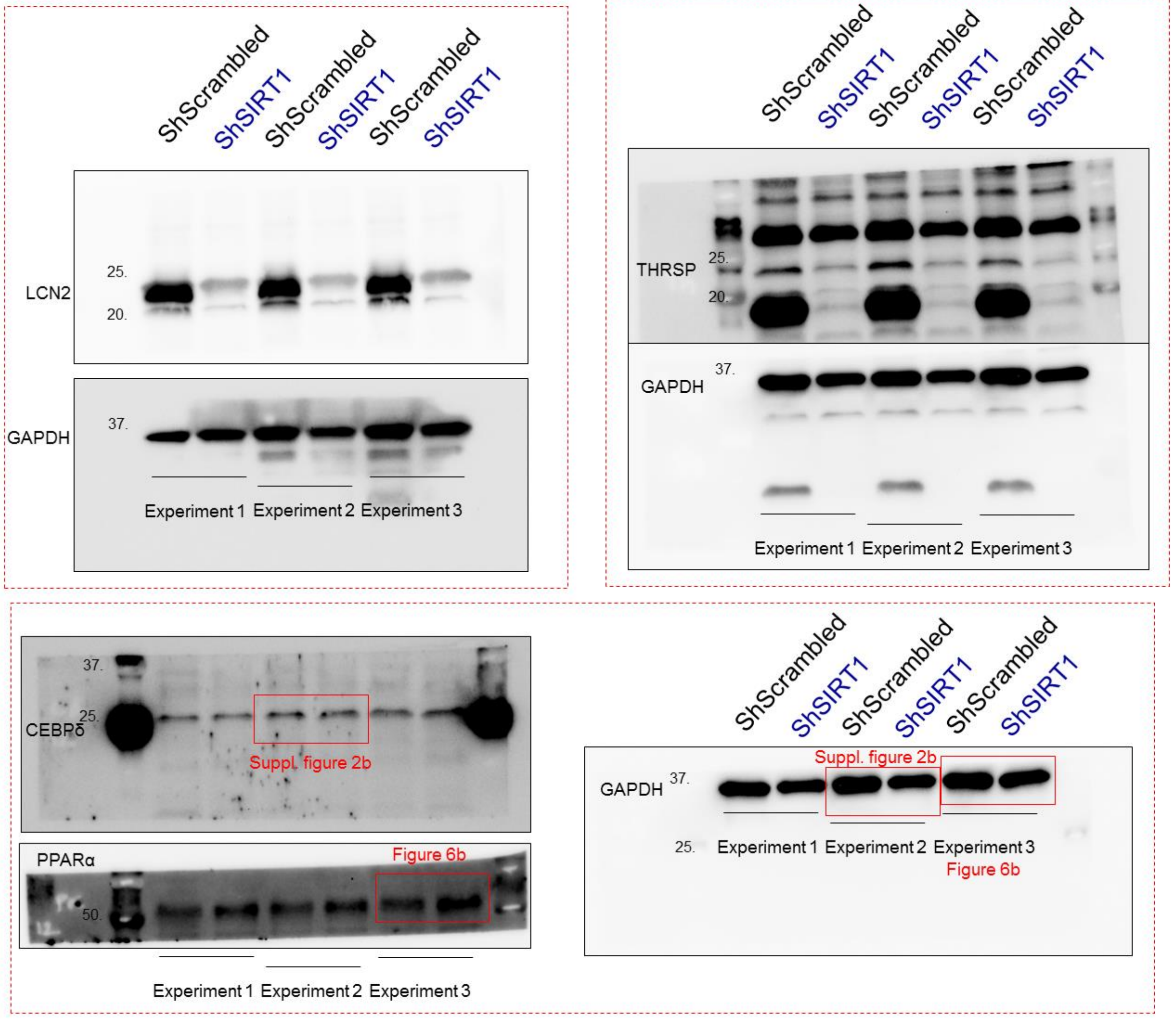

Supplementary Figure 6

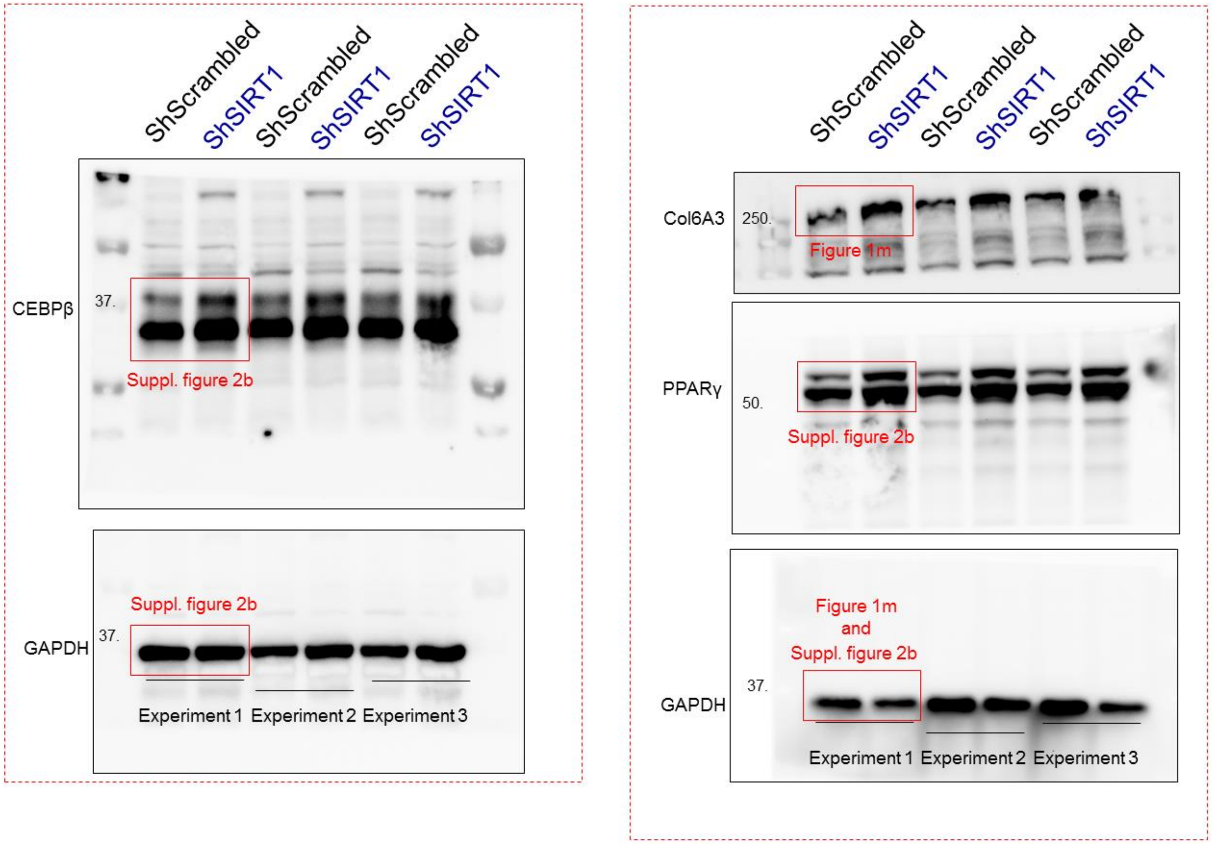

Supplementary Figure 7

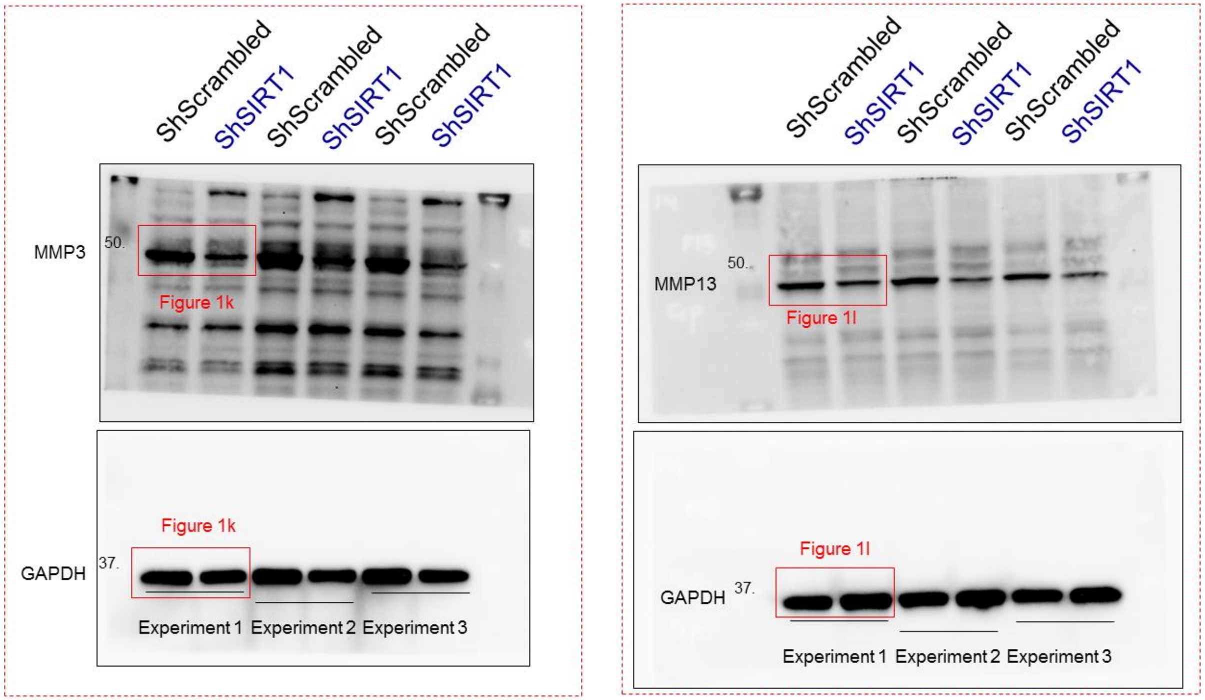

Supplementary Table 2: The table contains the full proteomics dataset.

PROTEOMICS DATASET

Contrast	ID	Uniprot AC	Gene	EntrezId	Effect [log ratio]	P	FDR
ShSirt1_vs_ShScrambled	P21981	P21981	Tgm2	21817	2.214586886	1.9312E-06	0.00415633
ShSirt1_vs_ShScrambled	Q62264	Q62264	Thrsp	21835	-2.875075163	2.3915E-06	0.00415633
ShSirt1_vs_ShScrambled	P41216	P41216	Acsl1	14081	-2.183237896	2.7949E-06	0.00415633
ShSirt1_vs_ShScrambled	Q8BJ56	Q8BJ56	Pnpla2	66853	-1.915867422	3.6831E-06	0.00415633
ShSirt1_vs_ShScrambled	O55239	O55239	Nnmt	18113	-2.329187769	5.2901E-06	0.00437837
ShSirt1_vs_ShScrambled	Q8K0C4	Q8K0C4	Cyp51a1	13121	-2.850313435	6.0778E-06	0.00437837
ShSirt1_vs_ShScrambled	Q91WU0	Q91WU0	Ces1f	234564	-2.315737112	7.3224E-06	0.00437837
ShSirt1_vs_ShScrambled	Q8K3K7	Q8K3K7	Agpat2	67512	-1.777352794	8.3689E-06	0.00437837
ShSirt1_vs_ShScrambled	Q01149	Q01149	Col1a2	12843	-2.354437003	9.0379E-06	0.00437837
ShSirt1_vs_ShScrambled	Q9QX15	Q9QX15	Clca3a1	12722	-1.93318898	9.6995E-06	0.00437837
ShSirt1_vs_ShScrambled	P14901	P14901	Hmox1	15368	-1.616725618	1.3074E-05	0.0052416
ShSirt1_vs_ShScrambled	P18242	P18242	Ctsd	13033	-1.635818143	1.4624E-05	0.0052416
ShSirt1_vs_ShScrambled	P20108	P20108	Prdx3	11757	-2.335892784	1.5095E-05	0.0052416
ShSirt1_vs_ShScrambled	P13707	P13707	Gpd1	14555	-2.051577912	1.6674E-05	0.00537612
ShSirt1_vs_ShScrambled	P02468	P02468	Lamc1		1.416010408	2.2786E-05	0.00685698
ShSirt1_vs_ShScrambled	P99025	P99025	Gchfr	320415	2.226629483	2.5379E-05	0.00716005
ShSirt1_vs_ShScrambled	P19096	P19096	Fasn	14104	-1.345735934	3.367E-05	0.00877062
ShSirt1_vs_ShScrambled	P25085	P25085	Il1rn	16181	-3.066518971	3.4974E-05	0.00877062
ShSirt1_vs_ShScrambled	P11087	P11087	Col1a1	12842	-2.386056004	3.8801E-05	0.00890393
ShSirt1_vs_ShScrambled	Q60994	Q60994	Adipoq	11450	-1.645028686	3.945E-05	0.00890393
ShSirt1_vs_ShScrambled	P26443	P26443	Glud1	14661	-1.408348043	4.2804E-05	0.00903978
ShSirt1_vs_ShScrambled	P06801	P06801	Me1	17436	-1.402079827	4.4057E-05	0.00903978
ShSirt1_vs_ShScrambled	Q05793	Q05793	Hspg2	15530	1.343152171	4.8347E-05	0.00936528
ShSirt1_vs_ShScrambled	P08121	P08121	Col3a1	12825	-2.153914483	5.1421E-05	0.00936528
ShSirt1_vs_ShScrambled	Q9DCN1	Q9DCN1	Nudt12	67993	-2.066330337	5.3059E-05	0.00936528
ShSirt1_vs_ShScrambled	Q8CFE6	Q8CFE6	Slc38a2	67760	-1.524496322	5.4024E-05	0.00936528
ShSirt1_vs_ShScrambled	P17563	P17563;Q63	Selenbp1		1.681130742	5.6017E-05	0.00936528
ShSirt1_vs_ShScrambled	P02463	P02463	Col4a1	12826	-3.187161307	5.8135E-05	0.00937216
ShSirt1_vs_ShScrambled	P97927	P97927	Lama4	16775	1.210526918	6.2883E-05	0.00978807
ShSirt1_vs_ShScrambled	Q8R2Y2	Q8R2Y2	Mcam	84004	-2.135032435	6.5543E-05	0.00986207
ShSirt1_vs_ShScrambled	Q7TN75	Q7TN75	Peg10	170676	1.466623101	7.2552E-05	0.01056453
ShSirt1_vs_ShScrambled	P47739	P47739	Aldh3a1	11670	-1.84667899	7.8027E-05	0.01075027
ShSirt1_vs_ShScrambled	Q6PIE5	Q6PIE5;Q6P	Atp1a2		-2.057931816	7.9126E-05	0.01075027
ShSirt1_vs_ShScrambled	Q9CYA6	Q9CYA6	Zcchc8	70650	1.590467771	8.0972E-05	0.01075027
ShSirt1_vs_ShScrambled	Q9D6J5	Q9D6J5	Ndufb8	67264	-1.325992668	9.0446E-05	0.011665
ShSirt1_vs_ShScrambled	P62075	P62075	Timm13	30055	-1.129931598	0.00011263	0.01412241
ShSirt1_vs_ShScrambled	P01831	P01831	Thy1	21838	-2.204986001	0.00013447	0.01617562
ShSirt1_vs_ShScrambled	Q80Y98	Q80Y98	Ddhd2	72108	-1.13050311	0.00013617	0.01617562
ShSirt1_vs_ShScrambled	P33267	P33267	Cyp2f2	13107	-1.195247632	0.00014154	0.01638278
ShSirt1_vs_ShScrambled	Q9Z222	Q9Z222	B3GNT2	53625	-2.53980161	0.00015307	0.0172739
ShSirt1_vs_ShScrambled	Q61881	Q61881	Mcm7	17220	1.15249822	0.00017368	0.01819864
ShSirt1_vs_ShScrambled	P52825	P52825	Cpt2	12896	-1.094965605	0.00017421	0.01819864
ShSirt1_vs_ShScrambled	Q8K354	Q8K354	Cbr3	109857	-1.176180135	0.00018004	0.01819864
ShSirt1_vs_ShScrambled	P36552	P36552	Cpox	12892	0.997721205	0.00018022	0.01819864
ShSirt1_vs_ShScrambled	Q8JZN5	Q8JZN5	Acad9	229211	-0.922056191	0.00018408	0.01819864
ShSirt1_vs_ShScrambled	Q8K009	Q8K009	Aldh1l2	216188	0.965381552	0.00018868	0.01819864
ShSirt1_vs_ShScrambled	Q921X9	Q921X9	Pdia5	72599	1.082182884	0.00018949	0.01819864
ShSirt1_vs_ShScrambled	Q9CZ28	Q9CZ28	Snf8	27681	2.16857413	0.00019879	0.01833989
ShSirt1_vs_ShScrambled	Q9Z211	Q9Z211	Pex11a	18631	-1.511115313	0.00019908	0.01833989
ShSirt1_vs_ShScrambled	Q9R002	Q9R002	Ifi202	26388	-1.396880131	0.00020339	0.01836164
ShSirt1_vs_ShScrambled	Q8C0N2	Q8C0N2	Agpat9	231510	-1.211258714	0.00021752	0.01925271
ShSirt1_vs_ShScrambled	P03953	P03953	Cfd	11537	-2.592216584	0.0002285	0.01947039
ShSirt1_vs_ShScrambled	Q99M51	Q99M51	Nck1	17973	-1.015105996	0.00023206	0.01947039
ShSirt1_vs_ShScrambled	Q3V3R1	Q3V3R1	Mthfd1l	270685	1.109855125	0.00023819	0.01947039
ShSirt1_vs_ShScrambled	Q9CQ91	Q9CQ91	Ndufa3	66091	-1.337281898	0.00024353	0.01947039
ShSirt1_vs_ShScrambled	P28650	P28650	Adssl1	11565	1.354765727	0.00024424	0.01947039

ShSirt1_vs_ShScrambled	Q9R0N0	Q9R0N0	Galk1	14635	0.966401411	0.00025314	0.01947039
ShSirt1_vs_ShScrambled	Q8JZK9	Q8JZK9	Hmgcs1	208715	-1.123566141	0.00025543	0.01947039
ShSirt1_vs_ShScrambled	P53668	P53668	Limk1	16885	1.365041568	0.00025564	0.01947039
ShSirt1_vs_ShScrambled	O88207	O88207	Col5a1	12831	-0.993571493	0.00026171	0.01947039
ShSirt1_vs_ShScrambled	O08739	O08739	Ampd3	11717	2.338823668	0.00026311	0.01947039
ShSirt1_vs_ShScrambled	P52840	P52840	Sult1a1		-1.378528016	0.00028294	0.02060017
ShSirt1_vs_ShScrambled	P00920	P00920	Ca2	12349	-6.262803337	0.00029406	0.02106952
ShSirt1_vs_ShScrambled	Q8QZR5	Q8QZR5	Gpt	76282	-1.564291181	0.00031231	0.02202752
ShSirt1_vs_ShScrambled	Q64429	Q64429	Cyp1b1	13078	-1.575853026	0.00033133	0.02300941
ShSirt1_vs_ShScrambled	P24270	P24270	Cat	12359	-1.348346117	0.00034298	0.02336823
ShSirt1_vs_ShScrambled	Q62048	Q62048	Pea15	18611	-1.279653623	0.00035012	0.02336823
ShSirt1_vs_ShScrambled	Q8K010	Q8K010	Oplah	75475	-1.19957085	0.00035864	0.02336823
ShSirt1_vs_ShScrambled	Q9JM51	Q9JM51	Ptges	64292	-1.119930737	0.00036337	0.02336823
ShSirt1_vs_ShScrambled	P00375	P00375	Dhfr	13361	1.364604362	0.00036415	0.02336823
ShSirt1_vs_ShScrambled	Q3TN34	Q3TN34	Micall2	231830	4.215565762	0.00037213	0.02336823
ShSirt1_vs_ShScrambled	O88455	O88455	Dhcr7	13360	-1.281883585	0.00037273	0.02336823
ShSirt1_vs_ShScrambled	Q9JLV6	Q9JLV6	Pnkp		1.104586478	0.00039609	0.02426097
ShSirt1_vs_ShScrambled	O55242	O55242	Sigmar1	18391	-0.983390806	0.00039772	0.02426097
ShSirt1_vs_ShScrambled	P51660	P51660	Hsd17b4	15488	-0.904653061	0.00040716	0.0245058
ShSirt1_vs_ShScrambled	Q8R1G6	Q8R1G6	Pdlim2	213019	0.86481226	0.00041493	0.02462532
ShSirt1_vs_ShScrambled	Q9CQ54	Q9CQ54	Ndufc2	68197	-1.22133645	0.00042467	0.02462532
ShSirt1_vs_ShScrambled	O89017	O89017	Lgmn	19141	-1.354192611	0.00042552	0.02462532
ShSirt1_vs_ShScrambled	P10493	P10493	Nid1	18073	1.632233431	0.00043105	0.02462974
ShSirt1_vs_ShScrambled	P23249	P23249	Mov10	17454	0.815090774	0.00043886	0.02476286
ShSirt1_vs_ShScrambled	P42337	P42337	Pik3ca	18706	-2.211764904	0.00044675	0.02489653
ShSirt1_vs_ShScrambled	Q9Z1P6	Q9Z1P6	Ndufa7	66416	-0.948246984	0.00046029	0.02533858
ShSirt1_vs_ShScrambled	Q9CQH3	Q9CQH3	Ndufb5	66046	-1.087721983	0.0004736	0.02575705
ShSirt1_vs_ShScrambled	Q91X52	Q91X52	Dcxr	67880	0.812799586	0.00048256	0.02593195
ShSirt1_vs_ShScrambled	Q8BJH1	Q8BJH1	Zc2hc1a	67306	2.105055296	0.00051547	0.02677902
ShSirt1_vs_ShScrambled	Q2TPA8	Q2TPA8	Hsdl2	72479	-1.231337079	0.00051658	0.02677902
ShSirt1_vs_ShScrambled	Q61553	Q61553	Fscn1	14086	0.796589283	0.00053801	0.02677902
ShSirt1_vs_ShScrambled	P55194	P55194	Sh3bp1	20401	1.066920024	0.00054456	0.02677902
ShSirt1_vs_ShScrambled	Q8CHS8	Q8CHS8	Vps37a	52348	-1.754970607	0.00054921	0.02677902
ShSirt1_vs_ShScrambled	P98197	P98197	Atp11a	50770	-1.217696808	0.0005505	0.02677902
ShSirt1_vs_ShScrambled	Q7TNV0	Q7TNV0	Dek	110052	1.195561706	0.00055874	0.02677902
ShSirt1_vs_ShScrambled	Q8R3V5	Q8R3V5	Sh3glb2	227700	-1.038771633	0.00055935	0.02677902
ShSirt1_vs_ShScrambled	Q05CL8	Q05CL8	Larp7	28036	1.136198522	0.00056886	0.02677902
ShSirt1_vs_ShScrambled	Q9DBS1	Q9DBS1	Tmem43	74122	-0.911381924	0.00057024	0.02677902
ShSirt1_vs_ShScrambled	Q6ZWY9	Q6ZWY9;Q6	Hist1h2bc		1.490983196	0.00057369	0.02677902
ShSirt1_vs_ShScrambled	P12382	P12382	Pfkl	18641	0.802602	0.00057637	0.02677902
ShSirt1_vs_ShScrambled	Q61183	Q61183	Papola	18789	1.743898958	0.00057901	0.02677902
ShSirt1_vs_ShScrambled	Q9D6Y7	Q9D6Y7	Msra	110265	-1.184676051	0.00058138	0.02677902
ShSirt1_vs_ShScrambled	Q91VB4	Q91VB4	Hps3	12807	-3.570692196	0.0005952	0.02678044
ShSirt1_vs_ShScrambled	P48036	P48036	Anxa5	11747	-0.757518112	0.00059587	0.02678044
ShSirt1_vs_ShScrambled	P56391	P56391	Cox6b1	110323	-1.774964148	0.00059921	0.02678044
ShSirt1_vs_ShScrambled	Q80UU1	Q80UU1	Ankzf1	52231	0.954905896	0.00065041	0.02869344
ShSirt1_vs_ShScrambled	Q61029	Q61029	Tmpo	21917	0.974448882	0.00066463	0.02869344
ShSirt1_vs_ShScrambled	Q80XL6	Q80XL6	Acad11	102632	-1.037568756	0.00067075	0.02869344
ShSirt1_vs_ShScrambled	P13516	P13516	Scd1	20249	-1.876271245	0.00067246	0.02869344
ShSirt1_vs_ShScrambled	Q80UW2	Q80UW2	Fbxo2	230904	-2.780987248	0.00067379	0.02869344
ShSirt1_vs_ShScrambled	Q61206	Q61206	Pafah1b2	18475	-1.069758543	0.00069196	0.02919162
ShSirt1_vs_ShScrambled	Q8BFQ6	Q8BFQ6	Dirc2	224132	-0.997244546	0.00070323	0.02939234
ShSirt1_vs_ShScrambled	Q80TV8	Q80TV8	Clasp1	76707	-0.92402584	0.00072074	0.02984796
ShSirt1_vs_ShScrambled	O88736	O88736	Hsd17b7	15490	-1.095941733	0.0007285	0.0298951
ShSirt1_vs_ShScrambled	Q6ZQM8	Q6ZQM8	Ugt1a7c	394432	-1.453164352	0.00075313	0.0306272
ShSirt1_vs_ShScrambled	P15105	P15105	Glul	14645	-1.55690082	0.00076136	0.03068562
ShSirt1_vs_ShScrambled	Q9CYV5	Q9CYV5	Tmem135	72759	-1.089795351	0.00078472	0.03134696
ShSirt1_vs_ShScrambled	Q8K4Q8	Q8K4Q8	Colec12	140792	-0.961986417	0.00079961	0.03166172
ShSirt1_vs_ShScrambled	Q9D6Y9	Q9D6Y9	Gbe1	74185	-1.087611909	0.00080757	0.03169881
ShSirt1_vs_ShScrambled	P70663	P70663	Sparcl1	13602	2.222823081	0.00081987	0.03190442
ShSirt1_vs_ShScrambled	P62869	P62869	Tceb2	67673	-0.976379182	0.00083	0.03202242
ShSirt1_vs_ShScrambled	Q8R1K4	Q8R1K4	Phykpl	72947	1.545244769	0.00083999	0.03213315
ShSirt1_vs_ShScrambled	Q8VCT4	Q8VCT4	Ces1d	104158	-1.314520784	0.00086003	0.03262338

ShSirt1_vs_ShScrambled	Q9JIL4	Q9JIL4	Pdzk1	59020	-0.931884283	0.0008923	0.03356534
ShSirt1_vs_ShScrambled	P52293	P52293	Kpna2	16647	-1.381545228	0.00090082	0.03360591
ShSirt1_vs_ShScrambled	E9Q735	E9Q735	Ube4a	140630	-0.92281595	0.00090963	0.03365624
ShSirt1_vs_ShScrambled	O35855	O35855	Bcat2	12036	-0.814487653	0.00093338	0.03410196
ShSirt1_vs_ShScrambled	Q8VBZ3	Q8VBZ3	Clptm1	56457	-0.850576773	0.00094294	0.03410196
ShSirt1_vs_ShScrambled	Q80UM7	Q80UM7	Mogs	57377	0.945079057	0.00094434	0.03410196
ShSirt1_vs_ShScrambled	P70699	P70699	Gaa	14387	-0.733341589	0.00097151	0.03480456
ShSirt1_vs_ShScrambled	O55098	O55098	Stk10	20868	2.153965359	0.00099129	0.03497142
ShSirt1_vs_ShScrambled	Q9CZC8	Q9CZC8	Scrn1	69938	-2.40152725	0.0010008	0.03497142
ShSirt1_vs_ShScrambled	P21956	P21956	Mfge8	17304	1.628919713	0.00100253	0.03497142
ShSirt1_vs_ShScrambled	P58802	P58802	Tbc1d10a	103724	3.781753605	0.00100715	0.03497142
ShSirt1_vs_ShScrambled	P42225	P42225	Stat1		-0.748878865	0.00104326	0.03571002
ShSirt1_vs_ShScrambled	Q91YX5	Q91YX5	Lpgat1	226856	-0.987909071	0.00104425	0.03571002
ShSirt1_vs_ShScrambled	Q3THW5	Q3THW5;P0	H2afv		0.727016149	0.00105251	0.03572198
ShSirt1_vs_ShScrambled	Q6P5F7	Q6P5F7	Ttyh3	78339	-0.772985996	0.00107883	0.03631227
ShSirt1_vs_ShScrambled	Q8C1E7	Q8C1E7	Tmem120a	215210	-1.329223432	0.00108599	0.03631227
ShSirt1_vs_ShScrambled	P97315	P97315	Csrp1	13007	1.140149843	0.00112818	0.03723377
ShSirt1_vs_ShScrambled	Q91VS7	Q91VS7	Mgst1	56615	-1.098886143	0.00113005	0.03723377
ShSirt1_vs_ShScrambled	Q3TIX9	Q3TIX9	Usp39	28035	1.121166257	0.0011476	0.03747893
ShSirt1_vs_ShScrambled	Q8K1E6	Q8K1E6	Alkbh3	69113	1.038302705	0.00115409	0.03747893
ShSirt1_vs_ShScrambled	Q3U962	Q3U962	Col5a2	12832	-1.335686589	0.00119192	0.03771608
ShSirt1_vs_ShScrambled	Q61037	Q61037	Tsc2		-1.345766524	0.00119417	0.03771608
ShSirt1_vs_ShScrambled	Q91YI0	Q91YI0	Asl	109900	0.888219697	0.00119522	0.03771608
ShSirt1_vs_ShScrambled	Q924K8	Q924K8	Mta3	116871	1.995802192	0.00119968	0.03771608
ShSirt1_vs_ShScrambled	Q99MR3	Q99MR3	Slc12a9	83704	-2.144861281	0.00120334	0.03771608
ShSirt1_vs_ShScrambled	Q05920	Q05920	Pc		-0.663359139	0.0012197	0.03771608
ShSirt1_vs_ShScrambled	P0DN34	P0DN34	P0DN34		-1.428817765	0.00122029	0.03771608
ShSirt1_vs_ShScrambled	Q923J1	Q923J1	Trpm7	58800	3.020446804	0.00125631	0.03771608
ShSirt1_vs_ShScrambled	Q8BV13	Q8BV13	Cops7b	26895	1.938522971	0.0012573	0.03771608
ShSirt1_vs_ShScrambled	P02469	P02469	Lamb1	16777	0.882586463	0.00125829	0.03771608
ShSirt1_vs_ShScrambled	P15306	P15306	Thbd	21824	1.380459028	0.00126779	0.03771608
ShSirt1_vs_ShScrambled	P54116	P54116	Stom	13830	-0.997253257	0.00127802	0.03771608
ShSirt1_vs_ShScrambled	P52196	P52196	Tst	22117	-0.833449489	0.00128637	0.03771608
ShSirt1_vs_ShScrambled	P49718	P49718	Mcm5		0.802888566	0.00128985	0.03771608
ShSirt1_vs_ShScrambled	Q9CZU3	Q9CZU3	Skiv2l2	72198	1.18005386	0.00130104	0.03771608
ShSirt1_vs_ShScrambled	Q9DCS9	Q9DCS9	Ndufb10	68342	-0.850780407	0.00130928	0.03771608
ShSirt1_vs_ShScrambled	O54782	O54782	Man2b2	17160	-0.719327458	0.00131143	0.03771608
ShSirt1_vs_ShScrambled	Q9JMF7	Q9JMF7	Dolpp1	57170	-2.942422873	0.00131179	0.03771608
ShSirt1_vs_ShScrambled	Q9CZN7	Q9CZN7	Shmt2	108037	0.835235804	0.00132196	0.03776784
ShSirt1_vs_ShScrambled	Q9CQX2	Q9CQX2	Cyb5b	66427	-1.162639999	0.00134001	0.03780675
ShSirt1_vs_ShScrambled	Q8CIF6	Q8CIF6	Sidt2	214597	-0.993845422	0.00135644	0.03780675
ShSirt1_vs_ShScrambled	Q9JK42	Q9JK42	Pdk2	18604	-1.911307734	0.00135645	0.03780675
ShSirt1_vs_ShScrambled	Q920I9	Q920I9	Wdr7	104082	-0.908835394	0.00135789	0.03780675
ShSirt1_vs_ShScrambled	Q60714	Q60714	Slc27a1	26457	-1.253718533	0.00137094	0.03780675
ShSirt1_vs_ShScrambled	Q9DBL9	Q9DBL9	Abhd5	67469	-0.864312397	0.00137357	0.03780675
ShSirt1_vs_ShScrambled	P16110	P16110	Lgals3		-0.705260016	0.00142124	0.03867467
ShSirt1_vs_ShScrambled	Q69ZN7	Q69ZN7	Myof	226101	-0.713454494	0.00142224	0.03867467
ShSirt1_vs_ShScrambled	P97311	P97311	Mcm6	17219	0.945184665	0.00144457	0.03877305
ShSirt1_vs_ShScrambled	Q9D9V3	Q9D9V3	Echdc1	52665	-1.052790459	0.00144768	0.03877305
ShSirt1_vs_ShScrambled	Q9D6J6	Q9D6J6	Ndufv2	72900	-0.697719631	0.00145163	0.03877305
ShSirt1_vs_ShScrambled	Q922E4	Q922E4	Pcyt2	68671	0.859908198	0.00147467	0.03915679
ShSirt1_vs_ShScrambled	Q99N84	Q99N84	Mrps18b	66973	-1.704148871	0.00149116	0.03932349
ShSirt1_vs_ShScrambled	Q8BYM8	Q8BYM8	Cars2	71941	-1.417659975	0.00151952	0.03932349
ShSirt1_vs_ShScrambled	P26231	P26231	Ctnna1	12385	-0.703616261	0.00152664	0.03932349
ShSirt1_vs_ShScrambled	Q9ET22	Q9ET22	Dpp7	83768	-0.906576541	0.00152703	0.03932349
ShSirt1_vs_ShScrambled	Q80UZ2	Q80UZ2	Sdad1	231452	1.328271261	0.00153086	0.03932349
ShSirt1_vs_ShScrambled	Q8BXA1	Q8BXA1	Golim4	73124	0.795466563	0.00154024	0.03932349
ShSirt1_vs_ShScrambled	Q8VC70	Q8VC70	Rbms2	56516	-0.714071375	0.00155311	0.03932349
ShSirt1_vs_ShScrambled	P97352	P97352	S100a13		-1.291104396	0.00155846	0.03932349
ShSirt1_vs_ShScrambled	Q6P8M1	Q6P8M1	Tatdn1	69694	1.07496579	0.00156598	0.03932349
ShSirt1_vs_ShScrambled	Q9D7N9	Q9D7N9	Apmap	71881	-0.812609828	0.00156806	0.03932349
ShSirt1_vs_ShScrambled	P49717	P49717	Mcm4	17217	0.928593197	0.00158772	0.03949864
ShSirt1_vs_ShScrambled	Q9R0B9	Q9R0B9	Plod2	26432	1.644898307	0.00159972	0.03949864

ShSirt1_vs_ShScrambled	P08775	P08775	Polr2a	20020	0.917934361	0.0016032	0.03949864
ShSirt1_vs_ShScrambled	C0HK80	C0HK80;C0H	C0HK80		-1.407604855	0.00161005	0.03949864
ShSirt1_vs_ShScrambled	B2RQC6	B2RQC6	Cad	69719	0.647681826	0.00161944	0.03951444
ShSirt1_vs_ShScrambled	P97449	P97449	Anpep	16790	-1.074543998	0.00165722	0.04021881
ShSirt1_vs_ShScrambled	P11672	P11672	Lcn2	16819	-3.616235508	0.00168128	0.04037711
ShSirt1_vs_ShScrambled	P61022	P61022	Chp1	56398	-1.068516871	0.00168163	0.04037711
ShSirt1_vs_ShScrambled	Q9WVL0	Q9WVL0	Gstz1	14874	-0.732999708	0.00169825	0.04056043
ShSirt1_vs_ShScrambled	Q8K353	Q8K353	Cystm1		-1.300967534	0.00171438	0.04059034
ShSirt1_vs_ShScrambled	O70503	O70503	Hsd17b12	56348	-0.712012849	0.00172425	0.04059034
ShSirt1_vs_ShScrambled	P27048	P27048;P631	Snrpb		0.787235934	0.00172648	0.04059034
ShSirt1_vs_ShScrambled	Q9Z329	Q9Z329	Itpr2	16439	1.316622831	0.00176713	0.04115057
ShSirt1_vs_ShScrambled	Q91YH5	Q91YH5	Atl3	109168	-0.748053646	0.00176854	0.04115057
ShSirt1_vs_ShScrambled	P56379	P56379	Mp68	70257	-2.811522227	0.0018037	0.04175331
ShSirt1_vs_ShScrambled	Q9D0T1	Q9D0T1	Nhp2l1	20826; 10	0.766266441	0.00181952	0.04190466
ShSirt1_vs_ShScrambled	Q64310	Q64310	Surf4	20932	-1.011181695	0.00184134	0.04198887
ShSirt1_vs_ShScrambled	O35126	O35126	Atn1	13498	0.90054188	0.00184178	0.04198887
ShSirt1_vs_ShScrambled	Q9DAW9	Q9DAW9	Cnn3	71994	-0.998998495	0.00185392	0.04199994
ShSirt1_vs_ShScrambled	Q61592	Q61592	Gas6	14456	-1.109734159	0.00186087	0.04199994
ShSirt1_vs_ShScrambled	Q3UUQ7	Q3UUQ7	Pgap1	241062	-1.717008701	0.00189941	0.04265094
ShSirt1_vs_ShScrambled	P43276	P43276	Hist1h1b	56702	0.832297422	0.00190862	0.04265094
ShSirt1_vs_ShScrambled	Q9EQW7	Q9EQW7	Kif13a		0.730415876	0.00193247	0.0429713
ShSirt1_vs_ShScrambled	Q9DCJ5	Q9DCJ5	Ndufa8	68375	-1.122525432	0.00198382	0.04389679
ShSirt1_vs_ShScrambled	Q9ERE7	Q9ERE7	Mesdc2	67943	0.798624414	0.00199883	0.04393004
ShSirt1_vs_ShScrambled	Q9DCM2	Q9DCM2	Gstk1	76263	-1.185804934	0.00200478	0.04393004
ShSirt1_vs_ShScrambled	Q8CJF7	Q8CJF7	Ahctf1	226747	1.405872621	0.00201863	0.04401984
ShSirt1_vs_ShScrambled	Q8BVW0	Q8BVW0	Ganc	76051	-0.803645428	0.00203752	0.04419268
ShSirt1_vs_ShScrambled	Q9CQF9	Q9CQF9	Pcyox1	66881	-0.766022488	0.00204614	0.04419268
ShSirt1_vs_ShScrambled	P08122	P08122	Col4a2	12827	-1.352416845	0.0021019	0.04518094
ShSirt1_vs_ShScrambled	P70335	P70335	Rock1	19877	-1.196910378	0.00212747	0.04542063
ShSirt1_vs_ShScrambled	Q8R180	Q8R180	Ero1l	50527	0.849490866	0.00214133	0.04542063
ShSirt1_vs_ShScrambled	Q91ZP3	Q91ZP3	Lpin1	14245	-0.938703337	0.00214906	0.04542063
ShSirt1_vs_ShScrambled	Q9JJE4	Q9JJE4	Paqr4	76498	-0.87714074	0.00216095	0.04542063
ShSirt1_vs_ShScrambled	Q6PAR5	Q6PAR5	Gapvd1	66691	0.778513287	0.00216337	0.04542063
ShSirt1_vs_ShScrambled	Q05816	Q05816	Fabp5	16592	-0.640813581	0.00218493	0.04554918
ShSirt1_vs_ShScrambled	Q61207	Q61207	Psap	19156	-1.214567376	0.00218967	0.04554918
ShSirt1_vs_ShScrambled	P63082	P63082	Atp6v0c	11984	-0.615874816	0.00224255	0.04643517
ShSirt1_vs_ShScrambled	Q9Z2I0	Q9Z2I0	Letm1	56384	-0.62495344	0.00225722	0.04652548
ShSirt1_vs_ShScrambled	Q9JII6	Q9JII6	Akr1a1	58810	-0.708851663	0.00227634	0.04670644
ShSirt1_vs_ShScrambled	Q9Z0G0	Q9Z0G0	Gipc1	67903	-0.646732729	0.00233674	0.04761728
ShSirt1_vs_ShScrambled	Q60716	Q60716	P4ha2	18452	1.168990554	0.00234183	0.04761728
ShSirt1_vs_ShScrambled	Q8VI75	Q8VI75	Ipo4	75751	0.611950295	0.00235535	0.04767743
ShSirt1_vs_ShScrambled	Q9CQC7	Q9CQC7	Ndufb4	68194; 10	-0.880027216	0.00238129	0.04798717
ShSirt1_vs_ShScrambled	Q64516	Q64516	Gk	14933	-0.937369874	0.00239742	0.04809756
ShSirt1_vs_ShScrambled	Q8BX70	Q8BX70	Vps13c	320528	-0.810929888	0.00242091	0.04835155
ShSirt1_vs_ShScrambled	Q08024	Q08024	Cbfb	12400	-0.787518373	0.00243638	0.04835155
ShSirt1_vs_ShScrambled	Q99M71	Q99M71	Epdr1	105298	-1.218627351	0.00244221	0.04835155
ShSirt1_vs_ShScrambled	Q60598	Q60598	Cttn	13043	-0.798097352	0.002471	0.04870777
ShSirt1_vs_ShScrambled	Q8R1F1	Q8R1F1	Fam129b	227737	0.623645242	0.0024823	0.04871793
ShSirt1_vs_ShScrambled	P26187	P26187	Mgmt	17314	-0.752922926	0.00252783	0.04883796
ShSirt1_vs_ShScrambled	Q99KX1	Q99KX1	Mlf2	30853	-0.746992611	0.00256737	0.04883796
ShSirt1_vs_ShScrambled	P97310	P97310	Mcm2	17216	0.96111792	0.00257106	0.04883796
ShSirt1_vs_ShScrambled	Q3UHC7	Q3UHC7	Dab2ip	69601	1.238659489	0.00257641	0.04883796
ShSirt1_vs_ShScrambled	Q3UMY5	Q3UMY5	Eml4	78798	0.867764849	0.00257994	0.04883796
ShSirt1_vs_ShScrambled	Q9EQK5	Q9EQK5	Mvp	78388	-0.688147866	0.00258048	0.04883796
ShSirt1_vs_ShScrambled	Q9CQ62	Q9CQ62	Decr1	67460	-0.610151563	0.00258707	0.04883796
ShSirt1_vs_ShScrambled	Q8BK67	Q8BK67	Rcc2	108911	0.649082988	0.00260282	0.04883796
ShSirt1_vs_ShScrambled	Q9JJZ4	Q9JJZ4	Ube2j1	56228	-0.608407891	0.00260438	0.04883796
ShSirt1_vs_ShScrambled	Q8CCF0	Q8CCF0	Prpf31	68988	1.508778787	0.00261796	0.04883796
ShSirt1_vs_ShScrambled	P18572	P18572	Bsg	12215	0.713608346	0.00262029	0.04883796
ShSirt1_vs_ShScrambled	Q9R1J0	Q9R1J0	Nsdhl	18194	-0.793835117	0.00262147	0.04883796
ShSirt1_vs_ShScrambled	P31786	P31786	Dbi	13167	-1.417315627	0.00262907	0.04883796
ShSirt1_vs_ShScrambled	Q9DBS2	Q9DBS2	Tprg1l	67808	-1.329020724	0.0026514	0.04905086
ShSirt1_vs_ShScrambled	Q8K1A5	Q8K1A5	Tmem41b	233724	-1.173775767	0.00266493	0.0491

ShSirt1_vs_ShScrambled	Q5U5M8	Q5U5M8	Bloc1s3	232946	-1.306421257	0.00267842	0.04914793
ShSirt1_vs_ShScrambled	Q9DBZ1	Q9DBZ1	Ikbip	67454	1.038166253	0.00269872	0.04919567
ShSirt1_vs_ShScrambled	P50114	P50114	S100b	20203	-2.194384414	0.00270282	0.04919567
ShSirt1_vs_ShScrambled	O70400	O70400	Pdlim1	54132	0.937674139	0.00272896	0.04947203
ShSirt1_vs_ShScrambled	P51881	P51881	Slc25a5	11740	-0.773341543	0.00275105	0.04954351
ShSirt1_vs_ShScrambled	Q9D1K7	Q9D1K7	Q9D1K7	67326	-1.149590604	0.00277328	0.04954351
ShSirt1_vs_ShScrambled	Q60715	Q60715	P4ha1	18451	0.587935	0.00277515	0.04954351
ShSirt1_vs_ShScrambled	Q5SWU9	Q5SWU9	Acaca	107476	-0.615607413	0.00277712	0.04954351
ShSirt1_vs_ShScrambled	P26638	P26638	Sars	20226	0.643795672	0.00278778	0.04954351
ShSirt1_vs_ShScrambled	P47934	P47934	Crat	12908	-0.62898423	0.0028277	0.04981955
ShSirt1_vs_ShScrambled	Q61699	Q61699	Hsph1	15505	0.831995217	0.00283034	0.04981955
ShSirt1_vs_ShScrambled	Q9D8B4	Q9D8B4	Ndufa11		-1.410681683	0.00284345	0.04981955
ShSirt1_vs_ShScrambled	Q99K51	Q99K51	Pls3	102866	-0.78466511	0.00284746	0.04981955
ShSirt1_vs_ShScrambled	A2AJL3	A2AJL3	Fggy	75578	0.806550261	0.00287328	0.04988957
ShSirt1_vs_ShScrambled	Q64FW2	Q64FW2	Retsat	67442	-0.758823964	0.00288333	0.04988957
ShSirt1_vs_ShScrambled	Q8VHK9	Q8VHK9	Dhx36	72162	0.563285905	0.00288672	0.04988957
ShSirt1_vs_ShScrambled	Q8VDP6	Q8VDP6	Cdipt	52858	-1.240800116	0.00289567	0.04988957
ShSirt1_vs_ShScrambled	P16015	P16015	Ca3	12350	-2.855581628	0.00291174	0.0498937
ShSirt1_vs_ShScrambled	Q91WG0	Q91WG0	Ces2c	234671	-2.511369934	0.0029257	0.0498937
ShSirt1_vs_ShScrambled	Q9ERN0	Q9ERN0	Scamp2	24044	-0.945489363	0.00293238	0.0498937
ShSirt1_vs_ShScrambled	Q8BGC4	Q8BGC4	Zadh2	225791	-1.08749217	0.00295433	0.0498937
ShSirt1_vs_ShScrambled	Q9QZA0	Q9QZA0	Ca5b	56078	-0.768532114	0.00296212	0.0498937
ShSirt1_vs_ShScrambled	Q9R111	Q9R111	Gda	14544	-0.753438367	0.00296783	0.0498937
ShSirt1_vs_ShScrambled	A3KGS3	A3KGS3	Ralgapa2	241694	-0.830694219	0.00298188	0.0498937
ShSirt1_vs_ShScrambled	Q8BMA6	Q8BMA6	Srp68	217337	0.713638562	0.00298434	0.0498937
ShSirt1_vs_ShScrambled	Q7TMM9	Q7TMM9	Tubb2a	22151	-1.5836667	0.00305767	0.05065592
ShSirt1_vs_ShScrambled	Q9WUU9	Q9WUU9	Mcm3ap	54387	2.202295198	0.00306152	0.05065592
ShSirt1_vs_ShScrambled	P29391	P29391;P499	Ftl1		1.231043275	0.00307595	0.05065592
ShSirt1_vs_ShScrambled	Q3UPH7	Q3UPH7	Arhgef40	268739	0.637288489	0.00307649	0.05065592
ShSirt1_vs_ShScrambled	Q9WUA3	Q9WUA3	Pfkp	56421	0.891773328	0.00308855	0.05065592
ShSirt1_vs_ShScrambled	P50518	P50518	Atp6v1e1	11973	-0.775226832	0.00310062	0.05065592
ShSirt1_vs_ShScrambled	Q9CPV4	Q9CPV4	Glod4	67201	0.6639266	0.00312083	0.05065592
ShSirt1_vs_ShScrambled	Q8VHR5	Q8VHR5	Gatad2b	229542	0.629123347	0.00312373	0.05065592
ShSirt1_vs_ShScrambled	P51807	P51807	Dynlt1	21648; 10	-0.796126375	0.00313374	0.05065592
ShSirt1_vs_ShScrambled	Q9ERS2	Q9ERS2	Ndufa13	67184	-0.727658089	0.00314215	0.05065592
ShSirt1_vs_ShScrambled	Q5U4D8	Q5U4D8	Slc5a6	330064	-1.39104272	0.00315829	0.05073494
ShSirt1_vs_ShScrambled	Q99P72	Q99P72	Rtn4	68585	-0.568793078	0.0031847	0.05081774
ShSirt1_vs_ShScrambled	P57716	P57716	Ncstn	59287	-1.023548272	0.00319491	0.05081774
ShSirt1_vs_ShScrambled	Q6PGB6	Q6PGB6	Naa50	72117	0.728789695	0.00320626	0.05081774
ShSirt1_vs_ShScrambled	Q3ULW8	Q3ULW8	Parp3	235587	-0.646664709	0.00320847	0.05081774
ShSirt1_vs_ShScrambled	Q6PA06	Q6PA06	Atl2	56298	-0.642616052	0.0032372	0.05101187
ShSirt1_vs_ShScrambled	P97820	P97820	Map4k4	26921	-0.660050305	0.00325467	0.05101187
ShSirt1_vs_ShScrambled	Q9ES97	Q9ES97	Rtn3	20168	-1.578553117	0.00326585	0.05101187
ShSirt1_vs_ShScrambled	Q61097	Q61097	Ksr1	16706	1.224661758	0.00326922	0.05101187
ShSirt1_vs_ShScrambled	Q921W4	Q921W4	Cryzl1	66609	1.217632178	0.00327724	0.05101187
ShSirt1_vs_ShScrambled	Q80X95	Q80X95;Q6N	Rraga		-0.718463765	0.00329458	0.05110558
ShSirt1_vs_ShScrambled	Q9WTQ5	Q9WTQ5	Akap12	83397	-1.136928581	0.00335461	0.05185867
ShSirt1_vs_ShScrambled	Q80VA0	Q80VA0	Galnt7	108150	1.578326541	0.00339378	0.05212258
ShSirt1_vs_ShScrambled	Q9WU81	Q9WU81	Slc37a2	56857	-0.944217155	0.00339493	0.05212258
ShSirt1_vs_ShScrambled	Q64735	Q64735	Cr1l	12946	0.851313134	0.00341368	0.05212258
ShSirt1_vs_ShScrambled	Q8C3R1	Q8C3R1	Brat1	231841	3.063903642	0.00341787	0.05212258
ShSirt1_vs_ShScrambled	Q8BYK4	Q8BYK4	Rdh12	77974	1.389889197	0.00345565	0.05223297
ShSirt1_vs_ShScrambled	P98192	P98192	Gnpat	14712	-0.725845377	0.00346189	0.05223297
ShSirt1_vs_ShScrambled	Q8BP40	Q8BP40	Acp6	66659	0.861933693	0.003486	0.05223297
ShSirt1_vs_ShScrambled	P61804	P61804	Dad1	13135	-2.226162043	0.00348864	0.05223297
ShSirt1_vs_ShScrambled	Q922J9	Q922J9	Far1	67420	-0.647443694	0.00349194	0.05223297
ShSirt1_vs_ShScrambled	Q9Z110	Q9Z110	Aldh18a1	56454	0.559255089	0.00349454	0.05223297
ShSirt1_vs_ShScrambled	Q9CZ44	Q9CZ44	Nsfl1c	386649	0.587323588	0.00350778	0.05225786
ShSirt1_vs_ShScrambled	P00405	P00405	Mtco2	17709	-0.860998969	0.00353672	0.05251569
ShSirt1_vs_ShScrambled	P25206	P25206	Mcm3	17215	0.825199764	0.00358861	0.05302407
ShSirt1_vs_ShScrambled	Q9R0Y5	Q9R0Y5	Ak1	11636	0.659621847	0.00359445	0.05302407
ShSirt1_vs_ShScrambled	P56395	P56395	Cyb5a	109672	-1.062967286	0.00365754	0.05377895
ShSirt1_vs_ShScrambled	Q3TSG4	Q3TSG4	Alkbh5	268420	0.538750708	0.00369576	0.05416453

ShSirt1_vs_ShScrambled	Q9DBJ1	Q9DBJ1	Pgam1	18648	0.594764151	0.0037204	0.05431084
ShSirt1_vs_ShScrambled	P59708	P59708	Sf3b6	66055	1.130882033	0.00374948	0.05431084
ShSirt1_vs_ShScrambled	Q9R1Q9	Q9R1Q9	Atp6ap1	54411	-0.662349824	0.00375914	0.05431084
ShSirt1_vs_ShScrambled	P07091	P07091	S100a4	20198	-0.927814918	0.00376518	0.05431084
ShSirt1_vs_ShScrambled	Q80Y84	Q80Y84	Kdm5b	75605	-5.200924902	0.0037659	0.05431084
ShSirt1_vs_ShScrambled	Q9CZU6	Q9CZU6	Cs	12974	-0.613465551	0.00381918	0.0547025
ShSirt1_vs_ShScrambled	P23780	P23780	Glb1	12091	-0.584961839	0.00382444	0.0547025
ShSirt1_vs_ShScrambled	Q9QZC8	Q9QZC8	Abhd1	57742	-1.083543034	0.00382942	0.0547025
ShSirt1_vs_ShScrambled	Q64261	Q64261	Cdk6	12571	-0.867530438	0.00391081	0.055689
ShSirt1_vs_ShScrambled	Q3ULD5	Q3ULD5	Mccc2	78038	-0.560222968	0.00393381	0.05584033
ShSirt1_vs_ShScrambled	Q8CHT0	Q8CHT0	Aldh4a1	212647	-0.676693298	0.00404106	0.05718288
ShSirt1_vs_ShScrambled	Q9JJW0	Q9JJW0	Pxmp4	59038	-0.813870898	0.00405598	0.0572147
ShSirt1_vs_ShScrambled	P70441	P70441	Slc9a3r1	26941	-1.745211106	0.00410769	0.05776357
ShSirt1_vs_ShScrambled	Q91W69	Q91W69	Epn3	71889	0.758613931	0.00413241	0.05793076
ShSirt1_vs_ShScrambled	Q9WV92	Q9WV92	Epb41l3	13823	0.616709966	0.00417251	0.05814049
ShSirt1_vs_ShScrambled	P81122	P81122	Irs2	384783	2.344420228	0.00417313	0.05814049
ShSirt1_vs_ShScrambled	Q3THG9	Q3THG9	Aarsd1	69684	0.854991358	0.00421521	0.05844942
ShSirt1_vs_ShScrambled	Q8BXC6	Q8BXC6	Commd2	52245	-0.852279107	0.0042212	0.05844942
ShSirt1_vs_ShScrambled	P50171	P50171	Hsd17b8	14979	0.575730519	0.00427983	0.05882448
ShSirt1_vs_ShScrambled	Q9QXD1	Q9QXD1	Acox2	93732	0.777819029	0.00428227	0.05882448
ShSirt1_vs_ShScrambled	Q9D2R0	Q9D2R0	Aacs	78894	-0.916869178	0.00430516	0.05882448
ShSirt1_vs_ShScrambled	Q80U78	Q80U78	Pum1	80912	-0.914182056	0.00431932	0.05882448
ShSirt1_vs_ShScrambled	Q9ERD7	Q9ERD7	Tubb3	22152	-1.881014804	0.0043203	0.05882448
ShSirt1_vs_ShScrambled	Q8BH69	Q8BH69	Sephs1	109079	1.20366131	0.00433825	0.05882448
ShSirt1_vs_ShScrambled	O08784	O08784	Tcof1	21453	0.792332756	0.00433956	0.05882448
ShSirt1_vs_ShScrambled	P09528	P09528	Fth1	14319	1.062862719	0.00435986	0.05882448
ShSirt1_vs_ShScrambled	P49615	P49615	Cdk5	12568	0.903056926	0.00436557	0.05882448
ShSirt1_vs_ShScrambled	Q9QZQ8	Q9QZQ8	H2afy	26914	0.523898602	0.00440858	0.05893922
ShSirt1_vs_ShScrambled	P01027	P01027	C3	12266	-0.835633919	0.00440977	0.05893922
ShSirt1_vs_ShScrambled	Q99JY8	Q99JY8	Ppap2b	67916	-1.078976813	0.00441543	0.05893922
ShSirt1_vs_ShScrambled	Q6P1B1	Q6P1B1	Xpnpep1	170750	0.569981223	0.00442632	0.05893922
ShSirt1_vs_ShScrambled	Q9QZ08	Q9QZ08	Nagk	56174	0.769688096	0.00451726	0.05997333
ShSirt1_vs_ShScrambled	O35459	O35459	Ech1	51798	0.874327662	0.00453558	0.06003989
ShSirt1_vs_ShScrambled	Q8R2Y0	Q8R2Y0	Abhd6	66082	1.218572135	0.00457683	0.06040874
ShSirt1_vs_ShScrambled	Q61335	Q61335	Bcap31	27061	-0.665952567	0.00460629	0.06062042
ShSirt1_vs_ShScrambled	P46735	P46735	Myo1b	17912	0.504341882	0.00464204	0.06087214
ShSirt1_vs_ShScrambled	Q8BU14	Q8BU14	Sec62	69276	-0.93864538	0.00465239	0.06087214
ShSirt1_vs_ShScrambled	P28653	P28653	Bgn	12111	-0.786855551	0.00470256	0.06113623
ShSirt1_vs_ShScrambled	P12265	P12265	Gusb	110006	-0.699346809	0.00471059	0.06113623
ShSirt1_vs_ShScrambled	Q9ET54	Q9ET54	Palld	72333	-0.713388934	0.00471574	0.06113623
ShSirt1_vs_ShScrambled	P48410	P48410	Abcd1	11666	-0.857062688	0.00474518	0.06113623
ShSirt1_vs_ShScrambled	Q8K2B0	Q8K2B0	Leprel4	66180	-0.787013802	0.00475275	0.06113623
ShSirt1_vs_ShScrambled	Q99P87	Q99P87	Retn	57264	-1.139363911	0.00475384	0.06113623
ShSirt1_vs_ShScrambled	Q9QY36	Q9QY36	Naa10	56292	0.729441347	0.00477116	0.06118466
ShSirt1_vs_ShScrambled	P11881	P11881	Itpr1	16438	-0.748836688	0.00482237	0.0614984
ShSirt1_vs_ShScrambled	Q8BGP6	Q8BGP6	Slc25a40	319653	0.828968659	0.00482287	0.0614984
ShSirt1_vs_ShScrambled	Q80TR8	Q80TR8	Vprbp	321006	1.766947301	0.00489466	0.06223802
ShSirt1_vs_ShScrambled	P29416	P29416	Hexa	15211	-0.516614867	0.0049758	0.06302784
ShSirt1_vs_ShScrambled	O35551	O35551	Rabep1	54189	0.872810171	0.0049847	0.06302784
ShSirt1_vs_ShScrambled	Q8K0U4	Q8K0U4	Hspa12a	73442	-1.511457409	0.00500811	0.06308095
ShSirt1_vs_ShScrambled	O35127	O35127	Grcc10	14790	-4.515263632	0.00501685	0.06308095
ShSirt1_vs_ShScrambled	Q9CZL5	Q9CZL5	Pcbd2	72562	1.138052305	0.00505993	0.06344596
ShSirt1_vs_ShScrambled	Q91VH6	Q91VH6	Memo1	76890	-0.910982709	0.0050839	0.06348174
ShSirt1_vs_ShScrambled	Q8BX09	Q8BX09	Rbbp5	213464	3.23780046	0.00509092	0.06348174
ShSirt1_vs_ShScrambled	Q9QZK2	Q9QZK2	Bcar3	29815	-1.573722515	0.00518362	0.06421138
ShSirt1_vs_ShScrambled	Q9JJF3	Q9JJF3	No66	71952	0.860414618	0.00519008	0.06421138
ShSirt1_vs_ShScrambled	P50516	P50516	Atp6v1a	11964	-0.512343362	0.0051921	0.06421138
ShSirt1_vs_ShScrambled	Q9R1T4	Q9R1T4	Sept6	56526	-0.625378426	0.00523599	0.06457719
ShSirt1_vs_ShScrambled	Q8K2I1	Q8K2I1	Fntb	110606	-1.178462228	0.00530339	0.0652302
ShSirt1_vs_ShScrambled	Q9WU40	Q9WU40	Lemd3	380664	1.38309061	0.00533434	0.06543266
ShSirt1_vs_ShScrambled	Q99L43	Q99L43	Cds2	110911	0.652318849	0.00542641	0.06638162
ShSirt1_vs_ShScrambled	Q9JMH9	Q9JMH9	Myo18a	360013	-1.366904461	0.00548612	0.0668602
ShSirt1_vs_ShScrambled	P97742	P97742	Cpt1a	12894	-1.008258916	0.00549516	0.0668602

ShSirt1_vs_ShScrambled	P48771	P48771	Cox7a2	12866	-1.939619851	0.00558936	0.06782353
ShSirt1_vs_ShScrambled	Q69ZR2	Q69ZR2	Hectd1		-1.447284768	0.00561318	0.06789544
ShSirt1_vs_ShScrambled	P39749	P39749	Fen1		0.71027758	0.00563787	0.06789544
ShSirt1_vs_ShScrambled	Q5DU37	Q5DU37	Zfyve26	211978	-0.626896365	0.00564041	0.06789544
ShSirt1_vs_ShScrambled	Q8K4Z3	Q8K4Z3	Apoa1bp	246703	0.57589937	0.00568537	0.0682547
ShSirt1_vs_ShScrambled	Q8BP92	Q8BP92	Rcn2	26611	-1.165162323	0.00571326	0.06840763
ShSirt1_vs_ShScrambled	Q8CHU3	Q8CHU3	Epn2	13855	1.388566766	0.00576088	0.06876141
ShSirt1_vs_ShScrambled	Q9QX60	Q9QX60	Dguok	27369	0.520150334	0.00577328	0.06876141
ShSirt1_vs_ShScrambled	P04117	P04117	Fabp4	11770	-0.662182608	0.00582181	0.06915693
ShSirt1_vs_ShScrambled	Q91V76	Q91V76	Q91V76	70984	-0.591167443	0.00585962	0.06925913
ShSirt1_vs_ShScrambled	Q8BXV2	Q8BXV2	Bri3bp	76809	0.565087915	0.00587804	0.06925913
ShSirt1_vs_ShScrambled	Q8K2T1	Q8K2T1	Nmral1	67824	0.525426779	0.00591383	0.06925913
ShSirt1_vs_ShScrambled	Q62425	Q62425	Ndufa4	17992	-0.579370334	0.00592388	0.06925913
ShSirt1_vs_ShScrambled	Q9WTU6	Q9WTU6	Mapk9	26420	0.81413158	0.00593421	0.06925913
ShSirt1_vs_ShScrambled	Q02819	Q02819	Nucb1	18220	-0.732363539	0.00593647	0.06925913
ShSirt1_vs_ShScrambled	Q61937	Q61937	Npm1	18148	0.485170403	0.00594668	0.06925913
ShSirt1_vs_ShScrambled	Q9CQT2	Q9CQT2	Rbm7	67010	0.906853791	0.00595316	0.06925913
ShSirt1_vs_ShScrambled	Q9Z0E6	Q9Z0E6	Gbp2	14469	-2.284871838	0.0059722	0.06930209
ShSirt1_vs_ShScrambled	P84102	P84102	Serf2	378702	1.633532671	0.00604273	0.06986634
ShSirt1_vs_ShScrambled	P51432	P51432	Plcb3	18797	0.541305343	0.00605828	0.06986634
ShSirt1_vs_ShScrambled	Q9QXX4	Q9QXX4	Slc25a13	50799	0.717541031	0.00606726	0.06986634
ShSirt1_vs_ShScrambled	P43024	P43024	Cox6a1	12861	-0.988767318	0.00610408	0.07011149
ShSirt1_vs_ShScrambled	Q8BK03	Q8BK03	Fam73b	108958	-0.921632135	0.00612557	0.0701785
ShSirt1_vs_ShScrambled	O08810	O08810	Eftud2	20624	0.489576638	0.00614549	0.0701785
ShSirt1_vs_ShScrambled	P03911	P03911	Mtnd4	17719	-1.634383545	0.00615655	0.0701785
ShSirt1_vs_ShScrambled	Q64337	Q64337	Sqstm1	18412	0.823757699	0.00619865	0.07048033
ShSirt1_vs_ShScrambled	Q3U3W5	Q3U3W5	Prmt9	102182	-1.241563798	0.00623826	0.07068427
ShSirt1_vs_ShScrambled	Q4VA55	Q4VA55	Mum1l1	245631	-0.975249438	0.0062479	0.07068427
ShSirt1_vs_ShScrambled	P20152	P20152	Vim	22352	-0.566230887	0.00629719	0.07106384
ShSirt1_vs_ShScrambled	Q8C650	Q8C650	Sept10	103080	-1.45441263	0.00635078	0.07148985
ShSirt1_vs_ShScrambled	P11438	P11438	Lamp1	16783	-0.942076576	0.00649541	0.07290451
ShSirt1_vs_ShScrambled	Q8VD04	Q8VD04	Gripap1	54645	0.599772997	0.00652402	0.07290451
ShSirt1_vs_ShScrambled	Q9D7A8	Q9D7A8	Armc1	74252	-0.56197621	0.00653256	0.07290451
ShSirt1_vs_ShScrambled	B1AVD1	B1AVD1	Xpnpep2	170745	-1.634162007	0.00654106	0.07290451
ShSirt1_vs_ShScrambled	Q8VEE4	Q8VEE4	Rpa1	68275	0.951630135	0.00657572	0.07311032
ShSirt1_vs_ShScrambled	Q80X82	Q80X82	Sympk		0.835045823	0.00660067	0.07320744
ShSirt1_vs_ShScrambled	P17751	P17751	Tpi1	21991	0.566223456	0.00663058	0.07335892
ShSirt1_vs_ShScrambled	Q60847	Q60847	Col12a1	12816	-1.645768214	0.00665217	0.07341781
ShSirt1_vs_ShScrambled	Q00612	Q00612	G6pdx	14381	-0.478750227	0.00671415	0.07373672
ShSirt1_vs_ShScrambled	Q07113	Q07113	Igf2r	16004	-0.52716376	0.00677221	0.07373672
ShSirt1_vs_ShScrambled	O08601	O08601	Mttp	17777	0.600200935	0.00678115	0.07373672
ShSirt1_vs_ShScrambled	Q6PIP5	Q6PIP5	Nudcd1	67429	1.324380873	0.00678323	0.07373672
ShSirt1_vs_ShScrambled	Q6ZQ89	Q6ZQ89	March6	223455	1.63006057	0.00679689	0.07373672
ShSirt1_vs_ShScrambled	P50285	P50285	Fmo1	14261	-0.739496768	0.00680571	0.07373672
ShSirt1_vs_ShScrambled	Q8C033	Q8C033	Arhgef10	234094	1.333324624	0.00680634	0.07373672
ShSirt1_vs_ShScrambled	Q9EQP2	Q9EQP2	Ehd4	98878	-0.617562921	0.00683258	0.07373672
ShSirt1_vs_ShScrambled	Q9ER60	Q9ER60	Scn4a	110880	-0.882234788	0.00684318	0.07373672
ShSirt1_vs_ShScrambled	Q8CD26	Q8CD26	Slc35e1	270066	-2.842214252	0.00684441	0.07373672
ShSirt1_vs_ShScrambled	Q9WV54	Q9WV54	Asah1	11886	-0.700760722	0.00689471	0.07410174
ShSirt1_vs_ShScrambled	Q9D1G2	Q9D1G2	Pmvk	68603	-0.644055367	0.00693057	0.07431024
ShSirt1_vs_ShScrambled	Q80Y14	Q80Y14	Glrx5	73046	-0.644920464	0.00695945	0.07444301
ShSirt1_vs_ShScrambled	Q6R891	Q6R891	Ppp1r9b	217124	0.664045916	0.00698825	0.07457438
ShSirt1_vs_ShScrambled	Q8C0E3	Q8C0E3	Trim47	217333	1.137179996	0.00702109	0.07474807
ShSirt1_vs_ShScrambled	Q61738	Q61738	Itga7		1.945923045	0.00707106	0.07510296
ShSirt1_vs_ShScrambled	Q99ME2	Q99ME2	Wdr6	83669	-0.870167572	0.00711835	0.07542778
ShSirt1_vs_ShScrambled	Q6P5D8	Q6P5D8	Smchd1	74355	0.629859644	0.00713983	0.07543199
ShSirt1_vs_ShScrambled	Q9JLJ5	Q9JLJ5	Elovl1	54325	-1.059396284	0.00715287	0.07543199
ShSirt1_vs_ShScrambled	Q8CFI0	Q8CFI0	Nedd4l		-1.16452161	0.00716888	0.07543199
ShSirt1_vs_ShScrambled	Q8R2R3	Q8R2R3	Aagab	66939	-1.08506624	0.00719239	0.07550336
ShSirt1_vs_ShScrambled	Q8K4P0	Q8K4P0	Wdr33	74320	0.761043522	0.0072376	0.075768
ShSirt1_vs_ShScrambled	P06797	P06797	Ctsl	13039	2.432840931	0.00725117	0.075768
ShSirt1_vs_ShScrambled	O70318	O70318	Epb41l2	13822	0.516002941	0.00728315	0.07586463
ShSirt1_vs_ShScrambled	Q61425	Q61425	Hadh	15107	-0.478049469	0.00729403	0.07586463

ShSirt1_vs_ShScrambled	Q8CFQ3	Q8CFQ3	Aqr	11834	0.647209627	0.00731755	0.07591626
ShSirt1_vs_ShScrambled	Q8CIG9	Q8CIG9	Fbxl8	50788	-1.554849211	0.00733263	0.07591626
ShSirt1_vs_ShScrambled	P01897	P01897	H2.L		-0.661224832	0.00736036	0.07602903
ShSirt1_vs_ShScrambled	Q9Z1Q9	Q9Z1Q9	Vars	22321	0.498972068	0.00740921	0.07604895
ShSirt1_vs_ShScrambled	Q02248	Q02248	Ctnnb1	12387	-0.984185117	0.00741871	0.07604895
ShSirt1_vs_ShScrambled	Q61576	Q61576	Fkbp10	14230	0.506614531	0.00741914	0.07604895
ShSirt1_vs_ShScrambled	Q8BTM8	Q8BTM8	Flna	192176	-0.535551168	0.00744622	0.07604895
ShSirt1_vs_ShScrambled	Q8C0M9	Q8C0M9	Asrgl1	66514	1.089308225	0.00745801	0.07604895
ShSirt1_vs_ShScrambled	Q920L1	Q920L1	Fads1	76267	0.505551134	0.00750035	0.07604895
ShSirt1_vs_ShScrambled	Q9WV55	Q9WV55	Vapa	30960	0.546550938	0.00750369	0.07604895
ShSirt1_vs_ShScrambled	Q9DCS2	Q9DCS2	Q9DCS2	68347	0.855095146	0.00751396	0.07604895
ShSirt1_vs_ShScrambled	Q9D2Y4	Q9D2Y4	Mlkl	74568	-1.262637845	0.00752341	0.07604895
ShSirt1_vs_ShScrambled	P08074	P08074	Cbr2	12409	0.57014142	0.00754626	0.07604895
ShSirt1_vs_ShScrambled	Q811J3	Q811J3	Ireb2	64602	-1.20319302	0.00756401	0.07604895
ShSirt1_vs_ShScrambled	Q8K4L0	Q8K4L0	Ddx54	71990	-1.695763596	0.00756637	0.07604895
ShSirt1_vs_ShScrambled	P10852	P10852	Slc3a2	17254	-0.589901636	0.00758131	0.07604895
ShSirt1_vs_ShScrambled	Q3UJB9	Q3UJB9	Edc4	234699	-0.494293228	0.00760868	0.0761543
ShSirt1_vs_ShScrambled	P48758	P48758	Cbr1	12408	-0.478501661	0.00763364	0.07623509
ShSirt1_vs_ShScrambled	Q8BWU5	Q8BWU5	Osgep	66246	0.664824283	0.00781265	0.07785055
ShSirt1_vs_ShScrambled	A2A4P0	A2A4P0	Dhx8	217207	1.28801114	0.00783491	0.07790042
ShSirt1_vs_ShScrambled	Q9R0Q9	Q9R0Q9	Mpdu1		-0.990512445	0.00785971	0.07797525
ShSirt1_vs_ShScrambled	P40630	P40630	Tfam	21780	1.031015893	0.00792571	0.07845757
ShSirt1_vs_ShScrambled	Q9WU56	Q9WU56	Pus1	56361	0.491971892	0.0079721	0.07856647
ShSirt1_vs_ShScrambled	Q9JLM8	Q9JLM8	Dclk1	13175	-0.858568314	0.00798788	0.07856647
ShSirt1_vs_ShScrambled	Q61490	Q61490	Alcam	11658	-1.211596336	0.00799966	0.07856647
ShSirt1_vs_ShScrambled	P53690	P53690	Mmp14	17387	-0.788595715	0.00800633	0.07856647
ShSirt1_vs_ShScrambled	P31428	P31428	Dpep1	13479	0.643599923	0.008063	0.07884197
ShSirt1_vs_ShScrambled	Q78T54	Q78T54	Vma21	67048	-1.106190446	0.00809022	0.07884197
ShSirt1_vs_ShScrambled	Q3U6U5	Q3U6U5	Gtpbp6	107999	0.700954096	0.00809054	0.07884197
ShSirt1_vs_ShScrambled	Q60870	Q60870	Reep5		-2.20288324	0.00810427	0.07884197
ShSirt1_vs_ShScrambled	Q61655	Q61655	Ddx19a	13680	-0.522165905	0.00817829	0.07930764
ShSirt1_vs_ShScrambled	Q01853	Q01853	Vcp	269523	-0.447172756	0.00820222	0.07930764
ShSirt1_vs_ShScrambled	Q9EQG7	Q9EQG7	Enpp5	83965	-0.738560852	0.00820526	0.07930764
ShSirt1_vs_ShScrambled	Q9CQ89	Q9CQ89	Cuta	67675	-0.878338092	0.00822241	0.07930764
ShSirt1_vs_ShScrambled	Q91ZW3	Q91ZW3	Smarca5	93762	0.745840551	0.00825538	0.07937054
ShSirt1_vs_ShScrambled	P43274	P43274	Hist1h1e	50709	0.500113124	0.0082641	0.07937054
ShSirt1_vs_ShScrambled	Q9D710	Q9D710	Tmx2	66958	-1.062669633	0.0083288	0.07954117
ShSirt1_vs_ShScrambled	Q9CYI0	Q9CYI0	Q9CYI0	70591	1.731433045	0.00833055	0.07954117
ShSirt1_vs_ShScrambled	P62774	P62774	Mtpn	14489	-0.950948984	0.00833473	0.07954117
ShSirt1_vs_ShScrambled	Q99LJ0	Q99LJ0	Cttnbp2nl	80281	2.666164961	0.00837271	0.07973509
ShSirt1_vs_ShScrambled	Q9JM62	Q9JM62	Reep6	70335	-1.382591516	0.00839093	0.07974034
ShSirt1_vs_ShScrambled	Q8BKX1	Q8BKX1	Baiap2	108100	0.710109795	0.00842565	0.07990209
ShSirt1_vs_ShScrambled	Q9JLI8	Q9JLI8	Sart3	53890	0.753467536	0.00852393	0.08066465
ShSirt1_vs_ShScrambled	P19783	P19783	Cox4i1	12857	-0.663667424	0.00854235	0.0806698
ShSirt1_vs_ShScrambled	P70347	P70347	Tank	21353	-1.586227555	0.00856929	0.08075528
ShSirt1_vs_ShScrambled	Q8K215	Q8K215	Lyrm4	380840	-1.553643839	0.00860011	0.0808681
ShSirt1_vs_ShScrambled	Q91X78	Q91X78	Erlin1	226144	1.011544319	0.00862775	0.0808681
ShSirt1_vs_ShScrambled	Q80UM3	Q80UM3	Naa15		0.698426244	0.00864944	0.0808681
ShSirt1_vs_ShScrambled	Q99LT0	Q99LT0	Dpy30	66310	-1.586494541	0.00865292	0.0808681
ShSirt1_vs_ShScrambled	P97363	P97363	Sptlc2	20773	-0.616653442	0.00869526	0.08097919
ShSirt1_vs_ShScrambled	Q61753	Q61753	Phgdh	236539	-0.560277271	0.00870069	0.08097919
ShSirt1_vs_ShScrambled	Q99KF0	Q99KF0	Card14	170720	2.741093803	0.00876679	0.08142653
ShSirt1_vs_ShScrambled	Q6PDG5	Q6PDG5	Smarcc2	68094	0.691044037	0.00879885	0.08153402
ShSirt1_vs_ShScrambled	Q8CGF7	Q8CGF7	Tcerg1	56070	0.544092659	0.00881449	0.08153402
ShSirt1_vs_ShScrambled	Q61578	Q61578	Fdxr	14149	0.626992397	0.00885957	0.08178344
ShSirt1_vs_ShScrambled	P39098	P39098	Man1a2	17156	1.119963327	0.0088892	0.08188949
ShSirt1_vs_ShScrambled	P83870	P83870	Phf5a	68479	2.811419992	0.00891429	0.08195341
ShSirt1_vs_ShScrambled	Q8VBZ0	Q8VBZ0	Dhrsx		-0.88678371	0.00898757	0.08231628
ShSirt1_vs_ShScrambled	Q9CZE3	Q9CZE3	Rab32	67844	-0.881169545	0.00900797	0.08231628
ShSirt1_vs_ShScrambled	P11152	P11152	Lpl	16956	-0.538981147	0.00900847	0.08231628
ShSirt1_vs_ShScrambled	Q6NVE8	Q6NVE8	Wdr44	72404	3.375821302	0.00902893	0.08233659
ShSirt1_vs_ShScrambled	Q8R4U7	Q8R4U7	Luzp1	269593	-0.875852801	0.00909584	0.08277946
ShSirt1_vs_ShScrambled	Q8VHX6	Q8VHX6	Flnc	68794	-0.553667972	0.00913698	0.08298659

ShSirt1_vs_ShScrambled	P13011	P13011	Scd2	20250	-1.01183978	0.0091607	0.08302296
ShSirt1_vs_ShScrambled	Q5NCI0	Q5NCI0	Urgcp	72046	-3.348514271	0.00917777	0.08302296
ShSirt1_vs_ShScrambled	Q5SRX1	Q5SRX1	Tom1l2	216810	-1.025845964	0.00924723	0.08348399
ShSirt1_vs_ShScrambled	Q9R233	Q9R233	Tapbp	21356	-0.469789858	0.0092932	0.08358396
ShSirt1_vs_ShScrambled	Q8K1R3	Q8K1R3	Pnpt1	71701	0.526996603	0.00929534	0.08358396
ShSirt1_vs_ShScrambled	Q3U0B3	Q3U0B3	Dhrs11	192970	-0.823484881	0.00934152	0.08383223
ShSirt1_vs_ShScrambled	P14115	P14115	Rpl27a	26451	0.602323503	0.00943684	0.08445826
ShSirt1_vs_ShScrambled	P15864	P15864	Hist1h1c	50708	0.701110211	0.0094487	0.08445826
ShSirt1_vs_ShScrambled	Q91WM2	Q91WM2	Cecr5	214932	0.48514448	0.00948301	0.08457754
ShSirt1_vs_ShScrambled	Q6NZB0	Q6NZB0	Dnajc8	68598	0.930341522	0.00949952	0.08457754
ShSirt1_vs_ShScrambled	P10605	P10605	Ctsb	13030	-0.550266587	0.00952603	0.08464664
ShSirt1_vs_ShScrambled	P31324	P31324	Prkar2b	19088	-0.474803225	0.00958521	0.08500516
ShSirt1_vs_ShScrambled	Q9CQW1	Q9CQW1	Ykt6	56418	-0.459444942	0.00963659	0.08529328
ShSirt1_vs_ShScrambled	P63328	P63328	Ppp3ca	19055	-0.445708772	0.00970255	0.08564829
ShSirt1_vs_ShScrambled	Q9D7V9	Q9D7V9	Naaa	67111	-1.923428065	0.00971802	0.08564829
ShSirt1_vs_ShScrambled	Q8BVQ5	Q8BVQ5	Ppme1	72590	0.738410881	0.00977259	0.08564829
ShSirt1_vs_ShScrambled	P50247	P50247	Ahcy	11615; 26	0.459848981	0.00977434	0.08564829
ShSirt1_vs_ShScrambled	P10518	P10518	Alad	17025	-0.535379291	0.00980321	0.08564829
ShSirt1_vs_ShScrambled	Q9WTM5	Q9WTM5	Ruvbl2	20174	0.493615897	0.00984048	0.08564829
ShSirt1_vs_ShScrambled	P68372	P68372;Q9D	Tubb4b		-0.43558766	0.00984365	0.08564829
ShSirt1_vs_ShScrambled	P55012	P55012	Slc12a2	20496	-1.558213749	0.00984759	0.08564829
ShSirt1_vs_ShScrambled	Q91WM3	Q91WM3	Rrp9	27966	0.755691167	0.00986108	0.08564829
ShSirt1_vs_ShScrambled	Q8CGK3	Q8CGK3	Lonp1	74142	0.497400101	0.00986644	0.08564829
ShSirt1_vs_ShScrambled	Q91YP2	Q91YP2	Nln	75805	-0.534531067	0.00992354	0.08582288
ShSirt1_vs_ShScrambled	Q78ZA7	Q78ZA7	Nap1l4	17955	-0.581873698	0.00992458	0.08582288
ShSirt1_vs_ShScrambled	Q60710	Q60710	Samhd1	56045	-0.533075225	0.00995345	0.08590797
ShSirt1_vs_ShScrambled	Q8R317	Q8R317	Ubqln1	56085	-0.95966033	0.00999214	0.08595893
ShSirt1_vs_ShScrambled	Q91YN1	Q91YN1	Fam118a	73225	3.20909457	0.00999744	0.08595893
ShSirt1_vs_ShScrambled	P07742	P07742	Rrm1	20133	0.984146035	0.01004976	0.08624451
ShSirt1_vs_ShScrambled	Q3UIU2	Q3UIU2	Ndufb6	230075	-1.44146326	0.01007727	0.08630002
ShSirt1_vs_ShScrambled	Q6P9R1	Q6P9R1	Ddx51	69663	0.726024082	0.01009446	0.08630002
ShSirt1_vs_ShScrambled	Q61712	Q61712	Dnajc1	13418	-1.021778439	0.01016743	0.08664219
ShSirt1_vs_ShScrambled	P97379	P97379	G3bp2	23881	-0.73329878	0.0101767	0.08664219
ShSirt1_vs_ShScrambled	Q8CDM1	Q8CDM1	Atad2	70472	1.373965593	0.01019207	0.08664219
ShSirt1_vs_ShScrambled	P59999	P59999	Arpc4	68089	-0.527685451	0.01027626	0.08705158
ShSirt1_vs_ShScrambled	Q9WVS8	Q9WVS8	Mapk7	23939	3.16084226	0.01030651	0.08705158
ShSirt1_vs_ShScrambled	Q99LB7	Q99LB7	Sardh	192166	0.744309032	0.01031567	0.08705158
ShSirt1_vs_ShScrambled	Q9CQM9	Q9CQM9	Glrx3	30926	-0.677350237	0.01031737	0.08705158
ShSirt1_vs_ShScrambled	Q99L13	Q99L13	Hibadh	58875	-0.587469705	0.01034801	0.08714725
ShSirt1_vs_ShScrambled	P14733	P14733	Lmnb1	16906	0.682890243	0.01040044	0.08742571
ShSirt1_vs_ShScrambled	Q9QXG4	Q9QXG4	Acss2	60525	-0.636466313	0.01048134	0.08794198
ShSirt1_vs_ShScrambled	Q9EP69	Q9EP69	Sacm1l	83493	-0.538376869	0.01051196	0.08796007
ShSirt1_vs_ShScrambled	Q9Z148	Q9Z148	Ehmt2	110147	1.211754453	0.01053897	0.08796007
ShSirt1_vs_ShScrambled	Q91WK2	Q91WK2	Eif3h	68135	0.458957779	0.01054196	0.08796007
ShSirt1_vs_ShScrambled	Q8R127	Q8R127	Sccpdh	109232	-0.549526063	0.01058275	0.08812811
ShSirt1_vs_ShScrambled	Q9D1J1	Q9D1J1	Necap2	66147	-0.623324772	0.01060293	0.08812811
ShSirt1_vs_ShScrambled	P54751	P54751	St3gal1	20442	-1.504643153	0.01063723	0.08812811
ShSirt1_vs_ShScrambled	Q6NVF9	Q6NVF9	Cpsf6	432508	0.567210738	0.01064876	0.08812811
ShSirt1_vs_ShScrambled	Q9JJU8	Q9JJU8	Sh3bgrl	56726	-0.464620352	0.01065971	0.08812811
ShSirt1_vs_ShScrambled	P27661	P27661	H2afx	15270	0.774471334	0.01077873	0.08894912
ShSirt1_vs_ShScrambled	O70274	O70274	Ptp4a2	19244	2.953856547	0.01083078	0.08921559
ShSirt1_vs_ShScrambled	Q8BH86	Q8BH86	Q8BH86	217830	-0.534177214	0.0108643	0.0893136
ShSirt1_vs_ShScrambled	Q9D379	Q9D379	Ephx1	13849	-0.450969013	0.01088304	0.0893136
ShSirt1_vs_ShScrambled	P48024	P48024;Q9C	Eif1		-0.635137056	0.01090204	0.0893136
ShSirt1_vs_ShScrambled	P62996	P62996	Tra2b	20462	0.576082515	0.01092641	0.08931954
ShSirt1_vs_ShScrambled	Q64435	Q64435	Ugt1a6	94284	-0.548218188	0.01097661	0.08931954
ShSirt1_vs_ShScrambled	Q99MR8	Q99MR8	Mccc1	72039	-0.626500276	0.01098178	0.08931954
ShSirt1_vs_ShScrambled	Q91Z53	Q91Z53	Grhpr	76238	0.497664602	0.01100809	0.08931954
ShSirt1_vs_ShScrambled	O89050	O89050	Mkln1	27418	-0.545091108	0.01101863	0.08931954
ShSirt1_vs_ShScrambled	Q8BGX0	Q8BGX0	Trim23		-1.250326881	0.01102148	0.08931954
ShSirt1_vs_ShScrambled	Q569Z6	Q569Z6	Thrap3	230753	0.559395778	0.01104744	0.08936941
ShSirt1_vs_ShScrambled	P28474	P28474	Adh5	11532	0.469515525	0.01113868	0.08994634
ShSirt1_vs_ShScrambled	Q6ZQ73	Q6ZQ73	Cand2	67088	0.726360185	0.01116137	0.08996861

ShSirt1_vs_ShScrambled	Q9JHS3	Q9JHS3	Lamtor2	83409	-0.82740045	0.01134856	0.09131445
ShSirt1_vs_ShScrambled	Q9D1L0	Q9D1L0	Chchd2	14004	0.468326968	0.0115356	0.09265431
ShSirt1_vs_ShScrambled	Q99LJ6	Q99LJ6	Gpx7	67305	0.511434954	0.011583	0.09286971
ShSirt1_vs_ShScrambled	Q5SF07	Q5SF07	Igf2bp2	319765	0.433652766	0.01168543	0.09328161
ShSirt1_vs_ShScrambled	Q3UPF5	Q3UPF5	Zc3hav1	78781	0.448221058	0.0116867	0.09328161
ShSirt1_vs_ShScrambled	Q6ZWM4	Q6ZWM4	Lsm8	76522	0.614284451	0.01169637	0.09328161
ShSirt1_vs_ShScrambled	Q6ZQ58	Q6ZQ58	Larp1	73158	0.482469428	0.01173623	0.09343443
ShSirt1_vs_ShScrambled	Q8BT60	Q8BT60	Cpne3	70568	-0.640522973	0.01177661	0.09352224
ShSirt1_vs_ShScrambled	O70423	O70423	Aoc3	11754	0.648425384	0.01182111	0.09352224
ShSirt1_vs_ShScrambled	Q9JMG7	Q9JMG7	Hdgfrp3	29877	-0.966594993	0.01182258	0.09352224
ShSirt1_vs_ShScrambled	P70202	P70202	Lxn	17035	1.553674792	0.01185955	0.09352224
ShSirt1_vs_ShScrambled	P80317	P80317	Cct6a	12466	0.455544417	0.01186273	0.09352224
ShSirt1_vs_ShScrambled	B9EJ86	B9EJ86	Osbpl8	237542	0.631195008	0.01188461	0.09352224
ShSirt1_vs_ShScrambled	P40336	P40336	Vps26a	30930	-0.479291665	0.01190629	0.09352224
ShSirt1_vs_ShScrambled	Q9D824	Q9D824	Fip1l1	66899	0.510290622	0.011913	0.09352224
ShSirt1_vs_ShScrambled	Q9Z1N5	Q9Z1N5	Ddx39b	53817	0.473615772	0.01195887	0.0936137
ShSirt1_vs_ShScrambled	O88351	O88351	Ikbkb	16150	-0.610114923	0.01196613	0.0936137
ShSirt1_vs_ShScrambled	Q3UQ84	Q3UQ84	Tars2	71807	0.527795342	0.01203655	0.09393901
ShSirt1_vs_ShScrambled	Q9DBL1	Q9DBL1	Acadsb	66885	-0.758893751	0.01204933	0.09393901
ShSirt1_vs_ShScrambled	Q91WG8	Q91WG8	Gne	50798	-0.481065826	0.0121761	0.09420021
ShSirt1_vs_ShScrambled	Q91VD1	Q91VD1	Lgals12	56072	-0.518404188	0.01218069	0.09420021
ShSirt1_vs_ShScrambled	Q9D706	Q9D706	Rpap3	71919	0.739309836	0.01218511	0.09420021
ShSirt1_vs_ShScrambled	Q9CXD6	Q9CXD6	Mcur1	76137	-0.83602749	0.0121992	0.09420021
ShSirt1_vs_ShScrambled	Q8R0F3	Q8R0F3	Sumf1	58911	-0.545996354	0.01220786	0.09420021
ShSirt1_vs_ShScrambled	Q80SY5	Q80SY5	Prpf38b	66921	0.624835697	0.01220805	0.09420021
ShSirt1_vs_ShScrambled	Q9DBZ5	Q9DBZ5	Eif3k	73830	0.531474623	0.01223293	0.09423115
ShSirt1_vs_ShScrambled	Q9WVK4	Q9WVK4	Ehd1	13660	-0.5129224	0.01225418	0.09423399
ShSirt1_vs_ShScrambled	P59108	P59108	Cpne2	234577	-0.981819032	0.01238982	0.09505929
ShSirt1_vs_ShScrambled	Q8VDT9	Q8VDT9	Mrpl50	28028	-0.562736428	0.01243337	0.09505929
ShSirt1_vs_ShScrambled	Q9QYB5	Q9QYB5	Add3	27360	0.547389814	0.01244506	0.09505929
ShSirt1_vs_ShScrambled	Q8R570	Q8R570	Snap47	67826	-0.968077303	0.01244573	0.09505929
ShSirt1_vs_ShScrambled	Q60770	Q60770	Stxbp3	20912	0.67481116	0.0125065	0.09511165
ShSirt1_vs_ShScrambled	Q4LDD4	Q4LDD4	Arap1	69710	-0.471822303	0.01251162	0.09511165
ShSirt1_vs_ShScrambled	Q8VBT9	Q8VBT9	Aspscr1	68938	0.424180657	0.0125158	0.09511165
ShSirt1_vs_ShScrambled	Q8R035	Q8R035	Ict1	68572	0.945564423	0.01255955	0.09518736
ShSirt1_vs_ShScrambled	Q3TNA1	Q3TNA1	Xylb	102448	-0.721182245	0.01258364	0.09518736
ShSirt1_vs_ShScrambled	Q9D3B1	Q9D3B1	Hacd2	70757	-0.565828267	0.01258902	0.09518736
ShSirt1_vs_ShScrambled	Q920Q6	Q920Q6	Msi2	76626	-1.157094048	0.01262733	0.09527589
ShSirt1_vs_ShScrambled	Q8BYJ6	Q8BYJ6	Tbc1d4	210789	0.47795291	0.01264295	0.09527589
ShSirt1_vs_ShScrambled	Q8C4Y3	Q8C4Y3	Nelfb	58202	1.112535521	0.01269978	0.09554465
ShSirt1_vs_ShScrambled	Q80YD1	Q80YD1	Supv3l1	338359	0.625157681	0.01276001	0.09578516
ShSirt1_vs_ShScrambled	Q8BYK6	Q8BYK6	Ythdf3	229096	-0.784750254	0.01277418	0.09578516
ShSirt1_vs_ShScrambled	Q3UGP9	Q3UGP9	Lrrc58	320184	-0.720127324	0.01281086	0.0959009
ShSirt1_vs_ShScrambled	Q91X88	Q91X88	Pomgnt1	68273	2.007557217	0.01284424	0.09592843
ShSirt1_vs_ShScrambled	Q9QXY9	Q9QXY9	Pex3	56535	-1.334787441	0.01285704	0.09592843
ShSirt1_vs_ShScrambled	O54974	O54974	Lgals7		2.826418745	0.01289701	0.09606781
ShSirt1_vs_ShScrambled	P54822	P54822	Adsl	11564	0.422883822	0.01295034	0.09630619
ShSirt1_vs_ShScrambled	Q9D0R9	Q9D0R9	Wdr89	72338	0.892832087	0.0130113	0.09660033
ShSirt1_vs_ShScrambled	Q6PDL0	Q6PDL0	Dync1li2	234663	-0.708679928	0.01308925	0.0969152
ShSirt1_vs_ShScrambled	P45952	P45952	Acadm	11364	-0.519246927	0.01311489	0.0969152
ShSirt1_vs_ShScrambled	Q8R3Q2	Q8R3Q2	Ppp6r2		2.40746224	0.01311812	0.0969152
ShSirt1_vs_ShScrambled	P52503	P52503	Ndufs6	407785	-0.798017978	0.01323047	0.0975855
ShSirt1_vs_ShScrambled	Q8K212	Q8K212	Pacs1	107975	1.343481935	0.01329689	0.09791545
ShSirt1_vs_ShScrambled	Q3TL26	Q3TL26	Tfb2m	15278	-1.324684656	0.01334945	0.09802717
ShSirt1_vs_ShScrambled	Q8CGN5	Q8CGN5	Plin1	103968	-0.671019848	0.0133555	0.09802717
ShSirt1_vs_ShScrambled	Q9JKB3	Q9JKB3	Ybx3	56449	0.803722378	0.01365207	0.10001679
ShSirt1_vs_ShScrambled	Q9CQJ8	Q9CQJ8	Ndufb9	66218	-0.578607242	0.0136872	0.10001679
ShSirt1_vs_ShScrambled	Q9CXV1	Q9CXV1	Sdhd	66925	-1.488285706	0.01369304	0.10001679
ShSirt1_vs_ShScrambled	Q91UZ5	Q91UZ5	Impa2	114663	0.562351584	0.01374665	0.10024618
ShSirt1_vs_ShScrambled	Q9ESP1	Q9ESP1	Sdf2l1	64136	-0.964980542	0.01379645	0.10033794
ShSirt1_vs_ShScrambled	Q6NS46	Q6NS46	Pdcd11	18572	0.491065049	0.01380369	0.10033794
ShSirt1_vs_ShScrambled	Q8C7R4	Q8C7R4	Uba6	231380	0.462342834	0.01382609	0.10033918
ShSirt1_vs_ShScrambled	Q9JK81	Q9JK81	Myg1	60315	0.504289941	0.01400132	0.10144775

ShSirt1_vs_ShScrambled	Q8K3W0	Q8K3W0	Bre	107976	2.278108503	0.01409305	0.10194873
ShSirt1_vs_ShScrambled	Q9D0K1	Q9D0K1	Pex13	72129	-0.551927391	0.01419909	0.10248304
ShSirt1_vs_ShScrambled	Q3TCN2	Q3TCN2	Plbd2	71772	-0.540119584	0.01421231	0.10248304
ShSirt1_vs_ShScrambled	Q8VCR7	Q8VCR7	Abhd14b		-0.942566169	0.01425141	0.10260103
ShSirt1_vs_ShScrambled	Q60864	Q60864	Stip1	20867	0.53109439	0.01434457	0.10310732
ShSirt1_vs_ShScrambled	Q7TMR0	Q7TMR0	Prcp	72461	-0.474903642	0.01440388	0.10336904
ShSirt1_vs_ShScrambled	Q8CJ53	Q8CJ53	Trip10	106628	1.131628603	0.01454221	0.10390616
ShSirt1_vs_ShScrambled	Q9DBA9	Q9DBA9	Gtf2h1	14884	0.93576191	0.01456238	0.10390616
ShSirt1_vs_ShScrambled	P59017	P59017	Bcl2l13	94044	-0.659960055	0.01457678	0.10390616
ShSirt1_vs_ShScrambled	Q8CIB5	Q8CIB5	Fermt2	218952	0.441821193	0.01459538	0.10390616
ShSirt1_vs_ShScrambled	P53798	P53798	Fdft1	14137	-2.656624873	0.01459643	0.10390616
ShSirt1_vs_ShScrambled	Q9EQS3	Q9EQS3	Mycbp	56309	-3.062163135	0.01461684	0.10390616
ShSirt1_vs_ShScrambled	Q80X41	Q80X41	Vrk1	22367	1.824528655	0.0146443	0.10393769
ShSirt1_vs_ShScrambled	Q8BH79	Q8BH79	Ano10	102566	-0.57179465	0.01472058	0.10431503
ShSirt1_vs_ShScrambled	Q91WS0	Q91WS0	Cisd1	52637	-0.413309259	0.01475234	0.10437626
ShSirt1_vs_ShScrambled	Q9QX47	Q9QX47	Son	20658	0.449178513	0.01481662	0.10451549
ShSirt1_vs_ShScrambled	Q9ESD7	Q9ESD7	Dysf	26903	-0.485357906	0.01481832	0.10451549
ShSirt1_vs_ShScrambled	Q3TJZ6	Q3TJZ6	Fam98a	72722	-0.536674904	0.01499355	0.10546322
ShSirt1_vs_ShScrambled	Q8VD62	Q8VD62	Bles03	107242	0.889335339	0.01499942	0.10546322
ShSirt1_vs_ShScrambled	Q6VN19	Q6VN19	Ranbp10	74334	-0.904405332	0.01506183	0.10564376
ShSirt1_vs_ShScrambled	Q78PG9	Q78PG9	Ccdc25	67179	0.853688546	0.01507191	0.10564376
ShSirt1_vs_ShScrambled	Q8K021	Q8K021	Scamp1	107767	-0.80821096	0.01523681	0.10657515
ShSirt1_vs_ShScrambled	P32020	P32020	Scp2	20280	-0.719890125	0.01526229	0.10657515
ShSirt1_vs_ShScrambled	Q8VCI5	Q8VCI5	Pex19	19298	-0.807299519	0.01529208	0.10657515
ShSirt1_vs_ShScrambled	Q3TXT3	Q3TXT3	Inip	66209	-2.594706597	0.01529922	0.10657515
ShSirt1_vs_ShScrambled	Q9R062	Q9R062	Gyg1	27357	-1.167382848	0.01542672	0.1071396
ShSirt1_vs_ShScrambled	P17047	P17047	Lamp2	16784	-1.063915627	0.01543588	0.1071396
ShSirt1_vs_ShScrambled	Q99LC3	Q99LC3	Ndufa10	67273	-0.467352528	0.01546613	0.1071396
ShSirt1_vs_ShScrambled	P16675	P16675	Ctsa	19025	-0.957409759	0.01547519	0.1071396
ShSirt1_vs_ShScrambled	P35290	P35290	Rab24	19336	0.842414592	0.01561122	0.10791582
ShSirt1_vs_ShScrambled	Q8CI71	Q8CI71	Ccdc132	73288	-0.551564693	0.01568585	0.10826592
ShSirt1_vs_ShScrambled	Q8BFP9	Q8BFP9	Pdk1	228026	0.693623551	0.01571466	0.10829918
ShSirt1_vs_ShScrambled	Q99MS7	Q99MS7	Ehbp1l1	114601	0.754720734	0.01574169	0.1083201
ShSirt1_vs_ShScrambled	Q3UV70	Q3UV70	Pdp1	381511	-0.712059357	0.01584876	0.10889084
ShSirt1_vs_ShScrambled	Q9JIY5	Q9JIY5	Htra2	64704	0.42136192	0.01588794	0.10899415
ShSirt1_vs_ShScrambled	Q80ZK0	Q80ZK0	Mrps10		1.15736841	0.01597531	0.10934946
ShSirt1_vs_ShScrambled	P54310	P54310	Lipe	16890	-0.404577322	0.01600033	0.10934946
ShSirt1_vs_ShScrambled	P39053	P39053	Dnm1	13429	-0.453976591	0.0160124	0.10934946
ShSirt1_vs_ShScrambled	Q2TBE6	Q2TBE6	Pi4k2a	84095	-0.515950673	0.01609081	0.10970379
ShSirt1_vs_ShScrambled	Q9JIF3	Q9JIF3	Slc2a8	56017	-2.528066648	0.0161129	0.10970379
ShSirt1_vs_ShScrambled	P97384	P97384	Anxa11	11744	0.529773934	0.01614633	0.10976587
ShSirt1_vs_ShScrambled	Q8C7E9	Q8C7E9	Cstf2t	83410	0.617084355	0.01629596	0.11041153
ShSirt1_vs_ShScrambled	Q8CIV2	Q8CIV2	Tmem259	216157	1.773522987	0.0163037	0.11041153
ShSirt1_vs_ShScrambled	P11930	P11930	Nudt19	110959	-1.05923517	0.01631469	0.11041153
ShSirt1_vs_ShScrambled	Q9DAT5	Q9DAT5	Trmu	72026	0.623749416	0.01634426	0.11044609
ShSirt1_vs_ShScrambled	Q62313	Q62313;Q62	Tgoln1		-0.525896648	0.01650403	0.11115722
ShSirt1_vs_ShScrambled	Q8K1N2	Q8K1N2	Phldb2	208177	-2.495157447	0.01653847	0.11115722
ShSirt1_vs_ShScrambled	Q3UMB9	Q3UMB9	Kiaa1033	319277	-0.797441946	0.01654185	0.11115722
ShSirt1_vs_ShScrambled	Q9WVD5	Q9WVD5	Slc25a15	18408	-0.974046717	0.016548	0.11115722
ShSirt1_vs_ShScrambled	Q3UVY5	Q3UVY5	Pcnxl4	67708	2.488784002	0.01662281	0.11149383
ShSirt1_vs_ShScrambled	Q8BNE1	Q8BNE1	Tcaf1	77574	-0.67057094	0.01672532	0.11201499
ShSirt1_vs_ShScrambled	Q9JLQ0	Q9JLQ0	Cd2ap	12488	0.700617697	0.01681449	0.11203449
ShSirt1_vs_ShScrambled	Q9JK23	Q9JK23	Psmg1	56088	0.51544217	0.01682773	0.11203449
ShSirt1_vs_ShScrambled	P08249	P08249	Mdh2	17448	-0.43048153	0.01685881	0.11203449
ShSirt1_vs_ShScrambled	O70481	O70481	Ubr1	22222	0.880124714	0.01687592	0.11203449
ShSirt1_vs_ShScrambled	Q8BMB3	Q8BMB3	Eif4e2	26987	0.655903771	0.01692762	0.11203449
ShSirt1_vs_ShScrambled	Q3UCQ1	Q3UCQ1	Foxk2	68837	2.465333808	0.01693863	0.11203449
ShSirt1_vs_ShScrambled	P24547	P24547	Impdh2	23918	0.446176518	0.01694126	0.11203449
ShSirt1_vs_ShScrambled	Q9D0K2	Q9D0K2	Oxct1	67041	-0.434908718	0.01694659	0.11203449
ShSirt1_vs_ShScrambled	P58043	P58043	Sesn2	230784	-1.154686786	0.01695161	0.11203449
ShSirt1_vs_ShScrambled	P41233	P41233	Abca1	11303	-1.376706937	0.01701117	0.11226376
ShSirt1_vs_ShScrambled	Q9DBP5	Q9DBP5	Cmpk1	66588	-0.419201264	0.01708881	0.11261154
ShSirt1_vs_ShScrambled	A2BE28	A2BE28	Las1l	76130	-0.674684118	0.01732106	0.1139756

ShSirt1_vs_ShScrambled	Q9R1S8	Q9R1S8	Capn7	12339	2.434299332	0.01737039	0.11413386
ShSirt1_vs_ShScrambled	Q920A5	Q920A5	Scpep1	74617	-0.589582995	0.01744549	0.11446068
ShSirt1_vs_ShScrambled	O35945	O35945	Aldh1a7	26358	-0.447942241	0.01751053	0.11472065
ShSirt1_vs_ShScrambled	Q9CYL5	Q9CYL5	Glipr2	384009	0.49795608	0.01756569	0.11491528
ShSirt1_vs_ShScrambled	Q8K221	Q8K221	Arfip2	76932	0.745726716	0.01764582	0.11515849
ShSirt1_vs_ShScrambled	P16460	P16460	Ass1	11898	-0.46630688	0.01765389	0.11515849
ShSirt1_vs_ShScrambled	Q69Z37	Q69Z37	Samd9l		-0.993874095	0.01768989	0.11522676
ShSirt1_vs_ShScrambled	Q9CQC6	Q9CQC6	Bzw1	66882	-0.686936595	0.01772419	0.11528382
ShSirt1_vs_ShScrambled	Q8CJ61	Q8CJ61	Cmtm4	97487	-1.119266834	0.01781567	0.11559397
ShSirt1_vs_ShScrambled	Q8R0G7	Q8R0G7	Spns1	73658	-0.418761587	0.01782308	0.11559397
ShSirt1_vs_ShScrambled	Q9D8S9	Q9D8S9	Bola1	69168	0.51519084	0.01793191	0.11613294
ShSirt1_vs_ShScrambled	Q922P9	Q922P9	Glyr1	74022	0.519813221	0.01802532	0.11656935
ShSirt1_vs_ShScrambled	Q8R3Q6	Q8R3Q6	Ccdc58	381045	1.62988852	0.01805095	0.11656935
ShSirt1_vs_ShScrambled	P70227	P70227	Itpr3	16440	-0.448335342	0.01807889	0.11658301
ShSirt1_vs_ShScrambled	Q91VM9	Q91VM9	Ppa2	74776	-0.585716385	0.01823794	0.11744091
ShSirt1_vs_ShScrambled	Q61171	Q61171	Prdx2	21672	-0.511987981	0.01827889	0.1175369
ShSirt1_vs_ShScrambled	Q6PD19	Q6PD19	Q6PD19	71617	0.740663374	0.01836254	0.11790281
ShSirt1_vs_ShScrambled	Q923D4	Q923D4	Sf3b5	66125	-1.332384682	0.01841305	0.11790281
ShSirt1_vs_ShScrambled	Q9CQ39	Q9CQ39	Med21	108098	-2.363764172	0.01841415	0.11790281
ShSirt1_vs_ShScrambled	Q60855	Q60855	Ripk1	19766	-0.752648822	0.01844591	0.11793884
ShSirt1_vs_ShScrambled	Q8BRT1	Q8BRT1	Clasp2	76499	-1.624260255	0.01856461	0.11809199
ShSirt1_vs_ShScrambled	Q60648	Q60648	Gm2a	14667	-1.530274179	0.01856545	0.11809199
ShSirt1_vs_ShScrambled	Q8K1X1	Q8K1X1	Wdr11	207425	0.524536168	0.01857669	0.11809199
ShSirt1_vs_ShScrambled	P14142	P14142	Slc2a4	20528	0.625291965	0.01861834	0.11809199
ShSirt1_vs_ShScrambled	Q91VC3	Q91VC3	Eif4a3	192170	0.412527604	0.01862213	0.11809199
ShSirt1_vs_ShScrambled	Q8VI36	Q8VI36	Pxn	19303	0.482445041	0.01862683	0.11809199
ShSirt1_vs_ShScrambled	Q9WVE8	Q9WVE8	Pacsin2	23970	0.416094509	0.01874583	0.11867979
ShSirt1_vs_ShScrambled	Q9QXS1	Q9QXS1	Plec	18810	-0.445306287	0.0187939	0.11870548
ShSirt1_vs_ShScrambled	Q9DB29	Q9DB29	Iah1	67732	-0.493236753	0.01885249	0.11870548
ShSirt1_vs_ShScrambled	Q8K273	Q8K273	Mmgt1	236792	-0.938650528	0.01885407	0.11870548
ShSirt1_vs_ShScrambled	Q8CCJ3	Q8CCJ3	Ufl1	67490	0.535345126	0.01888469	0.11870548
ShSirt1_vs_ShScrambled	O89032	O89032	Sh3pxd2a	14218	-0.509615387	0.01888745	0.11870548
ShSirt1_vs_ShScrambled	Q9ER00	Q9ER00	Stx12	100226	-0.435769592	0.01891042	0.11870548
ShSirt1_vs_ShScrambled	Q9ESW4	Q9ESW4	Agk	69923	0.537838651	0.01893397	0.11870548
ShSirt1_vs_ShScrambled	Q8R2U2	Q8R2U2	BC027231	212547	-1.123201833	0.0189897	0.11875629
ShSirt1_vs_ShScrambled	P11276	P11276	Fn1	14268	0.402761358	0.01899469	0.11875629
ShSirt1_vs_ShScrambled	Q8BFY9	Q8BFY9	Tnpo1	238799	0.377703924	0.01904735	0.11881287
ShSirt1_vs_ShScrambled	Q9CR67	Q9CR67	Tmem33	67878	-0.59910632	0.01905638	0.11881287
ShSirt1_vs_ShScrambled	Q8BMK1	Q8BMK1	Mettl2	52686	1.471544505	0.01910277	0.11893778
ShSirt1_vs_ShScrambled	Q6P5F9	Q6P5F9	Xpo1	103573	0.420996545	0.01917992	0.11925369
ShSirt1_vs_ShScrambled	A2RTL5	A2RTL5	Rsrc2	208606	0.501895991	0.01926356	0.11960896
ShSirt1_vs_ShScrambled	Q9CRC8	Q9CRC8	Lrrc40	67144	0.478119193	0.01935207	0.11999344
ShSirt1_vs_ShScrambled	P35601	P35601	Rfc1	19687	0.518793562	0.01940137	0.12007697
ShSirt1_vs_ShScrambled	Q9EPE9	Q9EPE9	Atp13a1	170759	-0.479117808	0.01941874	0.12007697
ShSirt1_vs_ShScrambled	Q11011	Q11011	Npepps	19155	0.391953693	0.01947353	0.12017392
ShSirt1_vs_ShScrambled	P42232	P42232	Stat5b	20851	3.148392327	0.01948766	0.12017392
ShSirt1_vs_ShScrambled	P58468	P58468	Fam207a	108707	0.612954783	0.019522	0.12022143
ShSirt1_vs_ShScrambled	P62307	P62307	Snrpf	69878	0.61669363	0.01957662	0.12023881
ShSirt1_vs_ShScrambled	Q9JKZ2	Q9JKZ2	Slc5a3	53881	-0.709853851	0.01960288	0.12023881
ShSirt1_vs_ShScrambled	Q9D4H8	Q9D4H8	Cul2	71745	0.426351641	0.01960473	0.12023881
ShSirt1_vs_ShScrambled	Q6A065	Q6A065	Cep170	545389	0.706116964	0.01976403	0.12101364
ShSirt1_vs_ShScrambled	P17665	P17665	Cox7c	12867	-1.213556705	0.01978468	0.12101364
ShSirt1_vs_ShScrambled	P58404	P58404	Strn4	97387	0.618560475	0.01989343	0.12136567
ShSirt1_vs_ShScrambled	Q9ERI6	Q9ERI6	Rdh14	105014	-0.558338086	0.01989601	0.12136567
ShSirt1_vs_ShScrambled	Q9D0M3	Q9D0M3	Cyc1	66445	0.741522325	0.0199761	0.12168976
ShSirt1_vs_ShScrambled	Q00519	Q00519	Xdh	22436	-0.375235887	0.02000501	0.12170163
ShSirt1_vs_ShScrambled	Q9DC40	Q9DC40	Telo2	71718	3.682538476	0.02009748	0.12174451
ShSirt1_vs_ShScrambled	P56375	P56375	Acyp2	75572	-0.622156784	0.02010238	0.12174451
ShSirt1_vs_ShScrambled	Q9DCL4	Q9DCL4	Mettl15	76894	0.75545771	0.02013757	0.12174451
ShSirt1_vs_ShScrambled	Q62203	Q62203	Sf3a2		0.728134969	0.02018727	0.12174451
ShSirt1_vs_ShScrambled	Q6PCN7	Q6PCN7	Hltf	20585	1.039158387	0.02019648	0.12174451
ShSirt1_vs_ShScrambled	Q9CPQ1	Q9CPQ1	Cox6c	12864	-0.636658265	0.02023034	0.12174451
ShSirt1_vs_ShScrambled	Q91WD5	Q91WD5	Ndufs2	226646	-0.617137894	0.02023299	0.12174451

ShSirt1_vs_ShScrambled	Q9CSN1	Q9CSN1	Snw1	66354	0.588581202	0.02027201	0.12174451
ShSirt1_vs_ShScrambled	Q6A0A9	Q6A0A9	FAM120A	218236	-0.415188588	0.02028162	0.12174451
ShSirt1_vs_ShScrambled	Q6P069	Q6P069	Sri	109552	0.761587058	0.02029321	0.12174451
ShSirt1_vs_ShScrambled	Q8K3X4	Q8K3X4	Irf2bpl	238330	1.187008429	0.02030873	0.12174451
ShSirt1_vs_ShScrambled	P07356	P07356	Anxa2	12306	-0.354237118	0.02061599	0.12321081
ShSirt1_vs_ShScrambled	Q6PHU5	Q6PHU5	Sort1	20661	-1.380730487	0.0206214	0.12321081
ShSirt1_vs_ShScrambled	Q9R112	Q9R112	Sqrdl	59010	-0.371635515	0.02063916	0.12321081
ShSirt1_vs_ShScrambled	Q9CRD2	Q9CRD2	Emc2	66736	-0.598532167	0.02066388	0.12321081
ShSirt1_vs_ShScrambled	Q61235	Q61235	Sntb2	20650	-0.465903327	0.02068981	0.12321081
ShSirt1_vs_ShScrambled	Q91V92	Q91V92	Acly	104112	-0.386511987	0.02071946	0.12322483
ShSirt1_vs_ShScrambled	P62317	P62317	Snrpd2	107686	0.533174221	0.02079939	0.12346196
ShSirt1_vs_ShScrambled	Q9DB16	Q9DB16	Cab39l	69008	2.959444008	0.02081403	0.12346196
ShSirt1_vs_ShScrambled	Q8BH04	Q8BH04	Pck2	74551	0.614444776	0.02089973	0.12380759
ShSirt1_vs_ShScrambled	Q9DB77	Q9DB77	Uqcrc2	67003	-0.427209294	0.02107887	0.12470516
ShSirt1_vs_ShScrambled	Q80VD1	Q80VD1	Fam98b	68215	0.543016512	0.02121507	0.12534664
ShSirt1_vs_ShScrambled	Q9QZL0	Q9QZL0	Ripk3	56532	0.974487778	0.02133097	0.12569219
ShSirt1_vs_ShScrambled	P03991	P03991;P144	H2.K1		-0.441763655	0.0214019	0.12569219
ShSirt1_vs_ShScrambled	P61327	P61327;Q9C	Magoh		0.682523081	0.02140319	0.12569219
ShSirt1_vs_ShScrambled	Q9CQB2	Q9CQB2	Fam195a	68241	-0.701151187	0.0214284	0.12569219
ShSirt1_vs_ShScrambled	P28741	P28741	Kif3a	16568	0.583892434	0.02144747	0.12569219
ShSirt1_vs_ShScrambled	Q56A10	Q56A10	Znf608	269023	2.187611841	0.02146256	0.12569219
ShSirt1_vs_ShScrambled	Q8BZM1	Q8BZM1	Glmn	170823	0.696473335	0.02146847	0.12569219
ShSirt1_vs_ShScrambled	Q3UVG3	Q3UVG3	Fam91a1	210998	0.586060136	0.02150298	0.12573116
ShSirt1_vs_ShScrambled	Q3UHX0	Q3UHX0	Nol8	70930	0.398308894	0.02158019	0.12583655
ShSirt1_vs_ShScrambled	Q91VR7	Q91VR7	Map1lc3a	66734	1.453863181	0.02162282	0.12583655
ShSirt1_vs_ShScrambled	Q9CWH5	Q9CWH5	Trmt11	73681	1.447799628	0.02164583	0.12583655
ShSirt1_vs_ShScrambled	O08800	O08800	Serpinb8	20725	-1.081908266	0.02167484	0.12583655
ShSirt1_vs_ShScrambled	Q63918	Q63918	Sdpr	20324	-0.428297349	0.02175351	0.12583655
ShSirt1_vs_ShScrambled	Q8VDL4	Q8VDL4	Adpgk	72141	-0.518140695	0.02177503	0.12583655
ShSirt1_vs_ShScrambled	Q8BRG8	Q8BRG8	Tmem209	72649	0.48934462	0.02181686	0.12583655
ShSirt1_vs_ShScrambled	Q9D051	Q9D051	Pdhb	68263	-0.400416438	0.02184006	0.12583655
ShSirt1_vs_ShScrambled	Q7TNG5	Q7TNG5	Eml2	72205	0.481498489	0.02186035	0.12583655
ShSirt1_vs_ShScrambled	Q6NZN0	Q6NZN0	Rbm26	74213	0.525740612	0.02191401	0.12583655
ShSirt1_vs_ShScrambled	Q922W5	Q922W5	Pycr1	209027	0.706519797	0.02193002	0.12583655
ShSirt1_vs_ShScrambled	Q8R015	Q8R015	Bloc1s5	17828	1.364648775	0.02193946	0.12583655
ShSirt1_vs_ShScrambled	Q9DCX2	Q9DCX2	Atp5h	71679	-0.61497484	0.02195317	0.12583655
ShSirt1_vs_ShScrambled	Q9CXF4	Q9CXF4	Tbc1d15	66687	0.657131788	0.02197723	0.12583655
ShSirt1_vs_ShScrambled	Q9R0Z9	Q9R0Z9	Dlc1	50768	-1.494216596	0.02197952	0.12583655
ShSirt1_vs_ShScrambled	P27601	P27601	Gna13	14674	0.491181789	0.02199172	0.12583655
ShSirt1_vs_ShScrambled	P19536	P19536	Cox5b		-0.563991033	0.02199491	0.12583655
ShSirt1_vs_ShScrambled	P63154	P63154	Crnkl1	66877	-1.38317187	0.02206823	0.1260962
ShSirt1_vs_ShScrambled	Q9D0R8	Q9D0R8	Lsm12	268490	-0.915931111	0.02214784	0.12613926
ShSirt1_vs_ShScrambled	Q9D0M0	Q9D0M0	Exosc7	66446	-0.773045873	0.02215507	0.12613926
ShSirt1_vs_ShScrambled	Q6ZWU9	Q6ZWU9	Rps27	57294; 10	0.427233132	0.02217448	0.12613926
ShSirt1_vs_ShScrambled	Q922B9	Q922B9	Ssfa2	70599	-1.065814966	0.02220082	0.12613926
ShSirt1_vs_ShScrambled	Q9CRA7	Q9CRA7	Atp5s	68055	1.261956665	0.02224295	0.12613926
ShSirt1_vs_ShScrambled	Q9WUR9	Q9WUR9	Ak4	11639	0.598347327	0.02224343	0.12613926
ShSirt1_vs_ShScrambled	Q7M759	Q7M759	Abhd17b	226016	-1.275679574	0.02227632	0.12616725
ShSirt1_vs_ShScrambled	Q9Z2X1	Q9Z2X1	Hnrnpf	98758	0.425404858	0.02234988	0.12642525
ShSirt1_vs_ShScrambled	Q8BMS1	Q8BMS1	Hadha	97212	-0.406325459	0.02241411	0.12662989
ShSirt1_vs_ShScrambled	Q9D6K5	Q9D6K5	Synj2bp	24071; 10	-0.632056947	0.02258606	0.12741143
ShSirt1_vs_ShScrambled	P63044	P63044	Vamp2	22318	1.401005469	0.02261824	0.12741143
ShSirt1_vs_ShScrambled	Q8BG30	Q8BG30	Nelfa	24116	0.97495252	0.02267926	0.12741143
ShSirt1_vs_ShScrambled	Q60902	Q60902	Eps15l1	13859	0.607210266	0.02270926	0.12741143
ShSirt1_vs_ShScrambled	Q8BJU9	Q8BJU9	Mtrf1l	108853	-2.442937955	0.02271717	0.12741143
ShSirt1_vs_ShScrambled	Q8CH72	Q8CH72	Trim32	69807	-1.073562208	0.0227218	0.12741143
ShSirt1_vs_ShScrambled	P48962	P48962	Slc25a4	11739	-0.479366743	0.0230464	0.12900836
ShSirt1_vs_ShScrambled	Q9D7B7	Q9D7B7	Gpx8	69590	-2.128517486	0.02306374	0.12900836
ShSirt1_vs_ShScrambled	Q9QZD8	Q9QZD8	Slc25a10	27376	0.35038722	0.02313911	0.12926973
ShSirt1_vs_ShScrambled	A2ADY9	A2ADY9	Ddi2	68817	0.544514701	0.02328032	0.12987443
ShSirt1_vs_ShScrambled	Q9D8Y0	Q9D8Y0	Efhd2		0.586793106	0.02330801	0.12987443
ShSirt1_vs_ShScrambled	Q9D6N1	Q9D6N1	Ca13	71934	-0.414611362	0.02333367	0.12987443
ShSirt1_vs_ShScrambled	Q8BGK6	Q8BGK6	Slc7a6	330836	-0.85825689	0.02338541	0.13000213

ShSirt1_vs_ShScrambled	P51912	P51912	Slc1a5		0.483380883	0.02368535	0.1315076
ShSirt1_vs_ShScrambled	P19157	P19157;P464	Gstp1		-0.397735775	0.02404071	0.13331667
ShSirt1_vs_ShScrambled	Q921G8	Q921G8	Tubgcp2	74237	0.646179788	0.02413323	0.13366122
ShSirt1_vs_ShScrambled	Q9EQH3	Q9EQH3	Vps35	65114	-0.473615642	0.02416206	0.13366122
ShSirt1_vs_ShScrambled	Q61122	Q61122	Nab1	17936	-2.055100113	0.02427201	0.13383004
ShSirt1_vs_ShScrambled	Q61550	Q61550	Rad21	19357	0.81057595	0.02427536	0.13383004
ShSirt1_vs_ShScrambled	Q922U1	Q922U1	Prpf3	70767	0.725063002	0.02430129	0.13383004
ShSirt1_vs_ShScrambled	G5E8F4	G5E8F4	Fpgt	75540	-0.544281772	0.02435139	0.13383004
ShSirt1_vs_ShScrambled	Q9D2C7	Q9D2C7	Tmbim6	110213	-0.741400357	0.02436151	0.13383004
ShSirt1_vs_ShScrambled	Q8R2W9	Q8R2W9	Pank3	211347	-0.763771128	0.02438109	0.13383004
ShSirt1_vs_ShScrambled	Q61214	Q61214	Dyrk1a	13548	-1.307339647	0.02440012	0.13383004
ShSirt1_vs_ShScrambled	Q62059	Q62059	Vcan		1.109361092	0.02449154	0.13408887
ShSirt1_vs_ShScrambled	O35099	O35099	Map3k5	26408	-2.045053448	0.02450672	0.13408887
ShSirt1_vs_ShScrambled	P49442	P49442	Inpp1	16329	0.811204454	0.0246328	0.13461556
ShSirt1_vs_ShScrambled	Q99NB9	Q99NB9	Sf3b1		0.393425017	0.02466683	0.13463853
ShSirt1_vs_ShScrambled	Q99JB8	Q99JB8	Pacsin3	80708	0.831141247	0.02472438	0.13478967
ShSirt1_vs_ShScrambled	Q9D3P8	Q9D3P8	Plgrkt	67759	-0.643023845	0.02475887	0.13481488
ShSirt1_vs_ShScrambled	Q9QYR6	Q9QYR6	Map1a	17754	-0.517077648	0.02485665	0.13483309
ShSirt1_vs_ShScrambled	Q80YV2	Q80YV2	Zc3hc1	232679	0.824295915	0.02493559	0.13483309
ShSirt1_vs_ShScrambled	Q8BGQ6	Q8BGQ6	Efcab14	230648	-2.026855081	0.02494044	0.13483309
ShSirt1_vs_ShScrambled	P58854	P58854	Tubgcp3	259279	0.799297256	0.02496654	0.13483309
ShSirt1_vs_ShScrambled	Q9WUZ9	Q9WUZ9	Entpd5	12499	-0.784052687	0.02499397	0.13483309
ShSirt1_vs_ShScrambled	P37238	P37238	Pparg	19016	2.721055369	0.02499489	0.13483309
ShSirt1_vs_ShScrambled	Q99KK2	Q99KK2	Cmas	12764	-0.550717874	0.02499686	0.13483309
ShSirt1_vs_ShScrambled	Q99KE1	Q99KE1	Me2	107029	-0.527290056	0.02502492	0.13483309
ShSirt1_vs_ShScrambled	Q9CQ22	Q9CQ22	Lamtor1	66508	-0.674601003	0.02503104	0.13483309
ShSirt1_vs_ShScrambled	P38647	P38647	Hspa9	15526	-0.395084972	0.02508336	0.13495386
ShSirt1_vs_ShScrambled	Q9ET30	Q9ET30	Tm9sf3	107358	0.805700369	0.02514584	0.13512896
ShSirt1_vs_ShScrambled	Q9ESW8	Q9ESW8	Pgpep1	66522	-0.544916202	0.02520685	0.13525119
ShSirt1_vs_ShScrambled	Q61411	Q61411	Hras	15461	0.608068953	0.02522851	0.13525119
ShSirt1_vs_ShScrambled	Q6P6J9	Q6P6J9	Txndc15	69672	-0.565053882	0.02532613	0.13550377
ShSirt1_vs_ShScrambled	Q62093	Q62093	Srsf2	20382	0.399282097	0.02533566	0.13550377
ShSirt1_vs_ShScrambled	Q8BWR4	Q8BWR4	Usp40	227334	0.942160666	0.02546231	0.13601996
ShSirt1_vs_ShScrambled	Q8VE98	Q8VE98	Cd276	102657	-1.086090154	0.02558034	0.13648893
ShSirt1_vs_ShScrambled	P84228	P84228;P684	Hist1h3b		0.531535536	0.02566122	0.13662454
ShSirt1_vs_ShScrambled	Q6P2B1	Q6P2B1	Tnpo3	320938	0.462795625	0.02566629	0.13662454
ShSirt1_vs_ShScrambled	Q9WU79	Q9WU79	Prodh	19125	0.493979793	0.02571104	0.13670156
ShSirt1_vs_ShScrambled	P35123	P35123	Usp4	22258	-0.461608018	0.02578163	0.13691563
ShSirt1_vs_ShScrambled	Q99LS3	Q99LS3	Psph	100678	0.562194805	0.02587288	0.13715347
ShSirt1_vs_ShScrambled	Q8BU31	Q8BU31	Rap2c	72065	-1.090395283	0.02588719	0.13715347
ShSirt1_vs_ShScrambled	Q9QZM0	Q9QZM0	Ubqln2	54609	-0.683850025	0.0259939	0.1375574
ShSirt1_vs_ShScrambled	Q8C129	Q8C129	Lnpep	240028	0.397772035	0.02618976	0.13843159
ShSirt1_vs_ShScrambled	Q9WVA2	Q9WVA2;Q4	Timm8a1		-1.974714591	0.02624745	0.13857425
ShSirt1_vs_ShScrambled	Q61249	Q61249	Igbp1	18518	-0.3894022	0.02636652	0.13881322
ShSirt1_vs_ShScrambled	P68368	P68368	Tuba4a	22145	0.869104966	0.02637183	0.13881322
ShSirt1_vs_ShScrambled	O89016	O89016	Abcd4	19300	1.490094146	0.02638497	0.13881322
ShSirt1_vs_ShScrambled	Q64311	Q64311	Ntan1	18203	0.74930287	0.02648802	0.13919316
ShSirt1_vs_ShScrambled	Q9DBS5	Q9DBS5	Klc4	74764	0.446583991	0.02652163	0.13920773
ShSirt1_vs_ShScrambled	Q501J2	Q501J2	Fam173a	214917	0.789260065	0.02659169	0.13941335
ShSirt1_vs_ShScrambled	P10833	P10833	Rras	20130	0.777366063	0.02675275	0.13996375
ShSirt1_vs_ShScrambled	P24788	P24788	Cdk11b	12537	0.496870832	0.02675869	0.13996375
ShSirt1_vs_ShScrambled	Q8VEF1	Q8VEF1	Gramd1a	52857	-0.762317858	0.02690991	0.14059183
ShSirt1_vs_ShScrambled	O55135	O55135	Eif6	16418	0.412008144	0.02698104	0.14064821
ShSirt1_vs_ShScrambled	Q8BSK8	Q8BSK8	Rps6kb1	72508	3.18701179	0.02698302	0.14064821
ShSirt1_vs_ShScrambled	Q3TP92	Q3TP92	Ctdnep1	67181	1.944218635	0.027059	0.14088159
ShSirt1_vs_ShScrambled	P56135	P56135	Atp5j2	57423	-0.398304928	0.0271681	0.14128664
ShSirt1_vs_ShScrambled	P16254	P16254	Srp14	20813	0.861549582	0.02720204	0.14130036
ShSirt1_vs_ShScrambled	Q8R0F8	Q8R0F8	Fahd1	68636	-0.665620979	0.02723899	0.14132967
ShSirt1_vs_ShScrambled	Q8CDD8	Q8CDD8	Kctd20	66989	-1.935370594	0.02730136	0.14136168
ShSirt1_vs_ShScrambled	Q4U2R1	Q4U2R1	Herc2	15204	-0.724625695	0.02730779	0.14136168
ShSirt1_vs_ShScrambled	Q9R257	Q9R257	Hebp1	15199	0.560866178	0.02735545	0.14144616
ShSirt1_vs_ShScrambled	O88428	O88428	Papss2	23972	0.566076283	0.02744443	0.14152854
ShSirt1_vs_ShScrambled	Q6ZWX6	Q6ZWX6	Eif2s1	13665	-0.425298358	0.02745741	0.14152854

ShSirt1_vs_ShScrambled	Q99LG0	Q99LG0	Usp16	74112	1.009870871	0.02746544	0.14152854
ShSirt1_vs_ShScrambled	Q80U95	Q80U95	Ube3c	100763	0.554156317	0.0276313	0.14213196
ShSirt1_vs_ShScrambled	Q9CR20	Q9CR20	Ier3ip1	66191	-1.135856281	0.02772736	0.14213196
ShSirt1_vs_ShScrambled	Q61545	Q61545	Ewsr1	14030	0.407461981	0.02773444	0.14213196
ShSirt1_vs_ShScrambled	P51174	P51174	Acadl	11363	-0.699766118	0.02775601	0.14213196
ShSirt1_vs_ShScrambled	Q9Z2I9	Q9Z2I9	Sucla2	20916	-0.413047426	0.0277761	0.14213196
ShSirt1_vs_ShScrambled	P52927	P52927	Hmga2	15364	1.322415371	0.02778897	0.14213196
ShSirt1_vs_ShScrambled	Q9WUQ2	Q9WUQ2	Preb	50907	0.362445375	0.02780295	0.14213196
ShSirt1_vs_ShScrambled	Q99N57	Q99N57	Raf1	110157	1.110616386	0.02803839	0.14317341
ShSirt1_vs_ShScrambled	O09111	O09111	Ndufb11	104130	-1.642049819	0.02814447	0.14355269
ShSirt1_vs_ShScrambled	Q99MN1	Q99MN1	Kars	85305	-0.355133502	0.02822129	0.14368591
ShSirt1_vs_ShScrambled	P97287	P97287	Mcl1	17210	-0.612876756	0.02823425	0.14368591
ShSirt1_vs_ShScrambled	P34914	P34914	Ephx2	13850	0.33049193	0.02831962	0.14395806
ShSirt1_vs_ShScrambled	P61759	P61759	Vbp1	22327	1.185986035	0.02841528	0.14428185
ShSirt1_vs_ShScrambled	O35114	O35114	Scarb2	12492	-0.55418617	0.02856032	0.14485537
ShSirt1_vs_ShScrambled	Q99PV0	Q99PV0	Prpf8	192159	0.416856848	0.02868398	0.14490755
ShSirt1_vs_ShScrambled	Q8C3X8	Q8C3X8	Lmf2	105847	0.772001244	0.02869112	0.14490755
ShSirt1_vs_ShScrambled	P34022	P34022	Ranbp1	19385	0.411108964	0.02871209	0.14490755
ShSirt1_vs_ShScrambled	Q62165	Q62165	Dag1	13138	0.528067987	0.02874747	0.14490755
ShSirt1_vs_ShScrambled	P11352	P11352	Gpx1	14775	-0.448493101	0.02875967	0.14490755
ShSirt1_vs_ShScrambled	Q8CDN6	Q8CDN6	Txnl1	53382	0.509791996	0.02876322	0.14490755
ShSirt1_vs_ShScrambled	Q8BGS7	Q8BGS7	Cept1	99712	0.634442222	0.02879697	0.14491588
ShSirt1_vs_ShScrambled	O89116	O89116	Vti1a	53611	0.786452775	0.02884499	0.14499584
ShSirt1_vs_ShScrambled	Q8C7V3	Q8C7V3	Utp15	105372	0.368609131	0.02889313	0.14507631
ShSirt1_vs_ShScrambled	Q8BFS6	Q8BFS6	Cpped1	223978	0.657840241	0.0290574	0.14546853
ShSirt1_vs_ShScrambled	Q8K0C9	Q8K0C9	Gmds	218138	0.91409253	0.02906531	0.14546853
ShSirt1_vs_ShScrambled	Q9CQP0	Q9CQP0	Mrpl33	66845	-0.936297621	0.02906792	0.14546853
ShSirt1_vs_ShScrambled	E9Q4Z2	E9Q4Z2	Acacb	100705	-0.731270991	0.02914968	0.1457161
ShSirt1_vs_ShScrambled	Q64430	Q64430	Atp7a	11977	-0.511778205	0.02927799	0.14609654
ShSirt1_vs_ShScrambled	Q8VDJ3	Q8VDJ3	Hdlbp	110611	-0.330863689	0.02929051	0.14609654
ShSirt1_vs_ShScrambled	P20060	P20060	Hexb	15212	-0.400539995	0.02946863	0.14677101
ShSirt1_vs_ShScrambled	Q9D9N8	Q9D9N8	Pradc1	73327	-1.109135855	0.02949076	0.14677101
ShSirt1_vs_ShScrambled	Q99KV1	Q99KV1	Dnajb11	67838	-0.439957917	0.0297735	0.14791802
ShSirt1_vs_ShScrambled	P00520	P00520	Abl1	11350	-0.974768742	0.02981572	0.14791802
ShSirt1_vs_ShScrambled	P62821	P62821	Rab1A	19324	-0.563533298	0.02983947	0.14791802
ShSirt1_vs_ShScrambled	O35900	O35900	Lsm2	27756	0.749377169	0.02985231	0.14791802
ShSirt1_vs_ShScrambled	O35143	O35143	Atpif1	11983	-1.273932747	0.0299699	0.1481305
ShSirt1_vs_ShScrambled	P52623	P52623	Uck1	22245	-0.605290211	0.02999873	0.1481305
ShSirt1_vs_ShScrambled	O70456	O70456	Sfn	55948	0.685018978	0.03000871	0.1481305
ShSirt1_vs_ShScrambled	Q8BL66	Q8BL66	Eea1	216238	-0.485692896	0.03002645	0.1481305
ShSirt1_vs_ShScrambled	P46471	P46471	Psmc2		-0.338899023	0.03009741	0.14822952
ShSirt1_vs_ShScrambled	Q60823	Q60823	Akt2	11652	-0.756957829	0.03013053	0.14822952
ShSirt1_vs_ShScrambled	Q8VC42	Q8VC42	Mic1	76482	-0.560467425	0.03014504	0.14822952
ShSirt1_vs_ShScrambled	Q8CB44	Q8CB44	Gramd4	223752	1.847704929	0.03057156	0.15010128
ShSirt1_vs_ShScrambled	Q8R3I3	Q8R3I3	Cog6	67542	0.389740774	0.0305922	0.15010128
ShSirt1_vs_ShScrambled	Q3UVL4	Q3UVL4	Vps51	68505	0.498295427	0.03066615	0.15030078
ShSirt1_vs_ShScrambled	Q9CXW3	Q9CXW3	Cacybp	12301	0.357205684	0.03072275	0.15040111
ShSirt1_vs_ShScrambled	Q8VE88	Q8VE88	Fam114a2	67726	0.61480691	0.03076071	0.15040111
ShSirt1_vs_ShScrambled	P08207	P08207	S100a10	20194	-0.680675929	0.03085203	0.15040111
ShSirt1_vs_ShScrambled	Q9CXL3	Q9CXL3	Q9CXL3	73212	0.680088199	0.03085628	0.15040111
ShSirt1_vs_ShScrambled	Q3U308	Q3U308	Ctu2	66965	-1.816350681	0.03089618	0.15040111
ShSirt1_vs_ShScrambled	P29351	P29351	Ptpn6	15170	1.044248944	0.03090332	0.15040111
ShSirt1_vs_ShScrambled	Q5XG71	Q5XG71	Utp20		-0.876223332	0.03091986	0.15040111
ShSirt1_vs_ShScrambled	Q9WV03	Q9WV03	Fam50a	108160	1.155012461	0.03096154	0.15044177
ShSirt1_vs_ShScrambled	Q3UL36	Q3UL36	Arglu1	234023	1.770746195	0.03104915	0.1505819
ShSirt1_vs_ShScrambled	Q99LQ7	Q99LQ7	Tmem189	407243	-0.791315817	0.0310571	0.1505819
ShSirt1_vs_ShScrambled	Q64514	Q64514	Tpp2	22019	-0.339214552	0.03110625	0.15065838
ShSirt1_vs_ShScrambled	P51175	P51175	Ppox	19044	0.43045701	0.03119486	0.15080171
ShSirt1_vs_ShScrambled	Q8CJ40	Q8CJ40	Crocc	230872	0.948372194	0.03124874	0.15080171
ShSirt1_vs_ShScrambled	Q9JHU4	Q9JHU4	Dync1h1	13424	-0.318332928	0.0312582	0.15080171
ShSirt1_vs_ShScrambled	Q61147	Q61147	Cp	12870	-0.859899595	0.03126947	0.15080171
ShSirt1_vs_ShScrambled	Q9D6S7	Q9D6S7	Mrrf	67871	0.797876893	0.03141156	0.15132528
ShSirt1_vs_ShScrambled	P53986	P53986	Slc16a1	20501	0.81402245	0.03150487	0.15142262

ShSirt1_vs_ShScrambled	Q6P549	Q6P549	Inppl1	16332	-0.446015906	0.03153171	0.15142262
ShSirt1_vs_ShScrambled	Q5HZI9	Q5HZI9	Slc25a51	230125	-0.359828016	0.03153949	0.15142262
ShSirt1_vs_ShScrambled	P80313	P80313	Cct7	12468	0.388999294	0.03156595	0.15142262
ShSirt1_vs_ShScrambled	Q99020	Q99020	Hnrnpab	15384	0.557729998	0.03172258	0.15187823
ShSirt1_vs_ShScrambled	Q8CEE7	Q8CEE7	Rdh13	108841	-1.031069418	0.03172822	0.15187823
ShSirt1_vs_ShScrambled	Q9QZH3	Q9QZH3	Ppie	56031	1.737629695	0.03185276	0.1521952
ShSirt1_vs_ShScrambled	Q64327	Q64327	Mea1	17256	0.413724795	0.03186187	0.1521952
ShSirt1_vs_ShScrambled	Q9ERA6	Q9ERA6	Tfip11	54723	0.50570261	0.03194822	0.1523231
ShSirt1_vs_ShScrambled	Q8CC88	Q8CC88	Vwa8	219189	-0.631113313	0.03195613	0.1523231
ShSirt1_vs_ShScrambled	Q8K3H0	Q8K3H0	Appl1	72993	0.452728392	0.03199573	0.15235097
ShSirt1_vs_ShScrambled	Q8BHL3	Q8BHL3	Tbc1d10b	68449	0.43951198	0.03206001	0.1524962
ShSirt1_vs_ShScrambled	Q91V01	Q91V01	Lpcat3	14792	0.403831659	0.03209517	0.15250272
ShSirt1_vs_ShScrambled	Q8JZQ2	Q8JZQ2	Afg3l2	69597	-0.455976898	0.03231027	0.15336337
ShSirt1_vs_ShScrambled	Q91VR2	Q91VR2	Atp5c1	11949	-0.352063739	0.03240724	0.15366207
ShSirt1_vs_ShScrambled	P70444	P70444	Bid	12122	-3.844461961	0.03249257	0.15388338
ShSirt1_vs_ShScrambled	P36371	P36371	Tap2	21355	-0.780794743	0.03253044	0.15388338
ShSirt1_vs_ShScrambled	Q3TW96	Q3TW96	Uap1l1	227620	-0.354298134	0.03255619	0.15388338
ShSirt1_vs_ShScrambled	O08553	O08553	Dpysl2	12934	-0.342285232	0.0327238	0.15419104
ShSirt1_vs_ShScrambled	O08992	O08992	Sdcbp	53378	1.011840459	0.03277641	0.15419104
ShSirt1_vs_ShScrambled	P57784	P57784	Snrpa1	68981	0.34686743	0.03279059	0.15419104
ShSirt1_vs_ShScrambled	Q8VDC1	Q8VDC1	Fyco1	17281	-0.523958706	0.03282157	0.15419104
ShSirt1_vs_ShScrambled	Q9D1K2	Q9D1K2	Atp6v1f	66144	-0.82854518	0.03284161	0.15419104
ShSirt1_vs_ShScrambled	Q91X96	Q91X96	Rabif	98710	2.250863241	0.03285376	0.15419104
ShSirt1_vs_ShScrambled	P16882	P16882	Ghr	14600	-1.758814958	0.03288708	0.15419104
ShSirt1_vs_ShScrambled	Q9DCT1	Q9DCT1	Akr1e2	56043	1.700785269	0.03289454	0.15419104
ShSirt1_vs_ShScrambled	Q9D855	Q9D855	Uqcrb		-0.454593246	0.03299664	0.15450914
ShSirt1_vs_ShScrambled	P47857	P47857	Pfkm	18642	0.356409639	0.03314353	0.15503618
ShSirt1_vs_ShScrambled	P68037	P68037	Ube2l3	22195	0.595167463	0.03318606	0.15507442
ShSirt1_vs_ShScrambled	Q7M6Y3	Q7M6Y3	Picalm	233489	-0.35710247	0.03330327	0.15546119
ShSirt1_vs_ShScrambled	O35685	O35685	Nudc	18221	0.524015205	0.03334124	0.15547764
ShSirt1_vs_ShScrambled	Q8R0F6	Q8R0F6	Ilkap	67444	0.769513096	0.03352197	0.15574634
ShSirt1_vs_ShScrambled	Q9D6M3	Q9D6M3	Slc25a22	68267	-0.808572258	0.03355502	0.15574634
ShSirt1_vs_ShScrambled	Q9D1I2	Q9D1I2	Q9D1I2	68480	1.740447617	0.03356224	0.15574634
ShSirt1_vs_ShScrambled	Q9CQ60	Q9CQ60	Pgls	66171	-0.850540146	0.03356526	0.15574634
ShSirt1_vs_ShScrambled	Q9CZD5	Q9CZD5	Mtif3	76366	1.740011787	0.03357851	0.15574634
ShSirt1_vs_ShScrambled	Q6PD26	Q6PD26	Pigs	276846	0.537814779	0.03365689	0.15574634
ShSirt1_vs_ShScrambled	Q91WG4	Q91WG4	Elp2	58523	0.734493899	0.03368027	0.15574634
ShSirt1_vs_ShScrambled	Q810A7	Q810A7	Ddx42	72047	0.505721342	0.03368034	0.15574634
ShSirt1_vs_ShScrambled	Q9QYR9	Q9QYR9	Acot2	171210	0.367987382	0.03370939	0.15574634
ShSirt1_vs_ShScrambled	Q923X4	Q923X4	Glrx2	69367	-1.732509909	0.03386031	0.15628369
ShSirt1_vs_ShScrambled	O08579	O08579	Emd	13726	0.382139238	0.03400525	0.15664456
ShSirt1_vs_ShScrambled	Q8CB77	Q8CB77	Tceb3	27224	-0.483162286	0.03401364	0.15664456
ShSirt1_vs_ShScrambled	Q8R0K4	Q8R0K4	Ccdc137	67291	0.869537244	0.03406966	0.15664456
ShSirt1_vs_ShScrambled	Q61398	Q61398	Pcolce	18542	0.555293739	0.03407731	0.15664456
ShSirt1_vs_ShScrambled	Q689Z5	Q689Z5	Sbno1		0.885950556	0.03414552	0.15679843
ShSirt1_vs_ShScrambled	Q9CQF0	Q9CQF0	Mrpl11	66419	-0.331676183	0.03420268	0.15690132
ShSirt1_vs_ShScrambled	Q6ZQK5	Q6ZQK5	Acap2	78618	0.399861305	0.03439291	0.1576138
ShSirt1_vs_ShScrambled	P70280	P70280	Vamp7	20955	-0.810126234	0.03444974	0.15771412
ShSirt1_vs_ShScrambled	P46978	P46978	Stt3a	16430	0.337607376	0.03451862	0.15786937
ShSirt1_vs_ShScrambled	Q61102	Q61102	Abcb7	11306	0.740998254	0.0346462	0.15814035
ShSirt1_vs_ShScrambled	Q9DB26	Q9DB26	Phyhd1	227696	0.634469775	0.03464794	0.15814035
ShSirt1_vs_ShScrambled	P97434	P97434	Mprip	26936	-2.051281359	0.03473488	0.15825003
ShSirt1_vs_ShScrambled	Q99LY9	Q99LY9	Ndufs5	595136	-3.252972875	0.03475969	0.15825003
ShSirt1_vs_ShScrambled	P21619	P21619	Lmnb2	16907	0.951460869	0.03479835	0.15825003
ShSirt1_vs_ShScrambled	P62855	P62855	Rps26	27370	-0.526121606	0.03491962	0.15825003
ShSirt1_vs_ShScrambled	O88958	O88958	Gnpda1	26384	-0.359398548	0.03493109	0.15825003
ShSirt1_vs_ShScrambled	O55033	O55033	Nck2	17974	0.875589475	0.03498629	0.15825003
ShSirt1_vs_ShScrambled	Q9D7N6	Q9D7N6	Mrpl30	107734	-1.033724814	0.03500651	0.15825003
ShSirt1_vs_ShScrambled	O70566	O70566	Diaph2	54004	-0.36708779	0.03501037	0.15825003
ShSirt1_vs_ShScrambled	Q8BHG2	Q8BHG2	Q8BHG2	74098	-1.258652169	0.03501317	0.15825003
ShSirt1_vs_ShScrambled	Q920Q4	Q920Q4	Vps16		-0.422805888	0.03502255	0.15825003
ShSirt1_vs_ShScrambled	O70496	O70496	Clcn7	26373	-0.82486682	0.03505903	0.15825644
ShSirt1_vs_ShScrambled	Q9CPW4	Q9CPW4	Arpc5	67771	-0.369752892	0.03518889	0.15868051

ShSirt1_vs_ShScrambled	P70268	P70268	Pkn1	320795	0.872746576	0.03522328	0.15868051
ShSirt1_vs_ShScrambled	Q8C147	Q8C147	Dock8	76088	-0.932723389	0.03528092	0.15871447
ShSirt1_vs_ShScrambled	O88379	O88379	Baz1a		-1.609627766	0.03530114	0.15871447
ShSirt1_vs_ShScrambled	Q4VGL6	Q4VGL6	Rc3h1	381305	-1.15385087	0.03541845	0.15908345
ShSirt1_vs_ShScrambled	Q7TPE5	Q7TPE5	Slc7a6os	66432	-2.145712474	0.03549115	0.15925155
ShSirt1_vs_ShScrambled	Q9JLV1	Q9JLV1	Bag3	29810	-0.842857629	0.03563028	0.15956881
ShSirt1_vs_ShScrambled	P61222	P61222	Abce1	24015	-0.348708586	0.03572046	0.15956881
ShSirt1_vs_ShScrambled	Q6TEK5	Q6TEK5	Vkorc1l1	69568	0.508377707	0.03572733	0.15956881
ShSirt1_vs_ShScrambled	P53996	P53996	Cnbp	12785	0.571395156	0.0357356	0.15956881
ShSirt1_vs_ShScrambled	Q9Z130	Q9Z130	Hnrnpdl		1.59040016	0.03577286	0.15956881
ShSirt1_vs_ShScrambled	Q80TL7	Q80TL7	Mon2	67074	0.411099298	0.03577396	0.15956881
ShSirt1_vs_ShScrambled	Q9D1I6	Q9D1I6	Mrpl14	68463	-0.664565573	0.03602077	0.1603695
ShSirt1_vs_ShScrambled	Q08857	Q08857	Cd36	12491	-0.360948172	0.03605092	0.1603695
ShSirt1_vs_ShScrambled	Q9JL56	Q9JL56	Gde1	56209	-0.743181749	0.03606004	0.1603695
ShSirt1_vs_ShScrambled	Q9EPJ9	Q9EPJ9	Arfgap1	228998	0.44002354	0.0361721	0.16070951
ShSirt1_vs_ShScrambled	P29533	P29533	Vcam1		-0.717706078	0.03622866	0.16080251
ShSirt1_vs_ShScrambled	Q8BU30	Q8BU30	Iars	105148	0.406001588	0.0362971	0.16094803
ShSirt1_vs_ShScrambled	Q9CR24	Q9CR24	Nudt8	66387	0.869523458	0.03635633	0.16105247
ShSirt1_vs_ShScrambled	P42227	P42227	Stat3	20848	0.404868427	0.03643731	0.16125296
ShSirt1_vs_ShScrambled	Q99JN2	Q99JN2	Klhl22	224023	0.574519724	0.03648586	0.16130966
ShSirt1_vs_ShScrambled	Q9D6N5	Q9D6N5	Drap1	66556	0.625275239	0.03660832	0.16169272
ShSirt1_vs_ShScrambled	Q8BKU8	Q8BKU8	Tmem87b	72477	1.262147278	0.03671116	0.16198844
ShSirt1_vs_ShScrambled	Q9QUH0	Q9QUH0	Glrx	93692	-0.576597326	0.03694096	0.16227848
ShSirt1_vs_ShScrambled	Q7JJ13	Q7JJ13	Brd2	14312	0.574488233	0.0369761	0.16227848
ShSirt1_vs_ShScrambled	Q3THS6	Q3THS6	Mat2a	232087	-0.410035197	0.03701227	0.16227848
ShSirt1_vs_ShScrambled	Q8BMK4	Q8BMK4	Ckap4	216197	0.330509469	0.03704075	0.16227848
ShSirt1_vs_ShScrambled	Q810A3	Q810A3	Ttc9c	70387	-0.849571591	0.0370564	0.16227848
ShSirt1_vs_ShScrambled	Q60605	Q60605	Myl6	17904	-0.568848161	0.03706591	0.16227848
ShSirt1_vs_ShScrambled	Q9JHI5	Q9JHI5	Ivd	56357	-0.342270089	0.0370956	0.16227848
ShSirt1_vs_ShScrambled	Q91W53	Q91W53	Golga7	57437	-1.035034626	0.03709848	0.16227848
ShSirt1_vs_ShScrambled	Q91XU0	Q91XU0	Wrnip1	78903	-1.486745362	0.03710044	0.16227848
ShSirt1_vs_ShScrambled	Q00PI9	Q00PI9	Hnrnpul2	68693	0.384698918	0.03716764	0.16241503
ShSirt1_vs_ShScrambled	Q9DC69	Q9DC69	Ndufa9	66108	-0.435294134	0.03725376	0.16263393
ShSirt1_vs_ShScrambled	P38060	P38060	Hmgcl	15356	0.422244235	0.03744501	0.16331091
ShSirt1_vs_ShScrambled	Q8BGT5	Q8BGT5	Gpt2	108682	0.335040238	0.03751257	0.16333498
ShSirt1_vs_ShScrambled	Q9JHS4	Q9JHS4	Clpx	270166	-0.446572879	0.03758928	0.16333498
ShSirt1_vs_ShScrambled	P70257	P70257	Nfix	18032	-0.633012541	0.03759009	0.16333498
ShSirt1_vs_ShScrambled	Q6XUX1	Q6XUX1	Dstyk	213452	-0.521699006	0.03762021	0.16333498
ShSirt1_vs_ShScrambled	P42669	P42669	Pura	19290	0.369643918	0.03763145	0.16333498
ShSirt1_vs_ShScrambled	Q9JKN1	Q9JKN1	Slc30a7	66500	-0.488262186	0.03774567	0.16367333
ShSirt1_vs_ShScrambled	P30412	P30412	Ppic	19038	-0.610820504	0.03779242	0.16371878
ShSirt1_vs_ShScrambled	P15920	P15920	Atp6v0a2	21871	-0.502760223	0.0378324	0.16373487
ShSirt1_vs_ShScrambled	P57759	P57759	Erp29	67397	0.345763838	0.03788129	0.16378941
ShSirt1_vs_ShScrambled	Q3TVI8	Q3TVI8	Pbxip1	229534	-1.63307246	0.03794641	0.16391396
ShSirt1_vs_ShScrambled	P15626	P15626	Gstm2	14863	-0.377476888	0.03801815	0.16395882
ShSirt1_vs_ShScrambled	Q9Z2A7	Q9Z2A7	Dgat1	13350	-0.392017215	0.03805385	0.16395882
ShSirt1_vs_ShScrambled	O35704	O35704	Sptlc1	268656	-0.475049892	0.03806576	0.16395882
ShSirt1_vs_ShScrambled	Q99JF8	Q99JF8	Psip1	101739	0.432813264	0.03817264	0.16418923
ShSirt1_vs_ShScrambled	Q67FY2	Q67FY2	Bcl9l	80288	0.544160581	0.038192	0.16418923
ShSirt1_vs_ShScrambled	O70439	O70439	Stx7		-0.783229904	0.03834976	0.16462906
ShSirt1_vs_ShScrambled	Q9WTP6	Q9WTP6	Ak2	11637	-0.414860184	0.03836725	0.16462906
ShSirt1_vs_ShScrambled	Q9DCG9	Q9DCG9	Trmt112	67674	0.422779654	0.03852769	0.1651605
ShSirt1_vs_ShScrambled	Q8R3G1	Q8R3G1	Ppp1r8	100336	0.482855164	0.03869815	0.16573381
ShSirt1_vs_ShScrambled	Q9JJV2	Q9JJV2	Pfn2	18645	-0.996904963	0.03886309	0.16613888
ShSirt1_vs_ShScrambled	Q61151	Q61151	Ppp2r5e	26932	-0.370924808	0.03888351	0.16613888
ShSirt1_vs_ShScrambled	Q04207	Q04207	Rela	19697	0.869510431	0.0389259	0.16613888
ShSirt1_vs_ShScrambled	Q8BGF6	Q8BGF6	Elmod2	244548	1.754684468	0.03893995	0.16613888
ShSirt1_vs_ShScrambled	Q9JIM1	Q9JIM1	Slc29a1	63959	0.844686932	0.03910393	0.16668096
ShSirt1_vs_ShScrambled	Q8C7X2	Q8C7X2	Emc1	230866	-0.423367608	0.03917117	0.16669514
ShSirt1_vs_ShScrambled	P40142	P40142	Tkt	21881	-0.308204095	0.03918111	0.16669514
ShSirt1_vs_ShScrambled	Q9CW46	Q9CW46	Raver1	71766	0.376064555	0.03931118	0.16709103
ShSirt1_vs_ShScrambled	Q8BYB9	Q8BYB9	Poglut1	224143	1.437494674	0.0394841	0.16763725
ShSirt1_vs_ShScrambled	Q3UKJ7	Q3UKJ7	Smu1	74255	0.616490061	0.03951396	0.16763725

ShSirt1_vs_ShScrambled	Q80TE0	Q80TE0	Rpap1	68925	1.340732572	0.03963342	0.16786141
ShSirt1_vs_ShScrambled	P16092	P16092	Fgfr1	14182	1.070017616	0.03964117	0.16786141
ShSirt1_vs_ShScrambled	Q9R1R2	Q9R1R2	Trim3	55992	-1.594084525	0.03974666	0.16815037
ShSirt1_vs_ShScrambled	Q8K1Z0	Q8K1Z0	Coq9	67914	-0.398866921	0.03991082	0.16868674
ShSirt1_vs_ShScrambled	Q99J56	Q99J56	Derl1	67819	-0.35575236	0.04004827	0.16871382
ShSirt1_vs_ShScrambled	Q8VE73	Q8VE73	Cul7	66515	0.495305669	0.04007117	0.16871382
ShSirt1_vs_ShScrambled	Q6P4T0	Q6P4T0	Atg2a	329015	-0.412718257	0.04012368	0.16871382
ShSirt1_vs_ShScrambled	Q505F1	Q505F1	Nr2c1	22025	1.586081559	0.04013157	0.16871382
ShSirt1_vs_ShScrambled	Q9D1R9	Q9D1R9	Rpl34	68436; 61	0.520346137	0.0401568	0.16871382
ShSirt1_vs_ShScrambled	P50544	P50544	Acadvl	11370	-0.359568322	0.0402059	0.16871382
ShSirt1_vs_ShScrambled	Q9CYH2	Q9CYH2	Fam213a	70564	-0.375400883	0.04022549	0.16871382
ShSirt1_vs_ShScrambled	Q8R146	Q8R146	Apeh	235606	-0.393804156	0.0402663	0.16871382
ShSirt1_vs_ShScrambled	Q8K4L3	Q8K4L3	Svil	225115	-0.340577226	0.04032923	0.16871382
ShSirt1_vs_ShScrambled	P63038	P63038	Hspd1	15510	-0.293881008	0.04033327	0.16871382
ShSirt1_vs_ShScrambled	Q9Z0J0	Q9Z0J0	Npc2	67963	0.684978719	0.04039196	0.16871382
ShSirt1_vs_ShScrambled	P59016	P59016	Vps33b	233405	1.534543518	0.04047511	0.16871382
ShSirt1_vs_ShScrambled	P14069	P14069	S100a6	20200	-0.965514706	0.0404803	0.16871382
ShSirt1_vs_ShScrambled	Q9CRB2	Q9CRB2	Nhp2	52530	0.444164863	0.04048511	0.16871382
ShSirt1_vs_ShScrambled	Q61140	Q61140	Bcar1	12927	-0.405538676	0.04051966	0.16871382
ShSirt1_vs_ShScrambled	Q99K70	Q99K70;Q7T	Rragc		-0.399685756	0.04053172	0.16871382
ShSirt1_vs_ShScrambled	Q8VCM8	Q8VCM8	Ncln	103425	-0.307783099	0.04055261	0.16871382
ShSirt1_vs_ShScrambled	P05132	P05132	Prkaca	18747	-0.480396926	0.04065453	0.16898208
ShSirt1_vs_ShScrambled	Q62376	Q62376	Snrnp70	20637	0.452650109	0.04071223	0.16898576
ShSirt1_vs_ShScrambled	Q4VC33	Q4VC33	Maea	59003	1.140204726	0.04079013	0.16898576
ShSirt1_vs_ShScrambled	Q9CR00	Q9CR00	Psmd9	67151	0.549819647	0.04085008	0.16898576
ShSirt1_vs_ShScrambled	Q3TXU5	Q3TXU5	Dhps	330817	0.689848228	0.04085464	0.16898576
ShSirt1_vs_ShScrambled	P08003	P08003	Pdia4	12304	-0.312246749	0.04087798	0.16898576
ShSirt1_vs_ShScrambled	Q99LH1	Q99LH1	Gnl2	230737	-0.775143743	0.04088003	0.16898576
ShSirt1_vs_ShScrambled	Q9DC48	Q9DC48	Cdc40	71713	1.19497442	0.04101638	0.16939428
ShSirt1_vs_ShScrambled	Q80W54	Q80W54	Zmpste24	230709	-0.464044767	0.04109803	0.16947366
ShSirt1_vs_ShScrambled	Q80TY5	Q80TY5	Vps13b	666173	0.843012874	0.04111069	0.16947366
ShSirt1_vs_ShScrambled	Q8BJS4	Q8BJS4	Sun2	223697	0.435484418	0.0413081	0.17013208
ShSirt1_vs_ShScrambled	P80314	P80314	Cct2	12461	0.381671582	0.04153396	0.17051349
ShSirt1_vs_ShScrambled	Q8R5C5	Q8R5C5	Actr1b	226977	-0.315620472	0.04156941	0.17051349
ShSirt1_vs_ShScrambled	P17225	P17225	Ptbp1		0.339039477	0.04159658	0.17051349
ShSirt1_vs_ShScrambled	Q8VDD8	Q8VDD8	Wash1	68767	0.517394411	0.04160025	0.17051349
ShSirt1_vs_ShScrambled	Q9JHE3	Q9JHE3	Asah2	54447	-1.556081528	0.0416238	0.17051349
ShSirt1_vs_ShScrambled	P70698	P70698	Ctps1	51797	-0.473109423	0.04163196	0.17051349
ShSirt1_vs_ShScrambled	Q02788	Q02788	Col6a2	12834	0.379497404	0.04166513	0.17051349
ShSirt1_vs_ShScrambled	B2RVL6	B2RVL6	Zcchc24	71918	-0.363288296	0.04175386	0.17072187
ShSirt1_vs_ShScrambled	P51655	P51655	Gpc4	14735	2.153532565	0.04185059	0.17090714
ShSirt1_vs_ShScrambled	P61979	P61979	Hnrnpk	15387	0.313071257	0.0418749	0.17090714
ShSirt1_vs_ShScrambled	Q9DCT2	Q9DCT2	Ndufs3	68349	-0.457862915	0.04197451	0.17115894
ShSirt1_vs_ShScrambled	Q9CQ40	Q9CQ40	Mrpl49	18120	-0.476981211	0.04256806	0.17342258
ShSirt1_vs_ShScrambled	Q7TSJ2	Q7TSJ2	Map6	17760	-1.254291288	0.04265147	0.17345176
ShSirt1_vs_ShScrambled	Q6P5G6	Q6P5G6	Ubxn7	224111	0.562980558	0.04265639	0.17345176
ShSirt1_vs_ShScrambled	Q8K135	Q8K135	Kiaa0319l	100317	2.286832591	0.0426905	0.17345176
ShSirt1_vs_ShScrambled	Q6PCM2	Q6PCM2	Ints6	18130	0.626681936	0.0427958	0.17372325
ShSirt1_vs_ShScrambled	Q8BGQ1	Q8BGQ1	Vipas39	104799	-1.398030748	0.04288023	0.17390959
ShSirt1_vs_ShScrambled	Q921M7	Q921M7	Fam49b	223601	-0.535741737	0.04299761	0.17422909
ShSirt1_vs_ShScrambled	P61161	P61161	Actr2	66713	-0.656454873	0.043166	0.17475454
ShSirt1_vs_ShScrambled	Q9Z0V7	Q9Z0V7	Timm17b	21855	0.515594238	0.04333009	0.17526169
ShSirt1_vs_ShScrambled	Q8CHG7	Q8CHG7	Rapgef2		-1.521462752	0.04344827	0.17558236
ShSirt1_vs_ShScrambled	Q3UMC0	Q3UMC0	Spata5	57815	-0.410015719	0.04364372	0.17621443
ShSirt1_vs_ShScrambled	Q3UMF0	Q3UMF0	Cobll1	319876	0.384439069	0.04380931	0.17645134
ShSirt1_vs_ShScrambled	Q62188	Q62188	Dpysl3	22240	-0.506893279	0.04384759	0.17645134
ShSirt1_vs_ShScrambled	Q3U3R4	Q3U3R4	Lmf1	76483	-0.515277884	0.04386173	0.17645134
ShSirt1_vs_ShScrambled	Q921Y2	Q921Y2	Imp3	102462	-0.405977548	0.04386621	0.17645134
ShSirt1_vs_ShScrambled	P97314	P97314	Csrp2	13008	-0.663144201	0.04389784	0.17645134
ShSirt1_vs_ShScrambled	P21958	P21958	Tap1	21354	-1.245659035	0.04399006	0.17666473
ShSirt1_vs_ShScrambled	O35621	O35621	Pmm1	29858	1.510562586	0.04404683	0.17672348
ShSirt1_vs_ShScrambled	P17918	P17918	Pcna	18538	0.475841208	0.04408299	0.17672348
ShSirt1_vs_ShScrambled	Q9CQL0	Q9CQL0	Mettl21A	67099	0.790675297	0.04420551	0.1769218

ShSirt1_vs_ShScrambled	Q3UHH8	Q3UHH8	Gxylt1	223827	-1.507613221	0.04421085	0.1769218
ShSirt1_vs_ShScrambled	Q60597	Q60597	Ogdh	18293	-0.317891498	0.04448841	0.17775441
ShSirt1_vs_ShScrambled	Q11136	Q11136	Pepd	18624	0.354852506	0.04449767	0.17775441
ShSirt1_vs_ShScrambled	O08580	O08580	Esrra	26379	0.427958394	0.04465186	0.17809992
ShSirt1_vs_ShScrambled	Q8R4H2	Q8R4H2	Arhgef12		-1.082337069	0.04469894	0.17809992
ShSirt1_vs_ShScrambled	P26039	P26039	Tln1	21894	-0.296607136	0.04470253	0.17809992
ShSirt1_vs_ShScrambled	O88531	O88531	Ppt1	19063	-0.762981591	0.04485196	0.17845195
ShSirt1_vs_ShScrambled	Q9CQR4	Q9CQR4	Acot13	66834	-0.870416342	0.044883	0.17845195
ShSirt1_vs_ShScrambled	Q8QZS1	Q8QZS1	Hibch	227095	-0.360591889	0.04493587	0.17845195
ShSirt1_vs_ShScrambled	Q8BWM0	Q8BWM0	Ptges2	96979	-0.320174387	0.04494902	0.17845195
ShSirt1_vs_ShScrambled	Q7TPR4	Q7TPR4	Actn1	109711	-0.294958934	0.04503919	0.1786176
ShSirt1_vs_ShScrambled	Q8VCH6	Q8VCH6	Dhcr24	74754	2.641785174	0.04512525	0.1786176
ShSirt1_vs_ShScrambled	Q8C1D8	Q8C1D8	Iws1	73473	0.611102362	0.04514541	0.1786176
ShSirt1_vs_ShScrambled	P47802	P47802	Mtx1		0.356680953	0.04514902	0.1786176
ShSirt1_vs_ShScrambled	Q9CWX2	Q9CWX2	Ndufaf1	69702	-0.40819898	0.04522757	0.17877168
ShSirt1_vs_ShScrambled	P54923	P54923	Adprh	11544	-0.408854804	0.04546693	0.17941252
ShSirt1_vs_ShScrambled	Q9WUP7	Q9WUP7	Uchl5	56207	0.503667227	0.04546919	0.17941252
ShSirt1_vs_ShScrambled	Q8BPG6	Q8BPG6	Sumf2	67902	-0.457495043	0.04564473	0.17994788
ShSirt1_vs_ShScrambled	P46935	P46935	Nedd4	17999	-0.414919859	0.04570646	0.17994889
ShSirt1_vs_ShScrambled	Q9QWR8	Q9QWR8	Naga	17939	-0.624517973	0.04572472	0.17994889
ShSirt1_vs_ShScrambled	Q9WTK5	Q9WTK5	Nfkb2	18034	0.758956472	0.04583586	0.18022915
ShSirt1_vs_ShScrambled	Q7TMY8	Q7TMY8	Huwe1	59026	0.326162935	0.04589373	0.18029967
ShSirt1_vs_ShScrambled	P63005	P63005	Pafah1b1	18472	-0.298874474	0.04599674	0.1805472
ShSirt1_vs_ShScrambled	P97499	P97499	Tep1	21745	-0.449079987	0.04604769	0.18059015
ShSirt1_vs_ShScrambled	Q9EQ06	Q9EQ06	Hsd17b11	114664	-0.574653396	0.04631382	0.18147621
ShSirt1_vs_ShScrambled	Q9WUK4	Q9WUK4	Rfc2	19718	1.71087668	0.04640817	0.1816414
ShSirt1_vs_ShScrambled	P28660	P28660	Nckap1	50884	-0.418034415	0.0464561	0.1816414
ShSirt1_vs_ShScrambled	P62627	P62627	Dynlrb1	67068	-0.495122617	0.04647928	0.1816414
ShSirt1_vs_ShScrambled	O70421	O70421	Fzd1	14362	1.114053341	0.04651694	0.1816414
ShSirt1_vs_ShScrambled	Q9EPL8	Q9EPL8	Ipo7	233726	-0.523591322	0.04660095	0.18171828
ShSirt1_vs_ShScrambled	Q61586	Q61586	Gpam	14732	-0.728627727	0.04661714	0.18171828
ShSirt1_vs_ShScrambled	Q8CFX1	Q8CFX1	H6pd	100198	0.324180424	0.04676937	0.1821173
ShSirt1_vs_ShScrambled	Q99KC8	Q99KC8	Vwa5a	67776	-0.359235293	0.04680019	0.1821173
ShSirt1_vs_ShScrambled	P54818	P54818	Galc	14420	-1.389555395	0.04724312	0.18368254
ShSirt1_vs_ShScrambled	E9PY46	E9PY46	Ift140	106633	1.454560502	0.04731938	0.18375866
ShSirt1_vs_ShScrambled	Q78XF5	Q78XF5	Ostc	66357	0.64269143	0.04734411	0.18375866
ShSirt1_vs_ShScrambled	Q9D2G2	Q9D2G2	Dlst	78920	-0.46723479	0.04745342	0.18402467
ShSirt1_vs_ShScrambled	P11499	P11499	Hsp90ab1	15516	0.286052716	0.04750611	0.18407088
ShSirt1_vs_ShScrambled	Q6P5B0	Q6P5B0	Rrp12	107094	0.400590416	0.04755622	0.184107
ShSirt1_vs_ShScrambled	Q9DCL9	Q9DCL9	Paics	67054	0.297705677	0.04764378	0.18428793
ShSirt1_vs_ShScrambled	P80315	P80315	Cct4	12464	0.280802106	0.04788746	0.18493136
ShSirt1_vs_ShScrambled	Q70E20	Q70E20	Sned1	208777	-1.510013127	0.04789206	0.18493136
ShSirt1_vs_ShScrambled	Q9Z0X1	Q9Z0X1	Aifm1	26926	-0.35746812	0.04803663	0.18517881
ShSirt1_vs_ShScrambled	Q60838	Q60838	Dvl2	13543	2.379367055	0.04803819	0.18517881
ShSirt1_vs_ShScrambled	Q9DBU0	Q9DBU0	Tm9sf1	74140	0.58772792	0.04815035	0.18545281
ShSirt1_vs_ShScrambled	P23116	P23116	Eif3a	13669	0.376572196	0.04836311	0.18611346
ShSirt1_vs_ShScrambled	Q91YT0	Q91YT0	Ndufv1	17995	-0.31885196	0.04842837	0.18620586
ShSirt1_vs_ShScrambled	Q9CRT8	Q9CRT8	Xpot		0.493329742	0.0484914	0.18622798
ShSirt1_vs_ShScrambled	P70398	P70398	Usp9x	22284	0.372746155	0.04854662	0.18622798
ShSirt1_vs_ShScrambled	Q9EP72	Q9EP72	Emc7	73024	-0.294682335	0.0485579	0.18622798
ShSirt1_vs_ShScrambled	Q8BMJ3	Q8BMJ3;Q60	Eif1ax		-0.591203	0.04864388	0.18634135
ShSirt1_vs_ShScrambled	P97807	P97807	Fh	14194	-0.289278415	0.048678	0.18634135
ShSirt1_vs_ShScrambled	Q9D6U8	Q9D6U8	Fam162a	70186	0.72221914	0.0487113	0.18634135
ShSirt1_vs_ShScrambled	Q8BHY2	Q8BHY2	Noc4l	100608	1.266117426	0.04884952	0.18650366
ShSirt1_vs_ShScrambled	Q8BWF0	Q8BWF0	Aldh5a1	214579	1.123772804	0.04888683	0.18650366
ShSirt1_vs_ShScrambled	B1AXP6	B1AXP6	Tomm5	68512	-0.823200406	0.04889062	0.18650366
ShSirt1_vs_ShScrambled	Q9DCE5	Q9DCE5	Pak1ip1	68083	-1.38434391	0.04891899	0.18650366
ShSirt1_vs_ShScrambled	Q99JY0	Q99JY0	Hadhb	231086	-0.360394979	0.04901065	0.18669543
ShSirt1_vs_ShScrambled	Q9D187	Q9D187	Fam96b	68523	0.765088381	0.04927873	0.18755836
ShSirt1_vs_ShScrambled	Q91W89	Q91W89	Man2c1	73744	0.386807099	0.04932103	0.18756119
ShSirt1_vs_ShScrambled	Q8K3A2	Q8K3A2	Trpt1	107328	1.421515862	0.04941837	0.18777318
ShSirt1_vs_ShScrambled	Q9CRC9	Q9CRC9	Gnpda2	67980	-0.348902047	0.04952815	0.18780367
ShSirt1_vs_ShScrambled	Q60676	Q60676	Ppp5c	19060	0.318141713	0.04958147	0.18780367

ShSirt1_vs_ShScrambled	Q61823	Q61823	Pdcd4	18569	0.490090039	0.04961317	0.18780367
ShSirt1_vs_ShScrambled	Q9CQZ6	Q9CQZ6	Ndufb3	66495	-0.499387176	0.04963163	0.18780367
ShSirt1_vs_ShScrambled	Q61733	Q61733	Mrps31	57312	-0.41412278	0.04966619	0.18780367
ShSirt1_vs_ShScrambled	Q9CXZ1	Q9CXZ1	Ndufs4	17993	-0.80367717	0.04967603	0.18780367
ShSirt1_vs_ShScrambled	P81117	P81117	Nucb2	53322	-0.43830496	0.04988918	0.18840591
ShSirt1_vs_ShScrambled	P70399	P70399	Tp53bp1	27223	0.469557792	0.04992979	0.18840591
ShSirt1_vs_ShScrambled	Q5U458	Q5U458	Dnajc11	230935	-0.35276625	0.04998839	0.18840591
ShSirt1_vs_ShScrambled	Q8K1C9	Q8K1C9	Lrrc41	230654	2.151849087	0.05000228	0.18840591
ShSirt1_vs_ShScrambled	Q922Q8	Q922Q8	Lrrc59	98238	0.354287394	0.0500468	0.18841641
ShSirt1_vs_ShScrambled	P40224	P40224	Cxcl12	20315	-1.411346143	0.05009154	0.18842766
ShSirt1_vs_ShScrambled	A2AIL4	A2AIL4	Ndufaf6	76947	0.640319109	0.05014811	0.18848342
ShSirt1_vs_ShScrambled	Q8BHL8	Q8BHL8	Psmf1	228769	-0.407175641	0.05019437	0.188488
ShSirt1_vs_ShScrambled	Q9R0C0	Q9R0C0	Bloc1s6	18457	0.729497398	0.05023285	0.188488
ShSirt1_vs_ShScrambled	Q99LM2	Q99LM2	Cdk5rap3	80280	-0.759334292	0.05028507	0.18852724
ShSirt1_vs_ShScrambled	P32507	P32507	Pvrl2	19294	0.500833349	0.05046934	0.18906109
ShSirt1_vs_ShScrambled	Q8QZY1	Q8QZY1	Eif3l	223691	0.367986082	0.05068649	0.18971709
ShSirt1_vs_ShScrambled	Q62159	Q62159	Rhoc	11853	0.573577411	0.05074758	0.18978838
ShSirt1_vs_ShScrambled	Q8BH58	Q8BH58	Tiprl	226591	0.406673894	0.05095668	0.19041261
ShSirt1_vs_ShScrambled	P61965	P61965	Wdr5	140858	1.715973704	0.05120039	0.19116507
ShSirt1_vs_ShScrambled	Q9QZ73	Q9QZ73	Dcun1d1	114893	-0.761266121	0.05132061	0.19139003
ShSirt1_vs_ShScrambled	Q9WVR4	Q9WVR4	Fxr2		0.361342037	0.05134544	0.19139003
ShSirt1_vs_ShScrambled	P62984	P62984	Uba52	22186	0.742130312	0.05144173	0.19159075
ShSirt1_vs_ShScrambled	Q80U63	Q80U63	Mfn2	170731	-0.461185527	0.05163601	0.19215575
ShSirt1_vs_ShScrambled	Q8BGT7	Q8BGT7	Smndc1	76479	1.38709359	0.05175149	0.19241164
ShSirt1_vs_ShScrambled	Q8R086	Q8R086	Suox	211389	0.487513486	0.05182418	0.19241164
ShSirt1_vs_ShScrambled	Q9JHQ5	Q9JHQ5	Lztfl1	93730	0.542344154	0.05192446	0.19241164
ShSirt1_vs_ShScrambled	Q9QZ23	Q9QZ23	Nfu1	56748	0.413745497	0.05198609	0.19241164
ShSirt1_vs_ShScrambled	Q9WUU7	Q9WUU7	Ctsz	64138	-0.521785931	0.05199712	0.19241164
ShSirt1_vs_ShScrambled	Q9R0I7	Q9R0I7	Ylpm1		0.584964374	0.05200873	0.19241164
ShSirt1_vs_ShScrambled	Q8R3N1	Q8R3N1	Nop14	75416	-0.52441146	0.05200896	0.19241164
ShSirt1_vs_ShScrambled	Q9CVB6	Q9CVB6	Arpc2	76709	-0.298012631	0.05204577	0.19241164
ShSirt1_vs_ShScrambled	Q9CWP6	Q9CWP6	Mospd2	76763	-0.612465336	0.05226375	0.19297633
ShSirt1_vs_ShScrambled	Q7TPV4	Q7TPV4	Mybbp1a	18432	0.384678849	0.05228402	0.19297633
ShSirt1_vs_ShScrambled	P17182	P17182	Eno1	13806; 43	0.668410149	0.05233504	0.19300684
ShSirt1_vs_ShScrambled	Q00915	Q00915	Rbp1	19659	-0.626124927	0.05248851	0.19329097
ShSirt1_vs_ShScrambled	O09012	O09012	Pex5	19305	-0.947339407	0.05249772	0.19329097
ShSirt1_vs_ShScrambled	P70365	P70365	Ncoa1	17977	1.328006942	0.05254244	0.19329793
ShSirt1_vs_ShScrambled	Q8BGF9	Q8BGF9	Slc25a44	229517	-0.660356684	0.05269977	0.19371887
ShSirt1_vs_ShScrambled	Q9D7H3	Q9D7H3	RtcA	66368	0.29857268	0.05304855	0.19435702
ShSirt1_vs_ShScrambled	O35646	O35646	Capn6	12338	-0.392504389	0.05307644	0.19435702
ShSirt1_vs_ShScrambled	Q922Y1	Q922Y1	Ubxn1	225896	0.737768293	0.05308957	0.19435702
ShSirt1_vs_ShScrambled	Q9CZ57	Q9CZ57	Nsun4	72181	0.689063981	0.05324705	0.19435702
ShSirt1_vs_ShScrambled	P58801	P58801	Ripk2	192656	0.783892759	0.0532515	0.19435702
ShSirt1_vs_ShScrambled	Q64442	Q64442	Sord	20322	-0.750551251	0.05329874	0.19435702
ShSirt1_vs_ShScrambled	C0HKG6	C0HKG6;C0I	C0HKG6		-0.744649529	0.05330251	0.19435702
ShSirt1_vs_ShScrambled	Q91YM4	Q91YM4	Tbrg4	21379	-0.277886887	0.05330939	0.19435702
ShSirt1_vs_ShScrambled	P07214	P07214	Sparc	20692	0.392602342	0.05332257	0.19435702
ShSirt1_vs_ShScrambled	Q8CI51	Q8CI51	Pdlim5	56376	-0.427690662	0.0533418	0.19435702
ShSirt1_vs_ShScrambled	Q9CR63	Q9CR63	Cox16	66272	-1.364821126	0.053347	0.19435702
ShSirt1_vs_ShScrambled	Q69ZS7	Q69ZS7	Hbs1l	56422	1.132052545	0.05379609	0.19574652
ShSirt1_vs_ShScrambled	P24452	P24452	Capg		0.411587023	0.05381512	0.19574652
ShSirt1_vs_ShScrambled	Q9Z247	Q9Z247	Fkbp9	27055	-0.341756309	0.05389961	0.19589601
ShSirt1_vs_ShScrambled	Q8BU33	Q8BU33	Ilvbl	216136	-0.335513417	0.05442382	0.1976421
ShSirt1_vs_ShScrambled	P45376	P45376	Akr1b1	11677	0.428142214	0.05447196	0.19765791
ShSirt1_vs_ShScrambled	Q9JK92	Q9JK92	Hspb8	80888	-0.859866061	0.05458672	0.19791521
ShSirt1_vs_ShScrambled	Q9CQJ2	Q9CQJ2	Pih1d1	68845	0.538209299	0.05481067	0.19856611
ShSirt1_vs_ShScrambled	Q91YP3	Q91YP3	Dera	232449	-0.457650077	0.05487697	0.19856611
ShSirt1_vs_ShScrambled	Q9CZU4	Q9CZU4	Eral1	57837	0.475291528	0.05489821	0.19856611
ShSirt1_vs_ShScrambled	Q3UH60	Q3UH60	Dip2b	239667	0.735185819	0.05519357	0.19938134
ShSirt1_vs_ShScrambled	P05480	P05480	Src	20779	0.57227065	0.05521194	0.19938134
ShSirt1_vs_ShScrambled	Q925N2	Q925N2	Sfxn2	94279	-1.159796509	0.05531657	0.19959952
ShSirt1_vs_ShScrambled	Q9CZW4	Q9CZW4	Acsl3	74205	1.169049051	0.05540021	0.19974165
ShSirt1_vs_ShScrambled	Q8BGZ4	Q8BGZ4	Cdc23	52563	0.683917329	0.05545051	0.19976346

ShSirt1_vs_ShScrambled	Q8C163	Q8C163	Exog	208194	-0.793241125	0.05553856	0.1999211
ShSirt1_vs_ShScrambled	Q9D6K8	Q9D6K8	Fundc2	67391	-0.501497956	0.05565287	0.2000149
ShSirt1_vs_ShScrambled	Q9QXA5	Q9QXA5	Lsm4	50783	0.71703812	0.05571939	0.2000149
ShSirt1_vs_ShScrambled	Q9JJY4	Q9JJY4	Ddx20	53975	1.361498118	0.05572235	0.2000149
ShSirt1_vs_ShScrambled	P11031	P11031	Sub1	20024	0.304587509	0.05574186	0.2000149
ShSirt1_vs_ShScrambled	Q8BGD9	Q8BGD9	Eif4b	75705	0.374887663	0.05589811	0.20025904
ShSirt1_vs_ShScrambled	P49817	P49817	Cav1	12389	-0.747757935	0.05589862	0.20025904
ShSirt1_vs_ShScrambled	P00158	P00158	Mt.Cyb	17711	-0.895647953	0.05612832	0.20092246
ShSirt1_vs_ShScrambled	O88851	O88851	Rbbp9	26450	0.516983258	0.05625753	0.20122542
ShSirt1_vs_ShScrambled	Q00420	Q00420	Gabpb1	14391	1.321354499	0.05667244	0.20247339
ShSirt1_vs_ShScrambled	O08529	O08529	Capn2	12334	-0.304016571	0.05670488	0.20247339
ShSirt1_vs_ShScrambled	O54941	O54941	Smarce1	57376	0.9846063	0.05674958	0.20247339
ShSirt1_vs_ShScrambled	Q3V4B5	Q3V4B5	Commd6	66200	-0.707162404	0.05682376	0.20247339
ShSirt1_vs_ShScrambled	Q80WQ2	Q80WQ2	Vac14	234729	-0.492393313	0.0568307	0.20247339
ShSirt1_vs_ShScrambled	Q91V16	Q91V16	Lyrm5	67636	1.29112292	0.05701721	0.20283088
ShSirt1_vs_ShScrambled	Q8JZM0	Q8JZM0	Tfb1m	224481	0.342352775	0.05703606	0.20283088
ShSirt1_vs_ShScrambled	O09159	O09159	Man2b1	17159	-0.359558325	0.05706584	0.20283088
ShSirt1_vs_ShScrambled	Q9ERF3	Q9ERF3	Wdr61	66317	0.385566923	0.0571378	0.20288612
ShSirt1_vs_ShScrambled	Q6TYB5	Q6TYB5	Fez2	225020	-1.024673865	0.05717128	0.20288612
ShSirt1_vs_ShScrambled	P28867	P28867	Prkcd	18753	0.787155595	0.05726601	0.20306266
ShSirt1_vs_ShScrambled	Q9CR76	Q9CR76	Tmem186	66690	1.312414226	0.05739343	0.20335475
ShSirt1_vs_ShScrambled	Q8CHH9	Q8CHH9	Sept8	20362	0.468887808	0.05743963	0.20335881
ShSirt1_vs_ShScrambled	Q8C522	Q8C522	Endod1	71946	-2.14474061	0.05759452	0.20374739
ShSirt1_vs_ShScrambled	Q99J83	Q99J83	Atg5	11793	1.158344699	0.0576427	0.20375814
ShSirt1_vs_ShScrambled	Q8C170	Q8C170	Myo9a	270163	1.53520924	0.0577651	0.20403103
ShSirt1_vs_ShScrambled	Q6DFV5	Q6DFV5	Helz	78455	0.970965502	0.05786551	0.20422588
ShSirt1_vs_ShScrambled	P0DOV1	P0DOV1	P0DOV1	381308	-1.398107901	0.05802337	0.20457854
ShSirt1_vs_ShScrambled	Q3U186	Q3U186	Rars2	109093	-0.506730572	0.05805607	0.20457854
ShSirt1_vs_ShScrambled	O55131	O55131	Sept7	235072	-0.328683583	0.05815112	0.20475364
ShSirt1_vs_ShScrambled	O35295	O35295	Purb	19291	0.298801496	0.05839101	0.20543804
ShSirt1_vs_ShScrambled	Q8BVE3	Q8BVE3	Atp6v1h	108664	-0.308643264	0.0584915	0.20563133
ShSirt1_vs_ShScrambled	Q9QY76	Q9QY76	Vapb		-0.440814527	0.05861831	0.20578829
ShSirt1_vs_ShScrambled	P97823	P97823	Lypla1	18777	-0.469719661	0.05871877	0.20578829
ShSirt1_vs_ShScrambled	Q9Z0P4	Q9Z0P4	Palm	18483	1.130259151	0.05872911	0.20578829
ShSirt1_vs_ShScrambled	Q9EPU0	Q9EPU0	Upf1	19704	-0.278982301	0.05878745	0.20578829
ShSirt1_vs_ShScrambled	Q91V08	Q91V08	Clec2d	93694	-0.610674912	0.05884307	0.20578829
ShSirt1_vs_ShScrambled	Q9EPB5	Q9EPB5	Serhl	68607	0.611713117	0.05888634	0.20578829
ShSirt1_vs_ShScrambled	Q9JL15	Q9JL15	Lgals8	56048	1.294295752	0.05889576	0.20578829
ShSirt1_vs_ShScrambled	Q80UP5	Q80UP5	Ankrd13a	68420	0.518431334	0.05890086	0.20578829
ShSirt1_vs_ShScrambled	Q9D7B6	Q9D7B6	Acad8	66948	-0.296546487	0.05916016	0.2065344
ShSirt1_vs_ShScrambled	Q80U49	Q80U49	Cep170b	217882	1.566450402	0.05924937	0.20664368
ShSirt1_vs_ShScrambled	P61961	P61961	Ufm1	67890	-0.377077164	0.05928302	0.20664368
ShSirt1_vs_ShScrambled	O89086	O89086	Rbm3	19652	-0.619395746	0.05934661	0.2067057
ShSirt1_vs_ShScrambled	Q9CZX9	Q9CZX9	Emc4	68032	-1.014726652	0.05956733	0.20715942
ShSirt1_vs_ShScrambled	Q91V09	Q91V09	Wdr13	73447	0.673884954	0.05956866	0.20715942
ShSirt1_vs_ShScrambled	Q8VEH3	Q8VEH3	Arl8a	68724	-1.051077004	0.05981023	0.20720998
ShSirt1_vs_ShScrambled	P11531	P11531	Dmd	13405	0.763904814	0.05982434	0.20720998
ShSirt1_vs_ShScrambled	O09117	O09117	Sypl1	19027	0.488810578	0.05985171	0.20720998
ShSirt1_vs_ShScrambled	Q3TC33	Q3TC33	Ccdc127	67433	0.873069912	0.05989125	0.20720998
ShSirt1_vs_ShScrambled	Q8R010	Q8R010	Aimp2	231872	0.335281477	0.05992777	0.20720998
ShSirt1_vs_ShScrambled	Q91VE6	Q91VE6	Nifk	67949	0.602367824	0.05994137	0.20720998
ShSirt1_vs_ShScrambled	Q8VEH6	Q8VEH6	Cbwd1	226043	0.602473166	0.05998842	0.20720998
ShSirt1_vs_ShScrambled	P30285	P30285	Cdk4	12567	0.370966214	0.05999663	0.20720998
ShSirt1_vs_ShScrambled	Q99KK1	Q99KK1	Reep3	28193	-0.989983019	0.06008024	0.20720998
ShSirt1_vs_ShScrambled	Q922R8	Q922R8	Pdia6	71853	-0.272314924	0.06008289	0.20720998
ShSirt1_vs_ShScrambled	Q5RL79	Q5RL79	Krtcap2	66059	-0.705779667	0.06008814	0.20720998
ShSirt1_vs_ShScrambled	Q9JLV5	Q9JLV5	Cul3	26554	0.272639488	0.06016823	0.20725903
ShSirt1_vs_ShScrambled	Q9D7S7	Q9D7S7	Rpl22l1	68028	-0.955127073	0.06019419	0.20725903
ShSirt1_vs_ShScrambled	Q8K2K6	Q8K2K6	Agfg1	15463	1.07169028	0.06028433	0.20741119
ShSirt1_vs_ShScrambled	P62309	P62309	Snrpg	68011	1.185402928	0.06047955	0.20792436
ShSirt1_vs_ShScrambled	Q62086	Q62086	Pon2	330260	0.279141044	0.06092134	0.20928382
ShSirt1_vs_ShScrambled	Q3UN02	Q3UN02	Lclat1	225010	0.891252801	0.06121544	0.21013423
ShSirt1_vs_ShScrambled	Q923G2	Q923G2	Polr2h	245841	0.351135387	0.06144402	0.2107586

ShSirt1_vs_ShScrambled	Q69Z38	Q69Z38	Peak1	244895	-0.667732507	0.06151074	0.21082723
ShSirt1_vs_ShScrambled	Q9D1M0	Q9D1M0	Sec13	110379	0.393139828	0.06157639	0.21089212
ShSirt1_vs_ShScrambled	Q9D2V7	Q9D2V7	Coro7	78885	0.448885132	0.06166482	0.21103364
ShSirt1_vs_ShScrambled	Q02053	Q02053	Uba1	22201	-0.282842856	0.06171121	0.21103364
ShSirt1_vs_ShScrambled	P54227	P54227	Stmn1	16765	0.930281734	0.06196656	0.21174646
ShSirt1_vs_ShScrambled	Q9WV86	Q9WV86	Katna1		-0.704267456	0.06213142	0.2121492
ShSirt1_vs_ShScrambled	Q91YR5	Q91YR5	Mettl13	71449	1.256040781	0.06225963	0.21233579
ShSirt1_vs_ShScrambled	Q80SW1	Q80SW1	Ahcyl1	229709	0.431579799	0.06228015	0.21233579
ShSirt1_vs_ShScrambled	Q9DB20	Q9DB20	Atp5o	28080	-0.392436799	0.06237023	0.21237424
ShSirt1_vs_ShScrambled	Q03265	Q03265	Atp5a1	11946	-0.324456692	0.06238552	0.21237424
ShSirt1_vs_ShScrambled	O55023	O55023	Impa1		0.740373524	0.06248862	0.21242262
ShSirt1_vs_ShScrambled	Q8BTW9	Q8BTW9	Pak4	70584	0.714910336	0.06250245	0.21242262
ShSirt1_vs_ShScrambled	Q9JIS8	Q9JIS8	Slc12a4	20498	0.404351706	0.06254091	0.21242262
ShSirt1_vs_ShScrambled	Q9DCZ1	Q9DCZ1	Gmpr	66355	-1.252080438	0.06262358	0.21254347
ShSirt1_vs_ShScrambled	Q8K0P3	Q8K0P3	Tldc1	74347	1.250702692	0.0627509	0.21270448
ShSirt1_vs_ShScrambled	Q8BY02	Q8BY02	Nkrf	77286	-1.844199002	0.06276526	0.21270448
ShSirt1_vs_ShScrambled	Q9D662	Q9D662	Sec23b	27054	0.555181969	0.06284962	0.21283061
ShSirt1_vs_ShScrambled	Q9CQN3	Q9CQN3	Tomm6	66119	-1.248873891	0.06292049	0.2128375
ShSirt1_vs_ShScrambled	Q9R0U0	Q9R0U0	Srsf10	14105	0.510973393	0.06294596	0.2128375
ShSirt1_vs_ShScrambled	Q6ZQ38	Q6ZQ38	Cand1	71902	0.280003662	0.06331435	0.21383105
ShSirt1_vs_ShScrambled	Q9R1P1	Q9R1P1	Psmb3	26446	0.306255097	0.06333454	0.21383105
ShSirt1_vs_ShScrambled	Q6P4T2	Q6P4T2	Snrnp200	320632	0.348949958	0.06366466	0.21473342
ShSirt1_vs_ShScrambled	P51855	P51855	Gss	14854	0.340739408	0.06369695	0.21473342
ShSirt1_vs_ShScrambled	P70388	P70388	Rad50		0.683410296	0.06380604	0.21494065
ShSirt1_vs_ShScrambled	Q99KI3	Q99KI3	Emc3	66087	-0.475109027	0.06428963	0.2164082
ShSirt1_vs_ShScrambled	Q571H0	Q571H0	Urb1	207932	0.565562357	0.06436769	0.21650951
ShSirt1_vs_ShScrambled	Q99NB8	Q99NB8	Ubqln4	94232	0.394101687	0.0646671	0.21735464
ShSirt1_vs_ShScrambled	P51150	P51150	Rab7a	19349	-0.388970108	0.06492267	0.2180513
ShSirt1_vs_ShScrambled	Q61112	Q61112	Sdf4	20318	-0.396478309	0.06504203	0.21828976
ShSirt1_vs_ShScrambled	Q80U44	Q80U44	Zfyve16	218441	-0.722534758	0.06514876	0.21848553
ShSirt1_vs_ShScrambled	P52875	P52875	Tmem165	21982	-0.488910308	0.06533231	0.21880064
ShSirt1_vs_ShScrambled	Q9ER72	Q9ER72	Cars	27267	0.326788787	0.06540426	0.21880064
ShSirt1_vs_ShScrambled	Q8BNU0	Q8BNU0	Armc6	76813	-0.559019407	0.06543034	0.21880064
ShSirt1_vs_ShScrambled	Q9Z120	Q9Z120	Mettl1	17299	1.222547724	0.06543661	0.21880064
ShSirt1_vs_ShScrambled	Q7TN98	Q7TN98	Cpeb4	67579	0.680135929	0.06561246	0.21922623
ShSirt1_vs_ShScrambled	P97494	P97494	Gclc	14629	0.754503065	0.06585338	0.21986846
ShSirt1_vs_ShScrambled	Q91WK1	Q91WK1	Spryd4	66701	0.450431555	0.06602667	0.22025084
ShSirt1_vs_ShScrambled	P28063	P28063	Psmb8	16913	-1.491204844	0.06610872	0.22025084
ShSirt1_vs_ShScrambled	Q9EPK7	Q9EPK7	Xpo7	65246	0.53056849	0.06611514	0.22025084
ShSirt1_vs_ShScrambled	Q64521	Q64521	Gpd2	14571	-0.264153658	0.06616308	0.22025084
ShSirt1_vs_ShScrambled	O70252	O70252	Hmox2	15369	0.320195352	0.06623077	0.22031369
ShSirt1_vs_ShScrambled	Q9JK38	Q9JK38	Gnpnat1	54342	0.424575184	0.06636628	0.22060189
ShSirt1_vs_ShScrambled	Q9CY62	Q9CY62	Rnf181	66510	-0.833657832	0.06651665	0.22080764
ShSirt1_vs_ShScrambled	P57780	P57780	Actn4	60595	-0.263650694	0.06652601	0.22080764
ShSirt1_vs_ShScrambled	Q3U0J8	Q3U0J8	Tbc1d2b	67016	-0.737466757	0.06664715	0.22104719
ShSirt1_vs_ShScrambled	Q3UM29	Q3UM29	Cog7	233824	0.354666451	0.06683527	0.22142778
ShSirt1_vs_ShScrambled	Q61211	Q61211	Eif2d	16865	0.774505545	0.06689572	0.22142778
ShSirt1_vs_ShScrambled	Q5SFM8	Q5SFM8	Rbm27	225432	-0.610984094	0.06690906	0.22142778
ShSirt1_vs_ShScrambled	Q68FE6	Q68FE6	Fam65a	75687	-0.542898887	0.06698948	0.22153151
ShSirt1_vs_ShScrambled	P26645	P26645	Marcks	17118	-0.535219637	0.06715613	0.22192005
ShSirt1_vs_ShScrambled	Q61464	Q61464	Znf638	18139	0.47034561	0.06725641	0.22204533
ShSirt1_vs_ShScrambled	Q9CS42	Q9CS42	Prps2	110639	0.666018473	0.06729243	0.22204533
ShSirt1_vs_ShScrambled	Q5XG73	Q5XG73	Acbd5	74159	-1.225986554	0.06766703	0.22280344
ShSirt1_vs_ShScrambled	O35387	O35387	Hax1	23897	0.3119729	0.0676906	0.22280344
ShSirt1_vs_ShScrambled	Q91VW3	Q91VW3	Sh3bgrl3	73723	-0.542566714	0.06771019	0.22280344
ShSirt1_vs_ShScrambled	Q99LL5	Q99LL5	Pwp1	103136	0.979059544	0.06772366	0.22280344
ShSirt1_vs_ShScrambled	Q922Q1	Q922Q1	Marc2	67247	-0.384326122	0.06777809	0.22280344
ShSirt1_vs_ShScrambled	Q8BKZ9	Q8BKZ9	Pdhx	27402	-0.353853683	0.06784285	0.22280344
ShSirt1_vs_ShScrambled	P54276	P54276	Msh6	17688	0.563110963	0.0678879	0.22280344
ShSirt1_vs_ShScrambled	Q9JI39	Q9JI39	Abcb10	56199	4.133566354	0.06793518	0.22280344
ShSirt1_vs_ShScrambled	P15379	P15379	Cd44	12505	-0.659572021	0.0679664	0.22280344
ShSirt1_vs_ShScrambled	Q8VCL2	Q8VCL2	Sco2	1E+08	-1.148628607	0.06830679	0.22370319
ShSirt1_vs_ShScrambled	Q8CBE3	Q8CBE3	Wdr37	207615	0.674404654	0.06857158	0.22370319

ShSirt1_vs_ShScrambled	Q61103	Q61103	Dpf2	19708	0.433978918	0.06862343	0.22370319
ShSirt1_vs_ShScrambled	Q9D0I9	Q9D0I9	Rars	104458	0.256228275	0.06862956	0.22370319
ShSirt1_vs_ShScrambled	Q8CG50	Q8CG50	Rab43	69834	1.191140301	0.06863174	0.22370319
ShSirt1_vs_ShScrambled	Q923D5	Q923D5	Wbp11	60321	0.378686234	0.06864234	0.22370319
ShSirt1_vs_ShScrambled	O08912	O08912	Galnt1	14423	-2.047881238	0.06865381	0.22370319
ShSirt1_vs_ShScrambled	O08599	O08599	Stxbp1	20910	0.701339453	0.06867505	0.22370319
ShSirt1_vs_ShScrambled	Q91YS8	Q91YS8	Camk1	52163	0.508384939	0.06868689	0.22370319
ShSirt1_vs_ShScrambled	Q8BVY0	Q8BVY0	Rsl1d1	66409	-0.501094302	0.06875885	0.22377609
ShSirt1_vs_ShScrambled	Q8JZQ9	Q8JZQ9	Eif3b	27979	0.269272491	0.06883354	0.22385778
ShSirt1_vs_ShScrambled	Q06185	Q06185	Atp5i	11958	-0.342255804	0.06922885	0.2248447
ShSirt1_vs_ShScrambled	P62259	P62259	Ywhae	22627	0.4999517	0.06928154	0.2248447
ShSirt1_vs_ShScrambled	P57746	P57746	Atp6v1d	73834	-0.501672333	0.06932706	0.2248447
ShSirt1_vs_ShScrambled	Q3UVK0	Q3UVK0	Ermp1	226090	-0.297091166	0.06933625	0.2248447
ShSirt1_vs_ShScrambled	Q9QYJ3	Q9QYJ3	Dnajb1	81489	0.300892773	0.06945827	0.22491811
ShSirt1_vs_ShScrambled	Q8R4X3	Q8R4X3	Rbm12	75710	0.268899469	0.06953788	0.22491811
ShSirt1_vs_ShScrambled	O08715	O08715	Akap1	11640	-0.57570054	0.06955057	0.22491811
ShSirt1_vs_ShScrambled	Q8CE96	Q8CE96	Trmt6	66926	1.369399651	0.06955819	0.22491811
ShSirt1_vs_ShScrambled	P70362	P70362	Ufd1l	22230	-0.604877473	0.06967704	0.22514111
ShSirt1_vs_ShScrambled	Q9QZX7	Q9QZX7	Srr	27364	0.497900028	0.06978436	0.22532661
ShSirt1_vs_ShScrambled	P40201	P40201	Chd1	12648	1.648076203	0.06985524	0.22539424
ShSirt1_vs_ShScrambled	Q8BTE5	Q8BTE5	Cebpzos	68554	-0.667370558	0.06997959	0.2256342
ShSirt1_vs_ShScrambled	Q9JI78	Q9JI78	Ngly1	59007	-0.936749505	0.0701743	0.22602446
ShSirt1_vs_ShScrambled	P0CG14	P0CG14	Chtf8	214987	0.756149524	0.07022019	0.22602446
ShSirt1_vs_ShScrambled	Q9D1M7	Q9D1M7	Fkbp11	66120	1.652023308	0.07025084	0.22602446
ShSirt1_vs_ShScrambled	Q9WV91	Q9WV91	Ptgfrn	19221	-1.666940154	0.07033352	0.22612928
ShSirt1_vs_ShScrambled	B2RY56	B2RY56	Rbm25	67039	0.389067756	0.0705752	0.2267448
ShSirt1_vs_ShScrambled	Q8VHH5	Q8VHH5	Agap3		0.609392045	0.07063009	0.22675975
ShSirt1_vs_ShScrambled	Q62433	Q62433	Ndrg1	17988	1.239170304	0.07073354	0.22693049
ShSirt1_vs_ShScrambled	P20352	P20352	F3	14066	0.807831719	0.07098723	0.22745948
ShSirt1_vs_ShScrambled	Q9JM76	Q9JM76	Arpc3	56378	-0.273324326	0.07099921	0.22745948
ShSirt1_vs_ShScrambled	Q80X71	Q80X71	Tmem106b	71900	-0.41642729	0.07116735	0.22783648
ShSirt1_vs_ShScrambled	O35969	O35969	Gamt	14431	0.379532072	0.07123939	0.22790548
ShSirt1_vs_ShScrambled	Q9CZJ2	Q9CZJ2	Hspa12b	72630	-0.680011371	0.07130624	0.22795777
ShSirt1_vs_ShScrambled	P59266	P59266	Fitm2	228859	-0.877255887	0.07137277	0.22800898
ShSirt1_vs_ShScrambled	Q99KH8	Q99KH8	Stk24	223255	1.032472953	0.07170971	0.22866341
ShSirt1_vs_ShScrambled	D3Z7P3	D3Z7P3	Gls	14660	0.612415044	0.07173688	0.22866341
ShSirt1_vs_ShScrambled	Q501J6	Q501J6	Ddx17	67040	0.365240266	0.07178088	0.22866341
ShSirt1_vs_ShScrambled	Q61696	Q61696;P17	Hspa1a		0.353342881	0.07178187	0.22866341
ShSirt1_vs_ShScrambled	Q9D1L9	Q9D1L9	Lamtor5	68576	-0.566980589	0.07183091	0.22866341
ShSirt1_vs_ShScrambled	Q8K2F8	Q8K2F8	Lsm14a	67070	0.772701537	0.07192047	0.22878718
ShSirt1_vs_ShScrambled	Q9QYC7	Q9QYC7	Ggcx	56316	-0.637012209	0.07199729	0.22887027
ShSirt1_vs_ShScrambled	Q9D7J9	Q9D7J9	Echdc3	67856	-0.31513182	0.07205766	0.22890097
ShSirt1_vs_ShScrambled	Q8BKI2	Q8BKI2	Tnrc6b	213988	-0.629646067	0.07212063	0.22893988
ShSirt1_vs_ShScrambled	Q9JIX8	Q9JIX8	Acin1	56215	0.349005536	0.07220908	0.22894155
ShSirt1_vs_ShScrambled	Q8BJY1	Q8BJY1	Psmd5	66998	-0.316722556	0.07222259	0.22894155
ShSirt1_vs_ShScrambled	Q9CQ86	Q9CQ86	Mien1	103742	0.66778637	0.07239956	0.22924779
ShSirt1_vs_ShScrambled	P63280	P63280	Ube2i	22196	0.566070854	0.07242077	0.22924779
ShSirt1_vs_ShScrambled	P0C7N9	P0C7N9	Psmg4	69666	0.47941279	0.07251959	0.22926286
ShSirt1_vs_ShScrambled	Q9CR68	Q9CR68	Uqcrfs1	66694	-0.352845784	0.07252711	0.22926286
ShSirt1_vs_ShScrambled	Q09XV5	Q09XV5	Chd8	67772	0.565545828	0.07267814	0.22948272
ShSirt1_vs_ShScrambled	Q60996	Q60996	Ppp2r5c	26931	0.814844878	0.07272475	0.22948272
ShSirt1_vs_ShScrambled	P28658	P28658	Atxn10	54138	0.563252014	0.07274917	0.22948272
ShSirt1_vs_ShScrambled	Q9CPS6	Q9CPS6	Hint3	66847	-0.524648272	0.07284193	0.22961487
ShSirt1_vs_ShScrambled	P41731	P41731	Cd63	12512	0.566578648	0.07308333	0.23021502
ShSirt1_vs_ShScrambled	Q8C4V1	Q8C4V1	Arhgap24	231532	0.811364859	0.07321659	0.23047398
ShSirt1_vs_ShScrambled	Q9Z0F8	Q9Z0F8	Adam17	11491	-0.639309837	0.07339153	0.23081345
ShSirt1_vs_ShScrambled	O08583	O08583	Alyref	21681	0.729126974	0.07343447	0.23081345
ShSirt1_vs_ShScrambled	Q8BZQ7	Q8BZQ7	Anapc2	99152	0.404980448	0.07347783	0.23081345
ShSirt1_vs_ShScrambled	Q8BPE4	Q8BPE4	Tmem177	66343	1.146046159	0.07362422	0.23101183
ShSirt1_vs_ShScrambled	Q8BQ47	Q8BQ47	Cnpy4	66455	2.087654352	0.07364334	0.23101183
ShSirt1_vs_ShScrambled	P30681	P30681	Hmgb2	97165	0.748553816	0.07376574	0.2312351
ShSirt1_vs_ShScrambled	Q3UFY8	Q3UFY8	Trmt10c	52575	0.628400092	0.07393349	0.23146675
ShSirt1_vs_ShScrambled	Q9QZD4	Q9QZD4	Ercc4	50505	-1.290073032	0.07394219	0.23146675

ShSirt1_vs_ShScrambled	Q64324	Q64324	Stxbp2	20911	0.494124905	0.07426116	0.23230415
ShSirt1_vs_ShScrambled	Q9DB34	Q9DB34	Chmp2a	68953	0.821192592	0.07477211	0.23370677
ShSirt1_vs_ShScrambled	Q91XB7	Q91XB7	Yif1a	68090	0.538574996	0.07484693	0.23370677
ShSirt1_vs_ShScrambled	Q9CY58	Q9CY58	Serbp1	66870	0.312816664	0.07486486	0.23370677
ShSirt1_vs_ShScrambled	Q9DBG7	Q9DBG7	Srpr	67398	0.449619075	0.07513202	0.23427797
ShSirt1_vs_ShScrambled	P32883	P32883	Kras	16653	-0.806912642	0.07515164	0.23427797
ShSirt1_vs_ShScrambled	Q6Y685	Q6Y685	Tacc1	320165	-1.131904805	0.07529693	0.23456892
ShSirt1_vs_ShScrambled	P14576	P14576	Srp54	24067; 66	0.534149231	0.07558032	0.23527059
ShSirt1_vs_ShScrambled	P62305	P62305	Snrpe	20643	0.281603593	0.07563219	0.23527059
ShSirt1_vs_ShScrambled	Q3UEB3	Q3UEB3	Puf60	67959	0.27986004	0.07567853	0.23527059
ShSirt1_vs_ShScrambled	Q91VD9	Q91VD9	Ndufs1	227197	-0.290776875	0.07590189	0.23563453
ShSirt1_vs_ShScrambled	P11103	P11103	Parp1		0.342738794	0.07593307	0.23563453
ShSirt1_vs_ShScrambled	Q8CI33	Q8CI33	Cwf19l1	72502	0.794284385	0.07598634	0.23563453
ShSirt1_vs_ShScrambled	Q9Z2L7	Q9Z2L7	Crlf3	54394	0.890170058	0.0760044	0.23563453
ShSirt1_vs_ShScrambled	Q00493	Q00493	Cpe	12876	-1.125123783	0.07611834	0.23578056
ShSirt1_vs_ShScrambled	Q8BHC4	Q8BHC4	Dcakd	68087	0.604985073	0.07616684	0.23578056
ShSirt1_vs_ShScrambled	Q6PDQ2	Q6PDQ2	Chd4	107932	0.309652975	0.07620821	0.23578056
ShSirt1_vs_ShScrambled	Q75N73	Q75N73	Slc39a14	213053	0.414154657	0.07637838	0.2361452
ShSirt1_vs_ShScrambled	Q3V1L4	Q3V1L4	Nt5c2	76952	0.275637159	0.07645444	0.23621859
ShSirt1_vs_ShScrambled	O88322	O88322	Nid2	18074	1.202611025	0.07663396	0.23661129
ShSirt1_vs_ShScrambled	Q8CI95	Q8CI95	Osbpl11		-0.397296957	0.07688697	0.23723019
ShSirt1_vs_ShScrambled	Q9CRD0	Q9CRD0	Ociad1	68095	-0.445144192	0.07703741	0.237532
ShSirt1_vs_ShScrambled	Q8BHB4	Q8BHB4	Wdr3	269470	0.561614206	0.07714105	0.23753611
ShSirt1_vs_ShScrambled	Q62384	Q62384	Zpr1	22687	1.513470462	0.07717356	0.23753611
ShSirt1_vs_ShScrambled	Q3UBX0	Q3UBX0	Tmem109	68539	-0.375283596	0.0771966	0.23753611
ShSirt1_vs_ShScrambled	Q3USZ8	Q3USZ8	Q3USZ8	68861	1.115914578	0.07725451	0.23755235
ShSirt1_vs_ShScrambled	Q8CJG0	Q8CJG0	Ago2	239528	0.337728107	0.07741286	0.23777978
ShSirt1_vs_ShScrambled	Q80UU2	Q80UU2	Rpp38		-0.683477751	0.07743382	0.23777978
ShSirt1_vs_ShScrambled	Q8BYU6	Q8BYU6	Tor1aip2	240832	-0.422950808	0.07781367	0.23847603
ShSirt1_vs_ShScrambled	Q99K41	Q99K41	Emilin1	100952	0.279802276	0.07789856	0.23847603
ShSirt1_vs_ShScrambled	Q99N85	Q99N85	Mrps18a	68565	1.110676718	0.07791154	0.23847603
ShSirt1_vs_ShScrambled	P12787	P12787	Cox5a	12858	-0.590699858	0.07792513	0.23847603
ShSirt1_vs_ShScrambled	P56382	P56382	Atp5e	67126	-0.445769293	0.07797888	0.23847603
ShSirt1_vs_ShScrambled	O88746	O88746	Tom1	21968	-0.374662444	0.07802213	0.23847603
ShSirt1_vs_ShScrambled	Q6RI63	Q6RI63	Fam120b	67544	-1.213536842	0.07807211	0.23847603
ShSirt1_vs_ShScrambled	Q8K1E0	Q8K1E0	Stx5	56389	0.46064888	0.0780832	0.23847603
ShSirt1_vs_ShScrambled	Q8K012	Q8K012	Fnbp1l		1.414239266	0.07853089	0.23956314
ShSirt1_vs_ShScrambled	Q9D071	Q9D071	Mms19	72199	0.435624155	0.07854529	0.23956314
ShSirt1_vs_ShScrambled	Q8VCS6	Q8VCS6	Med9	192191	-1.104736198	0.07866641	0.23972399
ShSirt1_vs_ShScrambled	Q6A028	Q6A028	Swap70	20947	-1.042020453	0.07870424	0.23972399
ShSirt1_vs_ShScrambled	P18654	P18654	Rps6ka3	110651	0.419575656	0.07882336	0.2399249
ShSirt1_vs_ShScrambled	Q3UFY7	Q3UFY7	Nt5c3b	68106	1.184524533	0.07904869	0.24044865
ShSirt1_vs_ShScrambled	Q91YR1	Q91YR1	Twf1	19230	-0.327631088	0.07915529	0.24048647
ShSirt1_vs_ShScrambled	Q9WVG6	Q9WVG6	Carm1	59035	0.621101396	0.07916768	0.24048647
ShSirt1_vs_ShScrambled	Q8BWW9	Q8BWW9	Pkn2	109333	-0.5044425	0.07943623	0.24113999
ShSirt1_vs_ShScrambled	Q9ER38	Q9ER38	Tor3a	30935	1.096262834	0.07976128	0.24194233
ShSirt1_vs_ShScrambled	P30115	P30115;P137	Gsta3		-2.821502525	0.07980774	0.24194233
ShSirt1_vs_ShScrambled	Q9D4H1	Q9D4H1	Exoc2	66482	0.275601602	0.0802571	0.24302854
ShSirt1_vs_ShScrambled	P09103	P09103	P4hb	18453	0.263453691	0.08027372	0.24302854
ShSirt1_vs_ShScrambled	P59672	P59672	Anks1a	224650	0.348355523	0.08052718	0.24363249
ShSirt1_vs_ShScrambled	Q99104	Q99104	Myo5a	17918	-0.457127031	0.08062628	0.24376893
ShSirt1_vs_ShScrambled	Q8BYH7	Q8BYH7	Tbc1d17	233204	-0.431620994	0.08077099	0.24400994
ShSirt1_vs_ShScrambled	Q61543	Q61543	Glg1	20340	-0.397912706	0.08083603	0.24400994
ShSirt1_vs_ShScrambled	Q7TSQ8	Q7TSQ8	Pdpr	319518	-1.071470799	0.0809026	0.24400994
ShSirt1_vs_ShScrambled	Q9CWU9	Q9CWU9	Nup37	69736	0.443919291	0.08095762	0.24400994
ShSirt1_vs_ShScrambled	Q9D0L7	Q9D0L7	Armc10	67211	0.327130991	0.08097627	0.24400994
ShSirt1_vs_ShScrambled	Q9Z0V8	Q9Z0V8	Timm17a	21854	-0.742064955	0.08105144	0.24407351
ShSirt1_vs_ShScrambled	P03958	P03958	Ada	11486	1.083072653	0.08150931	0.24518346
ShSirt1_vs_ShScrambled	Q91WK0	Q91WK0	Lrrfip2	71268	-0.312323595	0.0815715	0.24518346
ShSirt1_vs_ShScrambled	O70133	O70133	Dhx9	13211	0.260416079	0.08165134	0.24518346
ShSirt1_vs_ShScrambled	E9Q394	E9Q394	Akap13	75547	0.445204613	0.08166368	0.24518346
ShSirt1_vs_ShScrambled	P97825	P97825	Hn1	15374	-0.958384278	0.08169161	0.24518346
ShSirt1_vs_ShScrambled	Q60973	Q60973	Rbbp7	245688	0.564645928	0.08188477	0.24559992

ShSirt1_vs_ShScrambled	Q8R0X7	Q8R0X7	Sgpl1	20397	0.327725786	0.08198356	0.24573293
ShSirt1_vs_ShScrambled	Q8BHN3	Q8BHN3	Ganab	14376	0.24546514	0.08227245	0.24614259
ShSirt1_vs_ShScrambled	Q8BG05	Q8BG05	Hnrnpa3	229279	0.253638678	0.08228074	0.24614259
ShSirt1_vs_ShScrambled	O35074	O35074	Ptgis	19223	0.432275026	0.08233191	0.24614259
ShSirt1_vs_ShScrambled	P54103	P54103	Dnajc2	22791	0.559091438	0.0825214	0.24614259
ShSirt1_vs_ShScrambled	P29758	P29758	Oat	18242	0.293590547	0.08253494	0.24614259
ShSirt1_vs_ShScrambled	Q9Z127	Q9Z127	Slc7a5	20539	-0.519500672	0.08254797	0.24614259
ShSirt1_vs_ShScrambled	Q99P88	Q99P88	Nup155	170762	-0.57575911	0.08255287	0.24614259
ShSirt1_vs_ShScrambled	Q3TLP5	Q3TLP5	Echdc2	52430	-0.243755727	0.08255646	0.24614259
ShSirt1_vs_ShScrambled	Q8BLY2	Q8BLY2	Tarsl2	272396	1.17354433	0.08270716	0.24642913
ShSirt1_vs_ShScrambled	Q8BFV2	Q8BFV2	Pcid2	234069	-0.424509683	0.08290504	0.2465931
ShSirt1_vs_ShScrambled	Q99J93	Q99J93	Ifitm2	80876	-0.555195588	0.08290706	0.2465931
ShSirt1_vs_ShScrambled	Q8R409	Q8R409	Hexim1	192231	-0.290477124	0.08296651	0.2465931
ShSirt1_vs_ShScrambled	O88508	O88508	Dnmt3a	13435	0.405130394	0.08298071	0.2465931
ShSirt1_vs_ShScrambled	Q9D1B9	Q9D1B9	Mrpl28	68611	-0.457895829	0.08312109	0.24669772
ShSirt1_vs_ShScrambled	P35700	P35700	Prdx1	18477	-0.324743806	0.083135	0.24669772
ShSirt1_vs_ShScrambled	Q8BIJ7	Q8BIJ7	Rufy1	216724	-0.466580392	0.08317987	0.24669772
ShSirt1_vs_ShScrambled	Q9CQQ7	Q9CQQ7	Atp5f1	11950	-0.41067823	0.08340275	0.24719633
ShSirt1_vs_ShScrambled	Q8C0I1	Q8C0I1	Agps	228061	0.365622712	0.0834951	0.24720418
ShSirt1_vs_ShScrambled	Q80WS3	Q80WS3	Fbll1	237730	0.420196898	0.08351493	0.24720418
ShSirt1_vs_ShScrambled	Q9CQC8	Q9CQC8	Spg21	27965	-0.519860601	0.08372133	0.24764301
ShSirt1_vs_ShScrambled	Q9Z2D0	Q9Z2D0	Mtmr9	210376	-0.952616286	0.08381274	0.24764301
ShSirt1_vs_ShScrambled	Q9DB43	Q9DB43	Zfpl1	81909	1.066186686	0.08382776	0.24764301
ShSirt1_vs_ShScrambled	Q9EST5	Q9EST5	Anp32b	67628	0.572636585	0.08408711	0.24814347
ShSirt1_vs_ShScrambled	Q8VDS8	Q8VDS8	Stx18	71116	-0.528717896	0.08410711	0.24814347
ShSirt1_vs_ShScrambled	Q8CFD4	Q8CFD4	Snx8	231834	0.838714018	0.08429484	0.24841044
ShSirt1_vs_ShScrambled	Q8BSQ9	Q8BSQ9	Pbrm1		1.309471391	0.08430766	0.24841044
ShSirt1_vs_ShScrambled	Q9CY66	Q9CY66	Gar1	68147	0.638487954	0.08441719	0.24857089
ShSirt1_vs_ShScrambled	Q922D4	Q922D4	Ppp6r3	52036	0.293487431	0.08459755	0.24883875
ShSirt1_vs_ShScrambled	P35486	P35486	Pdha1	18597	-0.245572289	0.08467947	0.24883875
ShSirt1_vs_ShScrambled	Q8BX90	Q8BX90	Fndc3a	319448	1.059613569	0.08475564	0.24883875
ShSirt1_vs_ShScrambled	Q3THK3	Q3THK3	Gtf2f1	98053	0.321503993	0.08488324	0.24883875
ShSirt1_vs_ShScrambled	Q3TKT4	Q3TKT4	Smarca4	20586	0.325813377	0.08491876	0.24883875
ShSirt1_vs_ShScrambled	P97346	P97346	Nxn	18230	0.343786144	0.08492127	0.24883875
ShSirt1_vs_ShScrambled	O88492	O88492	Plin4	57435	0.357184538	0.0849434	0.24883875
ShSirt1_vs_ShScrambled	P41230	P41230	Kdm5c	20591	1.058255563	0.08494916	0.24883875
ShSirt1_vs_ShScrambled	P26450	P26450	Pik3r1	18708	-0.467769485	0.08503358	0.2489245
ShSirt1_vs_ShScrambled	Q9DB73	Q9DB73	Cyb5r1	72017	0.425150205	0.08518432	0.24909134
ShSirt1_vs_ShScrambled	A2RSY6	A2RSY6	Trmt1l	98685	-0.740396734	0.08525498	0.24909134
ShSirt1_vs_ShScrambled	Q8BZB2	Q8BZB2	Ppcdc	66812	-1.05611049	0.08525612	0.24909134
ShSirt1_vs_ShScrambled	P36993	P36993	Ppm1b	19043	0.737974368	0.08536752	0.24912012
ShSirt1_vs_ShScrambled	Q8K4Z5	Q8K4Z5	Sf3a1	67465	0.424211134	0.08537635	0.24912012
ShSirt1_vs_ShScrambled	P21278	P21278	Gna11	14672	0.305190999	0.0859554	0.25064772
ShSirt1_vs_ShScrambled	Q80YQ8	Q80YQ8;Q91	Rmnd5a		-1.050768038	0.0860275	0.25069602
ShSirt1_vs_ShScrambled	Q8R332	Q8R332	Nupl1	71844	0.670162327	0.08620075	0.25103884
ShSirt1_vs_ShScrambled	Q8R242	Q8R242	Ctbs	74245	0.505263153	0.08635655	0.25133041
ShSirt1_vs_ShScrambled	Q91XV3	Q91XV3	Basp1	70350	-0.748468136	0.08673871	0.25227999
ShSirt1_vs_ShScrambled	Q3TDQ1	Q3TDQ1	Stt3b	68292	-0.309528129	0.08701241	0.2529131
ShSirt1_vs_ShScrambled	Q9CQ79	Q9CQ79	Txndc9	98258	-0.646402583	0.08719952	0.25329384
ShSirt1_vs_ShScrambled	Q99K28	Q99K28	Arfgap2	77038	0.349336754	0.08739898	0.2535907
ShSirt1_vs_ShScrambled	Q6Q477	Q6Q477	Atp2b4	381290	0.731692	0.08741407	0.2535907
ShSirt1_vs_ShScrambled	P49312	P49312	Hnrnpa1	15382	0.359457616	0.08750258	0.25368443
ShSirt1_vs_ShScrambled	Q9WVM3	Q9WVM3	Anapc7	56317	-0.370237988	0.08772929	0.25417844
ShSirt1_vs_ShScrambled	Q9Z204	Q9Z204	Hnrnpc	15381	0.34065495	0.08783361	0.25428736
ShSirt1_vs_ShScrambled	Q60772	Q60772	Cdkn2c	12580	-0.597310623	0.08791275	0.25428736
ShSirt1_vs_ShScrambled	P62743	P62743	Ap2s1	232910	-1.445930296	0.08793588	0.25428736
ShSirt1_vs_ShScrambled	Q8R1N4	Q8R1N4	Nudcd3	209586	-0.276816137	0.08806909	0.2543857
ShSirt1_vs_ShScrambled	Q922F4	Q922F4	Tubb6	67951	0.267940842	0.0880826	0.2543857
ShSirt1_vs_ShScrambled	P51949	P51949	Mnat1	17420	0.861306363	0.08837622	0.25495308
ShSirt1_vs_ShScrambled	Q8BUV8	Q8BUV8	Gpr107	277463	0.505591805	0.08839723	0.25495308
ShSirt1_vs_ShScrambled	O35887	O35887	Calu	12321	0.467694577	0.08848443	0.25495308
ShSirt1_vs_ShScrambled	Q9EP89	Q9EP89	Lactb	80907	-0.414325086	0.08850498	0.25495308
ShSirt1_vs_ShScrambled	P28352	P28352	Apex1	11792	0.530987001	0.08863047	0.25515175

ShSirt1_vs_ShScrambled	Q9JHL1	Q9JHL1	Slc9a3r2	65962	1.200333713	0.08880236	0.25548366
ShSirt1_vs_ShScrambled	Q9CZX0	Q9CZX0	Elp3	74195	0.48655029	0.08900548	0.25570963
ShSirt1_vs_ShScrambled	Q8BQM4	Q8BQM4	Heatr3	234549	-0.582743818	0.08900859	0.25570963
ShSirt1_vs_ShScrambled	Q6PDI6	Q6PDI6	Fam63b	235461	-1.030466703	0.08905085	0.25570963
ShSirt1_vs_ShScrambled	Q8R059	Q8R059	Gale	74246	-0.279513789	0.08924496	0.25590045
ShSirt1_vs_ShScrambled	Q9CXR1	Q9CXR1	Dhrs7	66375	0.359600495	0.08928749	0.25590045
ShSirt1_vs_ShScrambled	Q921Y0	Q921Y0	Mob1a	232157	1.812511612	0.08931322	0.25590045
ShSirt1_vs_ShScrambled	P53395	P53395	Dbt	13171	-0.405427929	0.08934407	0.25590045
ShSirt1_vs_ShScrambled	P47941	P47941	Crkl	12929	0.37513598	0.08963071	0.25655868
ShSirt1_vs_ShScrambled	Q80UU9	Q80UU9	Pgrmc2	70804	-0.312928139	0.08969065	0.25656754
ShSirt1_vs_ShScrambled	P50580	P50580	Pa2g4	18813	0.327863955	0.08979787	0.25671157
ShSirt1_vs_ShScrambled	Q62009	Q62009	Postn	50706	-0.340598267	0.08993143	0.25693067
ShSirt1_vs_ShScrambled	P58871	P58871	Tnks1bp1	228140	0.399615137	0.09019369	0.25734801
ShSirt1_vs_ShScrambled	Q80X73	Q80X73	Pelo	105083	-0.591982832	0.09021152	0.25734801
ShSirt1_vs_ShScrambled	Q80U93	Q80U93	Nup214	227720	0.706020406	0.09024854	0.25734801
ShSirt1_vs_ShScrambled	Q8C9B9	Q8C9B9	Dido1	23856	-0.859055398	0.09040387	0.25755913
ShSirt1_vs_ShScrambled	Q8K2A7	Q8K2A7	Ints10	70885	-0.664794541	0.09043669	0.25755913
ShSirt1_vs_ShScrambled	Q571I9	Q571I9	Aldh16a1	69748	0.262135762	0.09060947	0.25786716
ShSirt1_vs_ShScrambled	Q9JJE7	Q9JJE7	Fads3	60527	0.46132376	0.09070411	0.25786716
ShSirt1_vs_ShScrambled	Q7TSS2	Q7TSS2	Ube2q1	70093	0.910202451	0.09071623	0.25786716
ShSirt1_vs_ShScrambled	Q8BFR5	Q8BFR5	Tufm	233870	-0.26048432	0.09094316	0.25834956
ShSirt1_vs_ShScrambled	Q78IS1	Q78IS1	Tmed3	66111	-0.674048393	0.09101852	0.258401
ShSirt1_vs_ShScrambled	P05202	P05202	Got2	14719	-0.250121865	0.09112822	0.25853783
ShSirt1_vs_ShScrambled	Q99JP0	Q99JP0	Map4k3	225028	-0.624028473	0.09118126	0.25853783
ShSirt1_vs_ShScrambled	A2A6Q5	A2A6Q5	Cdc27	217232	-1.015706596	0.09134437	0.25883773
ShSirt1_vs_ShScrambled	Q5U4D9	Q5U4D9	Thoc6	386612	0.451258918	0.09168898	0.2591003
ShSirt1_vs_ShScrambled	O08807	O08807	Prdx4	53381	0.43329102	0.09171359	0.2591003
ShSirt1_vs_ShScrambled	Q99PL6	Q99PL6	Ubxn6	66530	0.658593428	0.09173096	0.2591003
ShSirt1_vs_ShScrambled	Q00558	Q00558	F8a1		1.013264851	0.09173183	0.2591003
ShSirt1_vs_ShScrambled	O35215	O35215	Ddt	13202	-0.554020604	0.09174159	0.2591003
ShSirt1_vs_ShScrambled	Q99K01	Q99K01	Pdxdc1	94184	0.291168603	0.09178143	0.2591003
ShSirt1_vs_ShScrambled	P13439	P13439	Umps	22247	0.381726033	0.0919474	0.25940661
ShSirt1_vs_ShScrambled	Q6NS82	Q6NS82	Fam134a	227298	0.315298821	0.09218741	0.25984188
ShSirt1_vs_ShScrambled	Q14C51	Q14C51	Ptcd3	69956	-0.278394379	0.09221681	0.25984188
ShSirt1_vs_ShScrambled	Q8K2C8	Q8K2C8	Agpat6	102247	-0.582257677	0.09232439	0.25998271
ShSirt1_vs_ShScrambled	P97393	P97393	Arhgap5		0.568003621	0.09261147	0.26060125
ShSirt1_vs_ShScrambled	Q6PIU9	Q6PIU9	Q6PIU9	1.03E+08	-0.415753859	0.09265951	0.26060125
ShSirt1_vs_ShScrambled	Q8R5L3	Q8R5L3	Vps39	269338	-1.032957177	0.09283115	0.26089153
ShSirt1_vs_ShScrambled	P51859	P51859	Hdgf	15191	0.510748036	0.09294098	0.26089153
ShSirt1_vs_ShScrambled	Q9CZD3	Q9CZD3	Gars	353172	0.2349508	0.0929837	0.26089153
ShSirt1_vs_ShScrambled	Q9CQ71	Q9CQ71	Rpa3	68240	1.893646806	0.09304562	0.26089153
ShSirt1_vs_ShScrambled	P50136	P50136	Bckdha		-0.262063066	0.0930517	0.26089153
ShSirt1_vs_ShScrambled	P17439	P17439	Gba	14466	-0.408228325	0.09358884	0.26223465
ShSirt1_vs_ShScrambled	O35516	O35516	Notch2	18129	-0.746118203	0.09379759	0.26263988
ShSirt1_vs_ShScrambled	Q61024	Q61024	Asns	27053	0.257301508	0.09393989	0.26263988
ShSirt1_vs_ShScrambled	Q7TT23	Q7TT23	Q7TT23	228602	0.87869748	0.09396316	0.26263988
ShSirt1_vs_ShScrambled	Q3TIV5	Q3TIV5	Zc3h15	69082	-1.728471271	0.0939662	0.26263988
ShSirt1_vs_ShScrambled	Q99J95	Q99J95	Cdk9	107951	0.393354297	0.09411595	0.26289567
ShSirt1_vs_ShScrambled	Q5SYL3	Q5SYL3	Kiaa0100	72503	-0.99750207	0.09428992	0.26321874
ShSirt1_vs_ShScrambled	Q99KB8	Q99KB8	Hagh	14651	-0.503860905	0.09443627	0.26346437
ShSirt1_vs_ShScrambled	P10922	P10922	H1f0	14958	0.33022795	0.09463727	0.26386204
ShSirt1_vs_ShScrambled	Q9CQ75	Q9CQ75	Ndufa2	17991	-0.283595374	0.09469875	0.26387046
ShSirt1_vs_ShScrambled	Q91W67	Q91W67	Ubl7	69459	1.678549327	0.09484829	0.26400635
ShSirt1_vs_ShScrambled	Q5ND34	Q5ND34	Wdr81	192652	-0.304562068	0.09487084	0.26400635
ShSirt1_vs_ShScrambled	Q64437	Q64437	Adh7	11529	-0.841715758	0.09492298	0.26400635
ShSirt1_vs_ShScrambled	Q9Z1Y4	Q9Z1Y4	Trip6	22051	0.328492924	0.09506861	0.2641296
ShSirt1_vs_ShScrambled	Q8BFY6	Q8BFY6	Pef1	67898	-1.287629224	0.09518412	0.2641296
ShSirt1_vs_ShScrambled	Q99JW4	Q99JW4	Lims1	110829	-0.247491449	0.09519516	0.2641296
ShSirt1_vs_ShScrambled	Q9Z0L0	Q9Z0L0	Tpbg	21983	-0.991731815	0.09525159	0.2641296
ShSirt1_vs_ShScrambled	Q9CR95	Q9CR95	Necap1	67602	-0.30490796	0.09525985	0.2641296
ShSirt1_vs_ShScrambled	Q99MD9	Q99MD9	Nasp	50927	0.969243352	0.09539766	0.26434931
ShSirt1_vs_ShScrambled	P16332	P16332	Mut	17850	-0.241283994	0.09601851	0.26574868
ShSirt1_vs_ShScrambled	Q3UMW8	Q3UMW8	Cln5	211286	-1.364953862	0.09611703	0.26574868

ShSirt1_vs_ShScrambled	Q8BHL5	Q8BHL5	Elmo2	140579	-0.374662198	0.0961433	0.26574868
ShSirt1_vs_ShScrambled	Q6PB70	Q6PB70	Ano8	382014	-1.689961797	0.0961729	0.26574868
ShSirt1_vs_ShScrambled	Q4VBD2	Q4VBD2	Tapt1	231225	-0.445014926	0.09619702	0.26574868
ShSirt1_vs_ShScrambled	Q91WC0	Q91WC0	Setd3	52690	-0.463026777	0.09641084	0.26612277
ShSirt1_vs_ShScrambled	A2AJI0	A2AJI0	Map7d1	245877	0.45095905	0.09645035	0.26612277
ShSirt1_vs_ShScrambled	Q9DBE8	Q9DBE8	Alg2	56737	0.667127209	0.09661332	0.26640959
ShSirt1_vs_ShScrambled	P17427	P17427	Ap2a2	11772	-0.230694419	0.09687033	0.26695523
ShSirt1_vs_ShScrambled	Q3U182	Q3U182	Crtc2	74343	-1.310369094	0.09718457	0.26764252
ShSirt1_vs_ShScrambled	Q91YK2	Q91YK2	Rrp1b	72462	0.504359626	0.09723831	0.26764252
ShSirt1_vs_ShScrambled	Q60596	Q60596	Xrcc1	22594	1.640172542	0.09744218	0.26788719
ShSirt1_vs_ShScrambled	O70378	O70378	Emc8	18117	-0.471528092	0.0974459	0.26788719
ShSirt1_vs_ShScrambled	O35344	O35344	Kpna3	16648	0.743681299	0.09754627	0.26793665
ShSirt1_vs_ShScrambled	Q9CWS0	Q9CWS0	Ddah1	69219	-0.413816159	0.0975826	0.26793665
ShSirt1_vs_ShScrambled	Q59J78	Q59J78	Ndufaf2	75597	0.307808449	0.0976624	0.26799275
ShSirt1_vs_ShScrambled	P12815	P12815	Pdcd6	18570	0.344375908	0.09779045	0.26818109
ShSirt1_vs_ShScrambled	Q9D2N9	Q9D2N9	Vps33a	77573	-0.501574082	0.09819598	0.26908748
ShSirt1_vs_ShScrambled	Q99PT1	Q99PT1	Arhgdia	192662	1.531136369	0.09824018	0.26908748
ShSirt1_vs_ShScrambled	Q8VE95	Q8VE95	Q8VE95	223665	-0.379676378	0.098502	0.269641
ShSirt1_vs_ShScrambled	P62315	P62315	Snrpd1	20641	0.399843883	0.09869934	0.26986972
ShSirt1_vs_ShScrambled	Q6DVA0	Q6DVA0	Lemd2	224640	0.374804802	0.09872761	0.26986972
ShSirt1_vs_ShScrambled	Q80WW9	Q80WW9	Ddrgk1	77006	0.84914429	0.09880075	0.26986972
ShSirt1_vs_ShScrambled	Q80VI1	Q80VI1	Trim56	384309	-0.438788203	0.09882553	0.26986972
ShSirt1_vs_ShScrambled	Q8R3L2	Q8R3L2	Tcf25	66855	-1.049124518	0.09888447	0.26986972
ShSirt1_vs_ShScrambled	Q9R1T2	Q9R1T2	Sae1	56459	0.382131821	0.09897359	0.26994972
ShSirt1_vs_ShScrambled	Q9CRA5	Q9CRA5	Golph3	66629	0.532643176	0.09916356	0.27030454
ShSirt1_vs_ShScrambled	Q9JJG0	Q9JJG0	Tacc2	57752	-0.319114994	0.09946188	0.27051931
ShSirt1_vs_ShScrambled	Q62356	Q62356	Fstl1	14314	-0.967133848	0.09951019	0.27051931
ShSirt1_vs_ShScrambled	Q8VEE0	Q8VEE0	Rpe	66646	-1.359124764	0.09954966	0.27051931
ShSirt1_vs_ShScrambled	Q9Z0H8	Q9Z0H8	Clip2	269713	-0.242735848	0.09959721	0.27051931
ShSirt1_vs_ShScrambled	Q60668	Q60668	Hnrnpd	11991	0.405007944	0.09973733	0.27051931
ShSirt1_vs_ShScrambled	Q9CZR2	Q9CZR2	Naalad2	72560	-0.772155562	0.09973917	0.27051931
ShSirt1_vs_ShScrambled	Q922K7	Q922K7	Nop2		0.251746509	0.0998043	0.27051931
ShSirt1_vs_ShScrambled	Q8CAK1	Q8CAK1	Iba57	216792	0.805561117	0.09981392	0.27051931
ShSirt1_vs_ShScrambled	Q8K1N1	Q8K1N1	Pnpla8	67452	-0.236375634	0.09986248	0.27051931
ShSirt1_vs_ShScrambled	Q9CQS8	Q9CQS8	Sec61b	66212	-0.358197118	0.0998909	0.27051931
ShSirt1_vs_ShScrambled	Q9Z1X2	Q9Z1X2	Ptdss2	27388	-0.99880043	0.09990157	0.27051931
ShSirt1_vs_ShScrambled	Q3UR70	Q3UR70	Tgfbrap1	73122	0.471144243	0.10008439	0.27060958
ShSirt1_vs_ShScrambled	Q7TPD0	Q7TPD0	Ints3	229543	0.526818562	0.10012141	0.27060958
ShSirt1_vs_ShScrambled	Q8CFI7	Q8CFI7	Polr2b	231329	0.459737177	0.10016536	0.27060958
ShSirt1_vs_ShScrambled	P57722	P57722	Pcbp3	59093	0.539721126	0.1001747	0.27060958
ShSirt1_vs_ShScrambled	O35955	O35955	Psmb10	19171	0.962720312	0.10030259	0.27065189
ShSirt1_vs_ShScrambled	Q8K299	Q8K299	Scara5	71145	-1.729446106	0.10036311	0.27065189
ShSirt1_vs_ShScrambled	Q8BZ36	Q8BZ36	Rint1	72772	0.427524849	0.10037505	0.27065189
ShSirt1_vs_ShScrambled	Q9Z2I8	Q9Z2I8	Suclg2	20917	0.246614331	0.1004302	0.27065189
ShSirt1_vs_ShScrambled	Q8K3G9	Q8K3G9	Appl2	216190	-1.995582132	0.10088423	0.27153484
ShSirt1_vs_ShScrambled	Q8VDQ8	Q8VDQ8	Sirt2	64383	-0.302052233	0.1009158	0.27153484
ShSirt1_vs_ShScrambled	P46638	P46638;P624	Rab11b		-0.244769688	0.10100272	0.27153484
ShSirt1_vs_ShScrambled	Q5NCQ5	Q5NCQ5	Dph1	116905	-1.276962758	0.10100318	0.27153484
ShSirt1_vs_ShScrambled	Q91V81	Q91V81	Rbm42	68035	0.854527657	0.10108229	0.27153484
ShSirt1_vs_ShScrambled	Q9WVJ9	Q9WVJ9	Efemp2	58859	0.678826424	0.10111876	0.27153484
ShSirt1_vs_ShScrambled	Q9D1A2	Q9D1A2	Cndp2	66054	-0.248818278	0.10170962	0.27283957
ShSirt1_vs_ShScrambled	Q80W37	Q80W37	Snupn	66069	-0.954748617	0.10175647	0.27283957
ShSirt1_vs_ShScrambled	Q3UCV8	Q3UCV8	Otulin	432940	0.954588584	0.10178596	0.27283957
ShSirt1_vs_ShScrambled	Q5SUR0	Q5SUR0	Pfas	237823	0.331851741	0.10188556	0.27288906
ShSirt1_vs_ShScrambled	Q07797	Q07797	Lgals3bp	19039	0.63459629	0.10192533	0.27288906
ShSirt1_vs_ShScrambled	Q8BU11	Q8BU11	Tox4	268741	0.557137816	0.1020731	0.2729659
ShSirt1_vs_ShScrambled	A2BH40	A2BH40	Arid1a	93760	0.365036121	0.10207498	0.2729659
ShSirt1_vs_ShScrambled	O54962	O54962	Banf1	23825	0.477282816	0.10217876	0.27308166
ShSirt1_vs_ShScrambled	E1U8D0	E1U8D0	Soga1		1.157124854	0.1022748	0.27317659
ShSirt1_vs_ShScrambled	Q6P6M7	Q6P6M7	Sepsecs	211006	0.786770195	0.10272794	0.27422467
ShSirt1_vs_ShScrambled	Q9R0L6	Q9R0L6	Pcm1	18536	0.422727945	0.1029292	0.27459954
ShSirt1_vs_ShScrambled	Q3U0V1	Q3U0V1	Khsrp	16549	0.292642458	0.10384636	0.27688272
ShSirt1_vs_ShScrambled	P62880	P62880	Gnb2	14693	0.420297454	0.10391819	0.2769107

ShSirt1_vs_ShScrambled	Q9QXL2	Q9QXL2	Kif21a	16564	1.293338978	0.10407651	0.27716894
ShSirt1_vs_ShScrambled	Q9CY73	Q9CY73	Mrpl44	69163	-0.612582888	0.10432946	0.27751881
ShSirt1_vs_ShScrambled	Q8C1A3	Q8C1A3	Mtrr	210009	0.878728884	0.10435835	0.27751881
ShSirt1_vs_ShScrambled	P97819	P97819	Pla2g6	53357	-2.290857473	0.10439232	0.27751881
ShSirt1_vs_ShScrambled	Q9CQN1	Q9CQN1	Trap1	68015	-0.289967134	0.10451256	0.27752755
ShSirt1_vs_ShScrambled	Q9CX34	Q9CX34	Sugt1	67955	0.298525009	0.10451857	0.27752755
ShSirt1_vs_ShScrambled	P67871	P67871	Csnk2b	13001	0.466997382	0.1047152	0.2778862
ShSirt1_vs_ShScrambled	Q91V12	Q91V12	Acot7	70025	-0.260599818	0.10505318	0.27861932
ShSirt1_vs_ShScrambled	Q8C7H1	Q8C7H1	Mmaa	109136	-1.129940141	0.10523842	0.27883921
ShSirt1_vs_ShScrambled	Q924D0	Q924D0	Rtn4ip1	170728	-0.362934859	0.10535633	0.27883921
ShSirt1_vs_ShScrambled	P39054	P39054	Dnm2	13430	-0.243794128	0.10536482	0.27883921
ShSirt1_vs_ShScrambled	P58501	P58501	Paxbp1	67367	1.081010985	0.10544135	0.27883921
ShSirt1_vs_ShScrambled	Q8CGC7	Q8CGC7	Eprs	107508	0.22911736	0.10544495	0.27883921
ShSirt1_vs_ShScrambled	P54775	P54775	Psmc4	23996	-0.260427896	0.10556525	0.27885096
ShSirt1_vs_ShScrambled	Q9D853	Q9D853	Mettl10	72096	-0.538439374	0.10557295	0.27885096
ShSirt1_vs_ShScrambled	O88286	O88286	Wiz	22404	0.684843672	0.10568572	0.27898559
ShSirt1_vs_ShScrambled	Q5U419	Q5U419	Mfsd3	69572	0.933276245	0.10582312	0.27918503
ShSirt1_vs_ShScrambled	Q9DAM5	Q9DAM5	Slc25a19	67283	-0.573873334	0.10588729	0.27919114
ShSirt1_vs_ShScrambled	Q9CWM4	Q9CWM4	Pfdn1		-0.759439007	0.10611385	0.27962517
ShSirt1_vs_ShScrambled	Q8BRH0	Q8BRH0	Tmtc3	237500	-0.566460139	0.10623267	0.27977495
ShSirt1_vs_ShScrambled	P97370	P97370	Atp1b3	11933	0.95075523	0.1064855	0.28027482
ShSirt1_vs_ShScrambled	Q8BHT7	Q8BHT7	Rgp1	242406	-0.929577151	0.10654665	0.28027482
ShSirt1_vs_ShScrambled	Q9CZ42	Q9CZ42	Carkd	69225	0.342167508	0.10663075	0.28033269
ShSirt1_vs_ShScrambled	Q8CG76	Q8CG76	Akr7a2	110198	0.250526582	0.10678913	0.28058564
ShSirt1_vs_ShScrambled	Q91WJ8	Q91WJ8	Fubp1		-0.382224338	0.10690498	0.28069983
ShSirt1_vs_ShScrambled	P35550	P35550	Fbl	14113	0.506153891	0.10695696	0.28069983
ShSirt1_vs_ShScrambled	Q8R1K1	Q8R1K1	Ubac2	68889	-0.337869336	0.10736384	0.28160394
ShSirt1_vs_ShScrambled	A8Y5H7	A8Y5H7	Sec14l1	74136	-0.6369326	0.1074849	0.28175774
ShSirt1_vs_ShScrambled	Q91V04	Q91V04	Tram1	72265	1.363444143	0.10756902	0.28181461
ShSirt1_vs_ShScrambled	Q9JMB0	Q9JMB0	Gkap1	56278	0.559852918	0.10782398	0.2820465
ShSirt1_vs_ShScrambled	P97496	P97496	Smarcc1	20588	0.544936064	0.10784347	0.2820465
ShSirt1_vs_ShScrambled	Q9JKX6	Q9JKX6	Nudt5	53893	-0.283509849	0.10792832	0.2820465
ShSirt1_vs_ShScrambled	P50096	P50096	Impdh1	23917	0.272909362	0.10794194	0.2820465
ShSirt1_vs_ShScrambled	P14152	P14152	Mdh1	17449	-0.295401545	0.10800668	0.2820465
ShSirt1_vs_ShScrambled	Q3V1T4	Q3V1T4	Lepre1	56401	0.344103702	0.10803243	0.2820465
ShSirt1_vs_ShScrambled	Q8BM55	Q8BM55	Tmem214	68796	0.468876804	0.10824051	0.28242641
ShSirt1_vs_ShScrambled	Q9D7E3	Q9D7E3	Ovca2	246257	-0.322498517	0.10870248	0.28346794
ShSirt1_vs_ShScrambled	Q8BUV3	Q8BUV3	Gphn	268566	-0.31411937	0.10900283	0.28408706
ShSirt1_vs_ShScrambled	Q921F4	Q921F4	Hnrnpll	72692	0.301327494	0.10917483	0.28437115
ShSirt1_vs_ShScrambled	O09167	O09167	Rpl21		-0.271578677	0.10944413	0.28478605
ShSirt1_vs_ShScrambled	Q9DBF1	Q9DBF1	Aldh7a1	110695	-0.238726785	0.1094603	0.28478605
ShSirt1_vs_ShScrambled	P15532	P15532	Nme1	18102	-0.79671861	0.10962706	0.28499201
ShSirt1_vs_ShScrambled	Q9DBG9	Q9DBG9	Tax1bp3	76281	-0.36035256	0.10966573	0.28499201
ShSirt1_vs_ShScrambled	P50543	P50543	S100a11	20195	-0.485318263	0.10986891	0.28519611
ShSirt1_vs_ShScrambled	P83741	P83741	Wnk1	232341	0.342523119	0.10987063	0.28519611
ShSirt1_vs_ShScrambled	O88477	O88477	Igf2bp1	140486	0.314098101	0.11002378	0.2854295
ShSirt1_vs_ShScrambled	E9Q3C1	E9Q3C1	C2cd2	207781	0.936701703	0.11043208	0.28631356
ShSirt1_vs_ShScrambled	Q570Y9	Q570Y9	Deptor	97998	0.505752099	0.11057545	0.28631356
ShSirt1_vs_ShScrambled	Q91V61	Q91V61	Sfxn3	94280	-0.29742329	0.11057651	0.28631356
ShSirt1_vs_ShScrambled	Q99M28	Q99M28	Rnps1	19826	0.40085931	0.11061827	0.28631356
ShSirt1_vs_ShScrambled	Q9CX56	Q9CX56	Psmd8	57296	-0.480610246	0.11078215	0.28657342
ShSirt1_vs_ShScrambled	Q8BX57	Q8BX57	Pxk	218699	0.580461983	0.11084951	0.28658345
ShSirt1_vs_ShScrambled	O88986	O88986	Gcat	26912	0.439136321	0.11097311	0.28661067
ShSirt1_vs_ShScrambled	A6H630	A6H630	Armt1	73419	0.878592058	0.11098703	0.28661067
ShSirt1_vs_ShScrambled	P62806	P62806	Hist1h4a	69386; 97	0.359070445	0.11109381	0.2867224
ShSirt1_vs_ShScrambled	Q9CR16	Q9CR16	Ppid	67738	0.296550961	0.11119042	0.28680774
ShSirt1_vs_ShScrambled	Q8VBW6	Q8VBW6	Nae1	234664	0.295826888	0.1112825	0.28688134
ShSirt1_vs_ShScrambled	Q8K3B1	Q8K3B1	Fbxo45	268882	0.698765626	0.11152879	0.28735214
ShSirt1_vs_ShScrambled	P30999	P30999	Ctnnd1	12388	-0.387453234	0.11178868	0.28780302
ShSirt1_vs_ShScrambled	O70194	O70194	Eif3d	55944	0.263886564	0.1118313	0.28780302
ShSirt1_vs_ShScrambled	E9Q7G0	E9Q7G0	Numa1	101706	0.259021328	0.11190468	0.28782776
ShSirt1_vs_ShScrambled	Q64737	Q64737	Gart	14450	0.228272834	0.11254125	0.28930022
ShSirt1_vs_ShScrambled	O08586	O08586	Pten	19211	0.863968711	0.11260608	0.28930212

ShSirt1_vs_ShScrambled	Q9EQ61	Q9EQ61	Pes1	64934	0.47102123	0.11286567	0.28966514
ShSirt1_vs_ShScrambled	Q9WTQ8	Q9WTQ8	Timm23		0.324227811	0.11293226	0.28966514
ShSirt1_vs_ShScrambled	Q9CX86	Q9CX86	Hnrnpa0	77134	0.232357574	0.11298964	0.28966514
ShSirt1_vs_ShScrambled	Q9D5T0	Q9D5T0	Atad1	67979	0.234942472	0.11304688	0.28966514
ShSirt1_vs_ShScrambled	Q8K2X3	Q8K2X3	Obfc1	108689	0.897750314	0.11306823	0.28966514
ShSirt1_vs_ShScrambled	Q8BXN9	Q8BXN9	Tmem87a	211499	0.561431383	0.11329023	0.28980579
ShSirt1_vs_ShScrambled	Q69Z99	Q69Z99	Znf512	269639	0.746522126	0.11331158	0.28980579
ShSirt1_vs_ShScrambled	P18760	P18760	Cfl1	12631	0.234747004	0.11337527	0.28980579
ShSirt1_vs_ShScrambled	Q9CQN7	Q9CQN7	Mrpl41	107733	-0.831510352	0.11340957	0.28980579
ShSirt1_vs_ShScrambled	Q7TSE6	Q7TSE6	Stk38l	232533	0.916937926	0.11347404	0.28980579
ShSirt1_vs_ShScrambled	Q9DC37	Q9DC37	Mfsd1	66868	-0.449442157	0.11350834	0.28980579
ShSirt1_vs_ShScrambled	Q64475	Q64475;Q8C	Hist1h2bb		0.326123616	0.11372686	0.28993625
ShSirt1_vs_ShScrambled	O35593	O35593	Psmd14	59029	-0.270924024	0.11372978	0.28993625
ShSirt1_vs_ShScrambled	Q04857	Q04857	Col6a1	12833	0.350434448	0.11375213	0.28993625
ShSirt1_vs_ShScrambled	Q6GSS7	Q6GSS7;Q6	Hist2h2aa1		2.267680989	0.11383305	0.28997878
ShSirt1_vs_ShScrambled	P68181	P68181	Prkacb	18749	1.036686641	0.11400325	0.29010216
ShSirt1_vs_ShScrambled	Q3USH5	Q3USH5	Sfswap	231769	0.901401778	0.1140209	0.29010216
ShSirt1_vs_ShScrambled	O35841	O35841	Api5	11800	0.410401477	0.11407502	0.29010216
ShSirt1_vs_ShScrambled	P35569	P35569	Irs1	16367	-1.263712819	0.11421387	0.29010216
ShSirt1_vs_ShScrambled	G5E870	G5E870	Trip12	14897	-0.383153123	0.1142413	0.29010216
ShSirt1_vs_ShScrambled	O70152	O70152	Dpm1	13480	0.243877039	0.11426709	0.29010216
ShSirt1_vs_ShScrambled	Q9R0M6	Q9R0M6	Rab9a	56382	-0.413257997	0.1143327	0.29010558
ShSirt1_vs_ShScrambled	Q9ES28	Q9ES28	Arhgef7	54126	-0.36828011	0.11444767	0.29023416
ShSirt1_vs_ShScrambled	P52332	P52332	Jak1		-0.590143666	0.11461503	0.29037959
ShSirt1_vs_ShScrambled	Q8BMK0	Q8BMK0	Cep85	70012	0.890453003	0.11464188	0.29037959
ShSirt1_vs_ShScrambled	Q7TN29	Q7TN29	Smap2	69780	-0.890195318	0.11469801	0.29037959
ShSirt1_vs_ShScrambled	Q8CDJ8	Q8CDJ8	Ston1	77057	-0.452303895	0.11522202	0.2915427
ShSirt1_vs_ShScrambled	Q8BMJ2	Q8BMJ2	Lars	107045	0.268200326	0.11542705	0.29189787
ShSirt1_vs_ShScrambled	O70172	O70172	Pip4k2a	18718	-0.886128467	0.11558892	0.29214355
ShSirt1_vs_ShScrambled	P08030	P08030	Aprt	11821	0.3248701	0.11586111	0.29256558
ShSirt1_vs_ShScrambled	Q9CQY6	Q9CQY6	Uqcc2	67267	0.482850224	0.11588553	0.29256558
ShSirt1_vs_ShScrambled	Q8VCS3	Q8VCS3	Fam20b	215015	1.930247651	0.11612194	0.29299857
ShSirt1_vs_ShScrambled	Q62351	Q62351	Tfrc	22042	-0.346750795	0.11619971	0.293031
ShSirt1_vs_ShScrambled	Q3UDK1	Q3UDK1	Trafd1	231712	0.882592032	0.1163715	0.29330036
ShSirt1_vs_ShScrambled	Q9R008	Q9R008	Mvk	17855	-0.351399039	0.11650679	0.29347748
ShSirt1_vs_ShScrambled	Q7TSV4	Q7TSV4	Pgm2	66681	0.271005648	0.11712625	0.29487334
ShSirt1_vs_ShScrambled	Q99L48	Q99L48	Nmd3	97112	-1.444282409	0.11742362	0.29522729
ShSirt1_vs_ShScrambled	O70401	O70401	Tspan6		0.52532049	0.11742459	0.29522729
ShSirt1_vs_ShScrambled	Q921J2	Q921J2	Rheb	19744	-0.252512836	0.11746305	0.29522729
ShSirt1_vs_ShScrambled	O88967	O88967	Yme1l1	27377	-0.316967627	0.11769584	0.29564776
ShSirt1_vs_ShScrambled	Q4PZA2	Q4PZA2	Ece1	230857	0.535841456	0.11836885	0.29707869
ShSirt1_vs_ShScrambled	Q9CQH7	Q9CQH7	Btf3l4	70533	0.342123111	0.11839711	0.29707869
ShSirt1_vs_ShScrambled	O35382	O35382	Exoc4	20336	0.318882829	0.11880654	0.29794039
ShSirt1_vs_ShScrambled	Q61074	Q61074	Ppm1g	14208	0.25219567	0.11890832	0.29799026
ShSirt1_vs_ShScrambled	Q8C0J2	Q8C0J2	Atg16l1	77040	-0.856943485	0.11895845	0.29799026
ShSirt1_vs_ShScrambled	Q5U3K5	Q5U3K5	Rabl6	227624	0.296689521	0.11923482	0.29842143
ShSirt1_vs_ShScrambled	Q8CGB3	Q8CGB3	Uaca	72565	-0.708091218	0.1192628	0.29842143
ShSirt1_vs_ShScrambled	P15116	P15116	Cdh2	12558	-0.868776358	0.11950058	0.29885076
ShSirt1_vs_ShScrambled	A2A432	A2A432	Cul4b	72584	-0.253381664	0.1196581	0.2990061
ShSirt1_vs_ShScrambled	Q6ZPR6	Q6ZPR6	Ibtk	108837	-2.990526688	0.11969518	0.2990061
ShSirt1_vs_ShScrambled	Q8K4X7	Q8K4X7	Agpat4	68262	0.457242956	0.12013149	0.29980588
ShSirt1_vs_ShScrambled	Q9JHS9	Q9JHS9	Cwc15	66070	0.668702764	0.12014817	0.29980588
ShSirt1_vs_ShScrambled	Q922H2	Q922H2	Pdk3	236900	0.668691969	0.12049077	0.30042158
ShSirt1_vs_ShScrambled	Q99LI2	Q99LI2	Clcc1	229725	1.065434359	0.12058084	0.30042158
ShSirt1_vs_ShScrambled	Q9EP97	Q9EP97	Senp3	80886	0.462931604	0.12059457	0.30042158
ShSirt1_vs_ShScrambled	Q9D880	Q9D880	Timm50	66525	-0.422085042	0.12078596	0.30073239
ShSirt1_vs_ShScrambled	P61750	P61750	Arf4	11843	-0.30888462	0.12087304	0.3007833
ShSirt1_vs_ShScrambled	P54729	P54729	Nub1	53312	0.474851638	0.12103671	0.30102464
ShSirt1_vs_ShScrambled	Q8BFQ8	Q8BFQ8	Pddc1	213350	0.217274625	0.12115075	0.30114233
ShSirt1_vs_ShScrambled	O89110	O89110	Casp8	12370	-0.403952621	0.12125047	0.30122433
ShSirt1_vs_ShScrambled	P60487	P60487	Pdxp	57028	-0.603934768	0.12131925	0.30122942
ShSirt1_vs_ShScrambled	Q8BTJ4	Q8BTJ4	Enpp4	224794	-0.532780995	0.12144051	0.30136475
ShSirt1_vs_ShScrambled	P56695	P56695	Wfs1	22393	0.712687485	0.12157823	0.30154074

ShSirt1_vs_ShScrambled	Q9CQT5	Q9CQT5	Pomp	66537	-2.950377421	0.12166092	0.3015801
ShSirt1_vs_ShScrambled	Q9D1H7	Q9D1H7	Get4	67604	0.26924409	0.12181389	0.30165334
ShSirt1_vs_ShScrambled	O70404	O70404	Vamp8	22320	0.375438598	0.12182411	0.30165334
ShSirt1_vs_ShScrambled	Q9WTX5	Q9WTX5	Skp1	21402	-0.738237155	0.12244687	0.30302916
ShSirt1_vs_ShScrambled	Q9QYF1	Q9QYF1	Rdh11	17252	-0.394441783	0.1226553	0.30332001
ShSirt1_vs_ShScrambled	P62748	P62748;P840	Hpcal1		-0.446560247	0.12269879	0.30332001
ShSirt1_vs_ShScrambled	Q8VDK1	Q8VDK1	Nit1	27045	0.45702478	0.1228434	0.30346621
ShSirt1_vs_ShScrambled	Q91YM2	Q91YM2	Arhgap35	232906	0.500760697	0.12291791	0.30346621
ShSirt1_vs_ShScrambled	Q9D0I8	Q9D0I8	Mrto4	69902	0.604348551	0.12297414	0.30346621
ShSirt1_vs_ShScrambled	Q91VU7	Q91VU7	Pus7	78697	0.283469536	0.12308221	0.30346621
ShSirt1_vs_ShScrambled	Q9CZX7	Q9CZX7	Tmem55a	72519	-0.577338241	0.12310228	0.30346621
ShSirt1_vs_ShScrambled	Q9D7K5	Q9D7K5	Atp5sl	66349	-0.45957937	0.12320444	0.30346621
ShSirt1_vs_ShScrambled	P62267	P62267	Rps23	66475	-0.291104781	0.12323198	0.30346621
ShSirt1_vs_ShScrambled	O09172	O09172	Gclm	14630	-0.374047571	0.12333015	0.30346621
ShSirt1_vs_ShScrambled	P45878	P45878	Fkbp2	14227	-0.31153207	0.12338273	0.30346621
ShSirt1_vs_ShScrambled	Q9D975	Q9D975	Srxn1	76650	0.852122717	0.12343021	0.30346621
ShSirt1_vs_ShScrambled	P23475	P23475	Xrcc6	14375	-0.525434653	0.12362419	0.30377767
ShSirt1_vs_ShScrambled	Q91YP0	Q91YP0	L2hgdh	217666	0.404158718	0.12380848	0.304065
ShSirt1_vs_ShScrambled	Q61245	Q61245	Col11a1	12814	0.72716925	0.12411375	0.30464898
ShSirt1_vs_ShScrambled	Q9Z2B9	Q9Z2B9	Rps6ka4	56613	0.274039294	0.1244191	0.30523252
ShSirt1_vs_ShScrambled	O09174	O09174	Amacr	17117	-0.21455216	0.12473232	0.30573675
ShSirt1_vs_ShScrambled	Q99L20	Q99L20	Gstt3	103140	-0.521390797	0.1247601	0.30573675
ShSirt1_vs_ShScrambled	Q9CZ82	Q9CZ82	Med18	67219	0.834947802	0.1250052	0.30617118
ShSirt1_vs_ShScrambled	P60824	P60824	Cirbp	12696	0.59844549	0.12518855	0.3063725
ShSirt1_vs_ShScrambled	Q8BHS6	Q8BHS6	Armcx3	71703	-1.030516154	0.12522314	0.3063725
ShSirt1_vs_ShScrambled	Q5SV80	Q5SV80	Myo19	66196	1.545491904	0.12538231	0.30657758
ShSirt1_vs_ShScrambled	Q9EST4	Q9EST4	Psmg2	107047	0.345301196	0.1254428	0.30657758
ShSirt1_vs_ShScrambled	Q8CGA0	Q8CGA0	Ppm1f	68606	-0.335174679	0.12556346	0.30670642
ShSirt1_vs_ShScrambled	P70245	P70245	Ebp	13595	-1.357523533	0.12604828	0.30748804
ShSirt1_vs_ShScrambled	P47964	P47964	Rpl36		-0.311336441	0.1261916	0.30748804
ShSirt1_vs_ShScrambled	Q920B9	Q920B9	Supt16h	114741	0.285980375	0.12622929	0.30748804
ShSirt1_vs_ShScrambled	O88879	O88879	Apaf1	11783	1.555860745	0.12623264	0.30748804
ShSirt1_vs_ShScrambled	O08734	O08734	Bak1	12018	-0.272254328	0.12625011	0.30748804
ShSirt1_vs_ShScrambled	Q6PER3	Q6PER3	Mapre3	100732	-1.158319997	0.1262938	0.30748804
ShSirt1_vs_ShScrambled	Q9D1H8	Q9D1H8	Mrpl53	68499	0.412706056	0.12638834	0.30748804
ShSirt1_vs_ShScrambled	Q9JLB2	Q9JLB2	Mpp5	56217	-0.744419659	0.1264284	0.30748804
ShSirt1_vs_ShScrambled	P28700	P28700	Rxra	20181	0.382645221	0.12655565	0.30757543
ShSirt1_vs_ShScrambled	Q62245	Q62245	Sos1	20662	-0.919313574	0.12660061	0.30757543
ShSirt1_vs_ShScrambled	Q9DB40	Q9DB40	Med27	68975	0.507742864	0.12693628	0.30822505
ShSirt1_vs_ShScrambled	Q9WV02	Q9WV02	Rbmx	19655	0.837461258	0.12703982	0.30831061
ShSirt1_vs_ShScrambled	Q8BGB7	Q8BGB7	Enoph1	67870	-0.52074998	0.12725509	0.30860496
ShSirt1_vs_ShScrambled	Q9CQT1	Q9CQT1	Mri1	67873	0.492626596	0.12729784	0.30860496
ShSirt1_vs_ShScrambled	P49962	P49962	Srp9	27058	0.835926811	0.12742601	0.30870346
ShSirt1_vs_ShScrambled	Q8BH97	Q8BH97	Rcn3	52377	-0.322168571	0.12747524	0.30870346
ShSirt1_vs_ShScrambled	Q9R0Q6	Q9R0Q6	Arpc1a	56443	-0.83462623	0.12775463	0.30921415
ShSirt1_vs_ShScrambled	Q9WUM4	Q9WUM4	Coro1c	23790	0.248704206	0.12791194	0.30942899
ShSirt1_vs_ShScrambled	Q8BIA4	Q8BIA4	Fbxw8	231672	0.335291672	0.12808579	0.30968358
ShSirt1_vs_ShScrambled	Q9CXA2	Q9CXA2	L3hypdh	67217	-0.550260298	0.12844017	0.31030114
ShSirt1_vs_ShScrambled	Q9CZI9	Q9CZI9	Aen	68048	-0.831776777	0.1284787	0.31030114
ShSirt1_vs_ShScrambled	Q9R099	Q9R099	Tbl2	27368	0.286978512	0.12863444	0.31037173
ShSirt1_vs_ShScrambled	Q99LR1	Q99LR1	Abhd12	76192	-0.339310144	0.12870646	0.31037173
ShSirt1_vs_ShScrambled	Q501J7	Q501J7	Phactr4	100169	0.373269348	0.12871419	0.31037173
ShSirt1_vs_ShScrambled	Q9QZ06	Q9QZ06	Tollip	54473	-0.277271331	0.12891064	0.31067946
ShSirt1_vs_ShScrambled	Q9CRG1	Q9CRG1	Tm7sf3	67623	-0.986209864	0.12905125	0.31085237
ShSirt1_vs_ShScrambled	Q5SXY1	Q5SXY1	Specc1		0.451552565	0.12915572	0.31093319
ShSirt1_vs_ShScrambled	Q8VE19	Q8VE19	Mios	252875	0.297738229	0.12924868	0.31093319
ShSirt1_vs_ShScrambled	Q9CQ02	Q9CQ02	Commd4	66199	2.195188098	0.12929145	0.31093319
ShSirt1_vs_ShScrambled	P25911	P25911	Lyn	17096	-0.826988928	0.12970818	0.31176928
ShSirt1_vs_ShScrambled	Q64105	Q64105	Spr		0.2915203	0.13014065	0.31257797
ShSirt1_vs_ShScrambled	Q8K274	Q8K274	Fn3krp	238024	0.352768729	0.13018312	0.31257797
ShSirt1_vs_ShScrambled	Q9R0H0	Q9R0H0	Acox1	11430	-0.256975462	0.13034419	0.31277479
ShSirt1_vs_ShScrambled	F8VPU2	F8VPU2	Farp1	223254	0.498106747	0.13040367	0.31277479
ShSirt1_vs_ShScrambled	O70258	O70258	Sgce	20392	-0.822091528	0.13079613	0.31354951

ShSirt1_vs_ShScrambled	Q5F2F2	Q5F2F2	Abhd15	67477	-0.680477797	0.13152163	0.31498321
ShSirt1_vs_ShScrambled	Q922X9	Q922X9	Prmt7	214572	1.391044061	0.13153375	0.31498321
ShSirt1_vs_ShScrambled	Q9DBN5	Q9DBN5	Lonp2	66887	-0.454887341	0.13169145	0.31510818
ShSirt1_vs_ShScrambled	Q8R550	Q8R550	Sh3kbp1	58194	-0.266350439	0.13172555	0.31510818
ShSirt1_vs_ShScrambled	P53811	P53811	Pitpnb	56305	0.211439003	0.13207777	0.3157834
ShSirt1_vs_ShScrambled	Q8VDZ4	Q8VDZ4	Zdhhc5	228136	0.648755367	0.1323913	0.31623843
ShSirt1_vs_ShScrambled	Q8K1M6	Q8K1M6	Dnm1l	74006	-0.393959154	0.1324082	0.31623843
ShSirt1_vs_ShScrambled	Q78PY7	Q78PY7	Snd1	56463	-0.204677209	0.13273803	0.31685852
ShSirt1_vs_ShScrambled	Q3UFS0	Q3UFS0	Zyg11b	414872	0.79505598	0.13285659	0.31697393
ShSirt1_vs_ShScrambled	O88532	O88532	Zfr	22763	0.281736533	0.1330554	0.31728054
ShSirt1_vs_ShScrambled	Q8C3X4	Q8C3X4	Guf1	231279	0.474003066	0.13336871	0.3176702
ShSirt1_vs_ShScrambled	P70290	P70290	Mpp1	17524	-0.363150465	0.13339941	0.3176702
ShSirt1_vs_ShScrambled	Q91YR7	Q91YR7	Prpf6	68879	0.874782492	0.13342993	0.3176702
ShSirt1_vs_ShScrambled	Q9Z2D1	Q9Z2D1	Mtmr2	77116	-0.81182267	0.1337118	0.31817346
ShSirt1_vs_ShScrambled	Q9JHH9	Q9JHH9	Copz2	56358	0.511012019	0.13390083	0.31842952
ShSirt1_vs_ShScrambled	A2AWA9	A2AWA9	Rabgap1	227800	0.381509076	0.13399501	0.31842952
ShSirt1_vs_ShScrambled	Q69ZA1	Q69ZA1	Cdk13	69562	0.509816351	0.13405575	0.31842952
ShSirt1_vs_ShScrambled	Q921I9	Q921I9	Exosc4	109075	0.33961121	0.13410158	0.31842952
ShSirt1_vs_ShScrambled	Q3TYX3	Q3TYX3	Smyd5	232187	0.388824667	0.13427489	0.31867344
ShSirt1_vs_ShScrambled	Q99N91	Q99N91	Mrpl34	94065	-0.605987612	0.13466806	0.31924357
ShSirt1_vs_ShScrambled	Q9WV70	Q9WV70	Noc2l		0.681329921	0.13471035	0.31924357
ShSirt1_vs_ShScrambled	Q9CYZ2	Q9CYZ2	Tpd52l2	66314	-0.249634248	0.13472729	0.31924357
ShSirt1_vs_ShScrambled	Q3TCJ1	Q3TCJ1	Fam175b	109359	0.359268236	0.13503764	0.31981108
ShSirt1_vs_ShScrambled	Q7TNE3	Q7TNE3	Spag7	216873	1.381201693	0.13523281	0.32010534
ShSirt1_vs_ShScrambled	Q9DCT6	Q9DCT6	Bap18	104457	0.471261852	0.13543525	0.32024985
ShSirt1_vs_ShScrambled	Q99N94	Q99N94	Mrpl9	78523	-1.82778872	0.1354645	0.32024985
ShSirt1_vs_ShScrambled	P70295	P70295	Aup1		-0.249436259	0.13552451	0.32024985
ShSirt1_vs_ShScrambled	Q9Z0W3	Q9Z0W3	Nup160	59015	0.304686484	0.13558896	0.32024985
ShSirt1_vs_ShScrambled	A2BDX3	A2BDX3	Mocs3	69372	-0.634343481	0.13564859	0.32024985
ShSirt1_vs_ShScrambled	P17809	P17809	Slc2a1	20525	0.715976654	0.13584213	0.32043917
ShSirt1_vs_ShScrambled	Q70FJ1	Q70FJ1	Akap9	100986	0.371285148	0.13589211	0.32043917
ShSirt1_vs_ShScrambled	Q99ME9	Q99ME9	Gtpbp4	69237	0.961288868	0.13603252	0.32043917
ShSirt1_vs_ShScrambled	Q8BIP0	Q8BIP0	Dars2	226539	0.911183977	0.13608453	0.32043917
ShSirt1_vs_ShScrambled	P05201	P05201	Got1	14718	0.261227728	0.13608997	0.32043917
ShSirt1_vs_ShScrambled	Q62230	Q62230	Siglec1	20612	-0.35366496	0.1361547	0.32043917
ShSirt1_vs_ShScrambled	O08759	O08759	Ube3a	22215	0.280119538	0.13655569	0.32116201
ShSirt1_vs_ShScrambled	Q9WTX2	Q9WTX2	Prkra	23992	0.265402286	0.13671378	0.32116201
ShSirt1_vs_ShScrambled	Q99J77	Q99J77	Nans	94181	-0.384110607	0.13671975	0.32116201
ShSirt1_vs_ShScrambled	Q8R3N6	Q8R3N6	Thoc1	225160	-0.698886254	0.13674643	0.32116201
ShSirt1_vs_ShScrambled	Q91X21	Q91X21	Kiaa2013	77034	0.474956044	0.13687128	0.32128808
ShSirt1_vs_ShScrambled	P08556	P08556	Nras		0.79936746	0.13712833	0.32142235
ShSirt1_vs_ShScrambled	Q9CQI6	Q9CQI6	Cotl1	72042	0.327771995	0.13714611	0.32142235
ShSirt1_vs_ShScrambled	Q8BH59	Q8BH59	Slc25a12	78830	0.210325396	0.13722813	0.32142235
ShSirt1_vs_ShScrambled	Q9R1C7	Q9R1C7	Prpf40a	56194	0.428304681	0.13725531	0.32142235
ShSirt1_vs_ShScrambled	Q8VCF0	Q8VCF0	Mavs	228607	-0.257689537	0.13732251	0.32142235
ShSirt1_vs_ShScrambled	Q9CYW4	Q9CYW4	Hdhd3	72748	-0.224015279	0.13737065	0.32142235
ShSirt1_vs_ShScrambled	P00397	P00397	Mtco1	17708	-0.458038019	0.13746208	0.32142235
ShSirt1_vs_ShScrambled	Q8R1V4	Q8R1V4	Tmed4	103694	-0.220063939	0.13749813	0.32142235
ShSirt1_vs_ShScrambled	Q9Z103	Q9Z103	Adnp	11538	0.296070147	0.13764081	0.32158935
ShSirt1_vs_ShScrambled	Q8CGU1	Q8CGU1	Calcoco1	67488	0.296865096	0.13775279	0.32164457
ShSirt1_vs_ShScrambled	Q9CW79	Q9CW79	Golga1	76899	-0.637409208	0.13784901	0.32164457
ShSirt1_vs_ShScrambled	Q922B2	Q922B2	Dars	226414	0.205319882	0.13787821	0.32164457
ShSirt1_vs_ShScrambled	O70493	O70493	Snx12	55988	-0.297300861	0.13796957	0.32169145
ShSirt1_vs_ShScrambled	P50428	P50428	Arsa	11883	0.425965518	0.1380696	0.32175849
ShSirt1_vs_ShScrambled	P45591	P45591	Cfl2	12632	-0.531585531	0.13836883	0.32228943
ShSirt1_vs_ShScrambled	P17710	P17710	Hk1	15275	0.23026059	0.13847082	0.32233192
ShSirt1_vs_ShScrambled	Q922H9	Q922H9	Znf330	30932	-1.026165148	0.13863572	0.32233192
ShSirt1_vs_ShScrambled	Q9D0F3	Q9D0F3	Lman1	70361	-0.245781394	0.13868115	0.32233192
ShSirt1_vs_ShScrambled	P21460	P21460	Cst3	13010	0.56386124	0.13881334	0.32233192
ShSirt1_vs_ShScrambled	Q8R2Q4	Q8R2Q4	Gfm2	320806	0.262171997	0.13881927	0.32233192
ShSirt1_vs_ShScrambled	Q9D0E1	Q9D0E1	Hnrnpm	76936	0.227809254	0.13884267	0.32233192
ShSirt1_vs_ShScrambled	Q9CQW2	Q9CQW2	Arl8b	67166	-0.335239587	0.13894145	0.32233192
ShSirt1_vs_ShScrambled	Q8K370	Q8K370	Acad10	71985	0.335618122	0.13902469	0.32233192

ShSirt1_vs_ShScrambled	P70188	P70188	Kifap3	16579	-0.483757088	0.1391833	0.32233192
ShSirt1_vs_ShScrambled	P56480	P56480	Atp5b	11947	-0.19663112	0.1392033	0.32233192
ShSirt1_vs_ShScrambled	Q8C570	Q8C570	Rae1	66679	0.210920711	0.13923355	0.32233192
ShSirt1_vs_ShScrambled	Q68FL6	Q68FL6	Mars	216443	0.212757226	0.13924396	0.32233192
ShSirt1_vs_ShScrambled	P62911	P62911	Rpl32	19951	-0.288542624	0.13943288	0.32240565
ShSirt1_vs_ShScrambled	O08811	O08811	Ercc2	13871	0.336902806	0.13950884	0.32240565
ShSirt1_vs_ShScrambled	Q60767	Q60767	Ly75	17076	-0.790855009	0.13953282	0.32240565
ShSirt1_vs_ShScrambled	P97770	P97770	Thumpd3	14911	0.736713977	0.13962078	0.32240565
ShSirt1_vs_ShScrambled	Q9Z2C5	Q9Z2C5	Mtm1	17772	0.311727857	0.1396897	0.32240565
ShSirt1_vs_ShScrambled	Q61749	Q61749	Eif2b4	13667	0.218080625	0.13971622	0.32240565
ShSirt1_vs_ShScrambled	Q91W92	Q91W92	Cdc42ep1	104445	-0.464462856	0.13977578	0.32240565
ShSirt1_vs_ShScrambled	Q99N87	Q99N87	Mrps5	77721	-0.264672263	0.13999366	0.32274331
ShSirt1_vs_ShScrambled	Q2HXL6	Q2HXL6	Edem3	66967	-0.757460915	0.14036681	0.32343837
ShSirt1_vs_ShScrambled	P47738	P47738	Aldh2	11669	0.214799836	0.14046733	0.32350486
ShSirt1_vs_ShScrambled	Q9D358	Q9D358	Acp1	11431	0.257261829	0.14061011	0.32366856
ShSirt1_vs_ShScrambled	O88844	O88844	Idh1	15926	-0.223266031	0.14102652	0.32432834
ShSirt1_vs_ShScrambled	Q9JLR1	Q9JLR1	Sec61a2	57743	1.315933343	0.14109653	0.32432834
ShSirt1_vs_ShScrambled	Q8C5Q4	Q8C5Q4	Grsf1	231413	0.216221929	0.14114151	0.32432834
ShSirt1_vs_ShScrambled	Q921N7	Q921N7	Tmem70	70397	-0.470101822	0.14118414	0.32432834
ShSirt1_vs_ShScrambled	Q7TMY4	Q7TMY4	Thoc7	66231	1.384219827	0.14139259	0.32464198
ShSirt1_vs_ShScrambled	Q80ZS3	Q80ZS3	Mrps26	99045	-0.740398665	0.14158799	0.32492535
ShSirt1_vs_ShScrambled	P41241	P41241	Csk	12988	-0.543460258	0.14190454	0.32530983
ShSirt1_vs_ShScrambled	P27612	P27612	Plaa	18786	-0.312910155	0.141952	0.32530983
ShSirt1_vs_ShScrambled	Q8VC31	Q8VC31	Ccdc9	243846	-1.168803364	0.14197173	0.32530983
ShSirt1_vs_ShScrambled	P60122	P60122	Ruvbl1	56505	0.350287942	0.14236985	0.32605657
ShSirt1_vs_ShScrambled	Q91YY4	Q91YY4	Atpaf2	246782	0.340779682	0.14254821	0.32629951
ShSirt1_vs_ShScrambled	O35609	O35609	Scamp3	24045	-0.385649694	0.14268569	0.32644866
ShSirt1_vs_ShScrambled	Q62130	Q62130	Ptpn14	19250	-1.962732145	0.14289444	0.32676064
ShSirt1_vs_ShScrambled	Q8BTY8	Q8BTY8	Scfd2	212986	0.282736011	0.14306422	0.32698324
ShSirt1_vs_ShScrambled	P70336	P70336	Rock2	19878	-0.310876444	0.14347551	0.32771304
ShSirt1_vs_ShScrambled	Q8VEJ9	Q8VEJ9	Vps4a	116733	-0.314146293	0.14352873	0.32771304
ShSirt1_vs_ShScrambled	Q05512	Q05512	Mark2	13728	0.944373981	0.14363363	0.32778675
ShSirt1_vs_ShScrambled	P14148	P14148	Rpl7	19989	-0.276048087	0.14373104	0.32784332
ShSirt1_vs_ShScrambled	Q8R326	Q8R326	Pspc1	66645	0.256777077	0.14425053	0.32886208
ShSirt1_vs_ShScrambled	Q3UMW7	Q3UMW7	Mapkapk3	102626	0.801351723	0.14442637	0.32909675
ShSirt1_vs_ShScrambled	Q8VE37	Q8VE37	Rcc1	100088	0.256799438	0.14451379	0.3291298
ShSirt1_vs_ShScrambled	Q80T85	Q80T85	Dcaf5	320808	-0.675515334	0.14477265	0.32937126
ShSirt1_vs_ShScrambled	Q8QZV7	Q8QZV7	Asun	71177	0.49844165	0.14482683	0.32937126
ShSirt1_vs_ShScrambled	Q9D1N9	Q9D1N9	Mrpl21	353242	-0.284210123	0.14491729	0.32937126
ShSirt1_vs_ShScrambled	Q8VIJ6	Q8VIJ6	Sfpq	71514	0.23405547	0.1449697	0.32937126
ShSirt1_vs_ShScrambled	Q60930	Q60930	Vdac2	22334	0.22463216	0.14498465	0.32937126
ShSirt1_vs_ShScrambled	P62320	P62320	Snrpd3	67332	0.343854373	0.14526058	0.32983212
ShSirt1_vs_ShScrambled	Q5SSL4	Q5SSL4	Abr	109934	1.590959874	0.14584398	0.33099032
ShSirt1_vs_ShScrambled	Q8BP48	Q8BP48	Metap1	75624	0.428455	0.14635805	0.33199007
ShSirt1_vs_ShScrambled	P47809	P47809	Map2k4	26398	-0.49858062	0.14660186	0.33235067
ShSirt1_vs_ShScrambled	Q9Z129	Q9Z129	Recql	19691	0.407680713	0.14666427	0.33235067
ShSirt1_vs_ShScrambled	Q921F2	Q921F2	Tardbp	230908	0.256775279	0.14673924	0.33235371
ShSirt1_vs_ShScrambled	Q9D3E6	Q9D3E6	Stag1	20842	0.777766332	0.14684222	0.33237496
ShSirt1_vs_ShScrambled	Q9DC71	Q9DC71	Mrps15	66407	-0.324196402	0.14689589	0.33237496
ShSirt1_vs_ShScrambled	Q61810	Q61810	Ltbp3	16998	0.282967076	0.14704923	0.33255523
ShSirt1_vs_ShScrambled	Q5SSE9	Q5SSE9	Abca13	268379	0.765114491	0.14716562	0.33265177
ShSirt1_vs_ShScrambled	P35564	P35564	Canx	12330	-0.226081129	0.14726994	0.33272098
ShSirt1_vs_ShScrambled	Q8VD76	Q8VD76	Gtf2h3	209357	0.763279184	0.14773148	0.33350636
ShSirt1_vs_ShScrambled	P34884	P34884	Mif	17319	0.539477447	0.14776534	0.33350636
ShSirt1_vs_ShScrambled	Q9DA03	Q9DA03	Lyrm7	75530	-0.762693651	0.14791264	0.33367199
ShSirt1_vs_ShScrambled	Q9CYH6	Q9CYH6	Rrs1	59014	0.638266583	0.14827074	0.33431274
ShSirt1_vs_ShScrambled	Q4VAA7	Q4VAA7	Snx33	235406	-0.864303907	0.14898858	0.33576358
ShSirt1_vs_ShScrambled	P51480	P51480	Cdkn2a	12578	-0.328691149	0.14953555	0.33682809
ShSirt1_vs_ShScrambled	Q9DC61	Q9DC61	Pmpca	66865	-0.231977804	0.14973203	0.33710243
ShSirt1_vs_ShScrambled	Q9CWE0	Q9CWE0	Mtfr1l	76824	0.715760501	0.14988667	0.33728236
ShSirt1_vs_ShScrambled	Q9EQN3	Q9EQN3	Tsc22d4	78829	0.431303681	0.15004228	0.33746429
ShSirt1_vs_ShScrambled	Q920R0	Q920R0	Als2	74018	-0.755415701	0.15018992	0.33762814
ShSirt1_vs_ShScrambled	Q91VY9	Q91VY9	Znf622	52521	0.799318984	0.15050759	0.33817385

ShSirt1_vs_ShScrambled	P68254	P68254	Ywhaq	22630	-0.249090922	0.15083858	0.33860313
ShSirt1_vs_ShScrambled	Q9JKL4	Q9JKL4	Ndufaf3	66706	0.753338469	0.15084867	0.33860313
ShSirt1_vs_ShScrambled	Q9DBR0	Q9DBR0	Akap8	56399	0.277129528	0.15159604	0.33980542
ShSirt1_vs_ShScrambled	P56542	P56542	Dnase2	13423	-0.535284199	0.15159708	0.33980542
ShSirt1_vs_ShScrambled	Q9CY27	Q9CY27	Tecr	106529	-0.396530734	0.15161013	0.33980542
ShSirt1_vs_ShScrambled	Q8VED9	Q8VED9	Lgalsl	216551	-0.39972194	0.15217074	0.34089267
ShSirt1_vs_ShScrambled	P62814	P62814	Atp6v1b2	11966	-0.222091105	0.1522524	0.34090641
ShSirt1_vs_ShScrambled	Q8K2M0	Q8K2M0	Mrpl38	60441	-0.197438477	0.15243633	0.34105112
ShSirt1_vs_ShScrambled	Q91VR5	Q91VR5	Ddx1	104721	-0.189759863	0.15250973	0.34105112
ShSirt1_vs_ShScrambled	Q3U4I7	Q3U4I7	Pyroxd2	74580	0.432851399	0.15254369	0.34105112
ShSirt1_vs_ShScrambled	Q80Y17	Q80Y17	Llgl1	16897	0.769069612	0.15290996	0.34170077
ShSirt1_vs_ShScrambled	Q9Z1Z2	Q9Z1Z2	Strap	20901	-0.390401758	0.15302575	0.34176216
ShSirt1_vs_ShScrambled	Q9EPQ7	Q9EPQ7	Stard5	170460	-0.41182365	0.15312182	0.34176216
ShSirt1_vs_ShScrambled	Q9ESK9	Q9ESK9	Rb1cc1	12421	2.662556694	0.15322304	0.34176216
ShSirt1_vs_ShScrambled	P03930	P03930	Mtatp8	17706	-0.73881672	0.15324028	0.34176216
ShSirt1_vs_ShScrambled	E9Q1P8	E9Q1P8	Irf2bp2	270110	0.311572573	0.1537307	0.3426866
ShSirt1_vs_ShScrambled	P42932	P42932	Cct8	12469	0.266363214	0.15469834	0.34458957
ShSirt1_vs_ShScrambled	Q6PE01	Q6PE01	Snrnp40	66585	0.461441797	0.15473731	0.34458957
ShSirt1_vs_ShScrambled	Q8BMD8	Q8BMD8	Slc25a24	229731	0.511669083	0.1548826	0.34458957
ShSirt1_vs_ShScrambled	Q9DAX9	Q9DAX9	Appbp2	66884	0.961582039	0.15493107	0.34458957
ShSirt1_vs_ShScrambled	Q8BVU5	Q8BVU5	Nudt9	74167	0.371612423	0.15496607	0.34458957
ShSirt1_vs_ShScrambled	P97473	P97473	Tarbp2	21357	0.327554069	0.15514125	0.34466135
ShSirt1_vs_ShScrambled	Q8BYW9	Q8BYW9	Eogt	101351	0.490171771	0.15515105	0.34466135
ShSirt1_vs_ShScrambled	Q8R1S0	Q8R1S0	Coq6	217707	-0.236834935	0.15535015	0.34481136
ShSirt1_vs_ShScrambled	Q2YDW2	Q2YDW2	Msto1	229524	0.221173733	0.15537136	0.34481136
ShSirt1_vs_ShScrambled	Q8K0V4	Q8K0V4	Cnot3	232791	0.391514889	0.15553262	0.3449594
ShSirt1_vs_ShScrambled	Q8R4H9	Q8R4H9	Slc30a5	69048	0.524134161	0.1555909	0.3449594
ShSirt1_vs_ShScrambled	P55096	P55096	Abcd3	19299	-0.31119667	0.15608591	0.345887
ShSirt1_vs_ShScrambled	Q9DD18	Q9DD18	Dtd1	66044	0.567534815	0.156441	0.34650376
ShSirt1_vs_ShScrambled	Q8CFE4	Q8CFE4	Scyl2	213326	-0.561655725	0.15657619	0.34663312
ShSirt1_vs_ShScrambled	Q91WC9	Q91WC9	Daglb	231871	-0.735173089	0.15678078	0.34691591
ShSirt1_vs_ShScrambled	Q8K0T0	Q8K0T0	Rtn1	104001	-0.536816748	0.15694634	0.34711209
ShSirt1_vs_ShScrambled	Q8R3Y8	Q8R3Y8	Irf2bp1	272359	0.477761984	0.15705591	0.34718432
ShSirt1_vs_ShScrambled	G5E897	G5E897	Kdelc2	68304	-0.308113324	0.15762435	0.3481856
ShSirt1_vs_ShScrambled	E9Q3L2	E9Q3L2	Pi4ka	224020	0.472113213	0.15766313	0.3481856
ShSirt1_vs_ShScrambled	Q58NB6	Q58NB6	Dhrs9	241452	-0.801727465	0.15780192	0.3483217
ShSirt1_vs_ShScrambled	O88685	O88685	Psmc3	19182	-0.261478548	0.15790502	0.34837893
ShSirt1_vs_ShScrambled	Q9D023	Q9D023	Mpc2	70456	-0.6733778	0.15799696	0.34841148
ShSirt1_vs_ShScrambled	Q68FF6	Q68FF6	Git1	216963	0.299022437	0.15826365	0.34866291
ShSirt1_vs_ShScrambled	Q8K310	Q8K310	Matr3	17184	-0.216212921	0.15826546	0.34866291
ShSirt1_vs_ShScrambled	B2RXR6	B2RXR6	Ankrd44	329154	0.231105404	0.15837474	0.34873344
ShSirt1_vs_ShScrambled	Q61210	Q61210	Arhgef1	16801	0.423566102	0.15897695	0.34987665
ShSirt1_vs_ShScrambled	P61080	P61080	Ube2d1	216080	1.147841363	0.15909513	0.34987665
ShSirt1_vs_ShScrambled	P83887	P83887;Q8V	Tubg1		0.370839985	0.15912644	0.34987665
ShSirt1_vs_ShScrambled	Q3UMR5	Q3UMR5	Mcu	215999	0.222223642	0.15932975	0.35015311
ShSirt1_vs_ShScrambled	Q9JIF7	Q9JIF7	Copb1	70349	0.191152663	0.15955438	0.35047613
ShSirt1_vs_ShScrambled	Q9QUI1	Q9QUI1	Fam89b	17826	0.264911177	0.15965574	0.35052822
ShSirt1_vs_ShScrambled	Q8CAS9	Q8CAS9	Parp9	80285	1.945749761	0.16010362	0.35120971
ShSirt1_vs_ShScrambled	Q9D1I5	Q9D1I5	Mcee	73724	-0.440912406	0.16012175	0.35120971
ShSirt1_vs_ShScrambled	Q31125	Q31125	Slc39a7	14977	0.414553769	0.16042775	0.35163634
ShSirt1_vs_ShScrambled	Q8BVG4	Q8BVG4	Dpp9	224897	-0.196812959	0.16058522	0.35163634
ShSirt1_vs_ShScrambled	P62141	P62141	Ppp1cb	19046	0.372257939	0.1605897	0.35163634
ShSirt1_vs_ShScrambled	O89112	O89112	Lancl1	14768	0.569048021	0.16069145	0.35163634
ShSirt1_vs_ShScrambled	O88413	O88413	Tulp3	22158	-0.416145635	0.16070575	0.35163634
ShSirt1_vs_ShScrambled	Q810J8	Q810J8	Zfyve1	217695	-0.423366724	0.16080495	0.35168292
ShSirt1_vs_ShScrambled	P70677	P70677	Casp3	12367	-0.199435146	0.1609739	0.35188192
ShSirt1_vs_ShScrambled	Q8CHC4	Q8CHC4	Synj1		0.432192268	0.16107852	0.3519402
ShSirt1_vs_ShScrambled	Q60972	Q60972	Rbbp4	19646	0.211426397	0.16134838	0.3523483
ShSirt1_vs_ShScrambled	P70460	P70460	Vasp	22323	0.2141246	0.16145285	0.3523483
ShSirt1_vs_ShScrambled	Q99JY9	Q99JY9	Actr3	74117	-0.214276587	0.16164155	0.3523483
ShSirt1_vs_ShScrambled	Q9DB60	Q9DB60	Fam213b	66469	-0.271597874	0.16166855	0.3523483
ShSirt1_vs_ShScrambled	Q78RX3	Q78RX3	Smim12	80284	-0.713591157	0.16170698	0.3523483
ShSirt1_vs_ShScrambled	P00416	P00416	mt.Co3	17710	-0.411958312	0.16174455	0.3523483

ShSirt1_vs_ShScrambled	Q9CXJ4	Q9CXJ4	Abcb8	74610	-0.244997824	0.16188666	0.3523483
ShSirt1_vs_ShScrambled	Q9CR09	Q9CR09	Ufc1	66155	1.008888083	0.16190331	0.3523483
ShSirt1_vs_ShScrambled	Q6NXH2	Q6NXH2	Manea	242362	0.720038641	0.16196782	0.3523483
ShSirt1_vs_ShScrambled	Q8K297	Q8K297	Colgalt1	234407	-0.21682512	0.16216326	0.35260354
ShSirt1_vs_ShScrambled	P97371	P97371	Psme1	19186	0.277128695	0.1623011	0.35273334
ShSirt1_vs_ShScrambled	Q8C166	Q8C166	Cpne1	266692	0.337691438	0.16255525	0.35311568
ShSirt1_vs_ShScrambled	Q6NSR3	Q6NSR3	Adck2	57869	0.567590873	0.16332274	0.35461225
ShSirt1_vs_ShScrambled	Q7TMF3	Q7TMF3	Ndufa12	66414	-0.551187968	0.16346033	0.35474011
ShSirt1_vs_ShScrambled	Q61686	Q61686	Cbx5	12419	0.374623132	0.1635388	0.35474011
ShSirt1_vs_ShScrambled	Q6ZPU9	Q6ZPU9	Kbp	72320	0.599640369	0.16378376	0.35503164
ShSirt1_vs_ShScrambled	Q3U7R1	Q3U7R1	Esyt1	23943	-0.205138658	0.16388043	0.35503164
ShSirt1_vs_ShScrambled	Q9JHP7	Q9JHP7	Kdelc1	72050	-0.406321485	0.16390916	0.35503164
ShSirt1_vs_ShScrambled	Q922H4	Q922H4	Gmppa	69080	0.41064486	0.16428272	0.35567012
ShSirt1_vs_ShScrambled	Q3U9G9	Q3U9G9	Lbr	98386	0.203293626	0.16443903	0.35577174
ShSirt1_vs_ShScrambled	P15208	P15208	Insr	16337	-0.56740775	0.16448729	0.35577174
ShSirt1_vs_ShScrambled	P11609	P11609;P11(	Cd1d1		-0.61254529	0.16494721	0.35659564
ShSirt1_vs_ShScrambled	Q9Z0R9	Q9Z0R9	Fads2	56473	-0.326067734	0.16506894	0.35668798
ShSirt1_vs_ShScrambled	P27773	P27773	Pdia3	14827	-0.274509201	0.16543553	0.35730908
ShSirt1_vs_ShScrambled	Q8CCK0	Q8CCK0	H2afy2	404634	0.228812494	0.16596196	0.35822215
ShSirt1_vs_ShScrambled	Q3UQ44	Q3UQ44	Iqgap2	544963	-0.68201715	0.166017	0.35822215
ShSirt1_vs_ShScrambled	Q78JW9	Q78JW9	Ubfd1	28018	0.32348563	0.16653227	0.35894817
ShSirt1_vs_ShScrambled	Q9ES74	Q9ES74	Nek7	59125	0.250548828	0.16657218	0.35894817
ShSirt1_vs_ShScrambled	P03921	P03921	Mtnd5	17721	-0.707084629	0.16659388	0.35894817
ShSirt1_vs_ShScrambled	P53026	P53026	Rpl10a		-0.256323891	0.16674438	0.35894817
ShSirt1_vs_ShScrambled	Q8R151	Q8R151	Znfx1	98999	-0.560212332	0.16675107	0.35894817
ShSirt1_vs_ShScrambled	Q9QY06	Q9QY06	Myo9b		-0.353980509	0.16703865	0.35939583
ShSirt1_vs_ShScrambled	Q9JJK2	Q9JJK2	Lancl2	71835	0.686430964	0.1673237	0.35967464
ShSirt1_vs_ShScrambled	Q99LD4	Q99LD4	Gps1		0.190666791	0.16738158	0.35967464
ShSirt1_vs_ShScrambled	P52760	P52760	Hrsp12	15473	0.200539357	0.16740727	0.35967464
ShSirt1_vs_ShScrambled	P70372	P70372	Elavl1	15568	-0.277708202	0.16755243	0.35977906
ShSirt1_vs_ShScrambled	Q91VN4	Q91VN4	Chchd6	66098	0.35888894	0.16773415	0.35977906
ShSirt1_vs_ShScrambled	Q921H8	Q921H8;Q8V	Acaa1a		-0.24011771	0.16777353	0.35977906
ShSirt1_vs_ShScrambled	P63276	P63276	Rps17	20068	0.250993811	0.16783714	0.35977906
ShSirt1_vs_ShScrambled	P70302	P70302	Stim1	20866	-1.319992707	0.16785439	0.35977906
ShSirt1_vs_ShScrambled	O70435	O70435	Psma3	19167	-0.445276102	0.16794773	0.35980828
ShSirt1_vs_ShScrambled	A2AAY5	A2AAY5	Sh3pxd2b	268396	-0.409310816	0.16821048	0.35991553
ShSirt1_vs_ShScrambled	Q9Z1M4	Q9Z1M4	Rps6kb2	58988	0.538133749	0.16827203	0.35991553
ShSirt1_vs_ShScrambled	Q7TQI3	Q7TQI3	Otub1	107260	0.224903471	0.16837402	0.35991553
ShSirt1_vs_ShScrambled	Q8VDG7	Q8VDG7	Pafah2	100163	-0.801943894	0.16849713	0.35991553
ShSirt1_vs_ShScrambled	P62137	P62137	Ppp1ca	19045	0.291158011	0.16850676	0.35991553
ShSirt1_vs_ShScrambled	Q8CI59	Q8CI59	Steap3	68428	0.197622136	0.16853328	0.35991553
ShSirt1_vs_ShScrambled	P59997	P59997	Kdm2a	225876	0.225948915	0.16855592	0.35991553
ShSirt1_vs_ShScrambled	P97390	P97390	Vps45	22365	0.275148211	0.16880843	0.36022468
ShSirt1_vs_ShScrambled	Q9CXY9	Q9CXY9	Pigk	329777	0.594858764	0.16893975	0.36022468
ShSirt1_vs_ShScrambled	Q8K268	Q8K268	Abcf3	27406	0.221288099	0.16894011	0.36022468
ShSirt1_vs_ShScrambled	Q8R081	Q8R081	Hnrnpl	15388	0.210922457	0.16910165	0.3603989
ShSirt1_vs_ShScrambled	Q01341	Q01341	Adcy6	11512	-0.279650506	0.16962367	0.36134084
ShSirt1_vs_ShScrambled	Q8R323	Q8R323	Rfc3	69263	0.400318989	0.16981558	0.36157901
ShSirt1_vs_ShScrambled	O08663	O08663	Metap2	56307	0.311311139	0.1702127	0.36225371
ShSirt1_vs_ShScrambled	Q8JZU2	Q8JZU2	Slc25a1	13358	-0.185139086	0.17119852	0.36418007
ShSirt1_vs_ShScrambled	Q8BKC8	Q8BKC8	Pi4kb	107650	1.878931285	0.1713995	0.36443586
ShSirt1_vs_ShScrambled	P06151	P06151	Ldha	16828	0.295343031	0.17232297	0.36612122
ShSirt1_vs_ShScrambled	P07901	P07901	Hsp90aa1	15519	0.198652079	0.17238705	0.36612122
ShSirt1_vs_ShScrambled	Q8C6B9	Q8C6B9	Rps19bp1	66538	-0.242929561	0.17243547	0.36612122
ShSirt1_vs_ShScrambled	Q8CI11	Q8CI11	Gnl3	30877	0.335508276	0.17273402	0.36658269
ShSirt1_vs_ShScrambled	Q62426	Q62426	Cstb	13014	0.28492912	0.17287037	0.36669966
ShSirt1_vs_ShScrambled	P11983	P11983	Tcp1	21454	0.212787198	0.17321273	0.36712029
ShSirt1_vs_ShScrambled	Q9CQE1	Q9CQE1	Nipsnap3b	66536	0.25149794	0.17323133	0.36712029
ShSirt1_vs_ShScrambled	Q60737	Q60737	Csnk2a1	12995	0.187628095	0.17383505	0.36820724
ShSirt1_vs_ShScrambled	Q3UZ39	Q3UZ39	Lrrfip1	16978	-0.272265388	0.17390736	0.36820724
ShSirt1_vs_ShScrambled	Q9Z277	Q9Z277	Baz1b	22385	0.358952371	0.17416932	0.36848271
ShSirt1_vs_ShScrambled	Q9CQA5	Q9CQA5	Med4	67381	0.776679369	0.17420073	0.36848271
ShSirt1_vs_ShScrambled	Q4KMM3	Q4KMM3	Oxr1	170719	-0.25021624	0.17452885	0.36885026

ShSirt1_vs_ShScrambled	Q8VE18	Q8VE18	Smg8	74133	-0.296994573	0.17456276	0.36885026
ShSirt1_vs_ShScrambled	Q9D020	Q9D020	Nt5c3a	107569	0.454849748	0.17461963	0.36885026
ShSirt1_vs_ShScrambled	Q8CG72	Q8CG72	Adprhl2	100206	0.241151303	0.17523612	0.36997934
ShSirt1_vs_ShScrambled	P54728	P54728	Rad23b	19359	-0.218627021	0.17609466	0.37161818
ShSirt1_vs_ShScrambled	P47915	P47915	Rpl29	19944	-0.279169086	0.17626336	0.37177294
ShSirt1_vs_ShScrambled	P70349	P70349	Hint1	15254	0.234793014	0.17633271	0.37177294
ShSirt1_vs_ShScrambled	Q9EQU5	Q9EQU5	Set	56086	-0.388511041	0.17652297	0.37200032
ShSirt1_vs_ShScrambled	Q91YJ5	Q91YJ5	Mtif2	76784	0.602002206	0.17687646	0.37257133
ShSirt1_vs_ShScrambled	Q91XL9	Q91XL9	Osbpl1a	64291	-0.303512088	0.17711587	0.37290161
ShSirt1_vs_ShScrambled	P12970	P12970	Rpl7a	27176	-0.215884792	0.17744402	0.37341832
ShSirt1_vs_ShScrambled	Q3TDD9	Q3TDD9	Ppp1r21	73825	-0.180400763	0.17799389	0.37440095
ShSirt1_vs_ShScrambled	Q9D0S9	Q9D0S9	Hint2	68917	-0.319987682	0.17826627	0.3747762
ShSirt1_vs_ShScrambled	Q9Z315	Q9Z315	Sart1	20227	0.473412196	0.17833834	0.3747762
ShSirt1_vs_ShScrambled	Q60967	Q60967	Papss1	23971	0.228289281	0.17856029	0.375068
ShSirt1_vs_ShScrambled	Q8BHN1	Q8BHN1	Txlng	353170	0.262034749	0.17879349	0.37517723
ShSirt1_vs_ShScrambled	Q9DBE0	Q9DBE0	Csad	246277	0.307848935	0.17907401	0.37517723
ShSirt1_vs_ShScrambled	Q8BI72	Q8BI72	Cdkn2aip	70925	0.835365782	0.17907729	0.37517723
ShSirt1_vs_ShScrambled	Q9QZS3	Q9QZS3	Numb	18222	1.252142843	0.17911051	0.37517723
ShSirt1_vs_ShScrambled	Q8C050	Q8C050	Rps6ka5	73086	0.67427033	0.17913795	0.37517723
ShSirt1_vs_ShScrambled	Q91VH2	Q91VH2	Snx9	66616	0.313804429	0.17919562	0.37517723
ShSirt1_vs_ShScrambled	O88545	O88545	Cops6	26893	0.352353662	0.17920555	0.37517723
ShSirt1_vs_ShScrambled	Q1HFZ0	Q1HFZ0	Nsun2	28114	0.223224584	0.17929191	0.37517723
ShSirt1_vs_ShScrambled	Q9R1P3	Q9R1P3	Psmb2	26445	0.232570086	0.17936031	0.37517723
ShSirt1_vs_ShScrambled	P70122	P70122	Sbds	66711	0.886084369	0.17955848	0.37541777
ShSirt1_vs_ShScrambled	Q9CQS2	Q9CQS2	Nop10	66181	-0.404443378	0.17979517	0.37568277
ShSirt1_vs_ShScrambled	Q9CR13	Q9CR13	Q9CR13	66117	0.323133913	0.17985168	0.37568277
ShSirt1_vs_ShScrambled	P58058	P58058	Nadk	192185	0.376805396	0.17998902	0.37579577
ShSirt1_vs_ShScrambled	Q497V5	Q497V5	Srbd1		-0.886443681	0.18011118	0.37587696
ShSirt1_vs_ShScrambled	P54071	P54071	Idh2	269951	0.213617064	0.18107696	0.37763717
ShSirt1_vs_ShScrambled	O88393	O88393	Tgfbr3	21814	-0.308745836	0.18113672	0.37763717
ShSirt1_vs_ShScrambled	P56376	P56376	Acyp1	66204	0.321889266	0.18120561	0.37763717
ShSirt1_vs_ShScrambled	Q3UM45	Q3UM45	Ppp1r7	66385	0.171532236	0.18177596	0.37865099
ShSirt1_vs_ShScrambled	Q61234	Q61234	Snta1		0.765735275	0.18234402	0.37959133
ShSirt1_vs_ShScrambled	P54726	P54726	Rad23a	19358	0.222012978	0.18239557	0.37959133
ShSirt1_vs_ShScrambled	Q8VHK1	Q8VHK1	Caskin2	140721	-0.559471155	0.18298123	0.38063468
ShSirt1_vs_ShScrambled	Q9QYY0	Q9QYY0	Gab1	14388	-1.03802456	0.18319204	0.38089769
ShSirt1_vs_ShScrambled	Q8BZ20	Q8BZ20	Parp12	243771	-0.874481277	0.18329269	0.38093148
ShSirt1_vs_ShScrambled	Q6ZQB6	Q6ZQB6	Ppip5k2	227399	0.99948866	0.18354775	0.38128603
ShSirt1_vs_ShScrambled	Q8JZN7	Q8JZN7	Rhot2	214952	-0.474658797	0.18377737	0.38158742
ShSirt1_vs_ShScrambled	O35643	O35643	Ap1b1	11764	-0.243007496	0.18408462	0.38204965
ShSirt1_vs_ShScrambled	Q91YJ2	Q91YJ2	Snx4	69150	0.264763457	0.18433137	0.38236445
ShSirt1_vs_ShScrambled	Q8VE62	Q8VE62	Paip1	218693	-0.756494707	0.18446461	0.38236445
ShSirt1_vs_ShScrambled	Q9CZN8	Q9CZN8	Qrsl1	76563	-0.520533776	0.18449042	0.38236445
ShSirt1_vs_ShScrambled	F6ZDS4	F6ZDS4	Tpr	108989	0.22943704	0.18461622	0.38244958
ShSirt1_vs_ShScrambled	Q60722	Q60722	Tcf4	21413	0.660612413	0.18473286	0.38249144
ShSirt1_vs_ShScrambled	Q91YR9	Q91YR9	Ptgr1	67103	-0.420887794	0.1848059	0.38249144
ShSirt1_vs_ShScrambled	Q9JJZ2	Q9JJZ2	Tuba8	53857	-0.660131003	0.1849343	0.38258178
ShSirt1_vs_ShScrambled	Q78YY6	Q78YY6	Dnajc15	66148	-0.340453733	0.18510625	0.38276209
ShSirt1_vs_ShScrambled	Q922E6	Q922E6	Fastkd2	75619	-0.866134229	0.18547328	0.38334542
ShSirt1_vs_ShScrambled	Q99KG3	Q99KG3	Rbm10	236732	0.279401719	0.1856197	0.38346094
ShSirt1_vs_ShScrambled	Q811D0	Q811D0	Dlg1	13383	0.281886467	0.18570296	0.38346094
ShSirt1_vs_ShScrambled	Q8CIF4	Q8CIF4	Btd	26363	0.555639051	0.18578402	0.38346094
ShSirt1_vs_ShScrambled	P30416	P30416	Fkbp4	14228	0.196883634	0.18605057	0.38383559
ShSirt1_vs_ShScrambled	Q8R5A0	Q8R5A0	Smyd2	226830	1.076204279	0.18621509	0.38399951
ShSirt1_vs_ShScrambled	Q9CR60	Q9CR60	Golt1b	66964	-0.32090056	0.18655526	0.38452531
ShSirt1_vs_ShScrambled	P70452	P70452	Stx4	20909	0.393866867	0.18683644	0.38474564
ShSirt1_vs_ShScrambled	Q64133	Q64133	Maoa	17161	-0.429467728	0.18687053	0.38474564
ShSirt1_vs_ShScrambled	Q3TA38	Q3TA38	Tmem120b	330189	-0.475271011	0.18693739	0.38474564
ShSirt1_vs_ShScrambled	Q9CPR5	Q9CPR5	Mrpl15	27395	-0.179460455	0.18702765	0.38474564
ShSirt1_vs_ShScrambled	Q9D924	Q9D924	Isca1	69046	0.615419476	0.18716243	0.38474564
ShSirt1_vs_ShScrambled	Q3U2A8	Q3U2A8	Vars2	68915	0.270048985	0.18717355	0.38474564
ShSirt1_vs_ShScrambled	P59759	P59759	Mkl2	239719	-0.419992337	0.18731381	0.38485869
ShSirt1_vs_ShScrambled	Q8CHP5	Q8CHP5	Wibg	78428	0.4905531	0.18749346	0.38499365

ShSirt1_vs_ShScrambled	Q8VDP3	Q8VDP3	Mical1	171580	0.286474638	0.18759109	0.38499365
ShSirt1_vs_ShScrambled	Q6ZPJ0	Q6ZPJ0	Tex2	21763	-0.344992754	0.18763536	0.38499365
ShSirt1_vs_ShScrambled	Q8BJ05	Q8BJ05	Zc3h14	75553	0.321156026	0.18789859	0.38535859
ShSirt1_vs_ShScrambled	Q80VP1	Q80VP1	Epn1	13854	0.518296747	0.18806959	0.38553413
ShSirt1_vs_ShScrambled	P70288	P70288	Hdac2	15182	0.204323034	0.18853135	0.38630527
ShSirt1_vs_ShScrambled	P42230	P42230	Stat5a	20850	-0.298216922	0.1888581	0.38654993
ShSirt1_vs_ShScrambled	Q64674	Q64674	Srm	20810	0.19244347	0.1888761	0.38654993
ShSirt1_vs_ShScrambled	Q8R1X6	Q8R1X6	Spg20	229285	0.327381422	0.18890765	0.38654993
ShSirt1_vs_ShScrambled	B1AZP2	B1AZP2	Dlgap4	228836	0.700241106	0.18906828	0.38670332
ShSirt1_vs_ShScrambled	Q9CQV4	Q9CQV4	Fam134c	67998	-0.347127978	0.1897248	0.38787035
ShSirt1_vs_ShScrambled	Q8BKG3	Q8BKG3	Ptk7	71461	-0.225505966	0.19016408	0.38859243
ShSirt1_vs_ShScrambled	Q9DAM7	Q9DAM7	Tmem263	103266	0.628336	0.19045593	0.38885494
ShSirt1_vs_ShScrambled	Q9DAK9	Q9DAK9	Phpt1	75454	0.24825858	0.19051598	0.38885494
ShSirt1_vs_ShScrambled	P35278	P35278	Rab5c	19345	-0.185268199	0.19055098	0.38885494
ShSirt1_vs_ShScrambled	Q91WQ3	Q91WQ3	Yars	107271	0.20436571	0.1907525	0.38900017
ShSirt1_vs_ShScrambled	Q8R313	Q8R313	Exoc6		0.742639133	0.1907945	0.38900017
ShSirt1_vs_ShScrambled	Q9D2X0	Q9D2X0	Ankrd39	109346	0.64598962	0.19098443	0.38910127
ShSirt1_vs_ShScrambled	Q8VC65	Q8VC65	Nrm	106582	-0.588747521	0.19101648	0.38910127
ShSirt1_vs_ShScrambled	P63024	P63024	Vamp3	22319	-0.355782813	0.19137889	0.38949742
ShSirt1_vs_ShScrambled	Q9DCU6	Q9DCU6	Mrpl4	66163	-0.226955911	0.19145629	0.38949742
ShSirt1_vs_ShScrambled	O08709	O08709	Prdx6	11758	0.258571394	0.19154259	0.38949742
ShSirt1_vs_ShScrambled	A2ATU0	A2ATU0	Dhtkd1	209692	0.221279275	0.19155611	0.38949742
ShSirt1_vs_ShScrambled	Q99JX7	Q99JX7	Nxf1	53319	0.336703582	0.19184113	0.38974485
ShSirt1_vs_ShScrambled	Q9Z0Y1	Q9Z0Y1	Dctn3	53598	-0.290567918	0.19187115	0.38974485
ShSirt1_vs_ShScrambled	Q9CQV7	Q9CQV7	Dnajc19	67713	-0.521646191	0.19212331	0.38974485
ShSirt1_vs_ShScrambled	Q69ZC8	Q69ZC8	Gpalpp1	67467	-2.649096719	0.19219199	0.38974485
ShSirt1_vs_ShScrambled	P51410	P51410	Rpl9	20005	0.194716208	0.19224693	0.38974485
ShSirt1_vs_ShScrambled	Q922K9	Q922K9	Frk	14302	-0.642963863	0.19231301	0.38974485
ShSirt1_vs_ShScrambled	P27641	P27641	Xrcc5	22596	-0.642894534	0.1923436	0.38974485
ShSirt1_vs_ShScrambled	P56960	P56960	Exosc10	50912	0.273891874	0.19236852	0.38974485
ShSirt1_vs_ShScrambled	Q3UDE2	Q3UDE2	Ttll12	223723	0.356452586	0.1924899	0.3898158
ShSirt1_vs_ShScrambled	Q61033	Q61033	Tmpo.1	21917	0.505901861	0.19260254	0.389869
ShSirt1_vs_ShScrambled	Q8BP56	Q8BP56	Athl1	212974	-0.405511714	0.19272507	0.38990622
ShSirt1_vs_ShScrambled	Q8BKC5	Q8BKC5	Ipo5	70572	0.26086844	0.19279368	0.38990622
ShSirt1_vs_ShScrambled	Q4VA53	Q4VA53	Pds5b	100710	0.373612726	0.19352344	0.39120682
ShSirt1_vs_ShScrambled	Q5H8C4	Q5H8C4	Vps13a	271564	-0.281193065	0.19395604	0.3919058
ShSirt1_vs_ShScrambled	Q3UHQ6	Q3UHQ6	Dopey2	70028	0.247190586	0.1943542	0.39239968
ShSirt1_vs_ShScrambled	Q569Z5	Q569Z5	Ddx46	212880	0.206188302	0.19437432	0.39239968
ShSirt1_vs_ShScrambled	Q9D168	Q9D168	Ints12	71793	0.240604606	0.1946682	0.39267655
ShSirt1_vs_ShScrambled	Q7TMV3	Q7TMV3	Fastkd5	380601	-0.548244447	0.19468545	0.39267655
ShSirt1_vs_ShScrambled	P03888	P03888	Mtnd1	17716	-1.235699149	0.19482238	0.39269364
ShSirt1_vs_ShScrambled	O35566	O35566	Cd151	12476	-1.068753688	0.19492412	0.39269364
ShSirt1_vs_ShScrambled	Q9Z2W0	Q9Z2W0	Dnpep	13437	0.167674603	0.19501926	0.39269364
ShSirt1_vs_ShScrambled	Q9CY64	Q9CY64	Blvra	109778	0.195158545	0.1950419	0.39269364
ShSirt1_vs_ShScrambled	Q9QUI0	Q9QUI0	Rhoa	11848	0.268207766	0.19535904	0.3931568
ShSirt1_vs_ShScrambled	Q9DC16	Q9DC16	Ergic1	67458	0.188636063	0.19568706	0.39364143
ShSirt1_vs_ShScrambled	O55201	O55201	Supt5h	20924	0.419349202	0.19593377	0.39379294
ShSirt1_vs_ShScrambled	Q6PFD9	Q6PFD9	Nup98	269966	0.312083962	0.19593685	0.39379294
ShSirt1_vs_ShScrambled	Q61205	Q61205	Pafah1b3	18476	0.350679962	0.19608966	0.39392467
ShSirt1_vs_ShScrambled	Q9CZR8	Q9CZR8	Tsfm	66399	-0.379058355	0.19623731	0.39394835
ShSirt1_vs_ShScrambled	O35245	O35245	Pkd2	18764	-0.472502736	0.19627599	0.39394835
ShSirt1_vs_ShScrambled	Q9R0Q4	Q9R0Q4	Morf4l2	56397	0.633442675	0.19657449	0.39414684
ShSirt1_vs_ShScrambled	Q3UDR8	Q3UDR8	Yipf3	28064	0.5342837	0.19660813	0.39414684
ShSirt1_vs_ShScrambled	P05622	P05622	Pdgfrb	18596	-0.280109907	0.19663684	0.39414684
ShSirt1_vs_ShScrambled	P97372	P97372	Psme2	19188; 62	0.44196259	0.19686733	0.39443371
ShSirt1_vs_ShScrambled	Q8BUH8	Q8BUH8	Senp7	66315	0.736680372	0.19713263	0.39456053
ShSirt1_vs_ShScrambled	Q6PHZ2	Q6PHZ2	Camk2d	108058	-0.19211269	0.19715348	0.39456053
ShSirt1_vs_ShScrambled	Q3UJD6	Q3UJD6	Usp19	71472	-0.264854642	0.19719286	0.39456053
ShSirt1_vs_ShScrambled	O55234	O55234	Psmb5	19173	-0.208595907	0.19748781	0.39491899
ShSirt1_vs_ShScrambled	O70551	O70551	Srpk1	20815	-1.030463349	0.19757894	0.39491899
ShSirt1_vs_ShScrambled	Q9D902	Q9D902	Gtf2e2	68153	0.372468422	0.19763447	0.39491899
ShSirt1_vs_ShScrambled	Q6PNC0	Q6PNC0	Dmxl1	240283	-0.397273031	0.19784396	0.39516267
ShSirt1_vs_ShScrambled	Q9Z210	Q9Z210	Pex11b	18632	0.570659394	0.1980373	0.3953472

ShSirt1_vs_ShScrambled	Q9CR21	Q9CR21	Ndufab1	70316	-0.237624021	0.1982963	0.3953472
ShSirt1_vs_ShScrambled	P59024	P59024	Fkbp14	231997	-0.783308873	0.1983114	0.3953472
ShSirt1_vs_ShScrambled	Q8R0A0	Q8R0A0	Gtf2f2	68705	0.372790693	0.19840758	0.3953472
ShSirt1_vs_ShScrambled	O55028	O55028	Bckdk	12041	0.220389283	0.19845998	0.3953472
ShSirt1_vs_ShScrambled	P63158	P63158	Hmgb1	15289	0.26272911	0.19846184	0.3953472
ShSirt1_vs_ShScrambled	Q3U821	Q3U821	Wdr75	73674	0.614023914	0.19864972	0.39554691
ShSirt1_vs_ShScrambled	Q91YU8	Q91YU8	Ppan	235036	1.024829949	0.19874974	0.39557157
ShSirt1_vs_ShScrambled	Q9Z2V5	Q9Z2V5	Hdac6	15185	0.355478176	0.19892094	0.39573782
ShSirt1_vs_ShScrambled	Q3UGC7	Q3UGC7;Q6	Eif3j1		-0.294185962	0.19912601	0.39593381
ShSirt1_vs_ShScrambled	Q6PDM2	Q6PDM2	Srsf1	110809	0.286991871	0.19919488	0.39593381
ShSirt1_vs_ShScrambled	Q91XF0	Q91XF0	Pnpo	103711	0.254413571	0.19942327	0.3959799
ShSirt1_vs_ShScrambled	Q8VCH8	Q8VCH8	Ubxn4	67812	-0.590513346	0.19942641	0.3959799
ShSirt1_vs_ShScrambled	Q9Z2Z6	Q9Z2Z6	Slc25a20	57279	-0.510659599	0.19960112	0.3959799
ShSirt1_vs_ShScrambled	Q99LC2	Q99LC2	Cstf1	67337	0.490360107	0.19965116	0.3959799
ShSirt1_vs_ShScrambled	Q8R574	Q8R574	Prpsap2	212627	0.403187882	0.19965668	0.3959799
ShSirt1_vs_ShScrambled	P62073	P62073	Timm10	30059	-1.003934843	0.20001258	0.39651155
ShSirt1_vs_ShScrambled	Q8R016	Q8R016	Blmh	104184	0.222020553	0.20027736	0.39676975
ShSirt1_vs_ShScrambled	Q9D172	Q9D172	D10Jhu81e	28295	0.201404271	0.20031862	0.39676975
ShSirt1_vs_ShScrambled	Q60932	Q60932	Vdac1	22333	0.197788481	0.20121533	0.39837105
ShSirt1_vs_ShScrambled	Q9JI19	Q9JI19	Fibp	58249	0.570470726	0.20161983	0.39899689
ShSirt1_vs_ShScrambled	Q8K1J6	Q8K1J6	Trnt1	70047	0.297250367	0.20230039	0.40016826
ShSirt1_vs_ShScrambled	Q91ZN5	Q91ZN5	Slc35b2		0.398282295	0.20239501	0.40018006
ShSirt1_vs_ShScrambled	O35640	O35640	Anxa8	11752	-0.273075207	0.20250667	0.40022552
ShSirt1_vs_ShScrambled	Q7TMA2	Q7TMA2	Znf503	218820	-0.734201378	0.20263719	0.40030823
ShSirt1_vs_ShScrambled	Q3TIR3	Q3TIR3	Ric8a	101489	-0.481439192	0.20290928	0.40067038
ShSirt1_vs_ShScrambled	B9EJR8	B9EJR8	Dnaaf5	433956	0.521806683	0.20308562	0.40068476
ShSirt1_vs_ShScrambled	Q6DID7	Q6DID7	Wls	68151	0.460996651	0.20309409	0.40068476
ShSirt1_vs_ShScrambled	Q99MR6	Q99MR6	Srrt	83701	0.19604556	0.20330813	0.4009318
ShSirt1_vs_ShScrambled	O70310	O70310	Nmt1	18107	0.240231642	0.20378476	0.40169625
ShSirt1_vs_ShScrambled	O08796	O08796	Eef2k	13631	-0.458976134	0.20392329	0.40179386
ShSirt1_vs_ShScrambled	Q8BG51	Q8BG51	Rhot1	59040	-0.177327112	0.20435691	0.40247256
ShSirt1_vs_ShScrambled	Q8K296	Q8K296	Mtmr3	74302	-0.630174772	0.20495011	0.4034648
ShSirt1_vs_ShScrambled	Q91XY4	Q91XY4	Pcdhga4	93712	-0.644784841	0.20513213	0.40355974
ShSirt1_vs_ShScrambled	Q9CQN6	Q9CQN6	Tmem14c	66154	-0.50475805	0.20517714	0.40355974
ShSirt1_vs_ShScrambled	Q9JKV5	Q9JKV5	Scamp4	56214	-0.614107733	0.20562014	0.40425492
ShSirt1_vs_ShScrambled	Q9CR89	Q9CR89	Ergic2	67456	0.240301023	0.20601218	0.40484936
ShSirt1_vs_ShScrambled	Q5BLK4	Q5BLK4	Zcchc6	214290	0.846564946	0.20688084	0.40637951
ShSirt1_vs_ShScrambled	Q3UQ28	Q3UQ28	Pxdn	69675	0.391348143	0.20725386	0.40674213
ShSirt1_vs_ShScrambled	Q91W34	Q91W34	Q91W34	233913	-0.213517531	0.20729748	0.40674213
ShSirt1_vs_ShScrambled	Q8BRN9	Q8BRN9	Cc2d1b	319965	-1.093514125	0.20733576	0.40674213
ShSirt1_vs_ShScrambled	Q6A026	Q6A026	Pds5a		0.35614926	0.20753232	0.40695087
ShSirt1_vs_ShScrambled	Q91ZW2	Q91ZW2	Pofut1	140484	0.237052403	0.2076292	0.40696405
ShSirt1_vs_ShScrambled	Q791T5	Q791T5	Mtch1	56462	-0.309739329	0.20814507	0.40762522
ShSirt1_vs_ShScrambled	Q8R0S1	Q8R0S1	Atf7		1.605110569	0.20822559	0.40762522
ShSirt1_vs_ShScrambled	Q9CQ85	Q9CQ85	Timm22	56322	0.239666903	0.20824916	0.40762522
ShSirt1_vs_ShScrambled	Q8VCM5	Q8VCM5	Mul1	68350	0.304268522	0.20832773	0.40762522
ShSirt1_vs_ShScrambled	P61027	P61027	Rab10	19325	0.198200314	0.20850109	0.40778765
ShSirt1_vs_ShScrambled	O55091	O55091	Impact	16210	0.885258784	0.20874451	0.40806926
ShSirt1_vs_ShScrambled	P61358	P61358	Rpl27	19942; 10	0.208548269	0.20890116	0.40806926
ShSirt1_vs_ShScrambled	Q9CQV1	Q9CQV1	Pam16	66449	0.200489359	0.20891627	0.40806926
ShSirt1_vs_ShScrambled	P62717	P62717	Rpl18a	76808	-0.227414893	0.20930611	0.40835732
ShSirt1_vs_ShScrambled	P84089	P84089	Erh	13877	-0.228867396	0.20932004	0.40835732
ShSirt1_vs_ShScrambled	Q8BGV0	Q8BGV0	Nars2	244141	-1.559217904	0.20933514	0.40835732
ShSirt1_vs_ShScrambled	Q62432	Q62432	Smad2	17126	-0.287724339	0.20942662	0.4083593
ShSirt1_vs_ShScrambled	Q91W61	Q91W61	Fbxl15	68431	-0.935823339	0.20977605	0.40886403
ShSirt1_vs_ShScrambled	Q9D154	Q9D154	Serpinb1a	66222	-0.392038773	0.21007358	0.40919676
ShSirt1_vs_ShScrambled	Q6VNB8	Q6VNB8	Wdfy3	72145	-0.487412967	0.21012806	0.40919676
ShSirt1_vs_ShScrambled	Q8CFH6	Q8CFH6	Sik2	235344	-0.448468798	0.21052982	0.40964486
ShSirt1_vs_ShScrambled	Q99LE6	Q99LE6	Abcf2	27407	-0.189650404	0.21055794	0.40964486
ShSirt1_vs_ShScrambled	Q99JF5	Q99JF5	Mvd	192156	-0.373265744	0.21063042	0.40964486
ShSirt1_vs_ShScrambled	Q9D0F6	Q9D0F6	Rfc5	72151	0.505229989	0.21136017	0.41078187
ShSirt1_vs_ShScrambled	Q61239	Q61239	Fnta	14272	-0.183491176	0.21139705	0.41078187
ShSirt1_vs_ShScrambled	Q3TCH7	Q3TCH7	Cul4a	99375	-0.322435201	0.21160157	0.41088235

ShSirt1_vs_ShScrambled	Q62318	Q62318	Trim28	21849	0.184631368	0.21166779	0.41088235
ShSirt1_vs_ShScrambled	P22907	P22907	Hmbs	15288	0.175275532	0.21180209	0.41088235
ShSirt1_vs_ShScrambled	Q8C0T5	Q8C0T5	Sipa1l1	217692	-0.501389175	0.21181286	0.41088235
ShSirt1_vs_ShScrambled	Q8BU03	Q8BU03	Pwp2	110816	0.434034312	0.21197542	0.41102106
ShSirt1_vs_ShScrambled	Q8BGU5	Q8BGU5	Ccny	67974	0.531073232	0.21240013	0.41166774
ShSirt1_vs_ShScrambled	D3YZP9	D3YZP9	Ccdc6	76551	0.476672295	0.21284666	0.41230372
ShSirt1_vs_ShScrambled	Q8VHN8	Q8VHN8	Nudt16l1	66911	0.205292922	0.21291094	0.41230372
ShSirt1_vs_ShScrambled	Q8C1B7	Q8C1B7	Sept11	52398	-0.230836494	0.21342429	0.41312061
ShSirt1_vs_ShScrambled	Q8BYR1	Q8BYR1	Lcmt2	329504	-0.597825468	0.21366675	0.41341266
ShSirt1_vs_ShScrambled	Q60817	Q60817;P70(	Naca		1.406040864	0.2139146	0.41349972
ShSirt1_vs_ShScrambled	Q9WUM3	Q9WUM3	Coro1b	23789	-0.190292538	0.21401262	0.41349972
ShSirt1_vs_ShScrambled	Q6SKR2	Q6SKR2	N6amt1	67768	0.413015452	0.214013	0.41349972
ShSirt1_vs_ShScrambled	Q99KR7	Q99KR7	Ppif	105675	0.458993436	0.21407817	0.41349972
ShSirt1_vs_ShScrambled	O08585	O08585	Clta		-0.242390793	0.21421418	0.41358546
ShSirt1_vs_ShScrambled	Q02013	Q02013	Aqp1	11826	-0.596520953	0.21432915	0.41363053
ShSirt1_vs_ShScrambled	Q9CXT7	Q9CXT7	Tmem192	73067	-0.448933404	0.21472118	0.41412327
ShSirt1_vs_ShScrambled	Q8K1N4	Q8K1N4	Spats2	72572	0.59565967	0.21476796	0.41412327
ShSirt1_vs_ShScrambled	P37889	P37889	Fbln2	14115	0.399066665	0.21492985	0.4142069
ShSirt1_vs_ShScrambled	Q9CPT5	Q9CPT5	Nop16	28126	0.370176302	0.21515664	0.4142069
ShSirt1_vs_ShScrambled	Q99M01	Q99M01	Fars2	69955	-0.232967684	0.2151864	0.4142069
ShSirt1_vs_ShScrambled	Q9D0R4	Q9D0R4	Ddx56	52513	0.313916593	0.21525911	0.4142069
ShSirt1_vs_ShScrambled	Q6P1F6	Q6P1F6	Ppp2r2a	71978	0.184278578	0.21527014	0.4142069
ShSirt1_vs_ShScrambled	Q9CQF4	Q9CQF4	Q9CQF4	67851	-0.400208434	0.21546525	0.41440569
ShSirt1_vs_ShScrambled	Q9D4H4	Q9D4H4	Amotl1	75723	1.478798041	0.2156154	0.41451785
ShSirt1_vs_ShScrambled	Q8BSF4	Q8BSF4	Pisd	320951	-0.242691356	0.21617816	0.41529509
ShSirt1_vs_ShScrambled	P43277	P43277	Hist1h1d	14957	0.400934004	0.21620369	0.41529509
ShSirt1_vs_ShScrambled	Q8BK64	Q8BK64	Ahsa1	217737	-0.236738433	0.21648704	0.41566248
ShSirt1_vs_ShScrambled	Q9ERB0	Q9ERB0	Snap29	67474	-0.4428992	0.21688025	0.41624041
ShSirt1_vs_ShScrambled	P36916	P36916	Gnl1	14670	0.250507392	0.21698939	0.41627288
ShSirt1_vs_ShScrambled	Q8BYA0	Q8BYA0	Tbcd	108903	-0.18250499	0.21732911	0.41674749
ShSirt1_vs_ShScrambled	Q9JJH7	Q9JJH7	Trpm5	56843	0.249698405	0.21778334	0.41744118
ShSirt1_vs_ShScrambled	P60521	P60521	Gabarapl2	93739	-0.386579691	0.21795557	0.41759399
ShSirt1_vs_ShScrambled	Q9CQB5	Q9CQB5	Cisd2	67006	0.751046173	0.21818541	0.41775485
ShSirt1_vs_ShScrambled	Q9CXE7	Q9CXE7	Tmed5	73130	-0.658707957	0.21822462	0.41775485
ShSirt1_vs_ShScrambled	Q8BMP6	Q8BMP6	Acbd3	170760	0.277513425	0.21842006	0.41783992
ShSirt1_vs_ShScrambled	Q5ND52	Q5ND52	Rnmtl1	67390	0.588513968	0.21845419	0.41783992
ShSirt1_vs_ShScrambled	Q9QZE7	Q9QZE7	Tsnax	53424	0.334916496	0.21870952	0.41794583
ShSirt1_vs_ShScrambled	P56959	P56959	Fus	233908	0.264550631	0.21872795	0.41794583
ShSirt1_vs_ShScrambled	Q6KCD5	Q6KCD5	Nipbl	71175	0.334251455	0.21884108	0.41794583
ShSirt1_vs_ShScrambled	Q9D0A3	Q9D0A3	Arpin	70420	0.437500089	0.21895036	0.41794583
ShSirt1_vs_ShScrambled	P70700	P70700	Polr1b	20017	0.587521826	0.21897251	0.41794583
ShSirt1_vs_ShScrambled	Q9CQY5	Q9CQY5	Magt1		-0.372586694	0.21967145	0.41910268
ShSirt1_vs_ShScrambled	Q8R0S2	Q8R0S2	Iqsec1	232227	-0.272201309	0.22018196	0.41968433
ShSirt1_vs_ShScrambled	P47962	P47962	Rpl5	1.01E+08	0.162176852	0.22021438	0.41968433
ShSirt1_vs_ShScrambled	P53702	P53702	Hccs	15159	-0.401570002	0.22025525	0.41968433
ShSirt1_vs_ShScrambled	Q99KK7	Q99KK7	Dpp3	75221	-0.160539972	0.22078674	0.42051956
ShSirt1_vs_ShScrambled	Q99LF4	Q99LF4	Rtcb	28088	-0.260763937	0.22097237	0.4206956
ShSirt1_vs_ShScrambled	Q9ESX5	Q9ESX5	Dkc1	245474	0.174491219	0.22128014	0.4208098
ShSirt1_vs_ShScrambled	Q8BR70	Q8BR70	Yipf6	77929	0.468816637	0.22128571	0.4208098
ShSirt1_vs_ShScrambled	P63321	P63321	Rala	56044	0.376365	0.22138702	0.4208098
ShSirt1_vs_ShScrambled	Q9R0G7	Q9R0G7	Zeb2	24136	0.370427927	0.22140524	0.4208098
ShSirt1_vs_ShScrambled	Q9D404	Q9D404	Oxsm	71147	-0.258661398	0.22160972	0.42102116
ShSirt1_vs_ShScrambled	Q3UIA2	Q3UIA2	Arhgap17	70497	0.639825556	0.22215603	0.4218815
ShSirt1_vs_ShScrambled	Q8R307	Q8R307	Vps18	228545	0.273091929	0.22234423	0.42206133
ShSirt1_vs_ShScrambled	Q8C854	Q8C854	Myef2	17876	0.189616885	0.22288758	0.4229149
ShSirt1_vs_ShScrambled	Q80WT5	Q80WT5	Aftph	216549	0.224252007	0.22302618	0.42300007
ShSirt1_vs_ShScrambled	Q791V5	Q791V5	Mtch2	56428	-0.194668548	0.22323332	0.42305204
ShSirt1_vs_ShScrambled	Q61687	Q61687	Atrx	22589	-0.456747652	0.22325661	0.42305204
ShSirt1_vs_ShScrambled	Q9Z2U0	Q9Z2U0	Psma7	26444	-0.257870665	0.22333474	0.42305204
ShSirt1_vs_ShScrambled	Q8VD12	Q8VD12	Znf385a	29813	-0.426376043	0.22391381	0.42397103
ShSirt1_vs_ShScrambled	Q9CQG2	Q9CQG2	Mettl16	67493	0.284697058	0.22419723	0.42432969
ShSirt1_vs_ShScrambled	P58044	P58044	Idi1	319554; 1(	-0.272064495	0.22495794	0.42555903
ShSirt1_vs_ShScrambled	P17095	P17095	Hmga1	15361; 11	0.762520177	0.22503531	0.42555903

ShSirt1_vs_ShScrambled	Q9R0P6	Q9R0P6	Sec11a	56529	-0.426057284	0.22521914	0.42572831
ShSirt1_vs_ShScrambled	P51863	P51863	Atp6v0d1	11972	-0.333311348	0.22535535	0.42580746
ShSirt1_vs_ShScrambled	Q8CAY6	Q8CAY6	Acat2	110460	-0.170777371	0.22567054	0.42622462
ShSirt1_vs_ShScrambled	Q9D8P4	Q9D8P4	Mrpl17	27397	-0.181973196	0.22588944	0.42645961
ShSirt1_vs_ShScrambled	O55106	O55106	Strn	268980	0.195200051	0.22607226	0.42662632
ShSirt1_vs_ShScrambled	Q4VAE3	Q4VAE3	Tmem65	74868	0.293003868	0.22682368	0.42786548
ShSirt1_vs_ShScrambled	P61620	P61620	Sec61a1	53421	-0.24415791	0.22842085	0.43069829
ShSirt1_vs_ShScrambled	O88895	O88895	Hdac3		-0.477899722	0.22859415	0.4307104
ShSirt1_vs_ShScrambled	Q91VZ6	Q91VZ6	Smap1	98366	-0.364698671	0.2286181	0.4307104
ShSirt1_vs_ShScrambled	Q3UX10	Q3UX10	Tubal3	238463	-0.209078288	0.22877178	0.43082012
ShSirt1_vs_ShScrambled	Q5XKN4	Q5XKN4	Jagn1	67767	-0.264647122	0.22940892	0.43142619
ShSirt1_vs_ShScrambled	Q5I2A0	Q5I2A0;Q80X	Serpina3g		-1.031250113	0.22950075	0.43142619
ShSirt1_vs_ShScrambled	Q6PDH0	Q6PDH0	Phldb1	102693	-0.180627951	0.22958595	0.43142619
ShSirt1_vs_ShScrambled	Q9JJA2	Q9JJA2	Cog8	97484	0.557926213	0.22959829	0.43142619
ShSirt1_vs_ShScrambled	Q8BIJ6	Q8BIJ6	Iars2	381314	0.183015376	0.22964228	0.43142619
ShSirt1_vs_ShScrambled	Q9R078	Q9R078	Prkab1	19079	-0.281020557	0.22966707	0.43142619
ShSirt1_vs_ShScrambled	Q9CZP5	Q9CZP5	Bcs1l	66821	0.350789517	0.22991703	0.43161618
ShSirt1_vs_ShScrambled	Q60780	Q60780	Gas7		-0.533567114	0.23001996	0.43161618
ShSirt1_vs_ShScrambled	P97367	P97367;Q60	Meis2		-0.627880934	0.23005506	0.43161618
ShSirt1_vs_ShScrambled	P55264	P55264	Adk	11534	0.165044237	0.23030802	0.43175403
ShSirt1_vs_ShScrambled	Q9R1Z8	Q9R1Z8	Sorbs3	20410	0.236695549	0.230402	0.43175403
ShSirt1_vs_ShScrambled	Q8VD75	Q8VD75	Hip1	215114	0.228173536	0.23049057	0.43175403
ShSirt1_vs_ShScrambled	P59808	P59808	Sash1	70097	0.617800957	0.23054123	0.43175403
ShSirt1_vs_ShScrambled	P35831	P35831	Ptpn12	19248	0.282700812	0.23067066	0.43175403
ShSirt1_vs_ShScrambled	Q6NZM9	Q6NZM9	Hdac4	208727	-0.413130112	0.23078445	0.43175403
ShSirt1_vs_ShScrambled	Q8K2Z2	Q8K2Z2	Prpf39		0.565681775	0.23079807	0.43175403
ShSirt1_vs_ShScrambled	O08749	O08749	Dld	13382	-0.222417152	0.23109712	0.43213439
ShSirt1_vs_ShScrambled	P24549	P24549	Aldh1a1	11668	0.203063552	0.23164913	0.43275177
ShSirt1_vs_ShScrambled	P63101	P63101	Ywhaz	22631	0.281046456	0.23175334	0.43275177
ShSirt1_vs_ShScrambled	P26618	P26618	Pdgfra	18595	-0.48366736	0.23179069	0.43275177
ShSirt1_vs_ShScrambled	P55937	P55937	Golga3	269682	0.173900923	0.23181076	0.43275177
ShSirt1_vs_ShScrambled	Q64213	Q64213	Sf1		0.227380989	0.23204106	0.43300262
ShSirt1_vs_ShScrambled	Q9Z2Q6	Q9Z2Q6	Sept5	18951	0.314883644	0.23246216	0.43360917
ShSirt1_vs_ShScrambled	Q80UY1	Q80UY1	Q80UY1	67383	-0.342705009	0.23279727	0.43405489
ShSirt1_vs_ShScrambled	Q9Z2A0	Q9Z2A0	Pdpk1	18607	0.453598669	0.23296976	0.4341129
ShSirt1_vs_ShScrambled	Q8CG70	Q8CG70	Leprel2	14789	-0.475417007	0.233043	0.4341129
ShSirt1_vs_ShScrambled	P29341	P29341	Pabpc1	18458	0.220274144	0.2331169	0.4341129
ShSirt1_vs_ShScrambled	Q9CQD1	Q9CQD1	Rab5a	271457	0.259588891	0.23355066	0.43474131
ShSirt1_vs_ShScrambled	Q923D2	Q923D2	Blvrb	233016	0.202337781	0.2339978	0.43539409
ShSirt1_vs_ShScrambled	P35293	P35293	Rab18	19330	-0.235319557	0.23416344	0.43545534
ShSirt1_vs_ShScrambled	Q9CWU6	Q9CWU6	Uqcc1	56046	0.196420421	0.23427039	0.43545534
ShSirt1_vs_ShScrambled	Q80X90	Q80X90	Flnb	286940	0.157911924	0.23432012	0.43545534
ShSirt1_vs_ShScrambled	Q9WUR2	Q9WUR2	Eci2	23986	-0.232803841	0.23484707	0.436255
ShSirt1_vs_ShScrambled	Q9ESZ8	Q9ESZ8	Gtf2i	14886	0.232106613	0.23524234	0.43680951
ShSirt1_vs_ShScrambled	P43247	P43247	Msh2	17685	-0.757711244	0.23584243	0.43757768
ShSirt1_vs_ShScrambled	Q61009	Q61009	Scarb1	20778	0.583834179	0.23599373	0.43757768
ShSirt1_vs_ShScrambled	Q7TMI3	Q7TMI3	Uhrf2	109113	-0.350883545	0.23600787	0.43757768
ShSirt1_vs_ShScrambled	Q9CQL5	Q9CQL5	Mrpl18	67681	0.422357609	0.23604379	0.43757768
ShSirt1_vs_ShScrambled	Q5FWK3	Q5FWK3	Arhgap1	228359	0.314029785	0.23623861	0.43775907
ShSirt1_vs_ShScrambled	Q99M31	Q99M31	Hspa14	50497	0.167783799	0.23640339	0.43788466
ShSirt1_vs_ShScrambled	Q05186	Q05186	Rcn1	19672	0.827047953	0.23668445	0.43802607
ShSirt1_vs_ShScrambled	Q99LG2	Q99LG2	Tnpo2	212999	-0.307795433	0.23670255	0.43802607
ShSirt1_vs_ShScrambled	Q5SWD9	Q5SWD9	Tsr1	104662	-0.189541263	0.23682693	0.43802607
ShSirt1_vs_ShScrambled	Q91X58	Q91X58	Zfand2b	68818	-0.311245156	0.23686789	0.43802607
ShSirt1_vs_ShScrambled	P63087	P63087	Ppp1cc	19047	0.439613476	0.23702747	0.43814169
ShSirt1_vs_ShScrambled	Q9CQX8	Q9CQX8	Mrps36	66128	0.333302117	0.23733477	0.43853015
ShSirt1_vs_ShScrambled	Q8VE22	Q8VE22	Mrps23	64656	-0.169473246	0.23769033	0.43882031
ShSirt1_vs_ShScrambled	Q9ERK4	Q9ERK4	Cse1l	110750	0.199172416	0.2377728	0.43882031
ShSirt1_vs_ShScrambled	Q6NWW9	Q6NWW9	Fndc3b	72007	-0.471544777	0.2378721	0.43882031
ShSirt1_vs_ShScrambled	Q9QZB9	Q9QZB9	Dctn5	59288	0.568468937	0.23789767	0.43882031
ShSirt1_vs_ShScrambled	Q9WUD1	Q9WUD1	Stub1	56424	0.313803066	0.23797787	0.43882031
ShSirt1_vs_ShScrambled	Q9R0Q3	Q9R0Q3	Tmed2	56334	0.272346844	0.2381953	0.43899871
ShSirt1_vs_ShScrambled	Q8R366	Q8R366	Igsf8	140559	0.251032686	0.23829001	0.43899871

ShSirt1_vs_ShScrambled	Q80WC7	Q80WC7	Agfg2	231801	0.952781471	0.23836638	0.43899871
ShSirt1_vs_ShScrambled	Q9CXI5	Q9CXI5	Manf		-0.477211831	0.23870324	0.4392499
ShSirt1_vs_ShScrambled	Q8BGC0	Q8BGC0	Htatsf1	72459	0.499461701	0.23872516	0.4392499
ShSirt1_vs_ShScrambled	Q99KQ4	Q99KQ4	Nampt	59027	0.150201806	0.23879489	0.4392499
ShSirt1_vs_ShScrambled	Q9CYZ6	Q9CYZ6	Q9CYZ6	66462	-0.378479522	0.238892	0.4392499
ShSirt1_vs_ShScrambled	Q60790	Q60790	Rasa3	19414	-0.282182366	0.23919976	0.43963668
ShSirt1_vs_ShScrambled	Q8VDR9	Q8VDR9	Dock6	319899	1.23707868	0.23931372	0.43966713
ShSirt1_vs_ShScrambled	P50431	P50431	Shmt1	20425	-0.180971673	0.23947866	0.43979116
ShSirt1_vs_ShScrambled	Q9CQF3	Q9CQF3	Nudt21	68219	0.19054851	0.23985504	0.44012842
ShSirt1_vs_ShScrambled	Q9QXK3	Q9QXK3	Copg2	54160	0.181786942	0.23985731	0.44012842
ShSirt1_vs_ShScrambled	Q9R0B7	Q9R0B7	Znf346	26919	-0.549594999	0.24004053	0.4401496
ShSirt1_vs_ShScrambled	Q9WTR2	Q9WTR2	Map3k6		-0.390761565	0.24013766	0.4401496
ShSirt1_vs_ShScrambled	O88874	O88874	Ccnk		0.420184313	0.24016138	0.4401496
ShSirt1_vs_ShScrambled	Q8CIG8	Q8CIG8	Prmt5	27374	0.245815499	0.2404464	0.44035446
ShSirt1_vs_ShScrambled	B2RXS4	B2RXS4	Plxnb2	140570	-0.237248784	0.24051148	0.44035446
ShSirt1_vs_ShScrambled	Q9DCL8	Q9DCL8	Ppp1r2	66849	0.606736101	0.24070712	0.44035446
ShSirt1_vs_ShScrambled	Q91YD3	Q91YD3	Dcp1a	75901	0.234831617	0.24106715	0.44035446
ShSirt1_vs_ShScrambled	Q9D0W5	Q9D0W5	Ppil1	68816	0.203653591	0.2411105	0.44035446
ShSirt1_vs_ShScrambled	O08539	O08539	Bin1	30948	0.260789849	0.24114462	0.44035446
ShSirt1_vs_ShScrambled	Q8VCW8	Q8VCW8	Acsf2	264895	-0.214688395	0.24118706	0.44035446
ShSirt1_vs_ShScrambled	Q8VEB4	Q8VEB4	Pla2g15	192654	-0.403872387	0.24122132	0.44035446
ShSirt1_vs_ShScrambled	Q9CQI7	Q9CQI7	Snrpb2	20639	0.705012402	0.24144889	0.44035446
ShSirt1_vs_ShScrambled	Q8BPM2	Q8BPM2	Map4k5	399510	0.457473634	0.24145232	0.44035446
ShSirt1_vs_ShScrambled	Q9D3U0	Q9D3U0	Pus10	74467	0.667903204	0.24147069	0.44035446
ShSirt1_vs_ShScrambled	Q9D1G1	Q9D1G1	Rab1b	76308	-0.160637262	0.24153635	0.44035446
ShSirt1_vs_ShScrambled	Q61072	Q61072	Adam9	11502	-0.560981572	0.24157651	0.44035446
ShSirt1_vs_ShScrambled	Q91ZR1	Q91ZR1	Rab4b	19342	0.619445544	0.2416389	0.44035446
ShSirt1_vs_ShScrambled	Q8BYL4	Q8BYL4	Yars2	70120	0.357135525	0.2424432	0.44143909
ShSirt1_vs_ShScrambled	P97386	P97386	Lig3		0.309091287	0.24257644	0.44143909
ShSirt1_vs_ShScrambled	Q99J09	Q99J09	Wdr77	70465	0.266632404	0.24260907	0.44143909
ShSirt1_vs_ShScrambled	Q6ZWN5	Q6ZWN5	Rps9	76846	-0.205609893	0.24262525	0.44143909
ShSirt1_vs_ShScrambled	Q66T02	Q66T02	Plekhg5	269608	-0.595877061	0.24281986	0.44161516
ShSirt1_vs_ShScrambled	P35585	P35585	Ap1m1	11767	0.219944988	0.24316671	0.44187259
ShSirt1_vs_ShScrambled	P29595	P29595	Nedd8	18002	-0.344026816	0.24336071	0.44187259
ShSirt1_vs_ShScrambled	Q60575	Q60575	Kif1b	16561	-0.224361909	0.24339202	0.44187259
ShSirt1_vs_ShScrambled	Q9EQ80	Q9EQ80	Nif3l1	65102	-0.177693233	0.24343728	0.44187259
ShSirt1_vs_ShScrambled	P52019	P52019	Sqle	20775	-0.250711495	0.24345085	0.44187259
ShSirt1_vs_ShScrambled	Q62417	Q62417	Sorbs1	20411	-0.22568752	0.24357427	0.44191891
ShSirt1_vs_ShScrambled	Q7TMW6	Q7TMW6	Narfl	67563	0.385767111	0.24396849	0.44238673
ShSirt1_vs_ShScrambled	Q8C3W1	Q8C3W1	Q8C3W1	69551	-0.507252866	0.24407222	0.44238673
ShSirt1_vs_ShScrambled	Q91ZF0	Q91ZF0	Dnajc24	99349	-0.468955845	0.24412613	0.44238673
ShSirt1_vs_ShScrambled	P58064	P58064	Mrps6	121022	-0.866166066	0.24450218	0.4427229
ShSirt1_vs_ShScrambled	Q5EBG8	Q5EBG8	Q5EBG8	230696	-0.856746514	0.2445325	0.4427229
ShSirt1_vs_ShScrambled	Q9D7N3	Q9D7N3	Mrps9	69527	0.249067525	0.24461107	0.4427229
ShSirt1_vs_ShScrambled	P55258	P55258	Rab8a	17274	-0.181319828	0.24480928	0.4427229
ShSirt1_vs_ShScrambled	P49722	P49722	Psma2	19166	0.212372225	0.24488997	0.4427229
ShSirt1_vs_ShScrambled	P21279	P21279	Gnaq	14682	0.232528046	0.24490011	0.4427229
ShSirt1_vs_ShScrambled	O55222	O55222	Ilk	16202	0.153472989	0.24542471	0.44349366
ShSirt1_vs_ShScrambled	Q9CQM5	Q9CQM5	Txndc17	52700	0.528593089	0.24556402	0.44356783
ShSirt1_vs_ShScrambled	Q3UDW8	Q3UDW8	Hgsnat	52120	0.432471062	0.24572564	0.44368222
ShSirt1_vs_ShScrambled	P84084	P84084	Arf5	11844	0.23322536	0.24671789	0.4452957
ShSirt1_vs_ShScrambled	Q62189	Q62189	Snrpa	53607	0.19774668	0.24703074	0.44568215
ShSirt1_vs_ShScrambled	Q9EQ20	Q9EQ20	Aldh6a1	104776	0.159399254	0.24726936	0.44593444
ShSirt1_vs_ShScrambled	Q8CI04	Q8CI04	Cog3		0.599362824	0.24778993	0.44669479
ShSirt1_vs_ShScrambled	O88456	O88456	Capns1	12336	-0.221976012	0.2480122	0.44691699
ShSirt1_vs_ShScrambled	P49586	P49586	Pcyt1a	13026	0.172710068	0.24811439	0.44692272
ShSirt1_vs_ShScrambled	P07607	P07607	Tyms	22171	1.523177837	0.24849302	0.44742621
ShSirt1_vs_ShScrambled	Q9CR59	Q9CR59	Gadd45gip	102060	-0.330727883	0.24860478	0.44744895
ShSirt1_vs_ShScrambled	Q91VW5	Q91VW5	Golga4	54214	-0.380731169	0.24919275	0.44832845
ShSirt1_vs_ShScrambled	Q8R310	Q8R310	Tmcc3	319880	-0.578995943	0.24966832	0.44894921
ShSirt1_vs_ShScrambled	Q9WV68	Q9WV68	Decr2	26378	-0.298636755	0.24979388	0.44894921
ShSirt1_vs_ShScrambled	Q8R349	Q8R349	Cdc16	69957	0.270689263	0.24983616	0.44894921
ShSirt1_vs_ShScrambled	Q8R5K4	Q8R5K4	Nol6	230082	0.601190812	0.25000584	0.44907536

ShSirt1_vs_ShScrambled	Q9ER73	Q9ER73	Elp4	77766	0.197611914	0.25030837	0.44943992
ShSirt1_vs_ShScrambled	Q8VEH8	Q8VEH8	Erlec1	66753	0.205535377	0.25056687	0.44972519
ShSirt1_vs_ShScrambled	Q6PDI5	Q6PDI5	Ecm29	230249	-0.221497967	0.25083619	0.45002963
ShSirt1_vs_ShScrambled	Q9DD06	Q9DD06	Rarres2	71660	0.520703945	0.25107104	0.45006317
ShSirt1_vs_ShScrambled	P16858	P16858	Gapdh	14433	0.195757379	0.25113993	0.45006317
ShSirt1_vs_ShScrambled	Q99K48	Q99K48	Nono	53610	0.151354374	0.25115399	0.45006317
ShSirt1_vs_ShScrambled	Q8BGF3	Q8BGF3	Wdr92	103784	0.424559741	0.25138256	0.450294
ShSirt1_vs_ShScrambled	Q91WR3	Q91WR3	Ascc2	75452	-1.108622596	0.2517452	0.45067478
ShSirt1_vs_ShScrambled	Q9JLB0	Q9JLB0	Mpp6	56524	-0.184881997	0.25185533	0.45067478
ShSirt1_vs_ShScrambled	Q9JHJ3	Q9JHJ3	Glmp	56700	0.800575909	0.25189465	0.45067478
ShSirt1_vs_ShScrambled	Q9D883	Q9D883	U2af1	108121	0.3272882	0.25206655	0.45080364
ShSirt1_vs_ShScrambled	Q5DTN8	Q5DTN8	Jakmip3	74004	0.207924718	0.25227486	0.45099751
ShSirt1_vs_ShScrambled	Q64433	Q64433	Hspe1	15528	-0.353347097	0.25241082	0.45106194
ShSirt1_vs_ShScrambled	P97855	P97855	G3bp1	27041	-0.207692393	0.25318377	0.45210405
ShSirt1_vs_ShScrambled	Q9Z1G4	Q9Z1G4	Atp6v0a1	11975	-0.213365785	0.25322345	0.45210405
ShSirt1_vs_ShScrambled	Q3ULF4	Q3ULF4	Spg7	234847	-0.911295522	0.25339493	0.45210405
ShSirt1_vs_ShScrambled	Q8VDG5	Q8VDG5	Ppcs	106564	-0.461710463	0.25340154	0.45210405
ShSirt1_vs_ShScrambled	P53612	P53612	Rabggtb	19352	0.527544066	0.25349476	0.45210405
ShSirt1_vs_ShScrambled	Q91VX2	Q91VX2	Ubap2	68926	-0.486236375	0.25374942	0.45225927
ShSirt1_vs_ShScrambled	Q8BH95	Q8BH95	Echs1	93747	-0.150938193	0.25378217	0.45225927
ShSirt1_vs_ShScrambled	Q9DBL2	Q9DBL2	Gdap2	14547	-0.486405194	0.25497595	0.45420736
ShSirt1_vs_ShScrambled	Q641P0	Q641P0	Actr3b	242894	-1.826162583	0.2551114	0.45426938
ShSirt1_vs_ShScrambled	Q7TMK9	Q7TMK9	Syncrip	56403	-0.142744762	0.25550053	0.45478288
ShSirt1_vs_ShScrambled	Q9DC29	Q9DC29	Abcb6	74104	-0.200856599	0.2565759	0.45627492
ShSirt1_vs_ShScrambled	Q8JZR0	Q8JZR0	Acsl5	433256	-0.351665953	0.25659859	0.45627492
ShSirt1_vs_ShScrambled	P70297	P70297	Stam	20844	0.332981771	0.25664201	0.45627492
ShSirt1_vs_ShScrambled	Q8C3P7	Q8C3P7	Mettl3	56335	0.708237555	0.25676764	0.45631856
ShSirt1_vs_ShScrambled	Q9Z1K5	Q9Z1K5	Arih1	23806	-0.28872146	0.25727731	0.45704439
ShSirt1_vs_ShScrambled	P80316	P80316	Cct5	12465	0.219089483	0.25844668	0.45894112
ShSirt1_vs_ShScrambled	Q9WTR5	Q9WTR5	Cdh13	12554	-0.717383994	0.25886122	0.45933474
ShSirt1_vs_ShScrambled	Q61187	Q61187	Tsg101	22088	0.504572096	0.25887186	0.45933474
ShSirt1_vs_ShScrambled	Q9Z1F9	Q9Z1F9	Uba2	50995	0.158411573	0.25925231	0.45972531
ShSirt1_vs_ShScrambled	Q80U70	Q80U70	Suz12	52615	0.734388197	0.25929567	0.45972531
ShSirt1_vs_ShScrambled	Q68FL4	Q68FL4	Ahcyl2	74340	0.167579963	0.25991296	0.46027198
ShSirt1_vs_ShScrambled	Q99MD6	Q99MD6	Txnrd3	232223	-0.295959956	0.25997296	0.46027198
ShSirt1_vs_ShScrambled	P23198	P23198	Cbx3		0.341836191	0.25999325	0.46027198
ShSirt1_vs_ShScrambled	Q8K2C7	Q8K2C7	Os9	216440	-0.179926981	0.26001186	0.46027198
ShSirt1_vs_ShScrambled	Q61026	Q61026	Ncoa2	17978	-0.714434314	0.26019498	0.46041557
ShSirt1_vs_ShScrambled	Q9CQW9	Q9CQW9	Ifitm3	66141	-0.341222374	0.26051637	0.46080364
ShSirt1_vs_ShScrambled	P62889	P62889	Rpl30	19946	0.168730899	0.26067986	0.46091221
ShSirt1_vs_ShScrambled	P26041	P26041	Msn	17698	-0.195748048	0.2609366	0.46118552
ShSirt1_vs_ShScrambled	Q9D1J3	Q9D1J3	Sarnp	66118	0.301023789	0.26121074	0.46148934
ShSirt1_vs_ShScrambled	P35762	P35762	Cd81	12520	-0.534761223	0.26135783	0.46156856
ShSirt1_vs_ShScrambled	P70206	P70206	Plxna1	18844	1.037441046	0.26207305	0.46254916
ShSirt1_vs_ShScrambled	Q6ZWV7	Q6ZWV7	Rpl35	66489	-0.395412683	0.26211802	0.46254916
ShSirt1_vs_ShScrambled	Q9CRA4	Q9CRA4	Msmo1	66234	-0.428026056	0.26237792	0.46281984
ShSirt1_vs_ShScrambled	Q571E4	Q571E4	Galns	50917	1.187266624	0.26248961	0.46281984
ShSirt1_vs_ShScrambled	Q9D9I4	Q9D9I4	Tbc1d20	67231	-0.176243509	0.26267599	0.46281984
ShSirt1_vs_ShScrambled	Q8VDP4	Q8VDP4	Ccar2	219158	0.237901063	0.26268153	0.46281984
ShSirt1_vs_ShScrambled	P63028	P63028	Tpt1	22070	-0.252870864	0.26285184	0.46293922
ShSirt1_vs_ShScrambled	Q99L45	Q99L45	Eif2s2	67204	0.444418779	0.26299889	0.46301756
ShSirt1_vs_ShScrambled	Q925I1	Q925I1	Atad3	108888	0.213215921	0.26325316	0.46307757
ShSirt1_vs_ShScrambled	Q8CCH2	Q8CCH2	Nhlrc3	212114	0.534030298	0.26329317	0.46307757
ShSirt1_vs_ShScrambled	Q924L1	Q924L1	Letmd1	68614	-0.222847469	0.26334075	0.46307757
ShSirt1_vs_ShScrambled	Q8BWW4	Q8BWW4	Larp4	207214	-0.669080415	0.26365117	0.46335662
ShSirt1_vs_ShScrambled	Q9QZQ1	Q9QZQ1	Mllt4	17356	0.423337246	0.26370473	0.46335662
ShSirt1_vs_ShScrambled	Q9Z160	Q9Z160	Cog1	16834	0.289323571	0.26392281	0.46355937
ShSirt1_vs_ShScrambled	Q8VDN2	Q8VDN2	Atp1a1	11928	0.23369827	0.26403779	0.46358094
ShSirt1_vs_ShScrambled	Q01768	Q01768	Nme2	18103	-0.181970208	0.26472378	0.46460464
ShSirt1_vs_ShScrambled	Q9D3D9	Q9D3D9	Atp5d	66043	-0.365075043	0.26493365	0.46479226
ShSirt1_vs_ShScrambled	O88587	O88587	Comt	12846	0.285944532	0.26505312	0.46482121
ShSirt1_vs_ShScrambled	Q14CH7	Q14CH7	Aars2	224805	0.393459066	0.26526371	0.46488981
ShSirt1_vs_ShScrambled	Q8VE92	Q8VE92;Q8(	Rbm4b		-0.231377195	0.26536629	0.46488981

ShSirt1_vs_ShScrambled	Q91VT4	Q91VT4	Cbr4	234309	0.162099615	0.2654012	0.46488981
ShSirt1_vs_ShScrambled	Q6PAC3	Q6PAC3	Dcaf13	223499	0.508915635	0.26561485	0.46508356
ShSirt1_vs_ShScrambled	Q62446	Q62446	Fkbp3	30795	0.281062622	0.26596032	0.46544996
ShSirt1_vs_ShScrambled	P53569	P53569	Cebpz	12607	0.245925424	0.26603032	0.46544996
ShSirt1_vs_ShScrambled	Q80SY4	Q80SY4	Mib1	225164	-0.405784417	0.26638852	0.46589607
ShSirt1_vs_ShScrambled	P22437	P22437	Ptgs1	19224	0.610778113	0.26669673	0.46625446
ShSirt1_vs_ShScrambled	Q6QI06	Q6QI06	Rictor	78757	0.361376491	0.26680512	0.46626337
ShSirt1_vs_ShScrambled	Q7TQK5	Q7TQK5	Ccdc93	70829	-0.156562604	0.26701238	0.46643601
ShSirt1_vs_ShScrambled	Q99J39	Q99J39	Mlycd	56690	-0.177496242	0.26711057	0.46643601
ShSirt1_vs_ShScrambled	Q9EQC5	Q9EQC5	Scyl1	78891	0.402813915	0.26752158	0.46662539
ShSirt1_vs_ShScrambled	Q920E5	Q920E5	Fdps	110196	-0.164637765	0.26752796	0.46662539
ShSirt1_vs_ShScrambled	Q3TEA8	Q3TEA8	Hp1bp3	15441	-0.24513411	0.26752913	0.46662539
ShSirt1_vs_ShScrambled	P47856	P47856	Gfpt1	14583	-0.172190647	0.26811227	0.46731709
ShSirt1_vs_ShScrambled	Q8BUY5	Q8BUY5	Timmdc1	76916	-0.32268351	0.26813276	0.46731709
ShSirt1_vs_ShScrambled	Q8K2Q0	Q8K2Q0	Commd9	76501	-0.263897177	0.26828545	0.46740276
ShSirt1_vs_ShScrambled	Q01730	Q01730	Rsu1		0.338571819	0.26852284	0.46763583
ShSirt1_vs_ShScrambled	Q99PW4	Q99PW4	Tp53rk		0.469875864	0.26896431	0.46815068
ShSirt1_vs_ShScrambled	Q8BVI5	Q8BVI5	Stx16	228960	-0.480743109	0.26902589	0.46815068
ShSirt1_vs_ShScrambled	Q8R4N0	Q8R4N0	Clybl	69634	0.165181647	0.26920488	0.46828163
ShSirt1_vs_ShScrambled	Q66GT5	Q66GT5	Ptpmt1	66461	0.205334313	0.26932997	0.46831876
ShSirt1_vs_ShScrambled	Q9CXT8	Q9CXT8	Pmpcb	73078	-0.158941182	0.26969396	0.46877109
ShSirt1_vs_ShScrambled	Q3UA06	Q3UA06	Trip13	69716	0.316868986	0.26982862	0.46882462
ShSirt1_vs_ShScrambled	Q9CR57	Q9CR57	Rpl14	67115	0.298897888	0.27023894	0.4693569
ShSirt1_vs_ShScrambled	Q9QXZ0	Q9QXZ0	Macf1		-0.416565414	0.27054451	0.46970689
ShSirt1_vs_ShScrambled	Q8R4R6	Q8R4R6	Nup35	69482	0.247827345	0.27083095	0.47002343
ShSirt1_vs_ShScrambled	Q9CPR4	Q9CPR4	Rpl17		-0.281708951	0.27110926	0.4703256
ShSirt1_vs_ShScrambled	Q3TRM4	Q3TRM4	Pnpla6	50767	-0.512229994	0.27138336	0.47062025
ShSirt1_vs_ShScrambled	Q8BW70	Q8BW70	Usp38	74841	0.499673331	0.27189896	0.47110493
ShSirt1_vs_ShScrambled	P34152	P34152	Ptk2	14083	-0.292498532	0.2719044	0.47110493
ShSirt1_vs_ShScrambled	Q6NZJ6	Q6NZJ6	Eif4g1	208643	-0.172523015	0.27218035	0.47110493
ShSirt1_vs_ShScrambled	Q9DCS3	Q9DCS3	Mecr	26922	-0.244967907	0.27219724	0.47110493
ShSirt1_vs_ShScrambled	O35286	O35286	Dhx15	13204	0.153596133	0.27234675	0.47110493
ShSirt1_vs_ShScrambled	Q91VJ2	Q91VJ2	Prkcdbp	109042	0.19992336	0.27236579	0.47110493
ShSirt1_vs_ShScrambled	Q8VHZ7	Q8VHZ7	Imp4	27993	0.774164166	0.27239341	0.47110493
ShSirt1_vs_ShScrambled	Q5SUF2	Q5SUF2	Luc7l3	67684	0.203770834	0.27386931	0.47342992
ShSirt1_vs_ShScrambled	P14131	P14131	Rps16	20055	-0.210522198	0.27394749	0.47342992
ShSirt1_vs_ShScrambled	Q8BFW7	Q8BFW7	Lpp	210126	0.207562262	0.27457595	0.47433442
ShSirt1_vs_ShScrambled	Q8JZM7	Q8JZM7	Cdc73	214498	-0.169529358	0.27471517	0.47439337
ShSirt1_vs_ShScrambled	Q9D281	Q9D281	Fam114a1	68303	0.195308641	0.27484123	0.47442956
ShSirt1_vs_ShScrambled	Q3UHD6	Q3UHD6	Snx27	76742	-0.215610409	0.27512011	0.47472942
ShSirt1_vs_ShScrambled	O54940	O54940;Q8B	Bnip2		-0.315735661	0.27561278	0.47539782
ShSirt1_vs_ShScrambled	Q8BU88	Q8BU88	Mrpl22	216767	-0.253098113	0.27612784	0.47593802
ShSirt1_vs_ShScrambled	Q8C754	Q8C754	Vps52	224705	0.22900921	0.27613683	0.47593802
ShSirt1_vs_ShScrambled	Q8BWG8	Q8BWG8	Arrb1	109689	0.283584164	0.27635129	0.47612585
ShSirt1_vs_ShScrambled	Q9QYI4	Q9QYI4	Dnajb12	56709	-0.377502938	0.27652912	0.47625046
ShSirt1_vs_ShScrambled	Q9JKP5	Q9JKP5	Mbnl1	56758	0.552567903	0.27668905	0.47626355
ShSirt1_vs_ShScrambled	Q8CHI8	Q8CHI8	Ep400	75560	-0.492722829	0.27674774	0.47626355
ShSirt1_vs_ShScrambled	Q80U87	Q80U87	Usp8	84092	0.229856384	0.27708181	0.47645823
ShSirt1_vs_ShScrambled	Q9CWL8	Q9CWL8	Ctnnbl1	66642	0.268171963	0.27717827	0.47645823
ShSirt1_vs_ShScrambled	Q3THK7	Q3THK7	Gmps	229363	0.14841872	0.27719268	0.47645823
ShSirt1_vs_ShScrambled	Q80VJ2	Q80VJ2	Sra1	24068	-0.221173876	0.27729494	0.47645823
ShSirt1_vs_ShScrambled	Q9CQA6	Q9CQA6	Chchd1	66121	-0.491815528	0.27738862	0.47645823
ShSirt1_vs_ShScrambled	Q62465	Q62465	Vat1	26949	-0.219224271	0.27806379	0.47739873
ShSirt1_vs_ShScrambled	Q91VC9	Q91VC9	Ghitm	66092	-0.211293576	0.27817898	0.47739873
ShSirt1_vs_ShScrambled	Q9Z2X8	Q9Z2X8	Keap1	50868	0.599313732	0.27825345	0.47739873
ShSirt1_vs_ShScrambled	B2RUR8	B2RUR8	Otud7b	229603	0.479553243	0.27892613	0.47837102
ShSirt1_vs_ShScrambled	Q8BMF4	Q8BMF4	Dlat	235339	-0.174749719	0.2793491	0.47891448
ShSirt1_vs_ShScrambled	Q8BTW3	Q8BTW3	Exosc6	72544	0.220283071	0.27989674	0.47967117
ShSirt1_vs_ShScrambled	Q8VDD5	Q8VDD5	Myh9	17886	0.159822251	0.2800084	0.47968043
ShSirt1_vs_ShScrambled	Q9DBR1	Q9DBR1	Xrn2	24128	-0.162894863	0.28025128	0.47974081
ShSirt1_vs_ShScrambled	Q8BZ03	Q8BZ03;Q8K	Prkd2		-0.258620271	0.28037968	0.47974081
ShSirt1_vs_ShScrambled	O88271	O88271	Cfdp1	23837	0.349537907	0.28047352	0.47974081
ShSirt1_vs_ShScrambled	Q9CPN8	Q9CPN8	Igf2bp3	140488	0.143021997	0.28052766	0.47974081

ShSirt1_vs_ShScrambled	Q6A4J8	Q6A4J8	Usp7	252870	0.174778467	0.2806643	0.47974081
ShSirt1_vs_ShScrambled	G5E829	G5E829	Atp2b1	67972	0.238357533	0.28068132	0.47974081
ShSirt1_vs_ShScrambled	Q8BTZ4	Q8BTZ4	Anapc5	59008	0.729931628	0.28108225	0.48009859
ShSirt1_vs_ShScrambled	O88811	O88811	Stam2	56324	0.239805566	0.28110336	0.48009859
ShSirt1_vs_ShScrambled	Q8CG47	Q8CG47	Smc4	70099	0.345324329	0.28123164	0.48013602
ShSirt1_vs_ShScrambled	Q3TB82	Q3TB82	Plekhf1	72287	-0.662732741	0.28167496	0.48071106
ShSirt1_vs_ShScrambled	Q8CHT3	Q8CHT3	Ints5	109077	0.386567034	0.282156	0.48135003
ShSirt1_vs_ShScrambled	Q80X50	Q80X50	Ubap2l	74383	0.158747753	0.28229528	0.4814057
ShSirt1_vs_ShScrambled	Q9CQM2	Q9CQM2;Q9	Kdelr2		0.217184448	0.28249396	0.48156259
ShSirt1_vs_ShScrambled	Q8C0D5	Q8C0D5	Eftud1	101592	0.645936167	0.28277548	0.48186051
ShSirt1_vs_ShScrambled	Q8CAQ8	Q8CAQ8	Immt	76614	-0.157406413	0.28292631	0.48193561
ShSirt1_vs_ShScrambled	Q91V87	Q91V87	Fgfrl1	116701	-0.483421386	0.28340629	0.4825711
ShSirt1_vs_ShScrambled	Q920A7	Q920A7	Afg3l1	114896	-0.247600944	0.28379303	0.48303792
ShSirt1_vs_ShScrambled	Q9D0D4	Q9D0D4	Dimt1	66254	1.210453932	0.28389446	0.48303792
ShSirt1_vs_ShScrambled	Q921C5	Q921C5	Bicd2	76895	0.805699862	0.28417415	0.48333162
ShSirt1_vs_ShScrambled	Q80VQ0	Q80VQ0	Aldh3b1	67689	0.32232303	0.2844128	0.48355532
ShSirt1_vs_ShScrambled	Q91VK1	Q91VK1	Bzw2	66912	-0.499614432	0.28501038	0.48438887
ShSirt1_vs_ShScrambled	Q91YE7	Q91YE7	Rbm5	83486	-0.717630235	0.28538578	0.48473448
ShSirt1_vs_ShScrambled	Q6PR54	Q6PR54	Rif1	51869	-0.318697812	0.2854285	0.48473448
ShSirt1_vs_ShScrambled	Q91YL3	Q91YL3	Uckl1	68556	0.192977589	0.28560743	0.48485594
ShSirt1_vs_ShScrambled	P84104	P84104	Srsf3	20383	0.271005798	0.28573163	0.48488442
ShSirt1_vs_ShScrambled	Q99LB2	Q99LB2	Dhrs4	28200	-0.179509845	0.28597035	0.48510716
ShSirt1_vs_ShScrambled	P51163	P51163	Uros	22276	0.561721153	0.28620627	0.48532498
ShSirt1_vs_ShScrambled	Q93092	Q93092	Taldo1	21351	-0.268900772	0.28656653	0.4857534
ShSirt1_vs_ShScrambled	Q3UQN2	Q3UQN2	Fcho2	218503	0.302931342	0.28678317	0.48593816
ShSirt1_vs_ShScrambled	Q9JM14	Q9JM14	Nt5c	50773	0.220053655	0.28711575	0.48631914
ShSirt1_vs_ShScrambled	Q8JZV7	Q8JZV7	Amdhd2	245847	0.219344336	0.28741509	0.48651125
ShSirt1_vs_ShScrambled	Q8K1L5	Q8K1L5	Ppp1r11	76497	0.309894794	0.28744473	0.48651125
ShSirt1_vs_ShScrambled	Q6P8I4	Q6P8I4	Pcnp	76302	0.254363423	0.28820632	0.48752098
ShSirt1_vs_ShScrambled	Q9D554	Q9D554	Sf3a3	75062	0.532202239	0.28833094	0.48752098
ShSirt1_vs_ShScrambled	Q8VE38	Q8VE38	Oxnad1	218885	0.374486714	0.28836531	0.48752098
ShSirt1_vs_ShScrambled	Q91YE6	Q91YE6	Ipo9		-0.143653061	0.28854476	0.48764173
ShSirt1_vs_ShScrambled	Q505D7	Q505D7	Opa3	403187	-1.078109267	0.28891171	0.48807914
ShSirt1_vs_ShScrambled	Q8VH51	Q8VH51	Rbm39	170791	-0.197020807	0.2890439	0.48811978
ShSirt1_vs_ShScrambled	Q99KW9	Q99KW9	Itfg1	71927	0.892561912	0.28936457	0.48847856
ShSirt1_vs_ShScrambled	P52432	P52432	Polr1c	20016	0.257592237	0.28948505	0.48849926
ShSirt1_vs_ShScrambled	Q8C4S8	Q8C4S8	Dennd2a	209773	0.474864696	0.28970789	0.4885108
ShSirt1_vs_ShScrambled	Q9Z2G6	Q9Z2G6	Sel1l	20338	0.145494551	0.28970833	0.4885108
ShSirt1_vs_ShScrambled	Q9QZE5	Q9QZE5	Copg1	54161	-0.174630483	0.29064935	0.48991455
ShSirt1_vs_ShScrambled	Q9WTI7	Q9WTI7	Myo1c	17913	0.139261516	0.29076121	0.48992016
ShSirt1_vs_ShScrambled	Q8CEI1	Q8CEI1	Bola3	78653	-0.483997283	0.2908895	0.48995343
ShSirt1_vs_ShScrambled	Q8BMS4	Q8BMS4	Coq3	230027	0.694492466	0.29117727	0.49025521
ShSirt1_vs_ShScrambled	Q8BRH4	Q8BRH4	Kmt2c		-2.15356548	0.29176742	0.49106568
ShSirt1_vs_ShScrambled	Q9D8T4	Q9D8T4	Tvp23b	67510	0.537079531	0.29256393	0.49222273
ShSirt1_vs_ShScrambled	P14685	P14685	Psmd3	22123	-0.16343562	0.29274866	0.49235002
ShSirt1_vs_ShScrambled	Q9D708	Q9D708	S100a16	67860	-0.366545648	0.29311554	0.49274883
ShSirt1_vs_ShScrambled	P97452	P97452	Bop1	12181	0.369680589	0.29320411	0.49274883
ShSirt1_vs_ShScrambled	Q9CQL4	Q9CQL4	Mrpl20	66448	-0.4292597	0.29359269	0.49321824
ShSirt1_vs_ShScrambled	Q8K4F5	Q8K4F5	Abhd11	68758	0.189914045	0.29376591	0.49332564
ShSirt1_vs_ShScrambled	Q08093	Q08093	Cnn2	12798	0.251197027	0.29433744	0.49362548
ShSirt1_vs_ShScrambled	P62751	P62751	Rpl23a	268449	0.393210618	0.29435582	0.49362548
ShSirt1_vs_ShScrambled	Q8CII2	Q8CII2	Cdc123	98828	0.337840973	0.29445518	0.49362548
ShSirt1_vs_ShScrambled	Q9EPU4	Q9EPU4	Cpsf1	94230	0.250541652	0.29452176	0.49362548
ShSirt1_vs_ShScrambled	Q7TNC4	Q7TNC4	Luc7l2	192196	-0.139621432	0.29463478	0.49362548
ShSirt1_vs_ShScrambled	P35279	P35279	Rab6a	19346	-0.235259006	0.29479648	0.49362548
ShSirt1_vs_ShScrambled	O55101	O55101	Syngr2	20973	-0.468106665	0.29480734	0.49362548
ShSirt1_vs_ShScrambled	Q921K8	Q921K8	Tcaf2	232748	-0.31029812	0.29481929	0.49362548
ShSirt1_vs_ShScrambled	Q8BH64	Q8BH64	Ehd2	259300	0.156609247	0.29526743	0.49419251
ShSirt1_vs_ShScrambled	Q6P5E4	Q6P5E4	Uggt1	320011	0.147775007	0.29542912	0.49427985
ShSirt1_vs_ShScrambled	Q9CQ80	Q9CQ80	Vps25	28084	0.626073756	0.29568047	0.49437397
ShSirt1_vs_ShScrambled	Q9D8K8	Q9D8K8	Slc25a39	68066	0.253238168	0.29570441	0.49437397
ShSirt1_vs_ShScrambled	Q9D898	Q9D898	Arpc5l	74192	0.223008482	0.29591009	0.49443184
ShSirt1_vs_ShScrambled	Q924H7	Q924H7	Wac	225131	0.780320676	0.29603925	0.49443184

ShSirt1_vs_ShScrambled	P50429	P50429	Arsb	11881	-0.345557686	0.29606763	0.49443184
ShSirt1_vs_ShScrambled	Q6PGH1	Q6PGH1	Bud31	231889	-0.520715839	0.2968728	0.49559312
ShSirt1_vs_ShScrambled	P12367	P12367	Prkar2a		0.253474427	0.29727395	0.49584997
ShSirt1_vs_ShScrambled	Q8CIE6	Q8CIE6	Copa	12847	-0.149571586	0.29728432	0.49584997
ShSirt1_vs_ShScrambled	Q921L5	Q921L5	Cog2	76332	0.151665976	0.29739836	0.49584997
ShSirt1_vs_ShScrambled	Q91ZV0	Q91ZV0	Mia2		-0.262962746	0.29746604	0.49584997
ShSirt1_vs_ShScrambled	P58252	P58252	Eef2	13629	0.137598873	0.297792	0.49609719
ShSirt1_vs_ShScrambled	Q9CXI0	Q9CXI0	Coq5	52064	0.716327965	0.29783416	0.49609719
ShSirt1_vs_ShScrambled	Q99LH2	Q99LH2	Ptdss1	19210	-0.155329012	0.29800198	0.49619363
ShSirt1_vs_ShScrambled	Q9EPC1	Q9EPC1	Parva	57342	0.206464756	0.29815253	0.49626125
ShSirt1_vs_ShScrambled	Q9CZT5	Q9CZT5	Vasn	246154	-0.499439078	0.2984638	0.49659625
ShSirt1_vs_ShScrambled	Q8BTH8	Q8BTH8	Csnk1g1	214897	-0.343698826	0.299046	0.49734884
ShSirt1_vs_ShScrambled	Q9D8N0	Q9D8N0	Eef1g	67160	0.228132758	0.29913649	0.49734884
ShSirt1_vs_ShScrambled	Q9D7G0	Q9D7G0	Prps1	19139	-0.152720613	0.29938434	0.49757766
ShSirt1_vs_ShScrambled	Q6A068	Q6A068	Cdc5l	71702	0.270432233	0.30059267	0.49940203
ShSirt1_vs_ShScrambled	Q9CPQ8	Q9CPQ8	Atp5l	27425	-0.206337918	0.3015981	0.50073746
ShSirt1_vs_ShScrambled	A8C756	A8C756	Thada	240174	0.668333743	0.30161833	0.50073746
ShSirt1_vs_ShScrambled	Q8CCS6	Q8CCS6	Pabpn1	54196	0.400498365	0.30224935	0.50129035
ShSirt1_vs_ShScrambled	Q64331	Q64331	Myo6		-0.234317413	0.30225957	0.50129035
ShSirt1_vs_ShScrambled	Q9R0E2	Q9R0E2	Plod1	18822	0.193504082	0.30228452	0.50129035
ShSirt1_vs_ShScrambled	Q8CHQ0	Q8CHQ0	Fbxo4	106052	0.200672073	0.30239693	0.5012926
ShSirt1_vs_ShScrambled	Q9D0R2	Q9D0R2	Tars	110960	0.172604724	0.30259603	0.50143851
ShSirt1_vs_ShScrambled	Q80W00	Q80W00	Ppp1r10	52040	-0.271167591	0.30273764	0.50148906
ShSirt1_vs_ShScrambled	Q6RT24	Q6RT24	Cenpe	229841	1.214144651	0.30308807	0.50188538
ShSirt1_vs_ShScrambled	Q9D8V0	Q9D8V0	Hm13	14950	-0.246916087	0.30356211	0.50243959
ShSirt1_vs_ShScrambled	Q9DCC8	Q9DCC8	Tomm20	67952	0.264579187	0.30364537	0.50243959
ShSirt1_vs_ShScrambled	Q922Q9	Q922Q9	Chid1	68038	0.30275922	0.30389138	0.5026624
ShSirt1_vs_ShScrambled	P42128	P42128	Foxk1	17425	0.28674806	0.30448424	0.50345856
ShSirt1_vs_ShScrambled	Q8CBW3	Q8CBW3	Abi1	11308	0.306984969	0.30468277	0.50360236
ShSirt1_vs_ShScrambled	Q91YI1	Q91YI1	Atg13	51897	-0.240872515	0.30508974	0.50403434
ShSirt1_vs_ShScrambled	O08915	O08915	Aip	11632	0.159822164	0.30516745	0.50403434
ShSirt1_vs_ShScrambled	P63085	P63085	Mapk1	26413	0.143341362	0.30567857	0.50437406
ShSirt1_vs_ShScrambled	Q8K2I4	Q8K2I4	Manba	110173	0.2212441	0.30569862	0.50437406
ShSirt1_vs_ShScrambled	Q60936	Q60936	Adck3	67426	-1.832800879	0.30570834	0.50437406
ShSirt1_vs_ShScrambled	B1AVZ0	B1AVZ0	Uprt	331487	0.450664299	0.3061034	0.50466729
ShSirt1_vs_ShScrambled	Q8K2C6	Q8K2C6	Sirt5	68346	1.266575664	0.30610967	0.50466729
ShSirt1_vs_ShScrambled	Q9CWG9	Q9CWG9	Bloc1s2	73689	-0.27768656	0.30638098	0.5048631
ShSirt1_vs_ShScrambled	Q3U4G3	Q3U4G3	Xxylt1	268880	0.358316643	0.30645213	0.5048631
ShSirt1_vs_ShScrambled	O35638	O35638	Stag2	20843	0.256934074	0.30662273	0.50489087
ShSirt1_vs_ShScrambled	Q8K385	Q8K385	FRRS1	20321	0.421255577	0.30669268	0.50489087
ShSirt1_vs_ShScrambled	Q9JII5	Q9JII5	Dazap1	70248	0.266094664	0.30706603	0.50512582
ShSirt1_vs_ShScrambled	O35144	O35144	Terf2	21750	1.341293073	0.30713047	0.50512582
ShSirt1_vs_ShScrambled	P42567	P42567	Eps15	13858	0.322288786	0.30717111	0.50512582
ShSirt1_vs_ShScrambled	Q8BQ30	Q8BQ30	Ppp1r18	76448	-0.725506254	0.30765943	0.50531375
ShSirt1_vs_ShScrambled	Q91VN6	Q91VN6	Ddx41	72935	0.3593349	0.30767852	0.50531375
ShSirt1_vs_ShScrambled	Q8BKT7	Q8BKT7	Thoc5	107829	0.204852144	0.30777687	0.50531375
ShSirt1_vs_ShScrambled	E9PYH6	E9PYH6	Setd1a	233904	0.297396656	0.30782496	0.50531375
ShSirt1_vs_ShScrambled	Q62419	Q62419	Sh3gl1	20405	-0.499442036	0.30784511	0.50531375
ShSirt1_vs_ShScrambled	Q9CQU3	Q9CQU3	Rer1	67830	1.007581898	0.30841972	0.50607292
ShSirt1_vs_ShScrambled	P10107	P10107	Anxa1	16952	-0.133381162	0.30869969	0.50618184
ShSirt1_vs_ShScrambled	P97930	P97930	Dtymk	21915	0.204799191	0.30871037	0.50618184
ShSirt1_vs_ShScrambled	Q91WL8	Q91WL8	Wwox	80707	0.44984651	0.30914598	0.50670442
ShSirt1_vs_ShScrambled	Q9CWS4	Q9CWS4	Cpsf3l	71957	-0.179486869	0.30925358	0.50670442
ShSirt1_vs_ShScrambled	P47955	P47955	Rplp1	56040	0.162713625	0.30946311	0.50681744
ShSirt1_vs_ShScrambled	Q91YT7	Q91YT7	Ythdf2	213541	-0.290125098	0.30956903	0.50681744
ShSirt1_vs_ShScrambled	Q9CY97	Q9CY97	Ssu72	68991	0.289835382	0.30965939	0.50681744
ShSirt1_vs_ShScrambled	P37040	P37040	Por	18984	-0.154500704	0.30978609	0.50684103
ShSirt1_vs_ShScrambled	Q9CWG8	Q9CWG8	Ndufaf7	73694	-0.220021282	0.3107301	0.50820133
ShSirt1_vs_ShScrambled	Q9DC50	Q9DC50	Crot	74114	0.490736312	0.31119452	0.50847028
ShSirt1_vs_ShScrambled	O54946	O54946	Dnajb6	23950	-0.247300711	0.31120165	0.50847028
ShSirt1_vs_ShScrambled	E9PYK3	E9PYK3	Parp4	328417	-0.508753003	0.31123248	0.50847028
ShSirt1_vs_ShScrambled	Q7TSC1	Q7TSC1	Prrc2a	53761	0.163566501	0.31143108	0.50861067
ShSirt1_vs_ShScrambled	A2RSX7	A2RSX7	Tyw5	68736	0.446602706	0.31178118	0.50899827

ShSirt1_vs_ShScrambled	Q8VE11	Q8VE11	Mtmr6	219135	-0.429065288	0.31193891	0.50907167
ShSirt1_vs_ShScrambled	Q9QWV9	Q9QWV9	Ccnt1	12455	0.230894637	0.31248867	0.50978456
ShSirt1_vs_ShScrambled	Q8BG81	Q8BG81	Poldip3	73826	0.149955643	0.31262287	0.50981924
ShSirt1_vs_ShScrambled	Q9CR88	Q9CR88	Mrps14	64659	-1.682838228	0.31301299	0.51027108
ShSirt1_vs_ShScrambled	Q61216	Q61216	Mre11a	17535	-0.93599693	0.31385905	0.51120805
ShSirt1_vs_ShScrambled	Q8CI32	Q8CI32	Bag5	70369	-0.28664875	0.31387744	0.51120805
ShSirt1_vs_ShScrambled	Q04899	Q04899	Cdk18	18557	0.251508251	0.31393494	0.51120805
ShSirt1_vs_ShScrambled	A2APY7	A2APY7	Ndufaf5	69487	0.263646566	0.31404075	0.51120805
ShSirt1_vs_ShScrambled	Q9CQV8	Q9CQV8	Ywhab	54401	-0.23638952	0.31425664	0.51137508
ShSirt1_vs_ShScrambled	O55060	O55060	Tpmt	22017	-0.800923063	0.31537213	0.51300533
ShSirt1_vs_ShScrambled	Q9Z1Q5	Q9Z1Q5	Clic1	114584	0.158415489	0.31593145	0.51353519
ShSirt1_vs_ShScrambled	Q921Q3	Q921Q3	Alg1	208211	0.214944945	0.31598608	0.51353519
ShSirt1_vs_ShScrambled	P61021	P61021	Rab5b	19344	0.166670166	0.31603916	0.51353519
ShSirt1_vs_ShScrambled	Q921S7	Q921S7	Mrpl37	56280	0.702283765	0.31656488	0.51420434
ShSirt1_vs_ShScrambled	Q9Z1Z0	Q9Z1Z0	Uso1	56041	-0.142094596	0.31668068	0.51420741
ShSirt1_vs_ShScrambled	Q8BH74	Q8BH74	Nup107	103468	0.307602805	0.31718778	0.51484561
ShSirt1_vs_ShScrambled	O35134	O35134	Polr1a	20019	0.486977736	0.31755907	0.515263
ShSirt1_vs_ShScrambled	Q8CI61	Q8CI61	Bag4	67384	-0.300839458	0.31771435	0.51532971
ShSirt1_vs_ShScrambled	Q8VEM8	Q8VEM8	Slc25a3	18674	-0.160747538	0.31795757	0.51552322
ShSirt1_vs_ShScrambled	Q91XU3	Q91XU3	Pip4k2c	117150	0.197357487	0.31806207	0.51552322
ShSirt1_vs_ShScrambled	Q5DTM8	Q5DTM8	Rnf20	109331	0.314682258	0.31834864	0.51571113
ShSirt1_vs_ShScrambled	P58137	P58137	Acot8	170789	-0.210917533	0.31840649	0.51571113
ShSirt1_vs_ShScrambled	Q3TYS2	Q3TYS2	Q3TYS2	217370	0.235406952	0.31855651	0.51576904
ShSirt1_vs_ShScrambled	Q9D773	Q9D773	Mrpl2	27398	-0.227478553	0.31942742	0.51699368
ShSirt1_vs_ShScrambled	P26043	P26043	Rdx	19684	0.200193169	0.31954198	0.51699372
ShSirt1_vs_ShScrambled	Q9CWZ3	Q9CWZ3	Rbm8a	60365	0.173912664	0.31975531	0.51707701
ShSirt1_vs_ShScrambled	P42208	P42208	Sept2	18000	-0.165593982	0.31982255	0.51707701
ShSirt1_vs_ShScrambled	Q9JHJ0	Q9JHJ0	Tmod3	50875	-0.19177789	0.31998274	0.51715076
ShSirt1_vs_ShScrambled	P16125	P16125	Ldhb	16832	-0.127781807	0.32109901	0.51876912
ShSirt1_vs_ShScrambled	Q99J27	Q99J27	Slc33a1	11416	0.320058283	0.32152879	0.5190967
ShSirt1_vs_ShScrambled	Q3TC93	Q3TC93	Hs1bp3	58240	0.410261247	0.32153176	0.5190967
ShSirt1_vs_ShScrambled	O70572	O70572	Smpd2	20598	-0.203151149	0.32191159	0.51952411
ShSirt1_vs_ShScrambled	Q99KP6	Q99KP6	Prpf19	28000	0.161219566	0.32239033	0.51982657
ShSirt1_vs_ShScrambled	P97461	P97461	Rps5	20103	-0.160120358	0.32242301	0.51982657
ShSirt1_vs_ShScrambled	Q8CIE4	Q8CIE4	Parp10	671535	0.41597099	0.32255855	0.51982657
ShSirt1_vs_ShScrambled	P68510	P68510	Ywhah	22629	0.239425977	0.32267702	0.51982657
ShSirt1_vs_ShScrambled	P62858	P62858	Rps28	54127	-0.523507512	0.32270753	0.51982657
ShSirt1_vs_ShScrambled	Q91ZU6	Q91ZU6	Dst	13518	-0.279699621	0.32283563	0.51982657
ShSirt1_vs_ShScrambled	Q61164	Q61164	Ctcf	13018	0.341716926	0.32298894	0.51982657
ShSirt1_vs_ShScrambled	Q6NSU3	Q6NSU3	Glt8d1	76485	0.348983688	0.32302028	0.51982657
ShSirt1_vs_ShScrambled	Q9WUN2	Q9WUN2	Tbk1	56480	-0.260659648	0.32335525	0.52018018
ShSirt1_vs_ShScrambled	Q64008	Q64008	Rab34	19376	-1.053639817	0.32398349	0.52100516
ShSirt1_vs_ShScrambled	Q9QYC0	Q9QYC0	Add1	11518	0.147699005	0.32449731	0.52164561
ShSirt1_vs_ShScrambled	Q8CDM8	Q8CDM8	Fam160b1	226252	-0.368521224	0.32497732	0.52211264
ShSirt1_vs_ShScrambled	Q9CQV5	Q9CQV5	Mrps24	64660	-0.137271846	0.32501916	0.52211264
ShSirt1_vs_ShScrambled	Q8C0L9	Q8C0L9	Gpcpd1	74182	-0.213053231	0.32610974	0.52367818
ShSirt1_vs_ShScrambled	Q9CYD3	Q9CYD3	Crtap	56693	-0.267096012	0.32625816	0.5237302
ShSirt1_vs_ShScrambled	P61255	P61255	Rpl26	19941	-0.193954077	0.32676756	0.52436145
ShSirt1_vs_ShScrambled	Q8R3D1	Q8R3D1	Tbc1d13	70296	0.422966699	0.32696911	0.52444143
ShSirt1_vs_ShScrambled	Q8C5H8	Q8C5H8	Nadk2	68646	0.380363683	0.32704976	0.52444143
ShSirt1_vs_ShScrambled	Q9Z2C4	Q9Z2C4	Mtmr1	53332	0.840224045	0.32771632	0.52500755
ShSirt1_vs_ShScrambled	Q8BRK9	Q8BRK9	Man2a2	140481	0.253505887	0.32773346	0.52500755
ShSirt1_vs_ShScrambled	Q6ZWZ2	Q6ZWZ2	Ube2r2	67615	-0.255022435	0.32775172	0.52500755
ShSirt1_vs_ShScrambled	Q8CHY3	Q8CHY3	Dym	69190	-0.346551909	0.32789717	0.52505421
ShSirt1_vs_ShScrambled	Q9R1P0	Q9R1P0	Psma4	26441	0.141999739	0.32879369	0.5263031
ShSirt1_vs_ShScrambled	Q64669	Q64669	Nqo1	18104	-0.262396503	0.32967243	0.52752264
ShSirt1_vs_ShScrambled	Q3UH06	Q3UH06	Rreb1	68750	0.425036294	0.33000629	0.5277948
ShSirt1_vs_ShScrambled	O54984	O54984	Asna1	56495	-0.27469334	0.33007637	0.5277948
ShSirt1_vs_ShScrambled	Q9CQR6	Q9CQR6	Ppp6c	67857	0.220151943	0.33056354	0.52838663
ShSirt1_vs_ShScrambled	P56383	P56383;Q9C	Atp5g2		-1.308527145	0.33090824	0.52875036
ShSirt1_vs_ShScrambled	Q8R2U6	Q8R2U6	Nudt4	71207	0.422066011	0.33261508	0.53128962
ShSirt1_vs_ShScrambled	Q3TUH1	Q3TUH1	Tamm41		-0.219126413	0.33306952	0.53163741
ShSirt1_vs_ShScrambled	O55029	O55029	Copb2	50797	0.125391636	0.33310109	0.53163741

ShSirt1_vs_ShScrambled	O08573	O08573	Lgals9	16859	0.528283285	0.33324261	0.53163741
ShSirt1_vs_ShScrambled	Q8CCN5	Q8CCN5	Bcas3	192197	-1.276131265	0.33330391	0.53163741
ShSirt1_vs_ShScrambled	Q8BPA8	Q8BPA8	Dpcd	226162	2.432577326	0.33380225	0.5322442
ShSirt1_vs_ShScrambled	O54950	O54950	Prkag1	19082	-0.22006051	0.33392167	0.53224662
ShSirt1_vs_ShScrambled	O35250	O35250	Exoc7	53413	0.194162161	0.33414208	0.53240994
ShSirt1_vs_ShScrambled	Q0P5W1	Q0P5W1	Vps8	209018	0.249101377	0.33464048	0.53295974
ShSirt1_vs_ShScrambled	Q91Z38	Q91Z38	Ttc1	66827	-0.381255595	0.33472327	0.53295974
ShSirt1_vs_ShScrambled	O70325	O70325	Gpx4	625249	0.548216596	0.33504504	0.53324306
ShSirt1_vs_ShScrambled	A2AGT5	A2AGT5	Ckap5	75786	0.21424474	0.33513748	0.53324306
ShSirt1_vs_ShScrambled	Q9CQJ6	Q9CQJ6	Denr	68184	0.170328832	0.33528843	0.53329527
ShSirt1_vs_ShScrambled	Q9CXJ1	Q9CXJ1	Ears2	67417	-0.218744375	0.33568127	0.53373203
ShSirt1_vs_ShScrambled	P97300	P97300	Nptn	20320	0.241590757	0.33585017	0.53381257
ShSirt1_vs_ShScrambled	Q62422	Q62422	Ostf1	20409	0.219930661	0.33622976	0.53422583
ShSirt1_vs_ShScrambled	P62192	P62192	Psmc1	19179	-0.124524934	0.33634688	0.53422583
ShSirt1_vs_ShScrambled	Q6ZWR6	Q6ZWR6	Syne1	64009	-0.432689896	0.33691235	0.53468469
ShSirt1_vs_ShScrambled	Q80TY0	Q80TY0	Fnbp1	14269	0.35082509	0.33701651	0.53468469
ShSirt1_vs_ShScrambled	Q99LB6	Q99LB6	Mat2b	108645	-0.158630554	0.33708275	0.53468469
ShSirt1_vs_ShScrambled	Q9D1E6	Q9D1E6	Tbcb	66411	-0.125392491	0.3371658	0.53468469
ShSirt1_vs_ShScrambled	Q9CZ13	Q9CZ13	Uqcrc1	22273	-0.14818772	0.33722803	0.53468469
ShSirt1_vs_ShScrambled	Q9D8L5	Q9D8L5	Ccdc91	67015	-0.788553456	0.33768152	0.53521573
ShSirt1_vs_ShScrambled	Q9D0J8	Q9D0J8	Ptms	69202	0.31370415	0.33798908	0.53551516
ShSirt1_vs_ShScrambled	Q9CX00	Q9CX00	Ist1	71955	1.292546379	0.3382209	0.5355225
ShSirt1_vs_ShScrambled	P09411	P09411	Pgk1	18655	0.151252252	0.33823098	0.5355225
ShSirt1_vs_ShScrambled	Q9ES00	Q9ES00	Ube4b	63958	0.261556852	0.33858073	0.53577097
ShSirt1_vs_ShScrambled	Q6ZWY3	Q6ZWY3	Rps27l	67941	-0.480372286	0.3386253	0.53577097
ShSirt1_vs_ShScrambled	Q9D125	Q9D125	Mrps25	64658	0.294338751	0.3391018	0.53633691
ShSirt1_vs_ShScrambled	Q71LX4	Q71LX4	Tln2	70549	-0.250281154	0.33926559	0.53634065
ShSirt1_vs_ShScrambled	Q58A65	Q58A65	Spag9	70834	-0.167041653	0.3393418	0.53634065
ShSirt1_vs_ShScrambled	P47791	P47791	Gsr	14782	0.124810971	0.33968282	0.53669172
ShSirt1_vs_ShScrambled	Q99MI1	Q99MI1	Erc1	111173	0.173879082	0.33990721	0.53685834
ShSirt1_vs_ShScrambled	Q7TQ95	Q7TQ95	Lnp	69605	-0.191627783	0.34005868	0.53690972
ShSirt1_vs_ShScrambled	Q61035	Q61035	Hars	15115	0.153135836	0.34049469	0.53736787
ShSirt1_vs_ShScrambled	Q8K363	Q8K363	Ddx18	66942	0.336917886	0.34058695	0.53736787
ShSirt1_vs_ShScrambled	O88487	O88487	Dync1i2	13427	-0.177691699	0.34090351	0.53758653
ShSirt1_vs_ShScrambled	P10649	P10649	Gstm1	14862	-0.408601042	0.34096372	0.53758653
ShSirt1_vs_ShScrambled	O35465	O35465	Fkbp8	14232	0.505932683	0.3413594	0.53802247
ShSirt1_vs_ShScrambled	Q9DCW4	Q9DCW4	Etfb	110826	-0.121816645	0.34154409	0.53807291
ShSirt1_vs_ShScrambled	Q9Z2N8	Q9Z2N8	Actl6a	56456	-0.138370463	0.34162981	0.53807291
ShSirt1_vs_ShScrambled	Q8C8U0	Q8C8U0	Ppfibp1	67533	-0.175607306	0.34184931	0.5382008
ShSirt1_vs_ShScrambled	Q505F5	Q505F5	Lrrc47	72946	0.128195106	0.34194947	0.5382008
ShSirt1_vs_ShScrambled	P56183	P56183	Rrp1	18114	0.300180792	0.34218196	0.53837901
ShSirt1_vs_ShScrambled	Q99LC5	Q99LC5	Etfa	110842	-0.141205959	0.34317622	0.53968914
ShSirt1_vs_ShScrambled	P19324	P19324	Serpinh1	12406	0.128200665	0.34325377	0.53968914
ShSirt1_vs_ShScrambled	Q3URS9	Q3URS9	Ccdc51	66658	0.181711086	0.34348705	0.53986788
ShSirt1_vs_ShScrambled	Q9CZM2	Q9CZM2	Rpl15	66480	0.340311205	0.34394685	0.54024393
ShSirt1_vs_ShScrambled	Q9CYN2	Q9CYN2	Spcs2	66624	-0.647700101	0.34396567	0.54024393
ShSirt1_vs_ShScrambled	P10630	P10630	Eif4a2	13682	0.237757619	0.34426202	0.5405213
ShSirt1_vs_ShScrambled	P35282	P35282	Rab21	216344	-0.311955399	0.3447434	0.54078265
ShSirt1_vs_ShScrambled	Q9Z1B3	Q9Z1B3	Plcb1	18795	0.121624131	0.34484147	0.54078265
ShSirt1_vs_ShScrambled	Q6GQT9	Q6GQT9	Nomo1	211548	-0.150686884	0.3449811	0.54078265
ShSirt1_vs_ShScrambled	Q3URQ0	Q3URQ0	Tex10	269536	-0.241611656	0.34505708	0.54078265
ShSirt1_vs_ShScrambled	Q9CPT3	Q9CPT3	Nanp	67311	-0.185939963	0.3451456	0.54078265
ShSirt1_vs_ShScrambled	Q8R0G9	Q8R0G9	Nup133	234865	0.160270556	0.34514728	0.54078265
ShSirt1_vs_ShScrambled	P60670	P60670	Nploc4	217365	0.317315901	0.34540968	0.541006
ShSirt1_vs_ShScrambled	Q8BX10	Q8BX10	Pgam5	72542	-0.225336072	0.34594106	0.54150585
ShSirt1_vs_ShScrambled	Q6P2K6	Q6P2K6	Smek1	68734	-0.587680605	0.34596874	0.54150585
ShSirt1_vs_ShScrambled	Q91VA6	Q91VA6	Poldip2	67811	-0.143610448	0.34631293	0.54185669
ShSirt1_vs_ShScrambled	Q64378	Q64378	Fkbp5	14229	0.186279935	0.34713019	0.54285484
ShSirt1_vs_ShScrambled	Q69ZQ2	Q69ZQ2	Isy1	57905	0.276664779	0.34719139	0.54285484
ShSirt1_vs_ShScrambled	Q8K3J1	Q8K3J1	Ndufs8	225887	0.620584733	0.34736882	0.54294421
ShSirt1_vs_ShScrambled	Q9CR41	Q9CR41	Hypk	67693	0.233271282	0.34782812	0.54340543
ShSirt1_vs_ShScrambled	P58389	P58389	Ppp2r4	110854	0.156449594	0.34790467	0.54340543
ShSirt1_vs_ShScrambled	Q3TIU4	Q3TIU4	Pde12	211948	0.500784124	0.34870395	0.54446546

ShSirt1_vs_ShScrambled	Q7TSH2	Q7TSH2	Phkb	102093	-0.470233481	0.34907931	0.54457312
ShSirt1_vs_ShScrambled	Q6PE15	Q6PE15	Abhd10	213012	-0.227677384	0.34910682	0.54457312
ShSirt1_vs_ShScrambled	Q6TXD4	Q6TXD4	Dnmbp	71972	0.875546823	0.34913482	0.54457312
ShSirt1_vs_ShScrambled	Q9D6T0	Q9D6T0	Nosip	66394	0.411830727	0.34991763	0.54551289
ShSirt1_vs_ShScrambled	Q62418	Q62418	Dbnl	13169	-0.198378135	0.34997903	0.54551289
ShSirt1_vs_ShScrambled	Q7TSH9	Q7TSH9	Zfp184	193452	1.418375442	0.35017935	0.54563673
ShSirt1_vs_ShScrambled	B1AZA5	B1AZA5	Tmem245		0.294688434	0.35057709	0.54582628
ShSirt1_vs_ShScrambled	P48722	P48722	Hspa4l	18415	-0.245412329	0.3506263	0.54582628
ShSirt1_vs_ShScrambled	Q8VDD9	Q8VDD9	Phip		-0.213960104	0.35066376	0.54582628
ShSirt1_vs_ShScrambled	O54825	O54825	Bysl	53414	0.307738199	0.35083984	0.54591212
ShSirt1_vs_ShScrambled	O88622	O88622	Parg		-0.355421076	0.35138923	0.54657856
ShSirt1_vs_ShScrambled	Q6P6M5	Q6P6M5	Pex11g	69129	0.373885997	0.35170732	0.54688489
ShSirt1_vs_ShScrambled	Q8BVL3	Q8BVL3	Snx17	266781	0.577658309	0.35206855	0.54725806
ShSirt1_vs_ShScrambled	Q9CXY6	Q9CXY6	Ilf2	67781	0.166387582	0.35246723	0.54768918
ShSirt1_vs_ShScrambled	Q99K85	Q99K85	Psat1	107272	0.151851793	0.35273961	0.54792382
ShSirt1_vs_ShScrambled	Q8BZ98	Q8BZ98	Dnm3	103967	-1.445466245	0.35314468	0.54836432
ShSirt1_vs_ShScrambled	Q80SZ7	Q80SZ7	Gng5	14707	-0.975389701	0.35330211	0.54842013
ShSirt1_vs_ShScrambled	Q52KE7	Q52KE7	Ccnl1	56706	0.834837271	0.35358731	0.54867415
ShSirt1_vs_ShScrambled	P19182	P19182	Ifrd1	15982	0.218822274	0.35409749	0.549277
ShSirt1_vs_ShScrambled	Q99KD5	Q99KD5	Unc45a	101869	0.411718883	0.35525771	0.55088743
ShSirt1_vs_ShScrambled	Q9D5V5	Q9D5V5	Cul5	75717	-0.162924176	0.35572145	0.55101682
ShSirt1_vs_ShScrambled	O55128	O55128	Sap18	20220	0.232308803	0.35580943	0.55101682
ShSirt1_vs_ShScrambled	Q64012	Q64012	Raly	19383	-0.329316005	0.35581821	0.55101682
ShSirt1_vs_ShScrambled	Q9CPU0	Q9CPU0	Glo1	109801	-0.169206669	0.35582943	0.55101682
ShSirt1_vs_ShScrambled	Q8BJW6	Q8BJW6	Eif2a	229317	0.208402635	0.35616036	0.55134015
ShSirt1_vs_ShScrambled	Q3TZX8	Q3TZX8	Nol9	74035	0.370591946	0.35637277	0.55147984
ShSirt1_vs_ShScrambled	Q9JKV1	Q9JKV1	Adrm1	56436	0.144915158	0.35650987	0.55150293
ShSirt1_vs_ShScrambled	Q91YD9	Q91YD9	Wasl	73178	0.270228594	0.35687909	0.55188496
ShSirt1_vs_ShScrambled	O55142	O55142	Rpl35a	57808	-0.223091074	0.35760668	0.55282074
ShSirt1_vs_ShScrambled	Q80ZK9	Q80ZK9	Wdtc1	230796	0.765766966	0.35780871	0.55288042
ShSirt1_vs_ShScrambled	Q9D819	Q9D819	Ppa1	67895	-0.195472525	0.35799056	0.55288042
ShSirt1_vs_ShScrambled	Q5KU39	Q5KU39	Vps41	218035	-0.335508475	0.35801273	0.55288042
ShSirt1_vs_ShScrambled	E9QAT4	E9QAT4	Sec16a	227648	-0.198544637	0.35836845	0.55314829
ShSirt1_vs_ShScrambled	Q8R1B4	Q8R1B4	Eif3c	56347	0.116079045	0.35843127	0.55314829
ShSirt1_vs_ShScrambled	Q0VBL3	Q0VBL3	Rbm15	229700	0.138697929	0.35872729	0.5533337
ShSirt1_vs_ShScrambled	Q80UY2	Q80UY2	Kcmf1	74287	0.196924207	0.35882153	0.5533337
ShSirt1_vs_ShScrambled	O35598	O35598	Adam10	11487	-0.276768739	0.35891915	0.5533337
ShSirt1_vs_ShScrambled	Q91YU6	Q91YU6	Lzts2	226154	-0.258592271	0.35907754	0.55338887
ShSirt1_vs_ShScrambled	P62313	P62313	Lsm6	78651	0.190644603	0.35987947	0.5543482
ShSirt1_vs_ShScrambled	Q80XQ2	Q80XQ2	Tbc1d5	72238	0.197439284	0.35994563	0.5543482
ShSirt1_vs_ShScrambled	O35604	O35604	Npc1	18145	0.219238791	0.36144151	0.55646213
ShSirt1_vs_ShScrambled	P31750	P31750	Akt1	11651	-0.156604967	0.36166302	0.55661332
ShSirt1_vs_ShScrambled	Q8R1A4	Q8R1A4	Dock7		0.27975245	0.36192355	0.55679652
ShSirt1_vs_ShScrambled	Q61166	Q61166	Mapre1	13589	-0.141975538	0.36209631	0.55679652
ShSirt1_vs_ShScrambled	Q8R418	Q8R418	Dicer1		0.782476759	0.3621521	0.55679652
ShSirt1_vs_ShScrambled	O88447	O88447	Klc1		0.158389043	0.36318883	0.55799044
ShSirt1_vs_ShScrambled	Q8BYK8	Q8BYK8	Zc3h6		1.149284306	0.36330727	0.55799044
ShSirt1_vs_ShScrambled	Q5SNZ0	Q5SNZ0	Ccdc88a	108686	-0.270582532	0.36336812	0.55799044
ShSirt1_vs_ShScrambled	Q6NV83	Q6NV83	U2surp	67958	0.397474283	0.36357798	0.55799044
ShSirt1_vs_ShScrambled	Q8R1T1	Q8R1T1	Chmp7	105513	0.261214394	0.36366465	0.55799044
ShSirt1_vs_ShScrambled	P47199	P47199	Cryz	12972	0.232718383	0.36367033	0.55799044
ShSirt1_vs_ShScrambled	Q9D289	Q9D289	Trappc6b	78232	0.166405219	0.3638747	0.55811431
ShSirt1_vs_ShScrambled	Q8R1Q9	Q8R1Q9	Rbks	71336	-0.305511016	0.3642577	0.55851197
ShSirt1_vs_ShScrambled	Q61136	Q61136	Prpf4b	19134	0.291338182	0.36459384	0.55883756
ShSirt1_vs_ShScrambled	P09602	P09602	Hmgn2	15331	0.967859176	0.36575561	0.56042798
ShSirt1_vs_ShScrambled	Q6P542	Q6P542	Abcf1	224742	-0.214205487	0.36606074	0.56064803
ShSirt1_vs_ShScrambled	Q80YV3	Q80YV3	Trrap		0.158147097	0.36627299	0.56064803
ShSirt1_vs_ShScrambled	Q9CZH3	Q9CZH3	Psmg3	66506	0.500399191	0.36631341	0.56064803
ShSirt1_vs_ShScrambled	P23506	P23506	Pcmt1		0.119122246	0.36639603	0.56064803
ShSirt1_vs_ShScrambled	Q9CQ45	Q9CQ45	Nenf	66208	-0.326892677	0.36829962	0.56334331
ShSirt1_vs_ShScrambled	Q9D4H2	Q9D4H2	Gcc1	74375	0.180425679	0.36849992	0.56334331
ShSirt1_vs_ShScrambled	Q8BX94	Q8BX94	Osbpl2	228983	0.245920257	0.36859791	0.56334331
ShSirt1_vs_ShScrambled	P62071	P62071	Rras2	66922	0.147722855	0.36874495	0.56334331

ShSirt1_vs_ShScrambled	Q9CX13	Q9CX13	Cnih4	98417	-0.278618571	0.36878145	0.56334331
ShSirt1_vs_ShScrambled	Q9CPV3	Q9CPV3	Mrpl42	67270	0.297814658	0.369391	0.56408355
ShSirt1_vs_ShScrambled	Q9JM52	Q9JM52	Mink1	50932	0.46501873	0.37002687	0.56484891
ShSirt1_vs_ShScrambled	Q3TWW8	Q3TWW8	Srsf6	67996	0.176666916	0.37014246	0.56484891
ShSirt1_vs_ShScrambled	Q9CQZ5	Q9CQZ5	Ndufa6	67130	-0.143521674	0.37079472	0.56560388
ShSirt1_vs_ShScrambled	Q8CH25	Q8CH25	Sltm	66660	0.62995198	0.37088779	0.56560388
ShSirt1_vs_ShScrambled	P35288	P35288	Rab23		-0.300273257	0.37108556	0.56571436
ShSirt1_vs_ShScrambled	Q9D7W5	Q9D7W5	Med8	80509	-0.298943734	0.37143017	0.56573284
ShSirt1_vs_ShScrambled	Q8VD65	Q8VD65	Pik3r4	75669	-0.211689914	0.37144343	0.56573284
ShSirt1_vs_ShScrambled	Q9Z2M7	Q9Z2M7	Pmm2	54128	0.149792921	0.37147367	0.56573284
ShSirt1_vs_ShScrambled	P41105	P41105	Rpl28	19943	-0.185297898	0.37172503	0.56592472
ShSirt1_vs_ShScrambled	P21126	P21126	Ubl4a	27643; 10	0.142422804	0.37224153	0.56634732
ShSirt1_vs_ShScrambled	P14211	P14211	Calr	12317	-0.174199918	0.37225354	0.56634732
ShSirt1_vs_ShScrambled	P24472	P24472	Gsta4	14860	-0.489250434	0.3725047	0.56653849
ShSirt1_vs_ShScrambled	P63323	P63323	Rps12		-0.270727677	0.37269769	0.56664109
ShSirt1_vs_ShScrambled	O55047	O55047	Tlk2	24086	0.558301026	0.3730697	0.56686365
ShSirt1_vs_ShScrambled	P61600	P61600	Naa20	67877	-0.139509991	0.37311624	0.56686365
ShSirt1_vs_ShScrambled	P70218	P70218	Map4k1	26411	0.262699263	0.37322082	0.56686365
ShSirt1_vs_ShScrambled	Q9D273	Q9D273	Mmab	77697	0.208108316	0.3737946	0.56735334
ShSirt1_vs_ShScrambled	Q9Z2A5	Q9Z2A5	Ate1	11907	-0.451064585	0.37390277	0.56735334
ShSirt1_vs_ShScrambled	Q9JKR6	Q9JKR6	Hyou1	12282	-0.135063759	0.37392029	0.56735334
ShSirt1_vs_ShScrambled	Q5U430	Q5U430	Ubr3	68795	-0.271897057	0.37425897	0.56767641
ShSirt1_vs_ShScrambled	Q8BJ37	Q8BJ37	Tdp1		0.613649111	0.37450293	0.56767883
ShSirt1_vs_ShScrambled	Q8BW10	Q8BW10	Nob1	67619	-0.207044051	0.37451209	0.56767883
ShSirt1_vs_ShScrambled	Q9QWH1	Q9QWH1	Phc2	54383	-0.467128563	0.37482406	0.56796099
ShSirt1_vs_ShScrambled	P47968	P47968	Rpia	19895	0.163130978	0.37522644	0.56837992
ShSirt1_vs_ShScrambled	P18653	P18653	Rps6ka1	20111	-0.258193873	0.3758839	0.56918481
ShSirt1_vs_ShScrambled	Q8VEJ4	Q8VEJ4	Nle1	217011	-1.00787247	0.37666081	0.57016998
ShSirt1_vs_ShScrambled	Q8C6I2	Q8C6I2	Sdhaf2	66072	-0.326663833	0.37719746	0.57068041
ShSirt1_vs_ShScrambled	Q8N9S3	Q8N9S3	Ahsa2	268390	-0.41514339	0.37725085	0.57068041
ShSirt1_vs_ShScrambled	Q8BHT6	Q8BHT6	B3galtl	381694	0.325968017	0.37750395	0.57083447
ShSirt1_vs_ShScrambled	Q99LI7	Q99LI7	Cstf3	228410	0.262749186	0.37760561	0.57083447
ShSirt1_vs_ShScrambled	Q8BK08	Q8BK08	Tmem11	216821	-0.303921201	0.3779546	0.57117076
ShSirt1_vs_ShScrambled	Q9CR51	Q9CR51	Atp6v1g1	66290	0.564992955	0.37864053	0.57201585
ShSirt1_vs_ShScrambled	Q8K2Y9	Q8K2Y9	Ccm2	216527	-0.200947381	0.37908507	0.57237172
ShSirt1_vs_ShScrambled	P26350	P26350	Ptma	19231	0.184851261	0.3791297	0.57237172
ShSirt1_vs_ShScrambled	Q61194	Q61194	Pik3c2a	18704	-0.518699056	0.37942712	0.57262586
ShSirt1_vs_ShScrambled	Q8C4J7	Q8C4J7	Tbl3	213773	0.605317052	0.37955175	0.57262586
ShSirt1_vs_ShScrambled	Q3U7U3	Q3U7U3	Fbxo7	69754	-0.372436944	0.37999459	0.57310244
ShSirt1_vs_ShScrambled	Q9CWW6	Q9CWW6	Pin4	69713	0.772741166	0.38014485	0.57313756
ShSirt1_vs_ShScrambled	Q5RKZ7	Q5RKZ7	Mocs1	56738	-0.145994864	0.38044077	0.5733922
ShSirt1_vs_ShScrambled	Q99J99	Q99J99	Mpst		-0.183138313	0.38126197	0.5744381
ShSirt1_vs_ShScrambled	Q8BH15	Q8BH15	Cnot10	78893	-0.126113772	0.38161133	0.57473661
ShSirt1_vs_ShScrambled	P80318	P80318	Cct3	12462	0.134630582	0.38171474	0.57473661
ShSirt1_vs_ShScrambled	Q9Z0P5	Q9Z0P5	Twf2	23999	-0.220974547	0.38192681	0.57486415
ShSirt1_vs_ShScrambled	Q9JIF0	Q9JIF0	Prmt1	15469	-0.18250982	0.38273484	0.57588835
ShSirt1_vs_ShScrambled	Q61165	Q61165	Slc9a1	20544	-0.343511243	0.38326186	0.57600203
ShSirt1_vs_ShScrambled	Q6PGF7	Q6PGF7	Exoc8	102058	0.659521994	0.3833196	0.57600203
ShSirt1_vs_ShScrambled	Q9CPY7	Q9CPY7	Lap3	66988	0.136424792	0.38345267	0.57600203
ShSirt1_vs_ShScrambled	Q91W50	Q91W50	Csde1	229663	-0.111973268	0.38353335	0.57600203
ShSirt1_vs_ShScrambled	Q6P9Q6	Q6P9Q6	Fkbp15	338355	0.222579037	0.38360259	0.57600203
ShSirt1_vs_ShScrambled	Q9QXD8	Q9QXD8	Limd1	29806	0.248200459	0.38362343	0.57600203
ShSirt1_vs_ShScrambled	Q7TPM1	Q7TPM1	Prrc2b	227723	-0.386646931	0.38371077	0.57600203
ShSirt1_vs_ShScrambled	P63037	P63037	Dnaja1	15502	-0.228916225	0.38383122	0.57600203
ShSirt1_vs_ShScrambled	Q62241	Q62241	Snrpc	20630	-0.290988244	0.38402334	0.57609187
ShSirt1_vs_ShScrambled	O08738	O08738	Casp6	12368	0.483294249	0.38414642	0.57609187
ShSirt1_vs_ShScrambled	P62892	P62892	Rpl39	67248	-0.145397317	0.38441036	0.57609187
ShSirt1_vs_ShScrambled	Q9CQU5	Q9CQU5	Zwint	52696	0.951974455	0.38442885	0.57609187
ShSirt1_vs_ShScrambled	Q9CYG7	Q9CYG7	Tomm34	67145	0.254322364	0.3845292	0.57609187
ShSirt1_vs_ShScrambled	Q8VBT0	Q8VBT0	Tmx1	72736	-0.406519552	0.38480335	0.57631133
ShSirt1_vs_ShScrambled	P14869	P14869	Rplp0	11837	0.121651063	0.38503693	0.57646988
ShSirt1_vs_ShScrambled	Q99JX3	Q99JX3	Gorasp2	70231	0.213944511	0.38624769	0.57809088
ShSirt1_vs_ShScrambled	O70251	O70251	Eef1b	55949	0.183360259	0.38667667	0.5785411

ShSirt1_vs_ShScrambled	Q9QYY8	Q9QYY8	Spast	50850	-0.539096706	0.38745481	0.57926999
ShSirt1_vs_ShScrambled	Q921H9	Q921H9	Coa7	69893	0.654700442	0.38746933	0.57926999
ShSirt1_vs_ShScrambled	O35345	O35345	Kpna6	16650	-0.236748074	0.38754882	0.57926999
ShSirt1_vs_ShScrambled	Q5SVQ0	Q5SVQ0	Kat7	217127	-0.612893687	0.3889273	0.58113798
ShSirt1_vs_ShScrambled	Q8C0L8	Q8C0L8	Cog5	238123	0.229827277	0.39029743	0.58299225
ShSirt1_vs_ShScrambled	Q80UG5	Q80UG5	Sept9	53860	-0.127654936	0.39077277	0.58350919
ShSirt1_vs_ShScrambled	Q6PCP5	Q6PCP5	Mff	75734	-0.395399525	0.3911816	0.5839265
ShSirt1_vs_ShScrambled	Q3U0V2	Q3U0V2	Tradd	71609	0.496772227	0.39168843	0.58448977
ShSirt1_vs_ShScrambled	P50396	P50396	Gdi1	14567	0.150114673	0.39194467	0.58467887
ShSirt1_vs_ShScrambled	Q6PGC7	Q6PGC7	Slc35e3	215436	-0.360738075	0.39226546	0.58481074
ShSirt1_vs_ShScrambled	Q9EP53	Q9EP53	Tsc1	64930	0.268377465	0.39238787	0.58481074
ShSirt1_vs_ShScrambled	Q9Z0H4	Q9Z0H4	Celf2	14007	-0.301385192	0.39242174	0.58481074
ShSirt1_vs_ShScrambled	Q99JB2	Q99JB2	Stoml2	66592	0.13106209	0.3933323	0.58586233
ShSirt1_vs_ShScrambled	Q9QYA2	Q9QYA2	Tomm40	53333	0.149743323	0.39343028	0.58586233
ShSirt1_vs_ShScrambled	Q9R1S3	Q9R1S3	Pign		-0.299177506	0.39362797	0.58586233
ShSirt1_vs_ShScrambled	Q9CZY3	Q9CZY3	Ube2v1	66589	-0.186304632	0.39381363	0.58586233
ShSirt1_vs_ShScrambled	Q91VU8	Q91VU8	Ptov1	84113	0.32997205	0.39381641	0.58586233
ShSirt1_vs_ShScrambled	Q9EPK6	Q9EPK6	Sil1	81500	0.165775298	0.39390611	0.58586233
ShSirt1_vs_ShScrambled	Q80VP0	Q80VP0	Tecpr1	70381	-0.264726068	0.39428376	0.58623086
ShSirt1_vs_ShScrambled	Q80YQ2	Q80YQ2	Med23	70208	-0.358677333	0.39447284	0.58631886
ShSirt1_vs_ShScrambled	Q9D8B3	Q9D8B3	Chmp4b	75608	0.204475453	0.39559692	0.58779609
ShSirt1_vs_ShScrambled	Q8BJ03	Q8BJ03	Cox15	226139	0.1751366	0.39615377	0.58836156
ShSirt1_vs_ShScrambled	O88696	O88696	Clpp	53895	0.215658839	0.39623818	0.58836156
ShSirt1_vs_ShScrambled	P70193	P70193	Lrig1	16206	-0.168729441	0.39679388	0.58899296
ShSirt1_vs_ShScrambled	Q9EP71	Q9EP71	Rai14	75646	-0.159844716	0.39714487	0.58914896
ShSirt1_vs_ShScrambled	Q60875	Q60875	Arhgef2	16800	0.207069157	0.39716001	0.58914896
ShSirt1_vs_ShScrambled	P62082	P62082	Rps7	20115	-0.163000322	0.39852342	0.59081877
ShSirt1_vs_ShScrambled	Q3ULJ0	Q3ULJ0	Gpd1l	333433	-0.396684806	0.39854744	0.59081877
ShSirt1_vs_ShScrambled	P56564	P56564	Slc1a3	20512	-0.354778761	0.39868691	0.59083149
ShSirt1_vs_ShScrambled	P28659	P28659	Celf1	13046	-0.428663103	0.39984606	0.59234327
ShSirt1_vs_ShScrambled	Q07813	Q07813	Bax	12028	0.126509896	0.39996949	0.59234327
ShSirt1_vs_ShScrambled	Q9CWZ7	Q9CWZ7	Napg	108123	-0.213452028	0.40039509	0.59277909
ShSirt1_vs_ShScrambled	Q9CWD8	Q9CWD8	Nubpl	76826	-0.177088454	0.40106085	0.59347216
ShSirt1_vs_ShScrambled	A2AR02	A2AR02	Ppig	228005	0.158336654	0.40115628	0.59347216
ShSirt1_vs_ShScrambled	O35350	O35350	Capn1	12333	-0.125295337	0.40125765	0.59347216
ShSirt1_vs_ShScrambled	P85094	P85094	Isoc2a	664994	-0.169645275	0.40158731	0.59376518
ShSirt1_vs_ShScrambled	Q8CJ26	Q8CJ26	Nradd	67169	-0.351809448	0.40193019	0.59407756
ShSirt1_vs_ShScrambled	Q5SSH7	Q5SSH7	Zzef1	195018	0.420572323	0.40268534	0.5948827
ShSirt1_vs_ShScrambled	Q6ZPJ3	Q6ZPJ3	Ube2o	217342	-0.249927103	0.40273849	0.5948827
ShSirt1_vs_ShScrambled	Q8BR92	Q8BR92	Palm2	677884	0.24066939	0.40303482	0.59512567
ShSirt1_vs_ShScrambled	Q8BKY8	Q8BKY8	Mterf2	74238	-0.42051333	0.40328911	0.59519018
ShSirt1_vs_ShScrambled	Q8C547	Q8C547	Heatr5b	320473	0.314539287	0.40334222	0.59519018
ShSirt1_vs_ShScrambled	Q9QYB1	Q9QYB1	Clic4	29876	-0.16920535	0.40388415	0.59535832
ShSirt1_vs_ShScrambled	Q8R050	Q8R050;Q14	Gspt1		0.143495589	0.4039092	0.59535832
ShSirt1_vs_ShScrambled	Q3TLH4	Q3TLH4	Prrc2c	226562	0.191540214	0.40391799	0.59535832
ShSirt1_vs_ShScrambled	Q68FH4	Q68FH4	Galk2	69976	-0.20701451	0.40398373	0.59535832
ShSirt1_vs_ShScrambled	Q2VPU4	Q2VPU4	Mlxip	208104	-0.26984995	0.4042141	0.59550342
ShSirt1_vs_ShScrambled	Q9CZP7	Q9CZP7	Cdc37l1	67072	-0.157257721	0.40514269	0.5966767
ShSirt1_vs_ShScrambled	Q3UMB5	Q3UMB5	Smcr8	237782	0.169622529	0.40550447	0.59695254
ShSirt1_vs_ShScrambled	P26883	P26883	Fkbp1a	14225	-0.142941055	0.40559447	0.59695254
ShSirt1_vs_ShScrambled	Q78IK2	Q78IK2	Usmg5	66477	-0.265032139	0.40588607	0.59697537
ShSirt1_vs_ShScrambled	O35226	O35226	Psmd4	19185	-0.1455619	0.40591921	0.59697537
ShSirt1_vs_ShScrambled	Q06335	Q06335	Aplp2	11804	-0.292743179	0.40600673	0.59697537
ShSirt1_vs_ShScrambled	Q8R4C2	Q8R4C2	Rufy2	70432	-1.301408252	0.40626823	0.59716535
ShSirt1_vs_ShScrambled	Q9JHW4	Q9JHW4	Eefsec	65967	0.318793145	0.4064023	0.59716796
ShSirt1_vs_ShScrambled	Q921T2	Q921T2	Tor1aip1	208263	-0.244281709	0.40695136	0.59766677
ShSirt1_vs_ShScrambled	Q9WUB4	Q9WUB4	Dctn6	22428	0.473062702	0.40700657	0.59766677
ShSirt1_vs_ShScrambled	Q61263	Q61263	Soat1	20652	-0.357161413	0.40722609	0.59775429
ShSirt1_vs_ShScrambled	P61082	P61082	Ube2m	22192	-0.403627735	0.407337	0.59775429
ShSirt1_vs_ShScrambled	Q9CZ04	Q9CZ04	Cops7a	26894	0.209923537	0.40746343	0.59775429
ShSirt1_vs_ShScrambled	Q8K4M5	Q8K4M5	Commd1	17846	0.697998823	0.40781505	0.59807574
ShSirt1_vs_ShScrambled	Q71FD5	Q71FD5	Znrf2	387524	-0.210681147	0.40827341	0.59855349
ShSirt1_vs_ShScrambled	Q9WU28	Q9WU28	Pfdn5	56612	-0.331435662	0.40888744	0.59918062

ShSirt1_vs_ShScrambled	Q8R1U1	Q8R1U1	Cog4	102339	0.271270333	0.40902432	0.59918062
ShSirt1_vs_ShScrambled	P09405	P09405	Ncl	17975	0.119513951	0.4090994	0.59918062
ShSirt1_vs_ShScrambled	O88712	O88712	Ctbp1	13016	0.133516046	0.4093125	0.59927206
ShSirt1_vs_ShScrambled	Q9EQI8	Q9EQI8	Mrpl46	67308	0.174278804	0.40942735	0.59927206
ShSirt1_vs_ShScrambled	Q6NSR8	Q6NSR8	Npepl1	228961	0.156574048	0.4096578	0.59935877
ShSirt1_vs_ShScrambled	Q8BML9	Q8BML9	Qars	97541	-0.116654749	0.40978678	0.59935877
ShSirt1_vs_ShScrambled	E9Q6J5	E9Q6J5	Bod1l	665775	0.343903353	0.40988492	0.59935877
ShSirt1_vs_ShScrambled	Q6ZQ88	Q6ZQ88	Kdm1a	99982	0.245551966	0.41019854	0.5995827
ShSirt1_vs_ShScrambled	Q9R059	Q9R059	Fhl3	14201	-0.502729746	0.41039524	0.5995827
ShSirt1_vs_ShScrambled	Q9QXE7	Q9QXE7	Tbl1x	21372	-0.102279528	0.41043654	0.5995827
ShSirt1_vs_ShScrambled	O54931	O54931	Akap2	677884	0.207024215	0.41085843	0.59970271
ShSirt1_vs_ShScrambled	P22682	P22682	Cbl	12402	-0.521267469	0.4109119	0.59970271
ShSirt1_vs_ShScrambled	Q9DBT5	Q9DBT5	Ampd2	109674	0.244670122	0.41091726	0.59970271
ShSirt1_vs_ShScrambled	O08532	O08532	Cacna2d1	12293	0.586020966	0.41116213	0.59986615
ShSirt1_vs_ShScrambled	Q6ZQI3	Q6ZQI3	Mlec	109154	0.311778556	0.41173597	0.60032742
ShSirt1_vs_ShScrambled	Q6DFW4	Q6DFW4	Nop58	55989	0.201886362	0.41174428	0.60032742
ShSirt1_vs_ShScrambled	Q9JJI8	Q9JJI8	Rpl38	67671	-0.22048026	0.4122422	0.60085931
ShSirt1_vs_ShScrambled	Q9JJ80	Q9JJ80	Rpf2	67239	0.134345951	0.41265796	0.60093176
ShSirt1_vs_ShScrambled	P83093	P83093	Stim2	116873	-0.262220134	0.41267294	0.60093176
ShSirt1_vs_ShScrambled	Q80U58	Q80U58	Pum2	80913	0.361026753	0.41269128	0.60093176
ShSirt1_vs_ShScrambled	P60710	P60710;P632	Actb		-0.114530759	0.41300179	0.60104285
ShSirt1_vs_ShScrambled	Q6KAR6	Q6KAR6	Exoc3	211446	0.289571861	0.41303387	0.60104285
ShSirt1_vs_ShScrambled	Q6ZPR5	Q6ZPR5	Smpd4	77626	0.284764043	0.41329561	0.6012299
ShSirt1_vs_ShScrambled	Q9D8S4	Q9D8S4	Rexo2	104444	-0.237645532	0.41352758	0.60123885
ShSirt1_vs_ShScrambled	Q69ZQ1	Q69ZQ1	Kiaa1161	329828	-0.710560317	0.41356815	0.60123885
ShSirt1_vs_ShScrambled	O08756	O08756	Hsd17b10		0.115221314	0.41434303	0.60178708
ShSirt1_vs_ShScrambled	Q4VBE8	Q4VBE8	Wdr18	216156	-0.204288913	0.41442834	0.60178708
ShSirt1_vs_ShScrambled	Q80YR4	Q80YR4	Znf598	213753	0.202827981	0.41453161	0.60178708
ShSirt1_vs_ShScrambled	Q9CQE3	Q9CQE3	Mrps17	66258	-0.504524964	0.4146049	0.60178708
ShSirt1_vs_ShScrambled	Q921M4	Q921M4	Golga2	99412	0.136746034	0.41461183	0.60178708
ShSirt1_vs_ShScrambled	Q8R123	Q8R123	Flad1	319945	0.231252752	0.41522305	0.6024805
ShSirt1_vs_ShScrambled	P54823	P54823	Ddx6	13209	-0.111836378	0.41553341	0.60257281
ShSirt1_vs_ShScrambled	Q9QUR6	Q9QUR6	Prep	19072	0.112653725	0.4156047	0.60257281
ShSirt1_vs_ShScrambled	Q8BK63	Q8BK63	Csnk1a1		0.199858852	0.41570923	0.60257281
ShSirt1_vs_ShScrambled	Q8VCN9	Q8VCN9	Tbcc	72726	0.283225675	0.41593061	0.60257281
ShSirt1_vs_ShScrambled	Q60634	Q60634	Flot2	14252	0.219337959	0.41595412	0.60257281
ShSirt1_vs_ShScrambled	Q8VD66	Q8VD66	Abhd4	105501	-0.52578604	0.41621145	0.6025932
ShSirt1_vs_ShScrambled	Q9DCD0	Q9DCD0	Pgd	110208	-0.144658265	0.41631961	0.6025932
ShSirt1_vs_ShScrambled	P35505	P35505	Fah	14085	-0.111202112	0.41636868	0.6025932
ShSirt1_vs_ShScrambled	O54754	O54754	Aox1	11761	1.10979643	0.41656464	0.60268358
ShSirt1_vs_ShScrambled	P97412	P97412	Lyst	17101	0.195119891	0.4177224	0.60400161
ShSirt1_vs_ShScrambled	Q9D7P6	Q9D7P6	Iscu	66383	-0.230019057	0.41774325	0.60400161
ShSirt1_vs_ShScrambled	Q922J3	Q922J3	Clip1	56430	0.201046401	0.41834663	0.60448758
ShSirt1_vs_ShScrambled	O88384	O88384	Vti1b		0.218404137	0.41834719	0.60448758
ShSirt1_vs_ShScrambled	Q9WVJ3	Q9WVJ3	Cpq	54381	-0.148606905	0.41863194	0.60470546
ShSirt1_vs_ShScrambled	O88738	O88738	Birc6	12211	-0.127684563	0.41889548	0.60479683
ShSirt1_vs_ShScrambled	Q64152	Q64152	Btf3	218490	0.113106076	0.41906945	0.60479683
ShSirt1_vs_ShScrambled	P62737	P62737;P632	Acta2		-0.336521297	0.41909714	0.60479683
ShSirt1_vs_ShScrambled	Q9ESJ0	Q9ESJ0	Xpo4	57258	-0.340334196	0.41961078	0.60512563
ShSirt1_vs_ShScrambled	P68369	P68369;P052	Tuba1a		-0.367991704	0.41964633	0.60512563
ShSirt1_vs_ShScrambled	Q8CGY8	Q8CGY8	Ogt	108155	0.164543196	0.41976095	0.60512563
ShSirt1_vs_ShScrambled	P70665	P70665	Siae	22619	-0.311346798	0.4198612	0.60512563
ShSirt1_vs_ShScrambled	Q3TEI4	Q3TEI4	Q3TEI4	74211	-0.335581079	0.42017884	0.60539013
ShSirt1_vs_ShScrambled	Q8C2E7	Q8C2E7	Kiaa0196	223593	0.127281514	0.4213299	0.60685487
ShSirt1_vs_ShScrambled	Q9QYE6	Q9QYE6	Golga5	27277	0.150184541	0.42148099	0.60687884
ShSirt1_vs_ShScrambled	Q9JIG7	Q9JIG7	Ccdc22	54638	0.115251409	0.42174768	0.6070692
ShSirt1_vs_ShScrambled	Q9DD20	Q9DD20	Mettl7b	71664	-0.35523568	0.42196553	0.60718916
ShSirt1_vs_ShScrambled	Q8BTZ7	Q8BTZ7	Gmppb	331026	0.267947755	0.4222852	0.60745551
ShSirt1_vs_ShScrambled	Q8BGS2	Q8BGS2	Bola2	66162	-1.068315938	0.42277363	0.60782666
ShSirt1_vs_ShScrambled	P25976	P25976	Ubtf		-0.210908227	0.42281252	0.60782666
ShSirt1_vs_ShScrambled	Q8BWJ3	Q8BWJ3	Phka2	110094	-0.316911018	0.42319775	0.60807829
ShSirt1_vs_ShScrambled	P35922	P35922	Fmr1		-0.131348003	0.423321	0.60807829
ShSirt1_vs_ShScrambled	Q9CR64	Q9CR64	Tmem167a	66074	-0.212779072	0.42349055	0.60807829

ShSirt1_vs_ShScrambled	Q7TQE6	Q7TQE6	Tmem57	66146	-0.332564475	0.42366994	0.60807829
ShSirt1_vs_ShScrambled	Q9CYI4	Q9CYI4	Luc7l	66978	0.127558129	0.42368661	0.60807829
ShSirt1_vs_ShScrambled	O09110	O09110	Map2k3	26397	0.186993342	0.42379582	0.60807829
ShSirt1_vs_ShScrambled	Q9D8X2	Q9D8X2	Ccdc124	234388	0.422038563	0.42491235	0.60935631
ShSirt1_vs_ShScrambled	O35379	O35379	Abcc1	17250	-0.239801049	0.42495651	0.60935631
ShSirt1_vs_ShScrambled	Q9DCZ4	Q9DCZ4	Apoo	68316	-0.383607979	0.42626014	0.61103153
ShSirt1_vs_ShScrambled	Q8BGE6	Q8BGE6	Atg4b	66615	-0.19191328	0.42659773	0.61126372
ShSirt1_vs_ShScrambled	Q8BJ71	Q8BJ71	Nup93	71805	0.14307651	0.42672021	0.61126372
ShSirt1_vs_ShScrambled	Q8BIG7	Q8BIG7	Comtd1	69156	0.149851345	0.42682837	0.61126372
ShSirt1_vs_ShScrambled	P63330	P63330	Ppp2ca	19052	0.201363206	0.42720551	0.61157288
ShSirt1_vs_ShScrambled	Q99PL5	Q99PL5	Rrbp1	81910	-0.121599698	0.42731521	0.61157288
ShSirt1_vs_ShScrambled	P48441	P48441	Idua		-0.122559009	0.42839667	0.61283342
ShSirt1_vs_ShScrambled	P23591	P23591	Tsta3	22122	0.163853643	0.4284675	0.61283342
ShSirt1_vs_ShScrambled	Q6ZPE2	Q6ZPE2	Sbf1	77980	-0.117783185	0.42877419	0.61307783
ShSirt1_vs_ShScrambled	P53810	P53810	Pitpna	18738	0.134049332	0.42940925	0.61379144
ShSirt1_vs_ShScrambled	Q8CGB6	Q8CGB6	Tns2	209039	0.227797183	0.42966018	0.61395569
ShSirt1_vs_ShScrambled	Q8VCN5	Q8VCN5	Cth	107869	-0.159488582	0.42999477	0.61423936
ShSirt1_vs_ShScrambled	Q9JHU9	Q9JHU9	Isyna1	71780	0.354305805	0.43032411	0.61451535
ShSirt1_vs_ShScrambled	Q9JKW0	Q9JKW0	Arl6ip1	54208	-0.43973704	0.43122309	0.61560437
ShSirt1_vs_ShScrambled	Q9JLN9	Q9JLN9	Mtor	56717	-0.162298589	0.43147401	0.61576784
ShSirt1_vs_ShScrambled	Q6PDN3	Q6PDN3	Mylk		-0.67282938	0.43185505	0.6161066
ShSirt1_vs_ShScrambled	Q2KN98	Q2KN98	Specc1l	74392	-0.586353448	0.43221097	0.6161066
ShSirt1_vs_ShScrambled	Q91VJ5	Q91VJ5	Pqbp1	54633	0.21562314	0.43229694	0.6161066
ShSirt1_vs_ShScrambled	Q9QUJ7	Q9QUJ7	Acsl4	50790	0.152438487	0.43235305	0.6161066
ShSirt1_vs_ShScrambled	P63011	P63011	Rab3a	19339	-0.318435854	0.43251437	0.6161066
ShSirt1_vs_ShScrambled	B2RY04	B2RY04	Dock5	68813	-0.398903878	0.43265368	0.6161066
ShSirt1_vs_ShScrambled	Q04692	Q04692	Smarcad1	13990	0.187867978	0.4326668	0.6161066
ShSirt1_vs_ShScrambled	Q9DCB8	Q9DCB8	Isca2	74316	0.283340424	0.43297707	0.61628731
ShSirt1_vs_ShScrambled	Q9D287	Q9D287	Bcas2	68183	0.244514071	0.43306676	0.61628731
ShSirt1_vs_ShScrambled	Q6P5C5	Q6P5C5	Smug1	71726	0.143690796	0.43342469	0.61660229
ShSirt1_vs_ShScrambled	P82343	P82343	Renbp	19703	-0.360405444	0.43391043	0.61709882
ShSirt1_vs_ShScrambled	Q9D0L4	Q9D0L4	Adck1	72113	-0.445194427	0.43479747	0.6180046
ShSirt1_vs_ShScrambled	Q9JJX6	Q9JJX6	P2rx4		-0.158034499	0.43495005	0.6180046
ShSirt1_vs_ShScrambled	O55013	O55013	Trappc3	27096	0.130158261	0.43495805	0.6180046
ShSirt1_vs_ShScrambled	Q8BGS0	Q8BGS0	Mak16	67920	-0.151798703	0.4352651	0.61824627
ShSirt1_vs_ShScrambled	O88796	O88796	Rpp30	54364	0.121320906	0.43555576	0.61846451
ShSirt1_vs_ShScrambled	Q9JKF1	Q9JKF1	Iqgap1	29875	-0.099809497	0.43588073	0.6186315
ShSirt1_vs_ShScrambled	Q05D44	Q05D44	Eif5b	226982	0.115294935	0.43594746	0.6186315
ShSirt1_vs_ShScrambled	Q8CDG3	Q8CDG3	Vcpip1	70675	-0.139512438	0.43629742	0.61871928
ShSirt1_vs_ShScrambled	O54692	O54692	Zw10	26951	-0.640192601	0.43632583	0.61871928
ShSirt1_vs_ShScrambled	O89023	O89023	Tpp1	12751	0.182789111	0.43642051	0.61871928
ShSirt1_vs_ShScrambled	P62983	P62983;P0C	Rps27a		-0.251036896	0.43733333	0.61981873
ShSirt1_vs_ShScrambled	E9Q5K9	E9Q5K9	Ythdc1	231386	0.372835329	0.43772535	0.62017961
ShSirt1_vs_ShScrambled	Q8VE70	Q8VE70	Pdcd10	56426	-0.216542532	0.43816744	0.62061118
ShSirt1_vs_ShScrambled	Q9D2M8	Q9D2M8	Ube2v2	70620	-0.15913515	0.43844763	0.62081324
ShSirt1_vs_ShScrambled	Q9DBC7	Q9DBC7	Prkar1a	19084	-0.172253479	0.43903098	0.62144429
ShSirt1_vs_ShScrambled	Q99J47	Q99J47	Dhrs7b	216820	-0.247884965	0.43928375	0.62160717
ShSirt1_vs_ShScrambled	Q3UPL0	Q3UPL0	Sec31a	69162	-0.106905464	0.43956114	0.62180476
ShSirt1_vs_ShScrambled	Q8BMS9	Q8BMS9	Rassf2	215653	0.290657727	0.44022793	0.6225529
ShSirt1_vs_ShScrambled	Q9CQS4	Q9CQS4	Slc25a46	67453	0.986819511	0.44084579	0.62323141
ShSirt1_vs_ShScrambled	Q9D024	Q9D024	Ccdc47	67163	-0.111407798	0.4411011	0.62339711
ShSirt1_vs_ShScrambled	P60335	P60335	Pcbp1	23983	0.129252925	0.44227839	0.6248653
ShSirt1_vs_ShScrambled	Q62186	Q62186	Ssr4	20832	-0.12294666	0.44250534	0.62499034
ShSirt1_vs_ShScrambled	P26516	P26516	Psmd7	17463	-0.188727193	0.44291567	0.62518987
ShSirt1_vs_ShScrambled	P19253	P19253	Rpl13a	22121	0.145543351	0.44292362	0.62518987
ShSirt1_vs_ShScrambled	Q99MZ7	Q99MZ7	Pecr	111175	0.122350742	0.44348469	0.62578615
ShSirt1_vs_ShScrambled	Q9JHW2	Q9JHW2	Nit2	52633	0.181486956	0.44366502	0.62584497
ShSirt1_vs_ShScrambled	Q9D1P0	Q9D1P0	Mrpl13	68537	0.255765462	0.44419755	0.62640042
ShSirt1_vs_ShScrambled	P97873	P97873	Loxl1	16949	-0.165701455	0.44478111	0.62693069
ShSirt1_vs_ShScrambled	Q9QXE0	Q9QXE0	Hacl1	56794	0.184983638	0.44485135	0.62693069
ShSirt1_vs_ShScrambled	Q9WUM5	Q9WUM5	Suclg1	56451	-0.167998726	0.44525059	0.62729749
ShSirt1_vs_ShScrambled	O70311	O70311	Nmt2	18108	0.174769175	0.44546116	0.62739835
ShSirt1_vs_ShScrambled	P28271	P28271	Aco1	11428	-0.093573568	0.44588283	0.62779635

ShSirt1_vs_ShScrambled	Q8BMG8	Q8BMG8	Slc25a32	69906	0.151739985	0.4461688	0.6280031
ShSirt1_vs_ShScrambled	O35448	O35448	Ppt2	54397	-0.141851784	0.44716158	0.62920429
ShSirt1_vs_ShScrambled	P63073	P63073	Eif4e	13684	-0.111101839	0.44761966	0.62965259
ShSirt1_vs_ShScrambled	Q9DBR7	Q9DBR7	Ppp1r12a	17931	0.14300398	0.44839171	0.63023127
ShSirt1_vs_ShScrambled	Q8VC19	Q8VC19	Alas1	11655	-0.533614556	0.44848029	0.63023127
ShSirt1_vs_ShScrambled	Q9WVA3	Q9WVA3	Bub3	12237	0.157586187	0.44854003	0.63023127
ShSirt1_vs_ShScrambled	Q9JMA1	Q9JMA1	Usp14	59025	-0.101201293	0.44858951	0.63023127
ShSirt1_vs_ShScrambled	O88668	O88668	Creg1	433375	0.728394354	0.44883304	0.63037721
ShSirt1_vs_ShScrambled	Q80YV4	Q80YV4	Pank4	269614	0.342954086	0.45000979	0.63170976
ShSirt1_vs_ShScrambled	Q61656	Q61656	Ddx5		0.09761957	0.45006172	0.63170976
ShSirt1_vs_ShScrambled	Q0VGY8	Q0VGY8	Tanc1	66860	0.32496268	0.45021859	0.63173351
ShSirt1_vs_ShScrambled	Q9CPQ3	Q9CPQ3	Tomm22	223696	-0.112035476	0.45037244	0.63175301
ShSirt1_vs_ShScrambled	Q9WV84	Q9WV84	Nme4	56520	0.237726577	0.45072762	0.63205483
ShSirt1_vs_ShScrambled	Q9D0L8	Q9D0L8	Rnmt	67897	-0.161136069	0.45182793	0.63340102
ShSirt1_vs_ShScrambled	O35954	O35954	Pitpnm1	18739	0.198895116	0.45221494	0.63370356
ShSirt1_vs_ShScrambled	P58158	P58158	B3gat3	72727	-0.235219564	0.45232451	0.63370356
ShSirt1_vs_ShScrambled	Q8VE80	Q8VE80	Thoc3	73666	0.634435067	0.45265054	0.63396356
ShSirt1_vs_ShScrambled	Q8BXZ1	Q8BXZ1	Tmx3	67988	-0.160442195	0.45310292	0.63400639
ShSirt1_vs_ShScrambled	Q8R0Y8	Q8R0Y8	Slc25a42	73095	-0.208716959	0.45312721	0.63400639
ShSirt1_vs_ShScrambled	Q8K019	Q8K019	Bclaf1	72567	-0.159604794	0.45316039	0.63400639
ShSirt1_vs_ShScrambled	Q9CU62	Q9CU62	Smc1a	24061	0.137017813	0.45324294	0.63400639
ShSirt1_vs_ShScrambled	Q8VCB1	Q8VCB1	Ndc1	72787	0.187445156	0.45343644	0.63408057
ShSirt1_vs_ShScrambled	Q9WVQ5	Q9WVQ5	Apip	56369	0.163960705	0.45382435	0.63434756
ShSirt1_vs_ShScrambled	Q8K2Z4	Q8K2Z4	Ncapd2	68298	0.762948757	0.45390843	0.63434756
ShSirt1_vs_ShScrambled	P15806	P15806	Tcf3	21423	-1.727182806	0.45412565	0.63445471
ShSirt1_vs_ShScrambled	P62876	P62876	Polr2l	66491	0.938513085	0.45569823	0.63645476
ShSirt1_vs_ShScrambled	Q3U829	Q3U829	Ap5z1	231855	-0.241003515	0.45596958	0.63663677
ShSirt1_vs_ShScrambled	Q61879	Q61879	Myh10	77579	0.111986167	0.45614433	0.63668382
ShSirt1_vs_ShScrambled	O35639	O35639	Anxa3	11745	-0.214020887	0.45647375	0.63694668
ShSirt1_vs_ShScrambled	P62754	P62754	Rps6	20104; 10	0.266652646	0.45670077	0.63706652
ShSirt1_vs_ShScrambled	Q3SXD3	Q3SXD3	Hddc2	69692	-0.182740727	0.45703107	0.63714704
ShSirt1_vs_ShScrambled	Q61316	Q61316	Hspa4		0.100991963	0.45711429	0.63714704
ShSirt1_vs_ShScrambled	O35166	O35166	Gosr2	56494	-0.485179081	0.45718194	0.63714704
ShSirt1_vs_ShScrambled	P56873	P56873	Sssca1	56390	-0.266462108	0.4579645	0.63795487
ShSirt1_vs_ShScrambled	Q8CGC6	Q8CGC6	Rbm28	68272	0.243066214	0.45805789	0.63795487
ShSirt1_vs_ShScrambled	Q3UHX2	Q3UHX2	Pdap1	231887	0.350343608	0.45818557	0.63795487
ShSirt1_vs_ShScrambled	Q8BTS4	Q8BTS4	Nup54	269113	0.107090954	0.45866204	0.63842136
ShSirt1_vs_ShScrambled	P21300	P21300	Akr1b7	11997	-0.100654405	0.45896276	0.638643
ShSirt1_vs_ShScrambled	Q9DB30	Q9DB30	Phkg2	68961	0.57379613	0.45986138	0.63969623
ShSirt1_vs_ShScrambled	Q9CQK7	Q9CQK7	Rwdd1	66521	0.182552038	0.46036066	0.64005118
ShSirt1_vs_ShScrambled	Q91XI1	Q91XI1	Dus3l	224907	0.305348756	0.46040013	0.64005118
ShSirt1_vs_ShScrambled	Q9WU42	Q9WU42	Ncor2	20602	-0.238097344	0.46075624	0.64034903
ShSirt1_vs_ShScrambled	O35129	O35129	Phb2	12034	0.104975384	0.46102663	0.64048293
ShSirt1_vs_ShScrambled	O35130	O35130	Emg1	14791	1.143324186	0.46113636	0.64048293
ShSirt1_vs_ShScrambled	Q810V0	Q810V0	Mphosph1(	67973	0.148352283	0.46191532	0.64123846
ShSirt1_vs_ShScrambled	Q9D0B6	Q9D0B6	Pbdc1	67683	0.220533148	0.46196444	0.64123846
ShSirt1_vs_ShScrambled	Q6DID3	Q6DID3	Scaf8	106583	-0.340861168	0.46248926	0.6417696
ShSirt1_vs_ShScrambled	Q9JMA2	Q9JMA2	Qtrt1	60507	-0.163266998	0.46284288	0.64206293
ShSirt1_vs_ShScrambled	P62862	P62862	Fau		0.203703305	0.46323519	0.64234339
ShSirt1_vs_ShScrambled	Q6P8X1	Q6P8X1	Snx6	72183	-0.166158638	0.46338228	0.64234339
ShSirt1_vs_ShScrambled	Q04736	Q04736	Yes1	22612	0.257326635	0.46347196	0.64234339
ShSirt1_vs_ShScrambled	Q8VHE0	Q8VHE0	Sec63	140740	-0.122402176	0.46381725	0.64245585
ShSirt1_vs_ShScrambled	Q9CPX6	Q9CPX6	Atg3	67841	-0.12406951	0.46383775	0.64245585
ShSirt1_vs_ShScrambled	P58021	P58021	Tm9sf2	68059	-0.14617349	0.46424632	0.64282451
ShSirt1_vs_ShScrambled	Q8K157	Q8K157	Galm	319625	0.167257117	0.46517239	0.64390929
ShSirt1_vs_ShScrambled	P60843	P60843	Eif4a1	13681	0.094885375	0.46562583	0.64429344
ShSirt1_vs_ShScrambled	Q8K0G5	Q8K0G5	Tssc1	380752	0.186639476	0.46589275	0.64429344
ShSirt1_vs_ShScrambled	Q9JK48	Q9JK48	Sh3glb1	54673	0.110238932	0.46615081	0.64429344
ShSirt1_vs_ShScrambled	Q8C052	Q8C052	Map1s	270058	0.22242563	0.4663716	0.64429344
ShSirt1_vs_ShScrambled	Q91VU0	Q91VU0	Fam3c	27999	0.2309155	0.46639648	0.64429344
ShSirt1_vs_ShScrambled	Q7TT50	Q7TT50	Cdc42bpb	217866	0.101156832	0.46641918	0.64429344
ShSirt1_vs_ShScrambled	Q9D8S3	Q9D8S3	Arfgap3	66251	0.155774985	0.46644904	0.64429344
ShSirt1_vs_ShScrambled	P97351	P97351	Rps3a	20091	0.120542884	0.46722937	0.6451251

ShSirt1_vs_ShScrambled	Q61191	Q61191	Hcfc1	15161	0.107227503	0.46756123	0.6451251
ShSirt1_vs_ShScrambled	Q91VM3	Q91VM3	Wdr45	54636	-0.167075126	0.46762708	0.6451251
ShSirt1_vs_ShScrambled	Q9JLI6	Q9JLI6	Scly	50880	-0.306324467	0.46772349	0.6451251
ShSirt1_vs_ShScrambled	Q9WTK7	Q9WTK7	Stk11	20869	0.989944816	0.46780065	0.6451251
ShSirt1_vs_ShScrambled	Q8C0L0	Q8C0L0	Tmx4	52837	-0.601771643	0.46790863	0.6451251
ShSirt1_vs_ShScrambled	O35239	O35239	Ptpn9	56294	0.171151324	0.46892868	0.64633406
ShSirt1_vs_ShScrambled	Q60866	Q60866	Pter	19212	0.459545076	0.4695567	0.64700212
ShSirt1_vs_ShScrambled	P98083	P98083	Shc1	20416	-0.163872079	0.47034682	0.64780688
ShSirt1_vs_ShScrambled	A3KGB4	A3KGB4	Tbc1d8b	245638	-0.12053505	0.47042777	0.64780688
ShSirt1_vs_ShScrambled	Q6NZC7	Q6NZC7	Sec23ip		-0.162937152	0.4709972	0.64839321
ShSirt1_vs_ShScrambled	P62257	P62257	Ube2h	22214	-0.36024449	0.47142282	0.64878129
ShSirt1_vs_ShScrambled	Q9CQI3	Q9CQI3	Gmfb	63985	0.330854549	0.4721357	0.64941769
ShSirt1_vs_ShScrambled	Q9CPZ8	Q9CPZ8	Cmc1	67899	-0.565189834	0.47217298	0.64941769
ShSirt1_vs_ShScrambled	Q9CQJ4	Q9CQJ4	Rnf2	19821	0.373708572	0.47239943	0.64953123
ShSirt1_vs_ShScrambled	Q01147	Q01147	Creb1	12912	-0.296194868	0.47330842	0.65058289
ShSirt1_vs_ShScrambled	Q99JW2	Q99JW2	Acy1	109652	-0.13290396	0.47356202	0.65073332
ShSirt1_vs_ShScrambled	O08746	O08746	Matn2		0.302749191	0.47419426	0.65134792
ShSirt1_vs_ShScrambled	Q9CY57	Q9CY57	Chtop	66511	0.132489309	0.4743302	0.65134792
ShSirt1_vs_ShScrambled	Q99L04	Q99L04	Dhrs1	52585	0.178156438	0.47444217	0.65134792
ShSirt1_vs_ShScrambled	Q9D1Q6	Q9D1Q6	Erp44	76299	-0.143890781	0.47609355	0.65341632
ShSirt1_vs_ShScrambled	Q8BGH2	Q8BGH2	Samm50	68653	-0.113479813	0.47634005	0.65355593
ShSirt1_vs_ShScrambled	P97470	P97470	Ppp4c	56420	0.152903687	0.47650523	0.6535839
ShSirt1_vs_ShScrambled	O88441	O88441	Mtx2	53375	-0.109422969	0.47702613	0.65409962
ShSirt1_vs_ShScrambled	Q9D2E2	Q9D2E2	Toe1	68276	0.389982406	0.47760475	0.65469415
ShSirt1_vs_ShScrambled	Q8CCB4	Q8CCB4	Vps53	68299	-0.124132304	0.47794385	0.65496009
ShSirt1_vs_ShScrambled	P62897	P62897	Cycs	13063	-0.187559818	0.47830903	0.6552616
ShSirt1_vs_ShScrambled	Q9QUM9	Q9QUM9	Psma6	26443	-0.152807601	0.47848404	0.65530248
ShSirt1_vs_ShScrambled	Q8VDC0	Q8VDC0	Lars2	102436	-0.216818084	0.47945161	0.65642844
ShSirt1_vs_ShScrambled	Q9Z0R4	Q9Z0R4	Itsn1	16443	0.145487422	0.4798608	0.65678946
ShSirt1_vs_ShScrambled	Q3UPH1	Q3UPH1	Prrc1	73137	0.168027452	0.48100369	0.65815418
ShSirt1_vs_ShScrambled	Q99JY4	Q99JY4	Trabd	67976	0.45086025	0.48129309	0.65822166
ShSirt1_vs_ShScrambled	Q01320	Q01320	Top2a	21973	-0.58197721	0.48134464	0.65822166
ShSirt1_vs_ShScrambled	Q7TMC8	Q7TMC8	Fuk	234730	0.2606611	0.48264555	0.65980074
ShSirt1_vs_ShScrambled	Q61581	Q61581	Igfbp7	29817	0.178576532	0.48293036	0.6599902
ShSirt1_vs_ShScrambled	O08788	O08788	Dctn1	13191	-0.105780077	0.48405107	0.66118031
ShSirt1_vs_ShScrambled	Q3UHJ0	Q3UHJ0	Aak1	269774	-0.147768758	0.48409413	0.66118031
ShSirt1_vs_ShScrambled	Q3TBW2	Q3TBW2	Mrpl10	107732	-0.204941425	0.48458318	0.66152533
ShSirt1_vs_ShScrambled	Q7TN22	Q7TN22	Txndc16	70561	0.556294285	0.48463985	0.66152533
ShSirt1_vs_ShScrambled	P05064	P05064	Aldoa	11674	0.093879132	0.48512115	0.66198213
ShSirt1_vs_ShScrambled	P48678	P48678	Lmna	16905	-0.094566534	0.48602487	0.66301489
ShSirt1_vs_ShScrambled	Q6P4S6	Q6P4S6	Sik3	70661	-0.221076726	0.48696475	0.66391103
ShSirt1_vs_ShScrambled	Q99M87	Q99M87	Dnaja3	83945	0.134224779	0.48697595	0.66391103
ShSirt1_vs_ShScrambled	Q8R5A6	Q8R5A6	Tbc1d22a	223754	0.752634415	0.48859325	0.6657887
ShSirt1_vs_ShScrambled	Q99KN9	Q99KN9	Clint1		0.130139318	0.4886482	0.6657887
ShSirt1_vs_ShScrambled	O54879	O54879	Hmgb3	15354	0.220967748	0.48889718	0.66592694
ShSirt1_vs_ShScrambled	Q9Z1X4	Q9Z1X4	Ilf3	16201	0.116166086	0.4893425	0.66618163
ShSirt1_vs_ShScrambled	Q8BIW1	Q8BIW1	Prune	229589	0.174124449	0.48937932	0.66618163
ShSirt1_vs_ShScrambled	P17742	P17742	Ppia	268373	0.152042315	0.49039659	0.66736515
ShSirt1_vs_ShScrambled	E9Q555	E9Q555	Rnf213		0.102354567	0.49126898	0.66835087
ShSirt1_vs_ShScrambled	Q9D0M1	Q9D0M1	Prpsap1	67763	0.093753076	0.49196146	0.66902548
ShSirt1_vs_ShScrambled	Q0VGB7	Q0VGB7	Ppp4r2	232314	0.219752881	0.49206127	0.66902548
ShSirt1_vs_ShScrambled	Q9DBM0	Q9DBM0	Abcg8	67470	0.251363894	0.49229843	0.66914638
ShSirt1_vs_ShScrambled	Q8C460	Q8C460	Eri3	140546	-0.255962699	0.49302231	0.66992857
ShSirt1_vs_ShScrambled	P40124	P40124	Cap1	12331	0.124343693	0.49378752	0.67076644
ShSirt1_vs_ShScrambled	Q9DAR7	Q9DAR7	Dcps	69305	0.125417361	0.49429328	0.67125146
ShSirt1_vs_ShScrambled	Q8VI33	Q8VI33	Taf9	108143	0.26706978	0.49484881	0.67137427
ShSirt1_vs_ShScrambled	A2A5R2	A2A5R2	Arfgef2	99371	0.123424008	0.49490005	0.67137427
ShSirt1_vs_ShScrambled	Q91V64	Q91V64	Isoc1	66307	-0.115327229	0.49492075	0.67137427
ShSirt1_vs_ShScrambled	P51569	P51569	Gla		-0.264510825	0.49497864	0.67137427
ShSirt1_vs_ShScrambled	Q9DBC3	Q9DBC3	Cmtr1	74157	-0.17570108	0.4957656	0.67200096
ShSirt1_vs_ShScrambled	Q8K3C3	Q8K3C3	Lzic	69151	-0.160088148	0.49603609	0.67200096
ShSirt1_vs_ShScrambled	Q9DBY1	Q9DBY1	Syvn1	74126	0.145662546	0.49604811	0.67200096
ShSirt1_vs_ShScrambled	Q91V41	Q91V41	Rab14	68365	-0.12621362	0.49608567	0.67200096

ShSirt1_vs_ShScrambled	Q9DCF9	Q9DCF9	Ssr3	67437	0.156891943	0.49618502	0.67200096
ShSirt1_vs_ShScrambled	P52479	P52479	Usp10	22224	0.264342573	0.49659096	0.67234901
ShSirt1_vs_ShScrambled	Q811S7	Q811S7	Ubp1	22221	0.09561692	0.49683576	0.67247875
ShSirt1_vs_ShScrambled	P58742	P58742	Aaas	223921	-0.160917151	0.49735186	0.6729755
ShSirt1_vs_ShScrambled	Q8R1I1	Q8R1I1	Uqcr10	66152	0.153075117	0.49758949	0.67309528
ShSirt1_vs_ShScrambled	Q6P1H6	Q6P1H6	Ankle2	71782	-0.109210413	0.49774158	0.67309931
ShSirt1_vs_ShScrambled	Q9ERG0	Q9ERG0	Lima1	65970	-0.152189277	0.4983975	0.67378447
ShSirt1_vs_ShScrambled	Q6PHN9	Q6PHN9	Rab35	77407	0.148519161	0.4986928	0.67391196
ShSirt1_vs_ShScrambled	P35980	P35980	Rpl18	19899	-0.125765395	0.4987904	0.67391196
ShSirt1_vs_ShScrambled	Q03141	Q03141	Mark3	17169	-0.272983648	0.49928533	0.67437881
ShSirt1_vs_ShScrambled	Q9D0B0	Q9D0B0	Srsf9	108014	0.122821418	0.49954544	0.6745283
ShSirt1_vs_ShScrambled	P59235	P59235	Nup43	69912	0.260784347	0.50009882	0.67493192
ShSirt1_vs_ShScrambled	Q8BHF7	Q8BHF7	Pgs1	74451	0.151025986	0.50014339	0.67493192
ShSirt1_vs_ShScrambled	P52633	P52633	Stat6	20852	-0.443431364	0.50099211	0.67587519
ShSirt1_vs_ShScrambled	P24668	P24668	M6pr	17113	0.168327264	0.50184173	0.67681912
ShSirt1_vs_ShScrambled	Q921W0	Q921W0	Chmp1a	234852	-0.213806766	0.50230762	0.6772451
ShSirt1_vs_ShScrambled	P32233	P32233	Drg1	13494	0.2014775	0.50262926	0.67747641
ShSirt1_vs_ShScrambled	Q61189	Q61189	Clns1a		0.340694391	0.50328606	0.67784053
ShSirt1_vs_ShScrambled	Q91XE4	Q91XE4	Acy3	71670	0.110160972	0.5034724	0.67784053
ShSirt1_vs_ShScrambled	Q99M04	Q99M04	Lias	79464	-0.189129992	0.50361436	0.67784053
ShSirt1_vs_ShScrambled	Q9R190	Q9R190	Mta2	23942	0.115992123	0.50379645	0.67784053
ShSirt1_vs_ShScrambled	Q9DB05	Q9DB05	Napa	108124	-0.110342036	0.50379939	0.67784053
ShSirt1_vs_ShScrambled	E9PVA8	E9PVA8	Gcn1l1	231659	0.101694229	0.50391374	0.67784053
ShSirt1_vs_ShScrambled	P56380	P56380	Nudt2	66401	-0.143683892	0.50398439	0.67784053
ShSirt1_vs_ShScrambled	O09106	O09106	Hdac1	433759	0.16181982	0.50410072	0.67784053
ShSirt1_vs_ShScrambled	Q3UGP8	Q3UGP8	Alg10b	380959	0.226154444	0.50456127	0.67825776
ShSirt1_vs_ShScrambled	Q9CZW5	Q9CZW5	Tomm70a	28185	0.096503257	0.50483088	0.67841815
ShSirt1_vs_ShScrambled	Q8K1A6	Q8K1A6	Cc2d1a	212139	0.306084327	0.50505359	0.67851545
ShSirt1_vs_ShScrambled	G3X9K3	G3X9K3	Arfgef1	211673	-0.248040259	0.50521506	0.67853043
ShSirt1_vs_ShScrambled	P60904	P60904	Dnajc5	13002	-0.11242214	0.50548794	0.67869499
ShSirt1_vs_ShScrambled	P70296	P70296	Pebp1	23980	-0.104869023	0.50619903	0.67933011
ShSirt1_vs_ShScrambled	Q03147	Q03147	Cdk7	12572	-0.606112151	0.50641967	0.67933011
ShSirt1_vs_ShScrambled	Q9JID9	Q9JID9	Sh2b2	23921	-0.270363713	0.50645653	0.67933011
ShSirt1_vs_ShScrambled	Q9CWV0	Q9CWV0	Malsu1	75593	-0.232520871	0.50656295	0.67933011
ShSirt1_vs_ShScrambled	Q80UJ7	Q80UJ7	Rab3gap1	226407	0.104696201	0.50702843	0.6797524
ShSirt1_vs_ShScrambled	P98195	P98195	Atp9b	50771	-0.351264207	0.50725367	0.67985245
ShSirt1_vs_ShScrambled	Q8BMZ5	Q8BMZ5	Tsen34	66078	0.588483847	0.50791713	0.68043406
ShSirt1_vs_ShScrambled	Q9DBL7	Q9DBL7	Coasy	71743	0.101802508	0.5079891	0.68043406
ShSirt1_vs_ShScrambled	Q99LL3	Q99LL3	Chst12	59031	0.220965412	0.50855631	0.6809532
ShSirt1_vs_ShScrambled	Q8VCH5	Q8VCH5	Rabepk	227746	-0.1904129	0.50867838	0.6809532
ShSirt1_vs_ShScrambled	Q99NH0	Q99NH0	Ankrd17	81702	-0.140914641	0.50893065	0.68108893
ShSirt1_vs_ShScrambled	Q8K114	Q8K114	Ints9	210925	0.221149521	0.51003284	0.68216601
ShSirt1_vs_ShScrambled	Q3TWL2	Q3TWL2	Tmem55b	219024	-0.15775825	0.51003772	0.68216601
ShSirt1_vs_ShScrambled	Q8BGW1	Q8BGW1	Fto	26383	0.115015437	0.51062375	0.68274751
ShSirt1_vs_ShScrambled	Q8BVU0	Q8BVU0	Lrch3	70144	-0.130172091	0.51090068	0.6829155
ShSirt1_vs_ShScrambled	Q99N92	Q99N92	Mrpl27	94064	-0.228586327	0.51159893	0.6834732
ShSirt1_vs_ShScrambled	Q5SW19	Q5SW19	Cluh	74148	-0.10425764	0.51162072	0.6834732
ShSirt1_vs_ShScrambled	P53995	P53995	Anapc1	17222	0.146390463	0.51194643	0.68370597
ShSirt1_vs_ShScrambled	P62631	P62631	Eef1a2	13628	-0.840381788	0.5124053	0.68411639
ShSirt1_vs_ShScrambled	B2RXC1	B2RXC1	Trappc11	320714	0.121112418	0.51301019	0.68472146
ShSirt1_vs_ShScrambled	Q924Z4	Q924Z4	Cers2	76893	-0.150778484	0.51326101	0.68485374
ShSirt1_vs_ShScrambled	Q99JI6	Q99JI6	Rap1b	215449	0.282569752	0.51382642	0.6853409
ShSirt1_vs_ShScrambled	P68033	P68033;P681	Actc1		0.280005638	0.51392976	0.6853409
ShSirt1_vs_ShScrambled	Q91ZJ5	Q91ZJ5	Ugp2	216558	-0.094748586	0.51492344	0.68646321
ShSirt1_vs_ShScrambled	P70266	P70266	Pfkfb1	18639	0.33912453	0.51514324	0.68652741
ShSirt1_vs_ShScrambled	P62918	P62918	Rpl8	26961	0.100827958	0.51527578	0.68652741
ShSirt1_vs_ShScrambled	Q9ES56	Q9ES56	Trappc4	60409	-0.105031871	0.51555328	0.68669446
ShSirt1_vs_ShScrambled	Q5SSW2	Q5SSW2	Psme4	103554	-0.1386401	0.51572876	0.68672555
ShSirt1_vs_ShScrambled	Q9CQ69	Q9CQ69	Uqcrq	22272	-0.120095423	0.51593594	0.68679884
ShSirt1_vs_ShScrambled	Q9DCJ1	Q9DCJ1	Mlst8	56716	-0.776209836	0.51654931	0.68741261
ShSirt1_vs_ShScrambled	Q8BGY7	Q8BGY7	Fam210a	108654	-0.101634632	0.51697188	0.68772208
ShSirt1_vs_ShScrambled	Q99LC8	Q99LC8	Eif2b1	209354	0.19244569	0.51708656	0.68772208
ShSirt1_vs_ShScrambled	Q9D8M4	Q9D8M4	Rpl7l1	66229	0.232998948	0.51753438	0.68794351

ShSirt1_vs_ShScrambled	Q3UFF7	Q3UFF7	Lyplal1	226791	0.142190617	0.51755786	0.68794351
ShSirt1_vs_ShScrambled	Q60809	Q60809	Cnot7	18983	-0.259221287	0.51810666	0.6884476
ShSirt1_vs_ShScrambled	Q9DCR2	Q9DCR2	Ap3s1	11777	-0.168238612	0.518292	0.6884476
ShSirt1_vs_ShScrambled	Q80TI0	Q80TI0	Gramd1b	235283	-0.398245799	0.51839463	0.6884476
ShSirt1_vs_ShScrambled	Q9D5V6	Q9D5V6	Syap1	67043	0.132239173	0.51935423	0.68951911
ShSirt1_vs_ShScrambled	P27659	P27659	Rpl3	27367	0.087727033	0.51959855	0.68964065
ShSirt1_vs_ShScrambled	Q99JX4	Q99JX4	Eif3m	98221	0.142576308	0.52017808	0.68970305
ShSirt1_vs_ShScrambled	O35206	O35206	Col15a1	12819	-0.100601646	0.52021035	0.68970305
ShSirt1_vs_ShScrambled	Q8C407	Q8C407	Yipf4	67864	0.258098977	0.52022781	0.68970305
ShSirt1_vs_ShScrambled	P28740	P28740	Kif2a	16563	0.232514892	0.52025673	0.68970305
ShSirt1_vs_ShScrambled	P83882	P83882	Rpl36a	19982; 66	0.271052901	0.52090733	0.69035468
ShSirt1_vs_ShScrambled	Q07802	Q07802;O08	Ebf1		-0.399290186	0.52105414	0.69035468
ShSirt1_vs_ShScrambled	Q9DBB4	Q9DBB4	Naa16	66897	0.199659426	0.52145715	0.69063947
ShSirt1_vs_ShScrambled	Q9D920	Q9D920	Loh12cr1	67774	0.818798552	0.52157509	0.69063947
ShSirt1_vs_ShScrambled	P49138	P49138	Mapkapk2	17164	-0.207923369	0.52192703	0.69090283
ShSirt1_vs_ShScrambled	Q6P5D3	Q6P5D3	Dhx57	106794	0.303325043	0.52236001	0.69095052
ShSirt1_vs_ShScrambled	Q7TT37	Q7TT37	Ikbkap	230233	0.083674268	0.52251235	0.69095052
ShSirt1_vs_ShScrambled	Q8C2Q3	Q8C2Q3	Rbm14	56275	0.091798105	0.52272214	0.69095052
ShSirt1_vs_ShScrambled	Q91WF7	Q91WF7	Fig4	103199	0.616625655	0.52286226	0.69095052
ShSirt1_vs_ShScrambled	Q8R395	Q8R395	Commd5	66398	0.319693986	0.52292314	0.69095052
ShSirt1_vs_ShScrambled	P55302	P55302	Lrpap1	16976	-0.122474427	0.52322218	0.69095052
ShSirt1_vs_ShScrambled	P35235	P35235	Ptpn11	19247	0.115570899	0.52326925	0.69095052
ShSirt1_vs_ShScrambled	Q8VCM4	Q8VCM4	Lipt1	623661	-0.255836267	0.52348508	0.69095052
ShSirt1_vs_ShScrambled	Q8BZW8	Q8BZW8	Nhlrc2	66866	-0.105503487	0.52353396	0.69095052
ShSirt1_vs_ShScrambled	Q3TJD7	Q3TJD7	Pdlim7	67399	-0.36618063	0.52365522	0.69095052
ShSirt1_vs_ShScrambled	Q80V26	Q80V26	Impad1	242291	0.43486791	0.52368021	0.69095052
ShSirt1_vs_ShScrambled	Q920N2	Q920N2	Hlcs	110948	-0.372051258	0.52379988	0.69095052
ShSirt1_vs_ShScrambled	Q4FK66	Q4FK66	Prpf38a	230596	0.647077224	0.52445381	0.69161101
ShSirt1_vs_ShScrambled	Q64449	Q64449	Mrc2	17534	-0.105871404	0.52534665	0.69258609
ShSirt1_vs_ShScrambled	P40338	P40338	Vhl	22346	0.185576797	0.52605007	0.69315447
ShSirt1_vs_ShScrambled	Q9D945	Q9D945	Llph	66225	0.253964175	0.52619479	0.69315447
ShSirt1_vs_ShScrambled	P60867	P60867	Rps20	67427	0.120780546	0.5263455	0.69315447
ShSirt1_vs_ShScrambled	Q3TIR1	Q3TIR1	Trappc13	66975	-0.271247214	0.52639201	0.69315447
ShSirt1_vs_ShScrambled	P13020	P13020	Gsn	227753	-0.076593697	0.52679783	0.69330183
ShSirt1_vs_ShScrambled	Q91W18	Q91W18	Tdrd3	219249	-0.277698047	0.52691843	0.69330183
ShSirt1_vs_ShScrambled	P97822	P97822	Anp32e	66471	0.652137939	0.52696469	0.69330183
ShSirt1_vs_ShScrambled	Q9CWX4	Q9CWX4	Rpusd4	71989	0.253258797	0.52721923	0.69343462
ShSirt1_vs_ShScrambled	P62311	P62311	Lsm3	67678	0.095710855	0.52829449	0.69464646
ShSirt1_vs_ShScrambled	Q8R3U1	Q8R3U1	Pla2g16	225845	-0.394712797	0.52912592	0.6955371
ShSirt1_vs_ShScrambled	Q8CIM8	Q8CIM8	Ints4	101861	0.144238237	0.52968936	0.69607504
ShSirt1_vs_ShScrambled	P70279	P70279	Surf6	20935	0.243591649	0.53015838	0.69648863
ShSirt1_vs_ShScrambled	Q3UJU9	Q3UJU9	Rmdn3	67809	-0.095895477	0.53044079	0.69661607
ShSirt1_vs_ShScrambled	O09044	O09044	Snap23	20619	0.113206849	0.53056404	0.69661607
ShSirt1_vs_ShScrambled	Q69ZJ7	Q69ZJ7	Ric1		0.465639864	0.53095072	0.69692107
ShSirt1_vs_ShScrambled	Q8BP71	Q8BP71;Q9J	Rbfox2		-0.269182867	0.53144	0.69736052
ShSirt1_vs_ShScrambled	Q9ESE1	Q9ESE1	Lrba	80877	-0.202303709	0.53199701	0.69788855
ShSirt1_vs_ShScrambled	Q9CXW2	Q9CXW2	Mrps22	64655	0.096170366	0.53245034	0.69824781
ShSirt1_vs_ShScrambled	Q9JIQ3	Q9JIQ3	Diablo	66593	0.117311495	0.53258024	0.69824781
ShSirt1_vs_ShScrambled	Q6GV12	Q6GV12	Kdsr	70750	-0.22269463	0.53387609	0.69967627
ShSirt1_vs_ShScrambled	Q9JIK5	Q9JIK5	Ddx21	56200	-0.101579466	0.53397979	0.69967627
ShSirt1_vs_ShScrambled	A2ABV5	A2ABV5	Med14	26896	0.166774032	0.53528756	0.70118632
ShSirt1_vs_ShScrambled	P62849	P62849	Rps24	20088	0.097865444	0.53550305	0.70126509
ShSirt1_vs_ShScrambled	O35623	O35623	Bet1	12068	0.319116003	0.53587433	0.70154777
ShSirt1_vs_ShScrambled	Q8BUK6	Q8BUK6	Hook3	320191	-0.111846359	0.53620206	0.70174685
ShSirt1_vs_ShScrambled	Q8C878	Q8C878	Uba3	22200	-0.200249077	0.53633732	0.70174685
ShSirt1_vs_ShScrambled	Q62383	Q62383	Supt6h	20926	-0.318056516	0.53732078	0.70282991
ShSirt1_vs_ShScrambled	Q9DAZ9	Q9DAZ9	Zfyve19	72008	-0.362307564	0.53781316	0.70327016
ShSirt1_vs_ShScrambled	Q8C0Q2	Q8C0Q2	Zhx3	320799	0.802654194	0.53838179	0.70371548
ShSirt1_vs_ShScrambled	Q9JLQ2	Q9JLQ2	Git2	26431	-0.113139114	0.5384655	0.70371548
ShSirt1_vs_ShScrambled	Q9D6Z1	Q9D6Z1	Nop56	67134	0.124587714	0.53908658	0.70432324
ShSirt1_vs_ShScrambled	Q60865	Q60865	Caprin1	53872	-0.090258724	0.53929874	0.70439656
ShSirt1_vs_ShScrambled	P14824	P14824	Anxa6	11749	-0.095729336	0.54005585	0.7051814
ShSirt1_vs_ShScrambled	Q8BTI8	Q8BTI8	Srrm2	75956	0.107847105	0.5402548	0.70523718

ShSirt1_vs_ShScrambled	Q9R0A0	Q9R0A0	Pex14	56273	-0.097158335	0.54041749	0.70524561
ShSirt1_vs_ShScrambled	Q9Z321	Q9Z321	Top3b	21976	0.171388991	0.54140676	0.70617328
ShSirt1_vs_ShScrambled	Q9D844	Q9D844	Dnajc4	57431	-0.137339866	0.54144123	0.70617328
ShSirt1_vs_ShScrambled	Q9JIH2	Q9JIH2	Nup50	18141	-0.305992671	0.5418468	0.70649811
ShSirt1_vs_ShScrambled	Q8C5L3	Q8C5L3	Cnot2	72068	-0.109966267	0.54202536	0.70652684
ShSirt1_vs_ShScrambled	Q9D8C4	Q9D8C4	Ifi35	70110	0.151389934	0.54284148	0.70738639
ShSirt1_vs_ShScrambled	Q99LX0	Q99LX0	Park7	57320	-0.128968981	0.54330894	0.70776766
ShSirt1_vs_ShScrambled	P63325	P63325	Rps10	67097	-0.08620376	0.54362795	0.70776766
ShSirt1_vs_ShScrambled	Q80TP3	Q80TP3	Ubr5	70790	0.14709818	0.54363672	0.70776766
ShSirt1_vs_ShScrambled	P70318	P70318	Tial1	21843	0.113319848	0.54376124	0.70776766
ShSirt1_vs_ShScrambled	Q8BLN5	Q8BLN5	Lss	16987	0.091100226	0.54409941	0.70800367
ShSirt1_vs_ShScrambled	Q8QZS3	Q8QZS3	Flcn	216805	-0.253016551	0.54473866	0.70843112
ShSirt1_vs_ShScrambled	Q9WV32	Q9WV32	Arpc1b	11867	-0.12613354	0.54474178	0.70843112
ShSirt1_vs_ShScrambled	Q61598	Q61598	Gdi2	14569	0.085546384	0.54562338	0.70937325
ShSirt1_vs_ShScrambled	Q9CPS7	Q9CPS7	Pno1	66249	0.274812804	0.54644627	0.70980423
ShSirt1_vs_ShScrambled	Q9QZD9	Q9QZD9	Eif3i	54709	0.096358808	0.54646052	0.70980423
ShSirt1_vs_ShScrambled	Q61771	Q61771	Kif3b	16569	-0.547135478	0.54653565	0.70980423
ShSirt1_vs_ShScrambled	Q61595	Q61595	Ktn1	16709	0.08755217	0.54658385	0.70980423
ShSirt1_vs_ShScrambled	Q8BM72	Q8BM72	Hspa13	110920	-0.39459037	0.54697595	0.70999312
ShSirt1_vs_ShScrambled	O54998	O54998	Fkbp7	14231	-0.15447656	0.54704388	0.70999312
ShSirt1_vs_ShScrambled	Q6PB93	Q6PB93	Galnt2	108148	-0.461108756	0.54720642	0.70999994
ShSirt1_vs_ShScrambled	P50637	P50637	Tspo	12257	0.13620674	0.54760028	0.71015666
ShSirt1_vs_ShScrambled	Q8CJ19	Q8CJ19	Mical3	194401	0.102170758	0.54764186	0.71015666
ShSirt1_vs_ShScrambled	Q9CYX7	Q9CYX7	Rrp15	67223	0.254720205	0.54797455	0.710384
ShSirt1_vs_ShScrambled	Q9WVJ2	Q9WVJ2	Psmd13	23997	-0.073299226	0.54840223	0.71053486
ShSirt1_vs_ShScrambled	Q64010	Q64010	Crk	12928	-0.09408722	0.54863117	0.71053486
ShSirt1_vs_ShScrambled	P09055	P09055	Itgb1	16412	-0.089415469	0.54870769	0.71053486
ShSirt1_vs_ShScrambled	Q9Z2L6	Q9Z2L6	Minpp1	17330	0.150127327	0.54900124	0.71053486
ShSirt1_vs_ShScrambled	Q8BG32	Q8BG32	Psmd11	69077	-0.078309007	0.54900936	0.71053486
ShSirt1_vs_ShScrambled	O89051	O89051	Itm2b	16432	-0.236343077	0.54910357	0.71053486
ShSirt1_vs_ShScrambled	P99027	P99027	Rplp2	67186	-0.089945642	0.54919276	0.71053486
ShSirt1_vs_ShScrambled	Q07417	Q07417	Acads	11409	-0.082250625	0.55015108	0.71157076
ShSirt1_vs_ShScrambled	Q99LD8	Q99LD8	Ddah2	51793	-0.095990577	0.55031454	0.7115783
ShSirt1_vs_ShScrambled	P62874	P62874	Gnb1	14688	-0.276149761	0.55069405	0.71180674
ShSirt1_vs_ShScrambled	P22892	P22892	Ap1g1	11765	0.14886309	0.55080659	0.71180674
ShSirt1_vs_ShScrambled	Q61990	Q61990	Pcbp2	18521	0.073483312	0.55098447	0.71183282
ShSirt1_vs_ShScrambled	Q91YW3	Q91YW3	Dnajc3	1E+08	0.126460444	0.55177516	0.71257042
ShSirt1_vs_ShScrambled	Q9Z0R6	Q9Z0R6	Itsn2		-0.170931111	0.55187111	0.71257042
ShSirt1_vs_ShScrambled	Q9CYR6	Q9CYR6	Pgm3	109785	0.182733679	0.55359994	0.71459826
ShSirt1_vs_ShScrambled	Q8R3R8	Q8R3R8	Gabarapl1	57436	-0.107868374	0.55401846	0.71493406
ShSirt1_vs_ShScrambled	Q9JKC8	Q9JKC8	Ap3m1	55946	0.071975887	0.55454445	0.71528542
ShSirt1_vs_ShScrambled	Q9Z2U1	Q9Z2U1	Psma5	26442	0.077896353	0.55460766	0.71528542
ShSirt1_vs_ShScrambled	Q6P9R4	Q6P9R4	Arhgef18	102098	0.231180346	0.55481488	0.71534829
ShSirt1_vs_ShScrambled	Q9R210	Q9R210	Tfeb	21425	-0.188001689	0.55510425	0.71551702
ShSirt1_vs_ShScrambled	Q6A0A2	Q6A0A2	Larp4b	217980	0.227595394	0.55604512	0.71619828
ShSirt1_vs_ShScrambled	P39061	P39061	Col18a1	12822	-0.168181831	0.55610871	0.71619828
ShSirt1_vs_ShScrambled	O88856	O88856	Tpst2	22022	0.186871899	0.55625447	0.71619828
ShSirt1_vs_ShScrambled	Q9CW03	Q9CW03	Smc3	13006	0.115182261	0.55636804	0.71619828
ShSirt1_vs_ShScrambled	O08605	O08605	Mknk1	17346	-0.548324686	0.55642609	0.71619828
ShSirt1_vs_ShScrambled	Q922D8	Q922D8	Mthfd1	108156	0.072373007	0.55672355	0.71637688
ShSirt1_vs_ShScrambled	Q80Y81	Q80Y81	Elac2	68626	0.127034386	0.55732487	0.71681856
ShSirt1_vs_ShScrambled	P97376	P97376	Frg1	14300	-0.16173421	0.55758213	0.71681856
ShSirt1_vs_ShScrambled	Q8K183	Q8K183	Pdxk	216134	0.107250349	0.55761878	0.71681856
ShSirt1_vs_ShScrambled	Q9CQ10	Q9CQ10	Chmp3	66700	-0.35316162	0.55771091	0.71681856
ShSirt1_vs_ShScrambled	Q07076	Q07076	Anxa7	11750	-0.111758276	0.55786079	0.71681856
ShSirt1_vs_ShScrambled	Q8BL97	Q8BL97	Srsf7	225027	0.118415634	0.55825361	0.71711918
ShSirt1_vs_ShScrambled	Q9D136	Q9D136	Ogfod3	66179	0.329020876	0.55846584	0.7171877
ShSirt1_vs_ShScrambled	Q8C1Y8	Q8C1Y8	Ccz1	231874	-0.234896349	0.55905101	0.717735
ShSirt1_vs_ShScrambled	Q9CQF8	Q9CQF8	Mrpl57	67840	-0.280080519	0.55997941	0.71872251
ShSirt1_vs_ShScrambled	Q6NXI6	Q6NXI6	Rprd2	75137	0.167702222	0.56069408	0.71943521
ShSirt1_vs_ShScrambled	Q62095	Q62095	Ddx3y	26900	0.230685736	0.56100739	0.71963267
ShSirt1_vs_ShScrambled	P11688	P11688	Itga5	16402	-0.14283458	0.56208698	0.72081268
ShSirt1_vs_ShScrambled	P84099	P84099	Rpl19	19921	-0.097235143	0.56317464	0.72200237

ShSirt1_vs_ShScrambled	Q8BH60	Q8BH60	Gopc	94221	-0.170750933	0.5636633	0.72242366
ShSirt1_vs_ShScrambled	P27046	P27046	Man2a1	17158	0.101261132	0.56422261	0.72261293
ShSirt1_vs_ShScrambled	Q9D1D4	Q9D1D4	Tmed10	68581	-0.259388838	0.56428165	0.72261293
ShSirt1_vs_ShScrambled	Q8BG67	Q8BG67	Efr3a	76740	0.24151021	0.56429122	0.72261293
ShSirt1_vs_ShScrambled	Q9D1P4	Q9D1P4	Chordc1	66917	0.40629586	0.56466901	0.72289163
ShSirt1_vs_ShScrambled	Q9Z2R6	Q9Z2R6	Unc119	22248	0.309949043	0.56497207	0.72296175
ShSirt1_vs_ShScrambled	O35326	O35326	Srsf5	20384	0.184904657	0.56531288	0.72296175
ShSirt1_vs_ShScrambled	Q99KF1	Q99KF1	Tmed9	67511	-0.091422114	0.56533523	0.72296175
ShSirt1_vs_ShScrambled	Q9CY50	Q9CY50	Ssr1	107513	0.165644262	0.56536442	0.72296175
ShSirt1_vs_ShScrambled	Q80TA9	Q80TA9	Epg5	1.01E+08	0.203136044	0.56624736	0.72363628
ShSirt1_vs_ShScrambled	Q61941	Q61941	Nnt		-0.069088372	0.56630053	0.72363628
ShSirt1_vs_ShScrambled	Q4KML4	Q4KML4	Abracl	73112	-0.166086795	0.56637778	0.72363628
ShSirt1_vs_ShScrambled	Q8BX17	Q8BX17	Gemin5	216766	0.076254326	0.56660503	0.72363628
ShSirt1_vs_ShScrambled	B2RRE7	B2RRE7	Otud4	73945	-0.422071024	0.56682218	0.72363628
ShSirt1_vs_ShScrambled	Q3V3Q7	Q3V3Q7	Pacs2	217893	-0.462137229	0.5669825	0.72363628
ShSirt1_vs_ShScrambled	Q91YN9	Q91YN9	Bag2	213539	-0.175090509	0.56707104	0.72363628
ShSirt1_vs_ShScrambled	Q9WV85	Q9WV85	Nme3	79059	-0.82865104	0.56717438	0.72363628
ShSirt1_vs_ShScrambled	P62488	P62488	Polr2g	67710	0.500957698	0.56772974	0.72414016
ShSirt1_vs_ShScrambled	Q8BIQ5	Q8BIQ5	Cstf2	108062	-0.152529018	0.56808819	0.72439268
ShSirt1_vs_ShScrambled	P42859	P42859	Htt		0.564603323	0.56907884	0.72545097
ShSirt1_vs_ShScrambled	O88983	O88983	Stx8	55943	0.299377467	0.56969466	0.72603097
ShSirt1_vs_ShScrambled	Q52KI8	Q52KI8	Srrm1	51796	0.130334383	0.57041656	0.72674579
ShSirt1_vs_ShScrambled	Q8BXQ2	Q8BXQ2	Pigt	78928	0.108686421	0.5707118	0.72691677
ShSirt1_vs_ShScrambled	Q9ER41	Q9ER41	Tor1b	30934	0.248416449	0.5711888	0.72731911
ShSirt1_vs_ShScrambled	O88572	O88572	Lrp6	16974	-0.29570852	0.57235478	0.72859828
ShSirt1_vs_ShScrambled	Q8R361	Q8R361	Rab11fip5	52055	-0.218278535	0.57315308	0.7294088
ShSirt1_vs_ShScrambled	Q8VCT3	Q8VCT3	Rnpep	215615	0.122951154	0.5735364	0.7296909
ShSirt1_vs_ShScrambled	Q8C9S4	Q8C9S4	Ccdc186	213993	-0.124132552	0.57443525	0.73049153
ShSirt1_vs_ShScrambled	P42125	P42125	Eci1	13177	-0.133845634	0.57448935	0.73049153
ShSirt1_vs_ShScrambled	Q8BHG1	Q8BHG1	Nrd1	230598	0.153271265	0.5757601	0.73190119
ShSirt1_vs_ShScrambled	Q61334	Q61334	Bcap29	12033	-0.129763965	0.57603364	0.73204276
ShSirt1_vs_ShScrambled	Q8K2L8	Q8K2L8	Trappc12	217449	-0.116908418	0.57629401	0.73216752
ShSirt1_vs_ShScrambled	Q3UHA3	Q3UHA3	Spg11	214585	-0.118916919	0.57728012	0.73321398
ShSirt1_vs_ShScrambled	Q8BH43	Q8BH43	Wasf2	242687	0.129264599	0.57745241	0.7332265
ShSirt1_vs_ShScrambled	Q9Z2Q5	Q9Z2Q5	Mrpl40	18100	-0.091211138	0.57794748	0.73355201
ShSirt1_vs_ShScrambled	Q921G7	Q921G7	Etfdh	66841	-0.067481135	0.57803378	0.73355201
ShSirt1_vs_ShScrambled	Q9D486	Q9D486	Cmip	74440	0.154653623	0.57829686	0.73367152
ShSirt1_vs_ShScrambled	Q99L85	Q99L85	Elp5	54351	0.168659711	0.57845302	0.73367152
ShSirt1_vs_ShScrambled	Q8BHD7	Q8BHD7	Ptbp3	230257	-0.149702901	0.57895803	0.73393589
ShSirt1_vs_ShScrambled	Q80U56	Q80U56	Avl9	78937	-0.158198611	0.57898664	0.73393589
ShSirt1_vs_ShScrambled	P24527	P24527	Lta4h	16993	0.154470032	0.57929947	0.73412627
ShSirt1_vs_ShScrambled	Q8K003	Q8K003	Tma7	66167	-0.262565593	0.58034978	0.7351939
ShSirt1_vs_ShScrambled	Q9CPU4	Q9CPU4	Mgst3	66447	-0.142382328	0.58060808	0.7351939
ShSirt1_vs_ShScrambled	O08528	O08528	Hk2	15277	0.081956645	0.58068319	0.7351939
ShSirt1_vs_ShScrambled	P70404	P70404	Idh3g	15929	-0.100913676	0.58108361	0.7351939
ShSirt1_vs_ShScrambled	Q9QXT0	Q9QXT0	Cnpy2	56530	0.103730597	0.58114923	0.7351939
ShSirt1_vs_ShScrambled	Q99KY4	Q99KY4	Gak	231580	-0.079363896	0.58124478	0.7351939
ShSirt1_vs_ShScrambled	P60766	P60766	Cdc42	12540	0.108227235	0.58141652	0.7351939
ShSirt1_vs_ShScrambled	A3KMP2	A3KMP2	Ttc38	239570	0.081739543	0.58149409	0.7351939
ShSirt1_vs_ShScrambled	P61963	P61963	Dcaf7	71833	-0.383855242	0.58160776	0.7351939
ShSirt1_vs_ShScrambled	Q8BVI4	Q8BVI4	Qdpr	110391	-0.077838373	0.58178652	0.73521398
ShSirt1_vs_ShScrambled	P00493	P00493	Hprt1	15452	-0.0686518	0.58327514	0.73688889
ShSirt1_vs_ShScrambled	P63001	P63001;Q05	Rac1		0.282421442	0.58406906	0.73760348
ShSirt1_vs_ShScrambled	Q8K3D3	Q8K3D3	Swi5	72931	0.421396816	0.58437043	0.73760348
ShSirt1_vs_ShScrambled	P46737	P46737	Brcc3	210766	-0.324738698	0.58440169	0.73760348
ShSirt1_vs_ShScrambled	Q8BKS9	Q8BKS9	Kiaa0020	52874	0.074044916	0.58449439	0.73760348
ShSirt1_vs_ShScrambled	Q9ESL4	Q9ESL4	Zak	65964	-0.448922865	0.58482412	0.73769035
ShSirt1_vs_ShScrambled	Q566J8	Q566J8	Adck4		0.26358943	0.58489006	0.73769035
ShSirt1_vs_ShScrambled	O35737	O35737	Hnrnph1	59013	0.099904538	0.58552443	0.73828415
ShSirt1_vs_ShScrambled	P62830	P62830	Rpl23	65019; 10	0.079853348	0.5857219	0.7383269
ShSirt1_vs_ShScrambled	Q9DCD2	Q9DCD2	Xab2	67439	0.22555483	0.58669776	0.73916846
ShSirt1_vs_ShScrambled	P70195	P70195	Psmb7	19177	0.258593834	0.58676244	0.73916846
ShSirt1_vs_ShScrambled	Q9R0X4	Q9R0X4	Acot9	56360	0.109080164	0.58690239	0.73916846

ShSirt1_vs_ShScrambled	B7ZMP1	B7ZMP1	Xpnpep3	321003	0.103321426	0.58704451	0.73916846
ShSirt1_vs_ShScrambled	Q9D832	Q9D832	Dnajb4	67035	-0.082872546	0.58727001	0.73924619
ShSirt1_vs_ShScrambled	Q6NZF1	Q6NZF1	Zc3h11a	70579	0.186093895	0.58758664	0.73943856
ShSirt1_vs_ShScrambled	Q8VED2	Q8VED2	Bloc1s4	117197	0.113391768	0.58793058	0.73966517
ShSirt1_vs_ShScrambled	P62270	P62270	Rps18	20084	0.080986483	0.58931069	0.74119489
ShSirt1_vs_ShScrambled	Q99N96	Q99N96	Mrpl1	94061	-0.18252171	0.58947998	0.74120129
ShSirt1_vs_ShScrambled	Q9CXK8	Q9CXK8	Nip7	66164	0.138017083	0.590083	0.74175289
ShSirt1_vs_ShScrambled	Q9WTP7	Q9WTP7	Ak3	56248	0.070990918	0.5903264	0.74185227
ShSirt1_vs_ShScrambled	P27546	P27546	Map4	17758	-0.095179788	0.59070277	0.74205062
ShSirt1_vs_ShScrambled	Q6P9Q4	Q6P9Q4	Fhod1	234686	-0.137502156	0.59084355	0.74205062
ShSirt1_vs_ShScrambled	Q6P9J9	Q6P9J9	Ano6	105722	0.1541209	0.59105392	0.74205062
ShSirt1_vs_ShScrambled	Q9ER88	Q9ER88	Dap3	65111	-0.104793685	0.59114178	0.74205062
ShSirt1_vs_ShScrambled	Q91WC3	Q91WC3	Acsl6	216739	0.211080535	0.59193302	0.74283726
ShSirt1_vs_ShScrambled	P0DN91	P0DN91;P0D	P0DN91		0.171927123	0.5925468	0.74335488
ShSirt1_vs_ShScrambled	Q9DBV4	Q9DBV4	Mxra8	74761	0.543122467	0.59267484	0.74335488
ShSirt1_vs_ShScrambled	P70303	P70303	Ctps2	55936	0.084955682	0.59295485	0.74349949
ShSirt1_vs_ShScrambled	Q8BHA3	Q8BHA3	Dtd2	328092	0.230409896	0.59362247	0.74412991
ShSirt1_vs_ShScrambled	Q3URD3	Q3URD3	Slmap	83997	0.437535983	0.59386105	0.74422231
ShSirt1_vs_ShScrambled	P09671	P09671	Sod2	20656	0.11039684	0.59437574	0.74455028
ShSirt1_vs_ShScrambled	Q9QYJ0	Q9QYJ0	Dnaja2	56445	-0.084610184	0.59445264	0.74455028
ShSirt1_vs_ShScrambled	Q91W39	Q91W39	Ncoa5	228869	-0.162178379	0.59518182	0.74525679
ShSirt1_vs_ShScrambled	Q9QYI3	Q9QYI3	Dnajc7	56354	0.11349374	0.59564554	0.74551801
ShSirt1_vs_ShScrambled	Q811I0	Q811I0	Atpaf1		0.103083901	0.59572075	0.74551801
ShSirt1_vs_ShScrambled	P56399	P56399	Usp5	22225	-0.066209602	0.5969919	0.74690173
ShSirt1_vs_ShScrambled	Q9WTX8	Q9WTX8	Mad1l1	17120	-0.140077404	0.59720374	0.74694786
ShSirt1_vs_ShScrambled	Q9D1E8	Q9D1E8	Agpat5	52123	0.105438704	0.59735972	0.74694786
ShSirt1_vs_ShScrambled	O70492	O70492	Snx3		0.173686686	0.59828602	0.74770414
ShSirt1_vs_ShScrambled	Q9DB25	Q9DB25	Alg5	66248	0.192080219	0.59829583	0.74770414
ShSirt1_vs_ShScrambled	Q9EPN1	Q9EPN1	Nbea	26422	0.336784141	0.59900483	0.748383
ShSirt1_vs_ShScrambled	Q3TFQ1	Q3TFQ1	Spryd7	66674	0.102453429	0.59924968	0.74848176
ShSirt1_vs_ShScrambled	Q9ES89	Q9ES89	Extl2	58193	-0.089937195	0.60079739	0.75020731
ShSirt1_vs_ShScrambled	E9Q3E1	E9Q3E1;J3Q	Aldh3b2		-0.217913139	0.60121076	0.75051586
ShSirt1_vs_ShScrambled	Q9R0P3	Q9R0P3	Esd	13885	-0.095997975	0.60158116	0.75077063
ShSirt1_vs_ShScrambled	Q91VI7	Q91VI7	Rnh1	107702	-0.081455144	0.60206098	0.75116177
ShSirt1_vs_ShScrambled	P58281	P58281	Opa1	74143	-0.097766784	0.60234956	0.75131415
ShSirt1_vs_ShScrambled	Q9CZJ1	Q9CZJ1	Utp11l	67205	-0.295366844	0.60334486	0.75234771
ShSirt1_vs_ShScrambled	Q9CQ20	Q9CQ20	Mid1ip1	68041	-0.335904896	0.60356773	0.75239747
ShSirt1_vs_ShScrambled	P47754	P47754	Capza2	12343	-0.089640541	0.60371813	0.75239747
ShSirt1_vs_ShScrambled	Q3TWN3	Q3TWN3	Cnnm2	94219	-0.17769441	0.60406876	0.75261835
ShSirt1_vs_ShScrambled	Q9DAS9	Q9DAS9	Gng12	14701	-0.079300132	0.60422882	0.75261835
ShSirt1_vs_ShScrambled	O70305	O70305	Atxn2	20239	-0.100718522	0.60451471	0.75276674
ShSirt1_vs_ShScrambled	Q3U487	Q3U487	Hectd3	76608	0.086988608	0.60753033	0.75631326
ShSirt1_vs_ShScrambled	Q99JI4	Q99JI4	Psmd6	66413	0.08768758	0.60822082	0.7569641
ShSirt1_vs_ShScrambled	A6H611	A6H611	Mipep	70478	-0.120635729	0.60844958	0.75704008
ShSirt1_vs_ShScrambled	Q04690	Q04690	Nf1	18015	-0.284538954	0.60862761	0.75705292
ShSirt1_vs_ShScrambled	Q9Z1E4	Q9Z1E4	Gys1	14936	-0.062963619	0.60889286	0.7571742
ShSirt1_vs_ShScrambled	Q3UE37	Q3UE37	Ube2z	268470	0.11415708	0.60926271	0.75742546
ShSirt1_vs_ShScrambled	Q6PEB6	Q6PEB6	Mob4	19070	-0.466382631	0.60947366	0.7574791
ShSirt1_vs_ShScrambled	E9Q5C9	E9Q5C9	Nolc1	70769	0.115172812	0.60967954	0.75752641
ShSirt1_vs_ShScrambled	Q99N93	Q99N93	Mrpl16	94063	-0.09082911	0.6107469	0.75864378
ShSirt1_vs_ShScrambled	Q01705	Q01705	Notch1	18128	0.150363465	0.61128207	0.75900324
ShSirt1_vs_ShScrambled	Q9D8N2	Q9D8N2	Fam45a	67894	0.197767882	0.6114629	0.75900324
ShSirt1_vs_ShScrambled	Q6Y7W8	Q6Y7W8	Gigyf2	227331	0.129285179	0.61154071	0.75900324
ShSirt1_vs_ShScrambled	Q80TH2	Q80TH2	Erbb2ip	59079	-0.137455079	0.61182739	0.75915032
ShSirt1_vs_ShScrambled	Q6PGC1	Q6PGC1	Dhx29	218629	0.230812197	0.61216199	0.75915507
ShSirt1_vs_ShScrambled	Q91YQ5	Q91YQ5	Rpn1	103963	-0.075798062	0.61216758	0.75915507
ShSirt1_vs_ShScrambled	Q9QXN3	Q9QXN3	Trip4	56404	-0.159790282	0.61258446	0.7594634
ShSirt1_vs_ShScrambled	O88512	O88512	Ap1g2	11766	0.198165566	0.61310107	0.75989517
ShSirt1_vs_ShScrambled	Q8CGZ0	Q8CGZ0	Cherp	27967	0.255273361	0.61490122	0.76191713
ShSirt1_vs_ShScrambled	Q61469	Q61469	Ppap2a	19012	0.194638036	0.61626862	0.76322815
ShSirt1_vs_ShScrambled	Q6PAV2	Q6PAV2	Herc4	67345	-0.103030526	0.61629743	0.76322815
ShSirt1_vs_ShScrambled	Q9CZ30	Q9CZ30	Ola1	67059	-0.07798662	0.61654669	0.76332741
ShSirt1_vs_ShScrambled	Q9Z2F7	Q9Z2F7	Bnip3l	12177	0.123218685	0.61800087	0.76491799

ShSirt1_vs_ShScrambled	Q6ZQ08	Q6ZQ08	Cnot1	234594	0.100740473	0.61924183	0.76611812
ShSirt1_vs_ShScrambled	Q3UHB1	Q3UHB1	Nt5dc3	103466	-0.076628974	0.61930993	0.76611812
ShSirt1_vs_ShScrambled	Q8BWQ6	Q8BWQ6	Q8BWQ6	71517	0.140536231	0.62013848	0.76665762
ShSirt1_vs_ShScrambled	Q6PDK2	Q6PDK2	Kmt2d		0.132798061	0.6202255	0.76665762
ShSirt1_vs_ShScrambled	Q63850	Q63850	Nup62	18226	-0.2497706	0.62025557	0.76665762
ShSirt1_vs_ShScrambled	P48453	P48453	Ppp3cb	19056	-0.147209179	0.62106786	0.7672392
ShSirt1_vs_ShScrambled	P17426	P17426	Ap2a1	11771	-0.095943185	0.62112111	0.7672392
ShSirt1_vs_ShScrambled	Q9JKF7	Q9JKF7	Mrpl39	27393	-0.127143458	0.62131793	0.7672392
ShSirt1_vs_ShScrambled	P62334	P62334	Psmc6	67089	0.078581335	0.62140596	0.7672392
ShSirt1_vs_ShScrambled	Q99PU8	Q99PU8	Dhx30	72831	-0.119948151	0.6218066	0.76752392
ShSirt1_vs_ShScrambled	Q8CF66	Q8CF66	Lamtor4	66096	-0.192694204	0.62225709	0.76787001
ShSirt1_vs_ShScrambled	Q9CQC9	Q9CQC9	Sar1b	66397	0.204292202	0.62256691	0.76804237
ShSirt1_vs_ShScrambled	P99029	P99029	Prdx5	54683	0.074560106	0.62344702	0.76891799
ShSirt1_vs_ShScrambled	P22366	P22366	Myd88	17874	0.242239531	0.62388702	0.76925049
ShSirt1_vs_ShScrambled	Q6PDY2	Q6PDY2	Ado	211488	-0.167828185	0.624353	0.76934363
ShSirt1_vs_ShScrambled	Q8BY87	Q8BY87	Usp47	74996	0.099713923	0.62437537	0.76934363
ShSirt1_vs_ShScrambled	P70451	P70451	Fer	14158	-0.222503892	0.62447388	0.76934363
ShSirt1_vs_ShScrambled	P47753	P47753	Capza1		0.077364725	0.62481095	0.76954888
ShSirt1_vs_ShScrambled	Q924C1	Q924C1	Xpo5	72322	-0.1318323	0.62510262	0.76968988
ShSirt1_vs_ShScrambled	P61514	P61514	Rpl37a	19981	0.092162774	0.62526646	0.76968988
ShSirt1_vs_ShScrambled	P61202	P61202	Cops2	12848	0.099527664	0.62562124	0.76991665
ShSirt1_vs_ShScrambled	Q9DCC4	Q9DCC4	Pycrl	66194	0.12630582	0.62623966	0.77046765
ShSirt1_vs_ShScrambled	O09131	O09131	Gsto1	14873	0.090725066	0.62657332	0.77066811
ShSirt1_vs_ShScrambled	Q8BTY1	Q8BTY1	Ccbl1	70266	0.099875447	0.62716894	0.77073393
ShSirt1_vs_ShScrambled	P84096	P84096	Rhog	56212	-0.076482505	0.62732569	0.77073393
ShSirt1_vs_ShScrambled	O08795	O08795	Prkcsh	19089	0.088395129	0.62734283	0.77073393
ShSirt1_vs_ShScrambled	Q62084	Q62084	Ppp1r14b	18938	-0.152383211	0.62740292	0.77073393
ShSirt1_vs_ShScrambled	Q80U35	Q80U35	Arhgef17	207212	-0.559877648	0.62748055	0.77073393
ShSirt1_vs_ShScrambled	Q9CQL6	Q9CQL6	Mrpl35	66223	-0.195076662	0.62778601	0.77089936
ShSirt1_vs_ShScrambled	P59470	P59470	Polr3b	70428	0.189228064	0.6281149	0.77109346
ShSirt1_vs_ShScrambled	Q8CHY6	Q8CHY6	Gatad2a	234366	-0.128519806	0.62894711	0.77190518
ShSirt1_vs_ShScrambled	Q8BFU3	Q8BFU3	Rnf214	235315	-0.10763171	0.62924396	0.77191248
ShSirt1_vs_ShScrambled	P20029	P20029	Hspa5	14828	0.069770632	0.62929506	0.77191248
ShSirt1_vs_ShScrambled	Q8BWZ3	Q8BWZ3	Naa25	231713	0.12268693	0.62951554	0.77197314
ShSirt1_vs_ShScrambled	P99024	P99024	Tubb5	22154	-0.073582454	0.62970932	0.77200105
ShSirt1_vs_ShScrambled	Q8QZZ7	Q8QZZ7	Tprkb	69786	-0.091298854	0.63044546	0.77269367
ShSirt1_vs_ShScrambled	Q9WU84	Q9WU84	Ccs	12460	0.100316431	0.63090262	0.77288501
ShSirt1_vs_ShScrambled	Q8C2E4	Q8C2E4	Ptcd1	71799	-0.129328791	0.63097914	0.77288501
ShSirt1_vs_ShScrambled	Q60989	Q60989	Xiap	11798	-0.221986191	0.63125915	0.77288501
ShSirt1_vs_ShScrambled	P62281	P62281	Rps11	27207	0.087550614	0.63128645	0.77288501
ShSirt1_vs_ShScrambled	P61967	P61967	Ap1s1	11769	-0.075782169	0.63217092	0.77366958
ShSirt1_vs_ShScrambled	Q8BWY3	Q8BWY3	Etf1	225363	0.065712912	0.63227006	0.77366958
ShSirt1_vs_ShScrambled	Q7TMQ7	Q7TMQ7	Wdr91	101240	0.126045461	0.63271002	0.77399811
ShSirt1_vs_ShScrambled	Q8K2B3	Q8K2B3	Sdha	66945	-0.085057899	0.63355398	0.77482055
ShSirt1_vs_ShScrambled	A2AKG8	A2AKG8	Focad	230393	0.158142424	0.63458288	0.77586866
ShSirt1_vs_ShScrambled	Q8C3F2	Q8C3F2	Fam120c	207375	-0.185315742	0.6347799	0.77589939
ShSirt1_vs_ShScrambled	Q8BJZ4	Q8BJZ4	Mrps35	232536	0.083603019	0.63528422	0.77623133
ShSirt1_vs_ShScrambled	Q6ZQL4	Q6ZQL4	Wdr43	72515	-0.164385474	0.63539539	0.77623133
ShSirt1_vs_ShScrambled	Q9CQP2	Q9CQP2	Trappc2	66050; 66	-0.129514583	0.63585521	0.77658291
ShSirt1_vs_ShScrambled	Q8BRF7	Q8BRF7	Scfd1	76983	0.077486366	0.63614405	0.77672552
ShSirt1_vs_ShScrambled	O88630	O88630	Gosr1	53334	-0.220402287	0.63658434	0.77688568
ShSirt1_vs_ShScrambled	Q9CWK8	Q9CWK8	Snx2	67804	-0.080259629	0.63661944	0.77688568
ShSirt1_vs_ShScrambled	Q9WTK3	Q9WTK3	Gpaa1	14731	0.219284391	0.63812683	0.77851473
ShSirt1_vs_ShScrambled	Q8VDM6	Q8VDM6	Hnrnpul1	232989	0.077768621	0.63896825	0.77933064
ShSirt1_vs_ShScrambled	Q3TZZ7	Q3TZZ7	Esyt2	52635	0.087316018	0.63923664	0.77944738
ShSirt1_vs_ShScrambled	Q922B1	Q922B1	Macrod1	107227	0.230835049	0.63947099	0.77951865
ShSirt1_vs_ShScrambled	Q5SYD0	Q5SYD0	Myo1d	338367	0.143059136	0.63964047	0.77951865
ShSirt1_vs_ShScrambled	Q6IRU2	Q6IRU2	Tpm4	326618	0.109671548	0.64030121	0.78007774
ShSirt1_vs_ShScrambled	Q6X893	Q6X893	Slc44a1	100434	-0.094717517	0.64060458	0.78007774
ShSirt1_vs_ShScrambled	Q91VE0	Q91VE0	Slc27a4	26569	-0.092082881	0.64090401	0.78007774
ShSirt1_vs_ShScrambled	Q9R0Q7	Q9R0Q7	Ptges3	56351	0.100421678	0.64093682	0.78007774
ShSirt1_vs_ShScrambled	Q60974	Q60974	Ncor1		-0.140638074	0.6409633	0.78007774
ShSirt1_vs_ShScrambled	O08547	O08547	Sec22b	20333	0.05721945	0.64174005	0.78066392

ShSirt1_vs_ShScrambled	P56812	P56812	Pdcd5	56330	0.056180667	0.64179083	0.78066392
ShSirt1_vs_ShScrambled	Q6R0H7	Q6R0H7;P63	Gnas		0.080191437	0.64210234	0.78078869
ShSirt1_vs_ShScrambled	P97821	P97821	Ctsc	13032	0.214299598	0.64223935	0.78078869
ShSirt1_vs_ShScrambled	Q91VM5	Q91VM5	Rbmxl1	19656	0.071814701	0.64284468	0.78131419
ShSirt1_vs_ShScrambled	Q9JJA4	Q9JJA4	Wdr12	57750	0.106719958	0.64361176	0.78203594
ShSirt1_vs_ShScrambled	Q8K2A8	Q8K2A8	Alg3	208624	-0.114068425	0.64428044	0.78263776
ShSirt1_vs_ShScrambled	Q80YR5	Q80YR5	Safb2	224902	-0.168419665	0.64535006	0.78372617
ShSirt1_vs_ShScrambled	Q8CFV9	Q8CFV9	Rfk	54391	-0.107500201	0.64710748	0.78564905
ShSirt1_vs_ShScrambled	Q3TPX4	Q3TPX4	Exoc5	105504	0.310303314	0.64731311	0.78568738
ShSirt1_vs_ShScrambled	O35972	O35972	Mrpl23	19935	-0.068227292	0.64775074	0.78570952
ShSirt1_vs_ShScrambled	Q80X85	Q80X85	Mrps7	50529	-0.117280787	0.64776006	0.78570952
ShSirt1_vs_ShScrambled	Q99J62	Q99J62	Rfc4	106344	0.119874582	0.64785353	0.78570952
ShSirt1_vs_ShScrambled	Q6P9R2	Q6P9R2	Oxsr1	108737	-0.069048549	0.6482778	0.78601289
ShSirt1_vs_ShScrambled	Q91VY5	Q91VY5	Kdm4b	193796	0.367172243	0.64940503	0.78716818
ShSirt1_vs_ShScrambled	Q8BR90	Q8BR90	Q8BR90	106064	-0.097083597	0.65016569	0.78758928
ShSirt1_vs_ShScrambled	Q8BKF1	Q8BKF1	Polrmt	216151	0.093533836	0.65032787	0.78758928
ShSirt1_vs_ShScrambled	Q9CR26	Q9CR26	Vta1	66201	0.074540303	0.65034345	0.78758928
ShSirt1_vs_ShScrambled	Q9D1M4	Q9D1M4	Eef1e1	66143	0.090226236	0.65045034	0.78758928
ShSirt1_vs_ShScrambled	Q8BX02	Q8BX02	Kank2	235041	-0.071178347	0.65085138	0.78782746
ShSirt1_vs_ShScrambled	Q33DR2	Q33DR2	Pdss1	56075	0.55379969	0.65114001	0.78782746
ShSirt1_vs_ShScrambled	Q91W96	Q91W96	Anapc4	52206	-0.085808479	0.65130167	0.78782746
ShSirt1_vs_ShScrambled	Q9D903	Q9D903	Ebna1bp2	69072	0.103800656	0.6513951	0.78782746
ShSirt1_vs_ShScrambled	Q5XJY5	Q5XJY5	Arcn1	213827	-0.07135688	0.65178026	0.78782746
ShSirt1_vs_ShScrambled	Q61337	Q61337	Bad	12015	-0.086829242	0.65181014	0.78782746
ShSirt1_vs_ShScrambled	Q9JMG1	Q9JMG1	Edf1	59022	-0.110370152	0.65186876	0.78782746
ShSirt1_vs_ShScrambled	Q91XA2	Q91XA2	Golm1	105348	-0.155397039	0.65207564	0.78786035
ShSirt1_vs_ShScrambled	Q5SSK3	Q5SSK3	Tefm	68550	-0.238222878	0.65224504	0.78786035
ShSirt1_vs_ShScrambled	O35857	O35857	Timm44	21856	0.05926667	0.65353452	0.78920675
ShSirt1_vs_ShScrambled	B1AZI6	B1AZI6	Thoc2	331401	-0.099001793	0.65426295	0.7897158
ShSirt1_vs_ShScrambled	O88952	O88952	Lin7c	22343	-0.125088043	0.65437616	0.7897158
ShSirt1_vs_ShScrambled	Q8C436	Q8C436	Vcpkmt	207965	-0.173937171	0.65448091	0.7897158
ShSirt1_vs_ShScrambled	Q8BTW8	Q8BTW8	Cdk5rap1	66971	0.35233075	0.65526513	0.79016082
ShSirt1_vs_ShScrambled	Q91YE3	Q91YE3	Egln1	112405	-0.422469291	0.65526663	0.79016082
ShSirt1_vs_ShScrambled	Q5SUQ9	Q5SUQ9	Ctc1	68964	0.286951072	0.65549851	0.79016082
ShSirt1_vs_ShScrambled	Q9D6L8	Q9D6L8	Ppil3	70225	-0.124478766	0.6555499	0.79016082
ShSirt1_vs_ShScrambled	Q8C0E2	Q8C0E2	Vps26b	69091	-0.140037418	0.65578294	0.79023069
ShSirt1_vs_ShScrambled	Q8CH18	Q8CH18	Ccar1	67500	-0.073770676	0.65606891	0.79034797
ShSirt1_vs_ShScrambled	Q5SVR0	Q5SVR0	Tbc1d9b	76795	-0.083659001	0.65624779	0.79034797
ShSirt1_vs_ShScrambled	Q9DAW6	Q9DAW6	Prpf4	70052	-0.464406417	0.65647021	0.79034797
ShSirt1_vs_ShScrambled	Q9CQ56	Q9CQ56	Use1	67023	-0.116952536	0.65659364	0.79034797
ShSirt1_vs_ShScrambled	Q8VD00	Q8VD00	Tmem97	69071	0.10360101	0.65689783	0.79034797
ShSirt1_vs_ShScrambled	Q91WG2	Q91WG2	Rabep2	70314	-0.178296714	0.65693079	0.79034797
ShSirt1_vs_ShScrambled	Q8R2N2	Q8R2N2	Cirh1a	21771	0.112672835	0.65727261	0.7903619
ShSirt1_vs_ShScrambled	P22315	P22315	Fech		-0.07401779	0.65756164	0.7903619
ShSirt1_vs_ShScrambled	Q9D173	Q9D173	Tomm7	66169	-0.286840241	0.65758339	0.7903619
ShSirt1_vs_ShScrambled	Q8CI94	Q8CI94	Pygb	110078	-0.059370061	0.65764273	0.7903619
ShSirt1_vs_ShScrambled	Q8K2V1	Q8K2V1	Ppp4r1	70351	-0.153822182	0.65790531	0.79046702
ShSirt1_vs_ShScrambled	Q8BHS3	Q8BHS3	Rbm22	66810	0.347617789	0.65824172	0.79066075
ShSirt1_vs_ShScrambled	Q8BGQ7	Q8BGQ7	Aars	234734	-0.066061778	0.65875725	0.79104459
ShSirt1_vs_ShScrambled	P62242	P62242	Rps8	20116	0.116051492	0.65891175	0.79104459
ShSirt1_vs_ShScrambled	Q8R2Y3	Q8R2Y3	Dolk	227697	-0.150097781	0.65947092	0.79150538
ShSirt1_vs_ShScrambled	P70697	P70697	Urod	22275	-0.091915818	0.65972306	0.79159753
ShSirt1_vs_ShScrambled	Q91VJ4	Q91VJ4	Stk38	106504	-0.081726834	0.6606782	0.79221546
ShSirt1_vs_ShScrambled	P28656	P28656	Nap1l1	53605	-0.096505502	0.6606913	0.79221546
ShSirt1_vs_ShScrambled	Q64511	Q64511	Top2b	21974	0.092350813	0.66093055	0.79221546
ShSirt1_vs_ShScrambled	Q3U319	Q3U319	Rnf40	233900	0.2055035	0.66094006	0.79221546
ShSirt1_vs_ShScrambled	P61164	P61164	Actr1a	54130	-0.070664384	0.66134951	0.79249581
ShSirt1_vs_ShScrambled	P58059	P58059	Mrps21	66292	-0.309166379	0.6622457	0.79309683
ShSirt1_vs_ShScrambled	Q5NCF2	Q5NCF2	Trappc1	245828	-0.165690643	0.66228127	0.79309683
ShSirt1_vs_ShScrambled	Q63943	Q63943	Mef2d	17261	-0.118780974	0.66248146	0.79309683
ShSirt1_vs_ShScrambled	Q9CQ48	Q9CQ48	Nudcd2	52653	0.067276037	0.66267404	0.79309683
ShSirt1_vs_ShScrambled	Q8BUR4	Q8BUR4	Dock1	330662	0.082770039	0.66287704	0.79309683
ShSirt1_vs_ShScrambled	Q60854	Q60854	Serpinb6	20719	0.099007918	0.66305902	0.79309683

ShSirt1_vs_ShScrambled	Q9WV80	Q9WV80	Snx1	56440	0.05850497	0.66308095	0.79309683
ShSirt1_vs_ShScrambled	Q9CT10	Q9CT10	Ranbp3	71810	0.064755558	0.66354886	0.79344624
ShSirt1_vs_ShScrambled	Q60759	Q60759	Gcdh	270076	0.084738419	0.66382721	0.79356887
ShSirt1_vs_ShScrambled	P50172	P50172	Hsd11b1	15483	-0.100326592	0.66488823	0.79443546
ShSirt1_vs_ShScrambled	P68040	P68040	Gnb2l1	14694	0.059713909	0.66490411	0.79443546
ShSirt1_vs_ShScrambled	Q8R0N6	Q8R0N6	Adhfe1	76187	-0.079979845	0.6653772	0.79479034
ShSirt1_vs_ShScrambled	P63166	P63166	Sumo1	22218	0.084981331	0.66556505	0.7948044
ShSirt1_vs_ShScrambled	Q80U72	Q80U72	Scrib	105782	0.096037073	0.66623883	0.79539859
ShSirt1_vs_ShScrambled	P48774	P48774	Gstm5	14866	-0.17329248	0.66660263	0.79561381
ShSirt1_vs_ShScrambled	Q9QZB7	Q9QZB7	Actr10	56444	-0.207333704	0.66691728	0.79561381
ShSirt1_vs_ShScrambled	Q8CFJ9	Q8CFJ9	Wdr24	268933	0.1165285	0.66694786	0.79561381
ShSirt1_vs_ShScrambled	O35682	O35682	Myadm	50918	-0.090402085	0.66724549	0.79575856
ShSirt1_vs_ShScrambled	P62835	P62835	Rap1a	109905	0.097609718	0.6700689	0.79873118
ShSirt1_vs_ShScrambled	P83940	P83940	Tceb1	67923	0.132878021	0.67009466	0.79873118
ShSirt1_vs_ShScrambled	Q8K2Q7	Q8K2Q7	Brox	71678	0.094937873	0.67031792	0.79873118
ShSirt1_vs_ShScrambled	Q9DAU1	Q9DAU1	Cnpy3	72029	0.119348511	0.67044582	0.79873118
ShSirt1_vs_ShScrambled	Q925J9	Q925J9	Med1	19014	0.164612523	0.67097079	0.79914568
ShSirt1_vs_ShScrambled	P61982	P61982	Ywhag	22628	0.066270389	0.67156682	0.79945551
ShSirt1_vs_ShScrambled	Q9D9Z5	Q9D9Z5	Dda1	66498	0.168974982	0.67158514	0.79945551
ShSirt1_vs_ShScrambled	Q3UGS4	Q3UGS4	Fam195b	192173	-0.195790104	0.67184636	0.79954023
ShSirt1_vs_ShScrambled	A2AJ15	A2AJ15	Man1b1	227619	-0.112770275	0.67201055	0.79954023
ShSirt1_vs_ShScrambled	P36536	P36536	Sar1a		-0.069042671	0.67236816	0.79965341
ShSirt1_vs_ShScrambled	Q91ZR2	Q91ZR2	Snx18		0.107709793	0.67261202	0.79965341
ShSirt1_vs_ShScrambled	Q61735	Q61735	Cd47	16423	0.119318299	0.67285138	0.79965341
ShSirt1_vs_ShScrambled	Q63810	Q63810	Ppp3r1	19058	-0.532747501	0.6728796	0.79965341
ShSirt1_vs_ShScrambled	P23492	P23492	Pnp	18950	0.064164221	0.67299142	0.79965341
ShSirt1_vs_ShScrambled	Q8CHG3	Q8CHG3	Gcc2		-0.070972498	0.67400427	0.80050436
ShSirt1_vs_ShScrambled	C0HKE9	C0HKE9;C0H	Hist1h2ah		0.311837034	0.67406226	0.80050436
ShSirt1_vs_ShScrambled	Q9JLT4	Q9JLT4	Txnrd2	26462	0.129020168	0.67439283	0.8006074
ShSirt1_vs_ShScrambled	Q8VCX5	Q8VCX5	Micu1	216001	0.340958264	0.67450376	0.8006074
ShSirt1_vs_ShScrambled	Q8K1R7	Q8K1R7	Nek9	217718	0.104353564	0.67484963	0.8006155
ShSirt1_vs_ShScrambled	Q4PJX1	Q4PJX1	Odr4	226499	-0.157995959	0.6748653	0.8006155
ShSirt1_vs_ShScrambled	Q9WVC3	Q9WVC3	Cav2	12390	-0.054724388	0.67516536	0.80076102
ShSirt1_vs_ShScrambled	Q6A009	Q6A009	Ltn1	78913	0.138211309	0.67615139	0.80171983
ShSirt1_vs_ShScrambled	Q62348	Q62348	Tsn	22099	-0.150990196	0.67671142	0.80206479
ShSirt1_vs_ShScrambled	Q62018	Q62018	Ctr9	22083	0.088000083	0.67679814	0.80206479
ShSirt1_vs_ShScrambled	Q6PB66	Q6PB66	Lrpprc	72416	-0.064249961	0.67697538	0.80206479
ShSirt1_vs_ShScrambled	Q8K411	Q8K411	Pitrm1	69617	-0.068407709	0.67743969	0.80235102
ShSirt1_vs_ShScrambled	Q9D6R2	Q9D6R2	Idh3a	67834	-0.061583517	0.67757246	0.80235102
ShSirt1_vs_ShScrambled	Q8R1Q8	Q8R1Q8	Dync1li1	235661	-0.076953004	0.67831372	0.80301813
ShSirt1_vs_ShScrambled	E9Q4P1	E9Q4P1	Wdfy1	69368	-0.091400416	0.67862267	0.80317323
ShSirt1_vs_ShScrambled	Q99MN9	Q99MN9	Pccb	66904	0.090812442	0.67935952	0.80383457
ShSirt1_vs_ShScrambled	Q6P4S8	Q6P4S8	Ints1	68510	0.062440962	0.67974287	0.80398317
ShSirt1_vs_ShScrambled	Q99J36	Q99J36	Thumpd1	233802	-0.088353444	0.67984133	0.80398317
ShSirt1_vs_ShScrambled	Q924M7	Q924M7	Mpi	110119	-0.234583118	0.68044564	0.80448707
ShSirt1_vs_ShScrambled	Q6RHR9	Q6RHR9	Magi1	14924	-0.13512268	0.68104363	0.80487289
ShSirt1_vs_ShScrambled	Q8C0C7	Q8C0C7	Farsa	66590	0.093967425	0.68112859	0.80487289
ShSirt1_vs_ShScrambled	Q9QYF9	Q9QYF9	Ndrg3	29812	0.075269314	0.68199584	0.80568679
ShSirt1_vs_ShScrambled	Q8VDP2	Q8VDP2	Q8VDP2	77644	0.269754623	0.68263736	0.80623366
ShSirt1_vs_ShScrambled	Q922V4	Q922V4	Plrg1	53317	0.141133966	0.68286007	0.80624567
ShSirt1_vs_ShScrambled	Q06138	Q06138	Cab39	12283	-0.135629256	0.68312309	0.80624567
ShSirt1_vs_ShScrambled	Q8BVA5	Q8BVA5	Q8BVA5	68832	0.122509226	0.6832751	0.80624567
ShSirt1_vs_ShScrambled	Q9JMK0	Q9JMK0	B4galt5	56336	-0.064515751	0.68336197	0.80624567
ShSirt1_vs_ShScrambled	Q9D8T7	Q9D8T7	Slirp	380773	0.073389331	0.68439843	0.80702832
ShSirt1_vs_ShScrambled	Q61768	Q61768	Kif5b	16573	-0.063890548	0.68443513	0.80702832
ShSirt1_vs_ShScrambled	D3YYU8	D3YYU8	Obsl1	98733	0.058257708	0.68470125	0.80702832
ShSirt1_vs_ShScrambled	Q921V5	Q921V5	Mgat2	217664	-0.174971403	0.68481845	0.80702832
ShSirt1_vs_ShScrambled	Q99PM9	Q99PM9	Uck2	80914	0.148846816	0.68509665	0.80702832
ShSirt1_vs_ShScrambled	Q61510	Q61510	Trim25	217069	-0.119191262	0.68509803	0.80702832
ShSirt1_vs_ShScrambled	Q99LU0	Q99LU0;Q9C	Chmp1b1		0.226631762	0.68536147	0.80712802
ShSirt1_vs_ShScrambled	Q0KL02	Q0KL02	Trio	223435	0.266901945	0.68572777	0.80734876
ShSirt1_vs_ShScrambled	Q8VBV7	Q8VBV7	Cops8	108679	0.054511301	0.68594234	0.8073908
ShSirt1_vs_ShScrambled	O08848	O08848	Trove2	20822	0.084941645	0.68695993	0.80837777

ShSirt1_vs_ShScrambled	Q64092	Q64092	Tfe3	209446	0.218345594	0.68727595	0.80843355
ShSirt1_vs_ShScrambled	O08997	O08997	Atox1	11927	-0.110525899	0.68753	0.80843355
ShSirt1_vs_ShScrambled	Q32MW3	Q32MW3	Acot10	64833	-0.352923147	0.68767231	0.80843355
ShSirt1_vs_ShScrambled	Q7TNP2	Q7TNP2	Ppp2r1b	73699	-0.058183465	0.68772372	0.80843355
ShSirt1_vs_ShScrambled	Q8VHI3	Q8VHI3	Pofut2	80294	0.072392758	0.68889508	0.80959969
ShSirt1_vs_ShScrambled	P17156	P17156	Hspa2	15512	0.085083539	0.68964942	0.81027524
ShSirt1_vs_ShScrambled	Q7TPH6	Q7TPH6	Mycbp2	105689	-0.169725833	0.68992053	0.81038285
ShSirt1_vs_ShScrambled	Q62523	Q62523	Zyx	22793	-0.110750425	0.69083625	0.81124735
ShSirt1_vs_ShScrambled	P47911	P47911	Rpl6	19988	-0.058569972	0.69109946	0.81134537
ShSirt1_vs_ShScrambled	Q811B3	Q811B3	Adamts12	239337	-0.108343007	0.69145269	0.811549
ShSirt1_vs_ShScrambled	Q8BSY0	Q8BSY0	Asph	65973	-0.050208077	0.69184696	0.81180067
ShSirt1_vs_ShScrambled	Q5F2E7	Q5F2E7	Nufip2	68564	-0.132596092	0.69232337	0.81214857
ShSirt1_vs_ShScrambled	P51125	P51125	Cast	12380	0.078276828	0.69327923	0.81305858
ShSirt1_vs_ShScrambled	Q01405	Q01405	Sec23a	20334	0.063269883	0.69392885	0.81360904
ShSirt1_vs_ShScrambled	Q9JMD0	Q9JMD0	Znf207	22680	0.127125222	0.69494654	0.81439925
ShSirt1_vs_ShScrambled	P16546	P16546	Sptan1		-0.047856865	0.69503727	0.81439925
ShSirt1_vs_ShScrambled	A2AN08	A2AN08	Ubr4	69116	0.066991983	0.69514407	0.81439925
ShSirt1_vs_ShScrambled	Q9QZZ6	Q9QZZ6	Dpt	56429	0.169883606	0.69544902	0.81454512
ShSirt1_vs_ShScrambled	Q9Z1M8	Q9Z1M8	Ik	24010	0.256658372	0.69569383	0.81462047
ShSirt1_vs_ShScrambled	Q9JMH6	Q9JMH6	Txnrd1	50493	0.059195327	0.69671231	0.81560149
ShSirt1_vs_ShScrambled	Q9R1P4	Q9R1P4	Psma1	26440	-0.070204088	0.69767638	0.81651832
ShSirt1_vs_ShScrambled	Q8C3I8	Q8C3I8	Hgh1	59053	-0.111418976	0.69903048	0.81760015
ShSirt1_vs_ShScrambled	Q8WTY4	Q8WTY4	Ciapin1	109006	-0.157572746	0.69912838	0.81760015
ShSirt1_vs_ShScrambled	Q8BKR5	Q8BKR5	Ppp1r37	232947	0.286567068	0.69914413	0.81760015
ShSirt1_vs_ShScrambled	Q91WE1	Q91WE1	Snx15	69024	-0.066024112	0.70032076	0.81874408
ShSirt1_vs_ShScrambled	Q78JE5	Q78JE5	Fbxo22	71999	-0.148330659	0.70048507	0.81874408
ShSirt1_vs_ShScrambled	Q9CQ26	Q9CQ26	Stambp	70527	0.093620678	0.70073288	0.81882169
ShSirt1_vs_ShScrambled	Q61081	Q61081	Cdc37	12539	-0.055327709	0.70091959	0.81882791
ShSirt1_vs_ShScrambled	P13595	P13595	Ncam1	17967	-0.167398687	0.70211342	0.81988748
ShSirt1_vs_ShScrambled	P23949	P23949	Zfp36l2	12193	0.061204246	0.70218985	0.81988748
ShSirt1_vs_ShScrambled	Q60749	Q60749	Khdrbs1	20218	0.067338816	0.70331865	0.82099312
ShSirt1_vs_ShScrambled	Q9Z0H3	Q9Z0H3	Smarcb1	20587	-0.121461582	0.70350319	0.82099623
ShSirt1_vs_ShScrambled	Q6Q899	Q6Q899	Ddx58	230073	0.061962365	0.70387526	0.82115312
ShSirt1_vs_ShScrambled	Q9WVL6	Q9WVL6	Extl3		0.19186474	0.70400145	0.82115312
ShSirt1_vs_ShScrambled	Q8BGX2	Q8BGX2	Q8BGX2	69773	0.180660756	0.70448995	0.82149844
ShSirt1_vs_ShScrambled	Q9WU78	Q9WU78	Pdcd6ip	18571	-0.052946231	0.70466149	0.82149844
ShSirt1_vs_ShScrambled	Q8K0D5	Q8K0D5	Gfm1	28030	-0.048431669	0.70498479	0.82166314
ShSirt1_vs_ShScrambled	Q6P9S0	Q6P9S0	Mtss1l	244654	0.067983907	0.70588959	0.82201824
ShSirt1_vs_ShScrambled	Q9CQ06	Q9CQ06	Mrpl24	67707	-0.554173744	0.70595001	0.82201824
ShSirt1_vs_ShScrambled	Q8BK35	Q8BK35	Gltscr2	68077	-0.292033702	0.706039	0.82201824
ShSirt1_vs_ShScrambled	Q812A5	Q812A5	Prr5	109270	0.255634525	0.70617538	0.82201824
ShSirt1_vs_ShScrambled	Q8BYY4	Q8BYY4	Ttc39b	69863	0.607573391	0.7062274	0.82201824
ShSirt1_vs_ShScrambled	Q9R0E1	Q9R0E1	Plod3	26433	-0.054233499	0.70638209	0.82201824
ShSirt1_vs_ShScrambled	Q61646	Q61646	Hp	15439	0.276813923	0.70673404	0.82215566
ShSirt1_vs_ShScrambled	Q9QVP9	Q9QVP9	Ptk2b	19229	0.062231657	0.70686445	0.82215566
ShSirt1_vs_ShScrambled	Q9WV60	Q9WV60	Gsk3b	56637	-0.064186409	0.70855903	0.82372726
ShSirt1_vs_ShScrambled	Q5F285	Q5F285	Tmem256	69186	-0.121190451	0.70858063	0.82372726
ShSirt1_vs_ShScrambled	Q9WVA4	Q9WVA4	Tagln2	21346	-0.08508303	0.70910573	0.8239498
ShSirt1_vs_ShScrambled	P62274	P62274	Rps29	20090	-0.075091123	0.70913712	0.8239498
ShSirt1_vs_ShScrambled	Q03963	Q03963	Eif2ak2	19106	-0.077938685	0.70994227	0.82467303
ShSirt1_vs_ShScrambled	Q8C0D4	Q8C0D4	Arhgap12	75415	0.226557165	0.71046446	0.82503307
ShSirt1_vs_ShScrambled	O35943	O35943	Fxn	14297	0.112095067	0.71061776	0.82503307
ShSirt1_vs_ShScrambled	Q8BWT1	Q8BWT1	Acaa2	52538	0.055801757	0.71090989	0.82516
ShSirt1_vs_ShScrambled	Q99L47	Q99L47	St13	70356	-0.06425827	0.71161736	0.82564725
ShSirt1_vs_ShScrambled	Q8VBX6	Q8VBX6	Mpdz	17475	-0.174156492	0.71169549	0.82564725
ShSirt1_vs_ShScrambled	Q9JJ28	Q9JJ28	Flii	14248	-0.072677163	0.71191809	0.82569329
ShSirt1_vs_ShScrambled	Q810U5	Q810U5	Ccdc50	67501	-0.202507743	0.71262637	0.8261424
ShSirt1_vs_ShScrambled	Q8R2Y8	Q8R2Y8	Ptrh2	217057	0.059072999	0.71267135	0.8261424
ShSirt1_vs_ShScrambled	P0DOV2	P0DOV2	P0DOV2	15951	-0.08391226	0.71322887	0.82657642
ShSirt1_vs_ShScrambled	Q8R0P4	Q8R0P4	Aamdc	66273	-0.125584641	0.71360931	0.82680504
ShSirt1_vs_ShScrambled	Q9Z108	Q9Z108	Stau1	20853	-0.074499517	0.71395307	0.8268902
ShSirt1_vs_ShScrambled	Q8BGA9	Q8BGA9	Oxa1l	69089	0.156577052	0.71416836	0.8268902
ShSirt1_vs_ShScrambled	Q8R164	Q8R164	Bphl	68021	0.04682639	0.71429532	0.8268902

ShSirt1_vs_ShScrambled	Q80TM9	Q80TM9	Nisch	64652	0.0696003	0.71441555	0.8268902
ShSirt1_vs_ShScrambled	P18155	P18155	Mthfd2	17768	0.051861907	0.71553995	0.82797932
ShSirt1_vs_ShScrambled	Q9WUK2	Q9WUK2	Eif4h	22384	-0.080252011	0.7160092	0.82826886
ShSirt1_vs_ShScrambled	Q3U1J4	Q3U1J4	Ddb1	13194	0.068289932	0.71615715	0.82826886
ShSirt1_vs_ShScrambled	Q60649	Q60649	Clpb	20480	-0.117415884	0.71745535	0.82945731
ShSirt1_vs_ShScrambled	Q00422	Q00422	Gabpa	14390	-0.115851448	0.71755223	0.82945731
ShSirt1_vs_ShScrambled	P46664	P46664	Adss	11566	-0.055063873	0.71794426	0.82965628
ShSirt1_vs_ShScrambled	P70671	P70671	Irf3	54131	0.075469237	0.71827728	0.82965628
ShSirt1_vs_ShScrambled	Q9DB15	Q9DB15	Mrpl12	56282	-0.128761633	0.71835691	0.82965628
ShSirt1_vs_ShScrambled	Q8BQZ4	Q8BQZ4	Ralgapb		-0.21407121	0.71845955	0.82965628
ShSirt1_vs_ShScrambled	A6H8H2	A6H8H2	Dennd4c	329877	0.094063136	0.71903192	0.82976373
ShSirt1_vs_ShScrambled	Q60739	Q60739	Bag1	12017	-0.162892993	0.71908631	0.82976373
ShSirt1_vs_ShScrambled	Q60692	Q60692	Psmb6	19175	0.058092996	0.71910405	0.82976373
ShSirt1_vs_ShScrambled	Q99KN2	Q99KN2	Ciao1	26371	-0.148834595	0.71930204	0.82978007
ShSirt1_vs_ShScrambled	Q5IRJ6	Q5IRJ6	Slc30a9	109108	-0.110628636	0.71957472	0.82988254
ShSirt1_vs_ShScrambled	P35821	P35821	Ptpn1	19246	-0.057843688	0.72029282	0.83049854
ShSirt1_vs_ShScrambled	Q63932	Q63932	Map2k2	26396	0.111914618	0.72246592	0.83279141
ShSirt1_vs_ShScrambled	Q9WTX6	Q9WTX6	Cul1	26965	0.082438825	0.72297166	0.83316162
ShSirt1_vs_ShScrambled	Q9Z0M5	Q9Z0M5	Lipa	16889	0.293675209	0.72326667	0.83328886
ShSirt1_vs_ShScrambled	Q8BP67	Q8BP67	Rpl24	68193	-0.063657654	0.72345409	0.8332921
ShSirt1_vs_ShScrambled	O54916	O54916	Reps1	19707	0.108231049	0.72378412	0.83345957
ShSirt1_vs_ShScrambled	O08582	O08582	Gtpbp1	14904	0.095126369	0.72412773	0.83364259
ShSirt1_vs_ShScrambled	O08600	O08600	Endog	13804	0.139772124	0.72524639	0.83450959
ShSirt1_vs_ShScrambled	Q91ZA3	Q91ZA3	Pcca	110821	-0.045251059	0.72525058	0.83450959
ShSirt1_vs_ShScrambled	Q921L3	Q921L3	Tmco1	68944	-0.068923808	0.72610773	0.83490127
ShSirt1_vs_ShScrambled	Q9WUB0	Q9WUB0	Rbck1	24105	-0.44327885	0.726254	0.83490127
ShSirt1_vs_ShScrambled	Q9D1C8	Q9D1C8	Vps28	66914	-0.094294719	0.72649698	0.83490127
ShSirt1_vs_ShScrambled	P08228	P08228	Sod1	20655	0.175421038	0.7265179	0.83490127
ShSirt1_vs_ShScrambled	Q9DBY8	Q9DBY8	Nvl	67459	0.209070128	0.72665749	0.83490127
ShSirt1_vs_ShScrambled	Q91Z67	Q91Z67	Srgap2	14270	0.07626768	0.72676709	0.83490127
ShSirt1_vs_ShScrambled	P19258	P19258	Mpv17	17527	-0.116788574	0.72688569	0.83490127
ShSirt1_vs_ShScrambled	Q04750	Q04750	Top1	21969	0.052291979	0.72720029	0.83505015
ShSirt1_vs_ShScrambled	Q9JI10	Q9JI10	Stk3	56274	0.096224728	0.72768075	0.83538935
ShSirt1_vs_ShScrambled	Q9JMC3	Q9JMC3	Dnaja4	58233	0.144584964	0.72849125	0.83610717
ShSirt1_vs_ShScrambled	Q14AI6	Q14AI6	Rpusd3	101122	0.094716425	0.72915322	0.83665421
ShSirt1_vs_ShScrambled	Q9EQQ2	Q9EQQ2	Yipf5	67180	-0.074732528	0.73064106	0.83803406
ShSirt1_vs_ShScrambled	Q8CI75	Q8CI75	Dis3l2	208718	0.145551867	0.73072709	0.83803406
ShSirt1_vs_ShScrambled	A6H5Z3	A6H5Z3	Exoc6b	75914	-0.543319843	0.73136881	0.8382289
ShSirt1_vs_ShScrambled	Q8VEH5	Q8VEH5	Epm2aip1	77781	-0.08704992	0.73137818	0.8382289
ShSirt1_vs_ShScrambled	Q922Q4	Q922Q4	Pycr2	69051	0.071577644	0.73145406	0.8382289
ShSirt1_vs_ShScrambled	Q8CEC6	Q8CEC6	Ppwd1	238831	-0.084794018	0.73302097	0.83981134
ShSirt1_vs_ShScrambled	P47811	P47811	Mapk14	26416	0.167005881	0.73427323	0.84103257
ShSirt1_vs_ShScrambled	Q9ESY9	Q9ESY9	Ifi30	65972	0.222642885	0.73474871	0.8412621
ShSirt1_vs_ShScrambled	Q9CQA1	Q9CQA1	Trappc5	66682	-0.096473106	0.73490861	0.8412621
ShSirt1_vs_ShScrambled	Q9CWD3	Q9CWD3	Nudt17	78373	-0.162785192	0.73503273	0.8412621
ShSirt1_vs_ShScrambled	Q61387	Q61387	Cox7a2l	20463	-0.062508075	0.73632797	0.84236664
ShSirt1_vs_ShScrambled	P27808	P27808	Mgat1	17308	0.061376581	0.73637102	0.84236664
ShSirt1_vs_ShScrambled	Q9DBN4	Q9DBN4	P33monox	97820	-0.08897127	0.73716609	0.84306251
ShSirt1_vs_ShScrambled	P62900	P62900	Rpl31	114641	-0.04761766	0.73738854	0.84310331
ShSirt1_vs_ShScrambled	Q3V009	Q3V009	Tmed1	17083	-0.060465666	0.7378594	0.84342804
ShSirt1_vs_ShScrambled	Q9D0I4	Q9D0I4	Stx17	67727	0.119697223	0.73843945	0.84355459
ShSirt1_vs_ShScrambled	Q8BQZ5	Q8BQZ5	Cpsf4	54188	0.104630403	0.73846618	0.84355459
ShSirt1_vs_ShScrambled	Q9CPT4	Q9CPT4	Mydgf	28106	-0.071580027	0.73853073	0.84355459
ShSirt1_vs_ShScrambled	Q9Z0N1	Q9Z0N1;Q9Z	Eif2s3x		0.044185982	0.7387372	0.84357696
ShSirt1_vs_ShScrambled	Q8BH24	Q8BH24	Tm9sf4	99237	0.070505102	0.73897641	0.8436367
ShSirt1_vs_ShScrambled	Q9CXW4	Q9CXW4	Rpl11	67025	0.070828732	0.7393139	0.84375606
ShSirt1_vs_ShScrambled	P56546	P56546	Ctbp2	13017	0.067711577	0.7394548	0.84375606
ShSirt1_vs_ShScrambled	Q6ZQ93	Q6ZQ93	Usp34	17847	-0.242727622	0.74016528	0.84435332
ShSirt1_vs_ShScrambled	O55003	O55003	Bnip3	12176	-0.095479164	0.74056644	0.8445975
ShSirt1_vs_ShScrambled	Q9DCV4	Q9DCV4	Rmdn1	66302	0.067728393	0.74151924	0.84547054
ShSirt1_vs_ShScrambled	Q9D0P8	Q9D0P8	Ift27	67042	-0.072864025	0.74265151	0.8465477
ShSirt1_vs_ShScrambled	Q9DC28	Q9DC28	Csnk1d	104318	0.082196773	0.74298553	0.84671464
ShSirt1_vs_ShScrambled	P63242	P63242;Q8B	Eif5a		0.079609267	0.74393978	0.84758813

ShSirt1_vs_ShScrambled	Q9D0G0	Q9D0G0	Mrps30	59054	0.101762897	0.74504056	0.84862809
ShSirt1_vs_ShScrambled	O35657	O35657	Neu1	18010	-0.124312429	0.74560332	0.84905484
ShSirt1_vs_ShScrambled	Q9CZG3	Q9CZG3	Commd8	27784	0.262047273	0.74608824	0.84939277
ShSirt1_vs_ShScrambled	Q61624	Q61624	Znf148	22661	0.120748081	0.74655729	0.84971246
ShSirt1_vs_ShScrambled	Q91W90	Q91W90	Txndc5	105245	-0.053574531	0.74759674	0.85052745
ShSirt1_vs_ShScrambled	Q9R061	Q9R061	Nubp2	26426	0.196365933	0.74780059	0.85052745
ShSirt1_vs_ShScrambled	Q9Z2Y8	Q9Z2Y8	Prosc	114863	-0.098926606	0.7478386	0.85052745
ShSirt1_vs_ShScrambled	P09925	P09925	Surf1	20930	0.110957162	0.74823184	0.85076009
ShSirt1_vs_ShScrambled	Q3UM18	Q3UM18	Lsg1	224092	-0.069258463	0.7484412	0.85076009
ShSirt1_vs_ShScrambled	Q8K2C9	Q8K2C9	Hacd3	57874	-0.053333359	0.74860857	0.85076009
ShSirt1_vs_ShScrambled	P49443	P49443	Ppm1a	19042	0.085131999	0.74896925	0.85095575
ShSirt1_vs_ShScrambled	Q8JZY2	Q8JZY2	Commd10	69456	-0.052953162	0.74946638	0.85130631
ShSirt1_vs_ShScrambled	Q8K3A9	Q8K3A9	Mepce	231803	0.160166056	0.75115535	0.85284179
ShSirt1_vs_ShScrambled	Q8VC03	Q8VC03	Eml3	225898	0.121379665	0.75119605	0.85284179
ShSirt1_vs_ShScrambled	O09005	O09005	Degs1	13244	0.061786372	0.75156757	0.85304904
ShSirt1_vs_ShScrambled	Q9DBW3	Q9DBW3	Natd1	24083	-0.090071601	0.75207436	0.85340967
ShSirt1_vs_ShScrambled	P18872	P18872	Gnao1	14681	-0.039187672	0.75232352	0.85347785
ShSirt1_vs_ShScrambled	Q921M3	Q921M3	Sf3b3	101943	0.047557191	0.75281722	0.85382335
ShSirt1_vs_ShScrambled	Q9DCN2	Q9DCN2	Cyb5r3	109754	-0.046006208	0.75377351	0.85445695
ShSirt1_vs_ShScrambled	A2A8Z1	A2A8Z1	Osbpl9	100273	0.110800828	0.75388829	0.85445695
ShSirt1_vs_ShScrambled	Q60520	Q60520	Sin3a	20466	0.161453873	0.75394374	0.85445695
ShSirt1_vs_ShScrambled	O55022	O55022	Pgrmc1	53328	0.116933181	0.75457936	0.8546736
ShSirt1_vs_ShScrambled	Q3TDN2	Q3TDN2	Faf2	76577	-0.048695081	0.75458353	0.8546736
ShSirt1_vs_ShScrambled	Q9D2Z4	Q9D2Z4	Senp8	71599	-0.119579071	0.75470292	0.8546736
ShSirt1_vs_ShScrambled	Q99K23	Q99K23	Ufsp2	192169	-0.049518691	0.75559921	0.85529348
ShSirt1_vs_ShScrambled	Q8VDQ1	Q8VDQ1	Ptgr2	77219	-0.050128347	0.75562924	0.85529348
ShSirt1_vs_ShScrambled	Q9QYH6	Q9QYH6	Maged1	94275	0.101281101	0.7560618	0.8554413
ShSirt1_vs_ShScrambled	Q8VDW0	Q8VDW0	Ddx39a	68278	0.092453893	0.75624801	0.8554413
ShSirt1_vs_ShScrambled	Q8BI84	Q8BI84	Mia3	338366	-0.049769822	0.75632837	0.8554413
ShSirt1_vs_ShScrambled	Q9DBG6	Q9DBG6	Rpn2	20014	0.054009806	0.75672926	0.85568033
ShSirt1_vs_ShScrambled	Q8K2D3	Q8K2D3	Edc3	353190	-0.10066672	0.75760302	0.8564538
ShSirt1_vs_ShScrambled	Q8BGS1	Q8BGS1	Epb41l5	226352	-0.318788471	0.75798661	0.8566729
ShSirt1_vs_ShScrambled	P10711	P10711	Tcea1	21399	0.064431471	0.75848603	0.85698259
ShSirt1_vs_ShScrambled	P57776	P57776	Eef1d	66656	0.052216649	0.75864033	0.85698259
ShSirt1_vs_ShScrambled	Q8R087	Q8R087	B4galt7	218271	0.136262344	0.75931518	0.85737707
ShSirt1_vs_ShScrambled	Q9CPW9	Q9CPW9	Metap1d	66559	-0.316931953	0.75936941	0.85737707
ShSirt1_vs_ShScrambled	Q8R2U0	Q8R2U0	Seh1l	72124	0.039547148	0.75976625	0.85761062
ShSirt1_vs_ShScrambled	Q60631	Q60631	Grb2	14784	-0.065562716	0.76063039	0.85823626
ShSirt1_vs_ShScrambled	Q3TC46	Q3TC46	Patl1	225929	0.086726136	0.76070077	0.85823626
ShSirt1_vs_ShScrambled	Q3UKC1	Q3UKC1	Tax1bp1	52440	0.092908318	0.7612702	0.85866409
ShSirt1_vs_ShScrambled	Q8VE97	Q8VE97	Srsf4		0.125028022	0.76198438	0.85922767
ShSirt1_vs_ShScrambled	Q3U2P1	Q3U2P1	Sec24a	77371	0.05961653	0.76241516	0.85922767
ShSirt1_vs_ShScrambled	Q9JIW9	Q9JIW9	Ralb	64143	-0.073363533	0.76244373	0.85922767
ShSirt1_vs_ShScrambled	Q9JKB1	Q9JKB1;P58	Uchl3		0.054668696	0.76253124	0.85922767
ShSirt1_vs_ShScrambled	Q9DC33	Q9DC33	Hmg20a	66867	-0.115315177	0.7629553	0.85949094
ShSirt1_vs_ShScrambled	Q9D8U8	Q9D8U8	Snx5	69178	-0.098979548	0.76373789	0.8601579
ShSirt1_vs_ShScrambled	Q9JJL8	Q9JJL8	Sars2	71984	-0.243850003	0.76517936	0.86156638
ShSirt1_vs_ShScrambled	Q9Z2X2	Q9Z2X2	Psmd10	53380	0.143530199	0.76572394	0.86178384
ShSirt1_vs_ShScrambled	Q6PHQ8	Q6PHQ8	Naa35	78689	0.119968596	0.76575432	0.86178384
ShSirt1_vs_ShScrambled	Q8C4B4	Q8C4B4	Unc119b	106840	0.097864803	0.76610921	0.86196834
ShSirt1_vs_ShScrambled	Q5BL07	Q5BL07	Pex1	71382	0.079192969	0.76644636	0.86213279
ShSirt1_vs_ShScrambled	Q02780	Q02780	Nfia	18027	-0.05266132	0.76926304	0.86507521
ShSirt1_vs_ShScrambled	Q9D7B1	Q9D7B1	Dus2	66369	-0.170638675	0.76944549	0.86507521
ShSirt1_vs_ShScrambled	Q7TSG2	Q7TSG2	Ctdp1	67655	0.049331605	0.76997335	0.86545311
ShSirt1_vs_ShScrambled	Q3UJK4	Q3UJK4	Gtpbp2	56055	0.071808246	0.77160022	0.8670332
ShSirt1_vs_ShScrambled	Q99PM3	Q99PM3	Gtf2a1	83602	0.239129456	0.77179083	0.8670332
ShSirt1_vs_ShScrambled	Q9CY34	Q9CY34	Ube2f	67921	-0.100450545	0.77195534	0.8670332
ShSirt1_vs_ShScrambled	O55143	O55143	Atp2a2	11938	-0.046343328	0.77226875	0.86712413
ShSirt1_vs_ShScrambled	Q8CEC0	Q8CEC0	Nup88	19069	0.09189865	0.77256056	0.86712413
ShSirt1_vs_ShScrambled	B9EKI3	B9EKI3	Tmf1	232286	-0.067576872	0.7726126	0.86712413
ShSirt1_vs_ShScrambled	Q9CQF6	Q9CQF6	Aasdhppt	67618	-0.075113353	0.77297551	0.8673158
ShSirt1_vs_ShScrambled	Q5SSZ5	Q5SSZ5	Tns3	319939	-0.094815701	0.77442273	0.8687237
ShSirt1_vs_ShScrambled	Q99KJ8	Q99KJ8	Dctn2	69654	0.037903817	0.77489521	0.86903775

ShSirt1_vs_ShScrambled	Q9QWY8	Q9QWY8	Asap1	13196	0.095980475	0.77522907	0.86919623
ShSirt1_vs_ShScrambled	O70589	O70589	Cask	12361	0.055974341	0.77626788	0.87014482
ShSirt1_vs_ShScrambled	Q9CY16	Q9CY16	Mrps28	66230	0.14928774	0.77649523	0.87018358
ShSirt1_vs_ShScrambled	O54724	O54724	Ptrf	19285	0.04501429	0.77713052	0.87067936
ShSirt1_vs_ShScrambled	Q3TQI7	Q3TQI7	Q3TQI7	227707	-0.079527037	0.7776556	0.87090725
ShSirt1_vs_ShScrambled	Q9Z1T1	Q9Z1T1	Ap3b1	11774	0.040791511	0.77771979	0.87090725
ShSirt1_vs_ShScrambled	P62908	P62908	Rps3	27050	0.039670772	0.77852771	0.87159576
ShSirt1_vs_ShScrambled	Q9Z0S1	Q9Z0S1	Bpnt1	23827	-0.088062579	0.77887316	0.87176629
ShSirt1_vs_ShScrambled	Q9QZ88	Q9QZ88	Vps29	56433	0.050192313	0.77941417	0.87215557
ShSirt1_vs_ShScrambled	Q61792	Q61792	Lasp1	16796	-0.059854169	0.77961606	0.87216527
ShSirt1_vs_ShScrambled	Q3TZM9	Q3TZM9	Alg11	207958	0.098111389	0.77998124	0.87220538
ShSirt1_vs_ShScrambled	Q9D967	Q9D967	Mdp1	67881	-0.119278824	0.78037251	0.87220538
ShSirt1_vs_ShScrambled	Q8BP27	Q8BP27	Sfr1	67788	-0.091989911	0.78078323	0.87220538
ShSirt1_vs_ShScrambled	Q9WVL3	Q9WVL3	Slc12a7	20499	0.084440396	0.78079268	0.87220538
ShSirt1_vs_ShScrambled	A2AKB9	A2AKB9	Dcaf10	242418	-0.083607631	0.78080407	0.87220538
ShSirt1_vs_ShScrambled	Q9CUN6	Q9CUN6	Smurf1	75788	-0.150540993	0.78081124	0.87220538
ShSirt1_vs_ShScrambled	O35083	O35083	Agpat1	55979	-0.079093544	0.78387941	0.87541605
ShSirt1_vs_ShScrambled	Q4KWH5	Q4KWH5	Plch1	269437	-0.077778285	0.78451292	0.8758133
ShSirt1_vs_ShScrambled	Q76MZ3	Q76MZ3	Ppp2r1a	51792	0.040665762	0.78462317	0.8758133
ShSirt1_vs_ShScrambled	Q9JIA7	Q9JIA7	Sphk2	56632	0.076089728	0.78546802	0.87641568
ShSirt1_vs_ShScrambled	Q8BMG7	Q8BMG7	Rab3gap2	98732	0.045093298	0.78555114	0.87641568
ShSirt1_vs_ShScrambled	P26040	P26040	Ezr	22350	0.041651157	0.78595158	0.87664578
ShSirt1_vs_ShScrambled	Q5PRF0	Q5PRF0	Heatr5a	320487	-0.113373981	0.78646782	0.87700488
ShSirt1_vs_ShScrambled	Q8K245	Q8K245	Uvrag	78610	0.300718098	0.78673002	0.87708058
ShSirt1_vs_ShScrambled	Q9Z223	Q9Z223	Mocs2	17434	0.115869809	0.78718627	0.87720232
ShSirt1_vs_ShScrambled	Q6P3B9	Q6P3B9	Rbfa	68731	0.323110763	0.78722787	0.87720232
ShSirt1_vs_ShScrambled	P62702	P62702	Rps4x	20102	-0.038350702	0.78752085	0.87727795
ShSirt1_vs_ShScrambled	Q9Z1G3	Q9Z1G3	Atp6v1c1	66335	-0.043240725	0.78768443	0.87727795
ShSirt1_vs_ShScrambled	O70579	O70579	Slc25a17	20524	-0.040721681	0.78788049	0.87727986
ShSirt1_vs_ShScrambled	O70475	O70475	Ugdh	22235	0.039084931	0.78819864	0.87741767
ShSirt1_vs_ShScrambled	Q3UI43	Q3UI43	Babam1	68251	0.095553189	0.78877046	0.87769884
ShSirt1_vs_ShScrambled	O35381	O35381	Anp32a	11737	-0.085324415	0.78884009	0.87769884
ShSirt1_vs_ShScrambled	Q9CQ92	Q9CQ92	Fis1	66437	-0.058204213	0.79061318	0.87945488
ShSirt1_vs_ShScrambled	Q03173	Q03173	Enah	13800	-0.033131014	0.79096048	0.87948334
ShSirt1_vs_ShScrambled	Q8VC85	Q8VC85	Lsm1	67207	0.100998815	0.79102844	0.87948334
ShSirt1_vs_ShScrambled	Q8BKX6	Q8BKX6	Smg1	233789	0.04520238	0.79130194	0.87957079
ShSirt1_vs_ShScrambled	Q99LP6	Q99LP6	Grpel1	17713	0.06016857	0.79177371	0.87987852
ShSirt1_vs_ShScrambled	P31230	P31230	Aimp1		0.046779822	0.79268129	0.88027852
ShSirt1_vs_ShScrambled	P62264	P62264	Rps14	20044	-0.045896064	0.79288936	0.88027852
ShSirt1_vs_ShScrambled	Q9Z1R2	Q9Z1R2	Bag6	224727	0.041363896	0.7930208	0.88027852
ShSirt1_vs_ShScrambled	P54731	P54731	Faf1	14084	-0.039466048	0.79302395	0.88027852
ShSirt1_vs_ShScrambled	P62843	P62843	Rps15	20054	0.065256114	0.79310872	0.88027852
ShSirt1_vs_ShScrambled	P04627	P04627	Araf	11836	-0.033534266	0.79367389	0.88068926
ShSirt1_vs_ShScrambled	Q7TMB8	Q7TMB8	Cyfip1	20430	0.038660555	0.79400032	0.88069
ShSirt1_vs_ShScrambled	P18052	P18052	Ptpra	19262	0.531550954	0.79406476	0.88069
ShSirt1_vs_ShScrambled	Q9D820	Q9D820	Prorsd1	67939	-0.050151598	0.79502882	0.88154265
ShSirt1_vs_ShScrambled	Q91ZE0	Q91ZE0	Tmlhe	192289	-0.050414363	0.79562672	0.88198895
ShSirt1_vs_ShScrambled	Q8C0Z1	Q8C0Z1	Itfg3	106581	-0.134561699	0.7962166	0.88206701
ShSirt1_vs_ShScrambled	Q9Z1T6	Q9Z1T6	Pikfyve	18711	-0.109986373	0.79629108	0.88206701
ShSirt1_vs_ShScrambled	Q9ESJ4	Q9ESJ4	Nckipsd	80987	-0.342949075	0.79635992	0.88206701
ShSirt1_vs_ShScrambled	Q8R3F5	Q8R3F5	Mcat	223722	0.055919779	0.79651181	0.88206701
ShSirt1_vs_ShScrambled	C0HKD9	C0HKD9;C0H	C0HKD9		0.055416174	0.79667417	0.88206701
ShSirt1_vs_ShScrambled	Q3UH93	Q3UH93	Plxnd1	67784	-0.112670146	0.79701677	0.88215175
ShSirt1_vs_ShScrambled	Q9ER69	Q9ER69	Wtap	60532	0.097917022	0.79714156	0.88215175
ShSirt1_vs_ShScrambled	Q09143	Q09143	Slc7a1	11987	-0.290811259	0.79784186	0.88251944
ShSirt1_vs_ShScrambled	Q9DCA2	Q9DCA2	Mrps11		0.075446138	0.79786483	0.88251944
ShSirt1_vs_ShScrambled	Q8C7K6	Q8C7K6	Pcyox1l	240334	0.066394442	0.79833492	0.88282308
ShSirt1_vs_ShScrambled	Q63829	Q63829	Commd3	12238	0.196121442	0.79941322	0.88352641
ShSirt1_vs_ShScrambled	Q91ZS8	Q91ZS8	Adarb1	110532	-0.068075988	0.80005059	0.88352641
ShSirt1_vs_ShScrambled	Q6ZQH8	Q6ZQH8	Nup188	227699	0.167957887	0.80009136	0.88352641
ShSirt1_vs_ShScrambled	Q9D7M1	Q9D7M1	Gid8	76425	0.087376419	0.8000915	0.88352641
ShSirt1_vs_ShScrambled	Q8VE47	Q8VE47	Uba5	66663	0.051772284	0.80038176	0.88352641
ShSirt1_vs_ShScrambled	Q64727	Q64727	Vcl	22330	-0.031748216	0.80038181	0.88352641

ShSirt1_vs_ShScrambled	Q60931	Q60931	Vdac3	22335	-0.038320958	0.80044449	0.88352641
ShSirt1_vs_ShScrambled	Q99J45	Q99J45	Nrbp1	192292	0.067642939	0.80067829	0.88352641
ShSirt1_vs_ShScrambled	Q9WVB0	Q9WVB0	Rbpms	19663	-0.032598423	0.80073251	0.88352641
ShSirt1_vs_ShScrambled	Q99N95	Q99N95	Mrpl3	94062	0.057038758	0.80256085	0.88527389
ShSirt1_vs_ShScrambled	Q91ZX7	Q91ZX7	Lrp1	16971	0.034084243	0.80284337	0.88527389
ShSirt1_vs_ShScrambled	Q99JR8	Q99JR8	Smarcd2	83796	-0.042963133	0.80290459	0.88527389
ShSirt1_vs_ShScrambled	Q61127	Q61127	Nab2	17937	0.173918498	0.80338207	0.88554956
ShSirt1_vs_ShScrambled	Q61584	Q61584	Fxr1	14359	0.032088714	0.80373263	0.88554956
ShSirt1_vs_ShScrambled	Q9DBD5	Q9DBD5	Pelp1	75273	-0.046473405	0.80374315	0.88554956
ShSirt1_vs_ShScrambled	Q9D0D5	Q9D0D5	Gtf2e1	74197	-0.043075925	0.8048119	0.88651071
ShSirt1_vs_ShScrambled	Q923Q2	Q923Q2	Stard13	243362	0.108075126	0.80507308	0.88658206
ShSirt1_vs_ShScrambled	P84091	P84091	Ap2m1	11773	0.045189026	0.80543379	0.88665401
ShSirt1_vs_ShScrambled	Q9D5J6	Q9D5J6	Shpk	74637	0.09138399	0.80571625	0.88665401
ShSirt1_vs_ShScrambled	Q9JL62	Q9JL62	Gltp	56356	0.099948979	0.80573324	0.88665401
ShSirt1_vs_ShScrambled	P61089	P61089	Ube2n	93765	-0.053394337	0.8059241	0.88665401
ShSirt1_vs_ShScrambled	P70403	P70403;P535	Cux1		0.09505239	0.80677275	0.88737139
ShSirt1_vs_ShScrambled	O08917	O08917	Flot1	14251	-0.046158436	0.80748702	0.88794066
ShSirt1_vs_ShScrambled	Q8BGR9	Q8BGR9	Ublcp1	79560	0.050265749	0.80786055	0.8879547
ShSirt1_vs_ShScrambled	Q9D8E6	Q9D8E6	Rpl4	67891	-0.033022121	0.80789321	0.8879547
ShSirt1_vs_ShScrambled	Q8JZS9	Q8JZS9	Mrpl48	52443	-0.054953033	0.80845298	0.88835363
ShSirt1_vs_ShScrambled	P52912	P52912	Tia1	21841	-0.046048211	0.80916463	0.88891924
ShSirt1_vs_ShScrambled	O88845	O88845	Akap10	56697	0.240501366	0.80992503	0.88952847
ShSirt1_vs_ShScrambled	O35658	O35658	C1qbp		-0.072793101	0.81015285	0.88952847
ShSirt1_vs_ShScrambled	P46467	P46467	Vps4b	20479	-0.037551086	0.81065949	0.88952847
ShSirt1_vs_ShScrambled	Q9QUR7	Q9QUR7	Pin1	23988	-0.113205477	0.81074021	0.88952847
ShSirt1_vs_ShScrambled	Q9EQH2	Q9EQH2	Erap1	80898	0.043108467	0.81113828	0.88952847
ShSirt1_vs_ShScrambled	Q9R0P5	Q9R0P5	Dstn	56431	0.062110383	0.8113421	0.88952847
ShSirt1_vs_ShScrambled	Q922L6	Q922L6	Nelfcd	57314	0.085190553	0.81157671	0.88952847
ShSirt1_vs_ShScrambled	Q8BFW4	Q8BFW4	Trim65	338364	-0.119290558	0.8115911	0.88952847
ShSirt1_vs_ShScrambled	P54797	P54797	Tango2		-0.098306012	0.81164749	0.88952847
ShSirt1_vs_ShScrambled	Q80YS6	Q80YS6	Afap1	70292	-0.071514216	0.8117508	0.88952847
ShSirt1_vs_ShScrambled	O55126	O55126	Gbas		0.038685503	0.81188686	0.88952847
ShSirt1_vs_ShScrambled	Q149F1	Q149F1	Rpusd2	271842	-0.145940558	0.81234949	0.88974085
ShSirt1_vs_ShScrambled	Q9R1Q7	Q9R1Q7	Plp2	18824	-0.097134083	0.81249006	0.88974085
ShSirt1_vs_ShScrambled	A2AF47	A2AF47	Dock11	75974	0.085136502	0.81267202	0.88974085
ShSirt1_vs_ShScrambled	Q9CQA3	Q9CQA3	Sdhb	67680	0.036553163	0.81296334	0.88984397
ShSirt1_vs_ShScrambled	Q9JL35	Q9JL35	Hmgn5	50887	-0.068796553	0.81321507	0.88990371
ShSirt1_vs_ShScrambled	Q8VEK3	Q8VEK3	Hnrnpu	51810	0.04162165	0.81406918	0.89062246
ShSirt1_vs_ShScrambled	Q3TXS7	Q3TXS7	Psmd1	70247	0.032509241	0.8143233	0.8906846
ShSirt1_vs_ShScrambled	Q8BWD8	Q8BWD8	Cdk19	78334	-0.088921679	0.8151173	0.89122264
ShSirt1_vs_ShScrambled	Q9CWJ9	Q9CWJ9	Atic	108147	-0.031238105	0.81521008	0.89122264
ShSirt1_vs_ShScrambled	Q6PFR5	Q6PFR5	Tra2a		0.268076951	0.81581338	0.89161783
ShSirt1_vs_ShScrambled	Q9ESV0	Q9ESV0	Ddx24	27225	-0.054878932	0.81616914	0.89161783
ShSirt1_vs_ShScrambled	Q8R2M2	Q8R2M2	Dnttip2	99480	0.047484508	0.81624316	0.89161783
ShSirt1_vs_ShScrambled	P25444	P25444	Rps2	16898	-0.033808264	0.81641037	0.89161783
ShSirt1_vs_ShScrambled	P30280	P30280	Ccnd2	12444	-0.110231708	0.81655918	0.89161783
ShSirt1_vs_ShScrambled	Q9CR27	Q9CR27	Ccdc53	67282	0.099546788	0.81755569	0.89249006
ShSirt1_vs_ShScrambled	P56213	P56213	Gfer	11692	0.053818342	0.81816026	0.89268994
ShSirt1_vs_ShScrambled	Q8VI84	Q8VI84	Noc3l	57753	0.088926242	0.81823085	0.89268994
ShSirt1_vs_ShScrambled	P10639	P10639	Txn	22166	-0.061821119	0.81833207	0.89268994
ShSirt1_vs_ShScrambled	O88543	O88543	Cops3	26572	0.036868601	0.81855438	0.89271671
ShSirt1_vs_ShScrambled	Q9DCH4	Q9DCH4	Eif3f	66085	0.03589219	0.81891147	0.89289042
ShSirt1_vs_ShScrambled	P67984	P67984	Rpl22	19934	0.077637054	0.8194957	0.89331166
ShSirt1_vs_ShScrambled	Q9DC23	Q9DC23	Dnajc10	66861	0.050135878	0.82011445	0.89365328
ShSirt1_vs_ShScrambled	Q8BZA9	Q8BZA9	Tigar	319801	-0.078193137	0.82020504	0.89365328
ShSirt1_vs_ShScrambled	Q8BFR4	Q8BFR4	Gns	75612	0.031975412	0.8205845	0.89385097
ShSirt1_vs_ShScrambled	O88792	O88792	F11r	16456	0.086008506	0.82097927	0.89395664
ShSirt1_vs_ShScrambled	Q8K2W3	Q8K2W3	Txndc11	106200	-0.085839636	0.82131966	0.89395664
ShSirt1_vs_ShScrambled	Q8BUY9	Q8BUY9	Pggt1b	225467	0.085808659	0.82137553	0.89395664
ShSirt1_vs_ShScrambled	Q9JLJ2	Q9JLJ2	Aldh9a1	56752	0.047608996	0.82147367	0.89395664
ShSirt1_vs_ShScrambled	Q9CR62	Q9CR62	Slc25a11	67863	0.031695817	0.82207864	0.89439937
ShSirt1_vs_ShScrambled	Q64191	Q64191	Aga	11593	-0.124612686	0.82278338	0.8949504
ShSirt1_vs_ShScrambled	P97868	P97868	Rbbp6	19647	0.063382062	0.82353855	0.89546383

ShSirt1_vs_ShScrambled	Q499X9	Q499X9	Mars2	212679	-0.125998134	0.82366063	0.89546383
ShSirt1_vs_ShScrambled	Q80XJ3	Q80XJ3	Ttc28	209683	0.114512026	0.8239428	0.89546383
ShSirt1_vs_ShScrambled	Q3UGR5	Q3UGR5	Hdhd2	76987	-0.044768694	0.8240489	0.89546383
ShSirt1_vs_ShScrambled	Q9D0C4	Q9D0C4	Trmt5	76357	-0.033304126	0.82518432	0.89648184
ShSirt1_vs_ShScrambled	Q6WKZ8	Q6WKZ8	Ubr2	224826	0.047011909	0.82575579	0.89664768
ShSirt1_vs_ShScrambled	Q923T9	Q923T9	Camk2g	12325	0.094026545	0.82589703	0.89664768
ShSirt1_vs_ShScrambled	P31938	P31938	Map2k1	26395	0.049788732	0.82593289	0.89664768
ShSirt1_vs_ShScrambled	Q80ZM8	Q80ZM8	Crls1	66586	0.060354077	0.82638461	0.89692237
ShSirt1_vs_ShScrambled	Q8K2V6	Q8K2V6	Ipo11	76582	0.064420947	0.82716799	0.89737932
ShSirt1_vs_ShScrambled	Q6P5E6	Q6P5E6	Gga2	74105	-0.035741586	0.82720323	0.89737932
ShSirt1_vs_ShScrambled	Q3THE2	Q3THE2;Q9(	Myl12b		-0.070607501	0.82744176	0.89742242
ShSirt1_vs_ShScrambled	Q9JKK1	Q9JKK1	Stx6	58244	-0.048691999	0.82775889	0.89750479
ShSirt1_vs_ShScrambled	Q9CZX8	Q9CZX8	Rps19	20085	-0.036851475	0.82792275	0.89750479
ShSirt1_vs_ShScrambled	Q80V03	Q80V03	Adck5	268822	-0.074684281	0.82821757	0.89750479
ShSirt1_vs_ShScrambled	Q3UW53	Q3UW53	Fam129a	63913	0.043082277	0.82836792	0.89750479
ShSirt1_vs_ShScrambled	P34056	P34056	Tfap2a	21418	0.178872386	0.82851184	0.89750479
ShSirt1_vs_ShScrambled	P67778	P67778	Phb	18673	0.033551671	0.82891565	0.89772679
ShSirt1_vs_ShScrambled	Q9CYN9	Q9CYN9	Atp6ap2	70495	0.074364107	0.82944171	0.89808105
ShSirt1_vs_ShScrambled	O88522	O88522	Ikbkg	16151	0.106867253	0.83037923	0.89888054
ShSirt1_vs_ShScrambled	P47757	P47757	Capzb	12345	-0.035709529	0.8307614	0.89907862
ShSirt1_vs_ShScrambled	Q7TQH0	Q7TQH0	Atxn2l	233871	0.044921699	0.83136857	0.89952007
ShSirt1_vs_ShScrambled	Q9D2R8	Q9D2R8	Mrps33	14548	0.198887488	0.83197977	0.89996566
ShSirt1_vs_ShScrambled	Q03958	Q03958	Pfdn6	14976	0.122027873	0.83235479	0.90015561
ShSirt1_vs_ShScrambled	Q8VDV3	Q8VDV3	Rab3il1	74760	0.08003906	0.83304961	0.90069124
ShSirt1_vs_ShScrambled	Q8K2A1	Q8K2A1	Gulp1	70676	0.068292805	0.83375205	0.90123485
ShSirt1_vs_ShScrambled	O88569	O88569	Hnrnpa2b1	53379	-0.027484158	0.83434521	0.90148677
ShSirt1_vs_ShScrambled	Q8K224	Q8K224	Nat10	98956	0.052173416	0.83438452	0.90148677
ShSirt1_vs_ShScrambled	Q8R404	Q8R404	Qil1	224904	-0.042178225	0.83575736	0.90275394
ShSirt1_vs_ShScrambled	Q63844	Q63844	Mapk3	26417	0.05115282	0.83607661	0.90288273
ShSirt1_vs_ShScrambled	Q60680	Q60680	Chuk		0.114104802	0.83791287	0.90464929
ShSirt1_vs_ShScrambled	Q9QXB9	Q9QXB9	Drg2	13495	-0.030266775	0.83823641	0.9047822
ShSirt1_vs_ShScrambled	Q8BFZ9	Q8BFZ9	Erlin2	244373	0.056581664	0.83860925	0.90496824
ShSirt1_vs_ShScrambled	Q8R3C0	Q8R3C0	Mcmbp	210711	-0.033438342	0.83894131	0.9051102
ShSirt1_vs_ShScrambled	Q9CQA9	Q9CQA9	Ntpcr	66566	0.057438144	0.83916583	0.9051361
ShSirt1_vs_ShScrambled	Q62261	Q62261	Sptbn1	20742	0.0245852	0.84017767	0.90601099
ShSirt1_vs_ShScrambled	Q9Z1D1	Q9Z1D1	Eif3g	53356	-0.030225848	0.8405862	0.90623504
ShSirt1_vs_ShScrambled	P35285	P35285	Rab22a	19334	-0.040600408	0.84098337	0.90644674
ShSirt1_vs_ShScrambled	Q8C7V8	Q8C7V8	Ccdc134	76457	0.243929745	0.84146252	0.90661175
ShSirt1_vs_ShScrambled	Q91XD6	Q91XD6	Vps36	70160	-0.03203878	0.84153816	0.90661175
ShSirt1_vs_ShScrambled	Q9EPL4	Q9EPL4	Mettl9	59052	-0.092111694	0.84264124	0.90758353
ShSirt1_vs_ShScrambled	Q8VEK0	Q8VEK0	Tmem30a	69981	0.201405425	0.84305759	0.9077012
ShSirt1_vs_ShScrambled	Q9DBG3	Q9DBG3	Ap2b1	71770	0.038625816	0.84315267	0.9077012
ShSirt1_vs_ShScrambled	Q8VDG3	Q8VDG3	Parn	74108	0.121445191	0.84546189	0.90997019
ShSirt1_vs_ShScrambled	O35691	O35691	Pnn	18949	-0.03155108	0.84606117	0.91039812
ShSirt1_vs_ShScrambled	Q6ZPT1	Q6ZPT1	Klhl9	242521	0.073302188	0.84676108	0.91087238
ShSirt1_vs_ShScrambled	O88653	O88653	Lamtor3	56692	0.094881025	0.84690549	0.91087238
ShSirt1_vs_ShScrambled	Q5EG47	Q5EG47	Prkaa1	105787	-0.050047285	0.84807715	0.91191526
ShSirt1_vs_ShScrambled	P06537	P06537	Nr3c1		0.050163421	0.85062801	0.91444031
ShSirt1_vs_ShScrambled	Q8C1A5	Q8C1A5	Thop1	50492	-0.024547166	0.85126143	0.91490335
ShSirt1_vs_ShScrambled	O08919	O08919	Numbl	18223	0.054627755	0.85196907	0.9151496
ShSirt1_vs_ShScrambled	Q3URE1	Q3URE1	Acsf3	257633	0.048927855	0.85219664	0.9151496
ShSirt1_vs_ShScrambled	Q99KW3	Q99KW3	Triobp	110253	0.07329268	0.85227248	0.9151496
ShSirt1_vs_ShScrambled	Q9CX30	Q9CX30	Yif1b	77254	0.080366291	0.85230148	0.9151496
ShSirt1_vs_ShScrambled	Q68FD5	Q68FD5	Cltc	67300	-0.024789814	0.85282272	0.91527802
ShSirt1_vs_ShScrambled	P58771	P58771	Tpm1	22003	0.138269007	0.85282662	0.91527802
ShSirt1_vs_ShScrambled	Q3TMH2	Q3TMH2	Scrn3	74616	0.111986073	0.85379693	0.91610158
ShSirt1_vs_ShScrambled	Q8VIM9	Q8VIM9	Irgq	210146	-0.037126848	0.85435299	0.91648037
ShSirt1_vs_ShScrambled	Q9D892	Q9D892	Itpa	16434	0.022960548	0.85477296	0.91671303
ShSirt1_vs_ShScrambled	O35075	O35075	Dscr3	13185	0.053776634	0.85555465	0.91733342
ShSirt1_vs_ShScrambled	Q8BIK4	Q8BIK4	Dock9		0.044180693	0.85600917	0.91741462
ShSirt1_vs_ShScrambled	Q9EQQ9	Q9EQQ9	Mgea5	76055	0.065288141	0.85603686	0.91741462
ShSirt1_vs_ShScrambled	Q9CQ28	Q9CQ28	Dph6	66632	-0.048537469	0.85670333	0.91777846
ShSirt1_vs_ShScrambled	Q80WJ7	Q80WJ7	Mtdh	67154	-0.036632246	0.85678299	0.91777846

ShSirt1_vs_ShScrambled	Q61599	Q61599	Arhgdib	11857	0.028236764	0.85722465	0.91798443
ShSirt1_vs_ShScrambled	Q9WUA2	Q9WUA2	Farsb	23874	-0.021416907	0.857382	0.91798443
ShSirt1_vs_ShScrambled	Q6NVE9	Q6NVE9	Pptc7	320717	0.173174374	0.85824785	0.91869357
ShSirt1_vs_ShScrambled	P62827	P62827	Ran	19384	0.039138319	0.85861289	0.91877581
ShSirt1_vs_ShScrambled	Q5SSI6	Q5SSI6	Utp18	217109	0.037265155	0.85897282	0.91877581
ShSirt1_vs_ShScrambled	Q9DCT5	Q9DCT5	Sdf2	20316	-0.040004188	0.8590467	0.91877581
ShSirt1_vs_ShScrambled	Q5F2E8	Q5F2E8	Taok1	216965	0.033537323	0.859376	0.91877581
ShSirt1_vs_ShScrambled	P46938	P46938	Yap1	22601	0.039605228	0.85951633	0.91877581
ShSirt1_vs_ShScrambled	Q9Z1W9	Q9Z1W9	Stk39	53416	0.031713893	0.85971429	0.91877581
ShSirt1_vs_ShScrambled	Q9ERI5	Q9ERI5	Jmjd6	107817	-0.083417968	0.85974945	0.91877581
ShSirt1_vs_ShScrambled	P08752	P08752	Gnai2	14678	-0.024849313	0.86015809	0.91899494
ShSirt1_vs_ShScrambled	Q9CWX9	Q9CWX9	Ddx47	67755	0.052315231	0.8612529	0.91949177
ShSirt1_vs_ShScrambled	O54890	O54890	Itgb3	16416	-0.116914565	0.86130494	0.91949177
ShSirt1_vs_ShScrambled	Q7TSI3	Q7TSI3	Ppp6r1	243819	0.043900363	0.8614219	0.91949177
ShSirt1_vs_ShScrambled	Q6P3A8	Q6P3A8	Bckdhb	12040	-0.030225896	0.8614379	0.91949177
ShSirt1_vs_ShScrambled	O54833	O54833	Csnk2a2	13000	-0.038255375	0.86183729	0.91955319
ShSirt1_vs_ShScrambled	P24369	P24369	Ppib	19035	-0.030809914	0.86190287	0.91955319
ShSirt1_vs_ShScrambled	Q80UK8	Q80UK8	Ints2	70422	0.125146818	0.86216656	0.91961717
ShSirt1_vs_ShScrambled	Q8JZX4	Q8JZX4	Rbm17	76938	-0.092004834	0.86275856	0.92003122
ShSirt1_vs_ShScrambled	P61079	P61079	Ube2d3	66105	-0.037353002	0.86385214	0.92097101
ShSirt1_vs_ShScrambled	Q9CQU0	Q9CQU0	Txndc12	66073	0.030679881	0.86428437	0.92097101
ShSirt1_vs_ShScrambled	Q8R0H9	Q8R0H9	Gga1	106039	0.044591333	0.864608	0.92097101
ShSirt1_vs_ShScrambled	Q6PGL7	Q6PGL7	Fam21	28006	-0.025230885	0.8646773	0.92097101
ShSirt1_vs_ShScrambled	Q62448	Q62448	Eif4g2	13690	0.024175731	0.86501069	0.92097101
ShSirt1_vs_ShScrambled	O35435	O35435	Dhodh	56749	-0.026389252	0.86509438	0.92097101
ShSirt1_vs_ShScrambled	Q9CY28	Q9CY28	Gtpbp8	66067	-0.230812309	0.86511471	0.92097101
ShSirt1_vs_ShScrambled	P62331	P62331	Arf6	11845	0.071892515	0.86527206	0.92097101
ShSirt1_vs_ShScrambled	Q921E2	Q921E2	Rab31	106572	0.048427908	0.86549469	0.92099081
ShSirt1_vs_ShScrambled	A2APV2	A2APV2	Fmnl2	71409	-0.061126239	0.8658624	0.92116495
ShSirt1_vs_ShScrambled	Q62141	Q62141	Sin3b	20467	-0.059792839	0.86681912	0.92196548
ShSirt1_vs_ShScrambled	P10404	P10404	P10404		-0.024257405	0.86718099	0.9221331
ShSirt1_vs_ShScrambled	Q91W59	Q91W59	Rbms1	56878	0.098141423	0.86774915	0.92251994
ShSirt1_vs_ShScrambled	Q921N6	Q921N6	Ddx27	228889	0.035450194	0.8685878	0.9231941
ShSirt1_vs_ShScrambled	Q8R1F5	Q8R1F5	Hyi	68180	0.025150422	0.86890727	0.92331625
ShSirt1_vs_ShScrambled	O54988	O54988	Slk	20874	0.029074074	0.87006162	0.92432529
ShSirt1_vs_ShScrambled	Q9DBG5	Q9DBG5	Plin3	66905	0.022788815	0.87028255	0.92434246
ShSirt1_vs_ShScrambled	P16045	P16045	Lgals1	16852	0.028929041	0.87054562	0.92440435
ShSirt1_vs_ShScrambled	Q9D074	Q9D074	Mgrn1	17237	0.165628835	0.87091373	0.92443708
ShSirt1_vs_ShScrambled	O08808	O08808	Diaph1	13367	0.050064826	0.87098602	0.92443708
ShSirt1_vs_ShScrambled	B1AY13	B1AY13	Usp24	329908	-0.025292429	0.87179975	0.92505367
ShSirt1_vs_ShScrambled	Q80WG5	Q80WG5	Lrrc8a	241296	-0.161371278	0.87197682	0.92505367
ShSirt1_vs_ShScrambled	Q9DCD6	Q9DCD6	Gabarap	56486	0.054182045	0.87249697	0.925388
ShSirt1_vs_ShScrambled	Q9CR86	Q9CR86	Carhsp1	52502	0.138346151	0.87298903	0.92568822
ShSirt1_vs_ShScrambled	Q8BJU0	Q8BJU0	Sgta	52551	-0.026145834	0.87319018	0.92568822
ShSirt1_vs_ShScrambled	Q8BTV2	Q8BTV2	Cpsf7	269061	0.040400274	0.87433869	0.92652658
ShSirt1_vs_ShScrambled	P43275	P43275	Hist1h1a	80838	-0.059278697	0.87448015	0.92652658
ShSirt1_vs_ShScrambled	Q9D0J4	Q9D0J4	Arl2	56327	0.038550267	0.87459676	0.92652658
ShSirt1_vs_ShScrambled	P59326	P59326	Ythdf1	228994	-0.072875908	0.8754525	0.92721553
ShSirt1_vs_ShScrambled	Q8CCP0	Q8CCP0	Nemf	66244	0.042403347	0.87582933	0.92733542
ShSirt1_vs_ShScrambled	Q8K4Q0	Q8K4Q0	Rptor		0.033083547	0.87597656	0.92733542
ShSirt1_vs_ShScrambled	Q922H1	Q922H1	Prmt3	71974	-0.055868259	0.87630707	0.92746779
ShSirt1_vs_ShScrambled	Q99KR3	Q99KR3	Lactb2	212442	0.077817587	0.87847671	0.92939994
ShSirt1_vs_ShScrambled	P70168	P70168	Kpnb1	16211	0.023358713	0.87854443	0.92939994
ShSirt1_vs_ShScrambled	O88848	O88848	Arl6	56297	-0.113299838	0.8802172	0.93095137
ShSirt1_vs_ShScrambled	Q9Z1Q2	Q9Z1Q2	Abhd16a	193742	-0.019971703	0.88069363	0.93107558
ShSirt1_vs_ShScrambled	P62960	P62960	Ybx1	22608	-0.030482544	0.88109733	0.93107558
ShSirt1_vs_ShScrambled	Q9D8Y1	Q9D8Y1	Tmem126a	66271	0.067814358	0.88115687	0.93107558
ShSirt1_vs_ShScrambled	Q99JT9	Q99JT9	Adi1	104923	0.026431185	0.88115969	0.93107558
ShSirt1_vs_ShScrambled	P24638	P24638	Acp2	11432	-0.025694736	0.88169203	0.93142004
ShSirt1_vs_ShScrambled	P59481	P59481	Lman2l	214895	-0.163242188	0.88205565	0.93158615
ShSirt1_vs_ShScrambled	O88291	O88291	Znf326	54367	0.030586475	0.88288914	0.93224832
ShSirt1_vs_ShScrambled	Q61739	Q61739	Itga6	16403	-0.026286797	0.8843304	0.93355178
ShSirt1_vs_ShScrambled	P62301	P62301	Rps13	68052	0.022286885	0.88456715	0.93358338

ShSirt1_vs_ShScrambled	Q9WUL7	Q9WUL7	Arl3	56350	-0.040219176	0.88613036	0.9346889
ShSirt1_vs_ShScrambled	O88544	O88544	Cops4	26891	0.024183417	0.88625469	0.9346889
ShSirt1_vs_ShScrambled	Q6ZPZ3	Q6ZPZ3	Zc3h4	330474	-0.037634832	0.88642074	0.9346889
ShSirt1_vs_ShScrambled	Q6PD03	Q6PD03	Ppp2r5a	226849	-0.032380089	0.88663785	0.9346889
ShSirt1_vs_ShScrambled	Q9D8W5	Q9D8W5	Psmd12	66997	-0.035462386	0.88664995	0.9346889
ShSirt1_vs_ShScrambled	P11859	P11859	Agt		0.034399098	0.88687977	0.93471289
ShSirt1_vs_ShScrambled	Q8BVD5	Q8BVD5	Mpp7	75739	0.033046775	0.88711077	0.9347381
ShSirt1_vs_ShScrambled	Q9JIK9	Q9JIK9	Mrps34	79044	-0.040537743	0.88795788	0.93541233
ShSirt1_vs_ShScrambled	Q6PEE2	Q6PEE2	Ctif	269037	0.050725756	0.88939655	0.9364513
ShSirt1_vs_ShScrambled	Q6P1G0	Q6P1G0	Heatr6	217026	0.121542023	0.88945671	0.9364513
ShSirt1_vs_ShScrambled	Q8K5B2	Q8K5B2	Mcfd2	193813	-0.052386167	0.88985921	0.9364513
ShSirt1_vs_ShScrambled	Q6PB44	Q6PB44	Ptpn23	104831	-0.019274772	0.88997997	0.9364513
ShSirt1_vs_ShScrambled	Q91XC9	Q91XC9	Pex16	18633	0.023097261	0.89012389	0.9364513
ShSirt1_vs_ShScrambled	Q8CI08	Q8CI08	Slain2	75991	-0.041511076	0.89018886	0.9364513
ShSirt1_vs_ShScrambled	Q8BFZ3	Q8BFZ3	Actbl2	238880	-0.065411486	0.89118248	0.93727813
ShSirt1_vs_ShScrambled	Q8R480	Q8R480	Nup85	445007	0.21644877	0.89167025	0.93757268
ShSirt1_vs_ShScrambled	O35343	O35343	Kpna4	16649	-0.041050446	0.892158	0.93786708
ShSirt1_vs_ShScrambled	Q5XJY4	Q5XJY4	Parl	381038	-0.029391613	0.89238541	0.93788772
ShSirt1_vs_ShScrambled	Q9QXK7	Q9QXK7	Cpsf3	54451	0.034297143	0.89323052	0.93840538
ShSirt1_vs_ShScrambled	P47758	P47758	Srprb	20818	0.019364817	0.89329374	0.93840538
ShSirt1_vs_ShScrambled	P97429	P97429	Anxa4	11746	0.021015282	0.89402272	0.93895267
ShSirt1_vs_ShScrambled	Q8BHC9	Q8BHC9	Fut11	73068	0.04929484	0.8943307	0.93905764
ShSirt1_vs_ShScrambled	Q8C0L6	Q8C0L6	Paox	212503	0.04050603	0.89473496	0.93926363
ShSirt1_vs_ShScrambled	P0DP28	P0DP28;P0D	P0DP28		-0.046768536	0.89518818	0.93952091
ShSirt1_vs_ShScrambled	Q9Z0S9	Q9Z0S9	Rabac1	14470	-0.035488389	0.89561553	0.93966217
ShSirt1_vs_ShScrambled	Q80W47	Q80W47	Wipi2	74781	-0.030700144	0.89630026	0.93966217
ShSirt1_vs_ShScrambled	Q99KI0	Q99KI0	Aco2	11429	0.017110753	0.89656211	0.93966217
ShSirt1_vs_ShScrambled	Q8BGI4	Q8BGI4	Fam13a	58909	0.095916764	0.89671523	0.93966217
ShSirt1_vs_ShScrambled	Q9CZB0	Q9CZB0	Sdhc	66052	0.033144654	0.89677085	0.93966217
ShSirt1_vs_ShScrambled	Q9JI46	Q9JI46	Nudt3	56409	0.034911281	0.8967799	0.93966217
ShSirt1_vs_ShScrambled	Q148V7	Q148V7	Kiaa1468	227446	0.025301409	0.89696692	0.93966217
ShSirt1_vs_ShScrambled	P14206	P14206	Rpsa	16785	0.015931405	0.8971938	0.93966217
ShSirt1_vs_ShScrambled	P43883	P43883	Plin2	11520	-0.04398891	0.89719627	0.93966217
ShSirt1_vs_ShScrambled	O55229	O55229	Chkb	12651	-0.111769526	0.89814522	0.9403485
ShSirt1_vs_ShScrambled	Q71RI9	Q71RI9	Ccbl2	229905	-0.018547224	0.8986453	0.9403485
ShSirt1_vs_ShScrambled	P26369	P26369	U2af2	22185	0.022997209	0.89869724	0.9403485
ShSirt1_vs_ShScrambled	Q8VC30	Q8VC30	Dak	225913	-0.02324634	0.89879336	0.9403485
ShSirt1_vs_ShScrambled	Q9QZH6	Q9QZH6	Ecsit	26940	0.033562872	0.89889317	0.9403485
ShSirt1_vs_ShScrambled	Q7TMS5	Q7TMS5	Abcg2	26357	-0.031866034	0.89965193	0.94036512
ShSirt1_vs_ShScrambled	Q91X20	Q91X20	Ash2l	23808	0.082866994	0.8998141	0.94036512
ShSirt1_vs_ShScrambled	Q8BUE4	Q8BUE4	Aifm2	71361	0.02600287	0.89992646	0.94036512
ShSirt1_vs_ShScrambled	Q9D338	Q9D338	Mrpl19	56284	0.066305445	0.89997245	0.94036512
ShSirt1_vs_ShScrambled	Q3UFK8	Q3UFK8	Frmd8	67457	0.065313212	0.90012802	0.94036512
ShSirt1_vs_ShScrambled	Q99MK8	Q99MK8	Adrbk1	110355	0.043132158	0.90015899	0.94036512
ShSirt1_vs_ShScrambled	Q7TQK1	Q7TQK1	Ints7	77065	-0.064600971	0.90114427	0.9411766
ShSirt1_vs_ShScrambled	Q9CR98	Q9CR98	Fam136a	66488	-0.113160929	0.90135341	0.94117726
ShSirt1_vs_ShScrambled	Q8C5N3	Q8C5N3	Cwc22	80744	-0.098568611	0.9031091	0.94256674
ShSirt1_vs_ShScrambled	Q9DCA5	Q9DCA5	Brix1	67832	0.206231029	0.90328365	0.94256674
ShSirt1_vs_ShScrambled	P47740	P47740	Aldh3a2	11671	-0.032113601	0.90331053	0.94256674
ShSirt1_vs_ShScrambled	O54734	O54734	Ddost	13200	0.014674457	0.90423446	0.94295336
ShSirt1_vs_ShScrambled	Q9DC63	Q9DC63	Fbxo3	57443	0.03641988	0.9042679	0.94295336
ShSirt1_vs_ShScrambled	Q61409	Q61409	Pde3b	18576	-0.079183629	0.90445229	0.94295336
ShSirt1_vs_ShScrambled	Q9CPP6	Q9CPP6	Ndufa5	68202	-0.049866136	0.90451663	0.94295336
ShSirt1_vs_ShScrambled	Q9WTL7	Q9WTL7	Lypla2	26394	0.051495166	0.90486767	0.94310151
ShSirt1_vs_ShScrambled	Q99KK9	Q99KK9	Hars2	70791	-0.040599815	0.90583841	0.94371034
ShSirt1_vs_ShScrambled	Q99JR1	Q99JR1	Sfxn1	14057	0.014621592	0.90586994	0.94371034
ShSirt1_vs_ShScrambled	Q6PAQ4	Q6PAQ4	Rexo4	227656	-0.047358721	0.90726954	0.94478569
ShSirt1_vs_ShScrambled	P61971	P61971	Nutf2	68051	-0.021612488	0.90732077	0.94478569
ShSirt1_vs_ShScrambled	Q8QZT1	Q8QZT1	Acat1	110446	-0.014718368	0.90768626	0.94494829
ShSirt1_vs_ShScrambled	Q9D1F4	Q9D1F4	Akt1s1	67605	0.036896045	0.90808158	0.94514186
ShSirt1_vs_ShScrambled	Q9CQE8	Q9CQE8	Q9CQE8	68045	0.031567365	0.90834912	0.94520238
ShSirt1_vs_ShScrambled	Q8CIM3	Q8CIM3	D2hgdh	98314	0.024802546	0.90861269	0.94525874
ShSirt1_vs_ShScrambled	Q08288	Q08288	Lyar	17089	0.035528999	0.90952948	0.94599449

ShSirt1_vs_ShScrambled	Q9D906	Q9D906	Atg7	74244	-0.018943981	0.90989969	0.94616153
ShSirt1_vs_ShScrambled	P61957	P61957	Sumo2	170930	0.022819759	0.9102896	0.94634898
ShSirt1_vs_ShScrambled	P84244	P84244;P023	H3f3a		-0.025428904	0.91066609	0.94652238
ShSirt1_vs_ShScrambled	P62245	P62245	Rps15a	267019	-0.01676641	0.91149866	0.94716965
ShSirt1_vs_ShScrambled	Q3TLD5	Q3TLD5	Uri1	19777	0.043644596	0.91186881	0.94717136
ShSirt1_vs_ShScrambled	Q9ERU9	Q9ERU9	Ranbp2	19386	-0.018341176	0.91191997	0.94717136
ShSirt1_vs_ShScrambled	Q9D0Q7	Q9D0Q7	Mrpl45	67036	-0.040033959	0.913235	0.94814719
ShSirt1_vs_ShScrambled	P25799	P25799	Nfkb1	18033	-0.038432221	0.91327957	0.94814719
ShSirt1_vs_ShScrambled	Q9D938	Q9D938	Tmem160	69094	-0.050072053	0.91383614	0.94847367
ShSirt1_vs_ShScrambled	Q9D2V8	Q9D2V8	Mfsd10	68294	-0.015299586	0.91401428	0.94847367
ShSirt1_vs_ShScrambled	Q91WV0	Q91WV0	Dr1	13486	-0.035904067	0.9146254	0.94883735
ShSirt1_vs_ShScrambled	O88520	O88520	Shoc2	56392	-0.023778961	0.91478514	0.94883735
ShSirt1_vs_ShScrambled	Q6ZWV3	Q6ZWV3;P8	Rpl10		0.017578178	0.9150417	0.94888542
ShSirt1_vs_ShScrambled	Q62167	Q62167;P16	Ddx3x		0.013150157	0.91550282	0.94914555
ShSirt1_vs_ShScrambled	Q8BK72	Q8BK72	Mrps27	218506	-0.01812712	0.91710324	0.95058645
ShSirt1_vs_ShScrambled	P48428	P48428	Tbca	21371	0.021420239	0.91776331	0.95078502
ShSirt1_vs_ShScrambled	Q8CBY8	Q8CBY8	Dctn4	67665	0.01300528	0.91786528	0.95078502
ShSirt1_vs_ShScrambled	Q9ERG2	Q9ERG2	Strn3	94186	0.024333909	0.9179267	0.95078502
ShSirt1_vs_ShScrambled	P52480	P52480	Pkm	18746	0.01293602	0.91892091	0.95157353
ShSirt1_vs_ShScrambled	Q3U1V6	Q3U1V6	Uevld	54122	0.029479696	0.91910957	0.95157353
ShSirt1_vs_ShScrambled	Q9ESU6	Q9ESU6	Brd4	57261	0.01914091	0.91947808	0.95173677
ShSirt1_vs_ShScrambled	Q9JLZ3	Q9JLZ3	Auh	11992	-0.034091738	0.92010982	0.95217234
ShSirt1_vs_ShScrambled	O09061	O09061	Psmb1	19170	0.01298116	0.92128344	0.95282575
ShSirt1_vs_ShScrambled	Q9DBH5	Q9DBH5	Lman2	66890	-0.013832258	0.92130122	0.95282575
ShSirt1_vs_ShScrambled	Q3B7Z2	Q3B7Z2	Osbp	76303	0.020387369	0.92144587	0.95282575
ShSirt1_vs_ShScrambled	Q8R3B1	Q8R3B1	Plcd1	18799	-0.024062906	0.92167461	0.95282575
ShSirt1_vs_ShScrambled	Q6PF93	Q6PF93	Pik3c3	225326	-0.03269182	0.92179664	0.95282575
ShSirt1_vs_ShScrambled	Q80XC2	Q80XC2	Trmt61a	328162	-0.021900397	0.92244624	0.95327892
ShSirt1_vs_ShScrambled	P61924	P61924	Copz1	56447	-0.014843273	0.92384947	0.95431365
ShSirt1_vs_ShScrambled	Q9DC51	Q9DC51	Gnai3	14679	-0.014494903	0.92387032	0.95431365
ShSirt1_vs_ShScrambled	Q80XU3	Q80XU3	Nucks1	98415	-0.045879776	0.92468544	0.9549371
ShSirt1_vs_ShScrambled	Q91YN5	Q91YN5	Uap1	107652	0.029653983	0.92534837	0.95538414
ShSirt1_vs_ShScrambled	P10126	P10126	Eef1a1	13627	-0.011331059	0.92554161	0.95538414
ShSirt1_vs_ShScrambled	Q4VAA2	Q4VAA2	Cdv3	321022	-0.018221251	0.926251	0.95562178
ShSirt1_vs_ShScrambled	P06745	P06745	Gpi	14751	-0.012306289	0.92640037	0.95562178
ShSirt1_vs_ShScrambled	P70182	P70182	Pip5k1a	18720	0.024162871	0.92640694	0.95562178
ShSirt1_vs_ShScrambled	O88342	O88342	Wdr1	22388	0.012378256	0.92721018	0.95623183
ShSirt1_vs_ShScrambled	Q8CHW4	Q8CHW4	Eif2b5	224045	-0.015487417	0.92805223	0.95688163
ShSirt1_vs_ShScrambled	O35864	O35864	Cops5	26754	-0.011971055	0.92892382	0.95756157
ShSirt1_vs_ShScrambled	Q6PAM1	Q6PAM1	Txlna	109658	-0.015054432	0.93051078	0.95897846
ShSirt1_vs_ShScrambled	Q8BHE8	Q8BHE8	Q8BHE8	68115	-0.057543506	0.93152617	0.95980579
ShSirt1_vs_ShScrambled	P46460	P46460	Nsf	18195	-0.011875616	0.93201158	0.96008678
ShSirt1_vs_ShScrambled	Q5RJG1	Q5RJG1	Nol10	217431	0.056316328	0.93290467	0.96030037
ShSirt1_vs_ShScrambled	Q64127	Q64127	Trim24	21848	0.150606408	0.93296911	0.96030037
ShSirt1_vs_ShScrambled	Q9JI75	Q9JI75	Nqo2	18105	0.022397773	0.93305139	0.96030037
ShSirt1_vs_ShScrambled	Q9JKY0	Q9JKY0	Rqcd1	58184	0.03361158	0.93306988	0.96030037
ShSirt1_vs_ShScrambled	Q9Z266	Q9Z266	Snapin	20615	0.017773955	0.93337348	0.96039386
ShSirt1_vs_ShScrambled	P97377	P97377	Cdk2	12566	-0.033378913	0.93433858	0.96116781
ShSirt1_vs_ShScrambled	Q9JIX0	Q9JIX0	Eny2	223527	0.02581189	0.93571267	0.96235031
ShSirt1_vs_ShScrambled	Q8BKE6	Q8BKE6	Cyp20a1	77951	-0.024371976	0.93591446	0.96235031
ShSirt1_vs_ShScrambled	Q9D517	Q9D517	Agpat3	28169	-0.014503293	0.93641646	0.96255469
ShSirt1_vs_ShScrambled	D3YXK2	D3YXK2	Safb	224903	-0.014785994	0.9365397	0.96255469
ShSirt1_vs_ShScrambled	P35979	P35979	Rpl12	269261	-0.027534927	0.93679828	0.96260129
ShSirt1_vs_ShScrambled	Q2NL51	Q2NL51	Gsk3a	606496	0.044512703	0.93739248	0.96261775
ShSirt1_vs_ShScrambled	Q80Y55	Q80Y55	Bsdc1	100383	0.028526273	0.93744732	0.96261775
ShSirt1_vs_ShScrambled	P47713	P47713	Pla2g4a	18783	0.020524463	0.93752768	0.96261775
ShSirt1_vs_ShScrambled	Q9QZN4	Q9QZN4	Fbxo6	50762	0.024979997	0.93766731	0.96261775
ShSirt1_vs_ShScrambled	Q01279	Q01279	Egfr	13649	0.015122721	0.93909226	0.96386141
ShSirt1_vs_ShScrambled	Q8C3X2	Q8C3X2	Ccdc90b	66365	-0.018970137	0.93976777	0.96430171
ShSirt1_vs_ShScrambled	Q99L27	Q99L27	Gmpr2	105446	0.015724077	0.9399485	0.96430171
ShSirt1_vs_ShScrambled	Q9Z0H1	Q9Z0H1	Wdr46	57315	-0.032756245	0.94038445	0.96446286
ShSirt1_vs_ShScrambled	P70333	P70333	Hnrnph2	56258	-0.012586256	0.9405329	0.96446286
ShSirt1_vs_ShScrambled	A2AQ19	A2AQ19	Rtf1	76246	-0.021147607	0.94075692	0.96447348

ShSirt1_vs_ShScrambled	Q9CSH3	Q9CSH3	Dis3	72662	0.019442856	0.94100389	0.96450762
ShSirt1_vs_ShScrambled	Q9CSU0	Q9CSU0	Rprd1b	70470	-0.016444169	0.94122043	0.96451056
ShSirt1_vs_ShScrambled	Q8VDM4	Q8VDM4	Psmd2	21762	-0.009179499	0.94277727	0.96583441
ShSirt1_vs_ShScrambled	P21107	P21107	Tpm3	59069	-0.023076397	0.94314747	0.96583441
ShSirt1_vs_ShScrambled	Q8R1J9	Q8R1J9;P0C	Tor2a		0.012996389	0.94331128	0.96583441
ShSirt1_vs_ShScrambled	Q99MJ9	Q99MJ9	Ddx50	94213	0.032587198	0.94360056	0.96583441
ShSirt1_vs_ShScrambled	Q9CPR1	Q9CPR1	Rwdd4	192174	-0.017233777	0.94363276	0.96583441
ShSirt1_vs_ShScrambled	Q810B6	Q810B6	Ankfy1	11736	-0.010082436	0.9437961	0.96583441
ShSirt1_vs_ShScrambled	Q99N11	Q99N11	Dusp22	105352	-0.019369571	0.94438917	0.96622229
ShSirt1_vs_ShScrambled	Q9JI44	Q9JI44	Dmap1	66233	-0.026015915	0.94505344	0.96668281
ShSirt1_vs_ShScrambled	Q9JLC8	Q9JLC8	Sacs	50720	-0.016700437	0.94576755	0.96719409
ShSirt1_vs_ShScrambled	P32921	P32921	Wars	22375	-0.010667134	0.94614793	0.96724278
ShSirt1_vs_ShScrambled	Q08943	Q08943	Ssrp1	20833	0.019723283	0.94624371	0.96724278
ShSirt1_vs_ShScrambled	Q0GNC1	Q0GNC1	Inf2	70435	0.015871764	0.94687569	0.96766966
ShSirt1_vs_ShScrambled	P84078	P84078;P612	Arf1		-0.016931885	0.94821395	0.96881796
ShSirt1_vs_ShScrambled	Q9D2R6	Q9D2R6	Coa3	52469	0.014529776	0.94890866	0.96930837
ShSirt1_vs_ShScrambled	Q9CXK9	Q9CXK9	Rbm33	381626	0.015360022	0.94939223	0.96958293
ShSirt1_vs_ShScrambled	Q80YX1	Q80YX1	Tnc	21923	-0.011908776	0.95032676	0.9703178
ShSirt1_vs_ShScrambled	Q9CR70	Q9CR70	Lage3	66192	0.009911947	0.95058964	0.97036672
ShSirt1_vs_ShScrambled	Q9CQR2	Q9CQR2	Rps21	66481	-0.016538252	0.95339849	0.97301397
ShSirt1_vs_ShScrambled	Q9Z0U1	Q9Z0U1	Tjp2	21873	0.010278924	0.95422397	0.9736363
ShSirt1_vs_ShScrambled	Q9JHR7	Q9JHR7	Ide		0.008251044	0.95452852	0.97372695
ShSirt1_vs_ShScrambled	E9Q7E2	E9Q7E2	Arid2	77044	0.011724417	0.95539289	0.9743885
ShSirt1_vs_ShScrambled	Q9EPL9	Q9EPL9	Acox3	80911	-0.006836219	0.95581043	0.97446384
ShSirt1_vs_ShScrambled	Q640M1	Q640M1	Utp14a	72554	0.012428418	0.95589852	0.97446384
ShSirt1_vs_ShScrambled	P61211	P61211	Arl1	104303	0.014057836	0.95656031	0.97491832
ShSirt1_vs_ShScrambled	Q99LI8	Q99LI8	Hgs	15239	-0.009949251	0.95686546	0.97500918
ShSirt1_vs_ShScrambled	O70591	O70591	Pfdn2	18637	0.015751068	0.95725885	0.97518049
ShSirt1_vs_ShScrambled	Q80Y56	Q80Y56	Rbsn	78287	0.034248522	0.95761179	0.97518049
ShSirt1_vs_ShScrambled	Q80XI3	Q80XI3	Eif4g3	230861	0.054585201	0.95768168	0.97518049
ShSirt1_vs_ShScrambled	Q8R0W0	Q8R0W0	Eppk1	223650	0.023388028	0.95843417	0.97572662
ShSirt1_vs_ShScrambled	Q9ER39	Q9ER39	Tor1a	30931	0.053596295	0.95903381	0.97593029
ShSirt1_vs_ShScrambled	P08113	P08113	Hsp90b1	22027	0.007306311	0.95906663	0.97593029
ShSirt1_vs_ShScrambled	Q80XI4	Q80XI4	Pip4k2b	108083	-0.021489846	0.96010133	0.97623007
ShSirt1_vs_ShScrambled	P60605	P60605	Ube2g2	22213	0.026632813	0.96014732	0.97623007
ShSirt1_vs_ShScrambled	Q9CYR0	Q9CYR0	Ssbp1	381760	-0.009828321	0.96037289	0.97623007
ShSirt1_vs_ShScrambled	Q8K4B0	Q8K4B0	Mta1		0.015701901	0.96059709	0.97623007
ShSirt1_vs_ShScrambled	Q9Z1J3	Q9Z1J3	Nfs1	18041	0.008953329	0.96061129	0.97623007
ShSirt1_vs_ShScrambled	P53994	P53994	Rab2a	59021	0.006476044	0.96076103	0.97623007
ShSirt1_vs_ShScrambled	O55236	O55236	Rngtt	24018	0.029665096	0.9608751	0.97623007
ShSirt1_vs_ShScrambled	Q08122	Q08122	Tle3	21887	0.028763429	0.96110264	0.97624152
ShSirt1_vs_ShScrambled	Q8C6G8	Q8C6G8	Wdr26	226757	-0.020181989	0.96179349	0.97672347
ShSirt1_vs_ShScrambled	O89079	O89079	Cope	59042	0.010644539	0.9622582	0.9769756
ShSirt1_vs_ShScrambled	Q7TMY7	Q7TMY7	Ipo8	320727	-0.059553532	0.962542	0.97704398
ShSirt1_vs_ShScrambled	Q8C0M0	Q8C0M0	Wdr59	319481	0.018591204	0.96355191	0.97784921
ShSirt1_vs_ShScrambled	Q8BP47	Q8BP47	Nars	70223	0.007377531	0.96504413	0.97914345
ShSirt1_vs_ShScrambled	Q9DC70	Q9DC70	Ndufs7	75406	-0.015432356	0.9669817	0.98079513
ShSirt1_vs_ShScrambled	Q9JI90	Q9JI90	Rnf14	56736	-0.090309804	0.96732215	0.98079513
ShSirt1_vs_ShScrambled	Q62077	Q62077	Plcg1	18803	-0.007527939	0.96732386	0.98079513
ShSirt1_vs_ShScrambled	P00329	P00329	Adh1	11522	0.008801689	0.96755139	0.98080552
ShSirt1_vs_ShScrambled	Q91W86	Q91W86	Vps11	71732	-0.007770132	0.96875507	0.98180521
ShSirt1_vs_ShScrambled	Q80XK6	Q80XK6	Atg2b	76559	-0.021751276	0.97000158	0.98263368
ShSirt1_vs_ShScrambled	Q4FZC9	Q4FZC9	Syne3	212073	-0.010062012	0.9700079	0.98263368
ShSirt1_vs_ShScrambled	Q3TZX3	Q3TZX3;Q92	Slc25a33		0.007668358	0.97048213	0.9828935
ShSirt1_vs_ShScrambled	Q9Z2H5	Q9Z2H5	Epb41l1	13821	-0.005915965	0.970742	0.98293616
ShSirt1_vs_ShScrambled	Q8BXJ9	Q8BXJ9	Tmem62	96957	0.013121607	0.97098141	0.98295809
ShSirt1_vs_ShScrambled	Q3UMU9	Q3UMU9	Hdgfrp2	15193	0.008600216	0.97139654	0.98315785
ShSirt1_vs_ShScrambled	Q8BFQ4	Q8BFQ4	Wdr82	77305	0.019218434	0.97175211	0.98329725
ShSirt1_vs_ShScrambled	Q8R5A3	Q8R5A3	Apbb1ip	54519	0.008728688	0.97206856	0.98331934
ShSirt1_vs_ShScrambled	Q9D666	Q9D666	Sun1	77053	-0.007617503	0.97220962	0.98331934
ShSirt1_vs_ShScrambled	Q9ESN9	Q9ESN9	Mapk8ip3	30957	0.054606184	0.97306193	0.9837562
ShSirt1_vs_ShScrambled	Q8N7N5	Q8N7N5	Dcaf8	98193	0.006055538	0.97326793	0.9837562
ShSirt1_vs_ShScrambled	P61290	P61290	Psme3	19192	-0.005037885	0.97329535	0.9837562

ShSirt1_vs_ShScrambled	Q91Z49	Q91Z49	Fyttd1	69823	0.004620065	0.97487848	0.98474044
ShSirt1_vs_ShScrambled	Q9D0F9	Q9D0F9	Pgm1	72157	0.005133148	0.9750593	0.98474044
ShSirt1_vs_ShScrambled	P63017	P63017	Hspa8	15481	0.004360833	0.97522576	0.98474044
ShSirt1_vs_ShScrambled	P59325	P59325	Eif5	217869	0.004953182	0.97527561	0.98474044
ShSirt1_vs_ShScrambled	Q3TMX7	Q3TMX7	Qsox2	227638	-0.0121583	0.97535988	0.98474044
ShSirt1_vs_ShScrambled	Q8R5H1	Q8R5H1	Usp15	14479	0.010978811	0.97588525	0.98505054
ShSirt1_vs_ShScrambled	Q8R3S6	Q8R3S6	Exoc1	69940	0.012467328	0.97616499	0.98511263
ShSirt1_vs_ShScrambled	Q8CIN4	Q8CIN4	Pak2	224105	-0.00875511	0.97649133	0.9852217
ShSirt1_vs_ShScrambled	Q8K0Z7	Q8K0Z7	Taco1	70207	-0.010705948	0.97692264	0.98543661
ShSirt1_vs_ShScrambled	Q9EPK2	Q9EPK2	Rp2	19889	-0.006750756	0.97739676	0.98569459
ShSirt1_vs_ShScrambled	Q99LD9	Q99LD9	Eif2b2	217715	-0.009777694	0.97780669	0.98588774
ShSirt1_vs_ShScrambled	P45377	P45377	Akr1b8	14187	-0.004386491	0.97973448	0.98761086
ShSirt1_vs_ShScrambled	O54774	O54774	Ap3d1	11776	0.004808729	0.98031251	0.98785862
ShSirt1_vs_ShScrambled	P62196	P62196	Psmc5	19184	-0.003255364	0.98041795	0.98785862
ShSirt1_vs_ShScrambled	P05213	P05213	Tuba1b	22143	-0.003366111	0.98199974	0.98903827
ShSirt1_vs_ShScrambled	Q9DBR3	Q9DBR3	Armc8	74125	-0.013393639	0.98233265	0.98903827
ShSirt1_vs_ShScrambled	Q9Z2D6	Q9Z2D6	Mecp2	17257	0.00876659	0.9824498	0.98903827
ShSirt1_vs_ShScrambled	Q3UYV9	Q3UYV9	Ncbp1	433702	-0.01196043	0.98246513	0.98903827
ShSirt1_vs_ShScrambled	P62852	P62852	Rps25	75617	0.003640133	0.98363067	0.98999083
ShSirt1_vs_ShScrambled	Q99PG2	Q99PG2	Ogfr	72075	0.007745358	0.98391584	0.99005709
ShSirt1_vs_ShScrambled	Q3U0K8	Q3U0K8	Ogfod1	270086	0.007299503	0.98465115	0.99057617
ShSirt1_vs_ShScrambled	P47963	P47963	Rpl13	270106	0.002468192	0.98508229	0.99078909
ShSirt1_vs_ShScrambled	Q78IK4	Q78IK4	Apool	68117	-0.003377566	0.98560679	0.9910958
ShSirt1_vs_ShScrambled	O89001	O89001	Cpd	12874	0.003009582	0.98583894	0.99110846
ShSirt1_vs_ShScrambled	P32067	P32067	Ssb	20823	0.002552171	0.98618517	0.99123577
ShSirt1_vs_ShScrambled	Q8CHP8	Q8CHP8	Pgp	67078	-0.002674047	0.98650861	0.99134013
ShSirt1_vs_ShScrambled	A2AGH6	A2AGH6	Med12	59024	0.006118955	0.9870583	0.99167174
ShSirt1_vs_ShScrambled	Q8K2Y7	Q8K2Y7	Mrpl47	74600	-0.004506168	0.98776517	0.9921611
ShSirt1_vs_ShScrambled	Q9D0M5	Q9D0M5	Dynll2	68097	0.003840782	0.98812716	0.99227249
ShSirt1_vs_ShScrambled	Q8BJF9	Q8BJF9	Chmp2b	68942	-0.005603123	0.98831571	0.99227249
ShSirt1_vs_ShScrambled	Q9QYS9	Q9QYS9	Qki	19317	0.002195222	0.98917958	0.99291897
ShSirt1_vs_ShScrambled	Q91XL3	Q91XL3	Uxs1	67883	0.003189132	0.99074679	0.99427101
ShSirt1_vs_ShScrambled	Q924T2	Q924T2	Mrps2	118451	0.001927208	0.99110045	0.99440485
ShSirt1_vs_ShScrambled	P60229	P60229	Eif3e	16341	-0.00113275	0.9925702	0.9956582
ShSirt1_vs_ShScrambled	Q9DB27	Q9DB27	Mcts1	68995	-0.001724782	0.99304011	0.99590826
ShSirt1_vs_ShScrambled	Q9R0P4	Q9R0P4	Smap	56372	-0.003205064	0.99332401	0.99597169
ShSirt1_vs_ShScrambled	Q9EQG9	Q9EQG9	Col4a3bp	68018	-0.001141803	0.99416972	0.99659829
ShSirt1_vs_ShScrambled	O54784	O54784	Dapk3	13144	-0.002631939	0.99443302	0.99664091
ShSirt1_vs_ShScrambled	Q9CQ65	Q9CQ65	Mtap	66902	0.00105394	0.99470347	0.99669067
ShSirt1_vs_ShScrambled	P99026	P99026	Psmb4	19172	0.00099952	0.99531174	0.99694221
ShSirt1_vs_ShScrambled	E9PZJ8	E9PZJ8	Ascc3	77987	-0.001037679	0.99554286	0.99694221
ShSirt1_vs_ShScrambled	P43406	P43406	Itgav	16410	-0.000774215	0.99571459	0.99694221
ShSirt1_vs_ShScrambled	P39447	P39447	Tjp1	21872	0.001755417	0.99583793	0.99694221
ShSirt1_vs_ShScrambled	A2RSJ4	A2RSJ4	Uhrf1bp1l	75089	-0.001749863	0.99650283	0.99738664
ShSirt1_vs_ShScrambled	P61087	P61087	Ube2k	53323	-0.000601592	0.99712955	0.99779269
ShSirt1_vs_ShScrambled	P46061	P46061	Rangap1	19387	-0.000420416	0.99852185	0.99896446
ShSirt1_vs_ShScrambled	Q9CRB9	Q9CRB9	Chchd3	66075	0.000198093	0.99935327	0.9995747
ShSirt1_vs_ShScrambled	P97765	P97765	Wbp2	22378	-3.61276E-05	0.99996957	0.99996957
ShSirt1_vs_ShScrambled	A2A6A1	A2A6A1	Gpatch8	237943	#N/A	#N/A	#N/A
ShSirt1_vs_ShScrambled	A2A791	A2A791	Zmym4	67785	#N/A	#N/A	#N/A
ShSirt1_vs_ShScrambled	A2A8U2	A2A8U2	Tmem201	230917	#N/A	#N/A	#N/A
ShSirt1_vs_ShScrambled	A2A9C3	A2A9C3	Szt2	230676	#N/A	#N/A	#N/A
ShSirt1_vs_ShScrambled	A2ADA5	A2ADA5	Pusl1	433813	#N/A	#N/A	#N/A
ShSirt1_vs_ShScrambled	A2AG50	A2AG50	Map7d2	78283	#N/A	#N/A	#N/A
ShSirt1_vs_ShScrambled	A2AJ88	A2AJ88	Pnpla7	241274	#N/A	#N/A	#N/A
ShSirt1_vs_ShScrambled	A3KFM7	A3KFM7	Chd6	71389	#N/A	#N/A	#N/A
ShSirt1_vs_ShScrambled	A6H6A9	A6H6A9	Rabgap1l	29809	#N/A	#N/A	#N/A
ShSirt1_vs_ShScrambled	A6X8Z5	A6X8Z5	Arhgap31	12549	#N/A	#N/A	#N/A
ShSirt1_vs_ShScrambled	A6X919	A6X919	Dpy19l1	244745	#N/A	#N/A	#N/A
ShSirt1_vs_ShScrambled	B1AR13	B1AR13	Cisd3	217149	#N/A	#N/A	#N/A
ShSirt1_vs_ShScrambled	B1AUE5	B1AUE5	Pex10	668173	#N/A	#N/A	#N/A
ShSirt1_vs_ShScrambled	B1AVH7	B1AVH7	Tbc1d2	381605	#N/A	#N/A	#N/A
ShSirt1_vs_ShScrambled	B1AVY7	B1AVY7	Kif16b	16558	#N/A	#N/A	#N/A

ShSirt1_vs_ShScrambled	B2RRD7	B2RRD7	Brpf1	78783	#N/A	#N/A	#N/A
ShSirt1_vs_ShScrambled	B8ZXI1	B8ZXI1	Qtrtd1	106248	#N/A	#N/A	#N/A
ShSirt1_vs_ShScrambled	B9EJ80	B9EJ80	Pdzd8	107368	#N/A	#N/A	#N/A
ShSirt1_vs_ShScrambled	D3YZG8	D3YZG8	Mthfd2l	665563	#N/A	#N/A	#N/A
ShSirt1_vs_ShScrambled	E2JF22	E2JF22	Piezo1		#N/A	#N/A	#N/A
ShSirt1_vs_ShScrambled	E9PXF8	E9PXF8	Sbf2	319934	#N/A	#N/A	#N/A
ShSirt1_vs_ShScrambled	E9PYL2	E9PYL2	Prr12	233210	#N/A	#N/A	#N/A
ShSirt1_vs_ShScrambled	E9Q309	E9Q309	Cep350	74081	#N/A	#N/A	#N/A
ShSirt1_vs_ShScrambled	E9Q4F7	E9Q4F7	Ankrd11	77087	#N/A	#N/A	#N/A
ShSirt1_vs_ShScrambled	E9Q4N7	E9Q4N7	Arid1b	239985	#N/A	#N/A	#N/A
ShSirt1_vs_ShScrambled	E9Q634	E9Q634	Myo1e	71602	#N/A	#N/A	#N/A
ShSirt1_vs_ShScrambled	E9Q6P5	E9Q6P5	Ttc7b	104718	#N/A	#N/A	#N/A
ShSirt1_vs_ShScrambled	E9Q7D5	E9Q7D5	Arhgef5	54324	#N/A	#N/A	#N/A
ShSirt1_vs_ShScrambled	E9QAM5	E9QAM5	Helz2	229003	#N/A	#N/A	#N/A
ShSirt1_vs_ShScrambled	O08740	O08740	Polr2j		#N/A	#N/A	#N/A
ShSirt1_vs_ShScrambled	O35047	O35047	Psmc3ip	19183	#N/A	#N/A	#N/A
ShSirt1_vs_ShScrambled	O35071	O35071	Kif1c	16562	#N/A	#N/A	#N/A
ShSirt1_vs_ShScrambled	O35153	O35153	Bet1l	54399	#N/A	#N/A	#N/A
ShSirt1_vs_ShScrambled	O35218	O35218	Cpsf2	51786	#N/A	#N/A	#N/A
ShSirt1_vs_ShScrambled	O35309	O35309	Nmi	64685	#N/A	#N/A	#N/A
ShSirt1_vs_ShScrambled	O35409	O35409	Folh1	53320	#N/A	#N/A	#N/A
ShSirt1_vs_ShScrambled	O35492	O35492	Clk3	102414	#N/A	#N/A	#N/A
ShSirt1_vs_ShScrambled	O35594	O35594	Ift81	12589	#N/A	#N/A	#N/A
ShSirt1_vs_ShScrambled	O35654	O35654	Pold2	18972	#N/A	#N/A	#N/A
ShSirt1_vs_ShScrambled	O35963	O35963	Rab33b	19338	#N/A	#N/A	#N/A
ShSirt1_vs_ShScrambled	O35973	O35973	Per1	18626	#N/A	#N/A	#N/A
ShSirt1_vs_ShScrambled	O35984	O35984	Pbx2	18515	#N/A	#N/A	#N/A
ShSirt1_vs_ShScrambled	O54965	O54965	Rnf13	24017	#N/A	#N/A	#N/A
ShSirt1_vs_ShScrambled	O55057	O55057	Pde6d	18582	#N/A	#N/A	#N/A
ShSirt1_vs_ShScrambled	O55102	O55102	Bloc1s1	14533	#N/A	#N/A	#N/A
ShSirt1_vs_ShScrambled	O55187	O55187	Cbx4	12418	#N/A	#N/A	#N/A
ShSirt1_vs_ShScrambled	O70309	O70309	Itgb5	16419	#N/A	#N/A	#N/A
ShSirt1_vs_ShScrambled	O70348	O70348	Dxo	112403	#N/A	#N/A	#N/A
ShSirt1_vs_ShScrambled	O70405	O70405	Ulk1	22241	#N/A	#N/A	#N/A
ShSirt1_vs_ShScrambled	O70422	O70422	Gtf2h4	14885	#N/A	#N/A	#N/A
ShSirt1_vs_ShScrambled	O70583	O70583	Mid1	17318	#N/A	#N/A	#N/A
ShSirt1_vs_ShScrambled	O88448	O88448	Klc2		#N/A	#N/A	#N/A
ShSirt1_vs_ShScrambled	O88643	O88643	Pak1		#N/A	#N/A	#N/A
ShSirt1_vs_ShScrambled	O88697	O88697	Stk16	20872	#N/A	#N/A	#N/A
ShSirt1_vs_ShScrambled	O88876	O88876	Dhrs3	20148	#N/A	#N/A	#N/A
ShSirt1_vs_ShScrambled	O88878	O88878	Zfand5	22682	#N/A	#N/A	#N/A
ShSirt1_vs_ShScrambled	O88974	O88974	Setdb1		#N/A	#N/A	#N/A
ShSirt1_vs_ShScrambled	P01942	P01942	Hba		#N/A	#N/A	#N/A
ShSirt1_vs_ShScrambled	P03975	P03975	Iap		#N/A	#N/A	#N/A
ShSirt1_vs_ShScrambled	P04925	P04925	Prnp	19122	#N/A	#N/A	#N/A
ShSirt1_vs_ShScrambled	P06802	P06802	Enpp1	18605	#N/A	#N/A	#N/A
ShSirt1_vs_ShScrambled	P08042	P08042	Zfp1	22640	#N/A	#N/A	#N/A
ShSirt1_vs_ShScrambled	P09242	P09242	Alpl	11647	#N/A	#N/A	#N/A
ShSirt1_vs_ShScrambled	P0C0A3	P0C0A3	Chmp6	208092	#N/A	#N/A	#N/A
ShSirt1_vs_ShScrambled	P0DM40	P0DM40	Fer1l5	1.01E+08	#N/A	#N/A	#N/A
ShSirt1_vs_ShScrambled	P10076	P10076;Q8R	Zfp26		#N/A	#N/A	#N/A
ShSirt1_vs_ShScrambled	P11440	P11440	Cdk1	12534	#N/A	#N/A	#N/A
ShSirt1_vs_ShScrambled	P12023	P12023	App	11820	#N/A	#N/A	#N/A
ShSirt1_vs_ShScrambled	P13864	P13864	Dnmt1	13433	#N/A	#N/A	#N/A
ShSirt1_vs_ShScrambled	P15535	P15535	B4galt1	14595	#N/A	#N/A	#N/A
ShSirt1_vs_ShScrambled	P19426	P19426	Nelfe	27632	#N/A	#N/A	#N/A
ShSirt1_vs_ShScrambled	P20444	P20444	Prkca	18750	#N/A	#N/A	#N/A
ShSirt1_vs_ShScrambled	P23463	P23463;P090	Hoxd8		#N/A	#N/A	#N/A
ShSirt1_vs_ShScrambled	P23607	P23607;P170	Zfa		#N/A	#N/A	#N/A
ShSirt1_vs_ShScrambled	P28028	P28028	Braf	109880	#N/A	#N/A	#N/A
ShSirt1_vs_ShScrambled	P28033	P28033	Cebpb	12608	#N/A	#N/A	#N/A
ShSirt1_vs_ShScrambled	P28301	P28301	Lox	16948	#N/A	#N/A	#N/A
ShSirt1_vs_ShScrambled	P28481	P28481	Col2a1	12824	#N/A	#N/A	#N/A

ShSirt1_vs_ShScrambled	P28654	P28654	Dcn	13179	#N/A	#N/A	#N/A
ShSirt1_vs_ShScrambled	P28667	P28667	Marcksl1	17357	#N/A	#N/A	#N/A
ShSirt1_vs_ShScrambled	P30282	P30282	Ccnd3	12445	#N/A	#N/A	#N/A
ShSirt1_vs_ShScrambled	P33215	P33215	Nedd1	17997	#N/A	#N/A	#N/A
ShSirt1_vs_ShScrambled	P33434	P33434	Mmp2	17390	#N/A	#N/A	#N/A
ShSirt1_vs_ShScrambled	P35822	P35822	Ptprk	19272	#N/A	#N/A	#N/A
ShSirt1_vs_ShScrambled	P35951	P35951	Ldlr	16835	#N/A	#N/A	#N/A
ShSirt1_vs_ShScrambled	P37913	P37913	Lig1	16881	#N/A	#N/A	#N/A
ShSirt1_vs_ShScrambled	P38532	P38532	Hsf1	15499	#N/A	#N/A	#N/A
ShSirt1_vs_ShScrambled	P38585	P38585	Ttl	69737	#N/A	#N/A	#N/A
ShSirt1_vs_ShScrambled	P42703	P42703	Lifr	16880	#N/A	#N/A	#N/A
ShSirt1_vs_ShScrambled	P43135	P43135;Q60(	Nr2f2		#N/A	#N/A	#N/A
ShSirt1_vs_ShScrambled	P45481	P45481	Crebbp		#N/A	#N/A	#N/A
ShSirt1_vs_ShScrambled	P46062	P46062	Sipa1		#N/A	#N/A	#N/A
ShSirt1_vs_ShScrambled	P46656	P46656	Fdx1	14148	#N/A	#N/A	#N/A
ShSirt1_vs_ShScrambled	P48725	P48725	Pcnt	18541	#N/A	#N/A	#N/A
ShSirt1_vs_ShScrambled	P51141	P51141	Dvl1	13542	#N/A	#N/A	#N/A
ShSirt1_vs_ShScrambled	P51954	P51954	Nek1	18004	#N/A	#N/A	#N/A
ShSirt1_vs_ShScrambled	P52792	P52792	Gck	103988	#N/A	#N/A	#N/A
ShSirt1_vs_ShScrambled	P53566	P53566	Cebpa	12606	#N/A	#N/A	#N/A
ShSirt1_vs_ShScrambled	P53762	P53762	Arnt	11863	#N/A	#N/A	#N/A
ShSirt1_vs_ShScrambled	P56198	P56198	Cidec	14311	#N/A	#N/A	#N/A
ShSirt1_vs_ShScrambled	P56371	P56371	Rab4a	19341	#N/A	#N/A	#N/A
ShSirt1_vs_ShScrambled	P56392	P56392	Cox7a1	12865	#N/A	#N/A	#N/A
ShSirt1_vs_ShScrambled	P56581	P56581	Tdg	21665	#N/A	#N/A	#N/A
ShSirt1_vs_ShScrambled	P57080	P57080	Usp25	30940	#N/A	#N/A	#N/A
ShSirt1_vs_ShScrambled	P59048	P59048	Pdrg1	68559	#N/A	#N/A	#N/A
ShSirt1_vs_ShScrambled	P59114	P59114	Pcif1	228866	#N/A	#N/A	#N/A
ShSirt1_vs_ShScrambled	P59438	P59438	Hps5	246694	#N/A	#N/A	#N/A
ShSirt1_vs_ShScrambled	P59729	P59729	Rin3	217835	#N/A	#N/A	#N/A
ShSirt1_vs_ShScrambled	P59764	P59764	Dock4	238130	#N/A	#N/A	#N/A
ShSirt1_vs_ShScrambled	P59913	P59913	Pcmtd1	319263	#N/A	#N/A	#N/A
ShSirt1_vs_ShScrambled	P60060	P60060	Sec61g	20335	#N/A	#N/A	#N/A
ShSirt1_vs_ShScrambled	P60840	P60840	Ensa	56205	#N/A	#N/A	#N/A
ShSirt1_vs_ShScrambled	P61226	P61226	Rap2b	74012	#N/A	#N/A	#N/A
ShSirt1_vs_ShScrambled	P61294	P61294	Rab6b	270192	#N/A	#N/A	#N/A
ShSirt1_vs_ShScrambled	P61406	P61406	Smg6	103677	#N/A	#N/A	#N/A
ShSirt1_vs_ShScrambled	P62254	P62254	Ube2g1	67128	#N/A	#N/A	#N/A
ShSirt1_vs_ShScrambled	P62484	P62484	Abi2	329165	#N/A	#N/A	#N/A
ShSirt1_vs_ShScrambled	P62878	P62878	Rbx1	56438	#N/A	#N/A	#N/A
ShSirt1_vs_ShScrambled	P63013	P63013	Prrx1	18933	#N/A	#N/A	#N/A
ShSirt1_vs_ShScrambled	P63213	P63213	Gng2	14702	#N/A	#N/A	#N/A
ShSirt1_vs_ShScrambled	P68373	P68373	Tuba1c	22146	#N/A	#N/A	#N/A
ShSirt1_vs_ShScrambled	P69566	P69566	Ranbp9	56705	#N/A	#N/A	#N/A
ShSirt1_vs_ShScrambled	P70121	P70121	Zhx1	22770	#N/A	#N/A	#N/A
ShSirt1_vs_ShScrambled	P70196	P70196	Traf6	22034	#N/A	#N/A	#N/A
ShSirt1_vs_ShScrambled	P70236	P70236	Map2k6	26399	#N/A	#N/A	#N/A
ShSirt1_vs_ShScrambled	P70255	P70255	Nfic	18029	#N/A	#N/A	#N/A
ShSirt1_vs_ShScrambled	P70340	P70340;P974	Smad1		#N/A	#N/A	#N/A
ShSirt1_vs_ShScrambled	P70353	P70353	Nfyc	18046	#N/A	#N/A	#N/A
ShSirt1_vs_ShScrambled	P70426	P70426	Rit1	19769	#N/A	#N/A	#N/A
ShSirt1_vs_ShScrambled	P70428	P70428	Ext2	14043	#N/A	#N/A	#N/A
ShSirt1_vs_ShScrambled	P70429	P70429	Evl	14026	#N/A	#N/A	#N/A
ShSirt1_vs_ShScrambled	P81269	P81269	Atf1	11908	#N/A	#N/A	#N/A
ShSirt1_vs_ShScrambled	P82198	P82198	Tgfbi	21810	#N/A	#N/A	#N/A
ShSirt1_vs_ShScrambled	P82347	P82347	Sgcd	24052	#N/A	#N/A	#N/A
ShSirt1_vs_ShScrambled	P82349	P82349	Sgcb	24051	#N/A	#N/A	#N/A
ShSirt1_vs_ShScrambled	P83877	P83877	Txnl4a	27366	#N/A	#N/A	#N/A
ShSirt1_vs_ShScrambled	P97313	P97313	Prkdc	19090	#N/A	#N/A	#N/A
ShSirt1_vs_ShScrambled	P97360	P97360	Etv6	14011	#N/A	#N/A	#N/A
ShSirt1_vs_ShScrambled	P97366	P97366	Evi5		#N/A	#N/A	#N/A
ShSirt1_vs_ShScrambled	P97432	P97432	Nbr1	17966	#N/A	#N/A	#N/A
ShSirt1_vs_ShScrambled	P97460	P97460;O08	Npas2		#N/A	#N/A	#N/A

ShSirt1_vs_ShScrambled	P97465	P97465	Dok1	13448	#N/A	#N/A	#N/A
ShSirt1_vs_ShScrambled	P97471	P97471	Smad4	17128	#N/A	#N/A	#N/A
ShSirt1_vs_ShScrambled	P97479	P97479	Myo7a	17921	#N/A	#N/A	#N/A
ShSirt1_vs_ShScrambled	P97480	P97480	Eya3	14050	#N/A	#N/A	#N/A
ShSirt1_vs_ShScrambled	P97493	P97493	Txn2	56551	#N/A	#N/A	#N/A
ShSirt1_vs_ShScrambled	P97760	P97760	Polr2c	20021	#N/A	#N/A	#N/A
ShSirt1_vs_ShScrambled	P97798	P97798	Neo1		#N/A	#N/A	#N/A
ShSirt1_vs_ShScrambled	P97857	P97857	Adamts1	11504	#N/A	#N/A	#N/A
ShSirt1_vs_ShScrambled	P97863	P97863	Nfib	18028	#N/A	#N/A	#N/A
ShSirt1_vs_ShScrambled	P97865	P97865	Pex7	18634	#N/A	#N/A	#N/A
ShSirt1_vs_ShScrambled	P98078	P98078	Dab2	13132	#N/A	#N/A	#N/A
ShSirt1_vs_ShScrambled	Q00262	Q00262	Stx2		#N/A	#N/A	#N/A
ShSirt1_vs_ShScrambled	Q00322	Q00322	Cebpd	12609	#N/A	#N/A	#N/A
ShSirt1_vs_ShScrambled	Q00560	Q00560	Il6st	16195	#N/A	#N/A	#N/A
ShSirt1_vs_ShScrambled	Q00899	Q00899	Yy1	22632	#N/A	#N/A	#N/A
ShSirt1_vs_ShScrambled	Q01063	Q01063	Pde4d	238871	#N/A	#N/A	#N/A
ShSirt1_vs_ShScrambled	Q02257	Q02257	Jup	16480	#N/A	#N/A	#N/A
ShSirt1_vs_ShScrambled	Q03385	Q03385;Q61	Ralgds		#N/A	#N/A	#N/A
ShSirt1_vs_ShScrambled	Q05421	Q05421	Cyp2e1	13106	#N/A	#N/A	#N/A
ShSirt1_vs_ShScrambled	Q05BC3	Q05BC3	Eml1	68519	#N/A	#N/A	#N/A
ShSirt1_vs_ShScrambled	Q06180	Q06180	Ptpn2	19255	#N/A	#N/A	#N/A
ShSirt1_vs_ShScrambled	Q08509	Q08509	Eps8	13860	#N/A	#N/A	#N/A
ShSirt1_vs_ShScrambled	Q08775	Q08775	Runx2	12393	#N/A	#N/A	#N/A
ShSirt1_vs_ShScrambled	Q09163	Q09163	Dlk1	13386	#N/A	#N/A	#N/A
ShSirt1_vs_ShScrambled	Q0P678	Q0P678	Zc3h18	76014	#N/A	#N/A	#N/A
ShSirt1_vs_ShScrambled	Q11204	Q11204	St3gal2	20444	#N/A	#N/A	#N/A
ShSirt1_vs_ShScrambled	Q148V8	Q148V8	Fam83h	105732	#N/A	#N/A	#N/A
ShSirt1_vs_ShScrambled	Q149L6	Q149L6	Dnajb14	70604	#N/A	#N/A	#N/A
ShSirt1_vs_ShScrambled	Q14AX6	Q14AX6	Cdk12	69131	#N/A	#N/A	#N/A
ShSirt1_vs_ShScrambled	Q2YDW7	Q2YDW7	Setd8	67956	#N/A	#N/A	#N/A
ShSirt1_vs_ShScrambled	Q33DR3	Q33DR3	Pdss2	71365	#N/A	#N/A	#N/A
ShSirt1_vs_ShScrambled	Q3KNM2	Q3KNM2	March5	69104	#N/A	#N/A	#N/A
ShSirt1_vs_ShScrambled	Q3TAA7	Q3TAA7	Stk11ip	71728	#N/A	#N/A	#N/A
ShSirt1_vs_ShScrambled	Q3TAS6	Q3TAS6	Emc10	69683	#N/A	#N/A	#N/A
ShSirt1_vs_ShScrambled	Q3TGW2	Q3TGW2	Eepd1	67484	#N/A	#N/A	#N/A
ShSirt1_vs_ShScrambled	Q3TH73	Q3TH73	Ttyh2	117160	#N/A	#N/A	#N/A
ShSirt1_vs_ShScrambled	Q3THF9	Q3THF9	Coq10b	67876	#N/A	#N/A	#N/A
ShSirt1_vs_ShScrambled	Q3THJ3	Q3THJ3	Eif1ad	69860	#N/A	#N/A	#N/A
ShSirt1_vs_ShScrambled	Q3TKY6	Q3TKY6	Cwc27	67285	#N/A	#N/A	#N/A
ShSirt1_vs_ShScrambled	Q3TLI0	Q3TLI0	Trappc10		#N/A	#N/A	#N/A
ShSirt1_vs_ShScrambled	Q3TNH5	Q3TNH5	Fam172a	68675	#N/A	#N/A	#N/A
ShSirt1_vs_ShScrambled	Q3TQB2	Q3TQB2	Foxred1	235169	#N/A	#N/A	#N/A
ShSirt1_vs_ShScrambled	Q3TWF6	Q3TWF6	Wdr70		#N/A	#N/A	#N/A
ShSirt1_vs_ShScrambled	Q3TX08	Q3TX08	Trmt1	212528	#N/A	#N/A	#N/A
ShSirt1_vs_ShScrambled	Q3U0M1	Q3U0M1	Trappc9	76510	#N/A	#N/A	#N/A
ShSirt1_vs_ShScrambled	Q3U3I9	Q3U3I9	Znf865	319748	#N/A	#N/A	#N/A
ShSirt1_vs_ShScrambled	Q3U481	Q3U481	Mfsd12	73822	#N/A	#N/A	#N/A
ShSirt1_vs_ShScrambled	Q3UA37	Q3UA37	Qrich1	69232	#N/A	#N/A	#N/A
ShSirt1_vs_ShScrambled	Q3UE17	Q3UE17	Mex3d	237400	#N/A	#N/A	#N/A
ShSirt1_vs_ShScrambled	Q3UFS4	Q3UFS4	Gpatch11	53951	#N/A	#N/A	#N/A
ShSirt1_vs_ShScrambled	Q3UGF1	Q3UGF1	Wdr19	213081	#N/A	#N/A	#N/A
ShSirt1_vs_ShScrambled	Q3UHK8	Q3UHK8	Tnrc6a	233833	#N/A	#N/A	#N/A
ShSirt1_vs_ShScrambled	Q3UHX9	Q3UHX9	D2Wsu81e	227695	#N/A	#N/A	#N/A
ShSirt1_vs_ShScrambled	Q3UIR3	Q3UIR3	Dtx3l	209200	#N/A	#N/A	#N/A
ShSirt1_vs_ShScrambled	Q3UJP5	Q3UJP5	Q3UJP5	67157	#N/A	#N/A	#N/A
ShSirt1_vs_ShScrambled	Q3UMR0	Q3UMR0	Ankrd27	245886	#N/A	#N/A	#N/A
ShSirt1_vs_ShScrambled	Q3UR97	Q3UR97	Snx21	101113	#N/A	#N/A	#N/A
ShSirt1_vs_ShScrambled	Q3UU96	Q3UU96	Cdc42bpa	226751	#N/A	#N/A	#N/A
ShSirt1_vs_ShScrambled	Q4FZF3	Q4FZF3	Ddx49	234374	#N/A	#N/A	#N/A
ShSirt1_vs_ShScrambled	Q4G5Y1	Q4G5Y1	Klhdc2	69554	#N/A	#N/A	#N/A
ShSirt1_vs_ShScrambled	Q4VAC9	Q4VAC9	Plekhg3	263406	#N/A	#N/A	#N/A
ShSirt1_vs_ShScrambled	Q4VBD9	Q4VBD9	Gzf1	74533	#N/A	#N/A	#N/A
ShSirt1_vs_ShScrambled	Q5DTU0	Q5DTU0	Afap1l2	226250	#N/A	#N/A	#N/A

ShSirt1_vs_ShScrambled	Q5DTX6	Q5DTX6	Jcad	240185	#N/A	#N/A	#N/A
ShSirt1_vs_ShScrambled	Q5DTZ0	Q5DTZ0	Nynrin	277154	#N/A	#N/A	#N/A
ShSirt1_vs_ShScrambled	Q5DU25	Q5DU25	Iqsec2	245666	#N/A	#N/A	#N/A
ShSirt1_vs_ShScrambled	Q5HZI1	Q5HZI1	Mtus1	102103	#N/A	#N/A	#N/A
ShSirt1_vs_ShScrambled	Q5I012	Q5I012	Slc38a10	72055	#N/A	#N/A	#N/A
ShSirt1_vs_ShScrambled	Q5I1X5	Q5I1X5	Ppp1r13l	333654	#N/A	#N/A	#N/A
ShSirt1_vs_ShScrambled	Q5NCR9	Q5NCR9	Nsrp1	237859	#N/A	#N/A	#N/A
ShSirt1_vs_ShScrambled	Q5PSV9	Q5PSV9	Mdc1	240087	#N/A	#N/A	#N/A
ShSirt1_vs_ShScrambled	Q5PT53	Q5PT53	Slc10a7	76775	#N/A	#N/A	#N/A
ShSirt1_vs_ShScrambled	Q5RJ54	Q5RJ54	Zscan26	432731	#N/A	#N/A	#N/A
ShSirt1_vs_ShScrambled	Q5SS80	Q5SS80	Dhrs13	70451	#N/A	#N/A	#N/A
ShSirt1_vs_ShScrambled	Q5SV85	Q5SV85	Synrg	217030	#N/A	#N/A	#N/A
ShSirt1_vs_ShScrambled	Q5SWW4	Q5SWW4	Med13	327987	#N/A	#N/A	#N/A
ShSirt1_vs_ShScrambled	Q5U4C3	Q5U4C3	Scaf1	233208	#N/A	#N/A	#N/A
ShSirt1_vs_ShScrambled	Q5U4F6	Q5U4F6	Wdr34	71820	#N/A	#N/A	#N/A
ShSirt1_vs_ShScrambled	Q5U680	Q5U680	Slc25a26	67582	#N/A	#N/A	#N/A
ShSirt1_vs_ShScrambled	Q5XJE5	Q5XJE5	Leo1	235497	#N/A	#N/A	#N/A
ShSirt1_vs_ShScrambled	Q5XPI3	Q5XPI3	Rnf123	84585	#N/A	#N/A	#N/A
ShSirt1_vs_ShScrambled	Q5Y5T1	Q5Y5T1	Zdhhc20	75965	#N/A	#N/A	#N/A
ShSirt1_vs_ShScrambled	Q60778	Q60778	Nfkbib	18036	#N/A	#N/A	#N/A
ShSirt1_vs_ShScrambled	Q60960	Q60960	Kpna1	16646	#N/A	#N/A	#N/A
ShSirt1_vs_ShScrambled	Q61036	Q61036	Pak3	18481	#N/A	#N/A	#N/A
ShSirt1_vs_ShScrambled	Q61048	Q61048	Wbp4	22380	#N/A	#N/A	#N/A
ShSirt1_vs_ShScrambled	Q61083	Q61083	Map3k2		#N/A	#N/A	#N/A
ShSirt1_vs_ShScrambled	Q61084	Q61084	Map3k3	26406	#N/A	#N/A	#N/A
ShSirt1_vs_ShScrambled	Q61107	Q61107	Gbp4	55932	#N/A	#N/A	#N/A
ShSirt1_vs_ShScrambled	Q61301	Q61301	Ctnna2	12386	#N/A	#N/A	#N/A
ShSirt1_vs_ShScrambled	Q61315	Q61315	Apc		#N/A	#N/A	#N/A
ShSirt1_vs_ShScrambled	Q61458	Q61458	Ccnh	66671	#N/A	#N/A	#N/A
ShSirt1_vs_ShScrambled	Q61466	Q61466	Smarcd1	83797	#N/A	#N/A	#N/A
ShSirt1_vs_ShScrambled	Q61554	Q61554	Fbn1	14118	#N/A	#N/A	#N/A
ShSirt1_vs_ShScrambled	Q61559	Q61559	Fcgrt	14132	#N/A	#N/A	#N/A
ShSirt1_vs_ShScrambled	Q61666	Q61666	Hira	15260	#N/A	#N/A	#N/A
ShSirt1_vs_ShScrambled	Q62036	Q62036	Cep131	12009	#N/A	#N/A	#N/A
ShSirt1_vs_ShScrambled	Q62176	Q62176;D3Z	Rbm38		#N/A	#N/A	#N/A
ShSirt1_vs_ShScrambled	Q62193	Q62193	Rpa2		#N/A	#N/A	#N/A
ShSirt1_vs_ShScrambled	Q62311	Q62311	Taf6	21343	#N/A	#N/A	#N/A
ShSirt1_vs_ShScrambled	Q62371	Q62371	Ddr2	18214	#N/A	#N/A	#N/A
ShSirt1_vs_ShScrambled	Q62388	Q62388	Atm	11920	#N/A	#N/A	#N/A
ShSirt1_vs_ShScrambled	Q62393	Q62393	Tpd52	21985	#N/A	#N/A	#N/A
ShSirt1_vs_ShScrambled	Q64151	Q64151	Sema4c	20353	#N/A	#N/A	#N/A
ShSirt1_vs_ShScrambled	Q64282	Q64282	Ifit1	15957	#N/A	#N/A	#N/A
ShSirt1_vs_ShScrambled	Q64520	Q64520	Guk1	14923	#N/A	#N/A	#N/A
ShSirt1_vs_ShScrambled	Q64522	Q64522	Hist2h2ab	621893	#N/A	#N/A	#N/A
ShSirt1_vs_ShScrambled	Q65Z40	Q65Z40	Wapal	218914	#N/A	#N/A	#N/A
ShSirt1_vs_ShScrambled	Q68ED3	Q68ED3	Papd5	214627	#N/A	#N/A	#N/A
ShSirt1_vs_ShScrambled	Q68EF0	Q68EF0	Rab3ip	216363	#N/A	#N/A	#N/A
ShSirt1_vs_ShScrambled	Q68FE2	Q68FE2	Atg9a	245860	#N/A	#N/A	#N/A
ShSirt1_vs_ShScrambled	Q69ZN6	Q69ZN6	Gnptab	432486	#N/A	#N/A	#N/A
ShSirt1_vs_ShScrambled	Q69ZS0	Q69ZS0	Pdzrn3	55983	#N/A	#N/A	#N/A
ShSirt1_vs_ShScrambled	Q69ZW3	Q69ZW3	Ehbp1	216565	#N/A	#N/A	#N/A
ShSirt1_vs_ShScrambled	Q69ZZ6	Q69ZZ6	Tmcc1	330401	#N/A	#N/A	#N/A
ShSirt1_vs_ShScrambled	Q6A037	Q6A037	N4bp1	80750	#N/A	#N/A	#N/A
ShSirt1_vs_ShScrambled	Q6A070	Q6A070	Fam179b	328108	#N/A	#N/A	#N/A
ShSirt1_vs_ShScrambled	Q6DFV8	Q6DFV8	Vwde	232585	#N/A	#N/A	#N/A
ShSirt1_vs_ShScrambled	Q6IR34	Q6IR34	Gpsm1	67839	#N/A	#N/A	#N/A
ShSirt1_vs_ShScrambled	Q6KAQ7	Q6KAQ7	Zzz3	108946	#N/A	#N/A	#N/A
ShSirt1_vs_ShScrambled	Q6NS57	Q6NS57	Mapkbp1	26390	#N/A	#N/A	#N/A
ShSirt1_vs_ShScrambled	Q6NT99	Q6NT99	Dusp23	68440	#N/A	#N/A	#N/A
ShSirt1_vs_ShScrambled	Q6NVF0	Q6NVF0	Ocrl	320634	#N/A	#N/A	#N/A
ShSirt1_vs_ShScrambled	Q6NXJ0	Q6NXJ0	Wwc2	52357	#N/A	#N/A	#N/A
ShSirt1_vs_ShScrambled	Q6NXW6	Q6NXW6	Rad17	19356	#N/A	#N/A	#N/A
ShSirt1_vs_ShScrambled	Q6NZQ2	Q6NZQ2	Ddx31	227674	#N/A	#N/A	#N/A

ShSirt1_vs_ShScrambled	Q6P2L6	Q6P2L6	Whsc1l1	234135	#N/A	#N/A	#N/A
ShSirt1_vs_ShScrambled	Q6P6I6	Q6P6I6	Polr2m	28015	#N/A	#N/A	#N/A
ShSirt1_vs_ShScrambled	Q6PAL0	Q6PAL0	Bend3	331623	#N/A	#N/A	#N/A
ShSirt1_vs_ShScrambled	Q6PAL7	Q6PAL7	Ahdc1	230793	#N/A	#N/A	#N/A
ShSirt1_vs_ShScrambled	Q6PAL8	Q6PAL8	Dennd5a	19347	#N/A	#N/A	#N/A
ShSirt1_vs_ShScrambled	Q6PAM0	Q6PAM0	Prkab2	108097	#N/A	#N/A	#N/A
ShSirt1_vs_ShScrambled	Q6PAR0	Q6PAR0	Klhdc10	76788	#N/A	#N/A	#N/A
ShSirt1_vs_ShScrambled	Q6PB90	Q6PB90;Q91	Pcdhb14		#N/A	#N/A	#N/A
ShSirt1_vs_ShScrambled	Q6PCM1	Q6PCM1	Kdm3a	104263	#N/A	#N/A	#N/A
ShSirt1_vs_ShScrambled	Q6PD10	Q6PD10	Ip6k1	27399	#N/A	#N/A	#N/A
ShSirt1_vs_ShScrambled	Q6PD28	Q6PD28	Ppp2r5b	225849	#N/A	#N/A	#N/A
ShSirt1_vs_ShScrambled	Q6PFQ7	Q6PFQ7	Rasa4	54153	#N/A	#N/A	#N/A
ShSirt1_vs_ShScrambled	Q6PGF3	Q6PGF3	Med16		#N/A	#N/A	#N/A
ShSirt1_vs_ShScrambled	Q6PGH2	Q6PGH2	Hn1l	52009	#N/A	#N/A	#N/A
ShSirt1_vs_ShScrambled	Q6PIX5	Q6PIX5	Rhbdf1	13650	#N/A	#N/A	#N/A
ShSirt1_vs_ShScrambled	Q6PJN8	Q6PJN8	Dalrd3	67789	#N/A	#N/A	#N/A
ShSirt1_vs_ShScrambled	Q6Q2Z6	Q6Q2Z6;Q90	Acot5		#N/A	#N/A	#N/A
ShSirt1_vs_ShScrambled	Q6QD59	Q6QD59	Bnip1	224630	#N/A	#N/A	#N/A
ShSirt1_vs_ShScrambled	Q6R5N8	Q6R5N8	Tlr13	279572	#N/A	#N/A	#N/A
ShSirt1_vs_ShScrambled	Q6URW6	Q6URW6	Myh14	71960	#N/A	#N/A	#N/A
ShSirt1_vs_ShScrambled	Q6VH22	Q6VH22	Ift172	67661	#N/A	#N/A	#N/A
ShSirt1_vs_ShScrambled	Q6VVW5	Q6VVW5	Npr2	230103	#N/A	#N/A	#N/A
ShSirt1_vs_ShScrambled	Q6Y5D8	Q6Y5D8	Arhgap10	78514	#N/A	#N/A	#N/A
ShSirt1_vs_ShScrambled	Q6ZPQ6	Q6ZPQ6	Pitpnm2	19679	#N/A	#N/A	#N/A
ShSirt1_vs_ShScrambled	Q6ZPV2	Q6ZPV2	Ino80	68142	#N/A	#N/A	#N/A
ShSirt1_vs_ShScrambled	Q6ZPY7	Q6ZPY7	Kdm3b		#N/A	#N/A	#N/A
ShSirt1_vs_ShScrambled	Q6ZQ03	Q6ZQ03	Fnbp4	55935	#N/A	#N/A	#N/A
ShSirt1_vs_ShScrambled	Q6ZQ29	Q6ZQ29	Taok2	381921	#N/A	#N/A	#N/A
ShSirt1_vs_ShScrambled	Q6ZWQ0	Q6ZWQ0	Syne2	319565	#N/A	#N/A	#N/A
ShSirt1_vs_ShScrambled	Q6ZWQ7	Q6ZWQ7	Spcs3	76687	#N/A	#N/A	#N/A
ShSirt1_vs_ShScrambled	Q711T7	Q711T7	Nadsyn1	78914	#N/A	#N/A	#N/A
ShSirt1_vs_ShScrambled	Q76KJ5	Q76KJ5	Cd3eap	70333	#N/A	#N/A	#N/A
ShSirt1_vs_ShScrambled	Q7SIG6	Q7SIG6	Asap2	211914	#N/A	#N/A	#N/A
ShSirt1_vs_ShScrambled	Q7TMF2	Q7TMF2	Eri1	67276	#N/A	#N/A	#N/A
ShSirt1_vs_ShScrambled	Q7TNF9	Q7TNF9	Fam117a	215512	#N/A	#N/A	#N/A
ShSirt1_vs_ShScrambled	Q7TNS2	Q7TNS2	Minos1	433771	#N/A	#N/A	#N/A
ShSirt1_vs_ShScrambled	Q7TPC1	Q7TPC1	Cdsn	386463	#N/A	#N/A	#N/A
ShSirt1_vs_ShScrambled	Q7TPS5	Q7TPS5	C2cd5	74741	#N/A	#N/A	#N/A
ShSirt1_vs_ShScrambled	Q7TQK4	Q7TQK4	Exosc3	66362	#N/A	#N/A	#N/A
ShSirt1_vs_ShScrambled	Q7TSH3	Q7TSH3	Znf516	329003	#N/A	#N/A	#N/A
ShSirt1_vs_ShScrambled	Q7TSH6	Q7TSH6	Scaf4	224432	#N/A	#N/A	#N/A
ShSirt1_vs_ShScrambled	Q7TT18	Q7TT18	Atf7ip	54343	#N/A	#N/A	#N/A
ShSirt1_vs_ShScrambled	Q80T69	Q80T69	Rsbn1	229675	#N/A	#N/A	#N/A
ShSirt1_vs_ShScrambled	Q80TN5	Q80TN5	Zdhhc17	320150	#N/A	#N/A	#N/A
ShSirt1_vs_ShScrambled	Q80TZ9	Q80TZ9	Rere	68703	#N/A	#N/A	#N/A
ShSirt1_vs_ShScrambled	Q80U30	Q80U30	Clec16a	74374	#N/A	#N/A	#N/A
ShSirt1_vs_ShScrambled	Q80UE6	Q80UE6	Wnk4	69847	#N/A	#N/A	#N/A
ShSirt1_vs_ShScrambled	Q80UF7	Q80UF7	Ticam1	106759	#N/A	#N/A	#N/A
ShSirt1_vs_ShScrambled	Q80UW8	Q80UW8	Polr2e	66420	#N/A	#N/A	#N/A
ShSirt1_vs_ShScrambled	Q80UX8	Q80UX8	Abhd13	68904	#N/A	#N/A	#N/A
ShSirt1_vs_ShScrambled	Q80V94	Q80V94	Ap4e1	108011	#N/A	#N/A	#N/A
ShSirt1_vs_ShScrambled	Q80VL1	Q80VL1	Tdrkh	72634	#N/A	#N/A	#N/A
ShSirt1_vs_ShScrambled	Q80VP5	Q80VP5	Q80VP5	72650	#N/A	#N/A	#N/A
ShSirt1_vs_ShScrambled	Q80VY9	Q80VY9	Dhx33	216877	#N/A	#N/A	#N/A
ShSirt1_vs_ShScrambled	Q80W68	Q80W68	Kirrel	170643	#N/A	#N/A	#N/A
ShSirt1_vs_ShScrambled	Q80WC1	Q80WC1	Ubn2	320538	#N/A	#N/A	#N/A
ShSirt1_vs_ShScrambled	Q80XS6	Q80XS6	Samd4b		#N/A	#N/A	#N/A
ShSirt1_vs_ShScrambled	Q80Y44	Q80Y44	Ddx10	77591	#N/A	#N/A	#N/A
ShSirt1_vs_ShScrambled	Q80YA7	Q80YA7	Dpp8	74388	#N/A	#N/A	#N/A
ShSirt1_vs_ShScrambled	Q80YT7	Q80YT7	Pde4dip	83679	#N/A	#N/A	#N/A
ShSirt1_vs_ShScrambled	Q80ZI6	Q80ZI6	Lrsam1	227738	#N/A	#N/A	#N/A
ShSirt1_vs_ShScrambled	Q80ZW2	Q80ZW2	Them6	223626	#N/A	#N/A	#N/A
ShSirt1_vs_ShScrambled	Q810D6	Q810D6	Grwd1	101612	#N/A	#N/A	#N/A

ShSirt1_vs_ShScrambled	Q811L6	Q811L6	Mast4	328329	#N/A	#N/A	#N/A
ShSirt1_vs_ShScrambled	Q811U4	Q811U4	Mfn1	67414	#N/A	#N/A	#N/A
ShSirt1_vs_ShScrambled	Q812F8	Q812F8	Mgat4b	103534	#N/A	#N/A	#N/A
ShSirt1_vs_ShScrambled	Q8BG15	Q8BG15	Ctdspl2	329506	#N/A	#N/A	#N/A
ShSirt1_vs_ShScrambled	Q8BG60	Q8BG60	Txnip	56338	#N/A	#N/A	#N/A
ShSirt1_vs_ShScrambled	Q8BG94	Q8BG94	Commd7	99311	#N/A	#N/A	#N/A
ShSirt1_vs_ShScrambled	Q8BGA7	Q8BGA7	Q8BGA7	231868	#N/A	#N/A	#N/A
ShSirt1_vs_ShScrambled	Q8BGD6	Q8BGD6	Slc38a9	268706	#N/A	#N/A	#N/A
ShSirt1_vs_ShScrambled	Q8BGH7	Q8BGH7	Cdc42se2	72729	#N/A	#N/A	#N/A
ShSirt1_vs_ShScrambled	Q8BGI5	Q8BGI5	Pex26	74043	#N/A	#N/A	#N/A
ShSirt1_vs_ShScrambled	Q8BGT6	Q8BGT6	Micall1	27008	#N/A	#N/A	#N/A
ShSirt1_vs_ShScrambled	Q8BGV8	Q8BGV8	Mief1	239555	#N/A	#N/A	#N/A
ShSirt1_vs_ShScrambled	Q8BGW2	Q8BGW2	Wbp1l	226178	#N/A	#N/A	#N/A
ShSirt1_vs_ShScrambled	Q8BGW5	Q8BGW5	Nabp1	109019	#N/A	#N/A	#N/A
ShSirt1_vs_ShScrambled	Q8BH07	Q8BH07	Arl6ip6	65103	#N/A	#N/A	#N/A
ShSirt1_vs_ShScrambled	Q8BH31	Q8BH31	Mfsd8	72175	#N/A	#N/A	#N/A
ShSirt1_vs_ShScrambled	Q8BH44	Q8BH44	Coro2b	235431	#N/A	#N/A	#N/A
ShSirt1_vs_ShScrambled	Q8BH48	Q8BH48	Ubap1	67123	#N/A	#N/A	#N/A
ShSirt1_vs_ShScrambled	Q8BH57	Q8BH57	Wdr48	67561	#N/A	#N/A	#N/A
ShSirt1_vs_ShScrambled	Q8BH65	Q8BH65	Dennd6a	211922	#N/A	#N/A	#N/A
ShSirt1_vs_ShScrambled	Q8BH73	Q8BH73	Qpctl	67369	#N/A	#N/A	#N/A
ShSirt1_vs_ShScrambled	Q8BHG9	Q8BHG9	Cggbp1	106143	#N/A	#N/A	#N/A
ShSirt1_vs_ShScrambled	Q8BHJ5	Q8BHJ5	Tbl1xr1	81004	#N/A	#N/A	#N/A
ShSirt1_vs_ShScrambled	Q8BHK1	Q8BHK1	Nipa1	233280	#N/A	#N/A	#N/A
ShSirt1_vs_ShScrambled	Q8BHK9	Q8BHK9	Ercc6l	236930	#N/A	#N/A	#N/A
ShSirt1_vs_ShScrambled	Q8BIH0	Q8BIH0	Sap130	269003	#N/A	#N/A	#N/A
ShSirt1_vs_ShScrambled	Q8BIV3	Q8BIV3	Ranbp6	240614	#N/A	#N/A	#N/A
ShSirt1_vs_ShScrambled	Q8BJ63	Q8BJ63	Ap5m1	74385	#N/A	#N/A	#N/A
ShSirt1_vs_ShScrambled	Q8BJL1	Q8BJL1	Fbxo30	71865	#N/A	#N/A	#N/A
ShSirt1_vs_ShScrambled	Q8BJM7	Q8BJM7	Tyw1	100929	#N/A	#N/A	#N/A
ShSirt1_vs_ShScrambled	Q8BK58	Q8BK58	Hspbap1	66667	#N/A	#N/A	#N/A
ShSirt1_vs_ShScrambled	Q8BKH7	Q8BKH7	Mapkap1	227743	#N/A	#N/A	#N/A
ShSirt1_vs_ShScrambled	Q8BKT3	Q8BKT3	Gcfc2	330361	#N/A	#N/A	#N/A
ShSirt1_vs_ShScrambled	Q8BL03	Q8BL03	Slc25a29	214663	#N/A	#N/A	#N/A
ShSirt1_vs_ShScrambled	Q8BL48	Q8BL48	Unk	217331	#N/A	#N/A	#N/A
ShSirt1_vs_ShScrambled	Q8BLA8	Q8BLA8	Trpa1	277328	#N/A	#N/A	#N/A
ShSirt1_vs_ShScrambled	Q8BLR9	Q8BLR9	Hif1an	319594	#N/A	#N/A	#N/A
ShSirt1_vs_ShScrambled	Q8BMC4	Q8BMC4	Nop9	67842	#N/A	#N/A	#N/A
ShSirt1_vs_ShScrambled	Q8BMI0	Q8BMI0	Fbxo38	107035	#N/A	#N/A	#N/A
ShSirt1_vs_ShScrambled	Q8BMQ2	Q8BMQ2	Gtf3c4	269252	#N/A	#N/A	#N/A
ShSirt1_vs_ShScrambled	Q8BN21	Q8BN21	Vrk2	69922	#N/A	#N/A	#N/A
ShSirt1_vs_ShScrambled	Q8BND3	Q8BND3	Wdr35	74682	#N/A	#N/A	#N/A
ShSirt1_vs_ShScrambled	Q8BND5	Q8BND5	Qsox1	104009	#N/A	#N/A	#N/A
ShSirt1_vs_ShScrambled	Q8BNI4	Q8BNI4	Derl2	116891	#N/A	#N/A	#N/A
ShSirt1_vs_ShScrambled	Q8BNV1	Q8BNV1	Trmt2a	15547	#N/A	#N/A	#N/A
ShSirt1_vs_ShScrambled	Q8BP00	Q8BP00	Iqcb1	320299	#N/A	#N/A	#N/A
ShSirt1_vs_ShScrambled	Q8BP01	Q8BP01	Vmac	106639	#N/A	#N/A	#N/A
ShSirt1_vs_ShScrambled	Q8BP78	Q8BP78	Fra10ac1	70567	#N/A	#N/A	#N/A
ShSirt1_vs_ShScrambled	Q8BPS4	Q8BPS4	Gpr180	58245	#N/A	#N/A	#N/A
ShSirt1_vs_ShScrambled	Q8BPZ8	Q8BPZ8	Fam175a	70681	#N/A	#N/A	#N/A
ShSirt1_vs_ShScrambled	Q8BS95	Q8BS95	Gpr89a	67549	#N/A	#N/A	#N/A
ShSirt1_vs_ShScrambled	Q8BSI6	Q8BSI6	R3hcc1	71843	#N/A	#N/A	#N/A
ShSirt1_vs_ShScrambled	Q8BSN5	Q8BSN5	Phf23	78246	#N/A	#N/A	#N/A
ShSirt1_vs_ShScrambled	Q8BSS9	Q8BSS9	Ppfia2	327814	#N/A	#N/A	#N/A
ShSirt1_vs_ShScrambled	Q8BTG3	Q8BTG3	Tcp11l1	320554	#N/A	#N/A	#N/A
ShSirt1_vs_ShScrambled	Q8BTI7	Q8BTI7	Ankrd52	237615	#N/A	#N/A	#N/A
ShSirt1_vs_ShScrambled	Q8BTT6	Q8BTT6	Diexf	215193	#N/A	#N/A	#N/A
ShSirt1_vs_ShScrambled	Q8BTX9	Q8BTX9	Hsdl1	72552	#N/A	#N/A	#N/A
ShSirt1_vs_ShScrambled	Q8BTY2	Q8BTY2	Slc4a7		#N/A	#N/A	#N/A
ShSirt1_vs_ShScrambled	Q8BVF2	Q8BVF2	Pdcl3	68833	#N/A	#N/A	#N/A
ShSirt1_vs_ShScrambled	Q8BVH9	Q8BVH9	Mettl6	67011	#N/A	#N/A	#N/A
ShSirt1_vs_ShScrambled	Q8BVP5	Q8BVP5;Q8(	Csnk1g2		#N/A	#N/A	#N/A
ShSirt1_vs_ShScrambled	Q8BW00	Q8BW00	Ptrh1	329384	#N/A	#N/A	#N/A

ShSirt1_vs_ShScrambled	Q8BW75	Q8BW75	Maob	109731	#N/A	#N/A	#N/A
ShSirt1_vs_ShScrambled	Q8BW96	Q8BW96	Camk1d	227541	#N/A	#N/A	#N/A
ShSirt1_vs_ShScrambled	Q8BWT5	Q8BWT5	Dip2a	64451	#N/A	#N/A	#N/A
ShSirt1_vs_ShScrambled	Q8BXL7	Q8BXL7	Arfrp1	76688	#N/A	#N/A	#N/A
ShSirt1_vs_ShScrambled	Q8BY89	Q8BY89	Slc44a2	68682	#N/A	#N/A	#N/A
ShSirt1_vs_ShScrambled	Q8BYC6	Q8BYC6	Taok3	330177	#N/A	#N/A	#N/A
ShSirt1_vs_ShScrambled	Q8BYN3	Q8BYN3	Itpk1	217837	#N/A	#N/A	#N/A
ShSirt1_vs_ShScrambled	Q8BYR2	Q8BYR2	Lats1	16798	#N/A	#N/A	#N/A
ShSirt1_vs_ShScrambled	Q8BZH4	Q8BZH4	Pogz	229584	#N/A	#N/A	#N/A
ShSirt1_vs_ShScrambled	Q8BZR9	Q8BZR9	Q8BZR9	66874	#N/A	#N/A	#N/A
ShSirt1_vs_ShScrambled	Q8C006	Q8C006	Trim35	66854	#N/A	#N/A	#N/A
ShSirt1_vs_ShScrambled	Q8C079	Q8C079	Strip1	229707	#N/A	#N/A	#N/A
ShSirt1_vs_ShScrambled	Q8C092	Q8C092	Taf5	226182	#N/A	#N/A	#N/A
ShSirt1_vs_ShScrambled	Q8C0D0	Q8C0D0	Trub1	72133	#N/A	#N/A	#N/A
ShSirt1_vs_ShScrambled	Q8C0J6	Q8C0J6	Sowahc	268301	#N/A	#N/A	#N/A
ShSirt1_vs_ShScrambled	Q8C0K5	Q8C0K5	Slc25a16	73132	#N/A	#N/A	#N/A
ShSirt1_vs_ShScrambled	Q8C0V0	Q8C0V0	Tlk1	228012	#N/A	#N/A	#N/A
ShSirt1_vs_ShScrambled	Q8C142	Q8C142	Ldlrap1	100017	#N/A	#N/A	#N/A
ShSirt1_vs_ShScrambled	Q8C181	Q8C181	Mbnl2	105559	#N/A	#N/A	#N/A
ShSirt1_vs_ShScrambled	Q8C1B1	Q8C1B1	Camsap2	67886	#N/A	#N/A	#N/A
ShSirt1_vs_ShScrambled	Q8C1F4	Q8C1F4	Csgalnact2	78752	#N/A	#N/A	#N/A
ShSirt1_vs_ShScrambled	Q8C2B3	Q8C2B3	Hdac7	56233	#N/A	#N/A	#N/A
ShSirt1_vs_ShScrambled	Q8C4Q6	Q8C4Q6	Aida	108909	#N/A	#N/A	#N/A
ShSirt1_vs_ShScrambled	Q8C569	Q8C569	Fam118b	109229	#N/A	#N/A	#N/A
ShSirt1_vs_ShScrambled	Q8C5K5	Q8C5K5	Q8C5K5	69155	#N/A	#N/A	#N/A
ShSirt1_vs_ShScrambled	Q8C5N5	Q8C5N5	Pdcd2l	68079	#N/A	#N/A	#N/A
ShSirt1_vs_ShScrambled	Q8C5W3	Q8C5W3	Tbcel	272589	#N/A	#N/A	#N/A
ShSirt1_vs_ShScrambled	Q8C6E0	Q8C6E0	Cfap36	216618	#N/A	#N/A	#N/A
ShSirt1_vs_ShScrambled	Q8C6U2	Q8C6U2	Pqlc3	217430	#N/A	#N/A	#N/A
ShSirt1_vs_ShScrambled	Q8C761	Q8C761	Wdr60	217935	#N/A	#N/A	#N/A
ShSirt1_vs_ShScrambled	Q8C7D2	Q8C7D2	Crbn	58799	#N/A	#N/A	#N/A
ShSirt1_vs_ShScrambled	Q8C863	Q8C863	Itch	16396	#N/A	#N/A	#N/A
ShSirt1_vs_ShScrambled	Q8C8T8	Q8C8T8	Tsr2	69499	#N/A	#N/A	#N/A
ShSirt1_vs_ShScrambled	Q8C9S8	Q8C9S8	Atg4a	666468	#N/A	#N/A	#N/A
ShSirt1_vs_ShScrambled	Q8CBQ5	Q8CBQ5	Pi4k2b	67073	#N/A	#N/A	#N/A
ShSirt1_vs_ShScrambled	Q8CBY0	Q8CBY0	Gatc	384281	#N/A	#N/A	#N/A
ShSirt1_vs_ShScrambled	Q8CBY1	Q8CBY1	Samd4a	74480	#N/A	#N/A	#N/A
ShSirt1_vs_ShScrambled	Q8CC86	Q8CC86	Naprt	223646	#N/A	#N/A	#N/A
ShSirt1_vs_ShScrambled	Q8CD15	Q8CD15	Mina	67014	#N/A	#N/A	#N/A
ShSirt1_vs_ShScrambled	Q8CD92	Q8CD92	Ttc27	74196	#N/A	#N/A	#N/A
ShSirt1_vs_ShScrambled	Q8CDL9	Q8CDL9	Ccdc87	399599	#N/A	#N/A	#N/A
ShSirt1_vs_ShScrambled	Q8CF89	Q8CF89	Tab1	66513	#N/A	#N/A	#N/A
ShSirt1_vs_ShScrambled	Q8CFE2	Q8CFE2	Q8CFE2	72612	#N/A	#N/A	#N/A
ShSirt1_vs_ShScrambled	Q8CFE3	Q8CFE3;Q6F	Rcor1		#N/A	#N/A	#N/A
ShSirt1_vs_ShScrambled	Q8CFE5	Q8CFE5	Btbd7	238386	#N/A	#N/A	#N/A
ShSirt1_vs_ShScrambled	Q8CFU8	Q8CFU8	Tp53inp2	68728	#N/A	#N/A	#N/A
ShSirt1_vs_ShScrambled	Q8CG48	Q8CG48	Smc2	14211	#N/A	#N/A	#N/A
ShSirt1_vs_ShScrambled	Q8CGC4	Q8CGC4	Lsm14b	241846	#N/A	#N/A	#N/A
ShSirt1_vs_ShScrambled	Q8CGF1	Q8CGF1	Arhgap29	214137	#N/A	#N/A	#N/A
ShSirt1_vs_ShScrambled	Q8CGN4	Q8CGN4	Bcor	71458	#N/A	#N/A	#N/A
ShSirt1_vs_ShScrambled	Q8CH02	Q8CH02	Sugp1	70616	#N/A	#N/A	#N/A
ShSirt1_vs_ShScrambled	Q8CHP6	Q8CHP6	Phc3	241915	#N/A	#N/A	#N/A
ShSirt1_vs_ShScrambled	Q8CI78	Q8CI78	Rmnd1	66084	#N/A	#N/A	#N/A
ShSirt1_vs_ShScrambled	Q8CIB6	Q8CIB6	Tmem230	70612	#N/A	#N/A	#N/A
ShSirt1_vs_ShScrambled	Q8CIG3	Q8CIG3	Kdm1b	218214	#N/A	#N/A	#N/A
ShSirt1_vs_ShScrambled	Q8CIV8	Q8CIV8	Tbce	70430	#N/A	#N/A	#N/A
ShSirt1_vs_ShScrambled	Q8CJ67	Q8CJ67	Stau2	29819	#N/A	#N/A	#N/A
ShSirt1_vs_ShScrambled	Q8JZX9	Q8JZX9	Cdc42ep2	104252	#N/A	#N/A	#N/A
ShSirt1_vs_ShScrambled	Q8JZY4	Q8JZY4	Kiaa0391	66132	#N/A	#N/A	#N/A
ShSirt1_vs_ShScrambled	Q8K072	Q8K072	Reep4	72549	#N/A	#N/A	#N/A
ShSirt1_vs_ShScrambled	Q8K0C1	Q8K0C1	Ipo13	230673	#N/A	#N/A	#N/A
ShSirt1_vs_ShScrambled	Q8K0H5	Q8K0H5	Taf10	24075	#N/A	#N/A	#N/A
ShSirt1_vs_ShScrambled	Q8K124	Q8K124	Plekho2	102595	#N/A	#N/A	#N/A

ShSirt1_vs_ShScrambled	Q8K126	Q8K126	Mtrf1	211253	#N/A	#N/A	#N/A
ShSirt1_vs_ShScrambled	Q8K1L6	Q8K1L6	Q8K1L6	68918	#N/A	#N/A	#N/A
ShSirt1_vs_ShScrambled	Q8K284	Q8K284	Gtf3c1	233863	#N/A	#N/A	#N/A
ShSirt1_vs_ShScrambled	Q8K2F0	Q8K2F0	Brd3	67382	#N/A	#N/A	#N/A
ShSirt1_vs_ShScrambled	Q8K2H2	Q8K2H2	Otud6b	72201	#N/A	#N/A	#N/A
ShSirt1_vs_ShScrambled	Q8K2I9	Q8K2I9	Fbxo18	50755	#N/A	#N/A	#N/A
ShSirt1_vs_ShScrambled	Q8K2J0	Q8K2J0	Plcd3	72469	#N/A	#N/A	#N/A
ShSirt1_vs_ShScrambled	Q8K2T4	Q8K2T4	Uqcc3	107197	#N/A	#N/A	#N/A
ShSirt1_vs_ShScrambled	Q8K2T8	Q8K2T8	Paf1	54624	#N/A	#N/A	#N/A
ShSirt1_vs_ShScrambled	Q8K301	Q8K301	Ddx52	78394	#N/A	#N/A	#N/A
ShSirt1_vs_ShScrambled	Q8K327	Q8K327	Champ1	101994	#N/A	#N/A	#N/A
ShSirt1_vs_ShScrambled	Q8K330	Q8K330	Ssh3	245857	#N/A	#N/A	#N/A
ShSirt1_vs_ShScrambled	Q8K389	Q8K389	Cdk5rap2	214444	#N/A	#N/A	#N/A
ShSirt1_vs_ShScrambled	Q8K3A0	Q8K3A0	Hscb	100900	#N/A	#N/A	#N/A
ShSirt1_vs_ShScrambled	Q8K3W3	Q8K3W3	Casc3	192160	#N/A	#N/A	#N/A
ShSirt1_vs_ShScrambled	Q8K3Z9	Q8K3Z9	Pom121	107939	#N/A	#N/A	#N/A
ShSirt1_vs_ShScrambled	Q8K409	Q8K409	Polb	18970	#N/A	#N/A	#N/A
ShSirt1_vs_ShScrambled	Q8K479	Q8K479	C1qtnf5	235312	#N/A	#N/A	#N/A
ShSirt1_vs_ShScrambled	Q8QZY9	Q8QZY9	Sf3b4	107701	#N/A	#N/A	#N/A
ShSirt1_vs_ShScrambled	Q8R0J4	Q8R0J4	Tmem134	66990	#N/A	#N/A	#N/A
ShSirt1_vs_ShScrambled	Q8R0Z5	Q8R0Z5	Slc25a28	246696	#N/A	#N/A	#N/A
ShSirt1_vs_ShScrambled	Q8R138	Q8R138	Tmem119	231633	#N/A	#N/A	#N/A
ShSirt1_vs_ShScrambled	Q8R1F9	Q8R1F9	Rpp40	208366	#N/A	#N/A	#N/A
ShSirt1_vs_ShScrambled	Q8R1G2	Q8R1G2	Cmbl	69574	#N/A	#N/A	#N/A
ShSirt1_vs_ShScrambled	Q8R1N0	Q8R1N0	Znf830	66983	#N/A	#N/A	#N/A
ShSirt1_vs_ShScrambled	Q8R2G6	Q8R2G6	Ccdc80	67896	#N/A	#N/A	#N/A
ShSirt1_vs_ShScrambled	Q8R2H9	Q8R2H9	Phospho1	237928	#N/A	#N/A	#N/A
ShSirt1_vs_ShScrambled	Q8R2K4	Q8R2K4	Taf6l	225895	#N/A	#N/A	#N/A
ShSirt1_vs_ShScrambled	Q8R2L5	Q8R2L5	Mrps18c	68735	#N/A	#N/A	#N/A
ShSirt1_vs_ShScrambled	Q8R2R9	Q8R2R9	Ap3m2	64933	#N/A	#N/A	#N/A
ShSirt1_vs_ShScrambled	Q8R344	Q8R344	Ccdc12	72654	#N/A	#N/A	#N/A
ShSirt1_vs_ShScrambled	Q8R3E3	Q8R3E3	Wipi1	52639	#N/A	#N/A	#N/A
ShSirt1_vs_ShScrambled	Q8R3F9	Q8R3F9	Tut1	70044	#N/A	#N/A	#N/A
ShSirt1_vs_ShScrambled	Q8R3H7	Q8R3H7	Hs2st1	23908	#N/A	#N/A	#N/A
ShSirt1_vs_ShScrambled	Q8R3H9	Q8R3H9	Ttc4	72354	#N/A	#N/A	#N/A
ShSirt1_vs_ShScrambled	Q8R3K3	Q8R3K3	Ptcd2	68927	#N/A	#N/A	#N/A
ShSirt1_vs_ShScrambled	Q8R3L8	Q8R3L8	Cdk8	264064	#N/A	#N/A	#N/A
ShSirt1_vs_ShScrambled	Q8R3P6	Q8R3P6	Vwa9	69882	#N/A	#N/A	#N/A
ShSirt1_vs_ShScrambled	Q8R3Q0	Q8R3Q0	Saraf	67887	#N/A	#N/A	#N/A
ShSirt1_vs_ShScrambled	Q8R4F1	Q8R4F1	Ntng2	171171	#N/A	#N/A	#N/A
ShSirt1_vs_ShScrambled	Q8R5J9	Q8R5J9	Arl6ip5	65106	#N/A	#N/A	#N/A
ShSirt1_vs_ShScrambled	Q8VBV3	Q8VBV3	Exosc2	227715	#N/A	#N/A	#N/A
ShSirt1_vs_ShScrambled	Q8VC04	Q8VC04	Tmem106a	217203	#N/A	#N/A	#N/A
ShSirt1_vs_ShScrambled	Q8VC48	Q8VC48	Pex12	103737	#N/A	#N/A	#N/A
ShSirt1_vs_ShScrambled	Q8VC60	Q8VC60	Glb1l	74577	#N/A	#N/A	#N/A
ShSirt1_vs_ShScrambled	Q8VC74	Q8VC74	Cox18	231430	#N/A	#N/A	#N/A
ShSirt1_vs_ShScrambled	Q8VCB2	Q8VCB2	Med25	75613	#N/A	#N/A	#N/A
ShSirt1_vs_ShScrambled	Q8VCE1	Q8VCE1	Dnajc28	246738	#N/A	#N/A	#N/A
ShSirt1_vs_ShScrambled	Q8VCF1	Q8VCF1	Cant1	76025	#N/A	#N/A	#N/A
ShSirt1_vs_ShScrambled	Q8VCM3	Q8VCM3	Zfyve21	68520	#N/A	#N/A	#N/A
ShSirt1_vs_ShScrambled	Q8VCQ3	Q8VCQ3	Nrbf2	641340	#N/A	#N/A	#N/A
ShSirt1_vs_ShScrambled	Q8VCY6	Q8VCY6	Utp6	216987	#N/A	#N/A	#N/A
ShSirt1_vs_ShScrambled	Q8VD26	Q8VD26	Tmem143	70209	#N/A	#N/A	#N/A
ShSirt1_vs_ShScrambled	Q8VDI7	Q8VDI7	Ubac1	98766	#N/A	#N/A	#N/A
ShSirt1_vs_ShScrambled	Q8VDI9	Q8VDI9	Alg9	102580	#N/A	#N/A	#N/A
ShSirt1_vs_ShScrambled	Q8VE09	Q8VE09	Ttc39c	72747	#N/A	#N/A	#N/A
ShSirt1_vs_ShScrambled	Q8VE10	Q8VE10	Naa40	70999	#N/A	#N/A	#N/A
ShSirt1_vs_ShScrambled	Q8VE94	Q8VE94	Fam110c	104943	#N/A	#N/A	#N/A
ShSirt1_vs_ShScrambled	Q8VE99	Q8VE99	Ccdc115	69668	#N/A	#N/A	#N/A
ShSirt1_vs_ShScrambled	Q8VEA4	Q8VEA4	Chchd4	72170	#N/A	#N/A	#N/A
ShSirt1_vs_ShScrambled	Q8VEL2	Q8VEL2	Mtmr14	97287	#N/A	#N/A	#N/A
ShSirt1_vs_ShScrambled	Q8WUR0	Q8WUR0	Q8WUR0	72244	#N/A	#N/A	#N/A
ShSirt1_vs_ShScrambled	Q91VK4	Q91VK4	Itm2c	64294	#N/A	#N/A	#N/A

ShSirt1_vs_ShScrambled	Q91VR8	Q91VR8	Brk1	101314	#N/A	#N/A	#N/A
ShSirt1_vs_ShScrambled	Q91VS8	Q91VS8	Farp2	227377	#N/A	#N/A	#N/A
ShSirt1_vs_ShScrambled	Q91VV4	Q91VV4	Dennd2d	72121	#N/A	#N/A	#N/A
ShSirt1_vs_ShScrambled	Q91VX9	Q91VX9	Tmem168	101118	#N/A	#N/A	#N/A
ShSirt1_vs_ShScrambled	Q91WD0	Q91WD0	Gpr108	78308	#N/A	#N/A	#N/A
ShSirt1_vs_ShScrambled	Q91WE2	Q91WE2	Fam192a	102122	#N/A	#N/A	#N/A
ShSirt1_vs_ShScrambled	Q91WE6	Q91WE6	Cdkal1	68916	#N/A	#N/A	#N/A
ShSirt1_vs_ShScrambled	Q91WK5	Q91WK5	Gcsh	68133	#N/A	#N/A	#N/A
ShSirt1_vs_ShScrambled	Q91WT8	Q91WT8	Rbm47	245945	#N/A	#N/A	#N/A
ShSirt1_vs_ShScrambled	Q91X51	Q91X51	Gorasp1	74498	#N/A	#N/A	#N/A
ShSirt1_vs_ShScrambled	Q91X84	Q91X84	Crtc3	70461	#N/A	#N/A	#N/A
ShSirt1_vs_ShScrambled	Q91XC8	Q91XC8	Dap	223453	#N/A	#N/A	#N/A
ShSirt1_vs_ShScrambled	Q91XE8	Q91XE8	Tmem205	235043	#N/A	#N/A	#N/A
ShSirt1_vs_ShScrambled	Q91XL2	Q91XL2	Lnx2	140887	#N/A	#N/A	#N/A
ShSirt1_vs_ShScrambled	Q91XQ0	Q91XQ0	Dnah8	13417	#N/A	#N/A	#N/A
ShSirt1_vs_ShScrambled	Q91YI6	Q91YI6	Papd4	100715	#N/A	#N/A	#N/A
ShSirt1_vs_ShScrambled	Q91YJ3	Q91YJ3	Thyn1	77862	#N/A	#N/A	#N/A
ShSirt1_vs_ShScrambled	Q91YT2	Q91YT2	Rnf185	193670	#N/A	#N/A	#N/A
ShSirt1_vs_ShScrambled	Q91Z92	Q91Z92	B3galt6	117592	#N/A	#N/A	#N/A
ShSirt1_vs_ShScrambled	Q91ZM2	Q91ZM2	Sh2b1	20399	#N/A	#N/A	#N/A
ShSirt1_vs_ShScrambled	Q91ZP6	Q91ZP6	Ndfip2		#N/A	#N/A	#N/A
ShSirt1_vs_ShScrambled	Q91ZU1	Q91ZU1	Asb6	72323	#N/A	#N/A	#N/A
ShSirt1_vs_ShScrambled	Q91ZZ5	Q91ZZ5	Rxfp2	140498	#N/A	#N/A	#N/A
ShSirt1_vs_ShScrambled	Q921E6	Q921E6	Eed	13626	#N/A	#N/A	#N/A
ShSirt1_vs_ShScrambled	Q921Q7	Q921Q7	Rin1	225870	#N/A	#N/A	#N/A
ShSirt1_vs_ShScrambled	Q921Y4	Q921Y4	Mfsd5	106073	#N/A	#N/A	#N/A
ShSirt1_vs_ShScrambled	Q921Z5	Q921Z5	Tnfaip8	106869	#N/A	#N/A	#N/A
ShSirt1_vs_ShScrambled	Q922Q2	Q922Q2	Riok1	71340	#N/A	#N/A	#N/A
ShSirt1_vs_ShScrambled	Q922R1	Q922R1	Q922R1	234678	#N/A	#N/A	#N/A
ShSirt1_vs_ShScrambled	Q922R5	Q922R5	Smek2	104570	#N/A	#N/A	#N/A
ShSirt1_vs_ShScrambled	Q923B1	Q923B1	Dbr1	83703	#N/A	#N/A	#N/A
ShSirt1_vs_ShScrambled	Q923K4	Q923K4	Gtpbp3	70359	#N/A	#N/A	#N/A
ShSirt1_vs_ShScrambled	Q924H2	Q924H2	Med15	94112	#N/A	#N/A	#N/A
ShSirt1_vs_ShScrambled	Q924T7	Q924T7	Rnf31	268749	#N/A	#N/A	#N/A
ShSirt1_vs_ShScrambled	Q925H1	Q925H1	Trps1		#N/A	#N/A	#N/A
ShSirt1_vs_ShScrambled	Q925N1	Q925N1	Sfxn4	94281	#N/A	#N/A	#N/A
ShSirt1_vs_ShScrambled	Q99J72	Q99J72	Apobec3	80287	#N/A	#N/A	#N/A
ShSirt1_vs_ShScrambled	Q99JP6	Q99JP6	Homer3	26558	#N/A	#N/A	#N/A
ShSirt1_vs_ShScrambled	Q99JT1	Q99JT1	Gatb	229487	#N/A	#N/A	#N/A
ShSirt1_vs_ShScrambled	Q99JZ7	Q99JZ7	Errfi1	74155	#N/A	#N/A	#N/A
ShSirt1_vs_ShScrambled	Q99K90	Q99K90	Tab2	68652	#N/A	#N/A	#N/A
ShSirt1_vs_ShScrambled	Q99KU0	Q99KU0	Vmp1	75909	#N/A	#N/A	#N/A
ShSirt1_vs_ShScrambled	Q99LB0	Q99LB0	Dnttip1	76233	#N/A	#N/A	#N/A
ShSirt1_vs_ShScrambled	Q99LC9	Q99LC9	Pex6	224824	#N/A	#N/A	#N/A
ShSirt1_vs_ShScrambled	Q99LE1	Q99LE1	Rilpl2	80291	#N/A	#N/A	#N/A
ShSirt1_vs_ShScrambled	Q99LG4	Q99LG4	Ttc5	219022	#N/A	#N/A	#N/A
ShSirt1_vs_ShScrambled	Q99LI9	Q99LI9	Clp1	98985	#N/A	#N/A	#N/A
ShSirt1_vs_ShScrambled	Q99LJ7	Q99LJ7	Rcbtb2	105670	#N/A	#N/A	#N/A
ShSirt1_vs_ShScrambled	Q99LN9	Q99LN9	Dohh	102115	#N/A	#N/A	#N/A
ShSirt1_vs_ShScrambled	Q99M96	Q99M96	St7	64213	#N/A	#N/A	#N/A
ShSirt1_vs_ShScrambled	Q99MQ4	Q99MQ4	Aspn	66695	#N/A	#N/A	#N/A
ShSirt1_vs_ShScrambled	Q99MV1	Q99MV1	Tdrd1	83561	#N/A	#N/A	#N/A
ShSirt1_vs_ShScrambled	Q99N89	Q99N89	Mrpl43		#N/A	#N/A	#N/A
ShSirt1_vs_ShScrambled	Q99NF3	Q99NF3	Cep41	83922	#N/A	#N/A	#N/A
ShSirt1_vs_ShScrambled	Q99NH2	Q99NH2	Pard3	93742	#N/A	#N/A	#N/A
ShSirt1_vs_ShScrambled	Q99P30	Q99P30	Nudt7	67528	#N/A	#N/A	#N/A
ShSirt1_vs_ShScrambled	Q99P31	Q99P31	Hspbp1	66245	#N/A	#N/A	#N/A
ShSirt1_vs_ShScrambled	Q99PJ2	Q99PJ2	Trim8	93679	#N/A	#N/A	#N/A
ShSirt1_vs_ShScrambled	Q99PP7	Q99PP7	Trim33	94093	#N/A	#N/A	#N/A
ShSirt1_vs_ShScrambled	Q99PQ2	Q99PQ2	Trim11	94091	#N/A	#N/A	#N/A
ShSirt1_vs_ShScrambled	Q9CPP0	Q9CPP0	Npm3	18150	#N/A	#N/A	#N/A
ShSirt1_vs_ShScrambled	Q9CPW7	Q9CPW7	Zmat2	66492	#N/A	#N/A	#N/A
ShSirt1_vs_ShScrambled	Q9CPY1	Q9CPY1	Mrpl51	66493	#N/A	#N/A	#N/A

ShSirt1_vs_ShScrambled	Q9CPY6	Q9CPY6	Gid4	66771	#N/A	#N/A	#N/A
ShSirt1_vs_ShScrambled	Q9CQ25	Q9CQ25	Mzt2	72083	#N/A	#N/A	#N/A
ShSirt1_vs_ShScrambled	Q9CQB7	Q9CQB7	Lyrm1	73919	#N/A	#N/A	#N/A
ShSirt1_vs_ShScrambled	Q9CQE7	Q9CQE7	Ergic3	66366	#N/A	#N/A	#N/A
ShSirt1_vs_ShScrambled	Q9CQI9	Q9CQI9	Med30	69790	#N/A	#N/A	#N/A
ShSirt1_vs_ShScrambled	Q9CQL7	Q9CQL7	Mrfap1	67568	#N/A	#N/A	#N/A
ShSirt1_vs_ShScrambled	Q9CQU8	Q9CQU8	Immp1l	66541	#N/A	#N/A	#N/A
ShSirt1_vs_ShScrambled	Q9CQV6	Q9CQV6	Map1lc3b	67443	#N/A	#N/A	#N/A
ShSirt1_vs_ShScrambled	Q9CQW0	Q9CQW0	Emc6	66048	#N/A	#N/A	#N/A
ShSirt1_vs_ShScrambled	Q9CR08	Q9CR08	Pop4	66161	#N/A	#N/A	#N/A
ShSirt1_vs_ShScrambled	Q9CR37	Q9CR37	Ppdpf	66496	#N/A	#N/A	#N/A
ShSirt1_vs_ShScrambled	Q9CR39	Q9CR39	Wdr45b	66840	#N/A	#N/A	#N/A
ShSirt1_vs_ShScrambled	Q9CR56	Q9CR56	Nkiras2	71966	#N/A	#N/A	#N/A
ShSirt1_vs_ShScrambled	Q9CR61	Q9CR61	Ndufb7	66916	#N/A	#N/A	#N/A
ShSirt1_vs_ShScrambled	Q9CRA8	Q9CRA8	Exosc5	27998	#N/A	#N/A	#N/A
ShSirt1_vs_ShScrambled	Q9CRC3	Q9CRC3	Q9CRC3	67290	#N/A	#N/A	#N/A
ShSirt1_vs_ShScrambled	Q9CRC6	Q9CRC6	Q9CRC6	66439	#N/A	#N/A	#N/A
ShSirt1_vs_ShScrambled	Q9CRY7	Q9CRY7	Gdpd1	66569	#N/A	#N/A	#N/A
ShSirt1_vs_ShScrambled	Q9CS00	Q9CS00	Cactin	70312	#N/A	#N/A	#N/A
ShSirt1_vs_ShScrambled	Q9CVD2	Q9CVD2	Atxn3	110616	#N/A	#N/A	#N/A
ShSirt1_vs_ShScrambled	Q9CWI3	Q9CWI3	Bccip	66165	#N/A	#N/A	#N/A
ShSirt1_vs_ShScrambled	Q9CWN7	Q9CWN7	Cnot11	52846	#N/A	#N/A	#N/A
ShSirt1_vs_ShScrambled	Q9CWR1	Q9CWR1	Wdr73	71968	#N/A	#N/A	#N/A
ShSirt1_vs_ShScrambled	Q9CWU2	Q9CWU2	Zdhhc13	243983	#N/A	#N/A	#N/A
ShSirt1_vs_ShScrambled	Q9CWW7	Q9CWW7	Cxxc1	74322	#N/A	#N/A	#N/A
ShSirt1_vs_ShScrambled	Q9CWY8	Q9CWY8	Rnaseh2a	69724	#N/A	#N/A	#N/A
ShSirt1_vs_ShScrambled	Q9CX53	Q9CX53	Gemin6	67242	#N/A	#N/A	#N/A
ShSirt1_vs_ShScrambled	Q9CX97	Q9CX97	Wdr55	67936	#N/A	#N/A	#N/A
ShSirt1_vs_ShScrambled	Q9CXC3	Q9CXC3	Mgme1	74528	#N/A	#N/A	#N/A
ShSirt1_vs_ShScrambled	Q9CXG3	Q9CXG3	Ppil4	67418	#N/A	#N/A	#N/A
ShSirt1_vs_ShScrambled	Q9CXK4	Q9CXK4	Slc50a1	19729	#N/A	#N/A	#N/A
ShSirt1_vs_ShScrambled	Q9CXS4	Q9CXS4	Cenpv	73139	#N/A	#N/A	#N/A
ShSirt1_vs_ShScrambled	Q9CXV9	Q9CXV9	Dcun1d5	76863	#N/A	#N/A	#N/A
ShSirt1_vs_ShScrambled	Q9CXY1	Q9CXY1	Tmem175	72392	#N/A	#N/A	#N/A
ShSirt1_vs_ShScrambled	Q9CY24	Q9CY24	Tmem179b	67706	#N/A	#N/A	#N/A
ShSirt1_vs_ShScrambled	Q9CYA0	Q9CYA0	Creld2	76737	#N/A	#N/A	#N/A
ShSirt1_vs_ShScrambled	Q9CYC6	Q9CYC6	Dcp2	70640	#N/A	#N/A	#N/A
ShSirt1_vs_ShScrambled	Q9CYQ7	Q9CYQ7	Narf	67608	#N/A	#N/A	#N/A
ShSirt1_vs_ShScrambled	Q9CZ83	Q9CZ83	Mrpl55	67212	#N/A	#N/A	#N/A
ShSirt1_vs_ShScrambled	Q9CZ91	Q9CZ91	Srfbp1	67222	#N/A	#N/A	#N/A
ShSirt1_vs_ShScrambled	Q9CZH7	Q9CZH7	Mxra7	67622	#N/A	#N/A	#N/A
ShSirt1_vs_ShScrambled	Q9CZL2	Q9CZL2	Q9CZL2	70617	#N/A	#N/A	#N/A
ShSirt1_vs_ShScrambled	Q9CZP0	Q9CZP0	Ufsp1	70240	#N/A	#N/A	#N/A
ShSirt1_vs_ShScrambled	Q9CZT6	Q9CZT6	Cmss1	66497	#N/A	#N/A	#N/A
ShSirt1_vs_ShScrambled	Q9D009	Q9D009	Lipt2	67164	#N/A	#N/A	#N/A
ShSirt1_vs_ShScrambled	Q9D061	Q9D061	Acbd6	72482	#N/A	#N/A	#N/A
ShSirt1_vs_ShScrambled	Q9D0D3	Q9D0D3	Mtpap	67440	#N/A	#N/A	#N/A
ShSirt1_vs_ShScrambled	Q9D0Z3	Q9D0Z3	Tmem53	68777	#N/A	#N/A	#N/A
ShSirt1_vs_ShScrambled	Q9D114	Q9D114	Hddc3	68695	#N/A	#N/A	#N/A
ShSirt1_vs_ShScrambled	Q9D142	Q9D142	Nudt14	66174	#N/A	#N/A	#N/A
ShSirt1_vs_ShScrambled	Q9D1C9	Q9D1C9	Rrp7a	74778	#N/A	#N/A	#N/A
ShSirt1_vs_ShScrambled	Q9D1G5	Q9D1G5	Lrrc57		#N/A	#N/A	#N/A
ShSirt1_vs_ShScrambled	Q9D1H6	Q9D1H6	Ndufaf4	68493	#N/A	#N/A	#N/A
ShSirt1_vs_ShScrambled	Q9D1Q4	Q9D1Q4	Dpm3	68563	#N/A	#N/A	#N/A
ShSirt1_vs_ShScrambled	Q9D1R2	Q9D1R2	Kti12	100087	#N/A	#N/A	#N/A
ShSirt1_vs_ShScrambled	Q9D219	Q9D219	Bcl9	77578	#N/A	#N/A	#N/A
ShSirt1_vs_ShScrambled	Q9D2U9	Q9D2U9;Q8(	Hist3h2ba		#N/A	#N/A	#N/A
ShSirt1_vs_ShScrambled	Q9D2V5	Q9D2V5	Aar2	68295	#N/A	#N/A	#N/A
ShSirt1_vs_ShScrambled	Q9D2X5	Q9D2X5	Mau2	74549	#N/A	#N/A	#N/A
ShSirt1_vs_ShScrambled	Q9D3D0	Q9D3D0	Ttpal	76080	#N/A	#N/A	#N/A
ShSirt1_vs_ShScrambled	Q9D4F2	Q9D4F2	Ppapdc2	74411	#N/A	#N/A	#N/A
ShSirt1_vs_ShScrambled	Q9D4F8	Q9D4F8	Tubgcp4	51885	#N/A	#N/A	#N/A
ShSirt1_vs_ShScrambled	Q9D4H9	Q9D4H9	Phf14	75725	#N/A	#N/A	#N/A

ShSirt1_vs_ShScrambled	Q9D4J7	Q9D4J7	Phf6	70998	#N/A	#N/A	#N/A
ShSirt1_vs_ShScrambled	Q9D4V0	Q9D4V0	Etnk1	75320	#N/A	#N/A	#N/A
ShSirt1_vs_ShScrambled	Q9D6K7	Q9D6K7	Ttc33	67515	#N/A	#N/A	#N/A
ShSirt1_vs_ShScrambled	Q9D6K9	Q9D6K9	Cers5	71949	#N/A	#N/A	#N/A
ShSirt1_vs_ShScrambled	Q9D6W8	Q9D6W8	Q9D6W8	71923	#N/A	#N/A	#N/A
ShSirt1_vs_ShScrambled	Q9D6Z0	Q9D6Z0	Alkbh7	66400	#N/A	#N/A	#N/A
ShSirt1_vs_ShScrambled	Q9D711	Q9D711	Pir	69656	#N/A	#N/A	#N/A
ShSirt1_vs_ShScrambled	Q9D735	Q9D735	Q9D735	68544	#N/A	#N/A	#N/A
ShSirt1_vs_ShScrambled	Q9D742	Q9D742	Ap5s1	69596	#N/A	#N/A	#N/A
ShSirt1_vs_ShScrambled	Q9D753	Q9D753	Exosc8	69639	#N/A	#N/A	#N/A
ShSirt1_vs_ShScrambled	Q9D787	Q9D787	Ppil2	66053	#N/A	#N/A	#N/A
ShSirt1_vs_ShScrambled	Q9D7E4	Q9D7E4	Q9D7E4	66374	#N/A	#N/A	#N/A
ShSirt1_vs_ShScrambled	Q9D7S9	Q9D7S9	Chmp5	76959	#N/A	#N/A	#N/A
ShSirt1_vs_ShScrambled	Q9D7Z3	Q9D7Z3	Nol7	70078	#N/A	#N/A	#N/A
ShSirt1_vs_ShScrambled	Q9D842	Q9D842	Aplf	72103	#N/A	#N/A	#N/A
ShSirt1_vs_ShScrambled	Q9D8M7	Q9D8M7	Phf10	72057	#N/A	#N/A	#N/A
ShSirt1_vs_ShScrambled	Q9D8V7	Q9D8V7	Sec11c	66286	#N/A	#N/A	#N/A
ShSirt1_vs_ShScrambled	Q9D8X1	Q9D8X1	Cutc	66388	#N/A	#N/A	#N/A
ShSirt1_vs_ShScrambled	Q9D8Z1	Q9D8Z1	Ascc1	69090	#N/A	#N/A	#N/A
ShSirt1_vs_ShScrambled	Q9D958	Q9D958	Spcs1	69019	#N/A	#N/A	#N/A
ShSirt1_vs_ShScrambled	Q9D9K3	Q9D9K3	Aven	74268	#N/A	#N/A	#N/A
ShSirt1_vs_ShScrambled	Q9D9M2	Q9D9M2;P62	Usp12		#N/A	#N/A	#N/A
ShSirt1_vs_ShScrambled	Q9D9M5	Q9D9M5	Phospho2	73373	#N/A	#N/A	#N/A
ShSirt1_vs_ShScrambled	Q9DAI2	Q9DAI2	Ift22	67286	#N/A	#N/A	#N/A
ShSirt1_vs_ShScrambled	Q9DB70	Q9DB70	Fundc1	72018	#N/A	#N/A	#N/A
ShSirt1_vs_ShScrambled	Q9DB85	Q9DB85	Rrp8	101867	#N/A	#N/A	#N/A
ShSirt1_vs_ShScrambled	Q9DB90	Q9DB90	Smg9	71997	#N/A	#N/A	#N/A
ShSirt1_vs_ShScrambled	Q9DB96	Q9DB96	Ngdn	68966	#N/A	#N/A	#N/A
ShSirt1_vs_ShScrambled	Q9DBA6	Q9DBA6	Tysnd1	71767	#N/A	#N/A	#N/A
ShSirt1_vs_ShScrambled	Q9DBE9	Q9DBE9	Ftsj3	56095	#N/A	#N/A	#N/A
ShSirt1_vs_ShScrambled	Q9DBM2	Q9DBM2	Ehhadh	74147	#N/A	#N/A	#N/A
ShSirt1_vs_ShScrambled	Q9DBS9	Q9DBS9	Osbpl3	71720	#N/A	#N/A	#N/A
ShSirt1_vs_ShScrambled	Q9DBU3	Q9DBU3	Riok3	66878	#N/A	#N/A	#N/A
ShSirt1_vs_ShScrambled	Q9DBX2	Q9DBX2	Pdcl	67466	#N/A	#N/A	#N/A
ShSirt1_vs_ShScrambled	Q9DCD5	Q9DCD5	Tjap1	74094	#N/A	#N/A	#N/A
ShSirt1_vs_ShScrambled	Q9DCH2	Q9DCH2	Pop7	74097	#N/A	#N/A	#N/A
ShSirt1_vs_ShScrambled	Q9DCI9	Q9DCI9	Mrpl32	75398	#N/A	#N/A	#N/A
ShSirt1_vs_ShScrambled	Q9DCL2	Q9DCL2	Fam96a	68250	#N/A	#N/A	#N/A
ShSirt1_vs_ShScrambled	Q9DCM0	Q9DCM0	Ethe1	66071	#N/A	#N/A	#N/A
ShSirt1_vs_ShScrambled	Q9DCN7	Q9DCN7	Rnft1	76892	#N/A	#N/A	#N/A
ShSirt1_vs_ShScrambled	Q9DD02	Q9DD02	L7rn6	67669	#N/A	#N/A	#N/A
ShSirt1_vs_ShScrambled	Q9EP82	Q9EP82	Wdr4	57773	#N/A	#N/A	#N/A
ShSirt1_vs_ShScrambled	Q9EPL0	Q9EPL0	Xylt2	217119	#N/A	#N/A	#N/A
ShSirt1_vs_ShScrambled	Q9EPQ8	Q9EPQ8	Tcf20	21411	#N/A	#N/A	#N/A
ShSirt1_vs_ShScrambled	Q9EQJ0	Q9EQJ0	Tpcn1	252972	#N/A	#N/A	#N/A
ShSirt1_vs_ShScrambled	Q9EQY0	Q9EQY0	Ern1	78943	#N/A	#N/A	#N/A
ShSirt1_vs_ShScrambled	Q9ER81	Q9ER81	Tor1aip2.1	240832	#N/A	#N/A	#N/A
ShSirt1_vs_ShScrambled	Q9ERR1	Q9ERR1	Ndel1	83431	#N/A	#N/A	#N/A
ShSirt1_vs_ShScrambled	Q9ERV1	Q9ERV1	Mkrn2	67027	#N/A	#N/A	#N/A
ShSirt1_vs_ShScrambled	Q9ES70	Q9ES70	Nek6	59126	#N/A	#N/A	#N/A
ShSirt1_vs_ShScrambled	Q9EST3	Q9EST3	Eif4enif1	74203	#N/A	#N/A	#N/A
ShSirt1_vs_ShScrambled	Q9ET26	Q9ET26	Rnf114	81018	#N/A	#N/A	#N/A
ShSirt1_vs_ShScrambled	Q9ET47	Q9ET47	Espn	56226	#N/A	#N/A	#N/A
ShSirt1_vs_ShScrambled	Q9JHK4	Q9JHK4	Rabggta	56187	#N/A	#N/A	#N/A
ShSirt1_vs_ShScrambled	Q9JHZ2	Q9JHZ2	Ankh	11732	#N/A	#N/A	#N/A
ShSirt1_vs_ShScrambled	Q9JI99	Q9JI99	Sgpp1	81535	#N/A	#N/A	#N/A
ShSirt1_vs_ShScrambled	Q9JIB4	Q9JIB4	Gtf2h2	23894	#N/A	#N/A	#N/A
ShSirt1_vs_ShScrambled	Q9JJ06	Q9JJ06	C1galt1	94192	#N/A	#N/A	#N/A
ShSirt1_vs_ShScrambled	Q9JJ89	Q9JJ89	Ccdc86	108673	#N/A	#N/A	#N/A
ShSirt1_vs_ShScrambled	Q9JJC6	Q9JJC6	Rilpl1	75695	#N/A	#N/A	#N/A
ShSirt1_vs_ShScrambled	Q9JJG9	Q9JJG9	Noa1	56412	#N/A	#N/A	#N/A
ShSirt1_vs_ShScrambled	Q9JK83	Q9JK83;Q9Z	Pard6b		#N/A	#N/A	#N/A
ShSirt1_vs_ShScrambled	Q9JKX4	Q9JKX4	Aatf	56321	#N/A	#N/A	#N/A

ShSirt1_vs_ShScrambled	Q9JKY5	Q9JKY5	Hip1r	29816	#N/A	#N/A	#N/A
ShSirt1_vs_ShScrambled	Q9JL19	Q9JL19	Ncoa6		#N/A	#N/A	#N/A
ShSirt1_vs_ShScrambled	Q9JLR9	Q9JLR9	Higd1a	56295	#N/A	#N/A	#N/A
ShSirt1_vs_ShScrambled	Q9JM05	Q9JM05	Pias4	59004	#N/A	#N/A	#N/A
ShSirt1_vs_ShScrambled	Q9JM13	Q9JM13	Rabgef1	56715	#N/A	#N/A	#N/A
ShSirt1_vs_ShScrambled	Q9JM93	Q9JM93	Arl6ip4	65105	#N/A	#N/A	#N/A
ShSirt1_vs_ShScrambled	Q9JME7	Q9JME7	Trappc2l	59005	#N/A	#N/A	#N/A
ShSirt1_vs_ShScrambled	Q9JMK2	Q9JMK2	Csnk1e	27373	#N/A	#N/A	#N/A
ShSirt1_vs_ShScrambled	Q9QWT9	Q9QWT9	Kifc1	16580; 10	#N/A	#N/A	#N/A
ShSirt1_vs_ShScrambled	Q9QX11	Q9QX11	Cyth1	19157	#N/A	#N/A	#N/A
ShSirt1_vs_ShScrambled	Q9QXA1	Q9QXA1	Cyhr1	54151	#N/A	#N/A	#N/A
ShSirt1_vs_ShScrambled	Q9QXS6	Q9QXS6	Dbn1	56320	#N/A	#N/A	#N/A
ShSirt1_vs_ShScrambled	Q9QYI5	Q9QYI5	Dnajb2	56812	#N/A	#N/A	#N/A
ShSirt1_vs_ShScrambled	Q9QYI6	Q9QYI6	Dnajb9	27362	#N/A	#N/A	#N/A
ShSirt1_vs_ShScrambled	Q9QZ05	Q9QZ05	Eif2ak4	27103	#N/A	#N/A	#N/A
ShSirt1_vs_ShScrambled	Q9QZF2	Q9QZF2	Gpc1	14733	#N/A	#N/A	#N/A
ShSirt1_vs_ShScrambled	Q9QZW0	Q9QZW0	Atp11c	320940	#N/A	#N/A	#N/A
ShSirt1_vs_ShScrambled	Q9R020	Q9R020	Zranb2	53861	#N/A	#N/A	#N/A
ShSirt1_vs_ShScrambled	Q9R045	Q9R045	Angptl2	26360	#N/A	#N/A	#N/A
ShSirt1_vs_ShScrambled	Q9R049	Q9R049	Amfr	23802	#N/A	#N/A	#N/A
ShSirt1_vs_ShScrambled	Q9R060	Q9R060	Nubp1	26425	#N/A	#N/A	#N/A
ShSirt1_vs_ShScrambled	Q9R0C8	Q9R0C8	Vav3	57257	#N/A	#N/A	#N/A
ShSirt1_vs_ShScrambled	Q9R0L7	Q9R0L7	Akap8l		#N/A	#N/A	#N/A
ShSirt1_vs_ShScrambled	Q9R0X0	Q9R0X0	Med20	56771	#N/A	#N/A	#N/A
ShSirt1_vs_ShScrambled	Q9R1E6	Q9R1E6	Enpp2	18606	#N/A	#N/A	#N/A
ShSirt1_vs_ShScrambled	Q9R1Q6	Q9R1Q6	Tmem176b	65963	#N/A	#N/A	#N/A
ShSirt1_vs_ShScrambled	Q9R1V7	Q9R1V7	Adam23	23792	#N/A	#N/A	#N/A
ShSirt1_vs_ShScrambled	Q9R1X5	Q9R1X5	Abcc5	27416	#N/A	#N/A	#N/A
ShSirt1_vs_ShScrambled	Q9WTN0	Q9WTN0	Ggps1	14593	#N/A	#N/A	#N/A
ShSirt1_vs_ShScrambled	Q9WTP2	Q9WTP2	Spry4	24066	#N/A	#N/A	#N/A
ShSirt1_vs_ShScrambled	Q9WTS2	Q9WTS2	Fut8	53618	#N/A	#N/A	#N/A
ShSirt1_vs_ShScrambled	Q9WTU0	Q9WTU0	Phf2	18676	#N/A	#N/A	#N/A
ShSirt1_vs_ShScrambled	Q9WTV7	Q9WTV7	Rlim	19820	#N/A	#N/A	#N/A
ShSirt1_vs_ShScrambled	Q9WUU8	Q9WUU8	Tnip1	57783	#N/A	#N/A	#N/A
ShSirt1_vs_ShScrambled	Q9WV95	Q9WV95	Phlda3	27280	#N/A	#N/A	#N/A
ShSirt1_vs_ShScrambled	Q9WVH4	Q9WVH4	Foxo3	56484	#N/A	#N/A	#N/A
ShSirt1_vs_ShScrambled	Q9WVH6	Q9WVH6	Angpt4	11602	#N/A	#N/A	#N/A
ShSirt1_vs_ShScrambled	Q9WVL2	Q9WVL2	Stat2		#N/A	#N/A	#N/A
ShSirt1_vs_ShScrambled	Q9Z0L8	Q9Z0L8	Ggh	14590	#N/A	#N/A	#N/A
ShSirt1_vs_ShScrambled	Q9Z104	Q9Z104	Hmg20b	15353	#N/A	#N/A	#N/A
ShSirt1_vs_ShScrambled	Q9Z172	Q9Z172	Sumo3	20610	#N/A	#N/A	#N/A
ShSirt1_vs_ShScrambled	Q9Z1J1	Q9Z1J1	Tcf7l1	21415	#N/A	#N/A	#N/A
ShSirt1_vs_ShScrambled	Q9Z1K6	Q9Z1K6	Arih2	23807	#N/A	#N/A	#N/A
ShSirt1_vs_ShScrambled	Q9Z1R4	Q9Z1R4	D17h6s53e	114585	#N/A	#N/A	#N/A
ShSirt1_vs_ShScrambled	Q9Z1S8	Q9Z1S8	Gab2		#N/A	#N/A	#N/A
ShSirt1_vs_ShScrambled	Q9Z255	Q9Z255	Ube2a	22209	#N/A	#N/A	#N/A
ShSirt1_vs_ShScrambled	Q9Z2D8	Q9Z2D8	Mbd3	17192	#N/A	#N/A	#N/A
ShSirt1_vs_ShScrambled	Q9Z2E1	Q9Z2E1	Mbd2	17191	#N/A	#N/A	#N/A
ShSirt1_vs_ShScrambled	Q9Z2G0	Q9Z2G0	Fem1b	14155	#N/A	#N/A	#N/A
ShSirt1_vs_ShScrambled	Q9Z2V6	Q9Z2V6	Hdac5		#N/A	#N/A	#N/A
ShSirt1_vs_ShScrambled	Q9Z2Z9	Q9Z2Z9	Gfpt2	14584	#N/A	#N/A	#N/A
